# Supplementary material for: Catalytic stereodivergent allylic alkylation of 2-acylimidazoles for natural product synthesis
Source: Nat Commun. 2023 Dec 8;14:8118. doi: 10.1038/s41467-023-43986-6 (PMC10709448; doi:10.1038/s41467-023-43986-6)
Supplement: Supplementary file 1 — Supplementary information [file 41467_2023_43986_MOESM1_ESM.pdf]

Supplementary Information

Catalytic stereodivergent allylic alkylation of 2-acylimidazoles for the collective synthesis of natural products

Ruimin Lu[1,2], Qinglin Zhang[1,2], Chang Guo[1]✉

[1]Hefei National Research Center for Physical Sciences at the Microscale and Department of Chemistry, University of Science and Technology of China, Hefei, 230026, China.

[2]These authors contributed equally: Ruimin Lu, Qinglin Zhang.

✉Corresponding author: e-mail: guochang@ustc.edu.cn

CONTENTS:

1. Supplementary Note 2
2. Supplementary Methods 3
3. Supplementary Figure 49
4. Supplementary References..209

1. Supplementary Note

Unless stated otherwise, all reactions were carried out in flame-dried glassware under a dry argon atmosphere. All solvents are purified and dried according to standard methods prior to use.

^{1}H NMR and ^{13}C NMR spectra were recorded at 25 ºC on Bruker Advance 400M NMR spectrometers, Bruker Advance 500M NMR spectrometers and JEOL 600M NMR spectrometers. Chemical shifts for ^{1}H NMR spectra are reported as δ in units of parts per million (ppm) downfield from $SiMe_4$ (δ 0.00) and relative to the signal of chloroform-*d* (δ 7.26, singlet). Multiplicities were given as: s (singlet); d (doublet); t (triplet); q (quartet); dd (doublet of doublets); dt (doublet of triplets); m (multiplets), etc. Coupling constants are reported as a *J* value in Hz. ^{13}C NMR spectra are reported as δ in units of parts per million (ppm) downfield from $SiMe_4$ (δ 0.00) and relative to the signal of chloroform-*d* (δ 77.16, triplet). High-resolution mass spectral analysis (HRMS) was performed on Waters XEVO G2 Q-TOF. Optical rotations were determined at 589 nm (sodium D line) by using a Perkin-Elmer-343 polarimeter. The measurement of enantiomeric excesses was performed on Waters-Alliance (2998, Photodiode Array Detector). CHIRALCEL IA, IBN-5, IC, IE, IG, AD-H, OD-H, and OJ-H columns were purchased from Daicel Chemical Industries, LTD.

2. Supplementary Methods

2-Acylimidazoles **1** were synthesized according to the literature.[1,2] The *N*-arylimidazole and Weinreb amides were prepared following a reported procedure.[1,2]

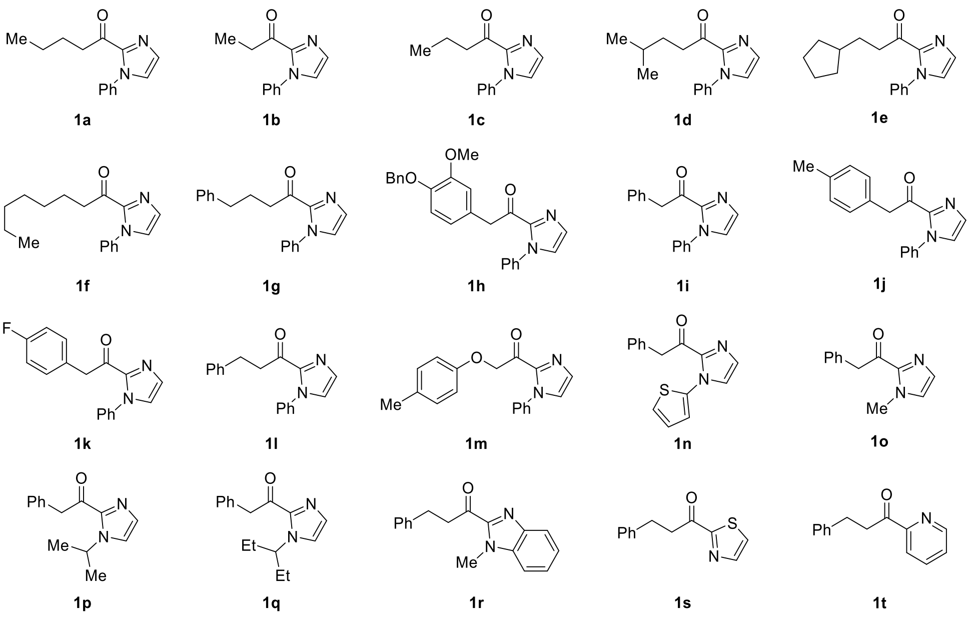

Synthesis of 2-acyl imidazoles (1a-1g, 1i-1t)

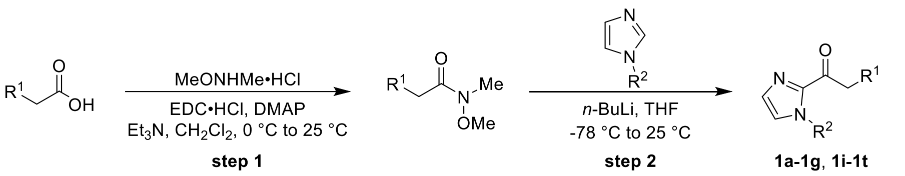

Step 1: To a solution of carboxylic acid (20 mmol, 1.0 equiv.), EDC•HCl (26 mmol, 1.3 equiv.), and *N,O*-dimethylhydroxylamine hydrochloride (26 mmol, 1.3 equiv.) in CH_2Cl_2 (80 mL) at 0 °C was added DMAP (2 mmol, 0.10 equiv.), followed by Et_3N (40 mmol, 2.0 equiv.). The resulting reaction mixture was stirred at 25 ℃ until complete consumption of the starting material (monitored by TLC). HCl (1.0 M) was added to quench the reaction. The layers were separated and the aqueous layer was extracted twice with CH_2Cl_2 (3 x 20 mL). The combined organic solution was washed with saturated $NaHCO_3$, dried over $MgSO_4$, filtered, and concentrated under reduced pressure. The crude Weinreb amide was directly used in the next step without further purification.

Step 2: To a solution of N-aryl imidazole (11 mmol, 1.1 equiv.) in THF (10 mL) at -78 °C was added dropwise *n*-BuLi (11 mmol, 1.1 equiv., 2.50 M in *n*-hexane). The reaction mixture was stirred at -78 °C for 30 min and 25 ℃ for another 30 min. The solution was cooled again to -78 °C and a solution of Weinreb amide (10 mmol, 1.0 equiv.) in THF (2 mL) was added dropwise to the lithium solution at -78 °C. The reaction mixture was slowly warmed to ambient temperature and stirred at 25 °C for overnight to complete the consumption of the starting material (monitored by TLC). The reaction was then quenched with saturated aqueous NH_4Cl solution and diluted with EtOAc (20 mL). The layers were separated and the aqueous layer was extracted twice with EtOAc (3 x 10 mL). The combined organic

solution was dried over $MgSO_4$, filtered, and concentrated under reduced pressure. The residue was chromatographed through silica gel to afford 2-acylimidazoles **1a-1g**, **1i-1t**.

Synthesis of 2-acyl imidazoles (1h)

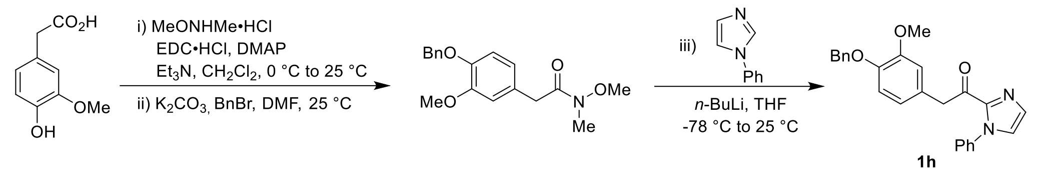

To a solution of carboxylic acid (20 mmol, 1.0 equiv.), EDC•HCl (26 mmol, 1.3 equiv.) and *N,O*-dimethylhydroxylamine hydrochloride (26 mmol, 1.3 equiv.) in CH_2Cl_2 (80 mL) at 0 °C was added DMAP (2 mmol, 0.10 equiv.), followed by Et_3N (40 mmol, 2.0 equiv.). The resulting reaction mixture was stirred at 25 °C until complete consumption of the starting material (monitored by TLC). HCl (1.0 M) was added to quench the reaction. The layers were separated and the aqueous layer was extracted twice with CH_2Cl_2 (3 x 20 mL). The combined organic solution was washed with saturated $NaHCO_3$, dried over $MgSO_4$, filtered, and concentrated under reduced pressure. The crude product was directly dissolved in DMF (60 mL) and then added K_2CO_3 (40 mmol 2.0 equiv.) and BnBr (40mmol, 2.0equiv.). The resulting reaction mixture was stirred at 25 °C until complete consumption of the starting material (monitored by TLC). H_2O (60mL) was added to quench the reaction. The layers were separated and the aqueous layer was extracted twice with EA (3 x 50 mL). The combined organic solution was dried over $MgSO_4$, filtered, and concentrated under reduced pressure. The residue was chromatographed through silica gel to afford Weinreb amide. To a solution of N-aryl imidazole (11 mmol, 1.1 equiv.) in THF (10 mL) at -78 °C was added dropwise *n*-BuLi (11 mmol, 1.1 equiv., 2.50 M in *n*-hexane). The reaction mixture was stirred at -78 °C for 30 min and 25 °C for another 30 min. The solution was cooled again to -78 °C and a solution of Weinreb amide (10 mmol, 1.0 equiv.) in THF (2 mL) was added dropwise to the lithium solution at -78 °C. The reaction mixture was slowly warmed to ambient temperature and stirred at 25 °C for overnight to complete the consumption of the starting material (monitored by TLC). The reaction was then quenched with saturated aqueous NH_4Cl solution and diluted with EtOAc (20 mL). The layers were separated and the aqueous layer was extracted twice with EtOAc (3 x 10 mL). The combined organic solution was dried over $MgSO_4$, filtered, and concentrated under reduced pressure. The residue was chromatographed through silica gel to afford 2-acylimidazole **1h**.

Synthesis of cinnamyl bromides (2a-2ai)

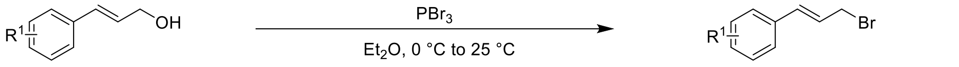

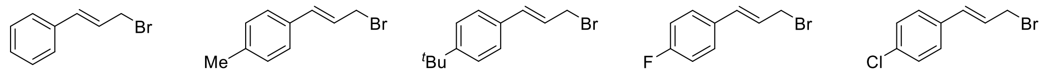

2a 2aa 2ab 2ac 2ad

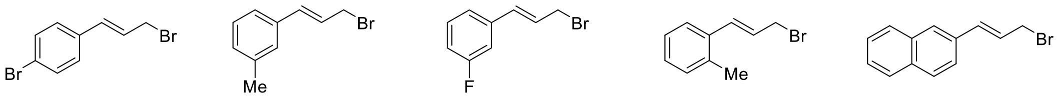

2ae 2af 2ag 2ah 2ai

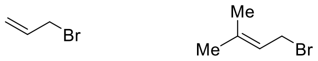

2aj 2ak

A solution of allylic alcohol (1.0 equiv.) in Et_2O (0.33 M) was cooled to 0 °C and treated with PBr_3 (0.4 equiv.). After stirred for 1 hour, the reaction was quenched by the dropwise addition of aq. sat. $NaHCO_3$ (equal volume), and then the mixture was allowed to warm to room temperature. The layers were separated, and the aqueous layer was extracted with Et_2O (2 × equal volume). The residue was washed with brine (equal volume), dried over $MgSO_4$, filtered, and concentrated under reduced pressure to give allylic bromides, which were used directly.[3,4]

Synthesis of Allyl Carbonates (2c-2o)

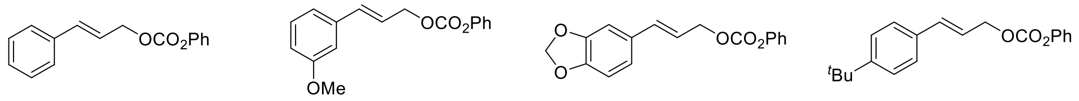

2c 2d 2e 2f

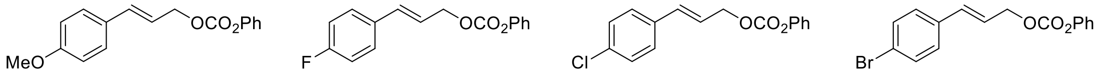

2g 2h 2i 2j

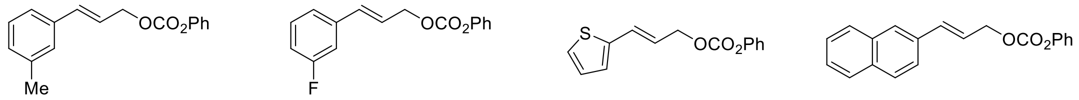

2k 2l 2m 2n

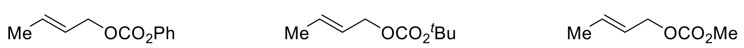

2o 2p 2q

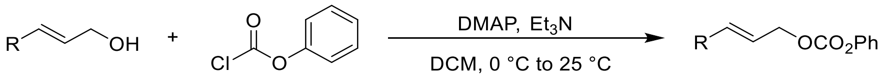

Allyl alcohol (10 mmol, 1.0 equiv.) and triethylamine (30 mmol, 3.0 equiv.) were dissolved in anhydrous CH_2Cl_2 (30 mL) and phenyl chloroformate (15 mmol, 1.5 equiv.) was added dropwise at 0 °C. The reaction mixture was allowed to warm to room temperature, stirred for 1 hour at room temperature

and quenched by the addition of sat. aq. $NaHCO_3$ (equal volume). The layers were separated and the aqueous phase was extracted with CH_2Cl_2 (3 × equal volume). The combined organic layers were washed with aq. HCl (1 M, 2 × equal volume), dried over $MgSO_4$, filtered, and concentrated in vacuo. The crude product was purified by column chromatography over silica gel to afford substrates **2c-2o**.[5]

Synthesis of (E)-but-2-en-1-yl tert-butyl carbonate (2p)

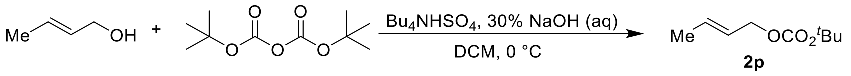

To a solution of the corresponding cinnamyl alcohol (1.0 equiv.) in dichloromethane(reaction concentration~2.2 M) were added Boc anhydride (1.4 equiv.) and Bu_4NHSO_4(0.03 equiv.). The solution was cooled to 0 °Cand 30% NaOH (aq) (0.25 mL/mmol alcohol) was added dropwise with vigorous stirring. The reaction was followed by TLC. After~2h, the reaction was diluted with dichloromethane (50 mL) and washed with water and brine. The organic layer was dried over Na_2SO_4, filtered, and concentrated. Column chromatography (5−10% EtOAc in hexanes) afforded the cinnamyl carbonates **2p**.

Synthesis of (E)-but-2-en-1-yl methyl carbonate (2q)

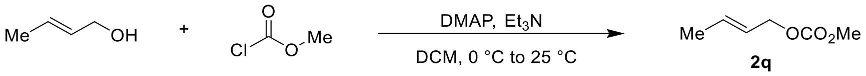

Crotonyl alcohol (10 mmol, 1.0 equiv.) and triethylamine (30 mmol, 3.0 equiv.) were dissolved in anhydrous CH_2Cl_2 (30 mL) and methyl chloroformate (15 mmol, 1.5 equiv.) was added dropwise at 0 °C. The reaction mixture was allowed to warm to room temperature, stirred for 1 hour at room temperature and quenched by the addition of sat. aq. $NaHCO_3$ (equal volume). The layers were separated and the aqueous phase was extracted with CH_2Cl_2 (3 × equal volume). The combined organic layers were washed with aq. HCl (1 M, 2 × equal volume), dried over $MgSO_4$, filtered, and concentrated in vacuo. The crude product was purified by column chromatography over silica gel to afford substrates **2q**.

Synthesis and characterization of products

General procedure for the synthesis of racemic products

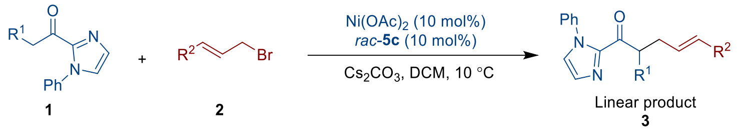

A 10 mL Schlenk tube was charged with **1** (0.1 mmol, 1.0 equiv.), **2** (0.15 mmol, 1.5 equiv.), racemic Ni catalyst (0.01 mmol, 0.1 equiv.), Cs_2CO_3 (0.2 mmol, 2.0 equiv.), and DCM (2 mL) under argon atmosphere. The reaction mixture was stirred at 10 ℃ until complete consumption of the substrate (monitored by TLC). The solution was diluted with dichloromethane and then filtered with celite. The residue was purified by flash column chromatography on silica gel to afford the desired product **3**.

The racemic nickel catalyst was prepared following a reported procedure.[6] A mixture of $Ni(OAc)_2·4H_2O$ (0.1 mmol, 1.0 equiv.) and *rac*-**5c** (0.1 mmol, 1.0 equiv.) in acetonitrile (2 mL) were heated to 92 ℃ under reflux for 5 hours. Subsequent to solvent removal, the residue was dissolved in dichloromethane and then evaporated to dryness. The preformed Ni-complex can be used directly.

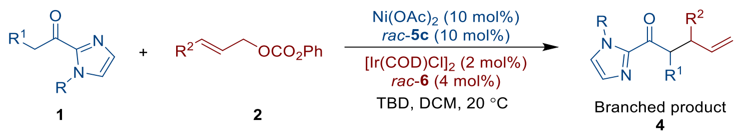

In a 10 mL Schlenk tube, $[Ir(COD)Cl]_2$ (0.002 mmol, 0.02 equiv.), *rac*-**6** (0.004 mmol, 0.04 equiv.), TBD (0.01mmol, 0.1 equiv.) were stirred in 1 mL DCM under argon at 25 °C for 10 min. **1** (0.1 mmol, 1.0 equiv.), allylic carbonate **2** (0.15 mmol, 1.5 equiv.), and racemic Ni catalyst (0.01 mmol, 0.1 equiv.) were then added successively and additional DCM (1 mL) was added. The mixture was stirred at 20 ℃. After the reaction was complete (monitored by TLC), the residue was purified by flash column chromatography on silica gel to afford the desired product **4**.

General procedure for the synthesis of chiral products.

General procedure for the synthesis of linear products

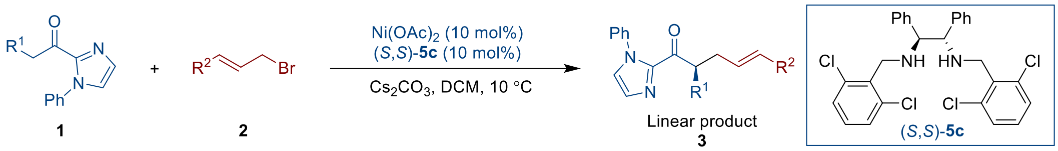

To a flame-dried and argon-purged Schlenk tube were added 2-acyl imidazoles **1** (0.1 mmol, 1.0 equiv.), allylic bromide derivatives **2** (0.15 mmol, 1.5 equiv.), Ni catalyst (0.01 mmol, 0.1 equiv.), Cs_2CO_3 (0.2 mmol, 2.0 equiv.), and DCM (2 mL). The reaction mixture was stirred at 10 °C until complete consumption of the substrates (monitored by TLC). The solution was diluted with dichloromethane and then filtered with celite. The residue was purified by flash column chromatography on silica gel to afford the desired product **3**.

(*R*,*E*)-5-phenyl-1-(1-phenyl-1H-imidazol-2-yl)-2-propylpent-4-en-1-one (3a)

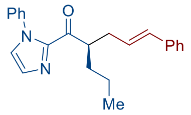

Reaction time: 1.5 d. Yield: 80%. **^{1}H NMR (400 MHz, $CDCl_3$)** δ 7.48 – 7.35 (m, 3H), 7.33 – 7.23 (m, 5H), 7.22 – 7.16 (m, 3H), 7.16 – 7.13 (m, 1H), 6.35 (d, *J* = 15.8 Hz, 1H), 6.25 – 6.12 (m, 1H), 4.15 – 4.02 (m, 1H), 2.66 – 2.52 (m, 1H), 2.51 – 2.39 (m, 1H), 1.83 – 1.70 (m, 1H), 1.59 – 1.47 (m, 1H), 1.42 – 1.26 (m, 2H), 0.89 (t, *J* = 7.3 Hz, 3H). **^{13}C NMR (125 MHz, $CDCl_3$)** δ 194.81, 143.51, 138.61, 137.62, 131.72, 129.67, 129.06, 128.75, 128.56, 128.03, 127.28, 127.11, 126.18, 125.95, 46.68, 35.90, 33.92, 20.78, 14.41. **ESI-MS:** calculated $[C_{23}H_{24}N_2O + H]^+$: 345.1961, found: 345.1971. $[\alpha]^{20}_D$= -96.1 (c = 0.76, CH_2Cl_2). The product was analyzed by HPLC to determine the enantiomeric excess: 95% e.e. (CHIRALPAK IBN-5, hexane/*i*-PrOH = 99/1, detector: 254 nm, T = 25 ℃, flow rate: 1 mL/min), t_1 (minor) = 10.61 min, t_2 (major) = 11.53 min.

(*R*,*E*)-2-methyl-5-phenyl-1-(1-phenyl-1H-imidazol-2-yl)pent-4-en-1-one (3b)

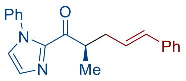

Reaction time: 2 d. Yield: 88%. **^{1}H NMR (500 MHz, $CDCl_3$)** δ 7.46 – 7.38 (m, 3H), 7.30 – 7.24 (m, 5H), 7.24 – 7.15 (m, 4H), 6.38 (d, *J* = 15.8 Hz, 1H), 6.25 – 6.14 (m, 1H), 4.14 – 4.00 (m, 1H), 2.72 – 2.60 (m, 1H), 2.43 – 2.32 (m, 1H), 1.23 (d, *J* = 7.0 Hz, 3H). **^{13}C NMR (100 MHz, $CDCl_3$)** δ 194.77, 142.80, 138.56, 137.60, 131.92, 129.67, 129.07, 128.79, 128.56, 127.90, 127.26, 127.14, 126.17, 125.93, 41.61, 36.94, 16.80. **ESI-MS:** calculated $[C_{21}H_{20}N_2O + H]^+$: 317.1648, found: 317.1658. $[\alpha]^{20}_D$= -106.2 (c = 0.55, CH_2Cl_2). The product was analyzed by HPLC

to determine the enantiomeric excess: 92% e.e. (CHIRALPAK IG, hexane/*i*-PrOH = 95/5, detector: 276 nm, T = 25 °C, flow rate: 1 mL/min), t_1 (minor) = 13.62 min, t_2 (major) = 14.88 min.

(*R*,*E*)-2-ethyl-5-phenyl-1-(1-phenyl-1H-imidazol-2-yl)pent-4-en-1-one (3c)

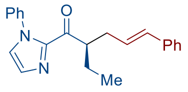

Reaction time: 3 d. Yield: 80%. **^{1}H NMR (400 MHz, CDCl$_3$)** δ 7.46 – 7.35 (m, 3H), 7.29 – 7.24 (m, 5H), 7.22 – 7.12 (m, 4H), 6.36 (d, *J* = 15.8 Hz, 1H), 6.25 – 6.11 (m, 1H), 4.08 – 3.94 (m, 1H), 2.66 – 2.54 (m, 1H), 2.52 – 2.39 (m, 1H), 1.90 – 1.73 (m, 1H), 1.70 – 1.57 (m, 1H), 0.92 (t, *J* = 7.4 Hz, 3H). **^{13}C NMR (100 MHz, CDCl$_3$)** δ 194.67, 143.63, 138.67, 137.68, 131.76, 129.67, 129.07, 128.75, 128.56, 128.06, 127.23, 127.11, 126.19, 125.98, 48.34, 35.25, 24.75, 11.86. **ESI-MS:** calculated $[C_{22}H_{22}N_2O + H]^+$: 331.1805, found: 331.1811. $[\alpha]^{20}_D$= -98.6 (c = 0.77, CH_2Cl_2). The product was analyzed by HPLC to determine the enantiomeric excess: 93% e.e. (CHIRALPAK IBN-5, hexane/*i*-PrOH = 95/5, detector: 248 nm, T = 25 °C, flow rate: 1 mL/min), t_1 (minor) = 6.63 min, t_2 (major) = 6.96 min.

(*R*,*E*)-2-isobutyl-5-phenyl-1-(1-phenyl-1H-imidazol-2-yl)pent-4-en-1-one (3d)

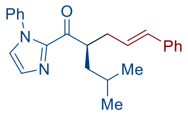

Reaction time: 2 d. Yield: 78%. **^{1}H NMR (400 MHz, CDCl$_3$)** δ 7.36 – 7.29 (m, 3H), 7.22 – 7.16 (m, 5H), 7.14 – 7.05 (m, 4H), 6.27 (d, *J* = 15.8 Hz, 1H), 6.17 – 6.03 (m, 1H), 4.18 – 4.06 (m, 1H), 2.56 – 2.43 (m, 1H), 2.43 – 2.32 (m, 1H), 1.74 – 1.62 (m, 1H), 1.55 – 1.42 (m, 1H), 1.31 – 1.20 (m, 1H), 0.86 (d, *J* = 6.5 Hz, 3H), 0.81 (d, *J* = 6.6 Hz, 3H). **^{13}C NMR (100 MHz, CDCl$_3$)** δ 194.91, 143.53, 138.64, 137.61, 131.73, 129.70, 129.03, 128.70, 128.54, 127.96, 127.27, 127.11, 126.19, 125.94, 44.79, 40.64, 36.77, 26.35, 23.24, 22.68. **ESI-MS:** calculated $[C_{24}H_{26}N_2O + H]^+$: 359.2118, found: 359.2124. $[\alpha]^{20}_D$= -95.5 (c = 0.85, CH_2Cl_2). The product was analyzed by HPLC to determine the enantiomeric excess: 96% e.e. (CHIRALPAK IG, hexane/*i*-PrOH = 90/10, detector: 300 nm, T = 25 °C, flow rate: 1 mL/min), t_1 (major) = 5.38 min, t_2 (minor) = 6.00 min.

(*R*,*E*)-2-(cyclopentylmethyl)-5-phenyl-1-(1-phenyl-1H-imidazol-2-yl)pent-4-en-1-one (3e)

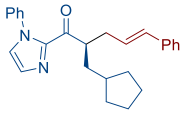

Reaction time: 2 d. Yield: 78%. **^{1}H NMR (400 MHz, CDCl$_3$)** δ 7.44 – 7.35 (m, 3H), 7.28 (d, *J* = 0.8 Hz, 1H), 7.27 – 7.23 (m, 4H), 7.21 – 7.12 (m, 4H), 6.34 (d, *J* = 15.8 Hz, 1H), 6.25 – 6.11 (m, 1H), 4.20 – 4.09 (m, 1H), 2.64 – 2.53 (m, 1H), 2.51 – 2.41 (m, 1H), 1.92 – 1.66 (m, 4H), 1.65 – 1.53 (m, 2H), 1.52 – 1.40 (m, 3H), 1.17 – 1.03 (m, 2H). **^{13}C NMR (100 MHz, CDCl$_3$)** δ 194.97, 143.60, 138.70, 137.68, 131.73, 129.71, 129.03, 128.71, 128.55, 128.07, 127.24, 127.10, 126.20, 125.99, 46.15, 38.48, 37.95, 36.61, 33.21, 33.02, 25.29, 25.26. **ESI-MS:** calculated $[C_{26}H_{28}N_2O + H]^+$: 385.2274, found: 385.2282. $[\alpha]^{20}_D$= -61.6 (c = 0.90, CH_2Cl_2). The product was analyzed by HPLC to determine the enantiomeric excess: 96% e.e. (CHIRALPAK IG, hexane/*i*-PrOH = 90/10, detector: 254 nm, T = 25 °C, flow rate: 1 mL/min), t_1 (major) = 6.35 min, t_2 (minor) = 8.36 min.

(*R*)-2-cinnamyl-1-(1-phenyl-1H-imidazol-2-yl)octan-1-one (3f)

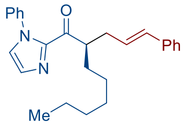

Reaction time: 1.5 d. Yield: 67%. **^{1}H NMR (400 MHz, CDCl$_3$)** δ 7.45 – 7.35 (m, 3H), 7.29 – 7.24 (m, 5H), 7.21 – 7.12 (m, 4H), 6.35 (d, *J* = 15.8 Hz, 1H), 6.17 (ddd, 1H), 4.13 – 4.02 (m, 1H), 2.63 – 2.53 (m, 1H), 2.50 – 2.39 (m, 1H), 1.83 – 1.71 (m, 1H), 1.61 – 1.48 (m, 1H), 1.34 – 1.19 (m, 8H), 0.84 (t, *J* = 6.8 Hz, 3H). **^{13}C NMR (100 MHz, CDCl$_3$)** δ 194.81, 143.61, 138.68, 137.69, 131.74, 129.68, 129.05, 128.73, 128.55, 128.09,

127.22, 127.10, 126.20, 125.98, 46.93, 35.85, 31.83, 31.78, 29.59, 27.48, 22.71, 14.18. **ESI-MS:** calculated $[C_{26}H_{30}N_2O + H]^+$: 387.2431, found: 387.2435. $[\alpha]^{20}_{D}$= -59.5 (c = 0.80, CH_2Cl_2). The product was analyzed by HPLC to determine the enantiomeric excess: 94% e.e. (CHIRALPAK IC, hexane/*i*-PrOH = 95/5, detector: 254 nm, T = 25 °C, flow rate: 1 mL/min), t_1 (minor) = 6.31 min, t_2 (major) = 6.87 min.

(*R*,*E*)-2-phenethyl-5-phenyl-1-(1-phenyl-1H-imidazol-2-yl)pent-4-en-1-one (3g)

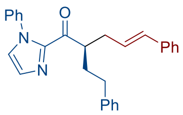

Reaction time: 4 d. Yield: 89%. **^{1}H NMR (400 MHz, $CDCl_3$)** δ 7.46 – 7.36 (m, 3H), 7.30 – 7.28 (m, 1H), 7.27 – 7.21 (m, 6H), 7.20 – 7.11 (m, 7H), 6.36 (d, *J* = 15.8 Hz, 1H), 6.24 – 6.12 (m, 1H), 4.23 – 4.11 (m, 1H), 2.70 – 2.57 (m, 3H), 2.55 – 2.45 (m, 1H), 2.20 – 2.08 (m, 1H), 1.93 – 1.79 (m, 1H). **^{13}C NMR (100 MHz, $CDCl_3$)** δ 194.22, 143.50, 142.20, 138.60, 137.57, 132.00, 129.76, 129.07, 128.79, 128.57, 128.53, 128.41, 127.68, 127.34, 127.18, 126.23, 125.98, 125.94, 46.85, 36.06, 33.93, 33.40. **ESI-MS:** calculated $[C_{28}H_{26}N_2O + H]^+$: 407.2118, found: 407.2124. $[\alpha]^{20}_{D}$= -39.4 (c = 1.19, CH_2Cl_2). The product was analyzed by HPLC to determine the enantiomeric excess: 95% e.e. (CHIRALPAK IG, hexane/*i*-PrOH = 90/10, detector: 254 nm, T = 25 °C, flow rate: 1 mL/min), t_1 (major) = 10.85 min, t_2 (minor) = 11.64 min.

(*S*,*E*)-2,5-diphenyl-1-(1-phenyl-1H-imidazol-2-yl)pent-4-en-1-one (3h)

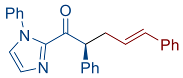

Reaction time: 1 d. T: 0 °C. Cs_2CO_3: 1.0 equiv. Yield: 90%. **^{1}H NMR (400 MHz, $CDCl_3$)** δ 7.47 – 7.36 (m, 5H), 7.30 (t, *J* = 7.5 Hz, 2H), 7.27 – 7.20 (m, 6H), 7.19 – 7.14 (m, 1H), 7.13 – 7.07 (m, 3H), 6.41 (d, *J* = 15.8 Hz, 1H), 6.20 – 6.07 (m, 1H), 5.33 (t, *J* = 7.6 Hz, 1H), 3.10 – 2.94 (m, 1H), 2.74 – 2.62 (m, 1H). **^{13}C NMR (100 MHz, $CDCl_3$)** δ 190.47, 143.02, 138.75, 138.38, 137.61, 132.09, 129.88, 129.07, 128.84, 128.78, 128.51, 127.65, 127.36, 127.22, 127.11, 126.19, 125.75, 53.19, 36.40. **ESI-MS:** calculated $[C_{26}H_{22}N_2O + H]^+$: 379.1805, found: 379.1819. $[\alpha]^{20}_{D}$= 81.8 (c = 1.25, CH_2Cl_2). The product was analyzed by HPLC to determine the enantiomeric excess: 90% e.e. (CHIRALPAK AD-H, hexane/*i*-PrOH = 90/10, detector: 254 nm, T = 25 °C, flow rate: 1 mL/min), t_1 (minor) = 9.08 min, t_2 (major) = 11.74 min.

(*S*,*E*)-5-phenyl-1-(1-phenyl-1H-imidazol-2-yl)-2-(p-tolyl)pent-4-en-1-one (3i)

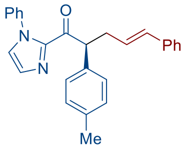

Reaction time: 1 d. T: 0 °C. Cs_2CO_3: 1.0 equiv. Yield: 96%. **^{1}H NMR (400 MHz, $CDCl_3$)** δ 7.45 – 7.37 (m, 3H), 7.34 – 7.29 (m, 2H), 7.26 – 7.22 (m, 5H), 7.19 – 7.07 (m, 6H), 6.40 (d, *J* = 15.8 Hz, 1H), 6.20 – 6.06 (m, 1H), 5.34 – 5.24 (m, 1H), 3.05 – 2.92 (m, 1H), 2.71 – 2.59 (m, 1H), 2.30 (s, 3H).. **^{13}C NMR (100 MHz, $CDCl_3$)** δ 190.62, 143.14, 138.49, 137.73, 136.81, 135.75, 132.02, 129.86, 129.52, 129.07, 128.76, 128.74, 128.51, 127.86, 127.25, 127.09, 126.22, 125.79, 52.86, 36.41, 21.20. **ESI-MS:** calculated $[C_{27}H_{24}N_2O + H]^+$: 393.1961, found: 393.1971. $[\alpha]^{20}_{D}$= 106.4 (c = 1.21, CH_2Cl_2). The product was analyzed by HPLC to determine the enantiomeric excess: 90% e.e. (CHIRALPAK IG, hexane/*i*-PrOH = 70/30, detector: 254 nm, T = 25 °C, flow rate: 1 mL/min), t_1 (major) = 6.94 min, t_2 (minor) = 8.04 min.

(*S*,*E*)-2-(4-fluorophenyl)-5-phenyl-1-(1-phenyl-1H-imidazol-2-yl)pent-4-en-1-one (3j)

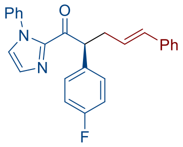

Reaction time: 1 d. T: 0 °C. Cs_2CO_3: 1.0 equiv. Yield: 98%. **^{1}H NMR (400 MHz, $CDCl_3$)** δ 7.47 – 7.36 (m, 5H), 7.26 – 7.22 (m, 5H), 7.19 – 7.14 (m, 1H), 7.13 – 7.08 (m, 3H), 7.03 – 6.95 (m, 2H), 6.39 (d, *J* = 15.8 Hz, 1H), 6.18 – 6.02 (m, 1H), 5.33 (t, *J* = 7.7 Hz, 1H), 3.05 – 2.92 (m, 1H), 2.73 – 2.60 (m, 1H). **^{13}C NMR (100 MHz,

CDCl3) δ 190.40, 162.13 (d, *J* = 245.5 Hz), 142.93, 138.39, 137.57, 134.49 (d, *J* = 3.0 Hz), 132.33, 130.42 (d, *J* = 7.9 Hz), 129.97, 129.12, 128.89, 128.56, 127.54, 127.35, 127.22, 126.22, 125.81, 115.61 (d, *J* = 21.3 Hz), 52.29, 36.47. **^{19}F NMR (565 MHz, CDCl3)** δ -115.51. **ESI-MS:** calculated $[C_{26}H_{21}FN_2O + H]^+$: 397.1711, found: 397.1712. $[\alpha]^{20}_D$= 36.4 (c = 1.49, CH_2Cl_2). The product was analyzed by HPLC to determine the enantiomeric excess: 90% e.e. (CHIRALPAK IA, hexane/*i*-PrOH = 90/10, detector: 254 nm, T = 25 °C, flow rate: 1 mL/min), t_1 (minor) = 6.94 min, t_2 (major) = 8.16 min.

(*R*,*E*)-1-(1-phenyl-1H-imidazol-2-yl)-2-propyl-5-(p-tolyl)pent-4-en-1-one (3k)

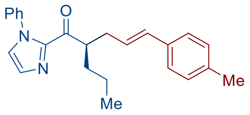

Reaction time: 3 d. Yield: 93%. **^{1}H NMR (400 MHz, CDCl3)** δ 7.46 – 7.35 (m, 3H), 7.30 – 7.26 (m, 1H), 7.21 – 7.12 (m, 5H), 7.10 – 7.04 (m, 2H), 6.32 (d, *J* = 15.8 Hz, 1H), 6.21 – 6.05 (m, 1H), 4.14 – 4.00 (m, 1H), 2.67 – 2.52 (m, 1H), 2.50 – 2.39 (m, 1H), 2.30 (s, 3H), 1.84 – 1.69 (m, 1H), 1.58 – 1.45 (m, 1H), 1.43 – 1.28 (m, 2H), 0.89 (t, *J* = 7.3 Hz, 3H). **^{13}C NMR (100 MHz, CDCl3)** δ 194.89, 143.62, 138.68, 136.82, 134.90, 131.58, 129.66, 129.25, 129.05, 128.72, 127.22, 126.99, 126.09, 125.98, 46.77, 35.96, 33.92, 21.26, 20.79, 14.40. **ESI-MS:** calculated $[C_{24}H_{26}N_2O + H]^+$: 359.2118, found: 359.2124. $[\alpha]^{20}_D$= -81.1 (c = 1.28, CH_2Cl_2). The product was analyzed by HPLC to determine the enantiomeric excess: 96% e.e. (CHIRALPAK IG, hexane/*i*-PrOH = 99/1, detector: 254 nm, T = 25 °C, flow rate: 1 mL/min), t_1 (major) = 19.97 min, t_2 (minor) = 21.44 min.

(*R*,*E*)-5-(4-(tert-butyl)phenyl)-1-(1-phenyl-1H-imidazol-2-yl)-2-propylpent-4-en-1-one (3l)

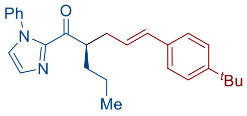

Reaction time: 4 d. Yield: 83%. **^{1}H NMR (400 MHz, CDCl3)** δ 7.46 – 7.37 (m, 3H), 7.32 – 7.27 (m, 3H), 7.24 – 7.18 (m, 4H), 7.15 (d, *J* = 1.0 Hz, 1H), 6.34 (d, *J* = 15.8 Hz, 1H), 6.20 – 6.08 (m, 1H), 4.13 – 4.00 (m, 1H), 2.67 – 2.52 (m, 1H), 2.48 – 2.36 (m, 1H), 1.81 – 1.66 (m, 1H), 1.59 – 1.45 (m, 1H), 1.35 – 1.27 (m, 11H). 0.88 (t, *J* = 7.3 Hz, 3H). **^{13}C NMR (100 MHz, CDCl3)** δ 194.86, 150.13, 143.53, 138.67, 134.91, 131.50, 129.68, 129.07, 128.74, 127.26, 127.25, 125.98, 125.90, 125.47, 46.77, 35.88, 34.63, 33.90, 31.44, 20.79, 14.43. **ESI-MS:** calculated $[C_{27}H_{32}N_2O + H]^+$: 401.2587, found: 401.2596. $[\alpha]^{20}_D$= -72.4 (c = 1.34, CH_2Cl_2). The product was analyzed by HPLC to determine the enantiomeric excess: 94% e.e. (CHIRALPAK IG, hexane/*i*-PrOH = 95/5, detector: 254 nm, T = 25 °C, flow rate: 1 mL/min), t_1 (major) = 6.46 min, t_2 (minor) = 7.94 min.

(*R*,*E*)-5-(4-fluorophenyl)-1-(1-phenyl-1H-imidazol-2-yl)-2-propylpent-4-en-1-one (3m)

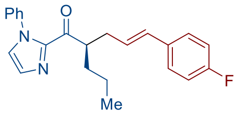

Reaction time: 4 d. Yield: 73%. **^{1}H NMR (400 MHz, CDCl3)** δ 7.49 – 7.39 (m, 3H), 7.31 – 7.27 (m, 1H), 7.25 – 7.14 (m, 5H), 6.99 – 6.90 (m, 2H), 6.31 (d, *J* = 15.8 Hz, 1H), 6.16 – 6.03 (m, 1H), 4.13 – 4.02 (m, 1H), 2.65 – 2.52 (m, 1H), 2.50 – 2.38 (m, 1H), 1.82 – 1.70 (m, 1H), 1.56 – 1.46 (m, 1H), 1.39 – 1.28 (m, 2H), 0.90 (t, *J* = 7.3 Hz, 3H). **^{13}C NMR (100 MHz, CDCl3)** δ 194.76, 162.10 (d, *J* = 245.8 Hz), 143.53, 138.63, 133.80 (d, *J* = 3.4 Hz), 130.52, 129.70, 129.08, 128.79, 127.81 (d, *J* = 2.1 Hz), 127.60 (d, *J* = 7.8 Hz), 127.31, 125.95, 115.42 (d, *J* = 21.4 Hz), 46.69, 35.80, 33.95, 20.78, 14.41. **^{19}F NMR (377 MHz, CDCl3)** δ -115.48. **ESI-MS:** calculated $[C_{23}H_{23}FN_2O + H]^+$**:** 363.1867, found: 363.1874. $[\alpha]^{20}_D$= -89.5 (c = 0.93, CH_2Cl_2). The product was analyzed by HPLC to determine the enantiomeric excess: 95% e.e. (CHIRALPAK IBN-5, hexane/*i*-PrOH = 98/2, detector: 244 nm, T = 25 °C, flow rate: 1 mL/min), t_1 (minor) = 7.66 min, t_2 (major) = 8.28 min.

(*R*,*E*)-5-(4-chlorophenyl)-1-(1-phenyl-1H-imidazol-2-yl)-2-propylpent-4-en-1-one (3n)

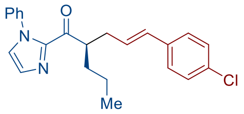

Reaction time: 4 d. Yield: 83%. **^{1}H NMR (400 MHz, CDCl$_3$)** δ 7.47 – 7.37 (m, 3H), 7.28 (d, *J* = 1.0 Hz, 1H), 7.25 – 7.14 (m, 7H), 6.30 (d, *J* = 15.8 Hz, 1H), 6.22 – 6.09 (m, 1H), 4.15 – 4.01 (m, 1H), 2.64 – 2.52 (m, 1H), 2.50 – 2.38 (m, 1H), 1.83 – 1.69 (m, 1H), 1.60 – 1.45 (m, 1H), 1.41 – 1.30 (m, 2H), 0.90 (t, *J* = 7.3 Hz, 3H). **^{13}C NMR (100 MHz, CDCl$_3$)** δ 194.66, 143.48, 138.59, 136.11, 132.65, 130.48, 129.71, 129.08, 128.85, 128.79, 128.68, 127.36, 127.34, 125.92, 46.59, 35.80, 33.98, 20.77, 14.40. **ESI-MS:** calculated [$C_{23}H_{23}ClN_2O$ + H]$^+$: 379.1572, found: 379.1582. $[\alpha]^{20}_{D}$= -98.5 (c = 1.07, CH_2Cl_2). The product was analyzed by HPLC to determine the enantiomeric excess: 96% e.e. (CHIRALPAK IBN-5, hexane/*i*-PrOH = 98/2, detector: 254 nm, T = 25 °C, flow rate: 1 mL/min), t_1 (minor) = 7.97 min, t_2 (major) = 8.57 min.

(*R*,*E*)-5-(4-bromophenyl)-1-(1-phenyl-1H-imidazol-2-yl)-2-propylpent-4-en-1-one (3o)

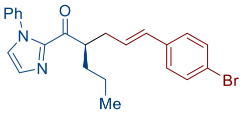

Reaction time: 4 d. Yield: 63%. **^{1}H NMR (400 MHz, CDCl$_3$)** δ 7.45 – 7.35 (m, 5H), 7.28 (d, *J* = 1.0 Hz, 1H), 7.20 – 7.15 (m, 3H), 7.14 – 7.12 (m, 1H), 7.12 – 7.10 (m, 1H), 6.29 (d, *J* = 15.9 Hz, 1H), 6.22 – 6.12 (m, 1H), 4.14 – 4.03 (m, 1H), 2.63 – 2.51 (m, 1H), 2.48 – 2.38 (m, 1H), 1.81 – 1.70 (m, 1H), 1.56 – 1.45 (m, 1H), 1.38 – 1.29 (m, 2H). 0.89 (t, *J* = 7.3 Hz, 3H). **^{13}C NMR (100 MHz, CDCl$_3$)** δ 194.66, 143.47, 138.59, 136.56, 131.63, 130.53, 129.72, 129.09, 129.02, 128.81, 127.71, 127.36, 125.93, 120.78, 46.57, 35.81, 33.99, 20.77, 14.41. **ESI-MS:** calculated [$C_{23}H_{23}BrN_2O$ + H]$^+$: 423.1067, found: 423.1074. $[\alpha]^{20}_{D}$= -94.4 (c = 0.89, CH_2Cl_2). The product was analyzed by HPLC to determine the enantiomeric excess: 92% e.e. (CHIRALPAK IBN-5, hexane/*i*-PrOH = 99/1, detector: 254 nm, T = 25 °C, flow rate: 1 mL/min), t_1 (minor) = 11.97 min, t_2 (major) = 13.28 min.

(*R*,*E*)-1-(1-phenyl-1H-imidazol-2-yl)-2-propyl-5-(m-tolyl)pent-4-en-1-one (3p)

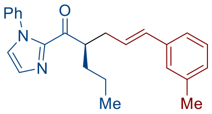

Reaction time: 3 d. Yield: 90%. **^{1}H NMR (400 MHz, CDCl$_3$)** δ 7.46 – 7.36 (m, 3H), 7.27 (d, *J* = 2.6 Hz, 1H), 7.22 – 7.13 (m, 4H), 7.10 – 7.04 (m, 2H), 6.99 (d, *J* = 7.4 Hz, 1H), 6.32 (d, *J* = 15.8 Hz, 1H), 6.22 – 6.10 (m, 1H), 4.13 – 4.01 (m, 1H), 2.66 – 2.52 (m, 1H), 2.50 – 2.38 (m, 1H), 2.31 (s, 3H), 1.83 – 1.69 (m, 1H), 1.60 – 1.46 (m, 1H), 1.42 – 1.29 (m, 2H), 0.89 (t, *J* = 7.3 Hz, 3H). **^{13}C NMR (100 MHz, CDCl$_3$)** δ 194.82, 143.60, 138.68, 138.05, 137.62, 131.82, 129.66, 129.05, 128.73, 128.45, 127.90, 127.85, 127.23, 126.91, 125.99, 123.38, 46.76, 35.91, 33.89, 21.51, 20.79, 14.40. **ESI-MS:** calculated [$C_{24}H_{26}N_2O$ + H]$^+$: 359.2118, found: 359.2126. $[\alpha]^{20}_{D}$= -76.9 (c = 1.09, CH_2Cl_2). The product was analyzed by HPLC to determine the enantiomeric excess: 95% e.e. (CHIRALPAK IBN-5, hexane/*i*-PrOH = 95/5, detector: 254 nm, T = 25 °C, flow rate: 1 mL/min), t_1 (minor) = 5.96 min, t_2 (major) = 6.30 min.

(*R*,*E*)-5-(3-fluorophenyl)-1-(1-phenyl-1H-imidazol-2-yl)-2-propylpent-4-en-1-one (3q)

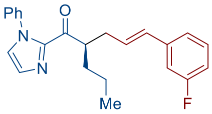

Reaction time: 1 d. Yield: 70%. **^{1}H NMR (400 MHz, CDCl$_3$)** δ 7.46 – 7.38 (m, 3H), 7.29 (d, *J* = 1.0 Hz, 1H), 7.25 – 7.12 (m, 4H), 7.04 – 7.00 (m, 1H), 6.98 – 6.93 (m, 1H), 6.90 – 6.84 (m, 1H), 6.32 (d, *J* = 15.8 Hz, 1H), 6.25 – 6.13 (m, 1H), 4.14 – 4.04 (m, 1H), 2.65 – 2.53 (m, 1H), 2.50 – 2.39 (m, 1H), 1.81 – 1.69 (m, 1H), 1.58 – 1.47 (m, 1H), 1.39 – 1.29 (m, 2H). **^{13}C NMR (100 MHz, CDCl$_3$)** δ 194.61, 163.21 (d, *J* = 244.9 Hz), 143.53, 140.06 (d, *J* = 7.6 Hz), 138.64, 130.68 (d, *J* = 2.3 Hz), 129.95 (d, *J* = 8.3 Hz), 129.73, 129.64, 129.08, 128.80, 127.33, 125.96, 122.05 (d, *J* = 2.5 Hz), 113.88 (d, *J* = 21.4 Hz), 112.60 (d, *J* = 21.5 Hz), 46.60, 35.72, 34.02, 20.76, 14.39. **^{19}F NMR (565 MHz, CDCl$_3$)** δ -113.73. **ESI-MS:** calculated

$[C_{23}H_{23}FN_2O + H]^+$: 363.1867, found: 363.1875. $[\alpha]^{20}_D$= -84.9 (c = 0.95, CH_2Cl_2). The product was analyzed by HPLC to determine the enantiomeric excess: 95% e.e. (CHIRALPAK IBN-5, hexane/*i*-PrOH = 95/5, detector: 260 nm, T = 25 °C, flow rate: 1 mL/min), t_1 (minor) = 5.99 min, t_2 (major) = 6.29 min.

(*R*,*E*)-1-(1-phenyl-1H-imidazol-2-yl)-2-propyl-5-(o-tolyl)pent-4-en-1-one (3r)

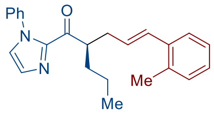

Reaction time: 3 d. Yield: 80%. **^{1}H NMR (400 MHz, $CDCl_3$)** δ 7.45 – 7.37 (m, 3H), 7.31 – 7.25 (m, 2H), 7.21 – 7.14 (m, 3H), 7.12 – 7.07 (m, 3H), 6.54 (d, *J* = 15.7 Hz, 1H), 6.11 – 5.98 (m, 1H), 4.17 – 4.05 (m, 1H), 2.69 – 2.54 (m, 1H), 2.53 – 2.43 (m, 1H), 2.25 (s, 3H), 1.83 – 1.72 (m, 1H), 1.61 – 1.48 (m, 1H), 1.42 – 1.30 (m, 2H), 0.90 (t, *J* = 7.3 Hz, 3H). **^{13}C NMR (100 MHz, $CDCl_3$)** δ 194.86, 143.60, 138.69, 136.84, 135.10, 130.20, 129.70, 129.60, 129.42, 129.05, 128.75, 127.27, 127.05, 126.08, 125.99, 125.78, 46.65, 36.18, 34.02, 20.78, 19.91, 14.42. **ESI-MS:** calculated $[C_{24}H_{26}N_2O + H]^+$: 359.2118, found: 359.2126. $[\alpha]^{20}_D$= -76.8 (c = 0.95, CH_2Cl_2). The product was analyzed by HPLC to determine the enantiomeric excess: 95% e.e. (CHIRALPAK IG, hexane/*i*-PrOH = 90/10, detector: 254 nm, T = 25 °C, flow rate: 1 mL/min), t_1 (minor) = 6.12 min, t_2 (major) = 6.57 min.

(*E*)-5-(naphthalen-2-yl)-1-(1-phenyl-1H-imidazol-2-yl)-2-propylpent-4-en-1-one (3s)

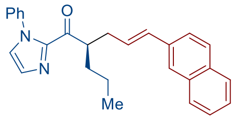

Reaction time: 2.5 d. Yield: 95%. **^{1}H NMR (400 MHz, $CDCl_3$)** δ 7.80 – 7.69 (m, 3H), 7.60 (s, 1H), 7.52 – 7.46 (m, 1H), 7.45 – 7.34 (m, 5H), 7.29 (d, *J* = 0.5 Hz, 1H), 7.20 – 7.15 (m, 2H), 7.12 (d, *J* = 0.8 Hz, 1H), 6.51 (d, *J* = 15.8 Hz, 1H), 6.39 – 6.25 (m, 1H), 4.20 – 4.08 (m, 1H), 2.71 – 2.59 (m, 1H), 2.57 – 2.46 (m, 1H), 1.86 – 1.73 (m, 1H), 1.61 – 1.50 (m, 1H), 1.44 – 1.29 (m, 2H), 0.91 (t, *J* = 7.3 Hz, 3H). **^{13}C NMR (100 MHz, $CDCl_3$)** δ 194.82, 143.54, 138.59, 135.08, 133.72, 132.81, 131.80, 129.67, 129.04, 128.73, 128.55, 128.13, 127.95, 127.72, 127.29, 126.24, 125.94, 125.66, 123.73, 46.72, 36.08, 33.96, 20.80, 14.42. **ESI-MS:** calculated $[C_{27}H_{26}N_2O + H]^+$: 395.2118, found: 395.2127. $[\alpha]^{20}_D$= -105.6 (c = 0.58, CH_2Cl_2). The product was analyzed by HPLC to determine the enantiomeric excess: 96% e.e. (CHIRALPAK IBN-5, hexane/*i*-PrOH = 90/10, detector: 254 nm, T = 25 °C, flow rate: 1 mL/min), t_1 (major) = 7.39 min, t_2 (minor) = 8.17 min.

(*R*)-1-(1-phenyl-1H-imidazol-2-yl)-2-propylpent-4-en-1-one (3t)

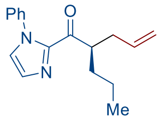

With **2aj** (0.3 mmol). Reaction time: 5 d. Yield: 60%. **^{1}H NMR (400 MHz, $CDCl_3$)** δ 7.51 – 7.41 (m, 3H), 7.30 – 7.27 (m, 1H), 7.26 – 7.21 (m, 2H), 7.20 – 7.15 (m, 1H), 5.84 – 5.70 (m, 1H), 5.07 – 4.92 (m, 2H), 4.06 – 3.94 (m, 1H), 2.51 – 2.40 (m, 1H), 2.32 – 2.23 (m, 1H), 1.75 – 1.67 (m, 1H), 1.53 – 1.43 (m, 1H), 1.33 – 1.26 (m, 2H), 0.88 (t, *J* = 7.3 Hz, 3H). **^{13}C NMR (100 MHz, $CDCl_3$)** δ 194.87, 143.45, 138.68, 136.15, 129.67, 129.10, 128.77, 127.28, 125.94, 116.52, 46.23, 36.50, 33.83, 20.70, 14.40. **ESI-MS:** calculated $[C_{17}H_{20}N_2O + H]^+$: 269.1648, found: 269.1649. $[\alpha]^{20}_D$= 5.0 (c = 0.4, CH_2Cl_2). The product was analyzed by HPLC to determine the enantiomeric excess: 82% e.e. (CHIRALPAK IF, hexane/*i*-PrOH = 98/2, detector: 280 nm, T = 25 °C, flow rate: 1 mL/min), t_1 (minor) = 7.58 min, t_2 (major) = 7.99 min.

(*R*)-5-methyl-1-(1-phenyl-1H-imidazol-2-yl)-2-propylhex-4-en-1-one (3u)

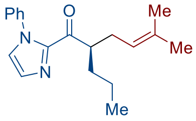

With **2ak** (0.3 mmol). Reaction time: 3 d. Yield: 78%. **^{1}H NMR (400 MHz, CDCl$_3$)** δ 7.49 – 7.42 (m, 3H), 7.27 (d, *J* = 0.9 Hz, 1H), 7.26 – 7.21 (m, 2H), 7.17 (d, *J* = 0.9 Hz, 1H), 5.14 – 5.07 (m, 1H), 3.94 – 3.85 (m, 1H), 2.41 – 2.32 (m, 1H), 2.28 – 2.20 (m, 1H), 1.71 – 1.63 (m, 4H), 1.55 (s, 3H), 1.51 – 1.42 (m, 1H), 1.33 – 1.25 (m, 2H), 0.88 (t, *J* = 7.3 Hz, 3H). **^{13}C NMR (100 MHz, CDCl$_3$)** δ 195.33, 143.52, 138.73, 133.22, 129.58, 129.07, 128.69, 127.06, 125.87, 121.83, 47.23, 33.59, 30.90, 25.93, 20.85, 17.92, 14.46. **ESI-MS:** calculated [$C_{19}H_{24}N_2O$ + H]$^+$: 297.1961, found: 297.1962. $[\alpha]^{20}_D$= -20.9 (c = 0.70, CH_2Cl_2). The product was analyzed by HPLC to determine the enantiomeric excess: 85% e.e. (CHIRALPAK IG, hexane/*i*-PrOH = 90/10, detector: 254 nm, T = 25 °C, flow rate: 1 mL/min), t_1 (major) = 5.70 min, t_2 (minor) = 6.09 min.

3.2.2 General procedure for the synthesis of branched products

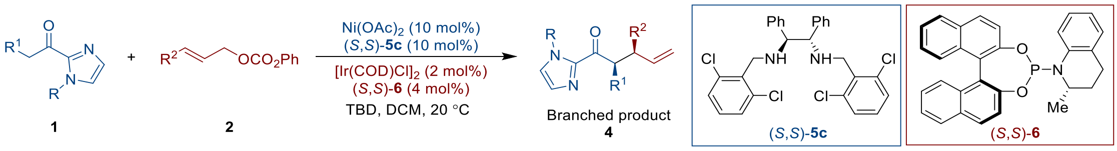

To a flame-dried and argon-purged Schlenk tube were added [Ir(COD)Cl]2 (0.002 mmol, 0.02 equiv.), 6 (0.004 mmol, 0.04 equiv.), TBD (0.01mmol, 0.1 equiv.), and DCM (1 mL), and the resulting solution was stirred at 25 °C for 10 min. Then, 2-acyl imidazoles **1** (0.1 mmol, 1.0 equiv.), allylic carbonates **2** (0.15 mmol, 1.5 equiv.), Ni catalyst (0.01 mmol, 0.1 equiv.), and DCM (1 mL) were added successively. The resulting solution was stirred at 20 °C. After the reaction was complete (monitored by TLC), the residue was purified by flash column chromatography on silica gel to afford the desired product **4**.

(2*R*,3*S*)-3-phenyl-1-(1-phenyl-1H-imidazol-2-yl)-2-propylpent-4-en-1-one (4a)

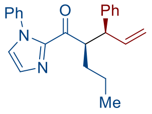

In a 10 mL Schlenk tube, [Ir(COD)Cl]$_2$ (0.002 mmol, 0.02 equiv.), (*S,S*)-**6** (0.004 mmol, 0.04 equiv.), TBD (0.01mmol, 0.1 equiv.) were stirred in 1 mL DCM under argon at 25 °C for 10 min. **1a** (0.1 mmol, 1.0 equiv.), allylic carbonate **2c** (0.15 mmol, 1.5equiv.), and (*S,S*)-Ni catalyst (0.01 mmol, 0.1equiv.) were then added successively and additional DCM (1 mL) was added. The mixture was stirred at 20 °C for 2 d. After the reaction was complete (monitored by TLC), the residue was purified by flash column chromatography on silica gel to afford the desired product **4a** (85% yield, >20:1 d.r., >99% e.e.). **^{1}H NMR (400 MHz, CDCl$_3$)** δ 7.53 – 7.42 (m, 3H), 7.35 – 7.21 (m, 8H), 7.19 – 7.16 (m, 1H), 5.97 (m, *J* = 17.1, 9.5 Hz, 1H), 4.97 (d, *J* = 17.0 Hz, 1H), 4.90 – 4.82 (m, 1H), 4.53 – 4.41 (m, 1H), 3.60 – 3.48 (m, 1H), 1.63 – 1.49 (m, 1H), 1.30 – 1.10 (m, 3H), 0.72 (t, *J* = 7.1 Hz, 3H). **^{13}C NMR (125 MHz, CDCl$_3$)** δ 195.05, 144.49, 142.41, 140.54, 138.75, 129.79, 129.03, 128.74, 128.24, 127.37, 126.65, 125.99, 115.08, 54.05, 50.67, 33.41, 20.68, 14.30. **ESI-MS:** calculated [$C_{23}H_{24}N_2O$ + H]$^+$: 345.1961, found: 345.1968. $[\alpha]^{20}_D$= -34.7 (c = 1.07, CH_2Cl_2). ^{1}H NMR spectroscopic analysis of the mixture indicated >20:1 d.r. The product was analyzed by HPLC to determine the enantiomeric excess: >99% e.e. (CHIRALPAK IG, hexane/*i*-PrOH = 96/4, detector: 300 nm, T = 40 °C, flow rate: 1 mL/min), t_1 (major) = 5.62 min, t_2 (minor) = 6.97 min.

(2*S*,3*R*)-3-phenyl-1-(1-phenyl-1H-imidazol-2-yl)-2-propylpent-4-en-1-one (4a)

In a 10 mL Schlenk tube, [Ir(COD)Cl]$_2$ (0.002 mmol, 0.02 equiv.), (*R,R*)-**6** (0.004 mmol, 0.04 equiv.), TBD (0.01mmol, 0.1 equiv.) were stirred in 1 mL DCM under argon at 25 °C for 10 min. **1a** (0.1 mmol,

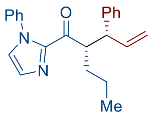

1.0 equiv.), allylic carbonate **2c** (0.15 mmol, 1.5equiv.), and (*R*,*R*)-Ni catalyst (0.01 mmol, 0.1 equiv.) were then added successively and additional DCM (1 mL) was added. The mixture was stirred at 20 °C for 3 d. After the reaction was complete (monitored by TLC), the residue was purified by flash column chromatography on silica gel to afford the desired product **4a** (72% yield, >20:1 d.r., >99% e.e.). **^{1}H NMR (400 MHz, $CDCl_3$)** δ 7.51 – 7.42 (m, 3H), 7.34 – 7.21 (m, 8H), 7.19 – 7.17 (m, 1H), 6.03 – 5.90 (m, 1H), 4.97 (d, *J* = 16.9 Hz, 1H), 4.91 – 4.82 (m, 1H), 4.54 – 4.40 (m, 1H), 3.60 – 3.48 (m, 1H), 1.62 – 1.48 (m, 1H), 1.28 – 1.10 (m, 3H), 0.72 (t, *J* = 7.1 Hz, 3H). **^{13}C NMR (100 MHz, $CDCl_3$)** δ 195.05, 144.48, 142.41, 140.54, 138.75, 129.79, 129.03, 128.74, 128.24, 127.37, 126.65, 125.98, 115.08, 54.05, 50.66, 33.41, 20.68, 14.30. **ESI-MS:** calculated $[C_{23}H_{24}N_2O + H]^+$: 345.1961, found: 345.1968. $[\alpha]^{20}_D$= 20.0 (c = 0.23, CH_2Cl_2). ^{1}H NMR spectroscopic analysis of the mixture indicated >20:1 d.r. The product was analyzed by HPLC to determine the enantiomeric excess: >99% e.e. (CHIRALPAK IG, hexane/*i*-PrOH = 96/4, detector: 300 nm, T = 40 °C, flow rate: 1 mL/min), t_1 (minor) = 5.44 min, t_2 (major) = 5.79 min.

Supplementary Table 1. Optimization studies of (*R*,*R*)-4a

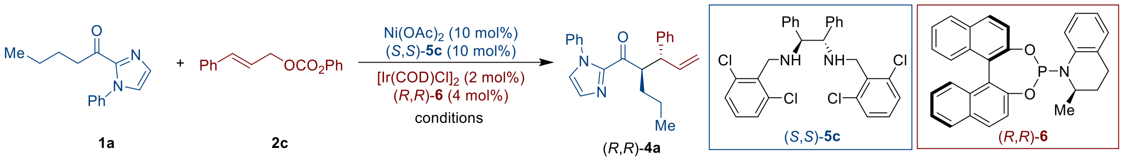

Entry	Conditions	Yield (%)	**3a**/**4a**	d.r. of **4a**	e.e. of **4a** (%)
1	TBD (0.1 equiv.), DCM, 20 °C	24	<1:20	6:1	99
2	NEt_3 (1.0 equiv.), DCM, 20 °C	6	<1:20	1:1	92
3	KO^{t}Bu (1.0 equiv.), DCM, 20 °C	20	<1:20	7:1	99
4	Cs_2CO_3 (1.0 equiv.), DCM, 0 °C	22	<1:20	20:1	>99
5	Cs_2CO_3 (1.0 equiv.), THF, 0 °C	<5			
6	Cs_2CO_3 (1.0 equiv.), toluene, 0 °C	<5			
7	Cs_2CO_3 (1.0 equiv.), 1,2-dichloropropane, 0 °C	20	<1:20	15:1	>99
8	Cs_2CO_3 (1.0 equiv.), DCE, 0 °C	45	<1:20	17:1	>99
9[a]	Cs_2CO_3 (1.0 equiv.), DCE, 0 °C	90	<1:20	14:1	>99

All the reactions were carried out at specified conditions by using **1a** (0.1 mmol), **2c** (0.15 mmol), $Ni(OAc)_2$ (10 mol%), (*S*,*S*)-**5c** (10 mol%), $[Ir(COD)Cl]_2$ (2 mol%), (*R*,*R*)-**6** (4 mol%) in solvent (2.0 mL). [a]**1a** (0.3 mmol), **2c** (0.9 mmol), in DCE (1.0 mL).

The general synthetic procedure of (*R*,*R*)-**4** were carried out as below: In a 10 mL Schlenk tube, $[Ir(COD)Cl]_2$ (0.006 mmol, 0.02 equiv.), (*R*,*R*)-**6** (0.012 mmol, 0.04 equiv.), and Cs_2CO_3 (0.3 mmol, 1.0 equiv.) were stirred in 0.5 mL DCE under argon at 25 °C for 1 hour. **1a** (0.3 mmol, 1.0 equiv.), allylic carbonate **2c** (0.9 mmol, 3.0 equiv.), and (*S*,*S*)-Ni catalyst (0.03 mmol, 0.1equiv.) were then added successively and additional DCE (0.5 mL) was added at 0 °C. After the reaction was complete (monitored by TLC), the residue was purified by flash column chromatography on silica gel to afford the desired product (*R*,*R*)-**4**.

Different enantiomers of the nickel and iridium catalysts were tested, and the combination of Ni/(*R*,*R*)-**5c** and Ir/(*S*,*S*)-**6** with Cs_2CO_3 as the base in DCE leads to (*S*,*S*)-**4a** in 94% yield, 12:1 d.r., and >99% e.e. The reaction was carried out by using **1a** (0.3 mmol), **2c** (0.9 mmol), $Ni(OAc)_2$ (10 mol%), (*R*,*R*)-**5c** (10

mol%), [Ir(COD)Cl]$_2$ (2 mol%), (*S*,*S*)-**6** (4 mol%), and Cs_2CO_3 (0.3 mmol) in dichloroethane (DCE) (1.0 mL) at 0 °C.

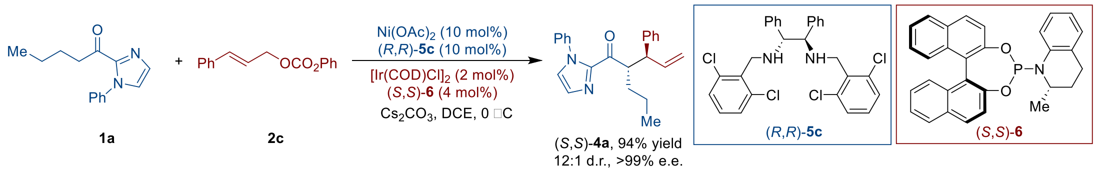

(2*R*,3*R*)-3-phenyl-1-(1-phenyl-1H-imidazol-2-yl)-2-propylpent-4-en-1-one (4a)

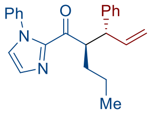

In a 10 mL Schlenk tube, [Ir(COD)Cl]$_2$ (0.006 mmol, 0.02 equiv.), (*R*,*R*)-**6** (0.012 mmol, 0.04 equiv.), Cs_2CO_3 (0.3 mmol, 1.0 equiv.) were stirred in 0.5 mL DCE under argon at 25 °C for 1 hour. **1a** (0.3 mmol, 1.0 equiv.), allylic carbonate **2c** (0.9 mmol, 3.0 equiv.), and (*S*,*S*)-Ni catalyst (0.03 mmol, 0.1 equiv.) were then added successively and additional DCE (0.5 mL) was added. The mixture was stirred at 0 °C for 2d. After the reaction was complete (monitored by TLC), the residue was purified by flash column chromatography on silica gel to afford the desired product **4a** (90% yield, 14:1 d.r., >99% e.e.). **^{1}H NMR (400 MHz, $CDCl_3$)** δ 7.34 – 7.26 (m, 3H), 7.24 – 7.15 (m, 5H), 7.14 – 7.07 (m, 1H), 6.99 (d, *J* = 0.5 Hz, 1H), 6.62 (d, *J* = 7.5 Hz, 2H), 6.05 – 5.91 (m, 1H), 5.15 – 5.03 (m, 2H), 4.43 – 4.32 (m, 1H), 3.58 (t, *J* = 9.9 Hz, 1H), 1.81 – 1.67 (m, 2H), 1.41 – 1.23 (m, 2H), 0.87 (t, *J* = 7.3 Hz, 3H). **^{13}C NMR (100 MHz, $CDCl_3$)** δ 194.72, 144.38, 142.45, 140.26, 138.30, 129.47, 128.79, 128.48, 128.42, 128.31, 126.69, 126.39, 125.54, 116.07, 54.01, 50.68, 33.72, 20.72, 14.46. **ESI-MS:** calculated $[C_{23}H_{24}N_2O + H]^+$: 345.1961, found: 345.1968. $[\alpha]^{20}_D$= 59.9 (c = 1.60, CH_2Cl_2). ^{1}H NMR spectroscopic analysis of the mixture indicated 14:1 d.r. The product was analyzed by HPLC to determine the enantiomeric excess: >99% e.e. (CHIRALPAK IG, hexane/*i*-PrOH = 96/4, detector: 300 nm, T = 40 °C, flow rate: 1 mL/min), t_1 (major) = 5.09 min, t_2 (minor) = 6.11 min.

(2*S*,3*S*)-3-phenyl-1-(1-phenyl-1H-imidazol-2-yl)-2-propylpent-4-en-1-one (4a)

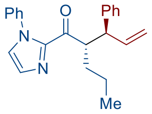

In a 10 mL Schlenk tube, [Ir(COD)Cl]$_2$ (0.006 mmol, 0.02 equiv.), (*S*,*S*)-**6** (0.012 mmol, 0.04 equiv.), Cs_2CO_3 (0.3 mmol, 1.0 equiv.) were stirred in 0.5 mL DCE under argon at 25 °C for 1 hour. **1a** (0.3 mmol, 1.0 equiv.), allylic carbonate **2c** (0.9 mmol, 3.0 equiv.), and (*R*,*R*)-Ni catalyst (0.03 mmol, 0.1 equiv.) were then added successively and additional DCE (0.5 mL) was added. The mixture was stirred at 0 °C for 2 d. After the reaction was complete (monitored by TLC), the residue was purified by flash column chromatography on silica gel to afford the desired product **4a** (94% yield, 12:1 d.r., >99% e.e.). **^{1}H NMR (400 MHz, $CDCl_3$)** δ 7.35 – 7.26 (m, 3H), 7.23 – 7.15 (m, 5H), 7.14 – 7.08 (m, 1H), 6.99 (d, *J* = 1.0 Hz, 1H), 6.70 – 6.55 (m, 2H), 6.07 – 5.90 (m, 1H), 5.16 – 5.01 (m, 2H), 4.45 – 4.32 (m, 1H), 3.58 (t, *J* = 9.9 Hz, 1H), 1.81 – 1.68 (m, 2H), 1.40 – 1.23 (m, 2H), 0.87 (t, *J* = 7.3 Hz, 3H). **^{13}C NMR (100 MHz, $CDCl_3$)** δ 194.77, 144.42, 142.49, 140.29, 138.34, 129.48, 128.83, 128.53, 128.47, 128.35, 126.73, 126.42, 125.59, 116.11, 54.04, 50.74, 33.75, 20.75, 14.49. **ESI-MS:** calculated $[C_{23}H_{24}N_2O + H]^+$: 345.1961, found: 345.1968. $[\alpha]^{20}_D$= -59.1 (c = 0.77, CH_2Cl_2). ^{1}H NMR spectroscopic analysis of the mixture indicated 12:1 d.r. The product was analyzed by HPLC to determine the enantiomeric excess: >99% e.e. (CHIRALPAK IG, hexane/*i*-PrOH = 96/4, detector: 300 nm, T = 40 °C, flow rate: 1 mL/min), t_1 (minor) = 4.78 min, t_2 (major) = 5.82 min.

(2*R*,3*S*)-2-methyl-3-phenyl-1-(1-phenyl-1H-imidazol-2-yl)pent-4-en-1-one (4b)

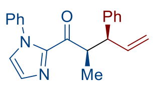

Reaction time: 1 d. Yield: 78%. **^{1}H NMR (400 MHz, CDCl$_3$)** δ 7.50 – 7.42 (m, 3H), 7.34 – 7.29 (m, 3H), 7.27 – 7.14 (m, 6H), 6.08 – 5.93 (m, 1H), 5.01 (d, *J* = 17.1 Hz, 1H), 4.91 (d, *J* = 10.3 Hz, 1H), 4.50 – 4.37 (m, 1H), 3.64 – 3.53 (m, 1H), 0.96 (d, *J* = 6.9 Hz, 3H). **^{13}C NMR (100 MHz, CDCl$_3$)** δ 194.76, 143.37, 142.04, 140.62, 138.64, 129.77, 129.10, 128.82, 128.73, 128.47, 127.47, 126.73, 125.91, 115.09, 53.58, 45.39, 16.46. **ESI-MS:** calculated [$C_{21}H_{20}N_2O$ + H]$^+$: 317.1648, found: 317.1658. $[\alpha]^{20}_{D}$= -52.3 (c = 0.33, CH_2Cl_2). ^{1}H NMR spectroscopic analysis of the mixture indicated >20:1 d.r. The product was analyzed by HPLC to determine the enantiomeric excess: 99% e.e. (CHIRALPAK IC, hexane/*i*-PrOH = 98/2, detector: 290 nm, T = 25 °C, flow rate: 1 mL/min), t_1 (major) = 11.18 min, t_2 (minor) = 12.71 min.

(2*R*,3*S*)-2-ethyl-3-phenyl-1-(1-phenyl-1H-imidazol-2-yl)pent-4-en-1-one (4c)

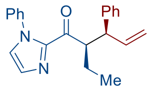

Reaction time: 5 d. Yield: 58%. **^{1}H NMR (500 MHz, CDCl$_3$)** δ 7.49 – 7.43 (m, 3H), 7.34 – 7.29 (m, 3H), 7.29 – 7.26 (m, 3H), 7.26 – 7.24 (m, 1H), 7.23 – 7.19 (m, 2H), 6.04 – 5.92 (m, 1H), 5.03 – 4.94 (m, 1H), 4.91 – 4.84 (m, 1H), 4.43 – 4.36 (m, 1H), 3.62 – 3.53 (m, 1H), 1.63 – 1.52 (m, 1H), 1.40 – 1.31 (m, 1H), 0.73 (t, *J* = 7.5 Hz, 3H). **^{13}C NMR (100 MHz, CDCl$_3$)** δ 194.82, 144.55, 142.37, 140.56, 138.76, 129.79, 129.06, 128.78, 128.74, 128.31, 127.35, 126.67, 126.01, 115.11, 53.37, 52.12, 24.26, 11.68. **ESI-MS:** calculated [$C_{22}H_{22}N_2O$ + H]$^+$: 331.1805, found: 331.1810. $[\alpha]^{20}_{D}$= -39.3 (c = 0.23, CH_2Cl_2). ^{1}H NMR spectroscopic analysis of the mixture indicated >20:1 d.r. The product was analyzed by HPLC to determine the enantiomeric excess: 98% e.e. (CHIRALPAK IG, hexane/*i*-PrOH = 97/3, detector: 300 nm, T = 25 °C, flow rate: 1 mL/min), t_1 (major) = 7.16 min, t_2 (minor) = 7.69 min.

(2*R*,3*S*)-2-isobutyl-3-phenyl-1-(1-phenyl-1H-imidazol-2-yl)pent-4-en-1-one (4d)

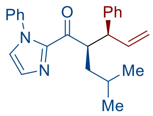

Reaction time: 2.5 d. Yield: 86%. **^{1}H NMR (400 MHz, CDCl$_3$)** δ 7.48 – 7.43 (m, 3H), 7.34 – 7.26 (m, 5H), 7.26 – 7.19 (m, 3H), 7.17 (d, *J* = 1.0 Hz, 1H), 6.05 – 5.91 (m, 1H), 5.00 – 4.90 (m, 1H), 4.89 – 4.79 (m, 1H), 4.61 – 4.51 (m, 1H), 3.45 (t, *J* = 10.0 Hz, 1H), 1.72 – 1.61 (m, 1H), 1.36 – 1.28 (m, 1H), 1.00 – 0.91 (m, 1H), 0.83 (d, *J* = 6.6 Hz, 3H), 0.70 (d, *J* = 6.6 Hz, 3H). **^{13}C NMR (100 MHz, CDCl$_3$)** δ 195.24, 144.58, 142.62, 140.60, 138.84, 129.84, 129.02, 128.77, 128.73, 128.15, 127.36, 126.65, 126.03, 114.98, 55.30, 48.95, 40.32, 26.38, 23.97, 21.74. **ESI-MS:** calculated [$C_{24}H_{26}N_2O$ + H]$^+$: 359.2118, found: 359.2125. $[\alpha]^{20}_{D}$= -45.5 (c = 0.51, CH_2Cl_2). ^{1}H NMR spectroscopic analysis of the mixture indicated >20:1 d.r. The product was analyzed by HPLC to determine the enantiomeric excess: 99% e.e. (CHIRALPAK IG, hexane/*i*-PrOH = 90/10, detector: 254 nm, T = 25 °C, flow rate: 1 mL/min), t_1 (major) = 4.20 min, t_2 (minor) = 4.76 min.

(2*R*,3*S*)-2-(cyclopentylmethyl)-3-phenyl-1-(1-phenyl-1H-imidazol-2-yl)pent-4-en-1-one (4e)

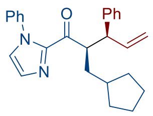

Reaction time: 5 d. Yield: 79%. **^{1}H NMR (400 MHz, CDCl$_3$)** δ 7.48 – 7.42 (m, 3H), 7.34 – 7.30 (m, 2H), 7.30 – 7.27 (m, 2H), 7.26 – 7.23 (m, 3H), 7.22 – 7.18 (m, 1H), 7.17 (d, *J* = 0.8 Hz, 1H), 6.04 – 5.89 (m, 1H), 4.99 – 4.90 (m, 1H), 4.88 – 4.78 (m, 1H), 4.57 – 4.44 (m, 1H), 3.52 – 3.42 (m, 1H), 1.87 – 1.74 (m, 2H), 1.55 – 1.47 (m, 2H), 1.46 – 1.31 (m, 4H), 1.13 – 1.05 (m, 1H), 0.96 – 0.82 (m, 2H). **^{13}C NMR (100 MHz, CDCl$_3$)** δ 195.32, 144.63, 142.68, 140.63, 138.90, 129.84, 129.02, 128.78, 128.73, 128.21, 127.33, 126.65, 126.09, 114.99, 55.13, 50.37, 38.55, 37.49, 33.63, 32.41, 25.21, 25.19. **ESI-MS:** calculated [$C_{26}H_{28}N_2O$ + H]$^+$: 385.2274, found: 385.2280. $[\alpha]^{20}_{D}$= -19.1 (c = 0.23, CH_2Cl_2). ^{1}H NMR spectroscopic analysis of the mixture indicated >20:1 d.r. The product was analyzed by HPLC to determine the enantiomeric excess: 99% e.e. (CHIRALPAK IG,

hexane/*i*-PrOH = 95/5, detector: 279 nm, T = 25 °C, flow rate: 1 mL/min), t_1 (major) = 5.36 min, t_2 (minor) = 6.74 min.

(*R*)-1-(1-phenyl-1H-imidazol-2-yl)-2-((*S*)-1-phenylallyl)octan-1-one (4f)

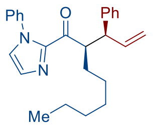

Reaction time: 5 d. Yield: 68%. **^{1}H NMR (400 MHz, CDCl$_3$)** δ 7.49 – 7.42 (m, 3H), 7.34 – 7.26 (m, 5H), 7.26 – 7.17 (m, 4H), 6.06 – 5.90 (m, 1H), 4.96 (d, *J* = 17.0 Hz, 1H), 4.91 – 4.81 (m, 1H), 4.50 – 4.37 (m, 1H), 3.60 – 3.48 (m, 1H), 1.64 – 1.50 (m, 1H), 1.27 – 1.22 (m, 1H), 1.19 – 0.98 (m, 8H), 0.77 (t, *J* = 7.1 Hz, 3H). **^{13}C NMR (100 MHz, CDCl$_3$)** δ 195.01, 144.57, 142.47, 140.61, 138.83, 129.80, 129.04, 128.74, 128.30, 127.31, 126.66, 126.04, 115.07, 53.96, 50.88, 31.67, 31.22, 29.44, 27.30, 22.60, 14.12. **ESI-MS:** calculated [$C_{26}H_{30}N_2O$ + H]$^+$: 387.2431, found: 387.2436. $[\alpha]^{20}_D$= -16.3 (c = 0.49, CH_2Cl_2). ^{1}H NMR spectroscopic analysis of the mixture indicated >20:1 d.r. The product was analyzed by HPLC to determine the enantiomeric excess: 97% e.e. (CHIRALPAK IG, hexane/*i*-PrOH = 99/1, detector: 270 nm, T = 25 °C, flow rate: 1 mL/min), t_1 (major) = 10.52 min, t_2 (minor) = 12.76 min.

(2*R*,3*S*)-2-benzyl-3-phenyl-1-(1-phenyl-1H-imidazol-2-yl)pent-4-en-1-one (4g)

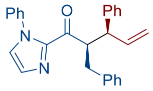

Reaction time: 2 d. T: 0 °C. Yield: 84%. **^{1}H NMR (400 MHz, CDCl$_3$)** δ 7.45 – 7.32 (m, 7H), 7.25 – 7.18 (m, 2H), 7.11 (t, *J* = 7.2 Hz, 2H), 7.08 – 6.95 (m, 6H), 6.13 – 5.98 (m, 1H), 5.00 (d, *J* = 17.0 Hz, 1H), 4.90 – 4.85 (m, 1H), 4.85 – 4.77 (m, 1H), 3.63 (t, *J* = 9.8 Hz, 1H), 2.89 – 2.74 (m, 1H), 2.69 – 2.55 (m, 1H). **^{13}C NMR (100 MHz, CDCl$_3$)** δ 193.79, 144.37, 142.23, 140.00, 139.37, 138.56, 129.67, 129.17, 128.93, 128.89, 128.67, 128.27, 128.09, 127.03, 126.88, 125.99, 125.96, 115.51, 54.49, 52.40, 37.36. **ESI-MS:** calculated [$C_{27}H_{24}N_2O$ + H]$^+$: 393.1961, found: 393.1969**.** $[\alpha]^{20}_D$= 7.5 (c = 0.59, CH_2Cl_2). ^{1}H NMR spectroscopic analysis of the mixture indicated >20:1 d.r. The product was analyzed by HPLC to determine the enantiomeric excess: >99% e.e. (CHIRALPAK IE, hexane/*i*-PrOH = 90/10, detector: 290 nm, T = 25 °C, flow rate: 1 mL/min), t_1 (major) = 6.72 min, t_2 (minor) = 7.16 min.

(2*R*,3*S*)-2-phenethyl-3-phenyl-1-(1-phenyl-1H-imidazol-2-yl)pent-4-en-1-one (4h)

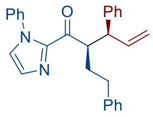

Reaction time: 2.5 d. Yield: 83%. **^{1}H NMR (400 MHz, CDCl$_3$)** δ 7.50 – 7.42 (m, 3H), 7.34 – 7.32 (m, 1H), 7.31 – 7.18 (m, 8H), 7.17 – 7.11 (m, 2H), 7.11 – 7.05 (m, 1H), 6.99 – 6.90 (m, 2H), 6.05 – 5.91 (m, 1H), 4.98 (d, *J* = 17.0 Hz, 1H), 4.90 – 4.82 (m, 1H), 4.60 – 4.46 (m, 1H), 3.65 – 3.55 (m, 1H), 2.50 – 2.36 (m, 2H), 2.01 – 1.83 (m, 1H), 1.65 – 1.53 (m, 1H). **^{13}C NMR (100 MHz, CDCl$_3$)** δ 194.41, 144.45, 142.09, 140.46, 138.74, 129.86, 129.05, 128.81, 128.45, 128.28, 128.24, 127.44, 126.78, 126.03, 125.80, 115.20, 53.86, 50.82, 33.70, 32.81. **ESI-MS:** calculated [$C_{28}H_{26}N_2O$ + H]$^+$: 407.2118, found: 407.2128. $[\alpha]^{20}_D$= 11.9 (c = 0.71, CH_2Cl_2). ^{1}H NMR spectroscopic analysis of the mixture indicated >20:1 d.r. The product was analyzed by HPLC to determine the enantiomeric excess: 98% e.e. (CHIRALPAK IG, hexane/*i*-PrOH = 80/20, detector: 300 nm, T = 25 °C, flow rate: 1 mL/min), t_1 (major) = 4.86 min, t_2 (minor) = 7.41 min.

(2*R*,3*R*)-3-phenyl-1-(1-phenyl-1H-imidazol-2-yl)-2-(p-tolyloxy)pent-4-en-1-one (4i)

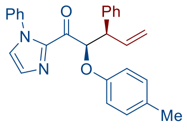

Reaction time: 1 d. Yield: 95%. **^{1}H NMR (500 MHz, CDCl$_3$)** δ 7.39 – 7.25 (m, 8H), 7.24 – 7.19 (m, 2H), 6.99 – 6.87 (m, 4H), 6.83 – 6.74 (m, 2H), 6.31 (d, *J* = 7.3 Hz, 1H), 6.21 – 6.10 (m, 1H), 5.11 (d, *J* = 17.0 Hz, 1H), 5.03 (d, *J* = 10.1 Hz, 1H), 4.30 – 4.22 (m, 1H), 2.19 (s, 3H). **^{13}C NMR (100 MHz, CDCl$_3$)** δ 188.10, 156.01, 142.28, 140.08, 137.80, 137.66, 130.53, 130.15, 129.85, 128.97, 128.79, 128.69, 128.63, 127.26, 127.01, 125.59, 116.78, 115.42, 81.10, 53.92, 20.55. **ESI-MS:** calculated [$C_{27}H_{24}N_2O_2$ + H]$^+$: 409.1911, found: 409.1907. $[\alpha]^{20}_D$= -9.8 (c = 1.37, CH_2Cl_2). ^{1}H NMR spectroscopic analysis of the mixture indicated >20:1 d.r. The

product was analyzed by HPLC to determine the enantiomeric excess: >99% e.e. (CHIRALPAK IG, hexane/*i*-PrOH = 80/20, detector: 300 nm, T = 25 °C, flow rate: 1 mL/min), t_1 (major) = 6.64 min, t_2 (minor) = 15.09 min.

(2*S*,3*S*)-2,3-diphenyl-1-(1-phenyl-1H-imidazol-2-yl)pent-4-en-1-one (4j)

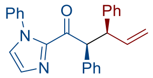

Reaction time: 1 d. T: 0 °C. Yield: 98%. **^{1}H NMR (400 MHz, $CDCl_3$)** δ 7.46 – 7.37 (m, 3H), 7.29 (s, 1H), 7.27 – 7.23 (m, 2H), 7.15 – 7.05 (m, 9H), 7.04 – 6.98 (m, 2H), 6.17 – 6.02 (m, 1H), 5.68 (d, *J* = 11.8 Hz, 1H), 5.18 (d, *J* = 17.1 Hz, 1H), 5.02 (d, *J* = 10.3 Hz, 1H), 4.28 – 4.13 (m, 1H). **^{13}C NMR (100 MHz, $CDCl_3$)** δ 190.19, 143.45, 141.19, 140.22, 138.44, 136.80, 129.91, 129.61, 129.06, 128.77, 128.63, 128.27, 128.22, 127.41, 126.90, 126.30, 125.71, 115.72, 57.32, 52.70. **ESI-MS:** calculated $[C_{26}H_{22}N_2O + H]^+$: 379.1805, found: 379.1811. $[\alpha]^{20}_D$= 145.2 (c = 0.88, CH_2Cl_2). ^{1}H NMR spectroscopic analysis of the mixture indicated >20:1 d.r. The product was analyzed by HPLC to determine the enantiomeric excess: >99% e.e. (CHIRALPAK IG, hexane/*i*-PrOH = 95/5, detector: 280 nm, T = 25 °C, flow rate: 1 mL/min), t_1 (major) = 8.62 min, t_2 (minor) = 11.01 min.

(2*S*,3*S*)-3-phenyl-1-(1-phenyl-1H-imidazol-2-yl)-2-(p-tolyl)pent-4-en-1-one (4k)

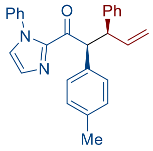

Reaction time: 1 d. T: 0 °C. Yield: 93%. **^{1}H NMR (400 MHz, $CDCl_3$)** δ 7.45 – 7.37 (m, 3H), 7.28 (s, 1H), 7.18 – 7.07 (m, 9H), 7.06 – 6.99 (m, 1H), 6.89 (d, *J* = 7.9 Hz, 2H), 6.13 – 6.01 (m, 1H), 5.65 (d, *J* = 11.8 Hz, 1H), 5.16 (d, *J* = 17.2 Hz, 1H), 5.01 (d, *J* = 10.3 Hz, 1H), 4.25 – 4.13 (m, 1H), 2.15 (s, 3H). **^{13}C NMR (100 MHz, $CDCl_3$)** δ 190.32, 143.47, 141.36, 140.39, 138.49, 136.43, 133.65, 129.88, 129.46, 129.05, 128.74, 128.66, 128.23, 128.23, 127.32, 126.25, 125.72, 115.63, 56.85, 52.57, 21.10. **ESI-MS:** calculated $[C_{27}H_{24}N_2O + H]^+$: 393.1961, found: 393.1971. $[\alpha]^{20}_D$= 167.6 (c = 0.95, CH_2Cl_2). ^{1}H NMR spectroscopic analysis of the mixture indicated >20:1 d.r. The product was analyzed by HPLC to determine the enantiomeric excess: 99% e.e. (CHIRALPAK IA, hexane/*i*-PrOH = 98/2, detector: 300 nm, T = 25 °C, flow rate: 1 mL/min), t_1 (major) = 8.18 min, t_2 (minor) = 8.86 min.

(2*S*,3*S*)-2-(4-fluorophenyl)-3-phenyl-1-(1-phenyl-1H-imidazol-2-yl)pent-4-en-1-one (4l)

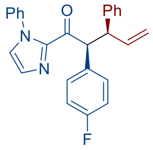

Reaction time: 1 d. T: 0 °C. Yield: 95%. **^{1}H NMR (400 MHz, $CDCl_3$)** δ 7.47 – 7.39 (m, 3H), 7.30 (d, *J* = 0.9 Hz, 1H), 7.24 – 7.18 (m, 2H), 7.17 – 6.99 (m, 8H), 6.82 – 6.72 (m, 2H), 6.14 – 6.01 (m, 1H), 5.68 (d, *J* = 11.8 Hz, 1H), 5.22 – 5.12 (m, 1H), 5.02 (d, *J* = 10.3 Hz, 1H), 4.21 – 4.09 (m, 1H). **^{13}C NMR (100 MHz, $CDCl_3$)** δ 190.13, 161.84 (d, *J* = 245.5 Hz), 143.30, 141.03, 140.02, 138.41, 132.67 (d, *J* = 3.2 Hz), 131.04 (d, *J* = 7.9 Hz), 129.99, 129.10, 128.87, 128.57, 128.35, 127.61, 126.44, 125.74, 115.84, 115.16 (d, *J* = 21.3 Hz), 56.37, 52.93. **^{19}F NMR (565 MHz, $CDCl_3$)** δ -115.68. **ESI-MS:** calculated $[C_{26}H_{21}FN_2O + H]^+$: 397.1711, found: 397.1709. $[\alpha]^{20}_D$= 125.7 (c = 1.09, CH_2Cl_2). ^{1}H NMR spectroscopic analysis of the mixture indicated >20:1 d.r. The product was analyzed by HPLC to determine the enantiomeric excess: 99% e.e. (CHIRALPAK IA, hexane/*i*-PrOH = 98/2, detector: 290 nm, T = 25 °C, flow rate: 1 mL/min), t_1 (minor) = 8.36 min, t_2 (major) = 9.04 min.

(2*S*,3*S*)-2,3-diphenyl-1-(1-(thiophen-2-yl)-1H-imidazol-2-yl)pent-4-en-1-one (4m)

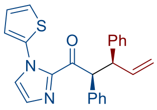

Reaction time: 1 d. T: 0 °C. Yield: 91%. **^{1}H NMR (400 MHz, CDCl$_3$)** δ 7.29 – 7.21 (m, 4H), 7.18 – 7.15 (m, 1H), 7.13 – 7.05 (m, 6H), 7.05 – 6.99 (m, 2H), 6.99 – 6.95 (m, 1H), 6.91 – 6.86 (m, 1H), 6.15 – 5.99 (m, 1H), 5.63 (d, *J* = 11.8 Hz, 1H), 5.17 (d, *J* = 17.1 Hz, 1H), 5.02 (d, *J* = 10.3 Hz, 1H), 4.20 (dd, *J* = 11.7, 7.7 Hz, 1H). **^{13}C NMR (100 MHz, CDCl$_3$)** δ 189.93, 144.20, 141.10, 139.97, 138.64, 136.60, 129.88, 129.56, 128.71, 128.60, 128.29, 128.23, 126.94, 126.33, 125.64, 124.54, 124.35, 115.88, 57.37, 52.79. **ESI-MS:** calculated [$C_{24}H_{20}N_2OS$ + H]$^+$: 385.1369, found: 385.1378. $[\alpha]^{20}_D$= 117.1 (c = 0.86, CH_2Cl_2). ^{1}H NMR spectroscopic analysis of the mixture indicated >20:1 d.r. The product was analyzed by HPLC to determine the enantiomeric excess: >99% e.e. (CHIRALPAK IG, hexane/*i*-PrOH = 95/5, detector: 300 nm, T = 25 °C, flow rate: 1 mL/min), t_1 (major) = 7.92 min, t_2 (minor) = 9.95 min.

(2*S*,3*S*)-1-(1-methyl-1H-imidazol-2-yl)-2,3-diphenylpent-4-en-1-one (4n)

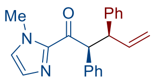

Reaction time: 2.5 d. Yield: 90%. **^{1}H NMR (500 MHz, CDCl$_3$)** δ 7.32 – 7.28 (m, 2H), 7.17 (s, 1H), 7.13 – 7.06 (m, 6H), 7.05 – 6.97 (m, 3H), 6.13 – 6.02 (m, 1H), 5.69 (d, *J* = 11.8 Hz, 1H), 5.23 – 5.15 (m, 1H), 4.99 (d, *J* = 10.3 Hz, 1H), 4.35 – 4.26 (m, 1H), 3.94 (s, 3H). **^{13}C NMR (100 MHz, CDCl$_3$)** δ 191.79, 143.34, 141.25, 140.19, 137.11, 129.51, 129.43, 128.64, 128.26, 128.23, 127.64, 126.85, 126.30, 115.70, 56.89, 52.77, 36.35. **ESI-MS:** calculated [$C_{21}H_{20}N_2O$ + H]$^+$: 317.1648, found: 317.1643. $[\alpha]^{20}_D$= 28.4 (c = 0.49, CH_2Cl_2). ^{1}H NMR spectroscopic analysis of the mixture indicated >20:1 d.r. The product was analyzed by HPLC to determine the enantiomeric excess: >99% e.e. (CHIRALPAK IA, hexane/*i*-PrOH = 99/1, detector: 283.3 nm, T = 25 °C, flow rate: 0.5 mL/min), t_1 (minor) = 21.97 min, t_2 (major) = 29.88 min.

(2*S*,3*S*)-1-(1-isopropyl-1H-imidazol-2-yl)-2,3-diphenylpent-4-en-1-one (4o)

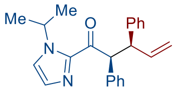

Reaction time: 1 d. T: 0 °C. Yield: 77%. **^{1}H NMR (400 MHz, CDCl$_3$)** δ 7.32 – 7.27 (m, 2H), 7.21 (d, *J* = 11.7 Hz, 2H), 7.14 – 7.05 (m, 6H), 7.05 – 6.98 (m, 2H), 6.15 – 6.01 (m, 1H), 5.74 (d, *J* = 11.8 Hz, 1H), 5.57 – 5.46 (m, 1H), 5.18 (d, *J* = 17.1 Hz, 1H), 4.98 (d, *J* = 10.3 Hz, 1H), 4.35 – 4.24 (m, 1H), 1.42 (d, *J* = 6.6 Hz, 3H), 1.31 (d, *J* = 6.7 Hz, 3H). **^{13}C NMR (100 MHz, CDCl$_3$)** δ 191.96, 142.80, 141.32, 140.28, 137.25, 129.82, 129.46, 128.62, 128.22, 126.77, 126.28, 121.72, 115.59, 57.40, 53.08, 49.41, 23.85, 23.36. **ESI-MS:** calculated [$C_{23}H_{24}N_2O$ + H]$^+$: 345.1961, found: 345.1968. $[\alpha]^{20}_D$= 32.3 (c = 0.66, CH_2Cl_2). ^{1}H NMR spectroscopic analysis of the mixture indicated >20:1 d.r. The product was analyzed by HPLC to determine the enantiomeric excess: >99% e.e. (CHIRALPAK IE, hexane/*i*-PrOH = 98/2, detector: 300 nm, T = 25 °C, flow rate: 1 mL/min), t_1 (minor) = 9.11 min, t_2 (major) = 9.63 min.

(2*S*,3*S*)-1-(1-(pentan-3-yl)-1H-imidazol-2-yl)-2,3-diphenylpent-4-en-1-one (4p)

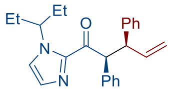

Reaction time: 2 d. T: 0 °C. Yield: 87%. **^{1}H NMR (400 MHz, CDCl$_3$)** δ 7.29 – 7.23 (m, 3H), 7.15 – 6.97 (m, 9H), 6.17 – 6.02 (m, 1H), 5.74 (d, *J* = 11.8 Hz, 1H), 5.30 – 5.22 (m, 1H), 5.18 (d, *J* = 17.2 Hz, 1H), 4.98 (d, *J* = 10.3 Hz, 1H), 4.33 – 4.25 (m, 1H), 1.87 – 1.77 (m, 1H), 1.73 – 1.64 (m, 2H), 1.59 – 1.48 (m, 1H), 0.80 (t, *J* = 7.4 Hz, 3H), 0.47 (t, *J* = 7.4 Hz, 3H). **^{13}C NMR (100 MHz, CDCl$_3$)** δ 192.28, 144.08, 141.36, 140.17, 137.15, 130.10, 129.47, 128.66, 128.21, 128.15, 126.72, 126.25, 121.91, 115.59, 60.17, 57.62, 52.75, 29.08, 29.05, 10.42, 10.09. **ESI-MS:** calculated [$C_{26}H_{22}N_2O$ + H]$^+$: 373.2274, found: 373.2282. $[\alpha]^{20}_D$= 54.3 (c = 0.85, CH_2Cl_2). ^{1}H NMR spectroscopic analysis of the mixture indicated >20:1 d.r. The product was analyzed by HPLC to

determine the enantiomeric excess: >99% e.e. (CHIRALPAK IG, hexane/*i*-PrOH = 98/2, detector: 283 nm, T = 25 °C, flow rate: 1 mL/min), t_1 (minor) = 6.36 min, t_2 (major) = 7.33 min.

(2*R*,3*S*)-2-benzyl-1-(1-methyl-1H-benzo[d]imidazol-2-yl)-3-phenylpent-4-en-1-one (4q)

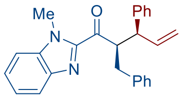

Reaction time: 3 d. T: 0 °C. Yield: 56%. **^{1}H NMR (400 MHz, CDCl$_3$)** δ 7.89 (d, *J* = 8.0 Hz, 1H), 7.45 – 7.31 (m, 7H), 7.26 – 7.22 (m, 1H), 7.08 – 7.01 (m, 4H), 6.99 – 6.92 (m, 1H), 6.11 – 5.94 (m, 1H), 5.12 – 4.92 (m, 2H), 4.83 – 4.74 (m, 1H), 3.94 (s, 3H), 3.83 – 3.73 (m, 1H), 3.08 – 2.95 (m, 1H), 2.81 – 2.69 (m, 1H). **^{13}C NMR (100 MHz, CDCl$_3$)** δ 198.53, 147.53, 142.01, 141.80, 139.97, 139.10, 137.01, 129.04, 128.96, 128.35, 128.10, 126.99, 126.04, 125.76, 123.45, 122.27, 115.50, 110.55, 54.50, 53.28, 37.08, 32.14. **ESI-MS:** calculated [$C_{26}H_{24}N_2O$ + H]$^+$: 381.1961, found: 381.1960. $[\alpha]^{20}_D$= -103.4 (c = 0.47, CH_2Cl_2). ^{1}H NMR spectroscopic analysis of the mixture indicated >20:1 d.r. The product was analyzed by HPLC to determine the enantiomeric excess: >99% e.e. (CHIRALPAK IA, hexane/*i*-PrOH = 98/2, detector: 300 nm, T = 40 °C, flow rate: 0.5 mL/min), t_1 (minor) = 13.69 min, t_2 (major) = 14.44 min.

(2*R*,3*S*)-2-benzyl-3-phenyl-1-(thiazol-2-yl)pent-4-en-1-one (4r)

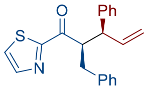

Reaction time: 2.5 d. T: 0 °C. Yield: 60%. **^{1}H NMR (400 MHz, CDCl$_3$)** δ 7.88 (d, *J* = 3.0 Hz, 1H), 7.55 (d, *J* = 3.0 Hz, 1H), 7.43 – 7.32 (m, 4H), 7.27 – 7.22 (m, 1H), 7.09 – 7.03 (m, 2H), 7.03 – 6.95 (m, 3H), 6.04 – 5.91 (m, 1H), 5.00 (d, *J* = 17.0 Hz, 1H), 4.87 – 4.81 (m, 1H), 4.74 – 4.65 (m, 1H), 3.85 – 3.73 (m, 1H), 3.03 – 2.92 (m, 1H), 2.76 – 2.64 (m, 1H). **^{13}C NMR (100 MHz, CDCl$_3$)** δ 196.71, 168.40, 144.72, 141.81, 139.30, 138.97, 129.01, 128.99, 128.25, 128.21, 127.05, 126.53, 126.12, 116.10, 53.94, 53.46, 37.34. **ESI-MS:** calculated [$C_{21}H_{19}NOS$ + H]$^+$: 334.1260, found: 334.1269. $[\alpha]^{20}_D$= -23.6 (c = 0.45, CH_2Cl_2). ^{1}H NMR spectroscopic analysis of the mixture indicated >20:1 d.r. The product was analyzed by HPLC to determine the enantiomeric excess: >99% e.e. (CHIRALPAK AD-H, hexane/*i*-PrOH = 99/1, detector: 300 nm, T = 25 °C, flow rate: 0.5 mL/min), t_1 (minor) = 25.94 min, t_2 (major) = 28.39 min.

(2*R*,3*S*)-2-benzyl-3-phenyl-1-(pyridin-2-yl)pent-4-en-1-one (4s)

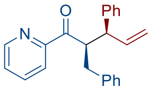

Reaction time: 1 d. T: 0 °C. Yield: 87%. **^{1}H NMR (400 MHz, CDCl$_3$)** δ 8.58 (d, *J* = 4.7 Hz, 1H), 7.85 (d, *J* = 7.8 Hz, 1H), 7.74 – 7.64 (m, 1H), 7.41 – 7.30 (m, 5H), 7.25 – 7.20 (m, 1H), 7.06 – 6.91 (m, 5H), 6.06 – 5.88 (m, 1H), 5.22 – 5.11 (m, 1H), 4.98 (d, *J* = 17.0 Hz, 1H), 4.78 (d, *J* = 10.2 Hz, 1H), 3.75 (t, *J* = 9.5 Hz, 1H), 3.00 – 2.85 (m, 1H), 2.77 – 2.64 (m, 1H). **^{13}C NMR (100 MHz, CDCl$_3$)** δ 204.88, 154.19, 148.75, 142.33, 139.84, 139.46, 136.69, 129.04, 128.88, 128.38, 128.00, 126.83, 126.78, 125.84, 122.13, 115.59, 54.00, 49.85, 37.43. **ESI-MS:** calculated [$C_{23}H_{21}NO$ + H]$^+$: 328.1696, found: 328.1703. $[\alpha]^{20}_D$= -51.0 (c = 0.51, CH_2Cl_2). ^{1}H NMR spectroscopic analysis of the mixture indicated >20:1 d.r. The product was analyzed by HPLC to determine the enantiomeric excess: 97% e.e. (CHIRALPAK OD-H, hexane/*i*-PrOH = 99/1, detector: 276 nm, T = 25 °C, flow rate: 1 mL/min), t_1 (minor) = 7.64 min, t_2 (major) = 8.55 min.

(2*R*,3*S*)-3-(4-(tert-butyl)phenyl)-1-(1-phenyl-1H-imidazol-2-yl)-2-propylpent-4-en-1-one (4t)

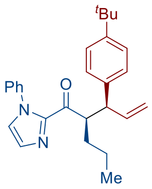

Reaction time: 6 d. Yield: 70%. **^{1}H NMR (400 MHz, $CDCl_3$)** δ 7.50 – 7.40 (m, 3H), 7.35 – 7.28 (m, 3H), 7.26 – 7.22 (m, 2H), 7.20 – 7.15 (m, 3H), 6.04 – 5.89 (m, 1H), 5.01 – 4.91 (m, 1H), 4.90 – 4.79 (m, 1H), 4.50 – 4.38 (m, 1H), 3.60 – 3.45 (m, 1H), 1.63 – 1.50 (m, 1H), 1.31 – 1.13 (m, 12H), 0.73 (t, *J* = 7.2 Hz, 3H). **^{13}C NMR (100 MHz, $CDCl_3$)** δ 195.22, 149.39, 144.62, 140.80, 139.25, 138.85, 129.76, 129.01, 128.71, 127.79, 127.25, 126.03, 125.59, 114.80, 53.67, 50.75, 34.53, 33.34, 31.54, 20.77, 14.32. **ESI-MS:** calculated $[C_{27}H_{32}N_2O + H]^+$: 401.2587, found: 401.2591. $[\alpha]^{20}_D$= -52.2 (c = 0.89, CH_2Cl_2). ^{1}H NMR spectroscopic analysis of the mixture indicated >20:1 d.r. The product was analyzed by HPLC to determine the enantiomeric excess: 98% e.e. (CHIRALPAK IG, hexane/*i*-PrOH = 95/5, detector: 254 nm, T = 25 °C, flow rate: 1 mL/min), t_1 (major) = 4.29 min, t_2 (minor) = 4.70 min.

(2*R*,3*S*)-3-(4-methoxyphenyl)-1-(1-phenyl-1H-imidazol-2-yl)-2-propylpent-4-en-1-one (4u)

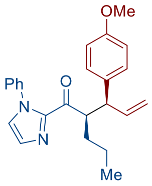

Reaction time: 4 d. Yield: 86%. **^{1}H NMR (400 MHz, $CDCl_3$)** δ 7.49 – 7.41 (m, 3H), 7.31 (d, *J* = 0.8 Hz, 1H), 7.26 – 7.22 (m, 2H), 7.21 – 7.15 (m, 3H), 6.89 – 6.81 (m, 2H), 6.02 – 5.89 (m, 1H), 4.94 (d, *J* = 17.0 Hz, 1H), 4.87 – 4.81 (m, 1H), 4.44 – 4.36 (m, 1H), 3.79 (s, 3H), 3.54 – 3.45 (m, 1H), 1.61 – 1.49 (m, 1H), 1.27 – 1.11 (m, 3H), 0.72 (t, *J* = 7.2 Hz, 3H). **^{13}C NMR (100 MHz, $CDCl_3$)** δ 195.14, 158.36, 144.56, 140.84, 138.82, 134.54, 129.77, 129.17, 129.02, 128.73, 127.30, 126.01, 114.69, 114.14, 55.35, 53.11, 50.89, 33.42, 20.68, 14.32. **ESI-MS:** calculated $[C_{24}H_{26}N_2O_2 + H]^+$: 375.2067, found: 375.2072. $[\alpha]^{20}_D$= -44.2 (c = 0.87, CH_2Cl_2). ^{1}H NMR spectroscopic analysis of the mixture indicated >20:1 d.r. The product was analyzed by HPLC to determine the enantiomeric excess: 99% e.e. (CHIRALPAK IBN-5, hexane/*i*-PrOH = 90/10, detector: 286 nm, T = 25 °C, flow rate: 0.5 mL/min), t_1 (minor) = 10.63 min, t_2 (major) = 11.23 min.

(2*R*,3*S*)-3-(4-fluorophenyl)-1-(1-phenyl-1H-imidazol-2-yl)-2-propylpent-4-en-1-one (4v)

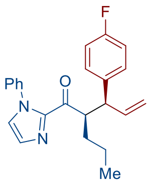

Reaction time: 5 d. Yield: 58%. **^{1}H NMR (400 MHz, $CDCl_3$)** δ 7.49 – 7.41 (m, 3H), 7.31 (d, *J* = 0.4 Hz, 1H), 7.26 – 7.16 (m, 5H), 7.05 – 6.96 (m, 2H), 6.02 – 5.87 (m, 1H), 4.96 (d, *J* = 17.0 Hz, 1H), 4.91 – 4.81 (m, 1H), 4.46 – 4.34 (m, 1H), 3.62 – 3.49 (m, 1H), 1.60 – 1.48 (m, 1H), 1.23 – 1.09 (m, 3H), 0.72 (t, *J* = 7.1 Hz, 3H). **^{13}C NMR (100 MHz, $CDCl_3$)** δ 194.76, 161.72 (d, *J* = 244.5 Hz), 144.47, 140.35, 138.78, 138.17 (d, *J* = 3.0 Hz), 129.85, 129.68 (d, *J* = 7.8 Hz), 129.07, 128.81, 127.43, 126.03, 115.54 (d, *J* = 21.1 Hz), 115.27, 53.05, 50.81, 33.44, 20.64, 14.31. **^{19}F NMR (565 MHz, $CDCl_3$)** δ -116.44. **ESI-MS:** calculated $[C_{23}H_{23}FN_2O + H]^+$: 363.1867, found: 363.1870. $[\alpha]^{20}_D$= -30.4 (c = 0.38, CH_2Cl_2). ^{1}H NMR spectroscopic analysis of the mixture indicated >20:1 d.r. The product was analyzed by HPLC to determine the enantiomeric excess: 98% e.e. (CHIRALPAK IG, hexane/*i*-PrOH = 98/2, detector: 254 nm, T = 25 °C, flow rate: 1 mL/min), t_1 (major) = 7.18 min, t_2 (minor) = 7.75 min.

(2*R*,3*S*)-3-(4-chlorophenyl)-1-(1-phenyl-1H-imidazol-2-yl)-2-propylpent-4-en-1-one (4w)

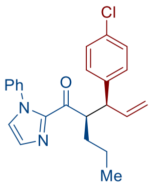

Reaction time: 7 d. Yield: 69%. **^{1}H NMR (400 MHz, $CDCl_3$)** δ 7.50 – 7.42 (m, 3H), 7.34 – 7.27 (m, 3H), 7.26 – 7.17 (m, 5H), 6.02 – 5.86 (m, 1H), 4.96 (d, *J* = 17.0 Hz, 1H), 4.92 – 4.85 (m, 1H), 4.48 – 4.35 (m, 1H), 3.59 – 3.47 (m, 1H), 1.62 – 1.47 (m, 1H), 1.22 – 1.05 (m, 3H), 0.73 (t, *J* = 7.1 Hz, 3H). **^{13}C NMR (100 MHz, $CDCl_3$)** δ 194.59, 144.43, 141.00, 140.04, 138.74, 132.38, 129.86, 129.64, 129.07, 128.89, 128.82, 127.46, 126.01,

115.53, 53.22, 50.62, 33.43, 20.63, 14.29. **ESI-MS:** calculated $[C_{23}H_{23}ClN_2O + H]^+$: 379.1572, found: 379.1576. $[\alpha]^{20}_D$= -55.0 (c = 0.51, CH_2Cl_2). ^{1}H NMR spectroscopic analysis of the mixture indicated >20:1 d.r. The product was analyzed by HPLC to determine the enantiomeric excess: 98% e.e. (CHIRALPAK IC, hexane/*i*-PrOH = 99/1, detector: 290 nm, T = 25 °C, flow rate: 0.5 mL/min), t_1 (minor) = 11.30 min, t_2 (major) = 13.11 min.

(2*R*,3*S*)-3-(4-bromophenyl)-1-(1-phenyl-1H-imidazol-2-yl)-2-propylpent-4-en-1-one (4x)

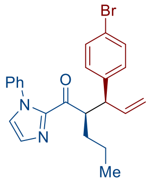

Reaction time: 6 d. Yield: 65%. **^{1}H NMR (400 MHz, $CDCl_3$)** δ 7.49 – 7.40 (m, 5H), 7.31 (d, *J* = 0.9 Hz, 1H), 7.25 – 7.20 (m, 2H), 7.18 (d, *J* = 0.9 Hz, 1H), 7.17 – 7.12 (m, 2H), 6.00 – 5.85 (m, 1H), 4.99 – 4.92 (m, 1H), 4.90 – 4.84 (m, 1H), 4.48 – 4.35 (m, 1H), 3.59 – 3.46 (m, 1H), 1.62 – 1.48 (m, 1H), 1.23 – 1.09 (m, 3H), 0.73 (t, *J* = 7.1 Hz, 3H). **^{13}C NMR (100 MHz, $CDCl_3$)** δ 194.55, 144.41, 141.53, 139.96, 138.73, 131.84, 130.04, 129.86, 129.07, 128.82, 127.46, 126.01, 120.45, 115.58, 53.29, 50.55, 33.42, 20.63, 14.28. **ESI-MS:** calculated $[C_{23}H_{23}BrN_2O + Na]^+$: 445.0886, found: 445.0895. $[\alpha]^{20}_D$= -50.1 (c = 0.85, CH_2Cl_2). ^{1}H NMR spectroscopic analysis of the mixture indicated >20:1 d.r. The product was analyzed by HPLC to determine the enantiomeric excess: 98% e.e. (CHIRALPAK IC, hexane/*i*-PrOH = 99/1, detector: 254 nm, T = 25 °C, flow rate: 0.5 mL/min), t_1 (minor) = 12.11 min, t_2 (major) = 13.96 min.

(2*R*,3*S*)-1-(1-phenyl-1H-imidazol-2-yl)-2-propyl-3-(m-tolyl)pent-4-en-1-one (4y)

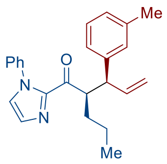

Reaction time: 2 d. Yield: 75%. **^{1}H NMR (400 MHz, $CDCl_3$)** δ 7.49 – 7.42 (m, 3H), 7.32 (d, *J* = 0.7 Hz, 1H), 7.26 – 7.16 (m, 4H), 7.10 – 7.00 (m, 3H), 6.04 – 5.90 (m, 1H), 4.96 (d, *J* = 17.0 Hz, 1H), 4.89 – 4.81 (m, 1H), 4.51 – 4.39 (m, 1H), 3.54 – 3.44 (m, 1H), 2.34 (s, 3H), 1.61 – 1.49 (m, 1H), 1.26 – 1.11 (m, 3H), 0.72 (t, *J* = 7.1 Hz, 3H). **^{13}C NMR (100 MHz, $CDCl_3$)** δ 195.10, 144.60, 142.37, 140.72, 138.83, 138.33, 129.78, 129.03, 128.85, 128.74, 128.60, 127.43, 127.32, 126.03, 125.36, 114.93, 54.06, 50.70, 33.41, 21.59, 20.70, 14.31. **ESI-MS:** calculated $[C_{24}H_{26}N_2O + H]^+$: 359.2118, found: 359.2128. $[\alpha]^{20}_D$= -41.2 (c = 0.65, CH_2Cl_2). ^{1}H NMR spectroscopic analysis of the mixture indicated >20:1 d.r. The product was analyzed by HPLC to determine the enantiomeric excess: 99% e.e. (CHIRALPAK IG, hexane/*i*-PrOH =98/2, detector: 254 nm, T = 25 °C, flow rate: 1 mL/min), t_1 (major) = 6.73 min, t_2 (minor) = 7.73 min.

(2*R*,3*S*)-3-(3-methoxyphenyl)-1-(1-phenyl-1H-imidazol-2-yl)-2-propylpent-4-en-1-one (4z)

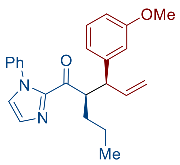

Reaction time: 7 d. Yield: 86%. **^{1}H NMR (600 MHz, $CDCl_3$)** δ 7.47 – 7.44 (m, 3H), 7.32 (s, 1H), 7.26 – 7.22 (m, 3H), 7.18 (s, 1H), 6.88 (d, *J* = 7.6 Hz, 1H), 6.83 – 6.80 (m, 1H), 6.78 – 6.74 (m, 1H), 6.04 – 5.91 (m, 1H), 5.02 – 4.93 (m, 1H), 4.90 – 4.83 (m, 1H), 4.48 – 4.38 (m, 1H), 3.81 (s, 3H), 3.56 – 3.47 (m, 1H), 1.60 – 1.51 (m, 1H), 1.26 – 1.21 (m, 1H), 1.19 – 1.12 (m, 2H), 0.73 (t, *J* = 7.2 Hz, 3H). **^{13}C NMR (150 MHz, CHLOROFORM-D)** δ 195.03, 159.88, 144.47, 144.04, 140.37, 138.75, 129.78, 129.71, 129.03, 128.75, 127.37, 125.99, 120.59, 115.16, 113.97, 111.90, 55.33, 54.08, 50.61, 33.42, 20.71, 14.32. **ESI-MS:** calculated $[C_{24}H_{27}N_2O_2 + H]^+$: 375.2607, found: 375.2607. $[\alpha]^{20}_D$= -32.6 (c = 0.95, CH_2Cl_2). ^{1}H NMR spectroscopic analysis of the mixture indicated >20:1 d.r. The product was analyzed by HPLC to determine the enantiomeric excess: 99% e.e. (CHIRALPAK IG, hexane/*i*-PrOH = 95/5, detector: 280 nm, T = 25 °C, flow rate: 1.0 mL/min), t_1 (major) = 7.36 min, t_2 (minor) = 8.74 min.

(2*R*,3*S*)-3-(3-fluorophenyl)-1-(1-phenyl-1H-imidazol-2-yl)-2-propylpent-4-en-1-one (4aa)

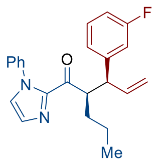

Reaction time: 5 d. Yield: 56%. **^{1}H NMR (400 MHz, CDCl$_3$)** δ 7.53 – 7.40 (m, 3H), 7.32 – 7.26 (m, 2H), 7.25 – 7.22 (m, 2H), 7.18 (d, *J* = 0.9 Hz, 1H), 7.08 – 7.03 (m, 1H), 7.02 – 6.96 (m, 1H), 6.94 – 6.87 (m, 1H), 6.03 – 5.86 (m, 1H), 4.98 (d, *J* = 17.0 Hz, 1H), 4.94 – 4.86 (m, 1H), 4.49 – 4.37 (m, 1H), 3.61 – 3.49 (m, 1H), 1.62 – 1.47 (m, 1H), 1.25 – 1.08 (m, 3H), 0.73 (t, *J* = 7.1 Hz, 3H). **^{13}C NMR (100 MHz, CDCl$_3$)** δ 194.55, 163.17 (d, *J* = 245.8 Hz), 145.10 (d, *J* = 6.8 Hz), 144.44, 139.85, 138.75, 130.15 (d, *J* = 8.3 Hz), 129.87, 129.07, 128.81, 127.45, 126.02, 123.99 (d, *J* = 2.5 Hz), 115.67, 115.10 (d, *J* = 21.2 Hz), 113.58 (d, *J* = 21.0 Hz), 53.61, 50.60, 33.43, 20.65, 14.30. **^{19}F NMR (565 MHz, CDCl$_3$)** δ -113.01. **ESI-MS:** calculated $[C_{23}H_{23}FN_2O + H]^+$: 363.1867, found: 363.1877. $[\alpha]^{20}_{D}$= -30.3 (c = 0.66, CH_2Cl_2). ^{1}H NMR spectroscopic analysis of the mixture indicated >20:1 d.r. The product was analyzed by HPLC to determine the enantiomeric excess: >99% e.e. (CHIRALPAK IC, hexane/*i*-PrOH = 99/1, detector: 300 nm, T = 25 °C, flow rate: 0.5 mL/min), t_1 (minor) = 11.05 min, t_2 (major) = 13.99 min.

(2*R*,3*S*)-3-(naphthalen-2-yl)-1-(1-phenyl-1H-imidazol-2-yl)-2-propylpent-4-en-1-one (4ab)

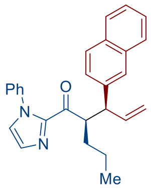

Reaction time: 4 d. Yield: 92%. **^{1}H NMR (400 MHz, CDCl$_3$)** δ 7.87 – 7.77 (m, 3H), 7.70 (s, 1H), 7.49 – 7.41 (m, 6H), 7.36 – 7.33 (m, 1H), 7.27 – 7.23 (m, 2H), 7.21 – 7.17 (m, 1H), 6.19 – 5.95 (m, 1H), 5.11 – 4.97 (m, 1H), 4.90 (d, *J* = 10.2 Hz, 1H), 4.64 – 4.53 (m, 1H), 3.77 – 3.66 (m, 1H), 1.68 – 1.55 (m, 1H), 1.27 – 1.12 (m, 3H), 0.68 (t, *J* = 7.1 Hz, 3H). **^{13}C NMR (100 MHz, CDCl$_3$)** δ 195.00, 144.53, 140.40, 139.91, 138.75, 133.77, 132.53, 129.84, 129.06, 128.78, 128.46, 127.83, 127.75, 127.44, 126.87, 126.47, 126.12, 126.01, 125.61, 115.36, 54.12, 50.61, 33.55, 20.71, 14.30. **ESI-MS:** calculated $[C_{27}H_{26}N_2O + H]^+$: 395.2118, found: 395.2129. $[\alpha]^{20}_{D}$= -59.6 (c = 0.77, CH_2Cl_2). ^{1}H NMR spectroscopic analysis of the mixture indicated >20:1 d.r. The product was analyzed by HPLC to determine the enantiomeric excess: 99% e.e. (CHIRALPAK IG, hexane/*i*-PrOH = 95/5, detector: 300 nm, T = 25 °C, flow rate: 1 mL/min), t_1 (major) = 6.58 min, t_2 (minor) = 7.17 min.

(2*R*,3*S*)-1-(1-phenyl-1H-imidazol-2-yl)-2-propyl-3-(thiophen-2-yl)pent-4-en-1-one (4ac)

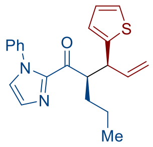

Reaction time: 5 d. Yield: 70%. **^{1}H NMR (400 MHz, CDCl3)** δ 7.49 – 7.40 (m, 3H), 7.33 – 7.29 (m, 1H), 7.24 – 7.19 (m, 2H), 7.19 – 7.15 (m, 2H), 6.96 – 6.92 (m, 1H), 6.89 – 6.87 (m, 1H), 6.04 – 5.93 (m, 1H), 5.06 – 4.96 (m, 1H), 4.96 – 4.88 (m, 1H), 4.41 – 4.32 (m, 1H), 3.96 – 3.87 (m, 1H), 1.67 – 1.57 (m, 1H), 1.44 – 1.35 (m, 1H), 1.24 – 1.14 (m, 2H), 0.78 (t, *J* = 7.3 Hz, 3H). **^{13}C NMR (100 MHz, CDCl$_3$)** δ 194.29, 145.79, 144.31, 140.02, 138.73, 129.86, 129.05, 128.77, 127.36, 126.81, 125.98, 124.69, 123.92, 115.55, 51.91, 48.58, 33.40, 20.66, 14.31. **ESI-MS:** calculated $[C_{21}H_{22}N_2OS + H]^+$: 351.1526, found: 351.1533. $[\alpha]^{20}_{D}$= -23.6 (c = 0.53, CH_2Cl_2). ^{1}H NMR spectroscopic analysis of the mixture indicated 7:1 d.r. The product was analyzed by HPLC to determine the enantiomeric excess: 87% e.e. (CHIRALPAK IG, hexane/*i*-PrOH = 99/1, detector: 254 nm, T = 25 °C, flow rate: 1 mL/min), t_1 (major) = 12.94 min, t_2 (minor) = 14.15 min.

Supplementary Table 2. Optimization studies with alkyl-substituted allylic carbonates

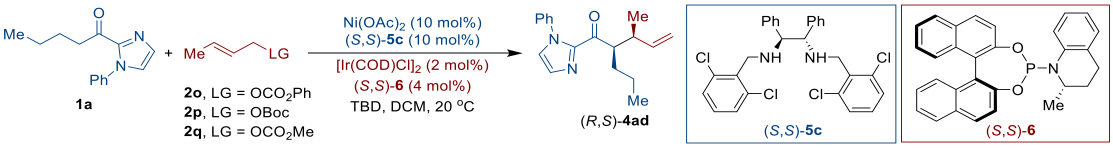

Entry	2	LG	Yield (%)	Branched/linear	d.r. of (*R*,*S*)-**4ad**	e.e. of (*R*,*S*)-**4ad** (%)
1	**2o**	OCO_2Ph	NR	—	—	—
2	**2p**	$OCO_2{}^tBu$	79	3.8:1	11:1	99
3	**2q**	OCO_2Me	78	4.6:1	10:1	99

Unless otherwise specified, all the reactions were carried out as below: In a 10 mL Schlenk tube, $[Ir(COD)Cl]_2$ (0.002 mmol, 0.02 equiv.), (*R*,*R*)-**6** (0.004 mmol, 0.04 equiv.), and TBD (0.01 mmol, 0.1 equiv.) were stirred in 1.0 mL DCM under argon at 25 °C for 10min. **1a** (0.1 mmol, 1.0 equiv.), allylic carbonate **2** (0.15 mmol, 1.5 equiv.), and (*S*,*S*)-Ni catalyst (0.01 mmol, 0.1 equiv.) were then added successively and additional DCM (1.0 mL) was added. The mixture was stirred at 20 °C. After the reaction was complete (monitored by TLC), the residue was purified by flash column chromatography on silica gel to afford the desired product **4ad**.

(2*R*,3*S*)-3-methyl-1-(1-phenyl-1H-imidazol-2-yl)-2-propylpent-4-en-1-one) (4ad)

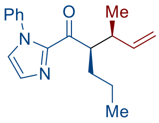

In a 10 mL Schlenk tube, $[Ir(COD)Cl]_2$ (0.002 mmol, 0.02 equiv.), (*R*,*R*)-**6** (0.004 mmol, 0.04 equiv.), and TBD (0.01 mmol, 0.1 equiv.) were stirred in 1.0 mL DCM under argon at 25 °C for 10min. **1a** (0.1 mmol, 1.0 equiv.), allylic carbonate **2q** (0.15 mmol, 1.5 equiv.), and (*S*,*S*)-Ni catalyst (0.01 mmol, 0.1 equiv.) were then added successively and additional DCM (1.0 mL) was added. The mixture was stirred at 20 °C. After the reaction was complete (monitored by TLC), the residue was purified by flash column chromatography on silica gel to afford the desired product **4ad**. Reaction time: 2 d. Yield: 78%. Branched/linear = 4.6:1. **^{1}H NMR (400 MHz, CDCl3)** δ 7.46 – 7.44 (m, 3H), 7.28 – 7.27 (m, 1H), 7.26 – 7.23 (m, 2H), 7.17 – 7.15 (m, 1H), 5.87 – 5.76 (m, 1H), 4.98 – 4.88 (m, 2H), 3.94 – 3.89 (m, 1H), 2.59 – 2.52 (m, 1H), 1.72 – 1.66 (m, 1H), 1.49 – 1.44 (m, 1H), 1.26 – 1.21 (m, 2H), 1.02 (d, *J* = 6.8 Hz, 3H), 0.87 (t, *J* = 7.2 Hz, 3H). **^{13}C NMR (100 MHz, $CDCl_3$)** δ 194.95, 144.12, 142.41, 138.79, 129.56, 129.06, 128.72, 127.19, 125.94, 114.00, 51.26, 40.68, 30.90, 21.04, 17.14, 14.50. **ESI-MS:** calculated $[C_{18}H_{22}N_2O + H]^+$: 283.1805, found: 283.1805. $[\alpha]^{20}_D$= -4.5 (c = 0.23, CH_2Cl_2). ^{1}H NMR spectroscopic analysis of the mixture indicated 10:1 d.r. The product was analyzed by HPLC to determine the enantiomeric excess: 99% e.e. (CHIRALPAK OJ-H, hexane/*i*-PrOH = 98/2, detector: 280 nm, T = 40 °C, flow rate: 0.5 mL/min), t_1 (major) = 18.86 min, t_2 (minor) = 25.18 min.

(2*R*,3*S*)-2-(cyclopentylmethyl)-3-methyl-1-(1-phenyl-1H-imidazol-2-yl)pent-4-en-1-one (4ae)

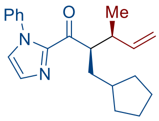

In a 10 mL Schlenk tube, $[Ir(COD)Cl]_2$ (0.002 mmol, 0.02 equiv.), (*R*,*R*)-**6** (0.004 mmol, 0.04 equiv.), and TBD (0.01 mmol, 0.1 equiv.) were stirred in 1.0 mL DCM under argon at 25 °C for 10min. **1e** (0.1 mmol, 1.0 equiv.), allylic carbonate **2q** (0.15 mmol, 1.5 equiv.), and (*S*,*S*)-Ni catalyst (0.01 mmol, 0.1 equiv.) were then added successively and additional DCM (1.0 mL) was added. The mixture was stirred at 20 °C. After the reaction was complete (monitored by TLC), the residue was purified by flash column chromatography on silica gel to afford the desired product **4ae**. Reaction time: 17h. Yield: 85%. Branched/linear= 4.6:1. **^{1}H NMR (400 MHz, CDCl3)** δ 7.46 – 7.43 (m, 3H), 7.29 – 7.27 (m, 1H), 7.26 – 7.23 (m, 2H), 7.16 – 7.14 (m, 1H), 5.87 – 5.76 (m, 1H), 4.97 – 4.88 (m, 2H), 4.01 – 3.95 (m, 1H), 2.58 – 2.49 (m, 1H), 1.86 – 1.79 (m, 2H), 1.68 – 1.52 (m, 6H), 1.45 – 1.39 (m, 2H), 1.11 – 1.06 (m, 1H),

1.01 (d, J = 6.8 Hz, 3H). **^{13}C NMR (100 MHz, CDCl$_3$)** δ 195.01, 144.09, 142.36, 138.85, 129.60, 129.04, 128.70, 127.17, 125.97, 114.00, 50.63, 41.29, 38.73, 34.64, 33.67, 32.58, 25.23, 17.07. **ESI-MS:** calculated [$C_{21}H_{26}N_2O$ + H]$^+$: 323.2118, found: 323.2117. $[\alpha]^{20}_D$= 24.3 (c = 0.20, CH_2Cl_2). ^{1}H NMR spectroscopic analysis of the mixture indicated 14:1 d.r. The product was analyzed by HPLC to determine the enantiomeric excess: >99% e.e. (CHIRALPAK OJ-H, hexane/*i*-PrOH = 98/2, detector: 254 nm, T = 40 °C, flow rate: 0.3 mL/min), t_1 (major) = 19.48 min, t_2 (minor) = 30.43 min.

Further study on the result of reactions with varied reaction times

Supplementary Table 3. Study on the results of 3a

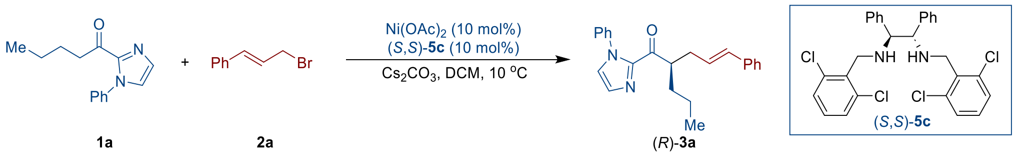

Entry	Reaction time	Yield (%)	e.e. of **3a** (%)
1	2 d	80	95
2	4 d	80	95
3	6 d	81	95

All the reactions were carried out by using **1a** (0.1 mmol), **2a** (0.15 mmol), Ni(OAc)$_2$ (10 mol%), (*S*,*S*)-**5c** (10 mol%), and Cs_2CO_3 (0.2 mmol) in dichloromethane (DCM) (2.0 mL) at 10 °C.

We evaluated alternative reaction times under optimum reaction conditions for the allylic alkylation processes (Figure S3). We found that increasing the reaction times with Ni/(*S*,*S*)-**5c** as the catalyst resulted in good yields with maintained e.e. We found that increasing the reaction times with Ni/(*S*,*S*)-**5c** as the catalyst resulted in good yields with maintained e.e. (Figure S3, entries 1-3, 95% e.e.).

Supplementary Table 4. Study on the results of 4a

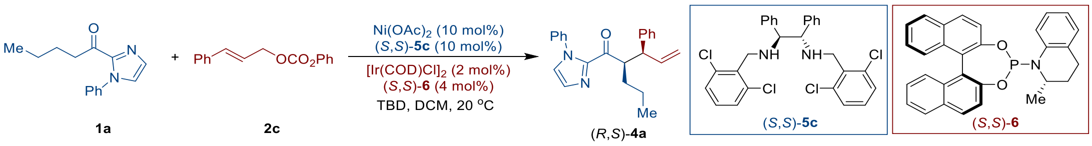

Entry	Reaction time	Yield (%)	d.r. of **4a**	e.e. of **4a** (%)
1	2 d	85	>20:1	>99
2	4 d	86	>20:1	>99
3	6 d	86	>20:1	>99

All the reactions were carried out by using **1a** (0.1 mmol), **2c** (0.15 mmol), Ni(OAc)$_2$ (10 mol%), (*S*,*S*)-**5c** (10 mol%), [Ir(COD)Cl]$_2$ (2 mol%), (*S*,*S*)-**6** (4 mol%), and TBD (10 mol%) in dichloromethane (DCM) (2.0 mL) at 20 °C.

Meanwhile, we explored the effect of varying reaction times on the reaction outcomes for the synergistic Ni/Ir-catalytic system. We found that increasing the reaction time had no effect on the outcome (Figure S4, entries 1-3). This finding shows that the reaction system has no effect on the α-stereocenters in the products.

Further study on the catalyst loadings

Supplementary Table 5. Survey of catalyst loading for the linear allylic alkylation

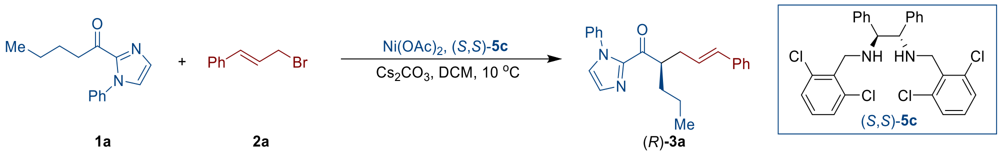

Entry	Loading of Ni(OAc)$_2$	Loading of (*S*,*S*)-**5c**	Yield of (*R*)-**3a** (%)	e.e. of (*R*)-**3a** (%)
1	10%	10%	80	95
2	5%	5%	74	95
3	2%	2%	17	80
4	1%	1%	trace	-

All the reactions were carried out by using **1a** (0.1 mmol), **2a** (0.15 mmol), Ni(OAc)$_2$ (X mol%), (*S*,*S*)-**5c** (X mol%), and Cs_2CO_3 (0.2 mmol) in dichloromethane (DCM) (2.0 mL) at 10 °C.

Supplementary Table 6. Survey of catalyst loading for the branched allylic alkylation

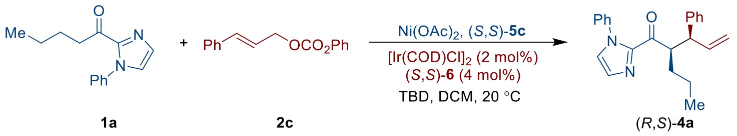

Entry	Loading of Ni(OAc)$_2$	Loading of (*S*,*S*)-**5c**	Yield (%)	d.r. of **4a**	e.e. of **4a** (%)
1	10%	10%	85	>20:1	>99
2	5%	5%	61	15:1	97
3	2%	2%	trace	—	—
4	1%	1%	trace	—	—

All the reactions were carried out by using **1a** (0.1 mmol), **2c** (0.15 mmol), Ni(OAc)$_2$ (X mol%), (*S*,*S*)-**5c** (X mol%), [Ir(COD)Cl]$_2$ (2 mol%), (*S*,*S*)-**6** (4 mol%), and TBD (10 mol%) in dichloromethane (DCM) (2.0 mL) at 20 °C.

Kinetic studies

Supplementary Table 7. Kinetic study for the linear allylic alkylation

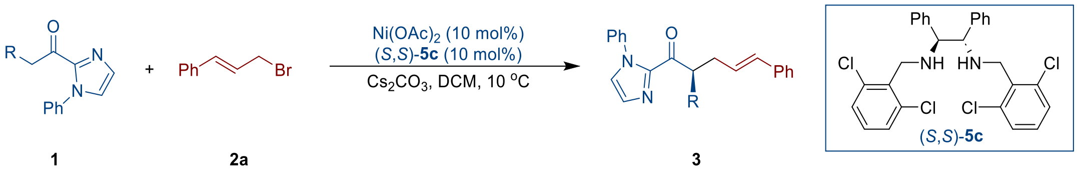

1 **2a** **3**

Entry	R = Ph[a]		R = ^{n}Pr	
	Reaction time	Yield of **3h** (%)	Reaction time	Yield of **3a** (%)
1	1 h	28	2 h	17
2	2 h	40	4 h	35
3	3 h	53	6 h	42
4	4 h	64	8 h	47
5	8 h	90	12 h	55

All the reactions were carried out by using **1** (0.1 mmol), **2** (0.15 mmol), $Ni(OAc)_2$ (10 mol%), (*S*,*S*)-**5c** (10 mol%), and Cs_2CO_3 (0.2 mmol) in dichloromethane (DCM) (2.0 mL) at 10 °C. [a]With Cs_2CO_3 (0.1 mmol) at 0 °C.

For highly reactive aryl-substituted acyl imidazole substrates, the desired products can be obtained at a rapid reaction rate (**3h**). However, the allylation reaction proceeds slower and requires a longer reaction time for alkyl-substituted acyl imidazole substrates which are significantly less reactive (**3a**) (Figure S1).

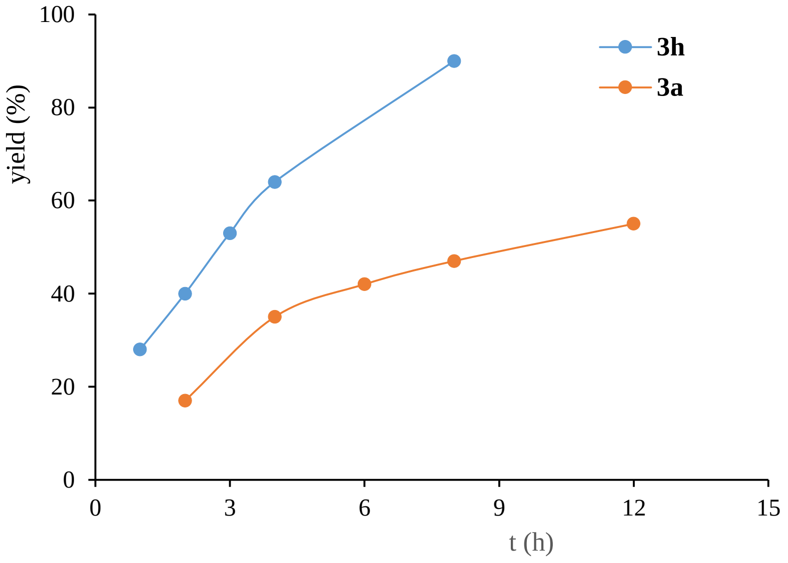

Supplementary Figure 1. Kinetic profile for the linear allylic alkylation

Supplementary Table 8. Kinetic study for the branched allylic alkylation

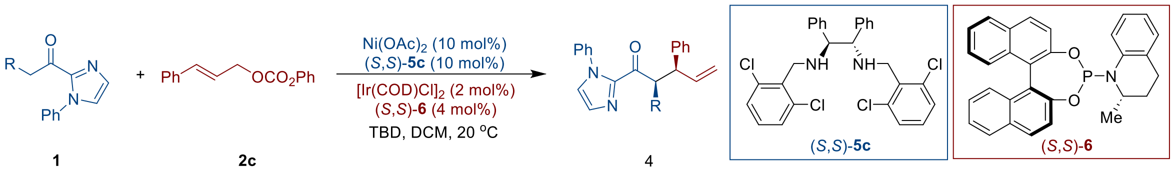

1 **2c** **4**

Entry	R = Ph[a]		R = ^{n}Pr	
	Reaction time	Yield of **4j** (%)	Reaction time	Yield of **4a** (%)
1	0.25 h	27	2 h	13
2	0.5 h	46	4 h	22
3	0.75 h	56	6 h	31
4	1 h	66	8 h	39
5	2 h	78	12 h	54
6	4 h	98		

All the reactions were carried out by using **1a** (0.1 mmol), **2c** (0.15 mmol), $Ni(OAc)_2$ (10 mol%), (*S*,*S*)-**5c** (10 mol%), $[Ir(COD)Cl]_2$ (2 mol%), (*S*,*S*)-**6** (4 mol%), and TBD (10 mol%) in dichloromethane (DCM) (2.0 mL) at 20 °C. [a]At 0 °C.

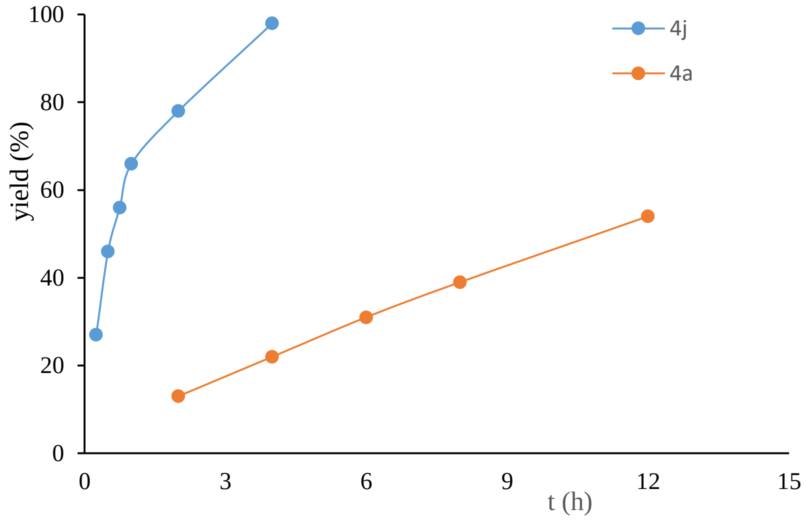

Supplementary Figure 2. Kinetic profile for the branched allylic alkylation

Synthetic application

Product derivation.

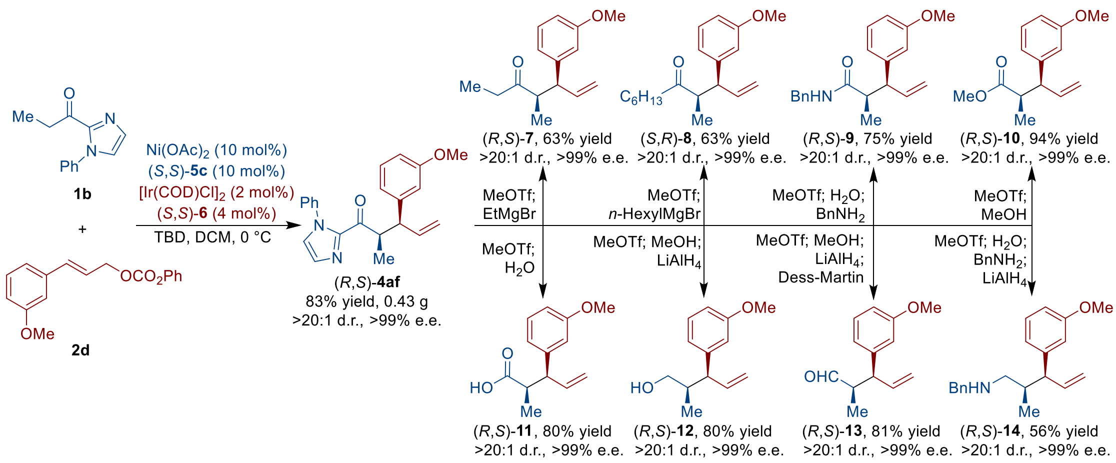

In a 10 mL Schlenk tube, $[Ir(COD)Cl]_2$ (0.03 mmol, 0.02 equiv.), (*S*,*S*)-**6** (0.06 mmol, 0.04 equiv.), TBD (0.15 mmol, 0.1 equiv.) were stirred in 3 mL DCM under argon at 25 ºC for 1.5 hours. **1b** (1.5 mmol), allylic carbonate **2d** (2.25 mmol, 1.5 equiv.), (*S*,*S*)-Ni catalyst (0.15 mmol, 0.1 equiv.), and additional DCM (3.5 mL) were then added successively and was added at 0 ℃. After the reaction was complete (monitored by TLC), the residue was purified by flash column chromatography on silica gel to afford the desired product (*R*,*S*)-**4af** (0.43 g, 83% yield, >20:1 d.r., >99% e.e.).

(2*R*,3*S*)-3-(3-methoxyphenyl)-2-methyl-1-(1-phenyl-1H-imidazol-2-yl)pent-4-en-1-one (4af)

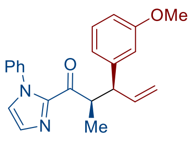

^{1}H NMR (400 MHz, $CDCl_3$) δ 7.51 – 7.42 (m, 3H), 7.31 (d, *J* = 1.0 Hz, 1H), 7.28 – 7.22 (m, 3H), 7.22 – 7.20 (m, 1H), 6.90 – 6.84 (m, 1H), 6.82 – 6.74 (m, 2H), 6.05 – 5.92 (m, 1H), 5.06 – 4.98 (m, 1H), 4.96 – 4.88 (m, 1H), 4.48 – 4.37 (m, 1H), 3.80 (s, 3H), 3.62 – 3.51 (m, 1H), 0.97 (d, *J* = 6.9 Hz, 3H). **^{13}C NMR (100 MHz, $CDCl_3$)** δ 194.69, 159.90, 143.66, 143.35, 140.44, 138.63, 129.76, 129.68, 129.08, 128.80, 127.45, 125.89, 120.83, 115.16, 114.15, 112.01, 55.33, 53.55, 45.34, 16.46. **ESI-MS:** calculated $[C_{22}H_{22}N_2O_2 + H]^+$: 347.1754, found: 347.1763. $[\alpha]^{20}_D$= -33.0 (c = 0.59, CH_2Cl_2). ^{1}H NMR spectroscopic analysis of the mixture indicated >20:1 d.r. The product was analyzed by HPLC to determine the enantiomeric excess: >99% e.e. (CHIRALPAK IBN-5, hexane/*i*-PrOH = 98/2, detector: 254 nm, T = 25 ℃, flow rate: 1.0 mL/min), t_1 (major) = 9.82 min, t_2 (minor) = 10.84 min.

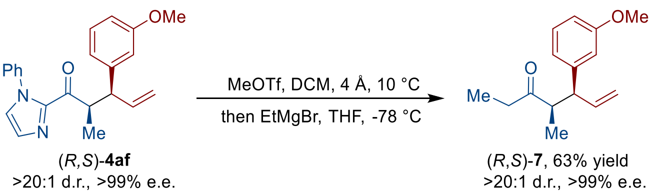

To a solution of **4af** (34.6 mg, 0.1 mmol, 1.0 equiv.) in dry DCM (2 mL) was added 4 Å molecular sieves (200 mg), and the resulting mixture was stirred vigorously for 2 hours at 10 ℃ under an argon atmosphere. Methyl trifluoromethanesulfonate (24.6 mg, 0.15 mmol, 1.5 equiv.) was then added and the mixture was stirred for 12 h. After complete consumption of the starting material **4af**, the solvent was

evaporated, and the residue was dissolved in dry THF (2 mL). Then, EtMgBr (0.1 mL, 0.2 mmol, 2.0 equiv., 2.0 M in THF) was added dropwise at -78 °C. After 30 min, the reaction was quenched with saturated aqueous $NaHCO_3$ at -78 °C. Then the reaction was stirred at 25 °C for 10 min. The reaction mixture was diluted with ethyl acetate. The organic layers were separated and the aqueous layer was extracted with ethyl acetate. The combined organic layers were dried over anhydrous $MgSO_4$, filtered, and concentrated under a vacuum. The residue was purified by silica gel chromatography to afford the desired product **7** (14.6 mg, 63% yield, >20:1 d.r., >99% e.e.).

(4*R*,5*S*)-5-(3-methoxyphenyl)-4-methylhept-6-en-3-one (7)

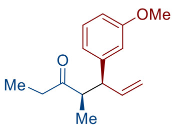

^{1}H NMR (400 MHz, $CDCl_3$) δ 7.23 (t, *J* = 7.9 Hz, 1H), 6.83 – 6.68 (m, 3H), 6.00 – 5.84 (m, 1H), 5.04 – 4.92 (m, 2H), 3.80 (s, 3H), 3.49 – 3.38 (m, 1H), 3.01 – 2.89 (m, 1H), 2.58 – 2.48 (m, 1H), 2.48 – 2.37 (m, 1H), 1.04 (t, *J* = 7.2 Hz, 3H), 0.87 (d, *J* = 6.9 Hz, 3H). **^{13}C NMR (100 MHz, $CDCl_3$)** ^{13}C NMR (100 MHz, $CDCl_3$) δ 214.68, 159.92, 143.47, 139.71, 129.77, 120.53, 115.86, 114.13, 111.79, 55.31, 53.41, 51.07, 36.25, 15.99, 7.57. **ESI-MS:** calculated $[C_{26}H_{22}N_2O + Na]^+$: 255.1356, found: 255.1366. $[\alpha]^{20}_D$= -67.8 (c = 0.26, CH_2Cl_2). ^{1}H NMR spectroscopic analysis of the mixture indicated >20:1 d.r. The product was analyzed by HPLC to determine the enantiomeric excess: >99% e.e. (CHIRALPAK IE, hexane/*i*-PrOH = 95/5, detector: 274 nm, T = 25 °C, flow rate: 1 mL/min), t_1 (minor) = 8.27 min, t_2 (major) = 11.66 min.

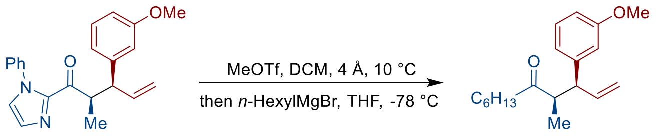

(*R*,*S*)-**4af**
>20:1 d.r., >99% e.e.

(*S*,*R*)-**8**, 63% yield
>20:1 d.r., >99% e.e.

To a solution of **4af** (34.1 mg, 0.1 mmol, 1.0 equiv.) in dry DCM (2 mL) was added 4 Å molecular sieves (200 mg), and the mixture was stirred vigorously for 2 hours at 10 °C under an argon atmosphere. Methyl trifluoromethanesulfonate (24.6 mg, 0.15 mmol, 1.5 equiv.) was then added and the mixture was stirred for 12 hours. After complete consumption of the starting material **4af**, the solvent was evaporated, and the residue was dissolved in dry THF (2 mL). Then, *n*-HexylMgBr (0.2 mL, 0.2 mmol, 2.0 equiv., 1.0 M in THF) was dropwise at -78 °C. After 30 min, the reaction was quenched with saturated aqueous $NaHCO_3$ at -78 °C. Then the reaction was stirred at 25 °C for 10 min. The reaction mixture was diluted with ethyl acetate. The organic layers were separated and the aqueous layer was extracted with ethyl acetate. The combined organic layers were dried over anhydrous $MgSO_4$, filtered, and concentrated under a vacuum. The residue was purified by silica gel chromatography to afford the desired product **8** (18.2 mg, 63% yield, >20:1 d.r., >99% e.e.).

(3*S*,4*R*)-3-(3-methoxyphenyl)-4-methylundec-1-en-5-one (8)

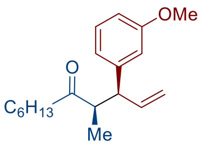

^{1}H NMR (400 MHz, $CDCl_3$) δ 7.23 (t, *J* = 7.9 Hz, 1H), 6.81 – 6.70 (m, 3H), 5.98 – 5.87 (m, 1H), 5.04 – 4.96 (m, 2H), 3.80 (s, 3H), 3.49 – 3.40 (m, 1H), 2.98 – 2.89 (m, 1H), 2.54 – 2.36 (m, 2H), 1.60 – 1.51 (m, 2H), 1.31 – 1.25 (m, 6H), 0.91 – 0.84 (m, 6H). **^{13}C NMR (100 MHz, $CDCl_3$)** δ 214.27, 159.89, 143.49, 139.70, 129.76, 120.53, 115.91, 114.13, 111.75, 55.30, 53.27, 51.14, 43.13, 31.78, 29.03, 23.35, 22.65, 15.95, 14.20. **ESI-MS:** calculated $[C_{26}H_{22}N_2O + H]^+$: 289.2162, found: 289.2165. $[\alpha]^{20}_D$= -64.3 (c = 0.37, CH_2Cl_2). ^{1}H NMR spectroscopic analysis of the mixture indicated >20:1 d.r. The product was analyzed by HPLC to determine the enantiomeric excess: >99% e.e. (CHIRALPAK IE, hexane/*i*-PrOH = 99/1, detector: 273 nm, T = 25 °C, flow rate: 0.5 mL/min), t_1 (minor) = 21.80 min, t_2 (major) = 24.40 min.

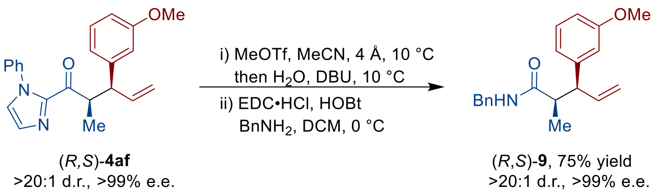

To a solution of **4af** (129 mg, 0.37 mmol, 1.0 equiv.) in dry MeCN (5 mL) was added 4 Å molecular sieves (500 mg), and the mixture was stirred vigorously for 2 hours at 10 °C under an argon atmosphere. Methyl trifluoromethanesulfonate (91.8 mg, 0.56 mmol, 1.5 equiv.) was then added and the mixture was stirred for 12 hours. After complete consumption of the starting material **4af**, water (1.0 mL) and 1,8-diazabicyclo[5.4.0]undec-7-ene (85.1 mg, 0.56 mmol, 1.5 equiv.) were added stepwise. The mixture was stirred at 10 °C under an argon atmosphere. After 2 hours the reaction was diluted with ethyl acetate and 1N HCl. The organic layers were separated and the aqueous layer was extracted with ethyl acetate. The combined organic layers were dried over anhydrous $MgSO_4$, filtered, and concentrated under a vacuum. The residue was purified by flash silica gel to afford the desired product **11** (65.1 mg, 80% yield, >20:1 d.r., >99% e.e.).

To a solution of **11** (41.36 mg, 0.19 mmol, 1.0 equiv.) in DCM (5 mL) at 0 °C was added benzylamine (0.23 mmol, 1.2 equiv.), EDC•HCl (0.23 mmol, 1.2 equiv.), and HOBt (0.23mmol, 1.2 equiv.) under argon atmosphere. The resulting solution was stirred for 4 hours at 0 °C. The reaction mixture was diluted with DCM and washed twice with water and once with brine. The organic layer was dried over anhydrous $MgSO_4$, filtered, and concentrated under a vacuum. The crude product was purified by flash chromatography to afford the desired product **9** (54.9 mg, 94% yield, >20:1 d.r., >99% e.e.).

(2*R*,3*S*)-N-benzyl-3-(3-methoxyphenyl)-2-methylpent-4-enamide (9)

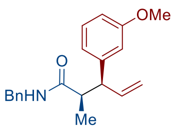

^{1}H NMR (500 MHz, CDCl$_3$) δ 7.36 – 7.18 (m, 6H), 6.79 – 6.70 (m, 3H), 6.05 – 5.95 (m, 1H), 5.88 (t, *J* = 5.1 Hz, 1H), 5.11 – 4.99 (m, 2H), 4.54 – 4.43 (m, 1H), 4.41 – 4.32 (m, 1H), 3.77 (s, 3H), 2.58 – 2.48 (m, 1H), 1.00 (d, *J* = 6.8 Hz, 3H). **^{13}C NMR (125 MHz, CDCl$_3$)** δ 174.94, 159.85, 143.49, 139.38, 138.36, 129.73, 128.73, 128.02, 127.57, 120.49, 116.22, 114.16, 111.75, 55.24, 53.71, 47.11, 43.58, 16.70. **ESI-MS:** calculated $[C_{26}H_{22}N_2O + H]^+$: 310.1802, found: 310.1812. $[\alpha]^{20}_D$= -57.1 (c = 0.35, CH_2Cl_2). ^{1}H NMR spectroscopic analysis of the mixture indicated >20:1 d.r. The product was analyzed by HPLC to determine the enantiomeric excess: >99% e.e. (CHIRALPAK IC, hexane/*i*-PrOH = 90/10, detector: 273 nm, T = 25 °C, flow rate: 1 mL/min), t_1 (minor) = 16.66 min, t_2 (major) = 18.16 min.

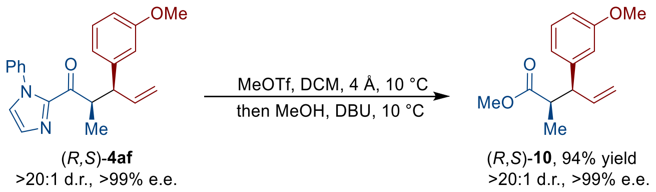

To a solution of **4af** (50 mg, 0.14 mmol, 1.0 equiv.) in dry DCM (2 mL) was added 4 Å molecular sieves (200 mg), and the mixture was stirred vigorously for 2 hours at 10 °C under an argon atmosphere. Methyl trifluoromethanesulfonate (34.4 mg, 0.21 mmol, 1.5 equiv.) was then added and the mixture was stirred for 12 hours. After complete consumption of the starting material **4af**, MeOH (0.5 mL) and 1,8-diazabicyclo[5.4.0]undec-7-ene (31.9 mg, 0.21 mmol, 1.5 equiv.) were added stepwise. The mixture was

stirred at 10 °C under an argon atmosphere. After 2 hours the solvent was evaporated and the residue was purified by column chromatography to give **10** (30.8 mg, 94% yield, >20:1 d.r., >99% e.e.).

methyl (2*R*,3*S*)-3-(3-methoxyphenyl)-2-methylpent-4-enoate (10)

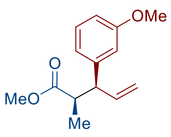

^{1}H NMR (500 MHz, CDCl$_3$) δ 7.24 (t, *J* = 7.9 Hz, 1H), 6.80 – 6.75 (m, 2H), 6.74 – 6.71 (m, 1H), 6.05 – 5.92 (m, 1H), 5.11 – 4.98 (m, 2H), 3.80 (s, 3H), 3.68 (s, 3H), 3.44 – 3.36 (m, 1H), 2.88 – 2.78 (m, 1H), 0.98 (d, *J* = 6.9 Hz, 3H). **^{13}C NMR (100 MHz, CDCl$_3$)** δ 176.26, 159.90, 143.02, 139.70, 129.80, 120.49, 115.64, 114.06, 111.93, 55.30, 53.96, 51.67, 45.33, 16.06. **ESI-MS:** calculated [$C_{26}H_{22}N_2O$ + Na]$^+$**:** 257.1148, found: 257.1156. $[\alpha]^{20}_{D}$= -52.8 (c = 0.31, CH_2Cl_2). ^{1}H NMR spectroscopic analysis of the mixture indicated >20:1 d.r. The product was analyzed by HPLC to determine the enantiomeric excess: >99% e.e. (CHIRALPAK IE, hexane/*i*-PrOH = 95/5, detector: 274 nm, T = 25 °C, flow rate: 1 mL/min), t_1 (major) = 8.53 min, t_2 (minor) = 14.28 min.

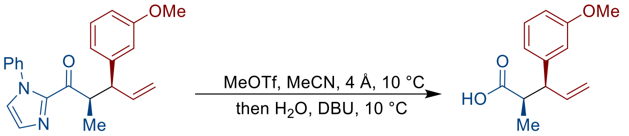

(*R*,*S*)-**4af**
>20:1 d.r., >99% e.e.

(*R*,*S*)-**11**, 80% yield
>20:1 d.r., >99% e.e.

To a solution of **4af** (129 mg, 0.37 mmol, 1.0 equiv.) in dry MeCN (5 mL) was added 4 Å molecular sieves (500 mg), and the mixture was stirred vigorously for 2 hours at 10 °C under an argon atmosphere. Methyl trifluoromethanesulfonate (91.8 mg, 0.56 mmol, 1.5 equiv.) was then added and the mixture was stirred for 12 hours. After complete consumption of the starting material **4af**, water (1.0 mL) and 1,8-diazabicyclo[5.4.0]undec-7-ene (85.1 mg, 0.56 mmol, 1.5 equiv.) were added stepwise. The mixture was stirred at 10 °C under an argon atmosphere. After 2 hours the reaction was diluted with ethyl acetate and 1N HCl. The organic layers were separated and the aqueous layer was extracted with ethyl acetate. The combined organic layers were dried over anhydrous $MgSO_4$, filtered, and concentrated under a vacuum. The residue was purified by flash silica gel to afford the desired product **11** (65.1 mg, 80% yield, >20:1 d.r., >99% e.e.).

(2*R*,3*S*)-3-(3-methoxyphenyl)-2-methylpent-4-enoic acid (11)

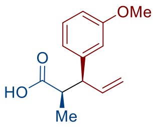

^{1}H NMR (400 MHz, CDCl$_3$) δ 7.31 – 7.19 (m, 1H), 6.83 – 6.72 (m, 3H), 6.10 – 5.95 (m, 1H), 5.16 – 5.02 (m, 2H), 3.80 (s, 3H), 3.49 – 3.38 (m, 1H), 2.89 – 2.77 (m, 1H), 1.03 (d, *J* = 6.9 Hz, 3H). **^{13}C NMR (101 MHz, CDCl$_3$)** δ 182.20, 159.92, 142.66, 139.31, 129.84, 120.56, 116.04, 114.11, 112.05, 55.30, 53.48, 45.19, 16.02. **ESI-MS:** calculated [$C_{26}H_{22}N_2O$ + H]$^+$: 221.1172, found: 221.1166. $[\alpha]^{20}_{D}$= -26.8 (c = 0.90, CH_2Cl_2). ^{1}H NMR spectroscopic analysis of the mixture indicated >20:1 d.r. The product was analyzed by HPLC to determine the enantiomeric excess: >99% e.e. (CHIRALPAK IG, hexane/*i*-PrOH/AcOH = 989/10/1, detector: 270 nm, T = 25 °C, flow rate: 1 mL/min), t_1 (minor) = 29.86 min, t_2 (major) = 32.03 min.

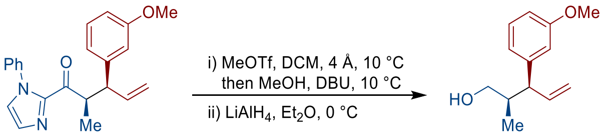

(*R*,*S*)-**4af**
>20:1 d.r., >99% e.e.

(*R*,*S*)-**12**, 80% yield
>20:1 d.r., >99% e.e.

To a solution of **4af** (50 mg, 0.14 mmol, 1.0 equiv.) in dry DCM (2 mL) was added 4 Å molecular sieves (200 mg), and the mixture was stirred vigorously for 2 hours at 10 °C under an argon atmosphere. Methyl trifluoromethanesulfonate (34.4 mg, 0.21 mmol, 1.5 equiv.) was then added and the mixture was stirred for 12 hours. After complete consumption of the starting material **4af**, MeOH (0.5 mL) and 1,8-diazabicyclo[5.4.0]undec-7-ene (31.9 mg, 0.21 mmol, 1.5 equiv.) were added stepwise. The mixture was stirred at 10 °C under an argon atmosphere. After 2 hours the solvent was evaporated and the residue was purified by column chromatography to give **10** (30.8 mg, 94% yield, >20:1 d.r., >99% e.e.).

To a solution of **10** (23.4 mg, 0.1 mmol, 1.0equiv.) in Et_2O (2 mL) at 0 °C was added $LiAlH_4$ (7.6 mg, 0.2 mmol, 2.0 equiv.). The mixture was allowed to warm to 25 °C and stirred for 2 hours until complete consumption of **10** (monitored by TLC). The reaction was quenched with water, and diluted with EtOAc. The aqueous phase was separated and extracted twice with EtOAc. The combined organic phase was dried over anhydrous $MgSO_4$ and concentrated under a vacuum. The residue was purified by column chromatography to give **12** (17.6 mg, 85% yield, >20:1 d.r., >99% e.e.).

(2*R*,3*S*)-3-(3-methoxyphenyl)-2-methylpent-4-en-1-ol (12)

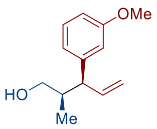

^{1}H NMR (400 MHz, $CDCl_3$) δ 7.22 (t, J = 7.8 Hz, 1H), 6.83 – 6.72 (m, 3H), 6.10 – 5.98 (m, 1H), 5.16 – 5.08 (m, 1H), 5.07 – 5.02 (m, 1H), 3.80 (s, 3H), 3.73 – 3.64 (m, 1H), 3.60 – 3.53 (m, 1H), 3.13 (t, J = 9.3 Hz, 1H), 2.09 – 1.98 (m, 1H), 1.53 (s, 1H), 0.81 (d, J = 6.8 Hz, 3H). **^{13}C NMR (100 MHz, $CDCl_3$)** δ 159.80, 144.89, 141.26, 129.63, 120.43, 115.35, 114.06, 111.36, 66.63, 55.27, 54.32, 40.20, 15.31. **ESI-MS:** calculated $[C_{26}H_{22}N_2O + H]^+$: 207.1380, found: 207.1376. $[\alpha]^{20}_D$= -90.1 (c = 0.55, CH_2Cl_2). ^{1}H NMR spectroscopic analysis of the mixture indicated >20:1 d.r. The product was analyzed by HPLC to determine the enantiomeric excess: >99% e.e. (CHIRALPAK IC, hexane/*i*-PrOH = 98/2, detector: 270 nm, T = 25 °C, flow rate: 1 mL/min), t_1 (major) = 14.58 min, t_2 (minor) = 16.51 min.

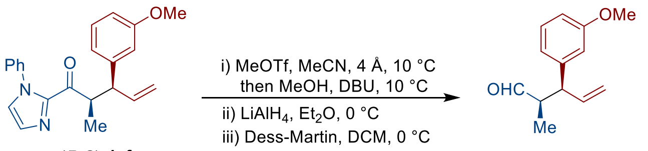

(*R*,*S*)-**4af**
>20:1 d.r., >99% e.e.

(*R*,*S*)-**13**, 81% yield
>20:1 d.r., >99% e.e.

To a solution of **4af** (51.9 mg, 0.15 mmol, 1.0equiv.) in dry DCM (3 mL) was added 4 Å molecular sieves (200 mg), and the mixture was stirred vigorously for 2 hours at 10 °C under an argon atmosphere. Methyl trifluoromethanesulfonate (37.7 mg, 0.23 mmol, 1.5 equiv.) was then added and the mixture was stirred for 12 hours. After complete consumption of the starting material **4af**, MeOH (0.5 mL) and 1,8-diazabicyclo[5.4.0]undec-7-ene (34.9 mg, 0.23 mmol, 1.5 equiv.) were added stepwise. The mixture was stirred at 10 °C under an argon atmosphere. After 2 hours the solvent was evaporated and the residue was purified by column chromatography to give **10**. To a solution of **10** in Et_2O (2 mL) at 0 °C was added $LiAlH_4$ (11.4 mg, 0.3 mmol, 2.0 equiv.). The mixture was allowed to warm to 25 °C and stirred for 2 hours until complete consumption of **10** (monitored by TLC). The reaction was quenched with water, and diluted with EtOAc. The aqueous phase was separated and extracted twice with EtOAc. The combined organic phase was dried over anhydrous $MgSO_4$ and concentrated under a vacuum. The residue was purified by column chromatography to give **12**. To a solution of **12** in DCM (2 mL) at 0 °C was added Dess-Martin periodinane (76.3mg, 0.18 mmol, 1.2 equiv.). The mixture was allowed to warm to 25 °C and stirred for overnight until complete consumption of **10** (monitored by TLC). The combined

organic phase was concentrated under a vacuum. The residue was purified by column chromatography to give **13** (24.7 mg, 81% yield, >20:1 d.r., >99% e.e.).

(2*R*,3*S*)-3-(3-methoxyphenyl)-2-methylpent-4-enal (13)

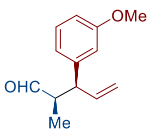

^{1}H NMR (400 MHz, $CDCl_3$) δ 9.68 (d, *J* = 3.1 Hz, 1H), 7.25 (t, *J* = 7.9 Hz, 1H), 6.80 – 6.72 (m, 3H), 6.06 – 5.96 (m, 1H), 5.14 – 5.08 (m, 2H), 3.80 (s, 3H), 3.49 (t, *J* = 8.9 Hz, 1H), 2.81 – 2.73 (m, 1H), 0.93 (d, *J* = 7.0 Hz, 3H). **^{13}C NMR (101 MHz, $CDCl_3$)** δ 204.66, 159.96, 142.64, 139.02, 129.88, 120.52, 116.57, 114.24, 111.94, 55.32, 51.62, 50.51, 12.71. **ESI-MS:** calculated $[C_{26}H_{22}N_2O + Na]^+$: 227.1043, found: 227.1049. $[\alpha]^{20}_D$= -37.8 (c = 0.63, CH_2Cl_2). ^{1}H NMR spectroscopic analysis of the mixture indicated >20:1 d.r. The product was analyzed by HPLC to determine the enantiomeric excess: >99% e.e. (CHIRALPAK OD-H, hexane/*i*-PrOH = 98/2, detector: 270 nm, T = 25 °C, flow rate: 1 mL/min), t_1 (major) = 8.11 min, t_2 (minor) = 8.88 min.

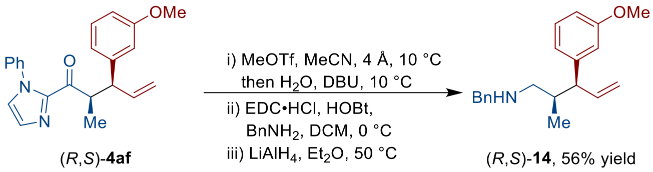

(*R*,*S*)-**4af** >20:1 d.r., >99% e.e.; (*R*,*S*)-**14**, 56% yield >20:1 d.r., >99% e.e.

To a solution of **4af** (129 mg, 0.37 mmol, 1.0 equiv.) in dry MeCN (5 mL) was added 4 Å molecular sieves (500 mg), and the mixture was stirred vigorously for 2 hours at 10 °C under an argon atmosphere. Methyl trifluoromethanesulfonate (91.8 mg, 0.56 mmol, 1.5 equiv.) was then added and the mixture was stirred for 12 hours. After complete consumption of the starting material **4af**, water (1.0 mL) and 1,8-diazabicyclo[5.4.0]undec-7-ene (85.1 mg, 0.56 mmol, 1.5 equiv.) were added stepwise. The mixture was stirred at 10 °C under an argon atmosphere. After 2 hours the reaction was diluted with ethyl acetate and 1N HCl. The organic layers were separated and the aqueous layer was extracted with ethyl acetate. The combined organic layers were dried over anhydrous $MgSO_4$, filtered, and concentrated under a vacuum. The residue was purified by flash silica gel to afford the desired product **11**(65.1 mg, 80% yield, >20:1 d.r., >99% e.e.).

To a solution of **11** (41.4 mg, 0.19 mmol, 1.0 equiv.) in DCM (5 mL) at 0 °C was added benzylamine (0.23 mmol, 1.2 equiv.), EDC•HCl (0.23 mmol, 1.2 equiv.) and HOBt (0.23 mmol, 1.2 equiv.) under argon atmosphere. The resulting solution was stirred for 4 hours at 0 °C. The reaction mixture was diluted with DCM and washed twice with water and once with brine. The organic layer was dried over anhydrous $MgSO_4$, filtered, and concentrated under a vacuum. The crude product was purified by flash chromatography to afford the desired product **9** (54.9 mg, 94% yield, >20:1 d.r., >99% e.e.).

To a solution of **9** (30.9 mg, 0.1 mmol, 1.0 equiv.) in Et_2O (2 mL) at 25 °C was added $LiAlH_4$ (30.4 mg, 0.8 mmol, 8.0 equiv.). The mixture was allowed to heat to 50 °C and stirred for overnight until complete consumption of **9** (monitored by TLC). The reaction was quenched with water, and diluted with EtOAc. The aqueous phase was separated and extracted twice with EtOAc. The combined organic phase was dried over anhydrous $MgSO_4$ and concentrated under a vacuum. The residue was purified by column chromatography to give **14** (22.1 mg, 75% yield, >20:1 d.r., >99% e.e.).

(2*R*,3*S*)-N-benzyl-3-(3-methoxyphenyl)-2-methylpent-4-en-1-amine (14)

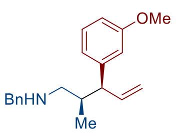

^{1}H NMR (400 MHz, $CDCl_3$) δ 7.34 – 7.30 (m, 4H), 7.26 – 7.25 (m, 1H), 7.20 (t, *J* = 7.8 Hz, 1H), 6.78 – 6.71 (m, 3H), 6.05 – 5.94 (m, 1H), 5.10 – 4.98 (m, 2H), 3.80 – 3.74 (m, 5H), 3.10 (t, *J* = 9.0 Hz, 1H), 2.81 – 2.73 (m, 1H), 2.50 – 2.42 (m, 1H), 2.09 – 1.98 (m, 1H), 1.53 (s, 1H), 0.80 (d, *J* = 6.7 Hz, 3H). **^{13}C NMR (101 MHz, $CDCl_3$)** δ 159.76, 145.28, 141.08, 140.77, 129.50, 128.49, 128.24, 127.00, 120.59, 115.28, 114.09, 111.33, 55.31, 55.27, 54.43, 53.85, 37.99, 16.66. **ESI-MS:** calculated $[C_{26}H_{22}N_2O + H]^+$: 296.2009, found: 296.2016. $[\alpha]^{20}_D$= -49.7 (c = 0.23, CH_2Cl_2). ^{1}H NMR spectroscopic analysis of the mixture indicated >20:1 d.r. The product was analyzed by HPLC to determine the enantiomeric excess: >99% e.e. (CHIRALPAK AD-H, hexane/*i*-PrOH/DEA = 989/10/1, detector: 272 nm, T = 25 °C, flow rate: 0.5 mL/min), t_1 (minor) = 16.88 min, t_2 (major) = 22.37 min.

Enantioselective synthesis of (*R*)-arundic acid

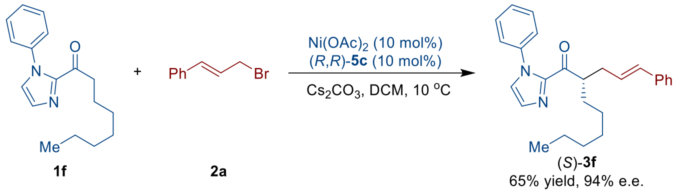

A 10 mL Schlenk tube was charged with **1f** (0.1 mmol, 1.0 equiv.), **2a** (0.15 mmol, 1.5 equiv.), Ni catalyst (0.01 mmol, 0.1 equiv.), Cs_2CO_3 (0.2 mmol, 2.0 equiv.) and DCM (2 mL) under argon atmosphere. The reaction mixture was stirred at 10 °C until complete consumption of the substrate (monitored by TLC). The solution was diluted with dichloromethane and then filtered with celite. The residue was purified by flash column chromatography on silica gel to afford the desired product **3f** (25.1 mg, 65% yield, 94% e.e.).

(*S*)-2-cinnamyl-1-(1-phenyl-1H-imidazol-2-yl)octan-1-one (3f)

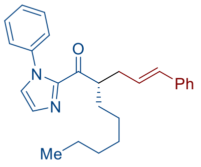

^{1}H NMR (400 MHz, $CDCl_3$) δ 7.45 – 7.36 (m, 3H), 7.28 – 7.24 (m, 5H), 7.21 – 7.12 (m, 4H), 6.35 (d, *J* = 15.8 Hz, 1H), 6.24 – 6.11 (m, 1H), 4.13 – 4.00 (m, 1H), 2.64 – 2.53 (m, 1H), 2.51 – 2.40 (m, 1H), 1.84 – 1.70 (m, 1H), 1.61 – 1.49 (m, 1H), 1.34 – 1.18 (m, 8H), 0.84 (t, *J* = 6.8 Hz, 3H). **^{13}C NMR (100 MHz, $CDCl_3$)** δ 194.81, 143.61, 138.68, 137.69, 131.74, 129.68, 129.05, 128.73, 128.55, 128.09, 127.22, 127.10, 126.20, 125.98, 46.93, 35.85, 31.83, 31.78, 29.59, 27.48, 22.71, 14.18. $[\alpha]^{20}_D$= 38.3 (c = 0.49, CH_2Cl_2). The product was analyzed by HPLC to determine the enantiomeric excess: 94% e.e. (CHIRALPAK IC, hexane/*i*-PrOH = 95/5, detector: 254 nm, T = 25 °C, flow rate: 1 mL/min), t_1 (major) = 6.52 min, t_2 (minor) = 7.12 min.

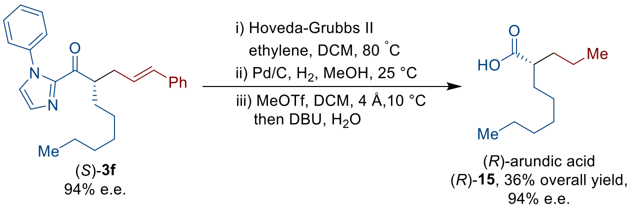

3f (0.1 mmol, 1.0 equiv.) was dissolved in 3 mL DCM and transferred to a Schlenk tube. Ethylene gas was bubbled through the reaction for approximately 7 min before the introduction of HG2 (0.02 mmol, 0.2 equiv.) in DCM (0.5 mL). The reaction mixture was then taken to a pre-heated oil bath at 80 °C

to initiate the ethenolysis reactions. After 2 hours, **3f** was completely converted to the desired ethenolysis products. The solvent was removed under reduced pressure to reveal a black oil which was purified by flash column chromatography to afford the intermediate products (40% yield, 94% e.e.). The intermediate products (32 mg, 0.103 mmol, 1.0 equiv.) was dissolved in 2 mL methanol and Pd/C (10 wt%, 3 mg) was added. The mixture was stirred under hydrogen atmosphere for 12 hours at room temperature, then filtered and concentrated in vacuo. The residue and 4 Å molecular sieves (200 mg) were stirred vigorously in DCM (2 mL) for 2 hours at 25 °C under argon atmosphere. Then methyl trifluoromethanesulfonate (0.15 mmol, 1.5 equiv.) was added, and the reaction mixture was stirred at 25 °C for 12 hours. The reaction mixture was filtered through celite and concentrated under vacuum. The residue was dissolved in acetonitrile (2.0 mL), then water (0.8 mL) and 1,8-diazabicyclo[5.4.0]undec-7-ene (1.5 equiv.) were added stepwise at 25 °C. After 30 min, the reaction was diluted with ethyl acetate and 1N HCl. The organic layers were separated and the aqueous layer was extracted with ethyl acetate. The combined organic layers were dried over $MgSO_4$, filtered, and concentrated under vacuum. The residue was purified by silica gel chromatography to afford the desired product **15** (17.3 mg, 90% yield, 94% e.e.).

(*R*)-arundic acid (15)

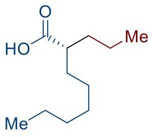

^{1}H NMR (400 MHz, $CDCl_3$) δ 2.41 – 2.33 (m, 1H), 1.68 – 1.56 (m, 2H), 1.52 – 1.41 (m, 2H), 1.39 – 1.24 (m, 10H), 0.96 – 0.83 (m, 6H). **^{13}C NMR (100 MHz, $CDCl_3$)** δ 182.35, 45.38, 34.50, 32.36, 31.81, 29.36, 27.47, 22.75, 20.71, 14.21, 14.15. **ESI-MS:** calculated $[C_{11}H_{22}O_2 + H]^+$: 187.1693, found: 187.1698. $[\alpha]^{20}_{D}$= -18.1 (c = 0.24, CH_2Cl_2). The e.e. value of **15** was determined after conversion into the corresponding phenacyl ester as below. To a suspension of **15** (0.05 mmol) and K_2CO_3 (1.1 equiv.) in 1 mL acetone was added phenacylbromide (1.1 equiv.). The mixture was stirred at room temperature for 1 hour, then it was filtered and the residue was purified by flash column chromatography on silica gel to afford the desired product. The product was analyzed by HPLC to determine the enantiomeric excess: 94% e.e. (CHIRALPAK OJ-H, hexane/*i*-PrOH = 99/1, detector: 250 nm, T = 25 °C, flow rate: 1 mL/min), t_1 (minor) = 11.27 min, t_2 (major) = 12.29 min.

Enantioselective synthesis of cinamomumolide

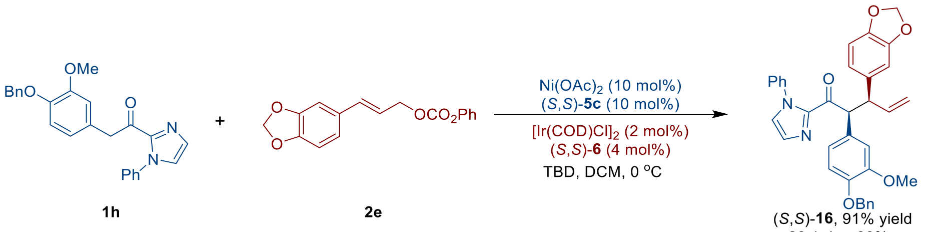

In a 10 mL Schlenk tube, $[Ir(COD)Cl]_2$ (0.002 mmol, 0.02 equiv.), (*S*,*S*)-**6** (0.004 mmol, 0.04 equiv.), TBD (0.01 mmol, 0.1 equiv.) were stirred in 1 mL DCM under argon at 25 °C for 10 min. **1h** (0.1 mmol, 1.0 equiv.), allylic carbonate **2e** (0.15 mmol, 1.5equiv.) and (*S*,*S*)-Ni catalyst (0.01 mmol, 0.1equiv.) were then added successively and additional DCM (1 mL) was added. The mixture was stirred at 0 °C for 4 hours. After the reaction was complete (monitored by TLC), the residue was purified by flash column chromatography on silica gel to afford the desired product **16** (91% yield, >20:1 d.r., 99% e.e.).

(2*S*,3*S*)-3-(benzo[d][1,3]dioxol-5-yl)-2-(4-(benzyloxy)-3-methoxyphenyl)-1-(1-phenyl-1H-imidazol-2-yl)pent-4-en-1-one (16)

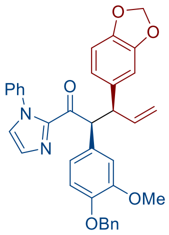

^{1}H NMR (400 MHz, CDCl$_3$) δ 7.44 – 7.25 (m, 9H), 7.17 – 7.07 (m, 3H), 6.81 – 6.70 (m, 2H), 6.68 – 6.59 (m, 2H), 6.58 – 6.48 (m, 2H), 6.09 – 5.96 (m, 1H), 5.84 – 5.78 (m, 2H), 5.51 (d, J = 11.8 Hz, 1H), 5.15 (d, J = 17.2 Hz, 1H), 5.06 – 4.97 (m, 3H), 4.13 – 4.04 (m, 1H), 3.74 (s, 3H). **^{13}C NMR (100 MHz, CDCl$_3$)** δ 190.07, 149.29, 147.46, 147.08, 145.85, 143.39, 140.21, 138.36, 137.21, 135.16, 129.82, 129.61, 129.01, 128.74, 128.54, 127.84, 127.39, 127.33, 125.63, 121.93, 121.78, 115.50, 113.61, 112.97, 108.76, 108.05, 100.81, 70.90, 56.85, 56.03, 52.12. **ESI-MS:** calculated $[C_{35}H_{30}N_2O_5 + H]^+$: 559.2227, found: 559.2228. $[\alpha]^{20}_D$= 112.6 (c = 2.52, CH_2Cl_2). ^{1}H NMR spectroscopic analysis of the mixture indicated >20:1 d.r. The product was analyzed by HPLC to determine the enantiomeric excess: 99% e.e. (CHIRALPAK AD-H, hexane/*i*-PrOH = 70/30, detector: 282 nm, T = 25 °C, flow rate: 1.0 mL/min), t_1 (minor) = 5.76 min, t_2 (major) = 6.60 min.

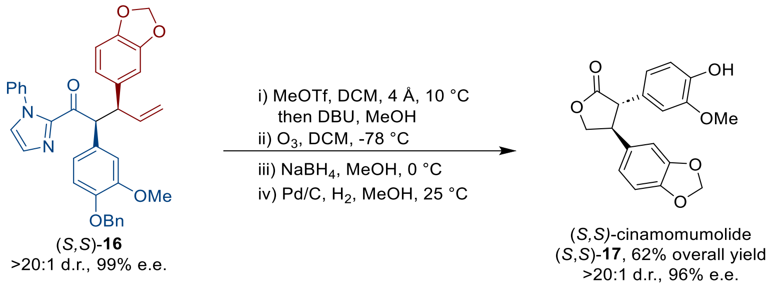

(*S*,*S*)-**16**
>20:1 d.r., 99% e.e.

(*S*,*S*)-cinamomumolide
(*S*,*S*)-**17**, 62% overall yield
>20:1 d.r., 96% e.e.

To a solution of **16** (111.6 mg, 0.2mmol, 1.0 equiv.) and 4 Å molecular sieves (400 mg) were stirred vigorously in DCM (4.0 mL) for 2 hours at 25 °C under argon atmosphere. Then methyl trifluoromethanesulfonate (0.3 mmol, 1.5 equiv.) was added, and the reaction mixture was stirred at 25 °C for 12 hours. After complete consumption of the starting material **16**, MeOH (1.0 mL) and 1,8-diazabicyclo[5.4.0]undec-7-ene (0.3 mmol, 1.5 equiv.) were added stepwise. After 30 min, the reaction was diluted with ethyl acetate. The organic layers were separated and the aqueous layer was extracted with ethyl acetate. The combined organic layers were dried over $MgSO_4$, filtered, and concentrated under vacuum. The residue was purified by silica gel chromatography to afford the intermediate product (84.7mg, 95% yield).

Intermediate product (45 mg, 0.1 mmol, 1.0 equiv.) was dissolved in DCM (3.0 mL) and cooled to -78 °C. Then, O_3 was bubbled through this solution for 1 min (TLC control). Oxygen gas was passed through the solution for 3 min. Dimethyl sulfide (1.0 mmol, 10.0 equiv.) was then added and the reaction mixture was allowed to warm to 25 °C and maintained for 1 hour. The reaction mixture was concentrated and dissolved in methanol (2 mL). The resulted mixture was cooled to 0 °C and $NaBH_4$ (11.4 mg, 0.3 mmol, 3.0 equiv.) was added. After stirring at room temperature for 10 min, 5 mL H_2O was added and the solvent was removed. The aqueous phase was extracted by EtOAc (5 mL x 3). The combined organic phase was dried over Na_2SO_4 and concentrated under reduced pressure. The residue was purified by silica gel chromatography to afford the cyclizing product (31.6 mg). The product was dissolved in 2 mL methanol and Pd/C (10 wt%, 3.0 mg) was added. The mixture was stirred under hydrogen atmosphere for 12 hours at room temperature, then filtered and concentrated in vacuo. The residue was purified with preparative TLC to furnish the cinnamomumolide **17** (21.3 mg, 65% yield, >20:1 d.r., 96% e.e.).

(3*S*,4*S*)-4-(benzo[d][1,3]dioxol-5-yl)-3-(4-hydroxy-3-methoxyphenyl)dihydrofuran-2(3H)-one (17)

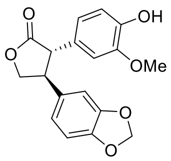

^{1}H NMR (400 MHz, CDCl$_3$) δ 6.84 (d, *J* = 8.1 Hz, 1H), 6.75 (d, *J* = 8.0 Hz, 1H), 6.72 – 6.60 (m, 4H), 5.96 (d, *J* = 1.7 Hz, 2H), 5.59 (s, 1H), 4.71 – 4.62 (m, 1H), 4.25 (t, *J* = 9.5 Hz, 1H), 3.85 (s, 3H), 3.81 – 3.68 (m, 2H). **^{13}C NMR (100 MHz, CDCl$_3$)** δ 176.55, 148.38, 147.40, 146.89, 145.46, 130.84, 126.92, 121.48, 120.90, 114.78, 110.79, 108.85, 107.37, 101.43, 71.79, 56.08, 53.14, 50.48. **ESI-MS:** calculated [$C_{18}H_{16}O_6$ +Na]$^+$: 351.0839, found: 351.0847. $[\alpha]^{20}_D$= 89.3 (c = 0.55, CH_2Cl_2). ^{1}H NMR spectroscopic analysis of the mixture indicated >20:1 d.r. The product was analyzed by HPLC to determine the enantiomeric excess: 96% e.e. (CHIRALPAK AD-H, hexane/*i*-PrOH = 70/30, detector: 290 nm, T = 25 °C, flow rate: 1.0 mL/min), t_1 (minor) = 22.11 min, t_2 (major) = 25.83 min.

Stereodivergent total synthesis of tapentadol

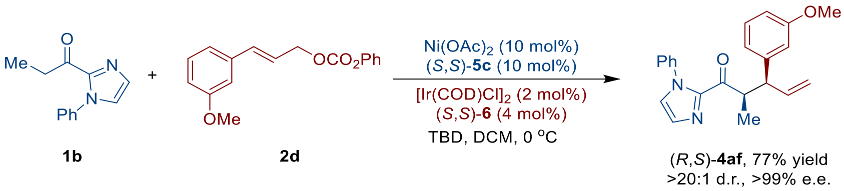

In a 10 mL Schlenk tube, [Ir(COD)Cl]$_2$ (0.002 mmol, 0.02 equiv.), (*S*,*S*)-**6** (0.004 mmol, 0.04 equiv.), TBD (0.01 mmol, 0.1 equiv.) were stirred in 1 mL DCM under argon at 25 °C for 10 min. **1b** (0.1 mmol, 1.0 equiv.), allylic carbonate **2d** (0.15 mmol, 1.5 equiv.) and (*S*,*S*)-Ni catalyst (0.01 mmol, 0.1equiv.) were then added successively and additional DCM (1 mL) was added. The mixture was stirred at 0 °C. After the reaction was complete (monitored by TLC), the residue was purified by flash column chromatography on silica gel to afford the desired product **4af** (77% yield, >20:1 d.r., >99% e.e.).

(2*R*,3*S*)-3-(3-methoxyphenyl)-2-methyl-1-(1-phenyl-1H-imidazol-2-yl)pent-4-en-1-one (4af)

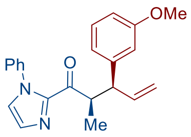

^{1}H NMR (400 MHz, CDCl$_3$) δ 7.51 – 7.42 (m, 3H), 7.31 (d, *J* = 1.0 Hz, 1H), 7.28 – 7.22 (m, 3H), 7.22 – 7.20 (m, 1H), 6.90 – 6.84 (m, 1H), 6.82 – 6.79 (m, 1H), 6.79 – 6.74 (m, 1H), 6.05 – 5.92 (m, 1H), 5.06 – 4.98 (m, 1H), 4.96 – 4.88 (m, 1H), 4.48 – 4.37 (m, 1H), 3.80 (s, 3H), 3.62 – 3.51 (m, 1H), 0.97 (d, *J* = 6.9 Hz, 3H). **^{13}C NMR (100 MHz, CDCl$_3$)** δ 194.69, 159.90, 143.66, 143.35, 140.44, 138.63, 129.76, 129.68, 129.08, 128.80, 127.45, 125.89, 120.83, 115.16, 114.15, 112.01, 55.33, 53.55, 45.34, 16.46. **ESI-MS:** calculated [$C_{22}H_{22}N_2O_2$ + H]$^+$: 347.1754, found: 347.1763. $[\alpha]^{20}_D$= -33.0 (c = 0.59, CH_2Cl_2). ^{1}H NMR spectroscopic analysis of the mixture indicated >20:1 d.r. The product was analyzed by HPLC to determine the enantiomeric excess: >99% e.e. (CHIRALPAK IBN-5, hexane/*i*-PrOH = 98/2, detector: 254 nm, T = 25 °C, flow rate: 1.0 mL/min), t_1 (major) = 9.82 min, t_2 (minor) = 10.84 min.

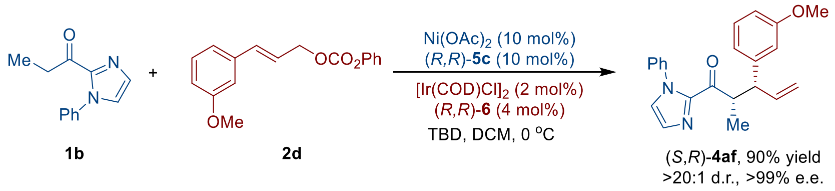

In a 10 mL Schlenk tube, [Ir(COD)Cl]$_2$ (0.002 mmol, 0.02 equiv.), (*R*,*R*)-**6** (0.004 mmol, 0.04 equiv.), TBD (0.01 mmol, 0.1 equiv.) were stirred in 1 mL DCM under argon at 25 °C for 10 min. **1b** (0.1 mmol, 1.0 equiv.), allylic carbonate **2d** (0.15 mmol, 1.5equiv.) and (*R*,*R*)-Ni catalyst (0.01 mmol,

0.1equiv.) were then added successively and additional DCM (1 mL) was added. The mixture was stirred at 0 °C. After the reaction was complete (monitored by TLC), the residue was purified by flash column chromatography on silica gel to afford the desired product **4af** (90% yield, >20:1 d.r., >99% e.e.).

(2*S*,3*R*)-3-(3-methoxyphenyl)-2-methyl-1-(1-phenyl-1H-imidazol-2-yl)pent-4-en-1-one (4af)

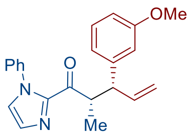

^{1}H NMR (500 MHz, $CDCl_3$) δ 7.48 – 7.44 (m, 3H), 7.31 (d, *J* = 0.9 Hz, 1H), 7.26 – 7.19 (m, 4H), 6.89 – 6.85 (m, 1H), 6.82 – 6.79 (m, 1H), 6.78 – 6.74 (m, 1H), 6.03 – 5.95 (m, 1H), 5.04 – 4.99 (m, 1H), 4.93 – 4.90 (m, 1H), 4.46 – 4.39 (m, 1H), 3.80 (s, 3H), 3.59 – 3.54 (m, 1H), 0.97 (d, *J* = 6.9 Hz, 3H). **^{13}C NMR (125 MHz, $CDCl_3$)** δ 194.69, 159.88, 143.65, 143.32, 140.43, 138.61, 129.76, 129.67, 129.07, 128.80, 127.46, 125.88, 120.82, 115.16, 114.13, 111.99, 55.32, 53.54, 45.31, 16.46. **ESI-MS:** calculated $[C_{22}H_{22}N_2O_2 + H]^+$: 347.1754, found: 347.1763. $[\alpha]^{20}_{D}$= 28.4 (c = 0.73, CH_2Cl_2). ^{1}H NMR spectroscopic analysis of the mixture indicated >20:1 d.r. The product was analyzed by HPLC to determine the enantiomeric excess: >99% e.e. (CHIRALPAK IBN-5, hexane/*i*-PrOH = 98/2, detector: 254 nm, T = 25 °C, flow rate: 1.0 mL/min), t_1 (minor) = 9.50 min, t_2 (major) = 10.30 min.

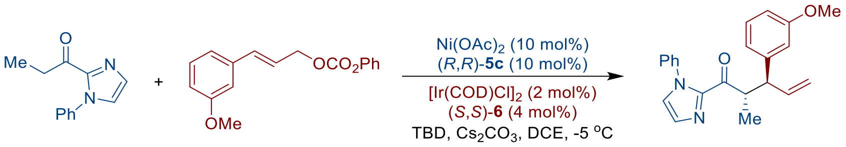

1b **2d** (*S*,*S*)-**4af**, 71% yield
14:1 d.r., >99% e.e.

Procedure A: In a 10 mL Schlenk tube, $[Ir(COD)Cl]_2$ (0.006 mmol, 0.02 equiv.), (*S*,*S*)-**6** (0.012 mmol, 0.04 equiv.), TBD (0.03mmol, 0.1 equiv.) were stirred in 0.5 mL DCE under argon at 25 °C for 1 huor. **1b** (0.3 mmol, 1.0 equiv.), allylic carbonate **2d** (0.9 mmol, 3.0 equiv.), Cs_2CO_3 (0.3 mmol, 1.0 equiv.) and (*R*,*R*)-Ni catalyst (0.03 mmol, 0.1 equiv.) were then added successively and additional DCE (0.5 mL) was added. The mixture was stirred at -5 °C. After the reaction was complete (monitored by TLC), the residue was purified by flash column chromatography on silica gel to afford the desired product **4af** (74% yield, 14:1 d.r., >99% e.e.).

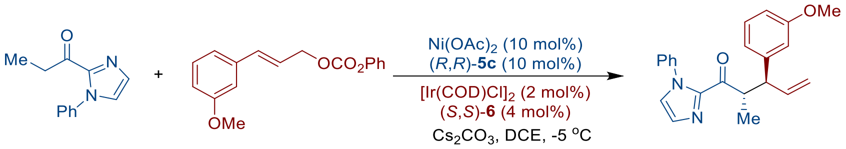

1b **2d** (*S*,*S*)-**4af**, 88% yield
>20:1 d.r., >99% e.e.

Procedure B: In a 10 mL Schlenk tube, $[Ir(COD)Cl]_2$ (0.006 mmol, 0.02 equiv.), (*S*,*S*)-**6** (0.012 mmol, 0.04 equiv.) were stirred in 0.5 mL THF and 0.5 mL *n*-propylamine under argon at 50 °C for 30 min and then the volatile solvents were removed in vacuo to give a pale yellow solid. After that, **1b** (0.3 mmol, 1.0 equiv.), allylic carbonate **2d** (0.9 mmol, 3.0 equiv.), Cs_2CO_3 (0.3 mmol, 1.0 equiv.), (*R*,*R*)-Ni catalyst (0.03 mmol, 0.1equiv.), and 1 mL DCE were added and stirred at -5 °C. After the reaction was complete (monitored by TLC), the residue was purified by flash column chromatography on silica gel to afford the desired product **4af** (88% yield, >20:1 d.r., >99% e.e.).

(2*S*,3*S*)-3-(3-methoxyphenyl)-2-methyl-1-(1-phenyl-1H-imidazol-2-yl)pent-4-en-1-one(4af)

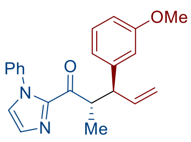

^{1}H NMR (400 MHz, $CDCl_3$) δ 7.39 – 7.27 (m, 3H), 7.22 (d, *J* = 1.0 Hz, 1H), 7.13 (t, *J* = 7.9 Hz, 1H), 7.06 (d, *J* = 1.0 Hz, 1H), 6.86 – 6.80 (m, 1H), 6.79 – 6.72 (m, 3H), 6.71 – 6.67 (m, 1H), 6.02 – 5.90 (m, 1H), 5.16 – 5.04 (m, 2H), 4.45 – 4.35 (m, 1H), 3.71 (s, 3H), 3.57 (t, *J* = 9.9 Hz, 1H), 1.28 (d, *J* = 6.9 Hz, 3H). **^{13}C NMR (100**

MHz, $CDCl_3$) δ 194.56, 159.68, 144.35, 143.50, 139.70, 138.26, 129.51, 129.43, 128.92, 128.55, 126.87, 125.53, 120.64, 116.41, 113.44, 112.59, 55.26, 54.22, 45.30, 16.53. **ESI-MS:** calculated $[C_{22}H_{22}N_2O_2 + H]^+$: 347.1754, found: 347.1763. $[\alpha]^{20}_D$= -21.7 (c = 0.49, CH_2Cl_2). ^{1}H NMR spectroscopic analysis of the mixture indicated >20:1 d.r. The product was analyzed by HPLC to determine the enantiomeric excess: >99% e.e. (CHIRALPAK IBN-5, hexane/*i*-PrOH = 98/2, detector: 254 nm, T = 25 °C, flow rate: 1.0 mL/min), t_1 (minor) = 9.20 min, t_2 (major) = 9.62 min.

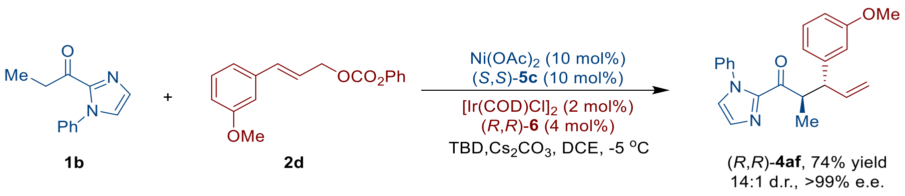

Procedure A: In a 10 mL Schlenk tube, $[Ir(COD)Cl]_2$ (0.006 mmol, 0.02 equiv.), (*R*,*R*)-**6** (0.012 mmol, 0.04 equiv.), TBD (0.03mmol, 0.1 equiv.) were stirred in 0.5 mL DCE under argon at 25 °C for 1 hour. **1b** (0.3 mmol, 1.0 equiv.), allylic carbonate **2d** (0.9 mmol, 3.0 equiv.), Cs_2CO_3 (0.3 mmol, 1.0 equiv.), and (*S*,*S*)-Ni catalyst (0.03 mmol, 0.1equiv.) were then added successively and additional DCE (0.5 mL) was added. The mixture was stirred at -5 °C. After the reaction was complete (monitored by TLC), the residue was purified by flash column chromatography on silica gel to afford the desired product **4af** (74% yield, 14:1 d.r., >99% e.e.).

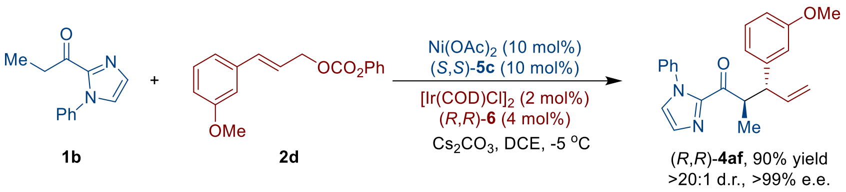

Procedure B: In a 10 mL Schlenk tube, $[Ir(COD)Cl]_2$ (0.006 mmol, 0.02 equiv.), (*R*,*R*)-**6** (0.012 mmol, 0.04 equiv.) were stirred in 0.5 mL THF and 0.5 mL *n*-propylamine under argon at 50 °C for 30 min and then the volatile solvents were removed in vacuo to give a pale yellow solid. After that, **1b** (0.3 mmol, 1.0 equiv.), allylic carbonate **2d** (0.9 mmol, 3.0 equiv.), Cs_2CO_3 (0.3 mmol, 1.0 equiv.), (*S*,*S*)-Ni catalyst (0.03 mmol, 0.1 equiv.), and 1 mL DCE were added and stirred at -5 °C. After the reaction was complete (monitored by TLC), the residue was purified by flash column chromatography on silica gel to afford the desired product **4af** (90% yieid, >20:1, >99% e.e.).

(2*R*,3*R*)-3-(3-methoxyphenyl)-2-methyl-1-(1-phenyl-1H-imidazol-2-yl)pent-4-en-1-one (4af)

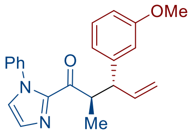

^{1}H NMR (400 MHz, $CDCl_3$) δ 7.37 – 7.26 (m, 3H), 7.21 (d, *J* = 1.0 Hz, 1H), 7.12 (t, *J* = 7.9 Hz, 1H), 7.04 (d, *J* = 1.0 Hz, 1H), 6.87 – 6.80 (m, 1H), 6.78 – 6.76 (m, 1H), 6.76 – 6.71 (m, 2H), 6.70 – 6.66 (m, 1H), 6.02 – 5.90 (m, 1H), 5.16 – 5.05 (m, 2H), 4.45 – 4.36 (m, 1H), 3.70 (s, 3H), 3.60 – 3.54 (m, 1H), 1.28 (d, *J* = 6.9 Hz, 3H). **^{13}C NMR (100 MHz, $CDCl_3$)** δ 194.49, 159.64, 144.29, 143.43, 139.66, 138.20, 129.46, 129.38, 128.87, 128.49, 126.83, 125.48, 120.58, 116.36, 113.41, 112.53, 55.19, 54.16, 45.26, 16.49. **ESI-MS:** calculated $[C_{22}H_{22}N_2O_2 + H]^+$: 347.1754, found: 347.1763. $[\alpha]^{20}_D$= 18.3 (c = 2.26, CH_2Cl_2). ^{1}H NMR spectroscopic analysis of the mixture indicated >20:1 d.r. The product was analyzed by HPLC to determine the enantiomeric excess: >99% e.e. (CHIRALPAK IBN-5, hexane/*i*-PrOH = 98/2, detector: 254 nm, T = 25 °C, flow rate: 1.0 mL/min), t_1 (major) = 8.83 min, t_2 (minor) = 9.61 min.

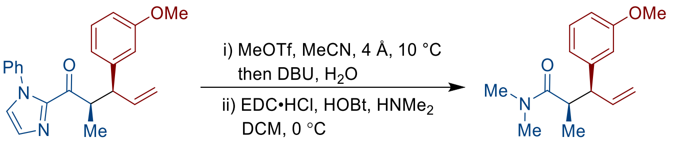

(*R*,*S*)-**4af**
>20:1 d.r., >99% e.e.

(*R*,*S*)-**18**, 55% yield
>20:1 d.r., >99% e.e.

To a solution of **4af** (177 mg, 0.51 mmol, 1.0 equiv.) in dry MeCN (6 mL) was added 4 Å molecular sieves (600 mg) and the mixture was stirred vigorously for 2 hours at 10 °C under an argon atmosphere. Methyl trifluoromethanesulfonate (125.5 mg, 0.76 mmol, 1.5 equiv.) was then added and the mixture was stirred for 12 hours. After complete consumption of the starting material **4af**, water (1.0 mL) and 1,8- diazabicyclo[5.4.0]undec-7-ene (116.2 mg, 0.76 mmol, 1.5 equiv.) were added stepwise. The mixture was stirred at 10 °C under an argon atmosphere. After 2 hours the reaction was diluted with ethyl acetate and 1N HCl. The organic layers were separated and the aqueous layer was extracted with ethyl acetate. The combined organic layers were dried over anhydrous $MgSO_4$, filtered, and concentrated under a vacuum. The residue was purified by flash silica gel to afford the desired product **11**. To a solution of **11** in DCM (5 mL) at 0 °C was added $HNMe_2$ (2 mL, 2.0 mol/L in THF), EDC•HCl (116.9 mg, 0.61 mmol, 1.2 equiv.), and HOBt (82.4 mg, 0.61 mmol, 1.2 equiv.) under argon atmosphere. The resulting solution was stirred for 4 hours at 0 °C. The reaction mixture was diluted with DCM and washed twice with water and once with brine. The organic layer was dried over anhydrous $MgSO_4$, filtered, and concentrated under a vacuum. The crude product was purified by flash chromatography to afford the desired product **18** (68.8 mg, 55% yield, >20:1 d.r., >99% e.e.).

(2*R*,3*S*)-3-(3-methoxyphenyl)-N,N,2-trimethylpent-4-enamide (18)

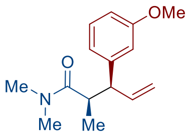

^{1}H NMR (400 MHz, $CDCl_3$) δ 7.26 – 7.21 (m, 1H), 6.83 – 6.73 (m, 3H), 6.03 – 5.93 (m, 1H), 5.04 – 4.94 (m, 2H), 3.81 (s, 3H), 3.62 – 3.55 (m, 1H), 3.15 – 3.03 (m, 4H), 2.98 (s, 3H), 0.91 (d, J = 6.8 Hz, 3H). **^{13}C NMR (100 MHz, $CDCl_3$)** δ 175.53, 159.84, 143.73, 139.90, 129.65, 120.90, 115.54, 114.61, 111.57, 55.31, 53.62, 40.46, 37.56, 35.84, 16.49. $[\alpha]^{20}_D$= -62.8 (c = 0.49, CH_2Cl_2). ^{1}H NMR spectroscopic analysis of the mixture indicated >20:1 d.r. The product was analyzed by HPLC to determine the enantiomeric excess: >99% e.e. (CHIRALPAK AD-H, hexane/*i*-PrOH = 98/2, detector: 276 nm, T = 25 °C, flow rate: 1.0 mL/min), t_1 (minor) = 16.39min, t_2 (major) = 21.50 min.

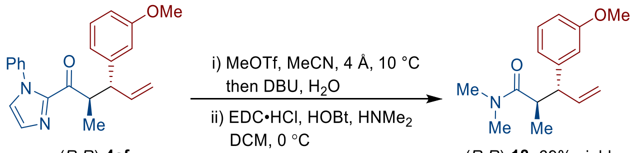

(*R*,*R*)-**4af**
>20:1 d.r., >99% e.e.

(*R*,*R*)-**18**, 69% yield
>20:1 d.r., >99% e.e.

To a solution of **4af** (186 mg, 0.54 mmol, 1.0 equiv.) in dry MeCN (6 mL) was added 4 Å molecular sieves (600 mg) and the mixture was stirred vigorously for 2 hours at 10 °C under an argon atmosphere. Methyl trifluoromethanesulfonate (132.8 mg, 0.81 mmol, 1.5 equiv.) was then added and the mixture was stirred for 12 hours. After complete consumption of the starting material **4af**, water (1.0 mL) and 1,8- diazabicyclo[5.4.0]undec-7-ene (123.1 mg, 0.81 mmol, 1.5 equiv.) were added stepwise. The mixture was stirred at 10 °C under an argon atmosphere. After 2 hours the reaction was diluted with ethyl acetate and 1N HCl. The organic layers were separated and the aqueous layer was extracted with ethyl acetate. The combined organic layers were dried over anhydrous $MgSO_4$, filtered, and concentrated under

a vacuum. The residue was purified by flash silica gel to afford the desired product **11**. To a solution of **11** in DCM (5 mL) at 0 °C was added $HNMe_2$ (2 mL, 2.0 mol/L in THF), EDC•HCl (124.6 mg, 0.65 mmol, 1.2 equiv.), and HOBt (87.8 mg, 0.65 mmol, 1.2 equiv.) under argon atmosphere. The resulting solution was stirred for 4 hours at 0 °C. The reaction mixture was diluted with DCM and washed twice with water and once with brine. The organic layer was dried over anhydrous $MgSO_4$, filtered, and concentrated under a vacuum. The crude product was purified by flash chromatography to afford the desired product **18** (91.7 mg, 69% yield, >20:1 d.r., >99% e.e.).

(2*R*,3*R*)-3-(3-methoxyphenyl)-N,N,2-trimethylpent-4-enamide (18)

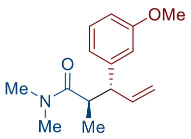

^{1}H NMR (500 MHz, CDCl$_3$) δ 7.17 (t, *J* = 7.9 Hz, 1H), 6.82 – 6.69 (m, 3H), 5.99 – 5.91 (m, 1H), 5.21 – 5.07 (m, 2H), 3.77 (s, 3H), 3.55 (t, *J* = 10.0 Hz, 1H), 3.08 – 3.03 (m, 1H), 2.77 (s, 3H), 2.73 (s, 3H). **^{13}C NMR (125 MHz, CDCl$_3$)** δ 175.26, 159.60, 145.01, 139.16, 129.40, 119.95, 116.86, 113.40, 111.81, 55.28, 54.04, 40.48, 37.26, 35.59, 16.59. **ESI-MS:** calculated $[C_{15}H_{21}NO_2 + H]^+$: 248.1645, found: 248.1651. $[\alpha]^{20}_{D}$= 32.3 (c = 1.00, CH_2Cl_2). ^{1}H NMR spectroscopic analysis of the mixture indicated >20:1 d.r. The product was analyzed by HPLC to determine the enantiomeric excess: >99% e.e. (CHIRALPAK AD-H, hexane/*i*-PrOH = 98/2, detector: 276 nm, T = 25 °C, flow rate: 1.0 mL/min), t_1 (major) = 14.76min, t_2 (minor) = 17.16 min.

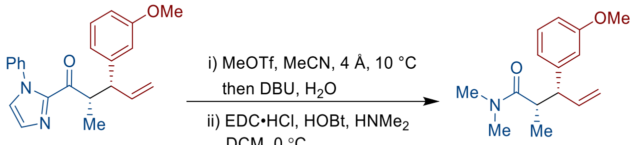

To a solution of **4af** (138.4 mg, 0.40 mmol, 1.0 equiv.) in dry MeCN (6 mL) was added 4 Å molecular sieves (600 mg) and the mixture was stirred vigorously for 2 hours at 10 °C under an argon atmosphere. Methyl trifluoromethanesulfonate (98.4 mg, 0.60 mmol, 1.5 equiv.) was then added and the mixture was stirred for 12 hours. After complete consumption of the starting material **4af**, water (1.0 mL) and 1,8- diazabicyclo[5.4.0]undec-7-ene (91.2 mg, 0.60 mmol, 1.5 equiv.) were added stepwise. The mixture was stirred at 10 °C under an argon atmosphere. After 2 hours the reaction was diluted with ethyl acetate and 1N HCl. The organic layers were separated and the aqueous layer was extracted with ethyl acetate. The combined organic layers were dried over anhydrous $MgSO_4$, filtered, and concentrated under a vacuum. The residue was purified by flash silica gel to afford the desired product **11**. To a solution of **11** in DCM (5 mL) at 0 °C was added $HNMe_2$ (2 mL, 2.0 mol/L in THF), EDC•HCl (92 mg, 0.48 mmol, 1.2 equiv.), and HOBt (64.8 mg, 0.48 mmol, 1.2 equiv.) under argon atmosphere. The resulting solution was stirred for 4 hours at 0 °C. The reaction mixture was diluted with DCM and washed twice with water and once with brine. The organic layer was dried over anhydrous $MgSO_4$, filtered, and concentrated under a vacuum. The crude product was purified by flash chromatography to afford the desired product **18** (68.0 mg, 69% yield, >20:1 d.r., >99% e.e.).

(2*S*,3*R*)-3-(3-methoxyphenyl)-N,N,2-trimethylpent-4-enamide (18)

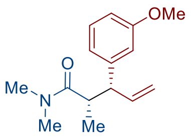

^{1}H NMR (500 MHz, CDCl$_3$) δ 7.26 – 7.21 (m, 1H), 6.83 – 6.75 (m, 3H), 6.02 – 5.93 (m, 1H), 5.02 – 4.96 (m, 2H), 3.81 (s, 3H), 3.62 – 3.56 (m, 1H), 3.12 – 3.05 (m, 4H), 2.98 (s, 3H), 0.91 (d, *J* = 6.8 Hz, 3H). **^{13}C NMR (125 MHz, CDCl$_3$)** δ 175.53, 159.83, 143.73, 139.90, 129.65, 120.90, 115.55, 114.60, 111.56, 55.31, 53.62, 40.45, 37.57, 35.85, 16.50. **ESI-MS:** calculated $[C_{15}H_{21}NO_2 + H]^+$: 248.1645, found: 248.1651. $[\alpha]^{20}_{D}$= -62.8 (c = 0.49, CH_2Cl_2). ^{1}H NMR spectroscopic analysis of the mixture indicated >20:1 d.r. The product was analyzed

by HPLC to determine the enantiomeric excess: >99% e.e. (CHIRALPAK AD-H, hexane/*i*-PrOH = 98/2, detector: 276 nm, T = 25 °C, flow rate: 1.0 mL/min), t_1 (major) = 16.17min, t_2 (minor) = 21.25 min.

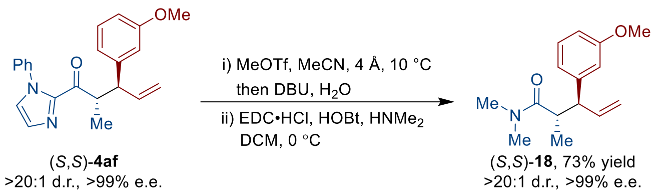

To a solution of **4af** (156 mg, 0.45 mmol, 1.0 equiv.) in dry MeCN (5 mL) was added 4 Å molecular sieves (500 mg) and the mixture was stirred vigorously for 2 hours at 10 °C under an argon atmosphere. Methyl trifluoromethanesulfonate (110.7 mg, 0.68 mmol, 1.5 equiv.) was then added and the mixture was stirred for 12 hours. After complete consumption of the starting material **4af**, water (1.0 mL) and 1,8- diazabicyclo[5.4.0]undec-7-ene (102.6 mg, 0.68 mmol, 1.5 equiv.) were added stepwise. The mixture was stirred at 10 °C under an argon atmosphere. After 2 hours the reaction was diluted with ethyl acetate and 1N HCl. The organic layers were separated and the aqueous layer was extracted with ethyl acetate. The combined organic layers were dried over anhydrous $MgSO_4$, filtered, and concentrated under a vacuum. The residue was purified by flash silica gel to afford the desired product **11**. To a solution of **11** in DCM (5 mL) at 0 °C was added $HNMe_2$ (2 mL, 2.0 mol/L in THF), EDC•HCl (104 mg, 0.81 mmol, 1.2 equiv.), and HOBt (72.9 mg, 0.81 mmol, 1.2 equiv.) under argon atmosphere. The resulting solution was stirred for 4 hours at 0 °C. The reaction mixture was diluted with DCM and washed twice with water and once with brine. The organic layer was dried over anhydrous $MgSO_4$, filtered, and concentrated under a vacuum. The crude product was purified by flash chromatography to afford the desired product **18** (81.5 mg, 73% yield, >20:1 d.r., >99% e.e.).

(2*S*,3*S*)-3-(3-methoxyphenyl)-N,N,2-trimethylpent-4-enamide (18)

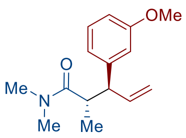

^{1}H NMR (500 MHz, CDCl$_3$) δ 7.17 (t, *J* = 7.9 Hz, 1H), 6.83 – 6.69 (m, 3H), 6.02 – 5.90 (m, 1H), 5.19 – 5.09 (m, 2H), 3.77 (s, 3H), 3.55 (t, *J* = 9.9 Hz, 1H), 3.10 – 3.03 (m, 1H), 2.77 (s, 3H), 2.73 (s, 3H), 1.17 (d, *J* = 6.7 Hz, 3H). **^{13}C NMR (125 MHz, CDCl$_3$)** δ 175.29, 159.62, 145.04, 139.17, 129.42, 119.98, 116.89, 113.43, 111.84, 55.30, 54.06, 40.51, 37.29, 35.62, 16.61. **ESI-MS:** calculated $[C_{15}H_{21}NO_2 + H]^+$: 248.1645, found: 248.1651. $[\alpha]^{20}_D$= -45.3 (c = 0.28, CH_2Cl_2). ^{1}H NMR spectroscopic analysis of the mixture indicated >20:1 d.r. The product was analyzed by HPLC to determine the enantiomeric excess: >99% e.e. (CHIRALPAK AD-H, hexane/*i*-PrOH = 98/2, detector: 276 nm, T = 25 °C, flow rate: 1.0 mL/min), t_1 (minor) = 15.04min, t_2 (major) = 17.29 min.

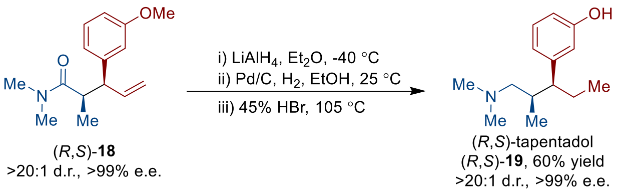

To a solution of **18** (49.4 mg, 0.2 mmol, 1.0 equiv.) in Et_2O (2 mL) at -40 °C was added $LiAlH_4$ (38.0 mg, 1.0 mmol, 5.0 equiv.). The mixture was allowed to stirred at -40 °C for 24 hours until complete consumption of **18** (monitored by TLC). The reaction was quenched with water and diluted with EtOAc. The aqueous phase was separated and extracted twice with EtOAc. The combined organic phase was dried over anhydrous $MgSO_4$ and concentrated under a vacuum. The residue was dissolved in 2 mL

ethanol and Pd/C (10 wt%, 5 mg) was added. The mixture was stirred under hydrogen atmosphere for 2 hours at room temperature, then filtered and concentrated in vacuo. The crude product and aqueous hydrobromic acid (46 % aq., 2 mL) was heated under stirring at 105 °C for 3 hours and cooled to room temperature. The excess acid of was degassed, and reaction mixture was neutralized with sodium bicarbonate. Resulting product was extracted with dichloromethane. The organic layers were washed with brine and dried over anhydrous $MgSO_4$, filtered, and concentrated under a vacuum. The residue was subjected to purification on a silica-gel column to afford compound **19** (26.6 mg, 60% yield, >20:1 d.r., >99% e.e.).

3-((2*R*,3*S*)-1-(dimethylamino)-2-methylpentan-3-yl)phenol (19)

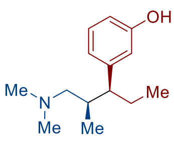

^{1}H NMR (400 MHz, $CDCl_3$) δ 7.09 (t, *J* = 7.8 Hz, 1H), 6.68 – 6.60 (m, 2H), 6.58 – 6.53 (m, 1H), 2.40 – 2.32 (m, 1H), 2.32 – 2.20 (m, 7H), 2.10 – 1.99 (m, 1H), 1.93 – 1.84 (m, 1H), 1.77 – 1.69 (m, 1H), 1.67 – 1.57 (m, 1H), 0.81 – 0.70 (m, 6H). **^{13}C NMR (100 MHz, $CDCl_3$)** δ 156.48, 144.53, 128.98, 121.34, 115.68, 113.59, 65.55, 50.97, 45.89, 35.88, 26.37, 16.00, 12.63. **ESI-MS:** calculated $[C_{14}H_{23}NO + H]^+$: 222.1852, found: 222.1861. $[\alpha]^{20}_D$= -15.2 (c = 0.77, CH_2Cl_2). ^{1}H NMR spectroscopic analysis of the mixture indicated >20:1 d.r. The product was analyzed by HPLC to determine the enantiomeric excess: >99% e.e. (CHIRALPAK AD-H, hexane/*i*-PrOH/DEA = 979/20/1, detector: 276 nm, T = 25 °C, flow rate: 1.0 mL/min), t_1 (minor) = 11.22 min, t_2 (major) = 12.32 min.

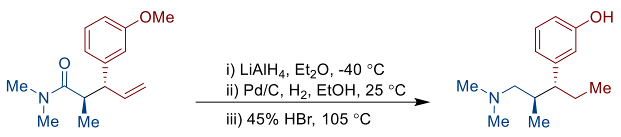

(*R*,*R*)-**18**
>20:1 d.r., >99% e.e.

(*R*,*R*)-tapentadol
(*R*,*R*)-**19**, 64% yield
>20:1 d.r., >99% e.e.

To a solution of **18** (49.4 mg, 0.2 mmol, 1.0 equiv.) in Et_2O (2 mL) at -40 °C was added $LiAlH_4$ (38.0 mg, 1.0 mmol, 5.0 equiv.). The mixture was allowed to stirred at -40 °C for 24 hours until complete consumption of **18** (monitored by TLC). The reaction was quenched with water and diluted with EtOAc. The aqueous phase was separated and extracted twice with EtOAc. The combined organic phase was dried over anhydrous $MgSO_4$ and concentrated under a vacuum. The residue was dissolved in 2 mL ethanol and Pd/C (10 wt%, 5 mg) was added. The mixture was stirred under hydrogen atmosphere for 2 hours at room temperature, then filtered and concentrated in vacuo. The crude product and aqueous hydrobromic acid (46 % aq., 2 mL) was heated under stirring at 105 °C for 3 hours and cooled to room temperature. The excess acid of was degassed, and reaction mixture was neutralized with sodium bicarbonate. Resulting product was extracted with dichloromethane. The organic layers were washed with brine and dried over anhydrous $MgSO_4$, filtered, and concentrated under a vacuum. The residue was subjected to purification on a silica-gel column to afford compound **19** (28.1 mg, 64% yield, >20:1 d.r., >99% e.e.).

3-((2*R*,3*R*)-1-(dimethylamino)-2-methylpentan-3-yl)phenol (19)

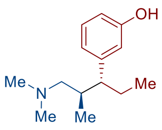

^{1}H NMR (500 MHz, $CDCl_3$) δ 7.12 (t, *J* = 7.8 Hz, 1H), 6.67 – 6.62 (m, 2H), 6.59 – 6.56 (m, 1H), 2.35 – 2.28 (m, 1H), 2.26 – 2.13 (m, 7H), 2.10 – 2.03 (m, 1H), 1.92 – 1.83 (m, 1H), 1.78 – 1.68 (m, 1H), 1.59 – 1.49 (m, 1H), 0.96 (d, *J* = 6.7 Hz, 3H), 0.69 (t, *J* = 7.3 Hz, 3H). **^{13}C NMR (125 MHz, $CDCl_3$)** δ 156.56, 146.04, 129.24, 120.37, 115.77, 113.41, 64.77, 51.31, 45.74, 36.62, 23.81, 16.12, 12.42. **ESI-MS:** calculated $[C_{14}H_{23}NO + H]^+$: 222.1852, found: 222.1861. $[\alpha]^{20}_D$= -26.4 (c = 0.66, CH_2Cl_2). ^{1}H NMR spectroscopic analysis of the mixture indicated >20:1 d.r. The product was analyzed by HPLC to determine the enantiomeric

excess: >99% e.e. (CHIRALPAK AD-H, hexane/*i*-PrOH/DEA = 979/20/1, detector: 276 nm, T = 25 °C, flow rate: 1.0 mL/min), t_1 (major) = 13.19 min, t_2 (minor) = 15.34 min.

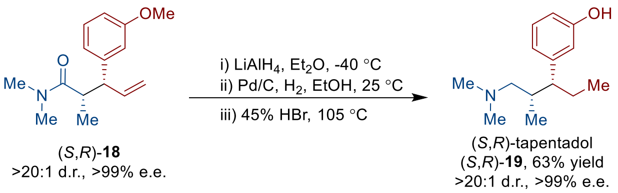

To a solution of **18** (49.4 mg, 0.2 mmol, 1.0 equiv.) in Et_2O (2 mL) at -40 °C was added $LiAlH_4$ (38.0 mg, 1.0 mmol, 5.0 equiv.). The mixture was allowed to stirred at -40 °C for 24 hours until complete consumption of **18** (monitored by TLC). The reaction was quenched with water and diluted with EtOAc. The aqueous phase was separated and extracted twice with EtOAc. The combined organic phase was dried over anhydrous $MgSO_4$ and concentrated under a vacuum. The residue was dissolved in 2 mL ethanol and Pd/C (10 wt%, 5 mg) was added. The mixture was stirred under hydrogen atmosphere for 2 hours at room temperature, then filtered and concentrated in vacuo. The crude product and aqueous hydrobromic acid (46 % aq., 2 mL) was heated under stirring at 105 °C for 3 hours and cooled to room temperature. The excess acid of was degassed, and reaction mixture was neutralized with sodium bicarbonate. Resulting product was extracted with dichloromethane. The organic layers were washed with brine and dried over anhydrous $MgSO_4$, filtered, and concentrated under a vacuum. The residue was subjected to purification on a silica-gel column to afford compound **19** (27.5 mg, 63% yield, >20:1 d.r., >99% e.e.).

3-((2*S*,3*R*)-1-(dimethylamino)-2-methylpentan-3-yl)phenol (19)

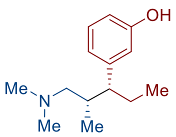

^{1}H NMR (500 MHz, $CDCl_3$) δ 7.09 (t, *J* = 7.8 Hz, 1H), 6.67 – 6.60 (m, 2H), 6.57 – 6.54 (m, 1H), 2.40 – 2.33 (m, 1H), 2.31 – 2.19 (m, 7H), 2.07 – 1.98 (m, 1H), 1.93 – 1.84 (m, 1H), 1.77 – 1.69 (m, 1H), 1.67 – 1.57 (m, 1H), 0.82 – 0.69 (m, 6H). **^{13}C NMR (125 MHz, $CDCl_3$)** δ 156.51, 144.49, 128.97, 121.28, 115.69, 113.57, 65.50, 50.95, 45.87, 35.85, 26.35, 15.97, 12.64. **ESI-MS:** calculated $[C_{14}H_{23}NO + H]^+$: 222.1852, found: 222.1861. $[\alpha]^{20}_D$= 7.0 (c = 1.22, CH_2Cl_2). ^{1}H NMR spectroscopic analysis of the mixture indicated >20:1 d.r. The product was analyzed by HPLC to determine the enantiomeric excess: >99% e.e. (CHIRALPAK AD-H, hexane/*i*-PrOH/DEA = 979/20/1, detector: 276 nm, T = 25 °C, flow rate: 1.0 mL/min), t_1 (major) = 11.53 min, t_2 (minor) = 12.75 min.

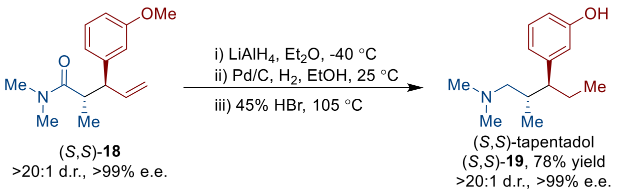

To a solution of **18** (49.4 mg, 0.2 mmol, 1.0 equiv.) in Et_2O (2 mL) at -40 °C was added $LiAlH_4$ (38.0 mg, 1.0 mmol, 5.0 equiv.). The mixture was allowed to stirred at -40 °C for 24 hours until complete consumption of **18** (monitored by TLC). The reaction was quenched with water and diluted with EtOAc. The aqueous phase was separated and extracted twice with EtOAc. The combined organic phase was dried over anhydrous $MgSO_4$ and concentrated under a vacuum. The residue was dissolved in 2 mL ethanol and Pd/C (10 wt%, 5 mg) was added. The mixture was stirred under hydrogen atmosphere for 2 hours at room temperature, then filtered and concentrated in vacuo. The crude product and aqueous hydrobromic acid (46 % aq., 2 mL) was heated under stirring at 105 °C for 3 hours and cooled to room temperature. The excess acid of was degassed, and reaction mixture was neutralized with sodium

bicarbonate. Resulting product was extracted with dichloromethane. The organic layers were washed with brine and dried over anhydrous $MgSO_4$, filtered, and concentrated under a vacuum. The residue was subjected to purification on a silica-gel column to afford compound **19** (34.5 mg, 78% yield, >20:1 d.r., >99% e.e.).

3-((2*S*,3*S*)-1-(dimethylamino)-2-methylpentan-3-yl)phenol (19)

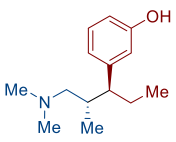

^{1}H NMR (500 MHz, CDCl$_3$) δ 7.11 (t, *J* = 7.8 Hz, 1H), 6.67 – 6.61 (m, 2H), 6.61 – 6.55 (m, 1H), 2.35 – 2.27 (m, 1H), 2.26 – 2.13 (m, 7H), 2.13 – 2.05 (m, 1H), 1.92 – 1.83 (m, 1H), 1.78 – 1.68 (m, 1H), 1.59 – 1.48 (m, 1H), 0.97 (d, *J* = 6.7 Hz, 3H), 0.69 (t, *J* = 7.3 Hz, 3H). **^{13}C NMR (125 MHz, CDCl$_3$)** δ 156.68, 145.94, 129.23, 120.26, 115.80, 113.48, 64.74, 51.37, 45.67, 36.57, 23.87, 16.16, 12.42. **ESI-MS:** calculated $[C_{14}H_{23}NO + H]^+$: 222.1852, found: 222.1861. $[\alpha]^{20}_{D}$= 21.6 (c = 1.05, CH_2Cl_2). ^{1}H NMR spectroscopic analysis of the mixture indicated >20:1 d.r. The product was analyzed by HPLC to determine the enantiomeric excess: >99% e.e. (CHIRALPAK AD-H, hexane/*i*-PrOH/DEA = 979/20/1, detector: 276 nm, T = 25 °C, flow rate: 1.0 mL/min), t_1 (minor) = 14.06 min, t_2 (major) = 16.05 min.

X-ray data

Characterization of 4n by X-ray crystallography:

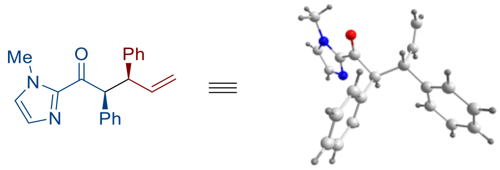

4n

Chemical formula	$C_{21}H_{20}N_2O$
Formula weight	316.39
Space group	P 1 21 1
Z	2
a, Å	8.3045(2)
b, Å	12.6728(4)
c, Å	9.2978(3)
α, °	90
β, °	115.844(2)
γ, °	90
V, Å^3	880.65(5)

3. Supplementary Figure

^{1}H NMR spectrum of **3a**

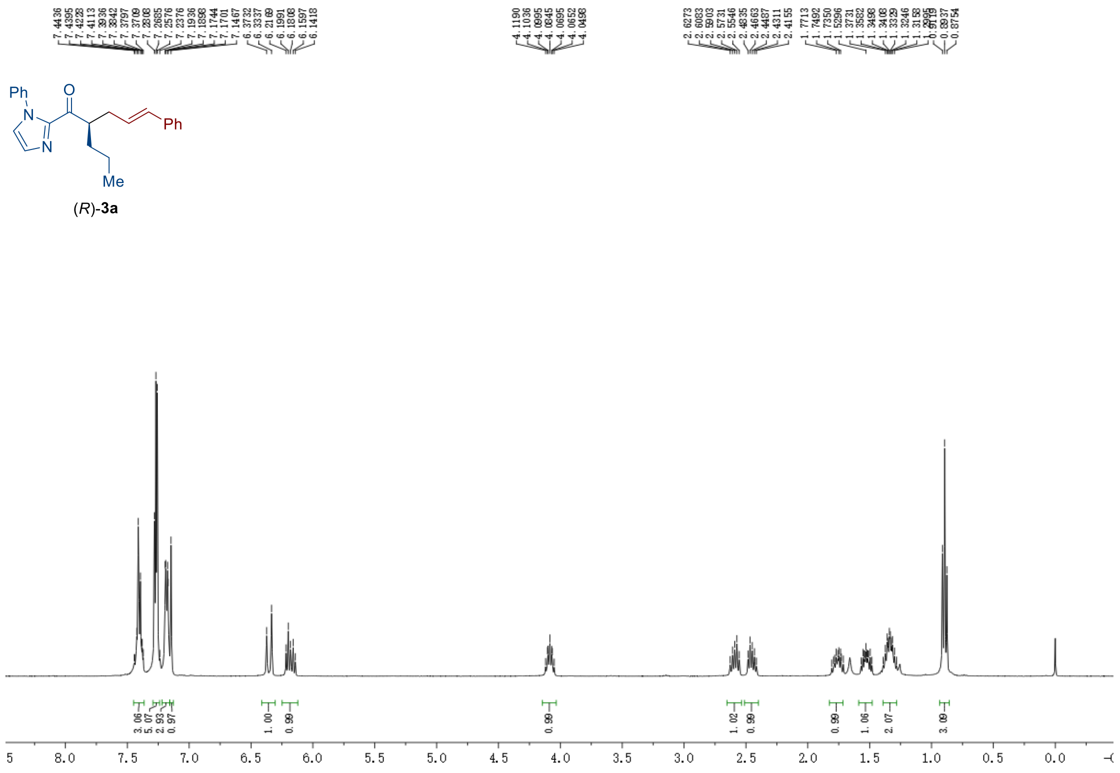

^{13}C NMR spectrum of **3a**

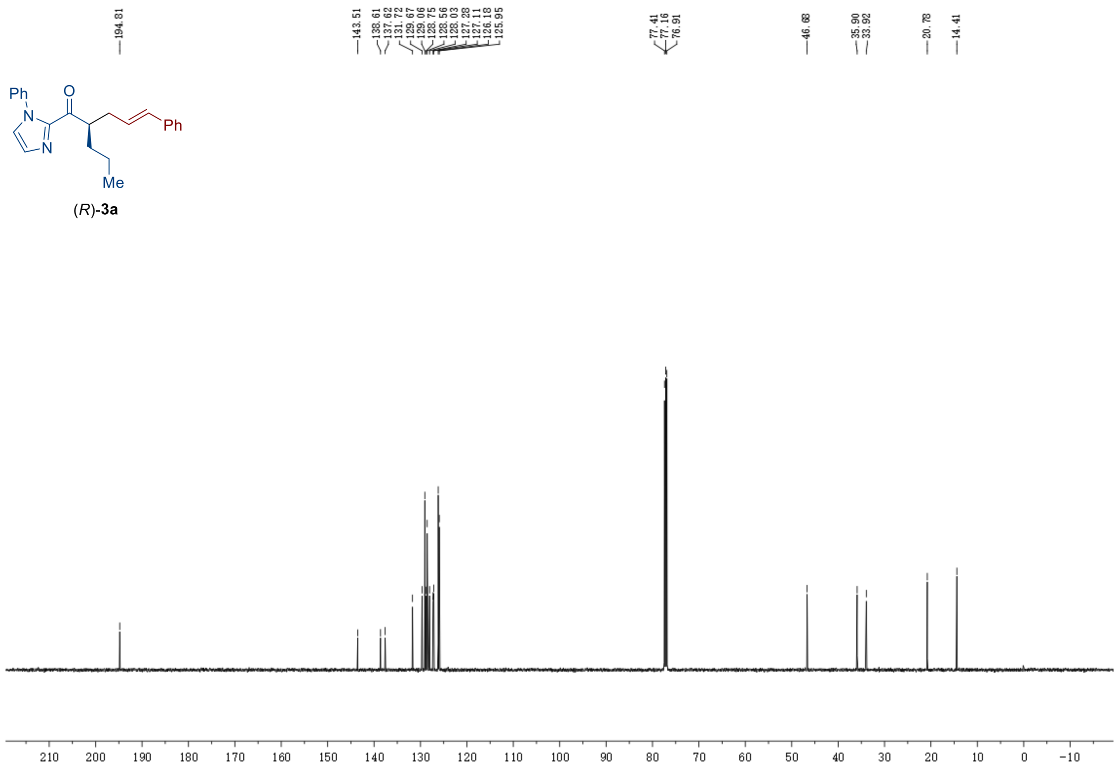

Supplementary Figure 3. NMR spectra of compound **3a**

^{1}H NMR spectrum of **3b**

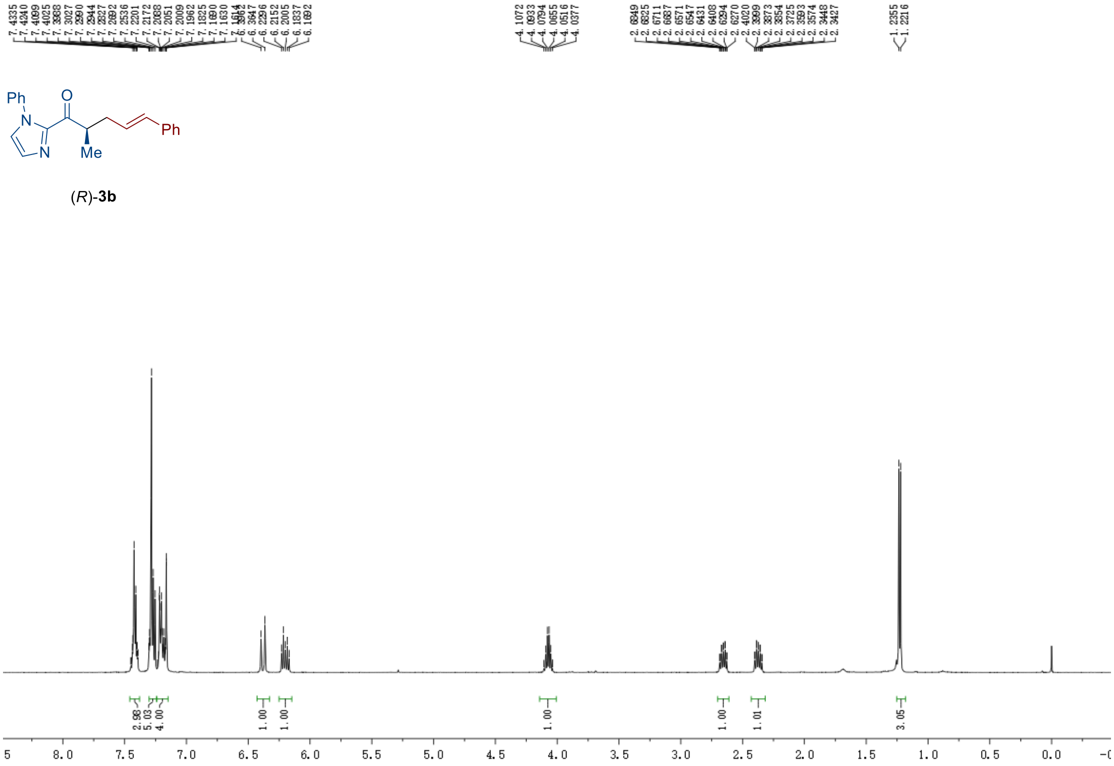

^{13}C NMR spectrum of **3b**

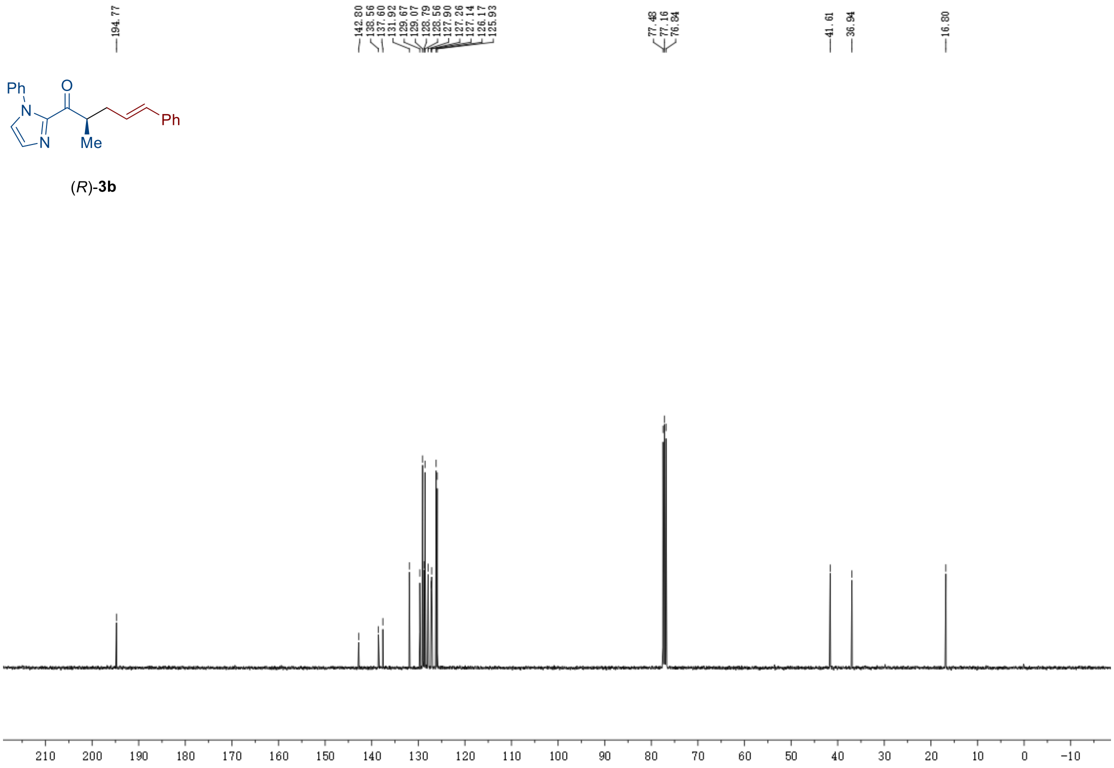

Supplementary Figure 4. NMR spectra of compound **3b**

^{1}H NMR spectrum of **3c**

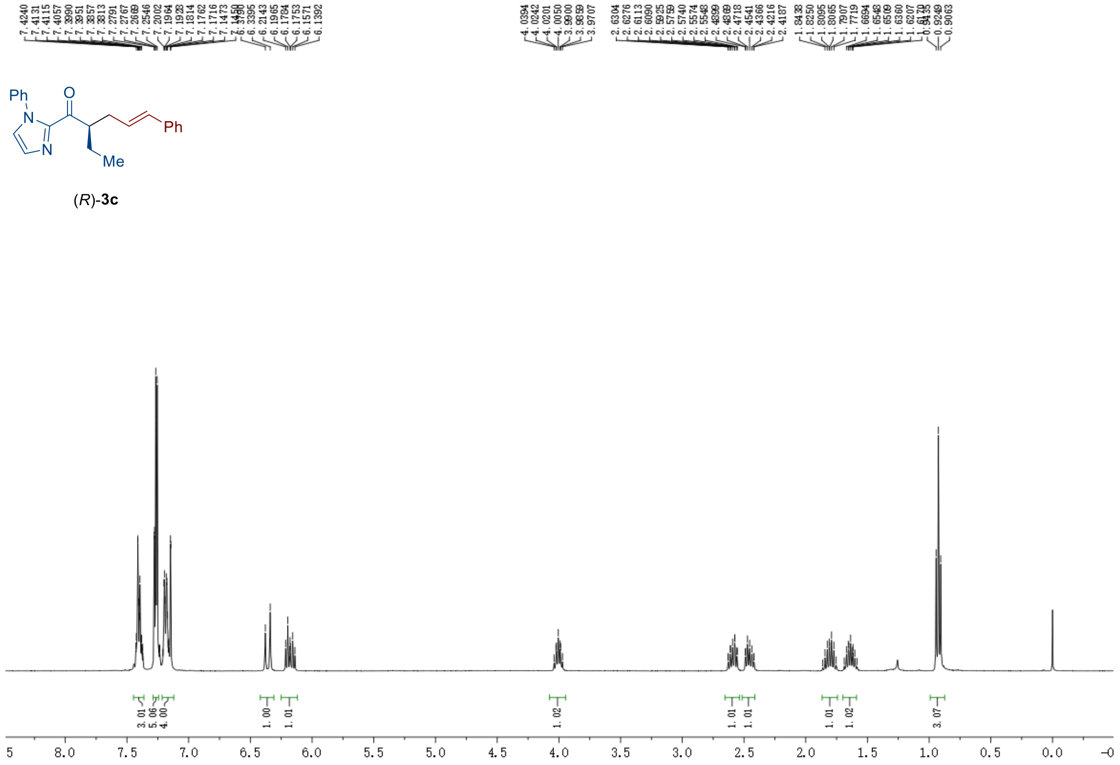

^{13}C NMR spectrum of **3c**

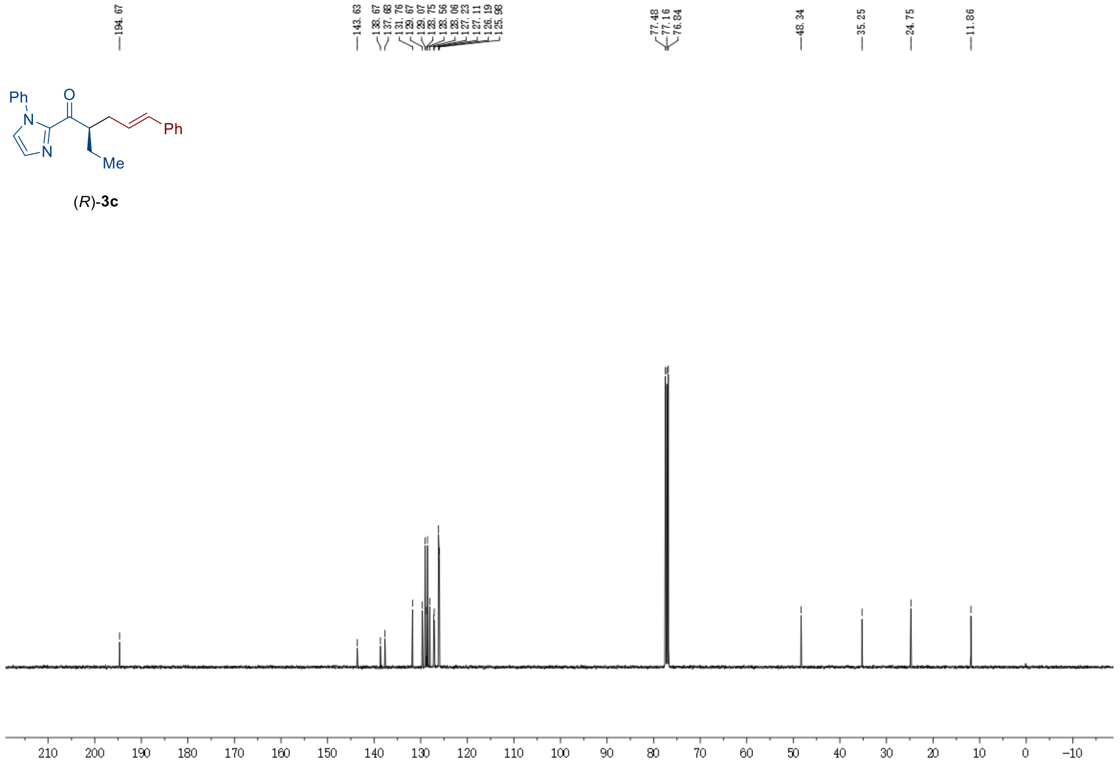

Supplementary Figure 5. NMR spectra of compound **3c**

^{1}H NMR spectrum of **3d**

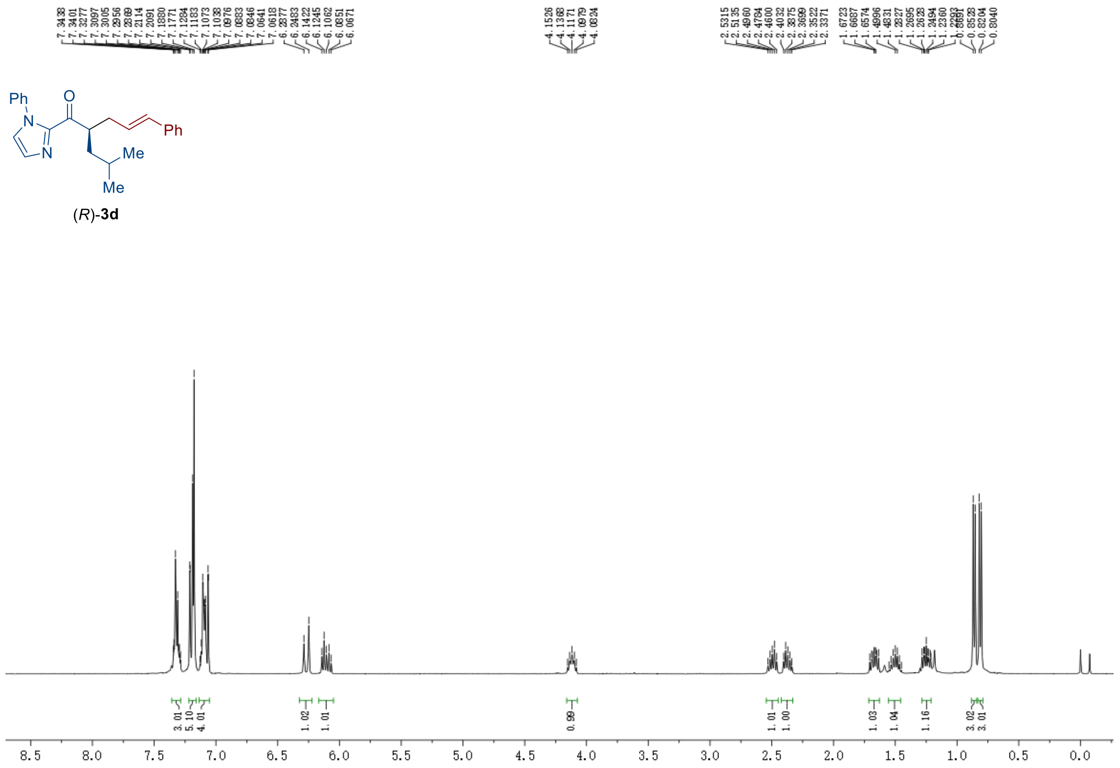

^{13}C NMR spectrum of **3d**

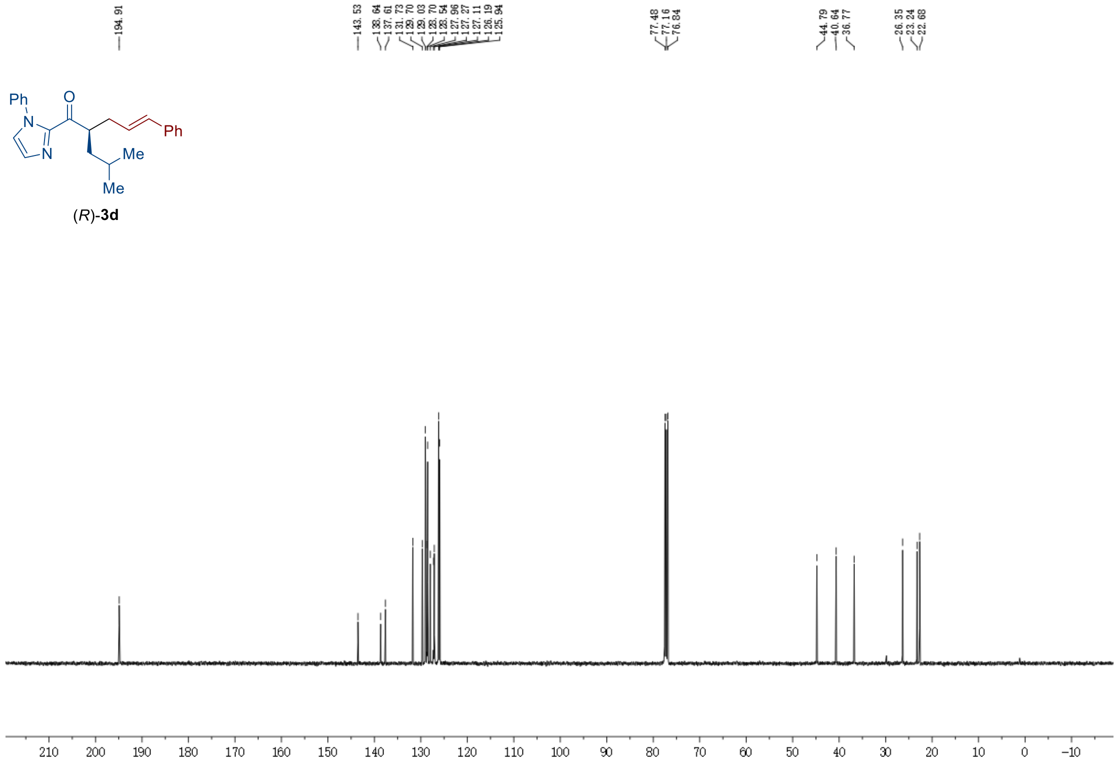

Supplementary Figure 6. NMR spectra of compound **3d**

^{1}H NMR spectrum of **3e**

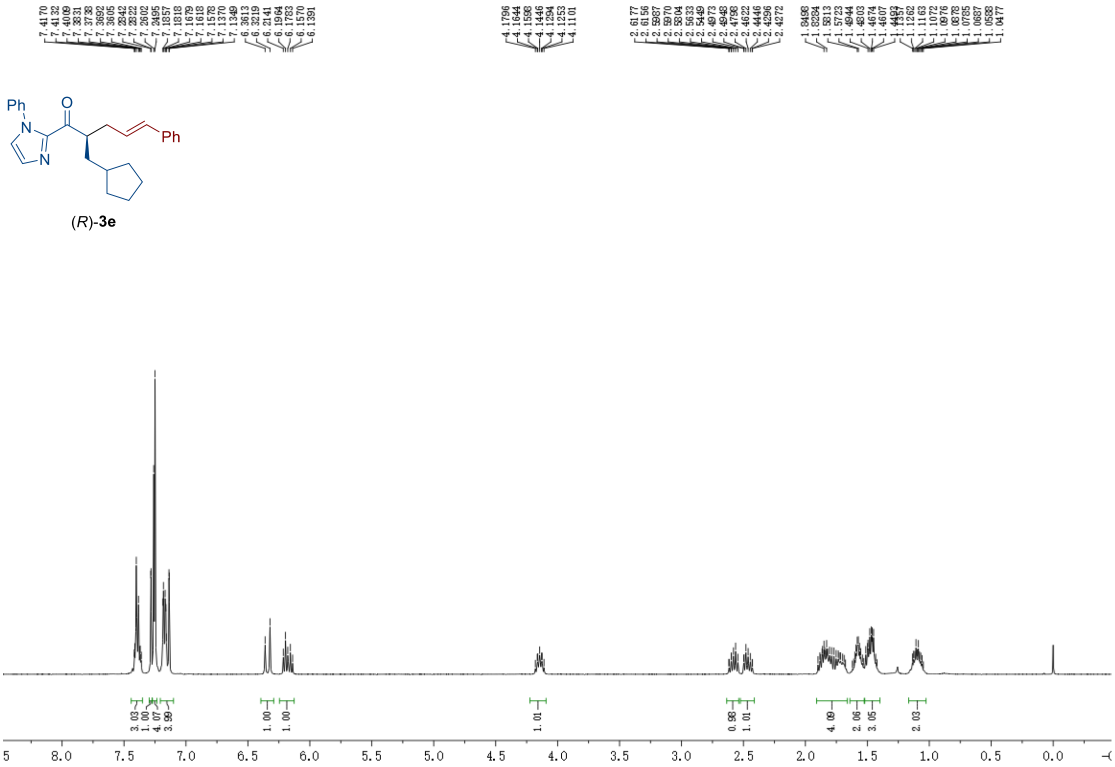

^{13}C NMR spectrum of **3e**

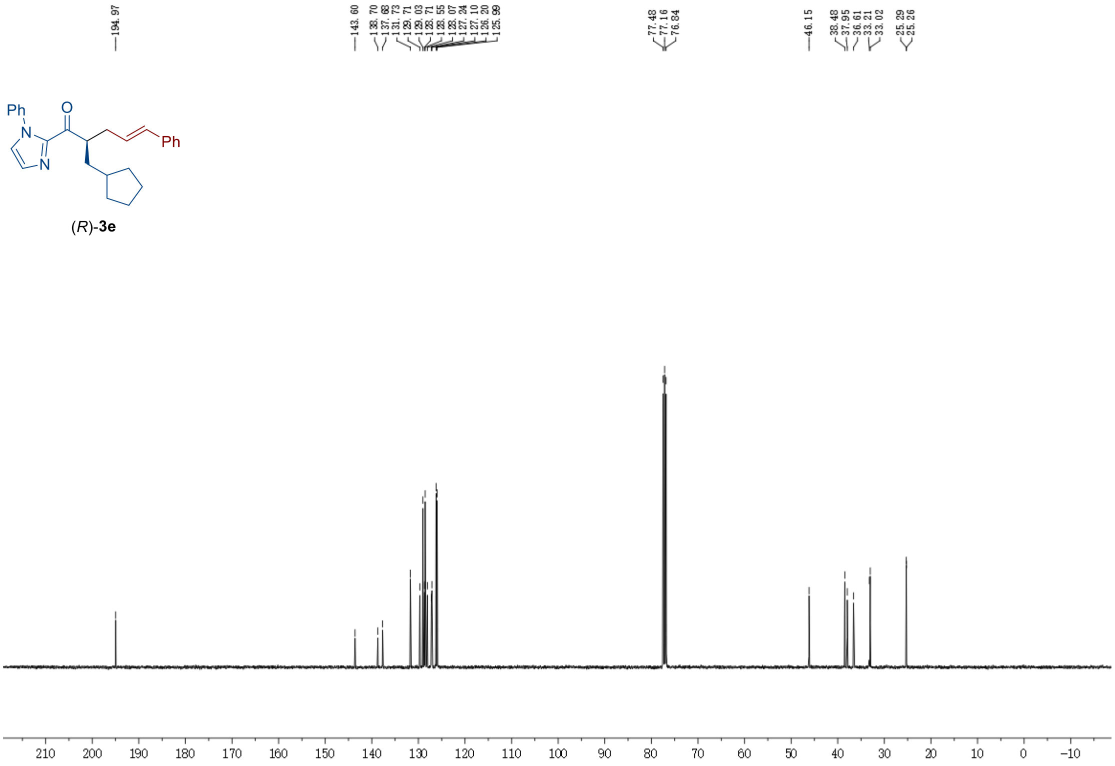

Supplementary Figure 7. NMR spectra of compound **3e**

^{1}H NMR spectrum of **3f**

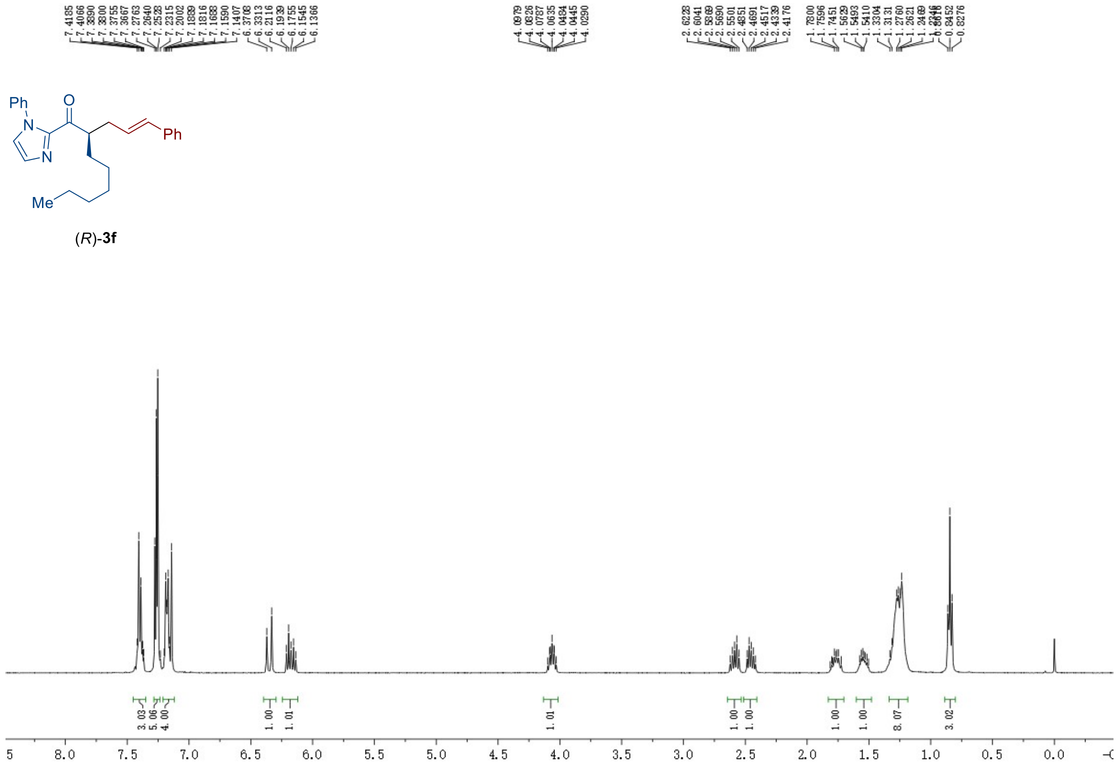

^{13}C NMR spectrum of **3f**

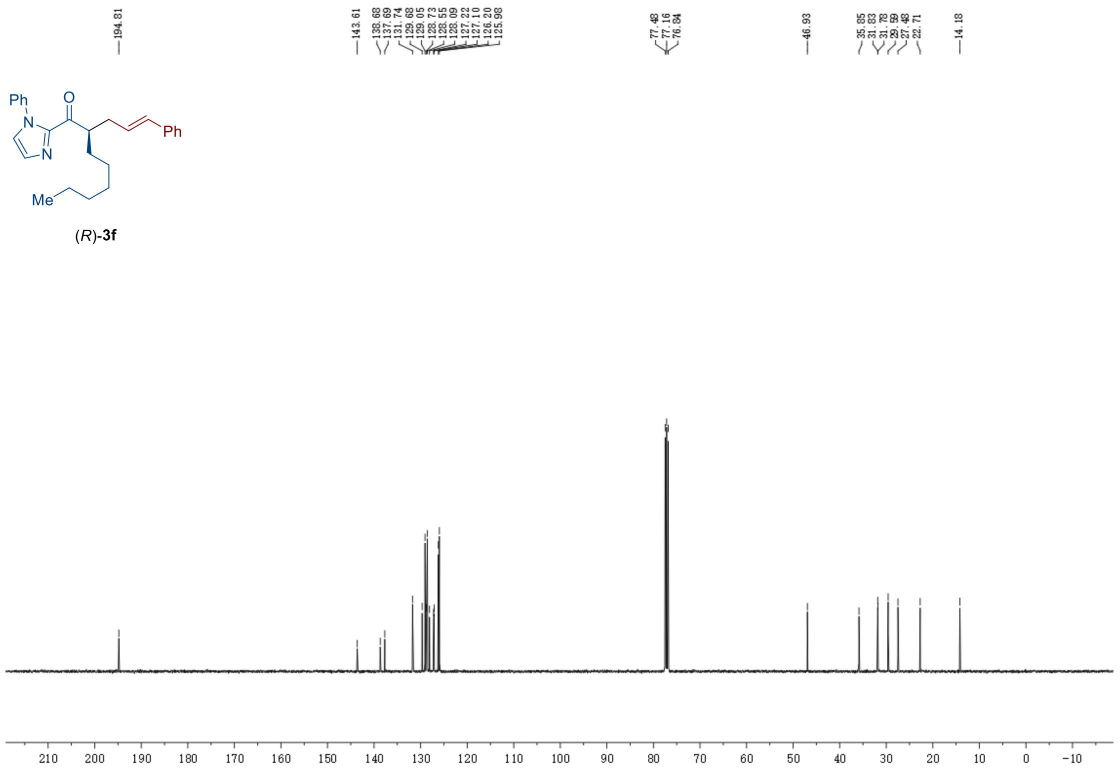

Supplementary Figure 8. NMR spectra of compound **3f**

^{1}H NMR spectrum of **3g**

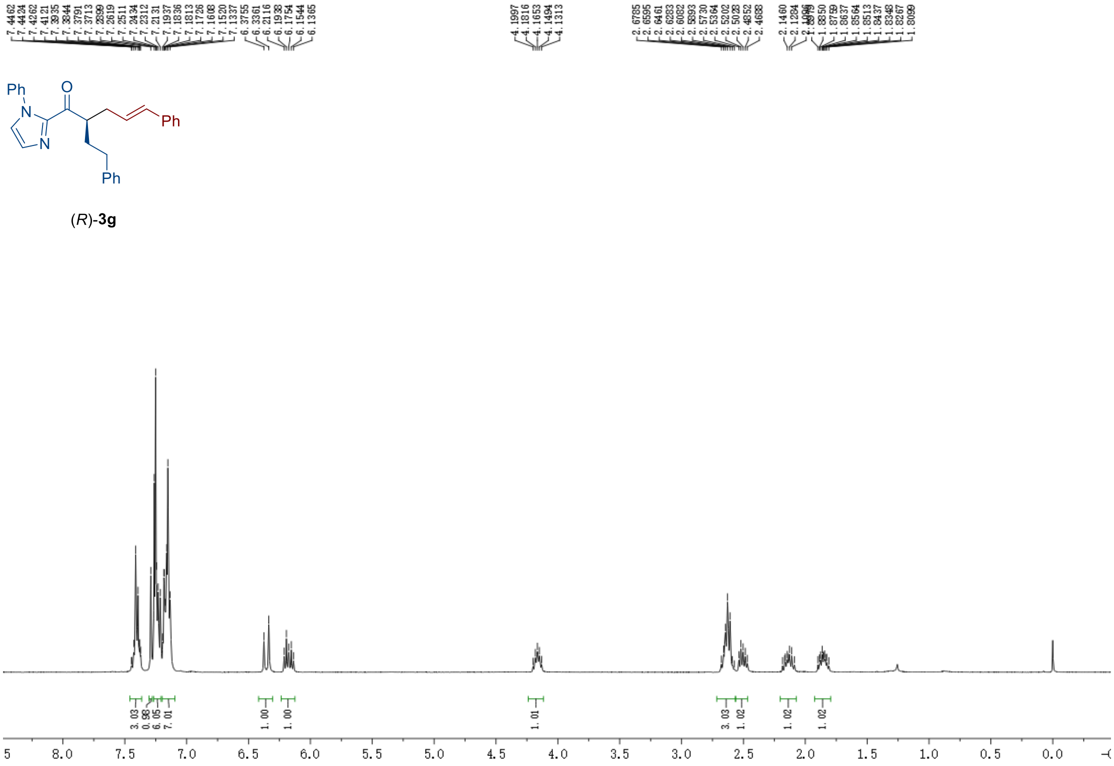

^{13}C NMR spectrum of **3g**

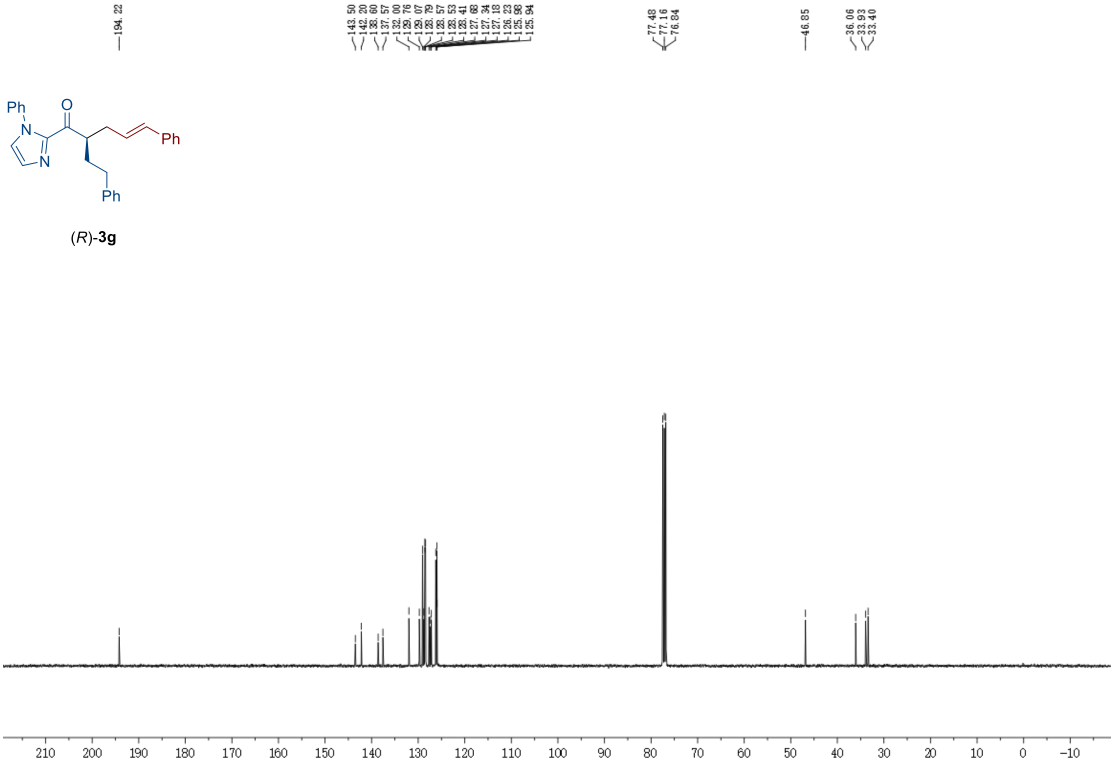

Supplementary Figure 9. NMR spectra of compound **3g**

^{1}H NMR spectrum of **3h**

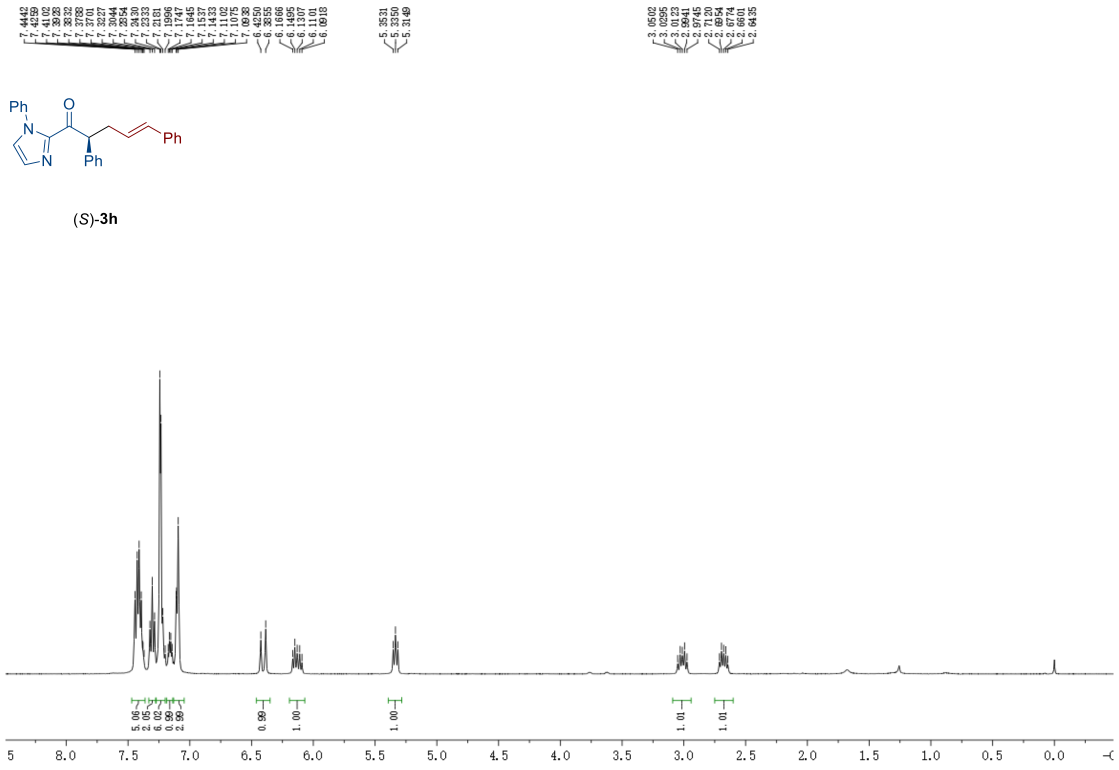

^{13}C NMR spectrum of **3h**

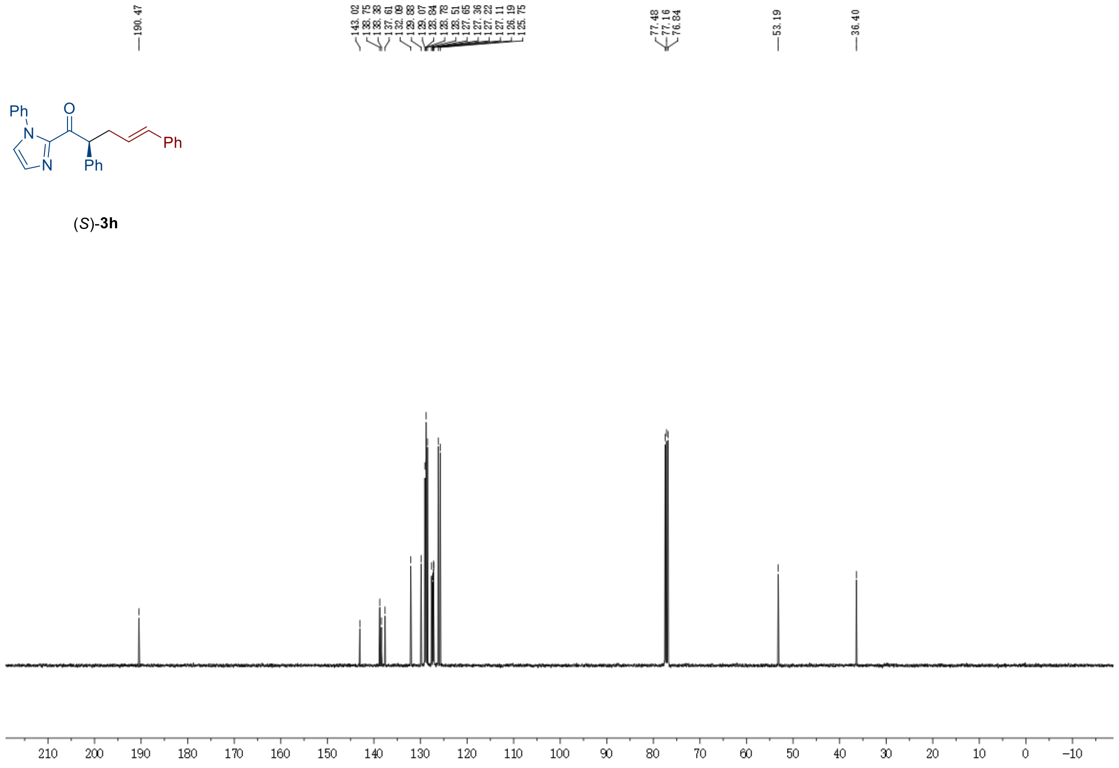

Supplementary Figure 10. NMR spectra of compound **3h**

^{1}H NMR spectrum of **3i**

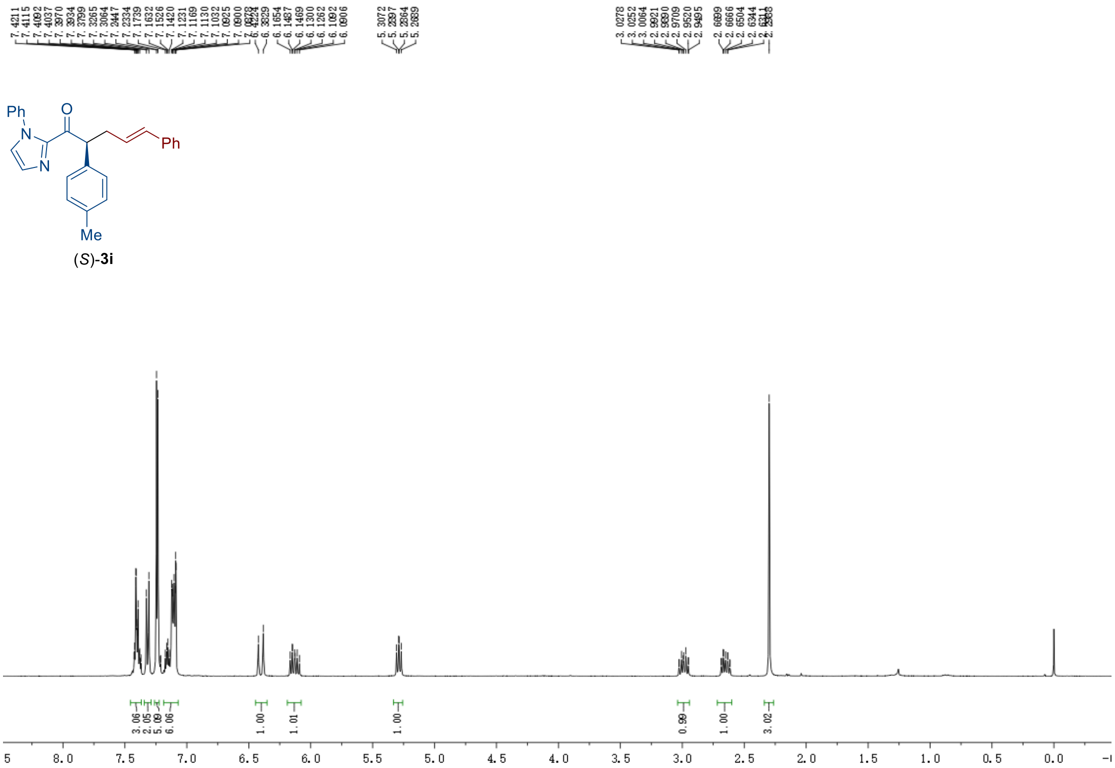

^{13}C NMR spectrum of **3i**

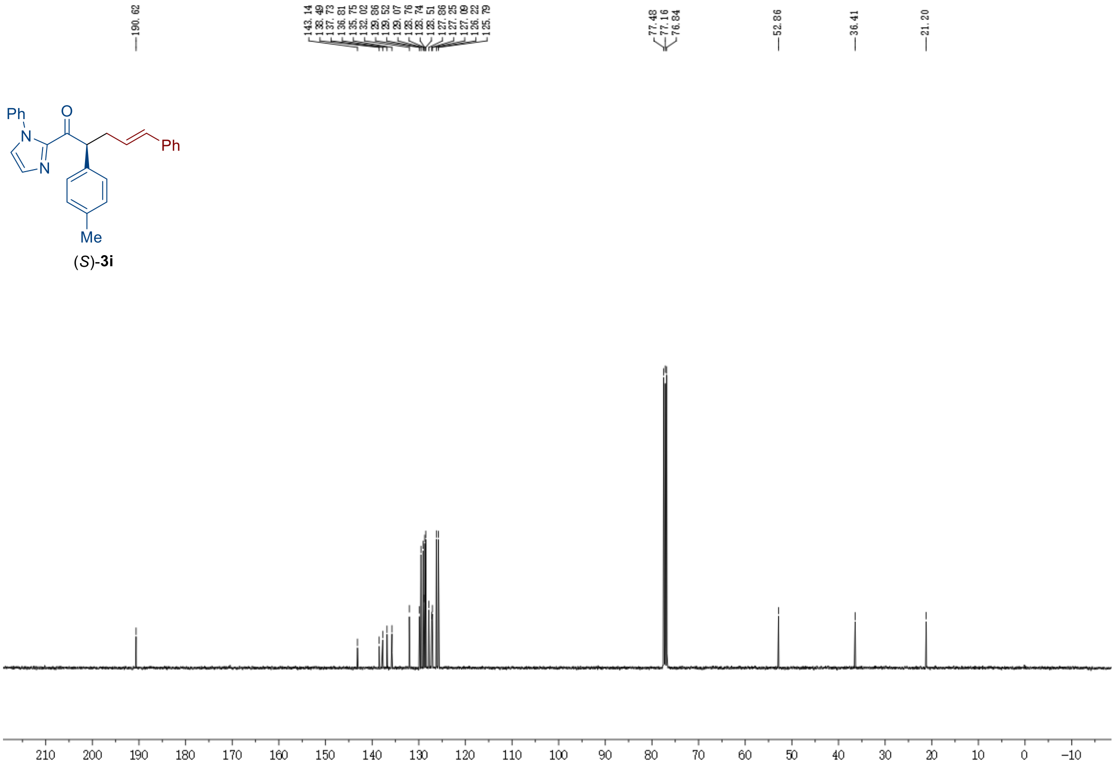

Supplementary Figure 11. NMR spectra of compound **3i**

^{1}H NMR spectrum of **3j**

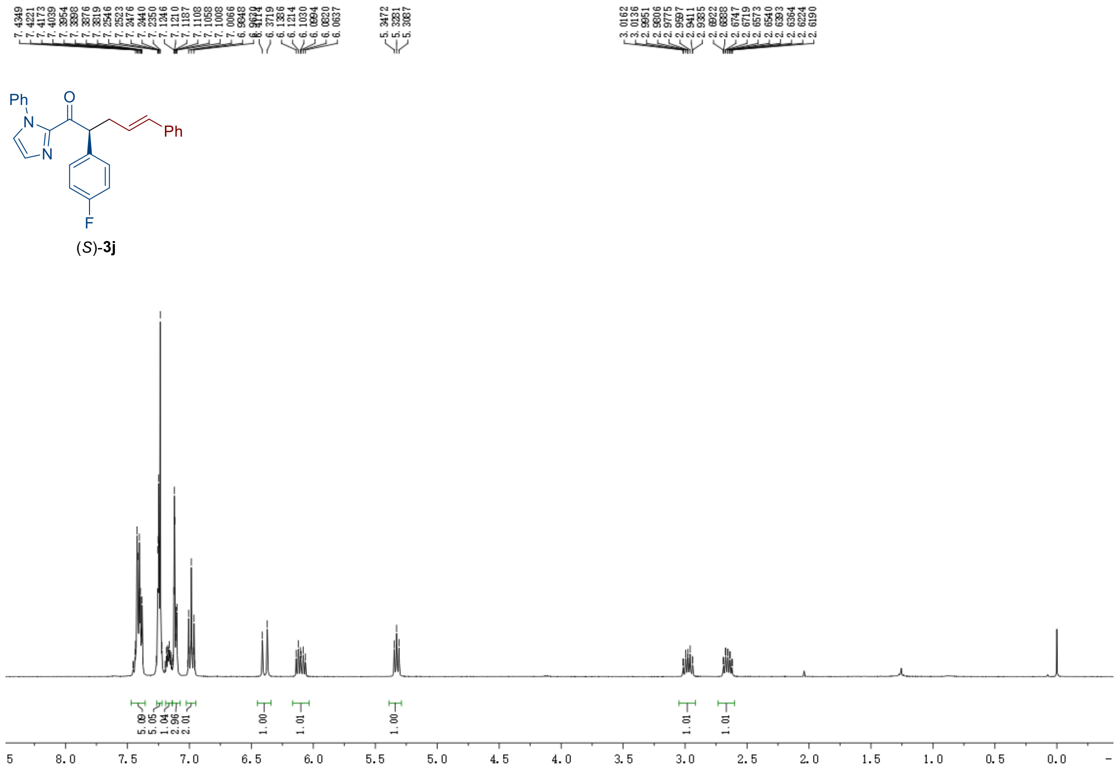

^{13}C NMR spectrum of **3j**

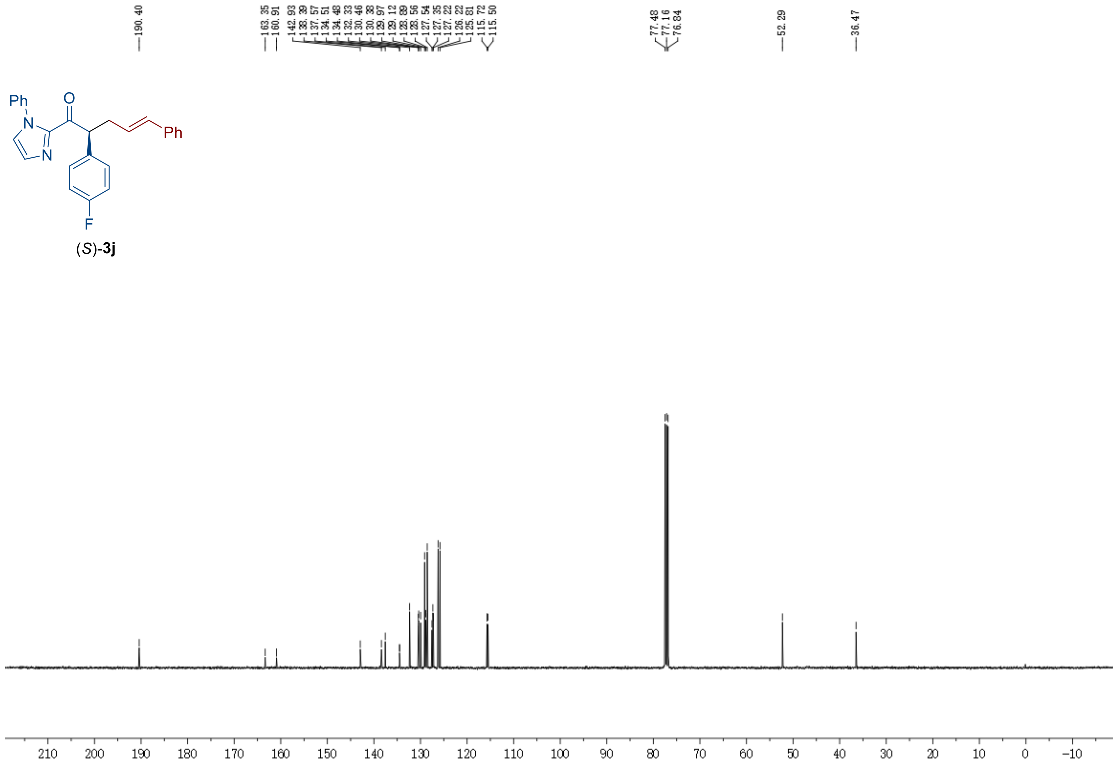

^{19}F NMR spectrum of **3j**

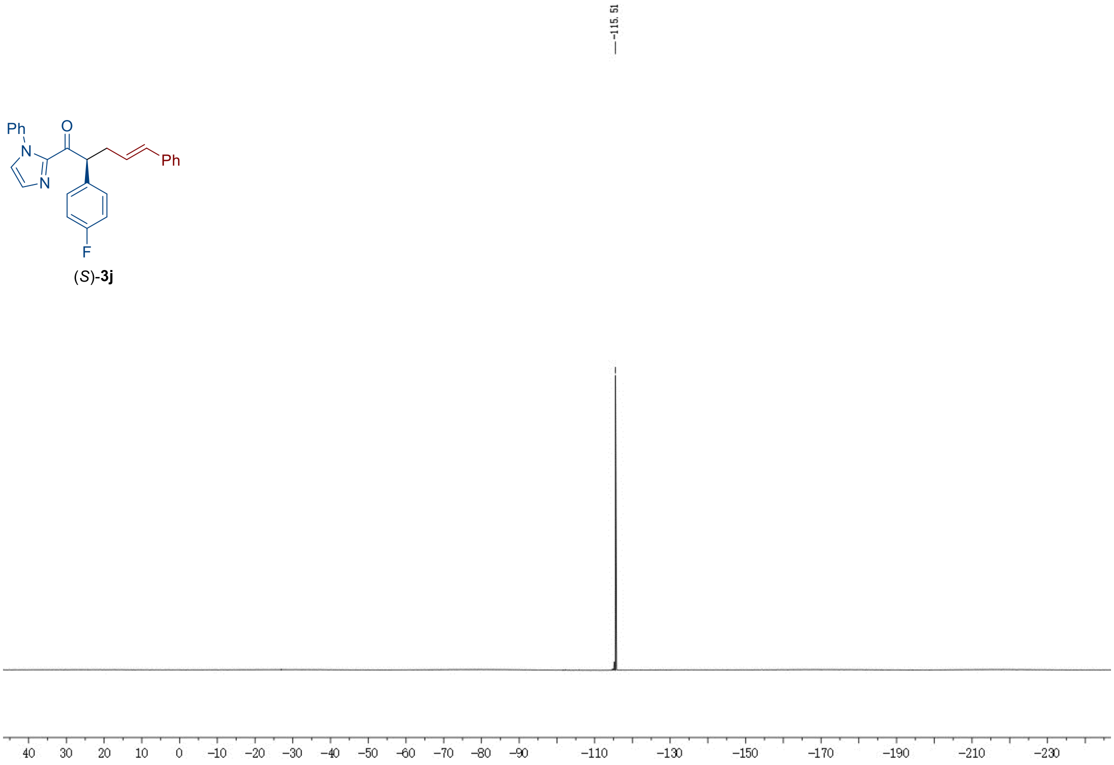

Supplementary Figure 12. NMR spectra of compound **3j**

^{1}H NMR spectrum of **3k**

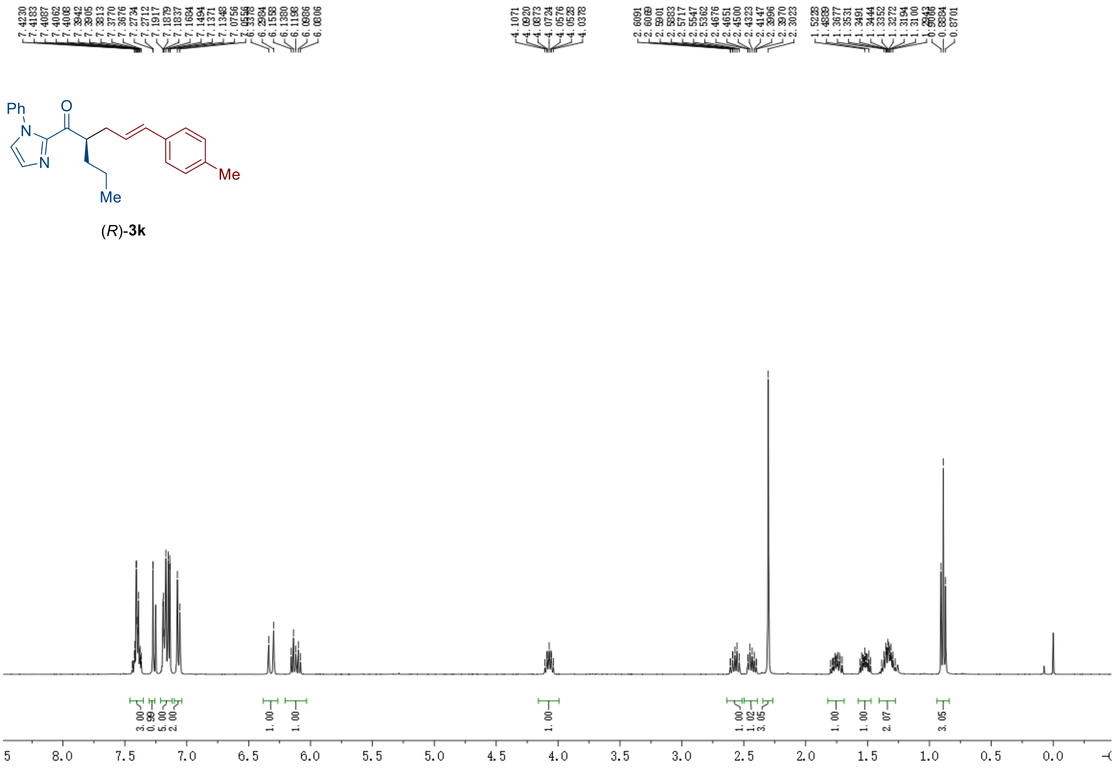

^{13}C NMR spectrum of **3k**

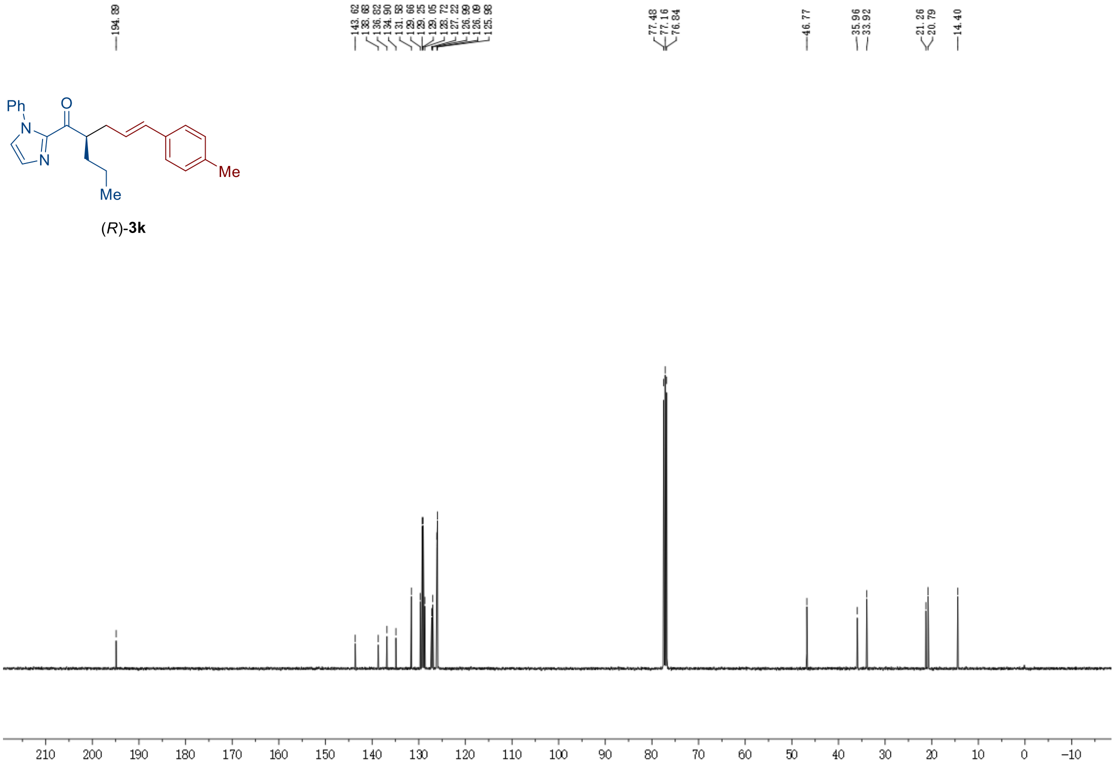

Supplementary Figure 13. NMR spectra of compound **3k**

^{1}H NMR spectrum of **3l**

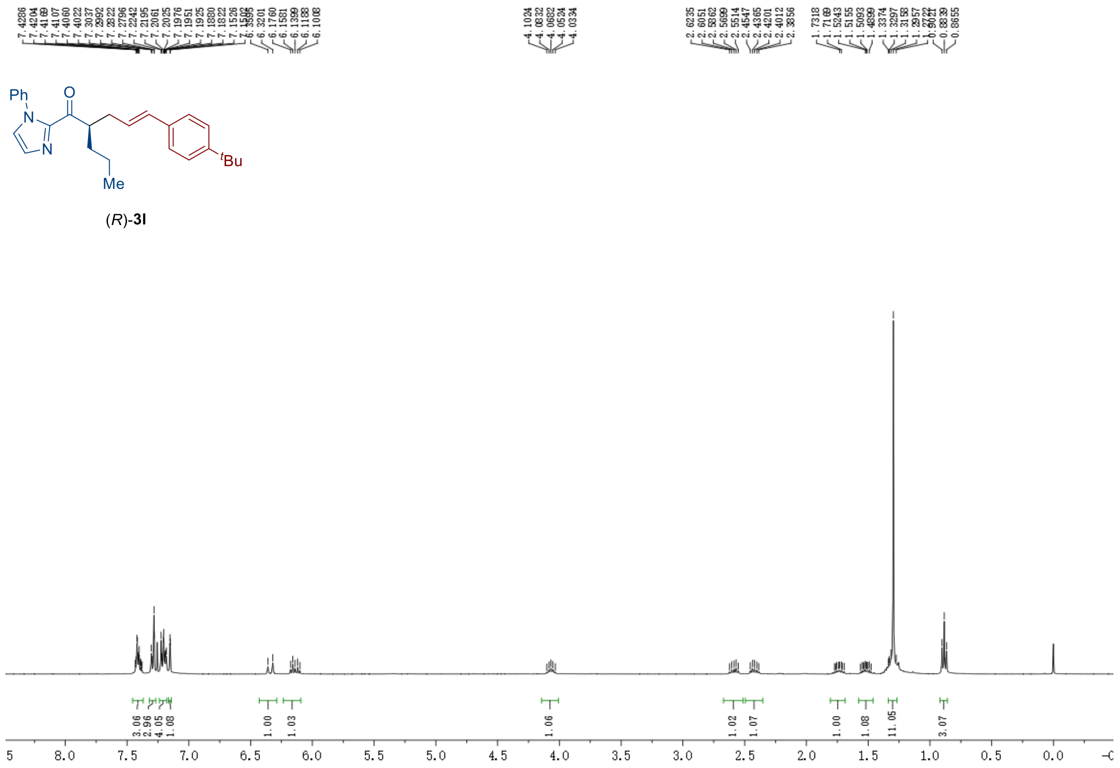

^{13}C NMR spectrum of **3l**

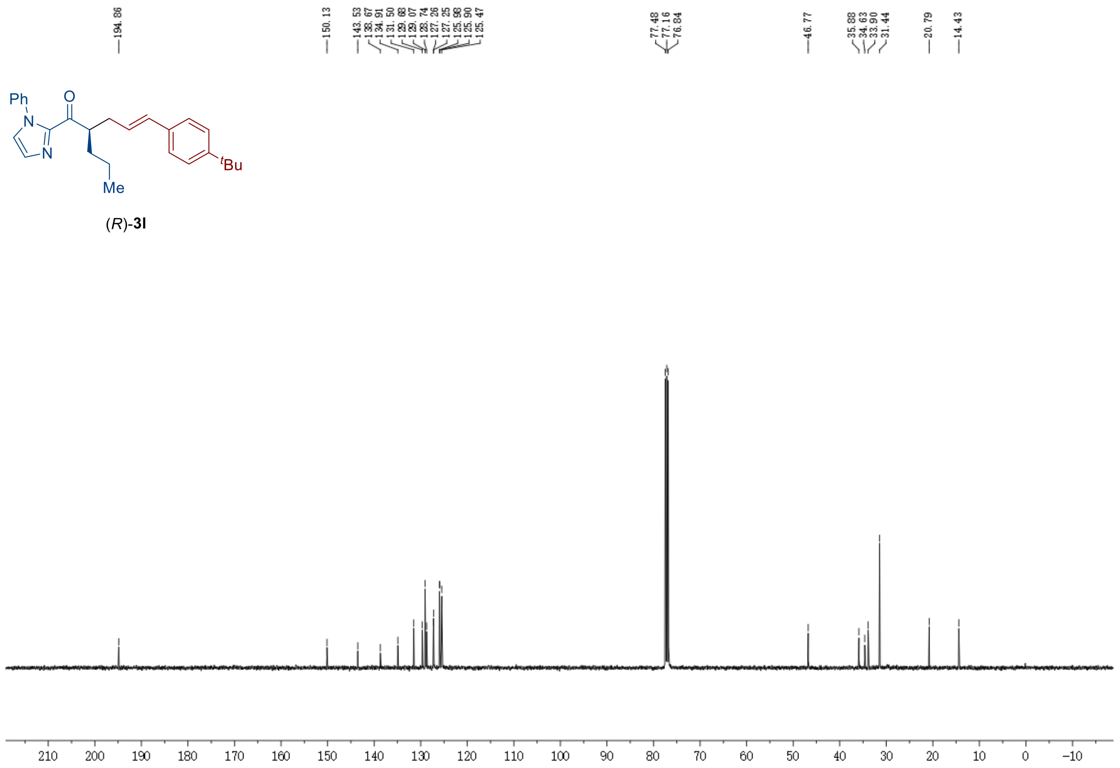

Supplementary Figure 14. NMR spectra of compound **3l**

^{1}H NMR spectrum of **3m**

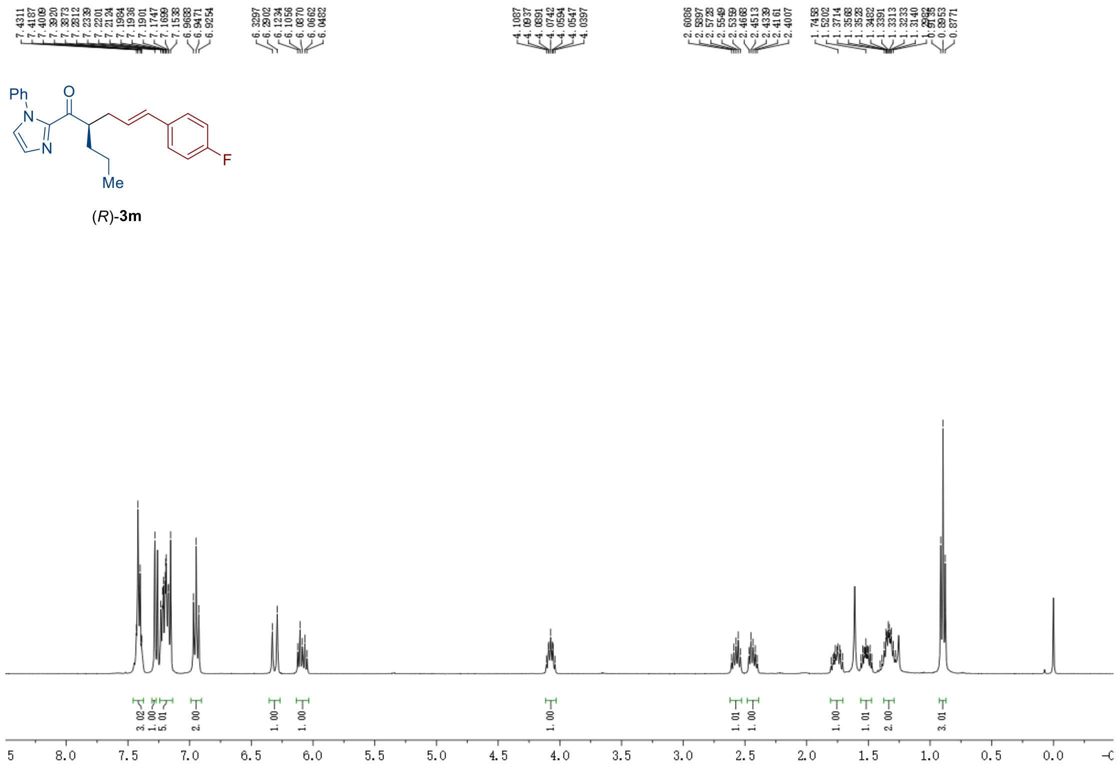

^{13}C NMR spectrum of **3m**

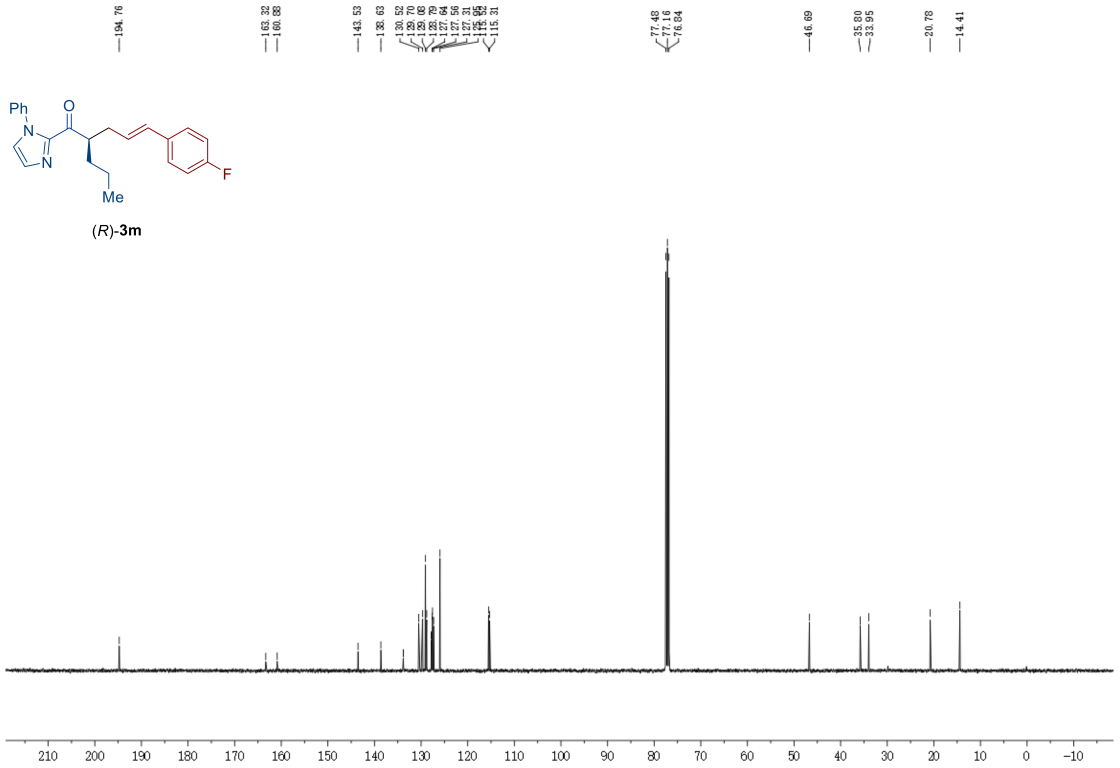

^{19}F NMR spectrum of **3m**

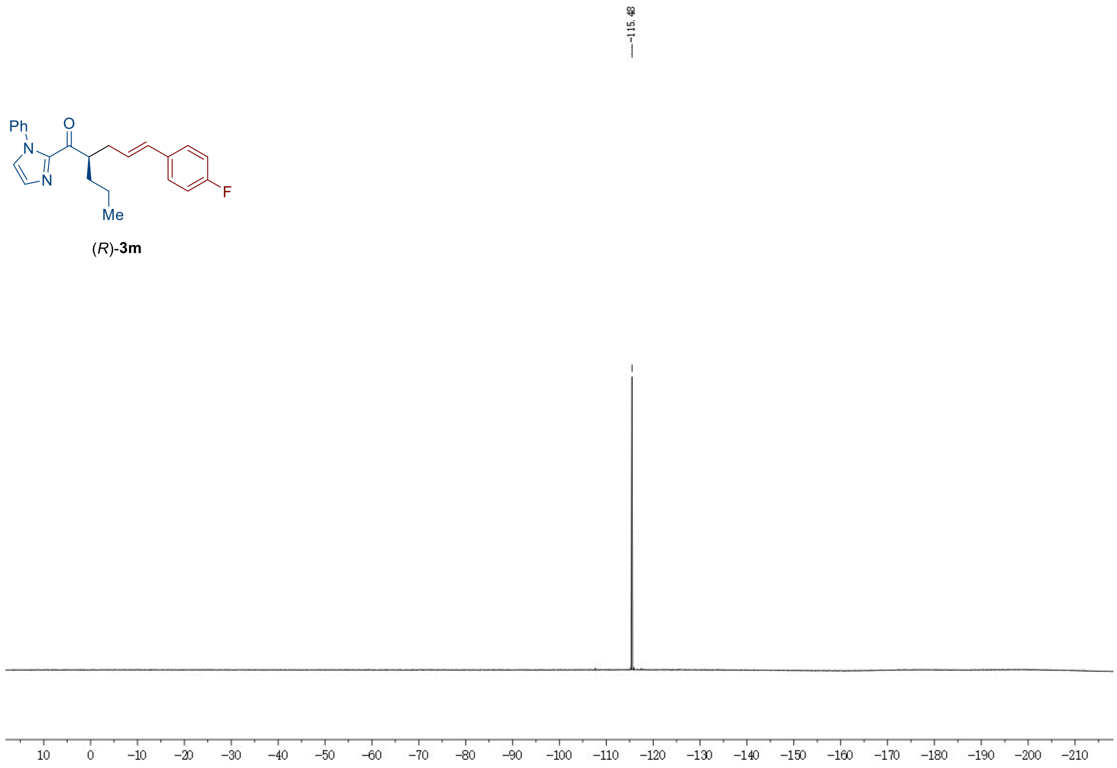

Supplementary Figure 15. NMR spectra of compound **3m**

^{1}H NMR spectrum of **3n**

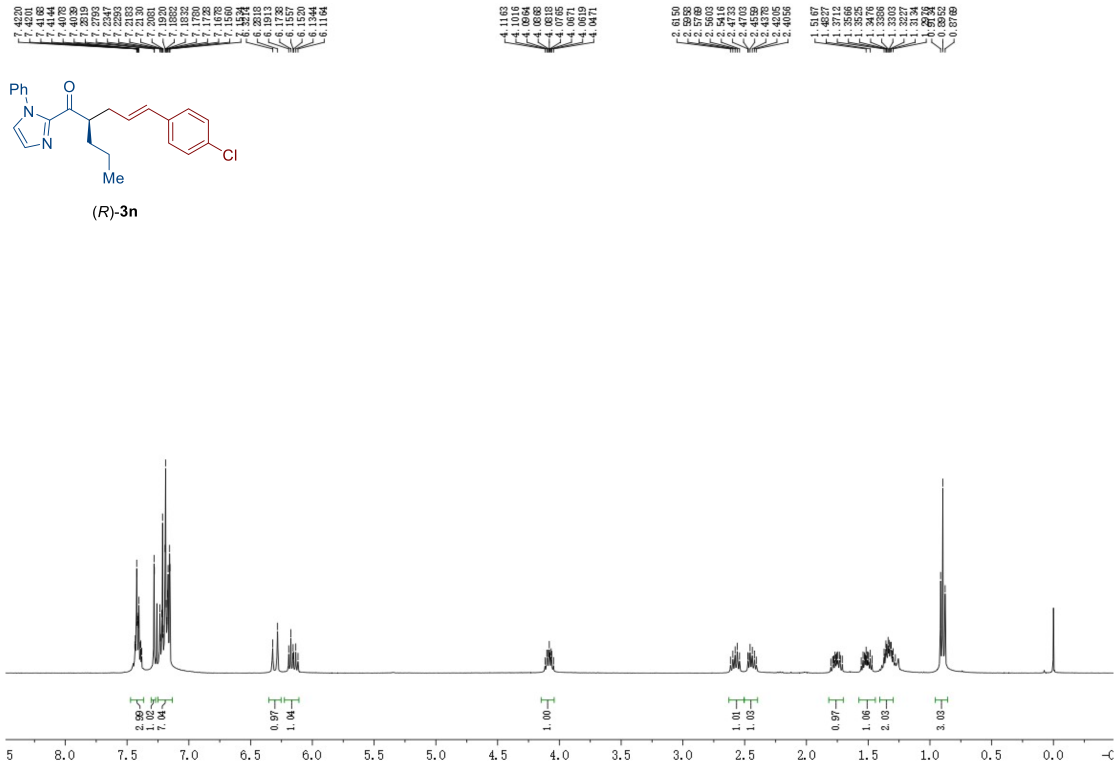

^{13}C NMR spectrum of **3n**

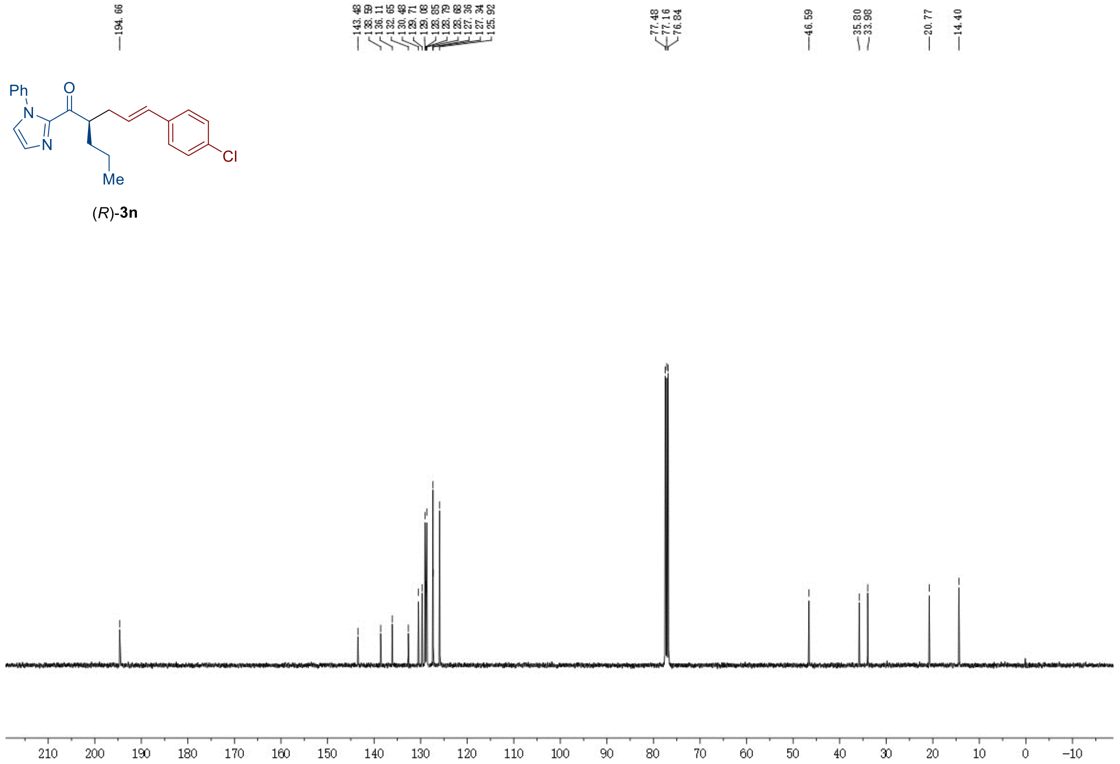

Supplementary Figure 16. NMR spectra of compound **3n**

^{1}H NMR spectrum of **3o**

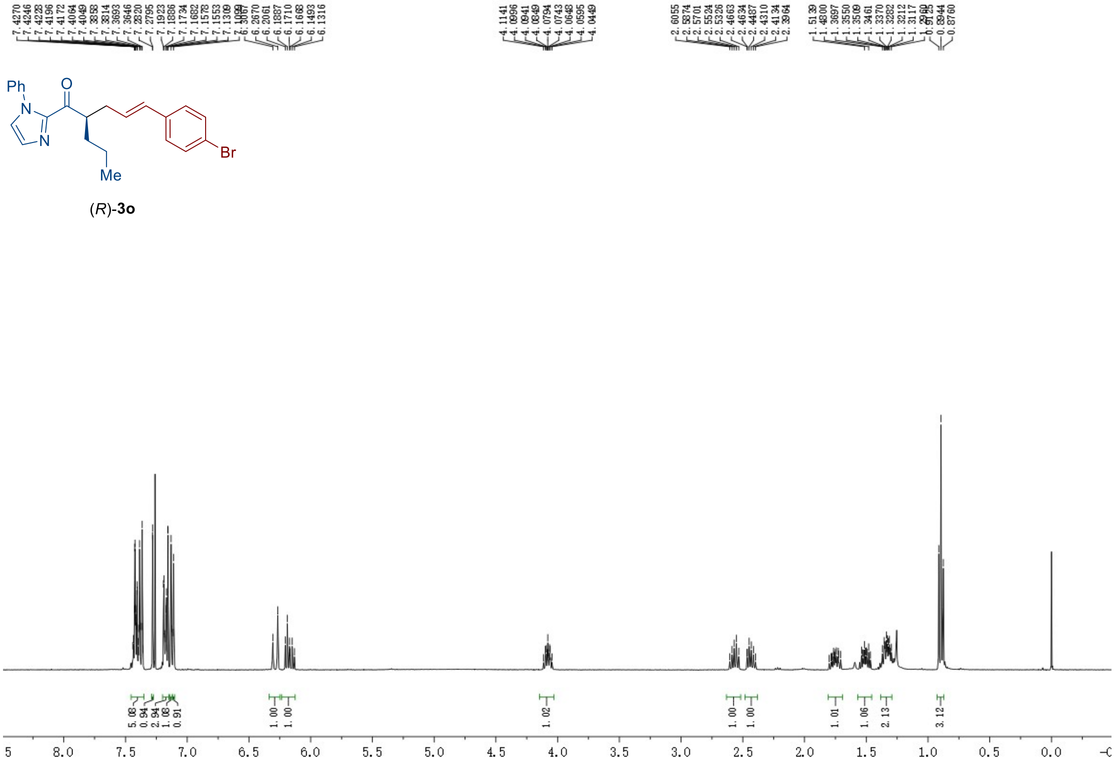

^{13}C NMR spectrum of **3o**

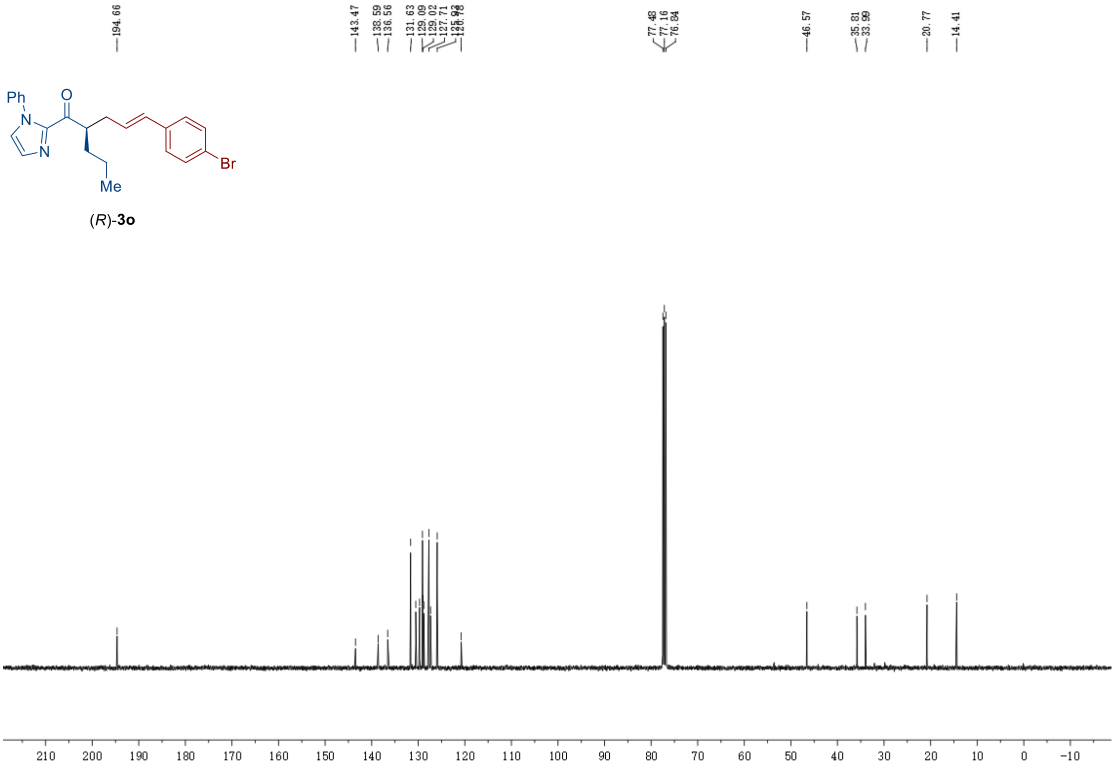

Supplementary Figure 17. NMR spectra of compound **3o**

^{1}H NMR spectrum of **3p**

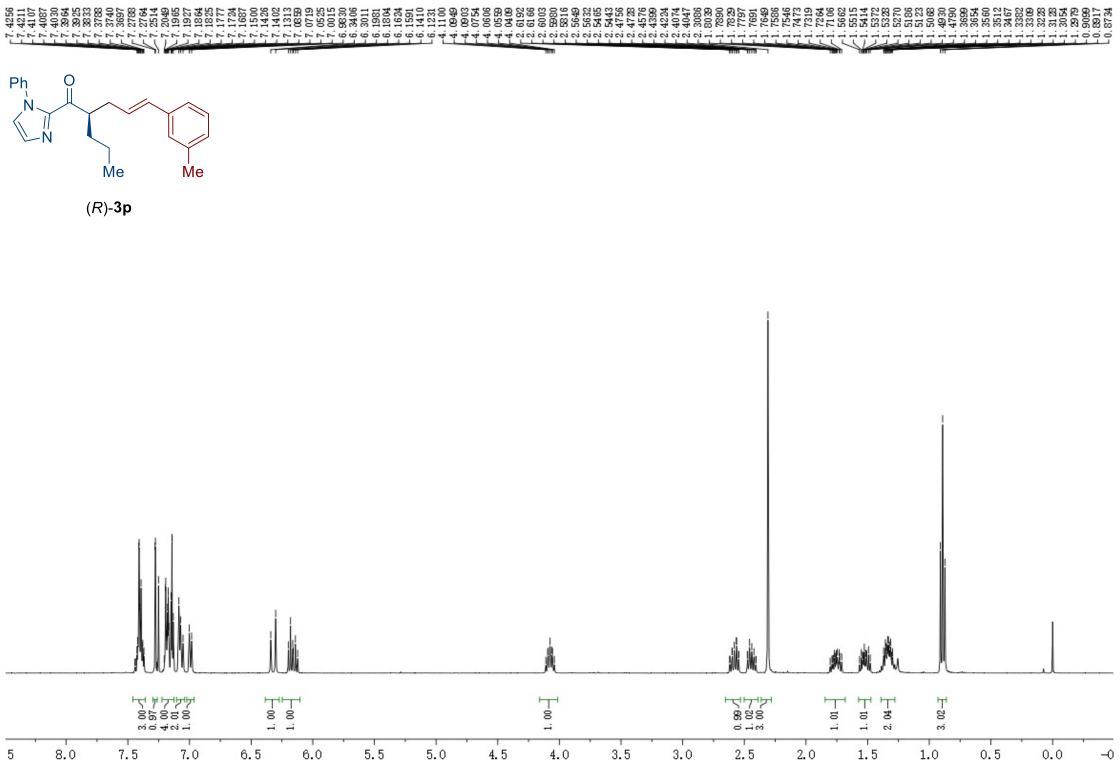

^{13}C NMR spectrum of **3p**

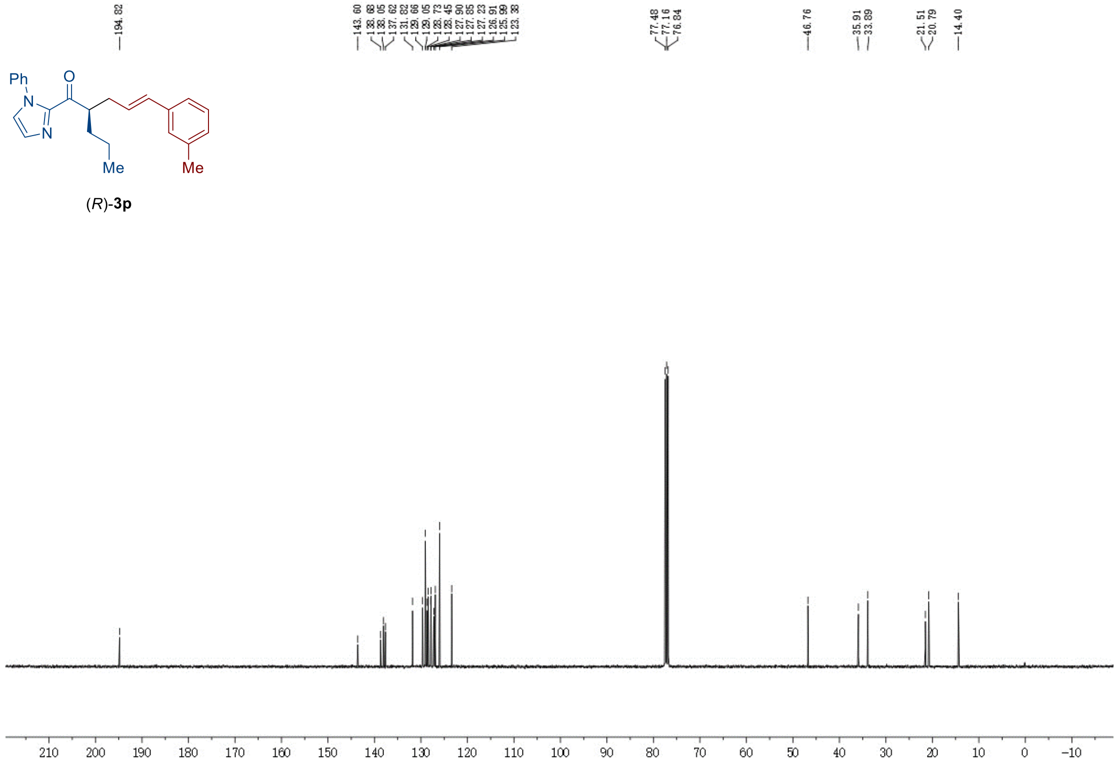

Supplementary Figure 18. NMR spectra of compound **3p**

^{1}H NMR spectrum of **3q**

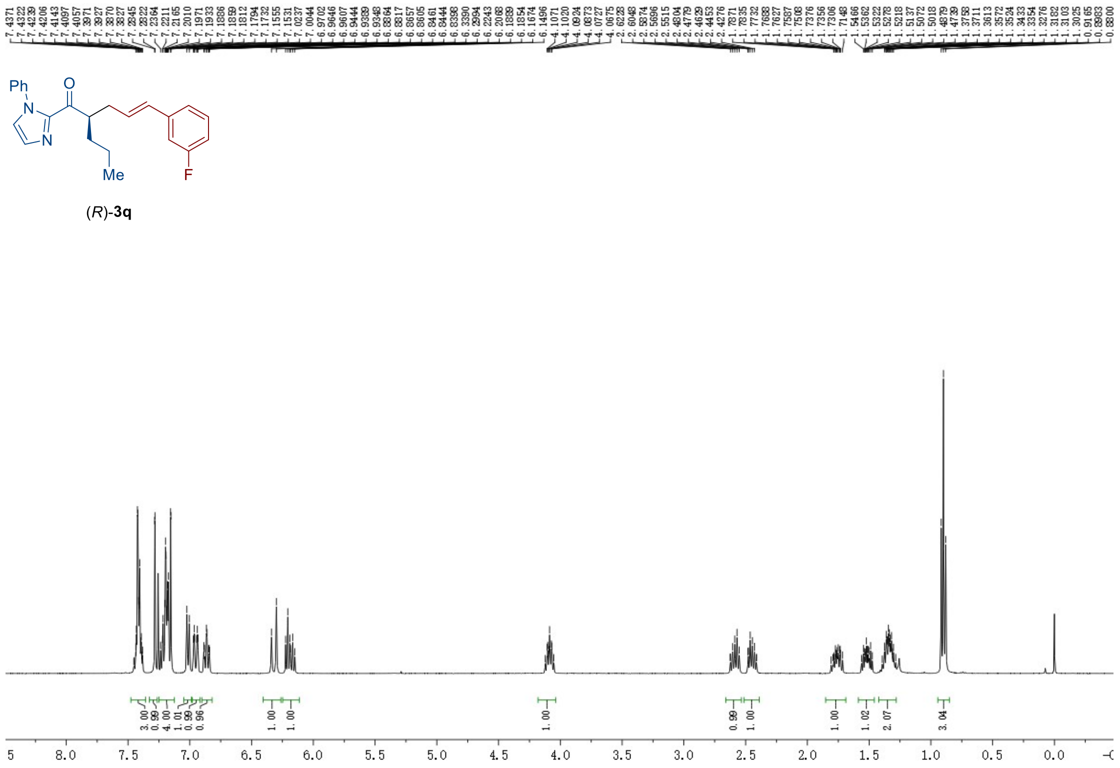

^{13}C NMR spectrum of **3q**

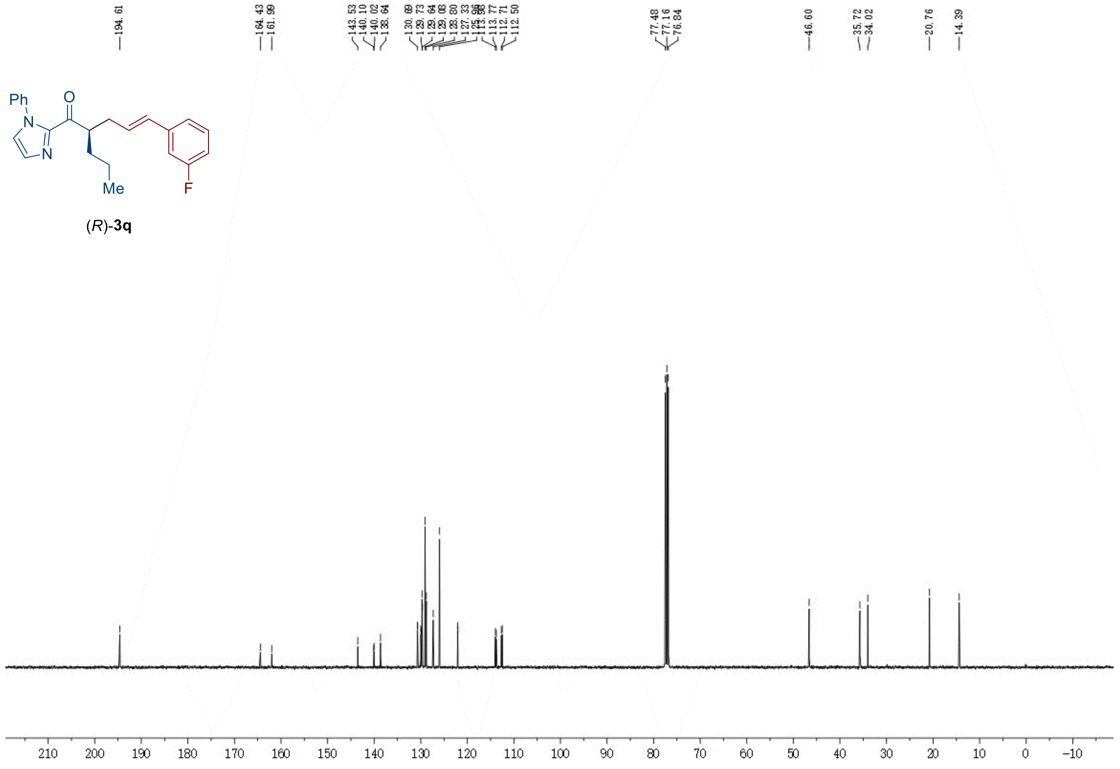

^{19}F NMR spectrum of **3q**

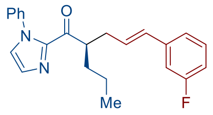

(*R*)-**3q**

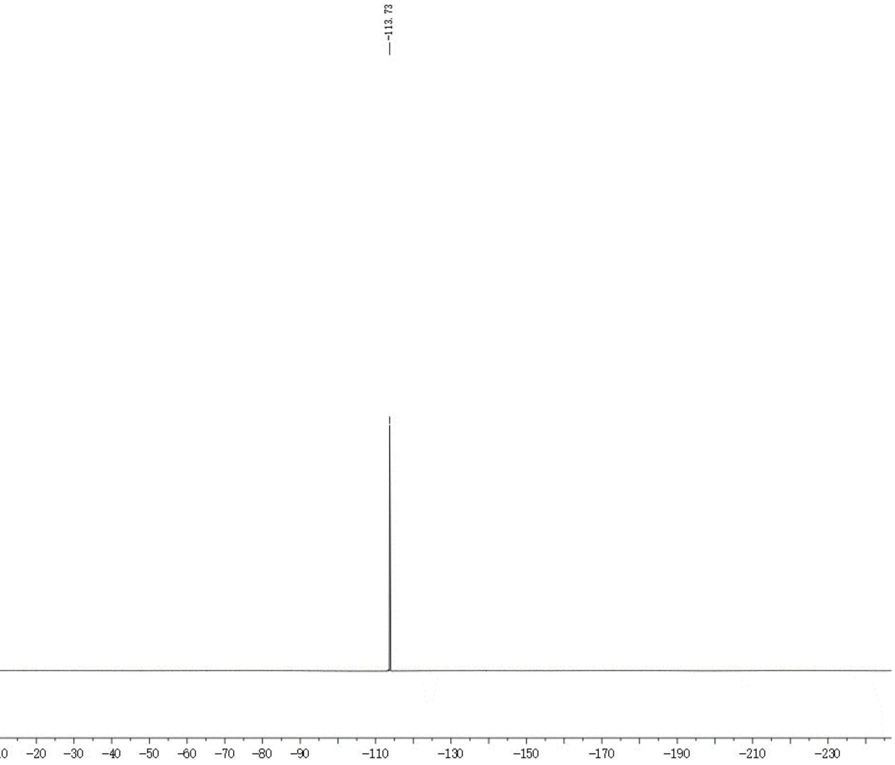

Supplementary Figure 19. NMR spectra of compound **3q**

^{1}H NMR spectrum of **3r**

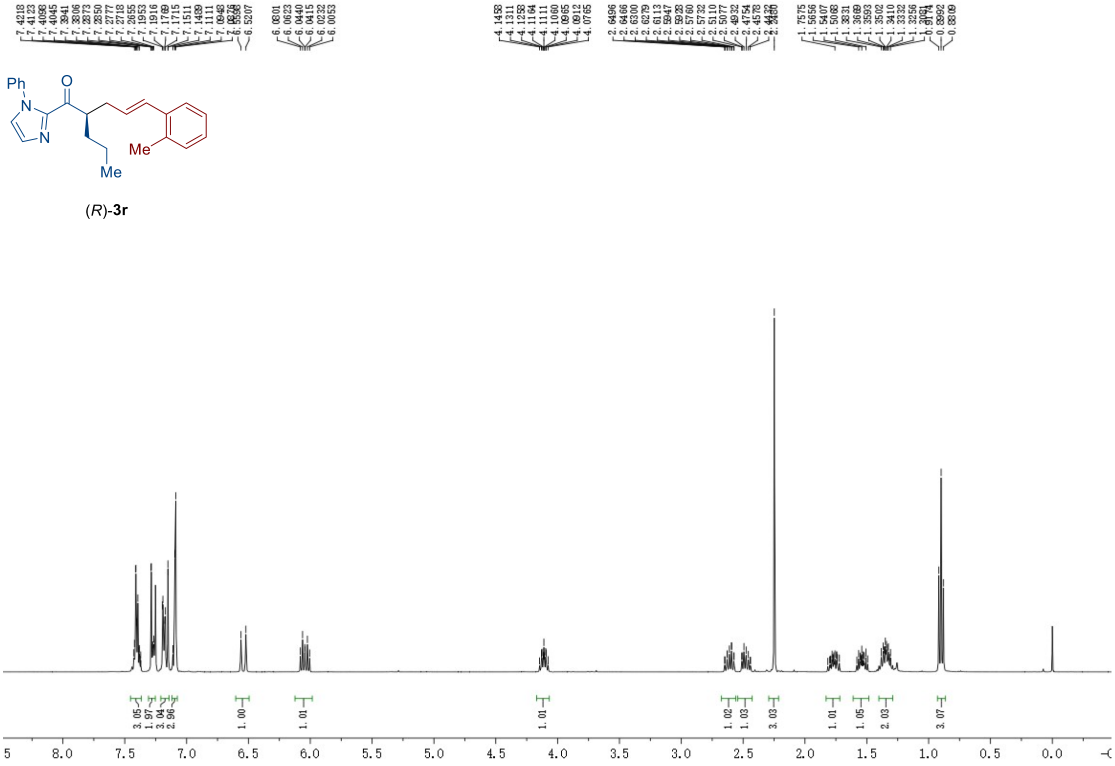

^{13}C NMR spectrum of **3r**

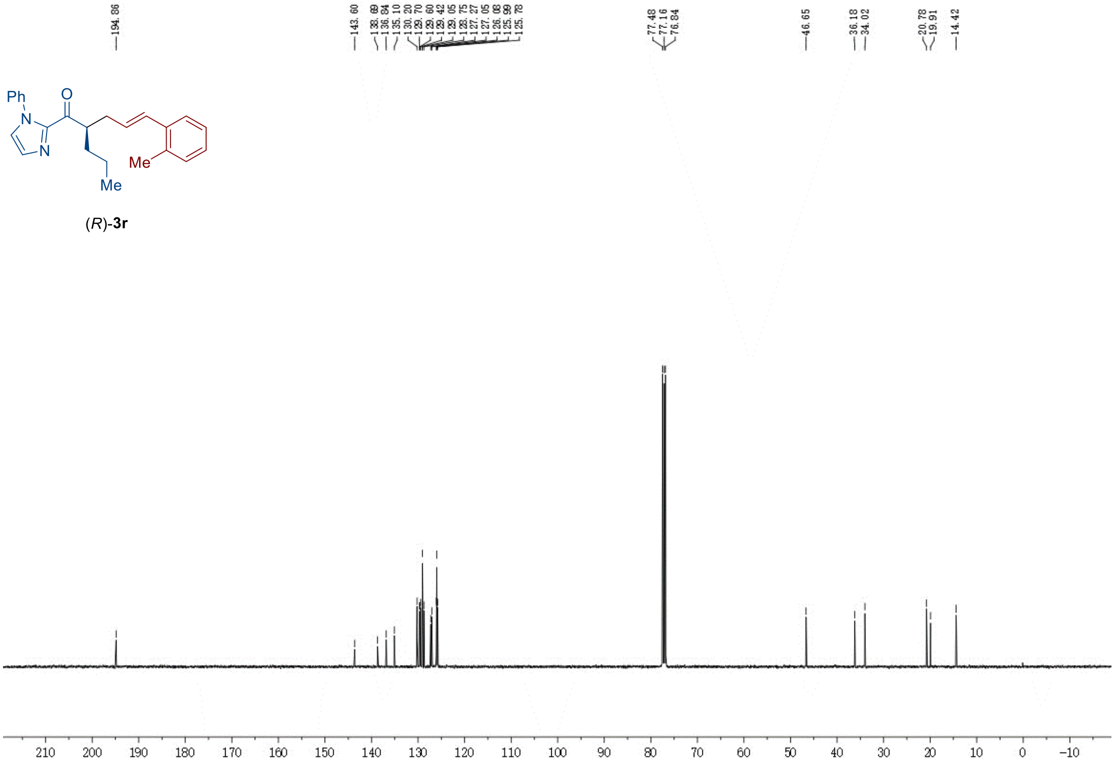

Supplementary Figure 20. NMR spectra of compound **3r**

^{1}H NMR spectrum of **3s**

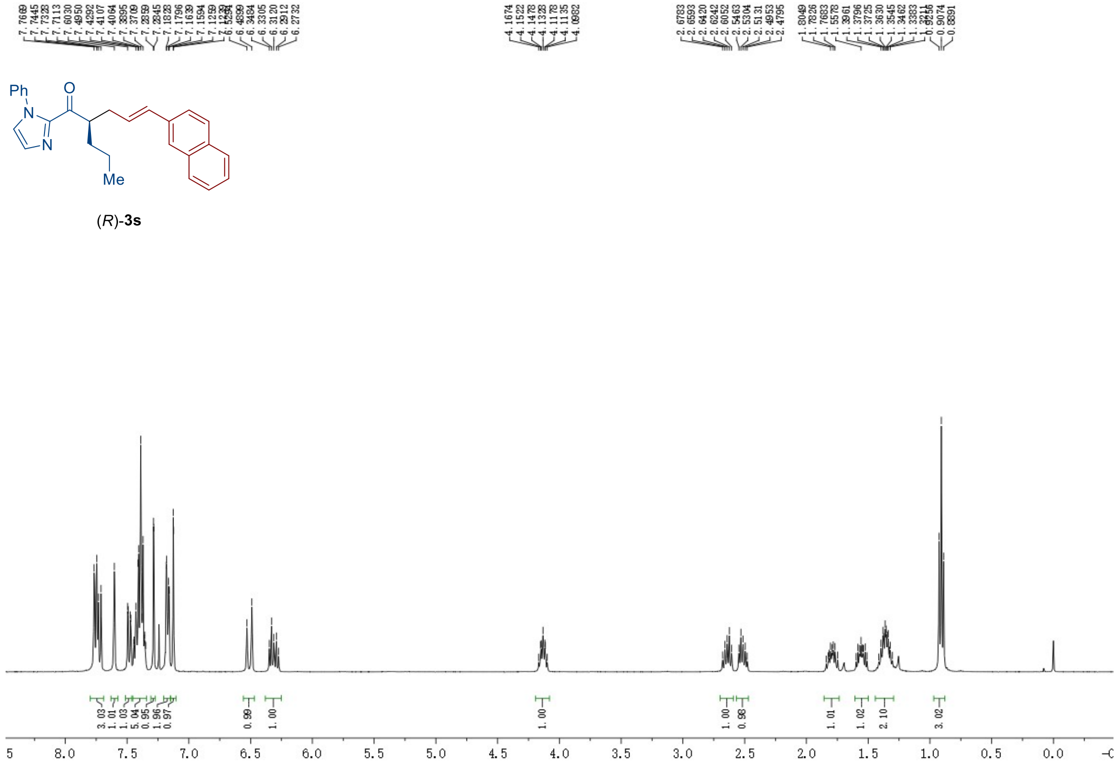

^{13}C NMR spectrum of **3s**

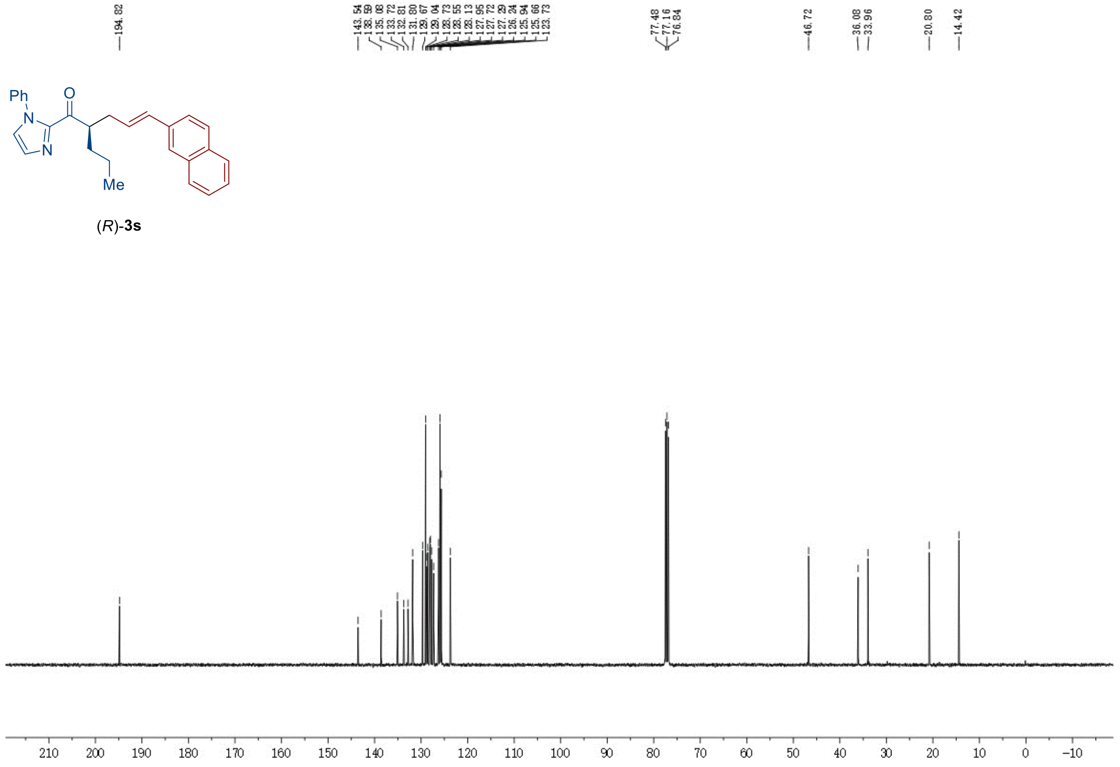

Supplementary Figure 21. NMR spectra of compound **3s**

^{1}H NMR spectrum of **3t**

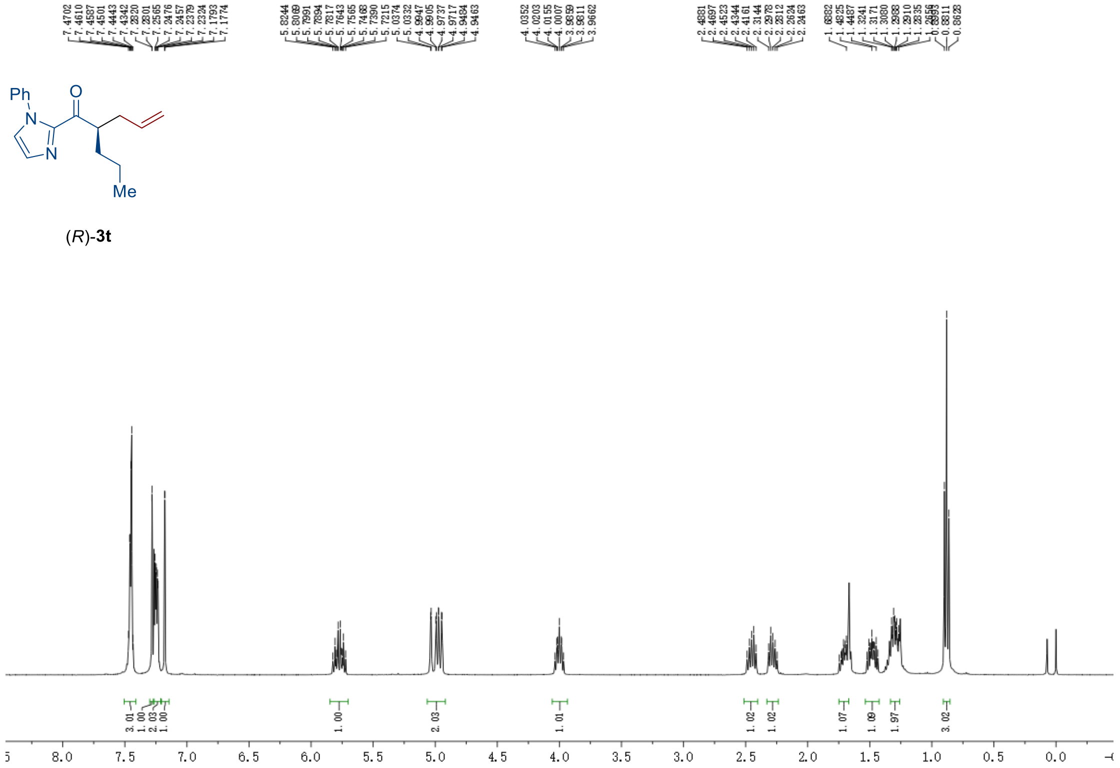

^{13}C NMR spectrum of **3t**

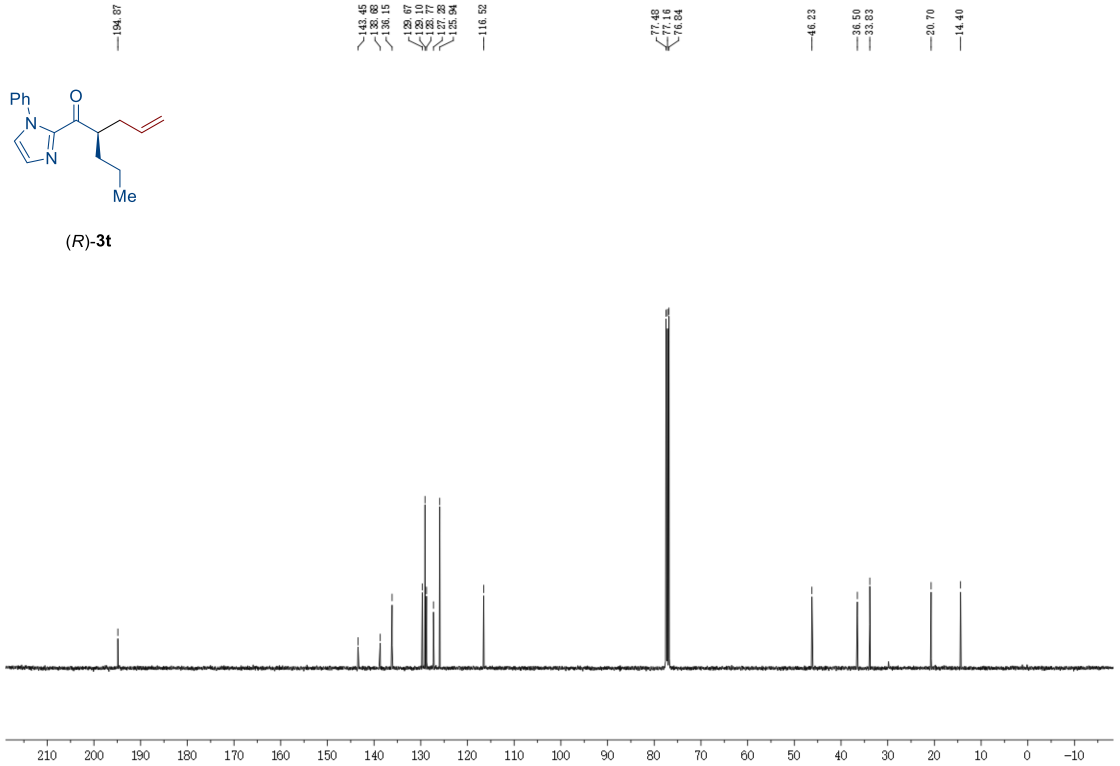

Supplementary Figure 22. NMR spectra of compound **3t**

^{1}H NMR spectrum of **3u**

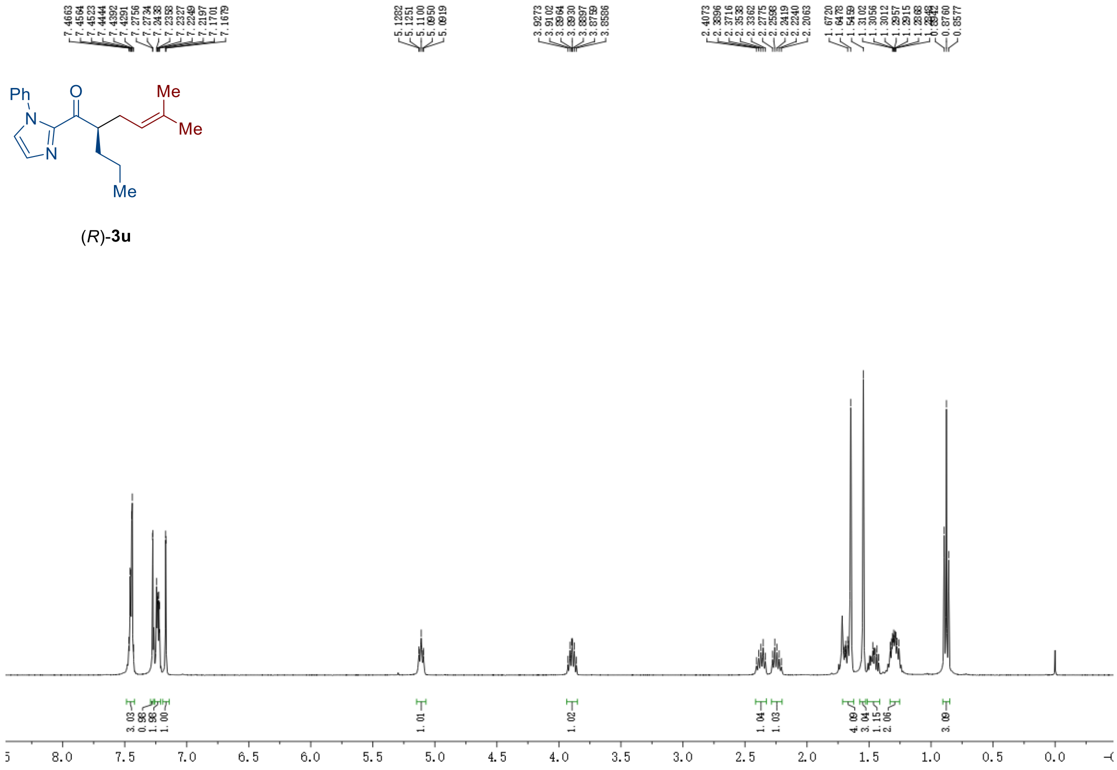

^{13}C NMR spectrum of **3u**

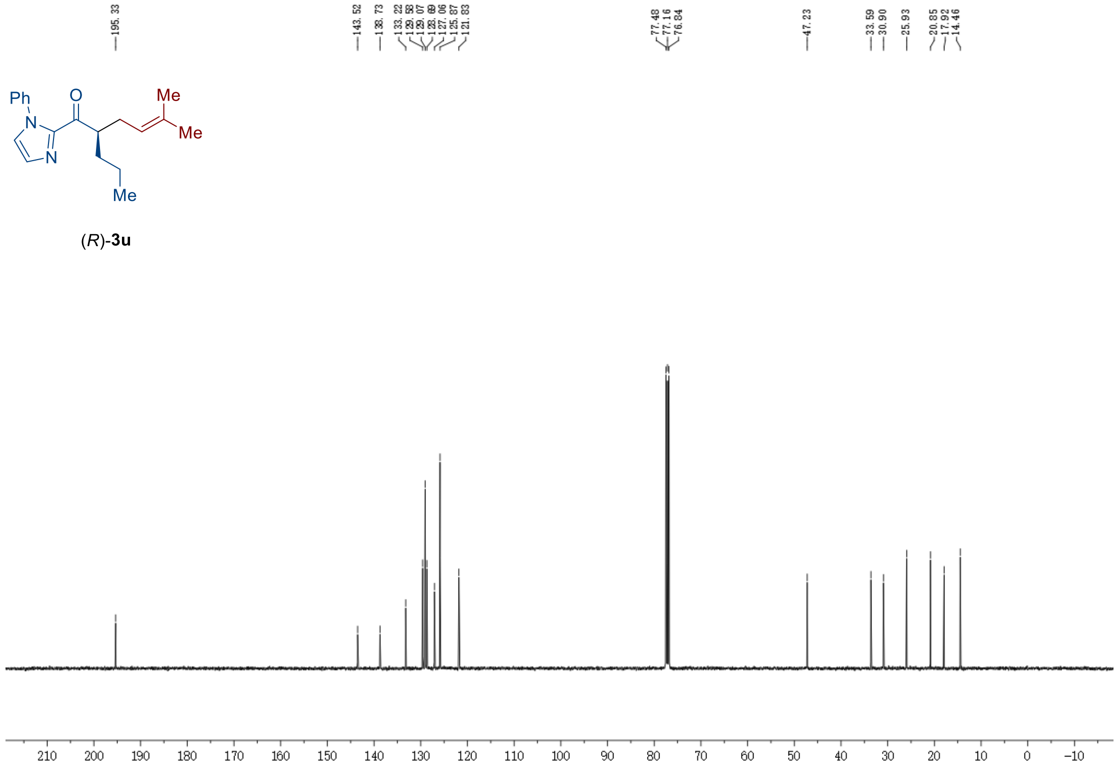

Supplementary Figure 23. NMR spectra of compound **3u**

^{1}H NMR spectrum of (*R*,*S*)-**4a**

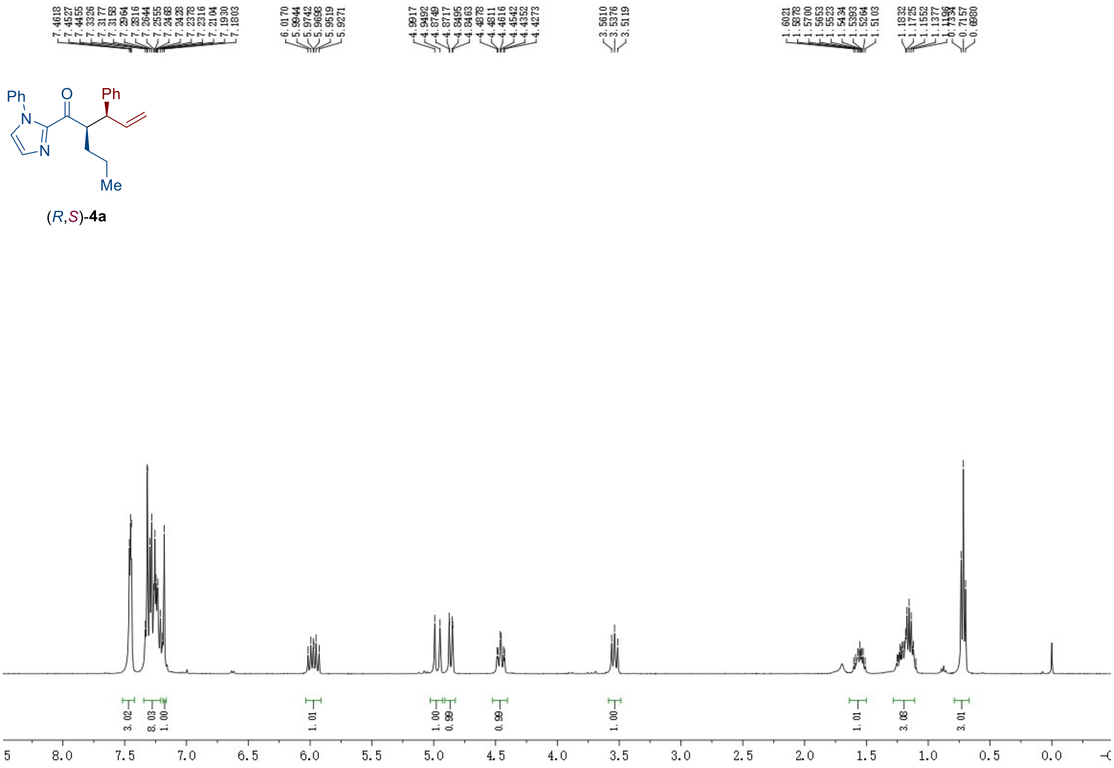

^{13}C NMR spectrum of (*R*,*S*)-**4a**

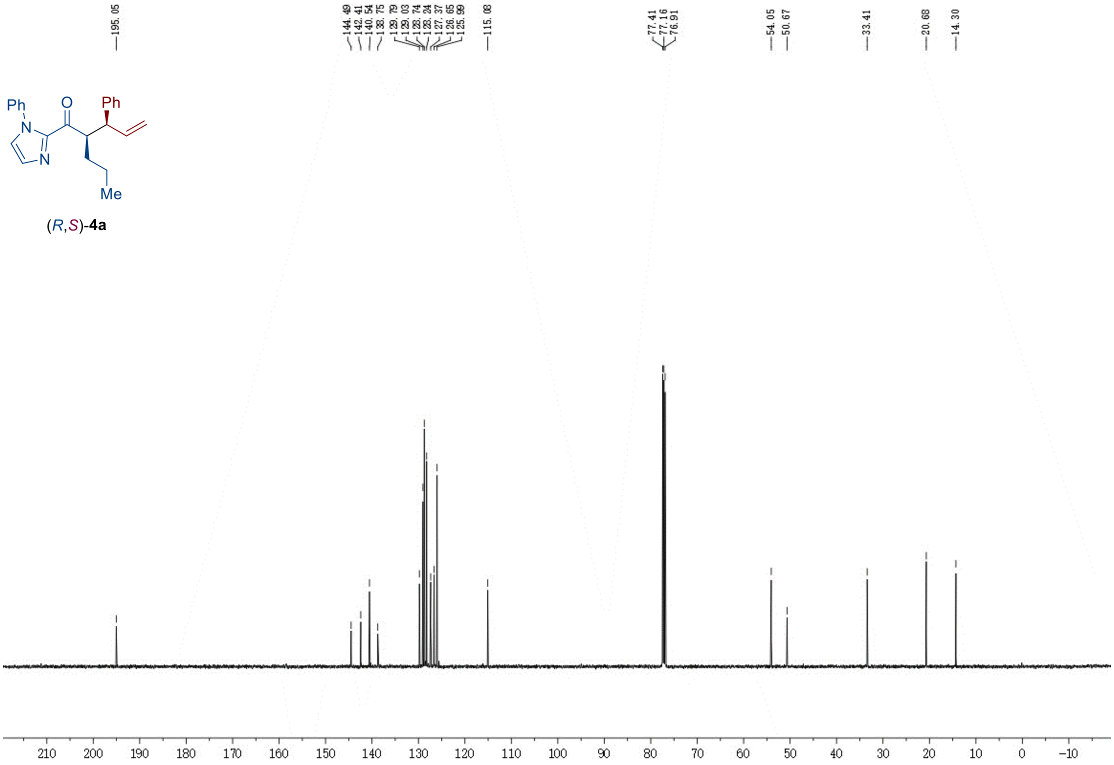

Supplementary Figure 24. NMR spectra of compound (*R*,*S*)-**4a**

^{1}H NMR spectrum of (*S*,*R*)**4a**

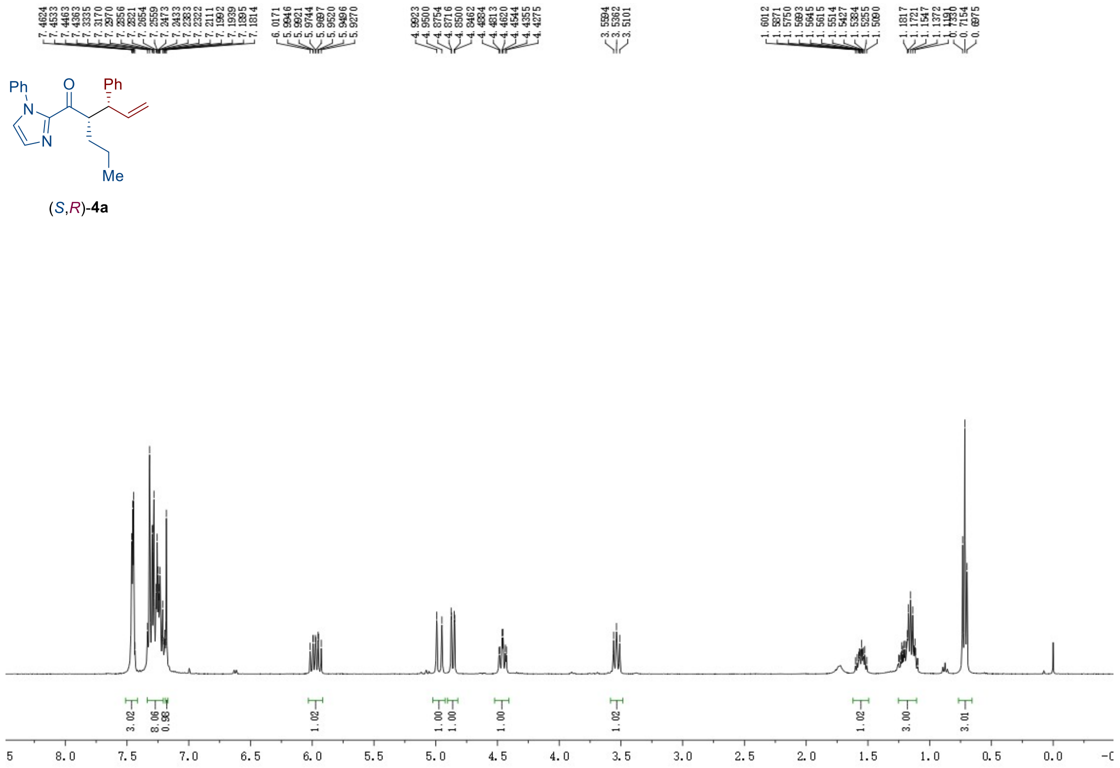

^{13}C NMR spectrum of (*S*,*R*)-**4a**

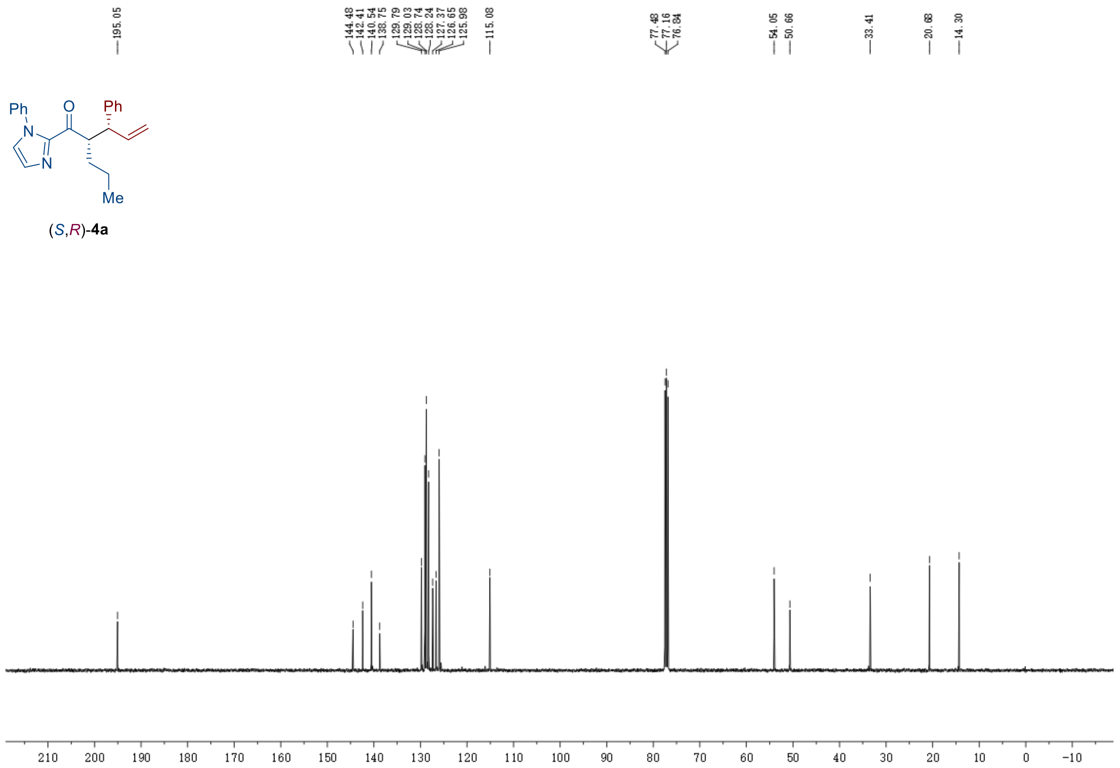

Supplementary Figure 25. NMR spectra of compound (*S*,*R*)-**4a**

^{1}H NMR spectrum of (*R*,*R*)-**4a**

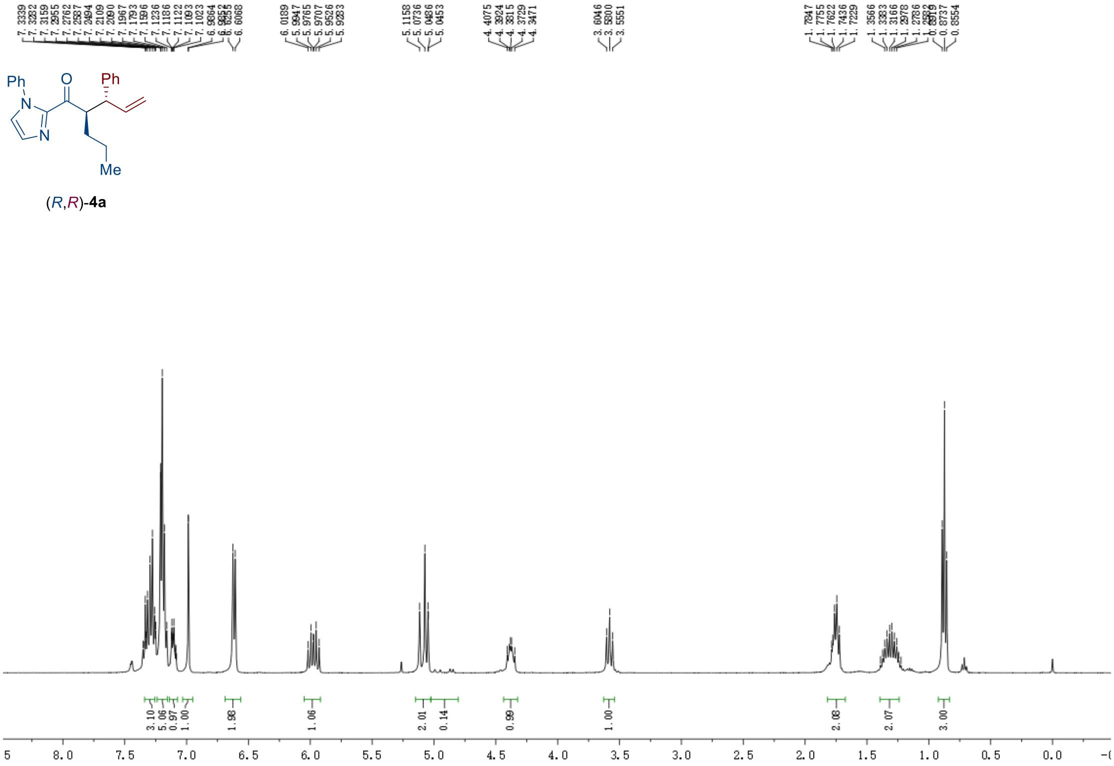

^{13}C NMR spectrum of (*R*,*R*)-**4a**

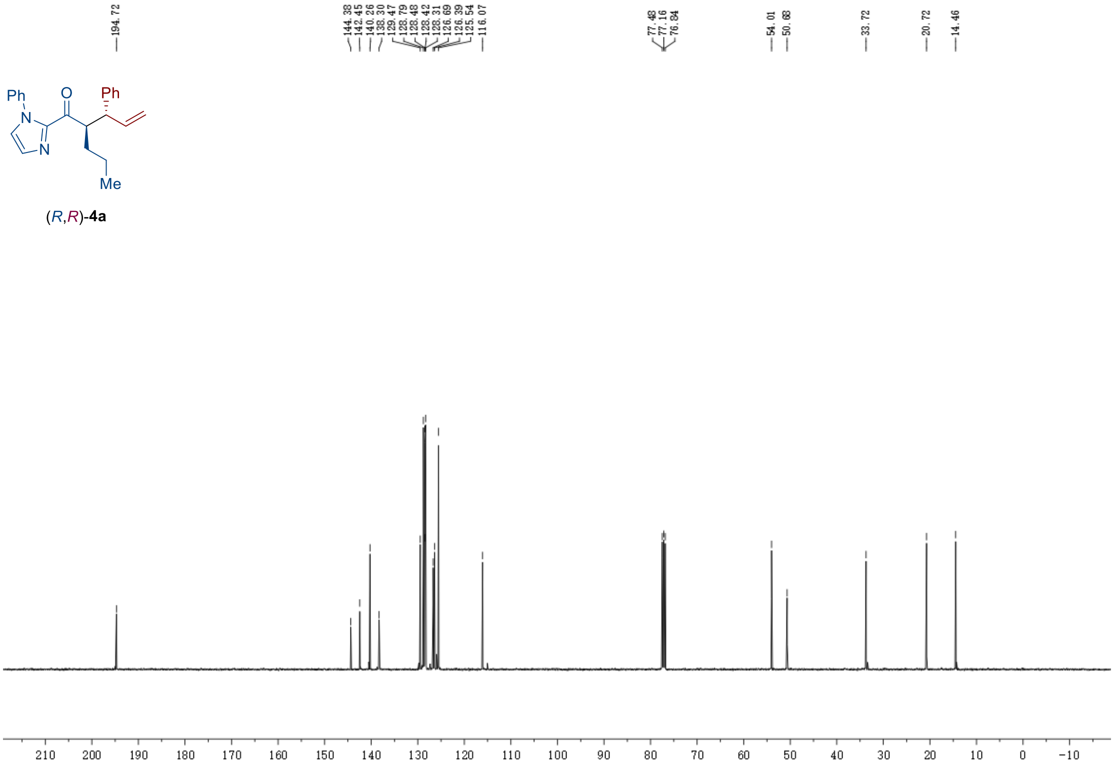

Supplementary Figure 26. NMR spectra of compound (*R*,*R*)-**4a**

^{1}H NMR spectrum of (*S*,*S*)-**4a**

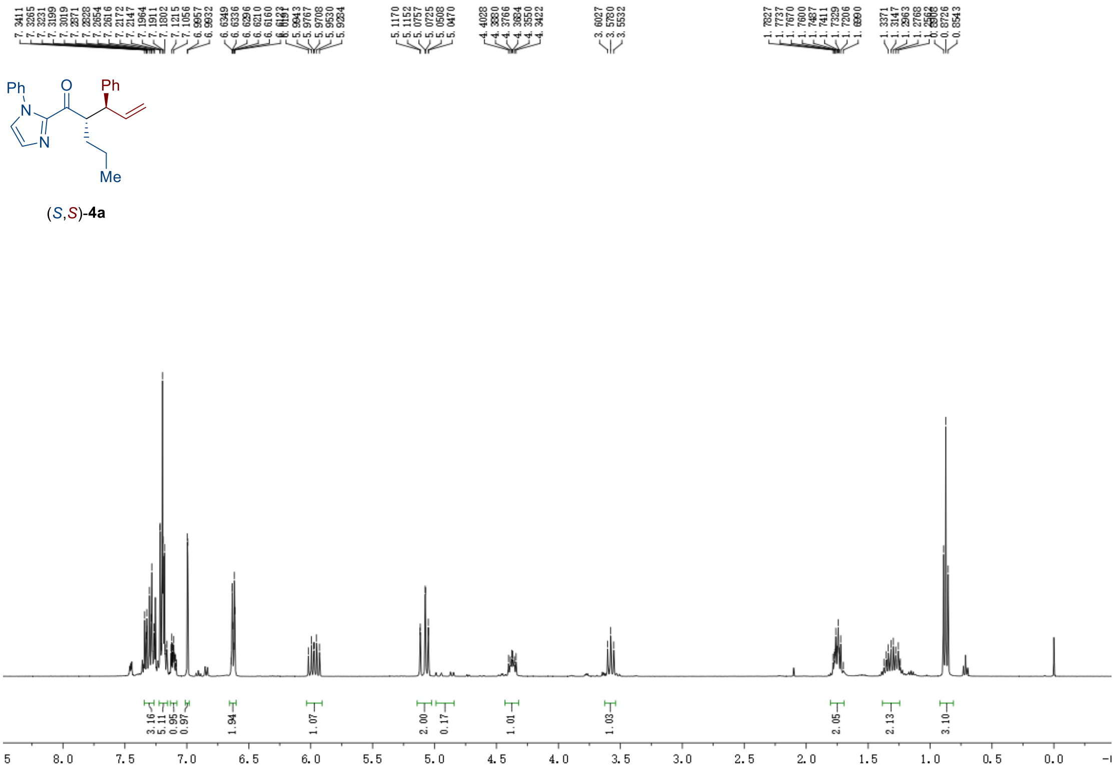

^{13}C NMR spectrum of (*S*,*S*)-**4a**

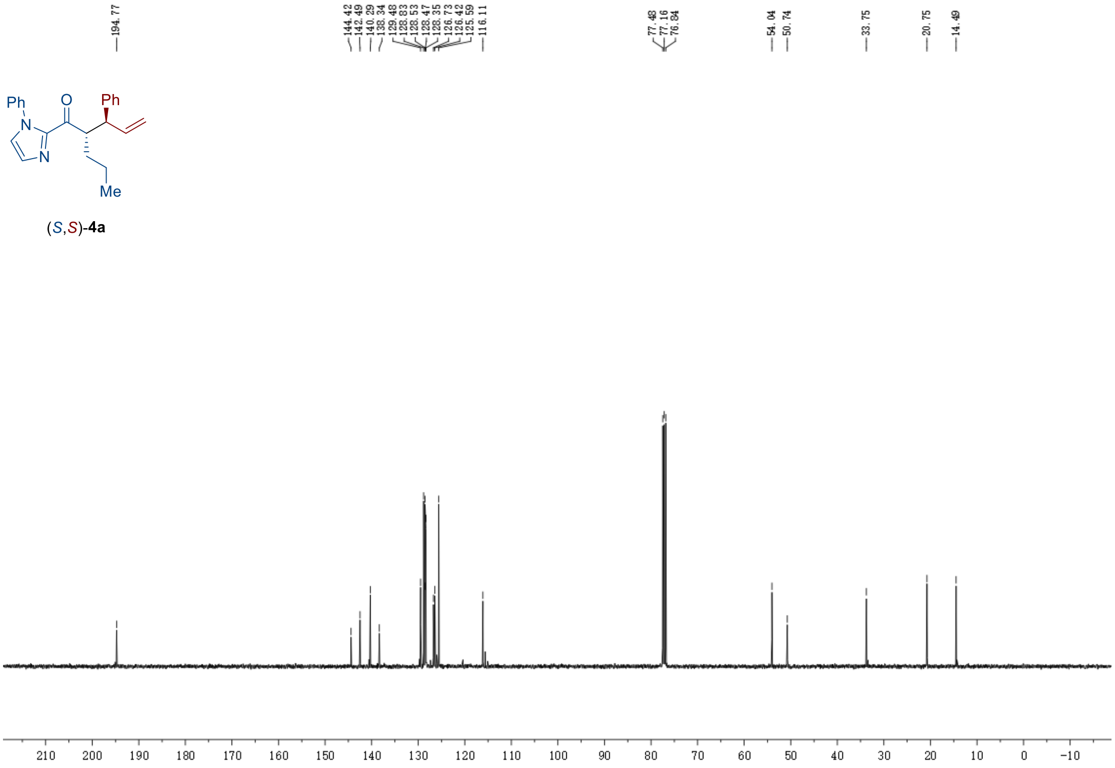

Supplementary Figure 27. NMR spectra of compound (*S*,*S*)-**4a**

^{1}H NMR spectrum of **4b**

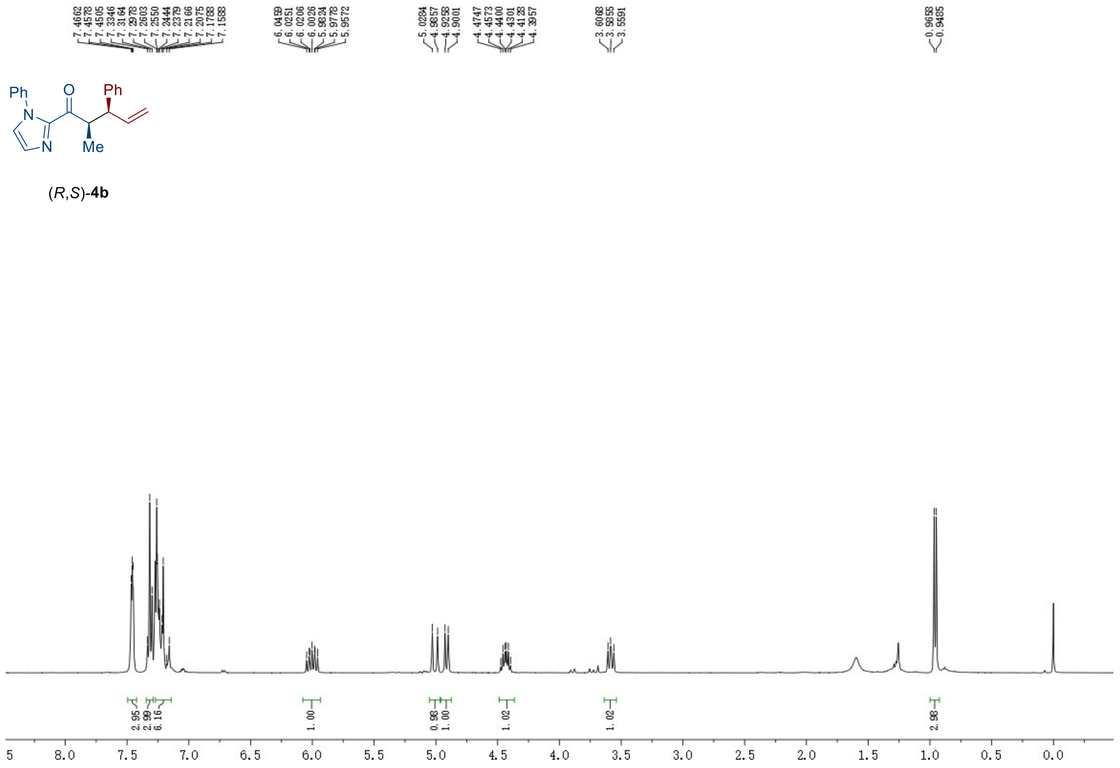

^{13}C NMR spectrum of **4b**

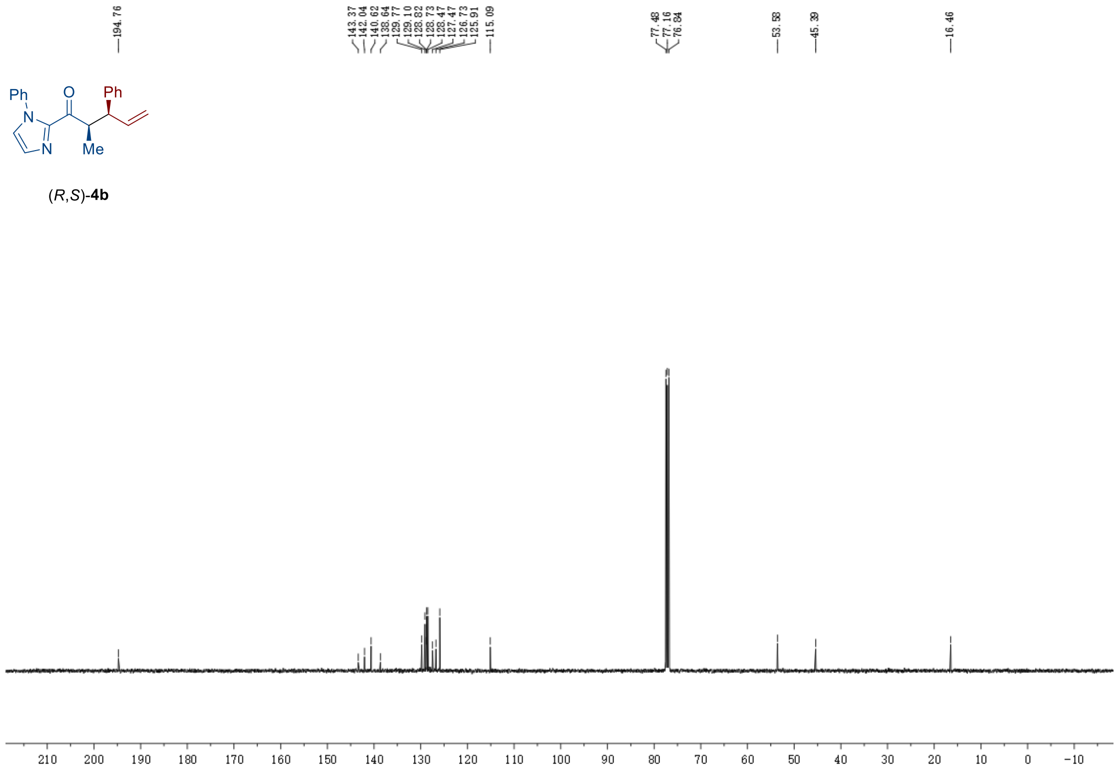

Supplementary Figure 28. NMR spectra of compound **4b**

^{1}H NMR spectrum of **4c**

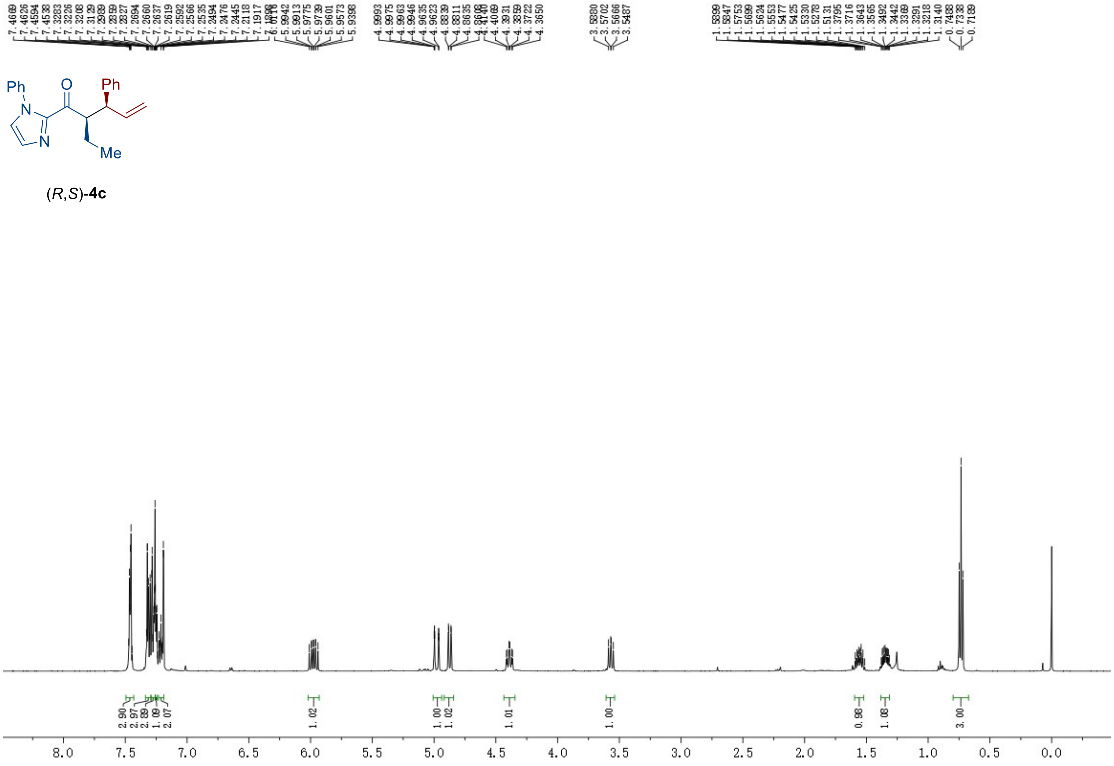

^{13}C NMR spectrum of **4c**

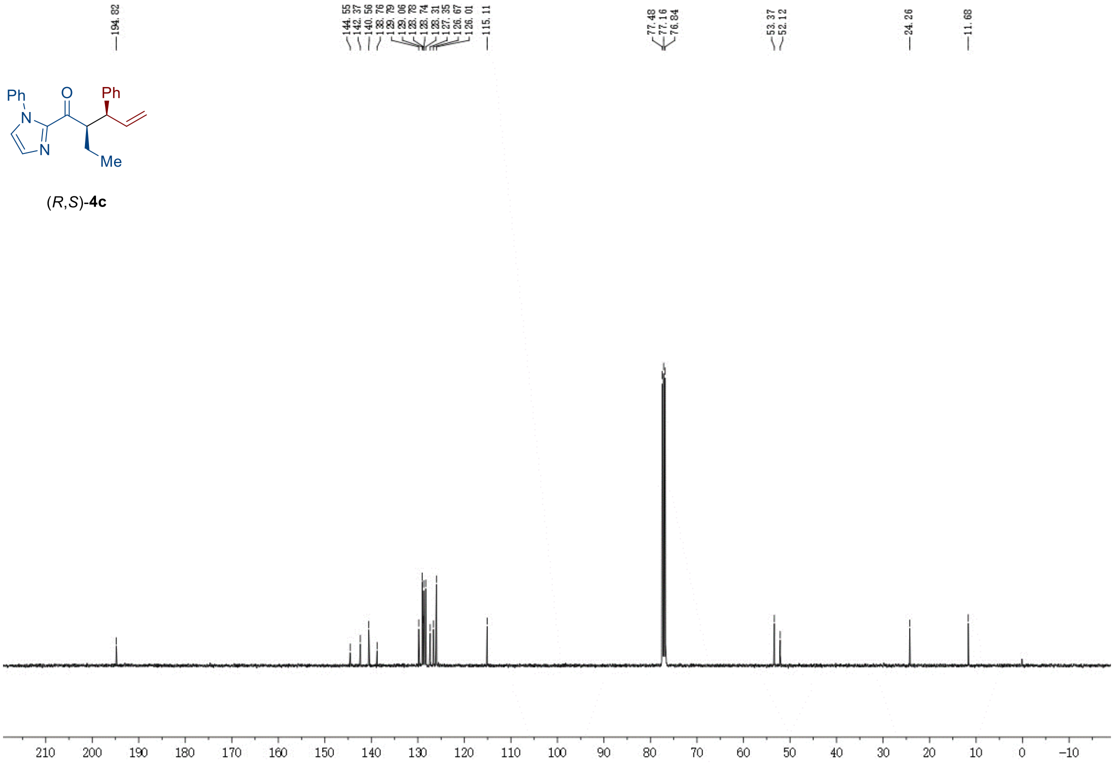

Supplementary Figure 29. NMR spectra of compound **4c**

^{1}H NMR spectrum of **4d**

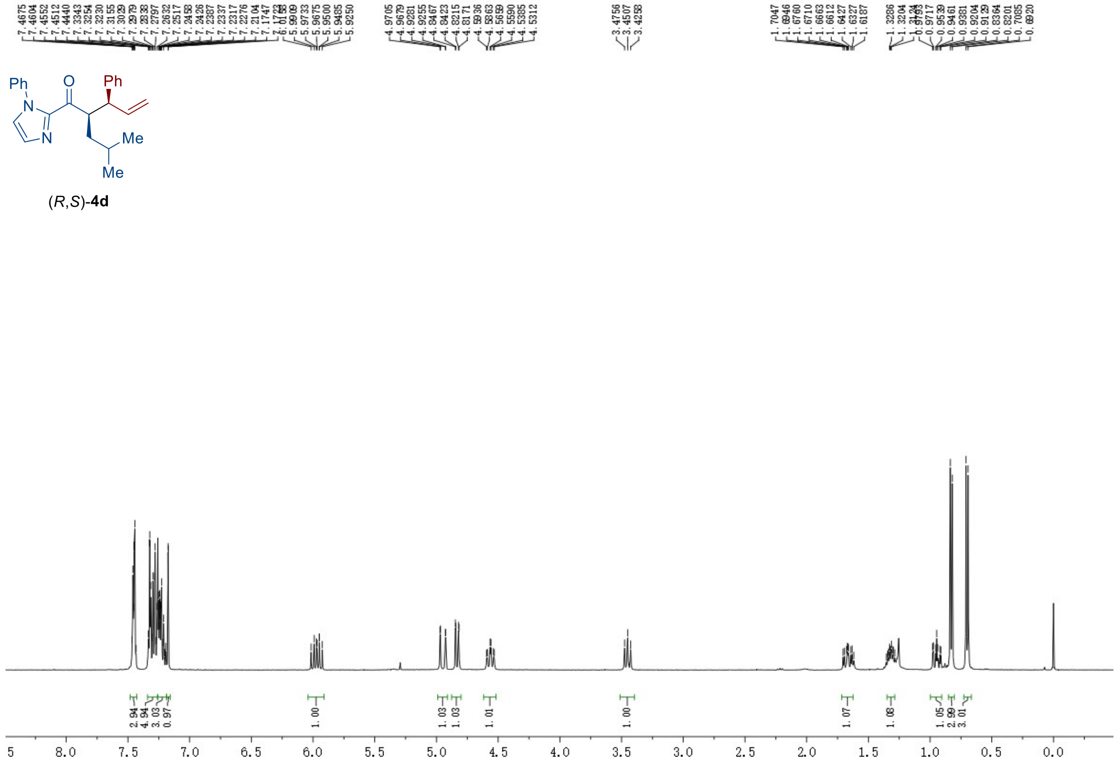

^{13}C NMR spectrum of **4d**

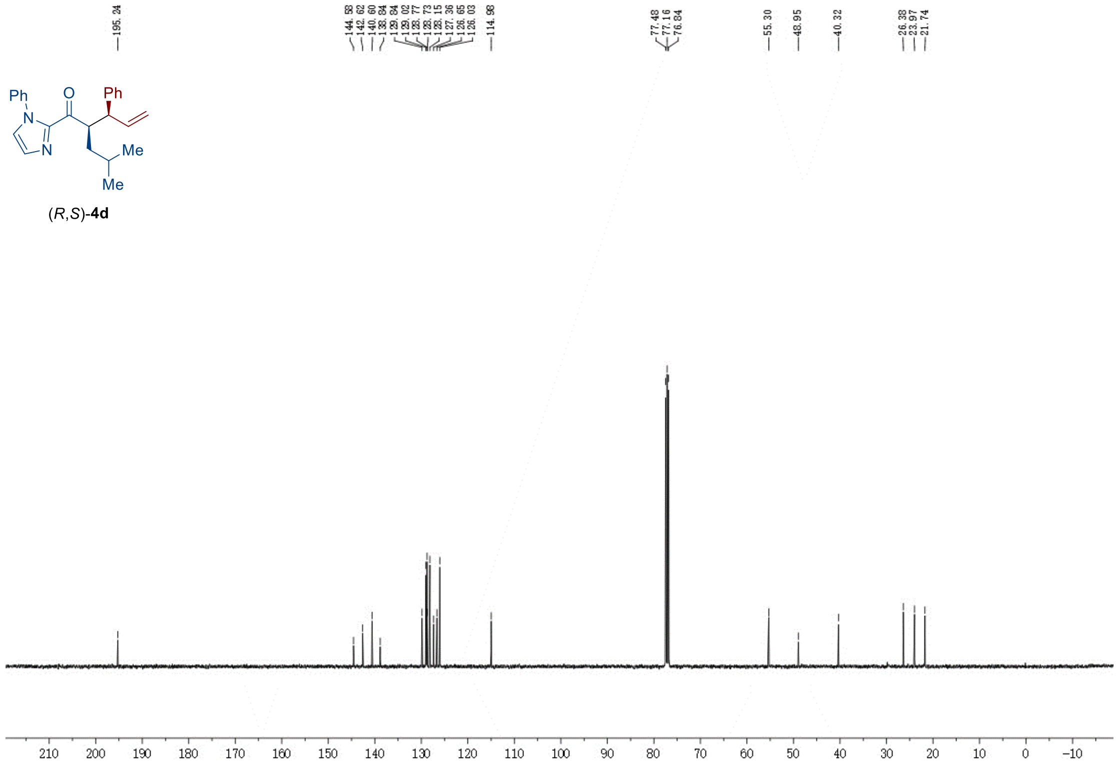

Supplementary Figure 30. NMR spectra of compound **4d**

^{1}H NMR spectrum of **4e**

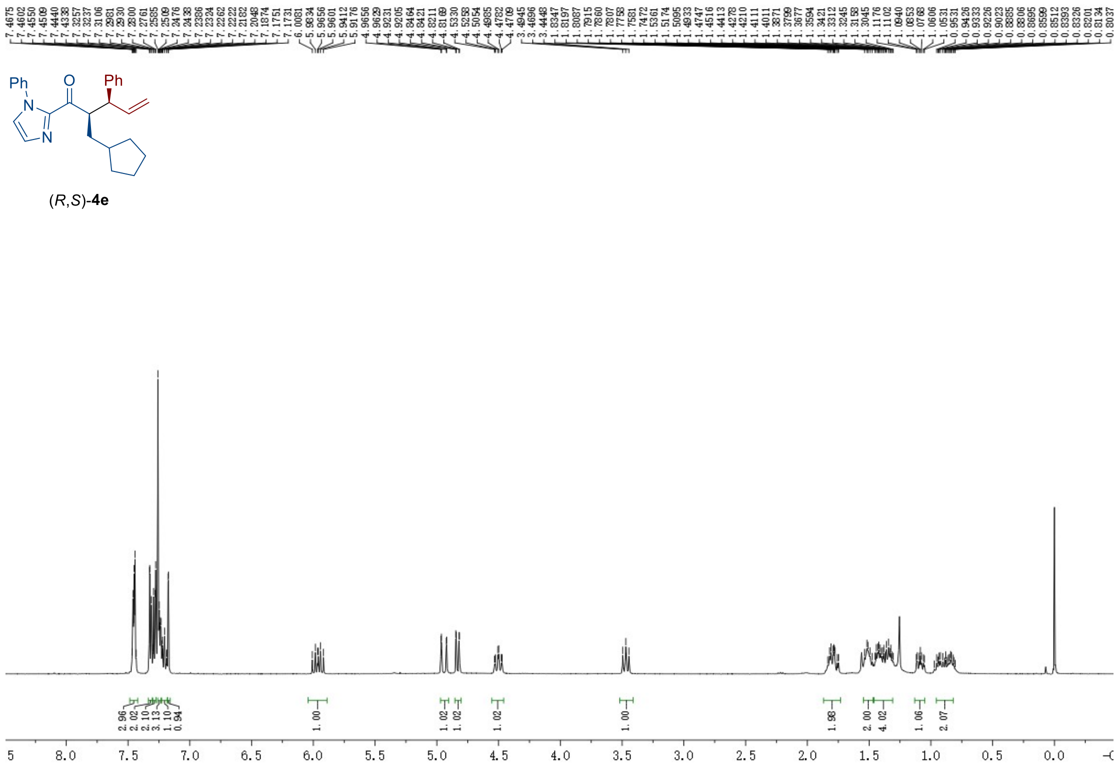

^{13}C NMR spectrum of **4e**

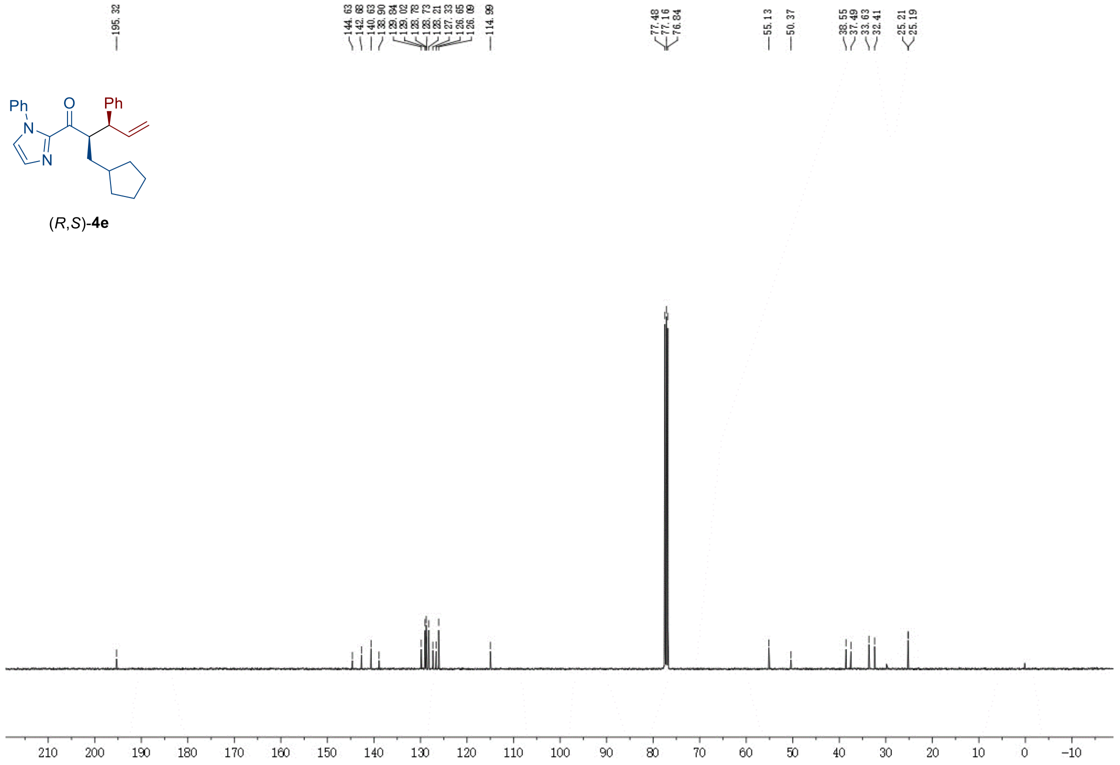

Supplementary Figure 31. NMR spectra of compound **4e**

^{1}H NMR spectrum of **4f**

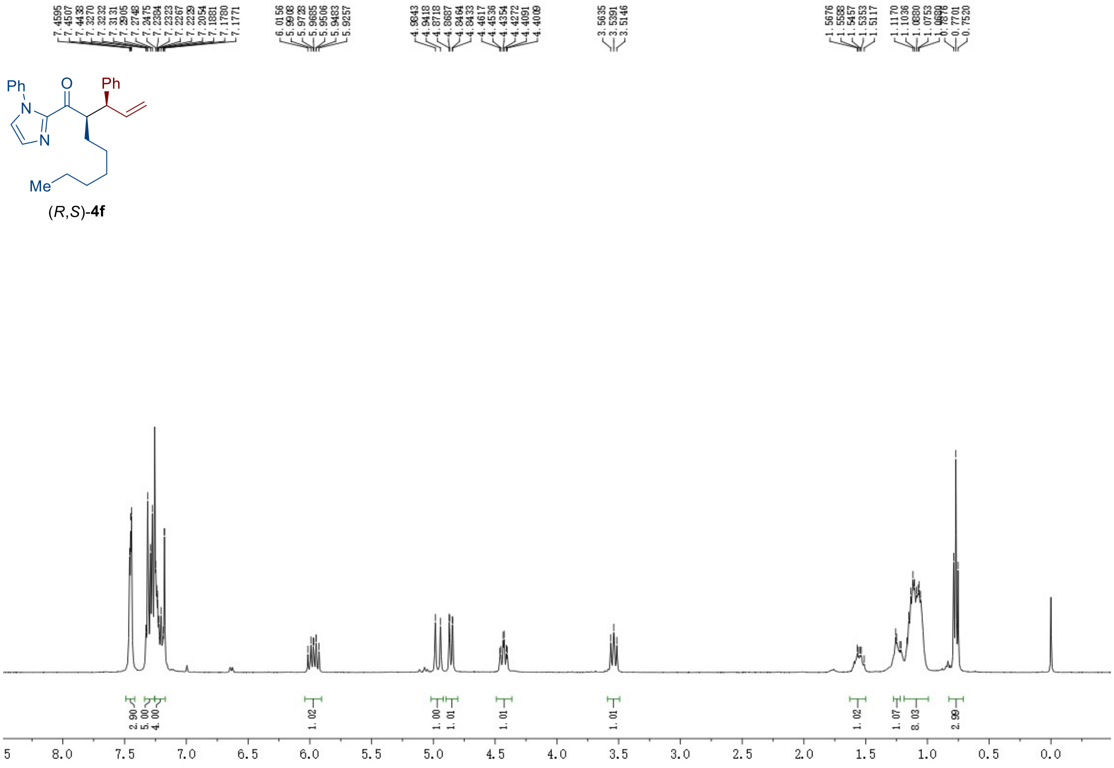

^{13}C NMR spectrum of **4f**

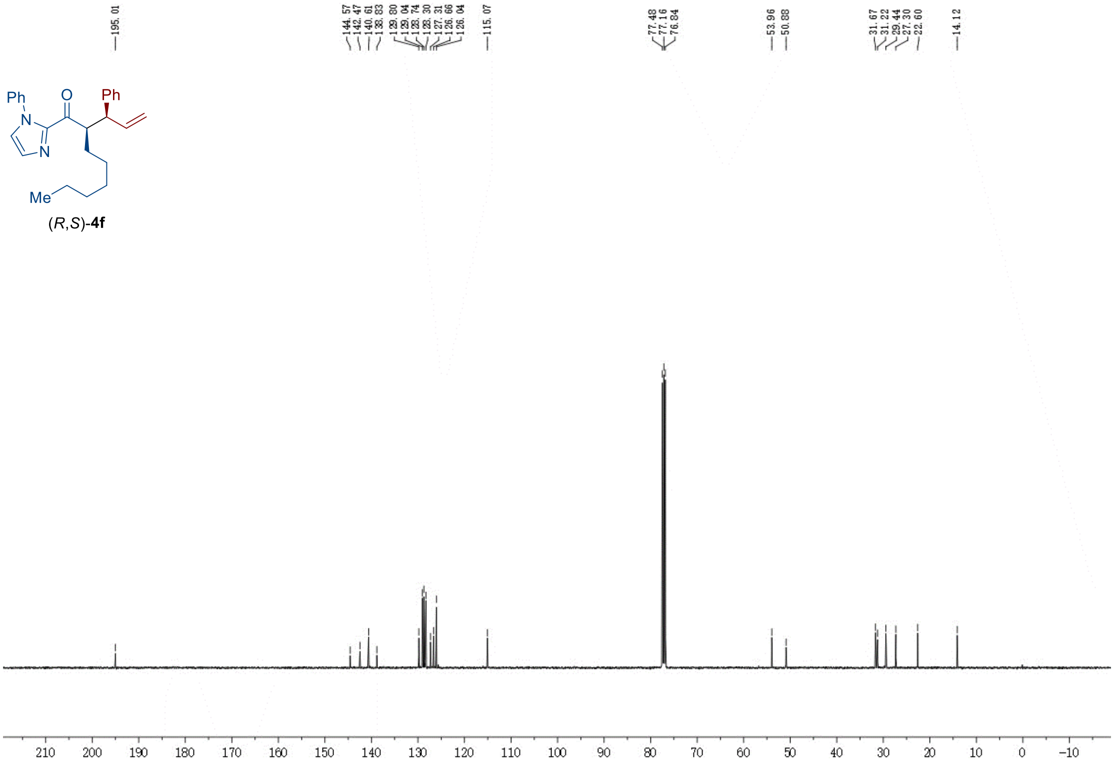

Supplementary Figure 32. NMR spectra of compound **4f**

^{1}H NMR spectrum of **4g**

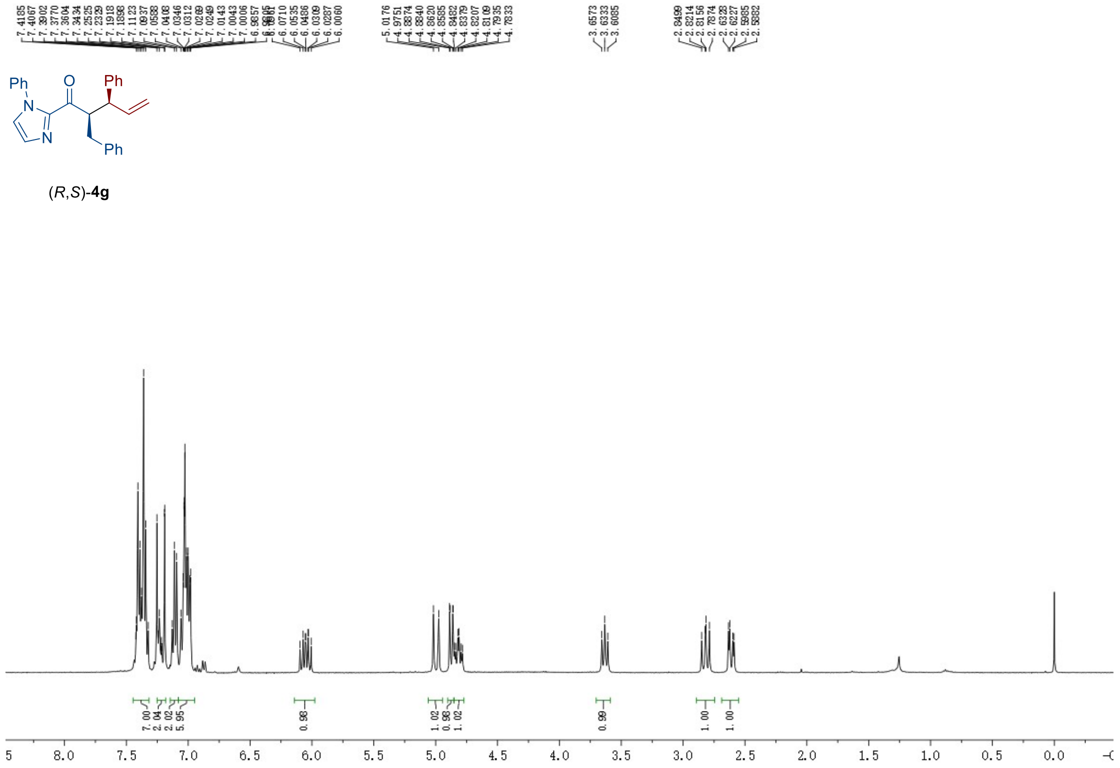

^{13}C NMR spectrum of **4g**

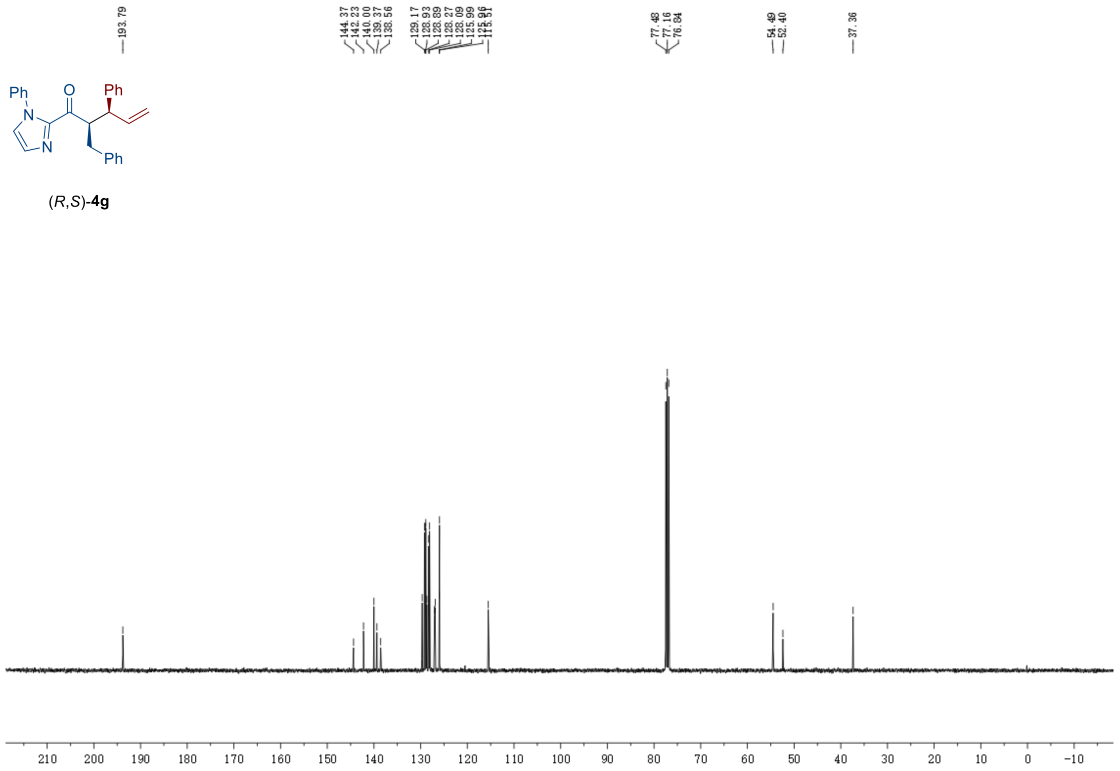

Supplementary Figure 33. NMR spectra of compound **4g**

^{1}H NMR spectrum of **4h**

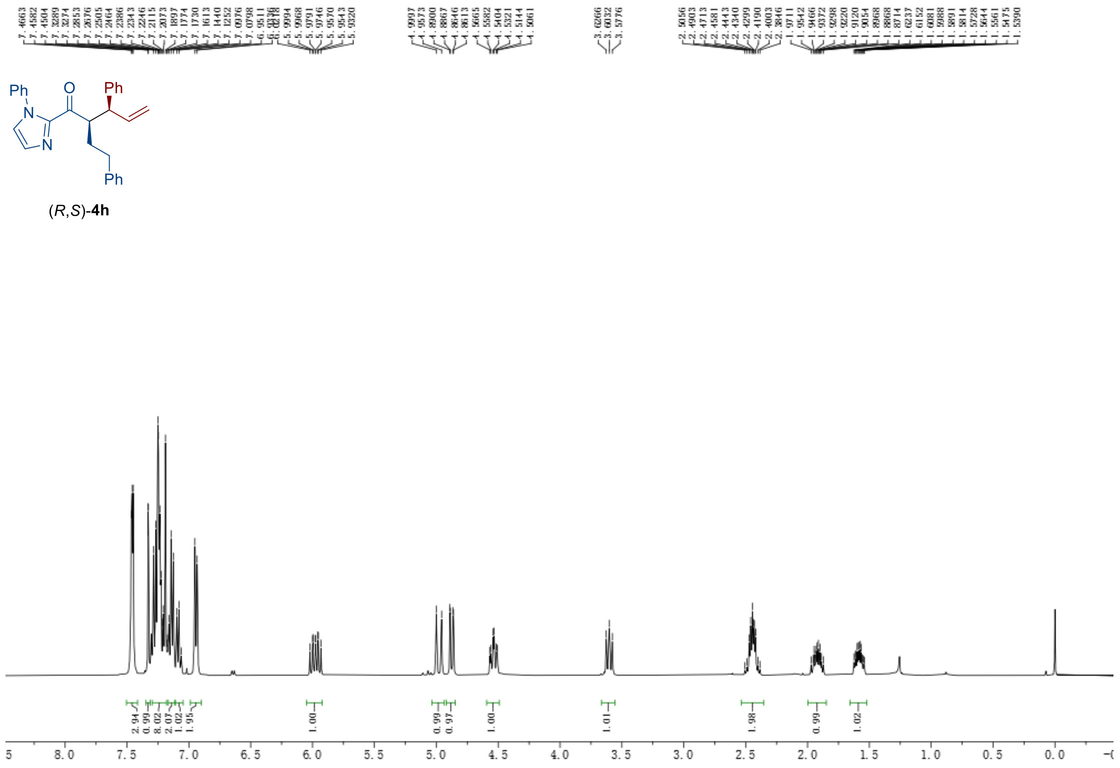

^{13}C NMR spectrum of **4h**

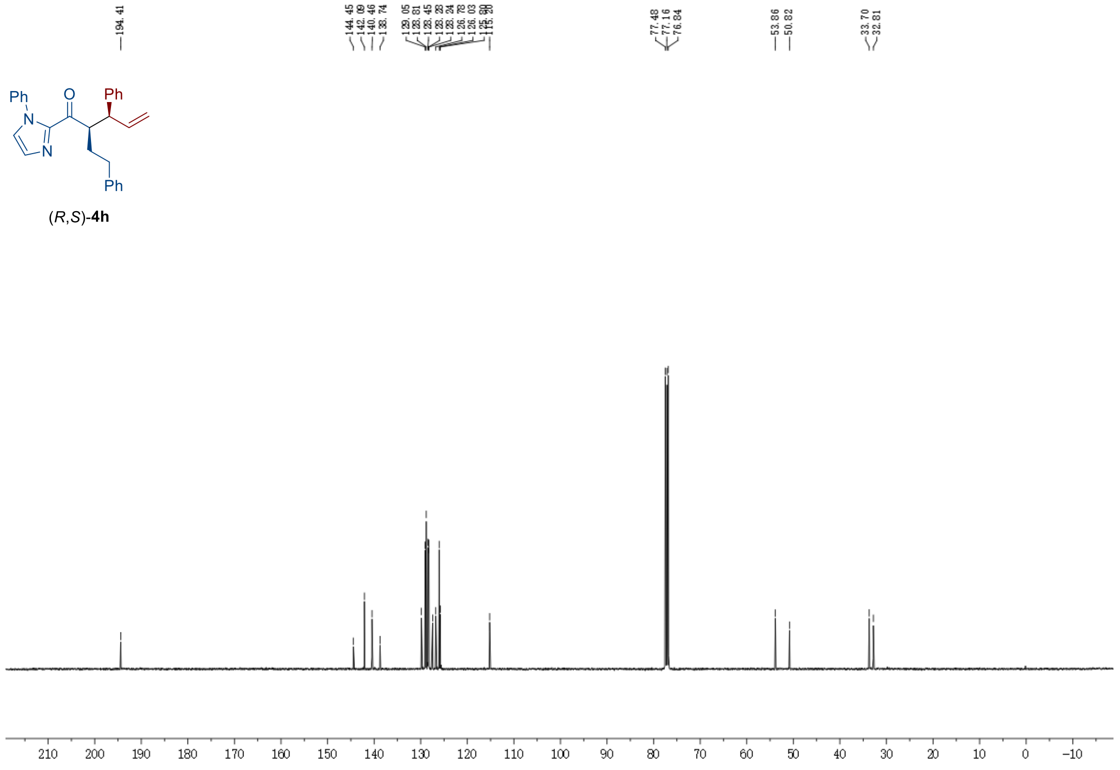

Supplementary Figure 34. NMR spectra of compound **4h**

^{1}H NMR spectrum of **4i**

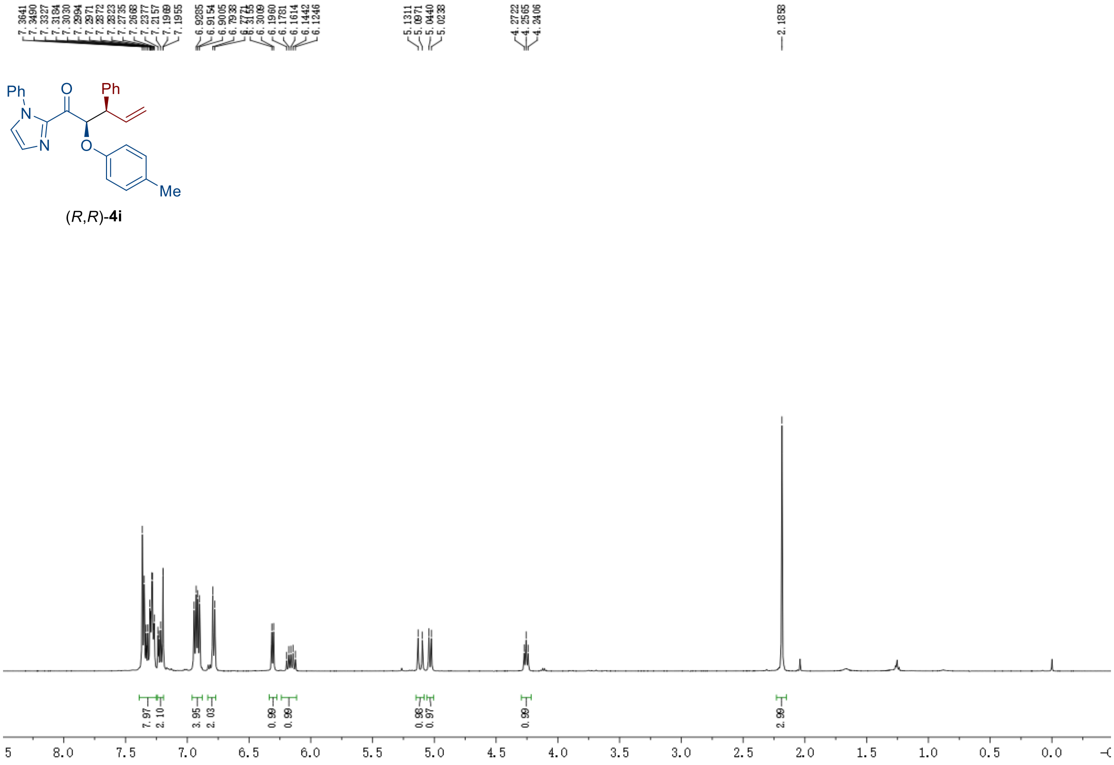

^{13}C NMR spectrum of **4i**

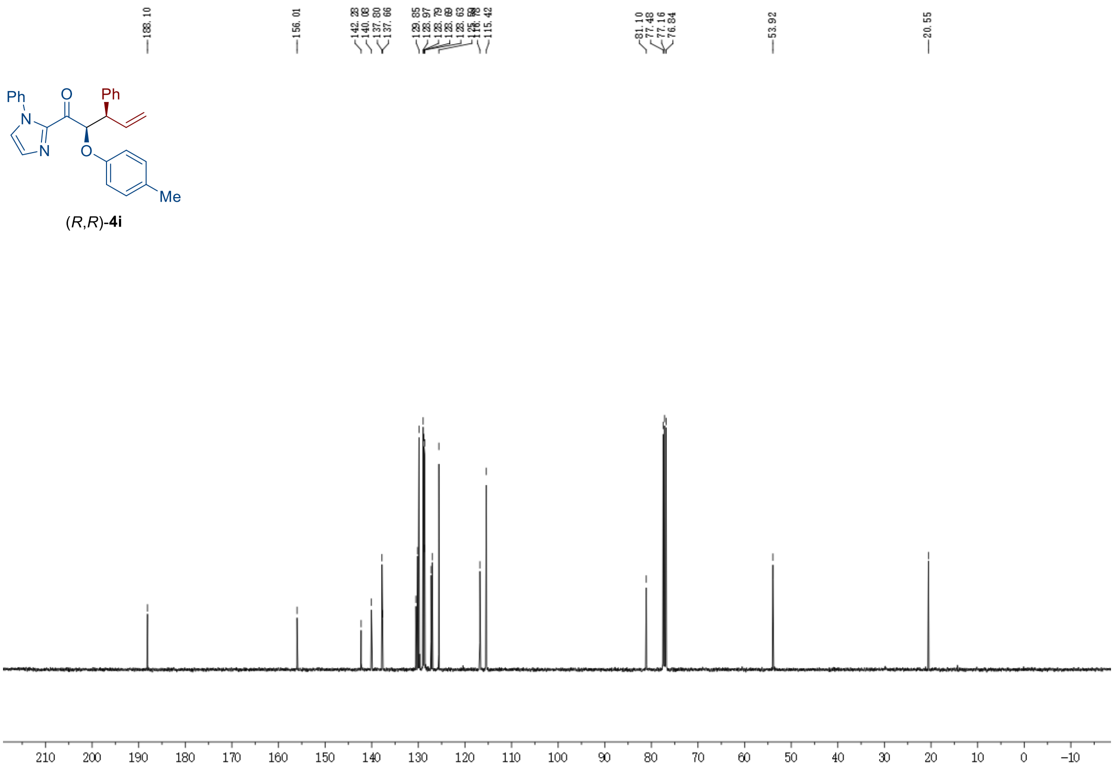

Supplementary Figure 35. NMR spectra of compound **4i**

^{1}H NMR spectrum of **4j**

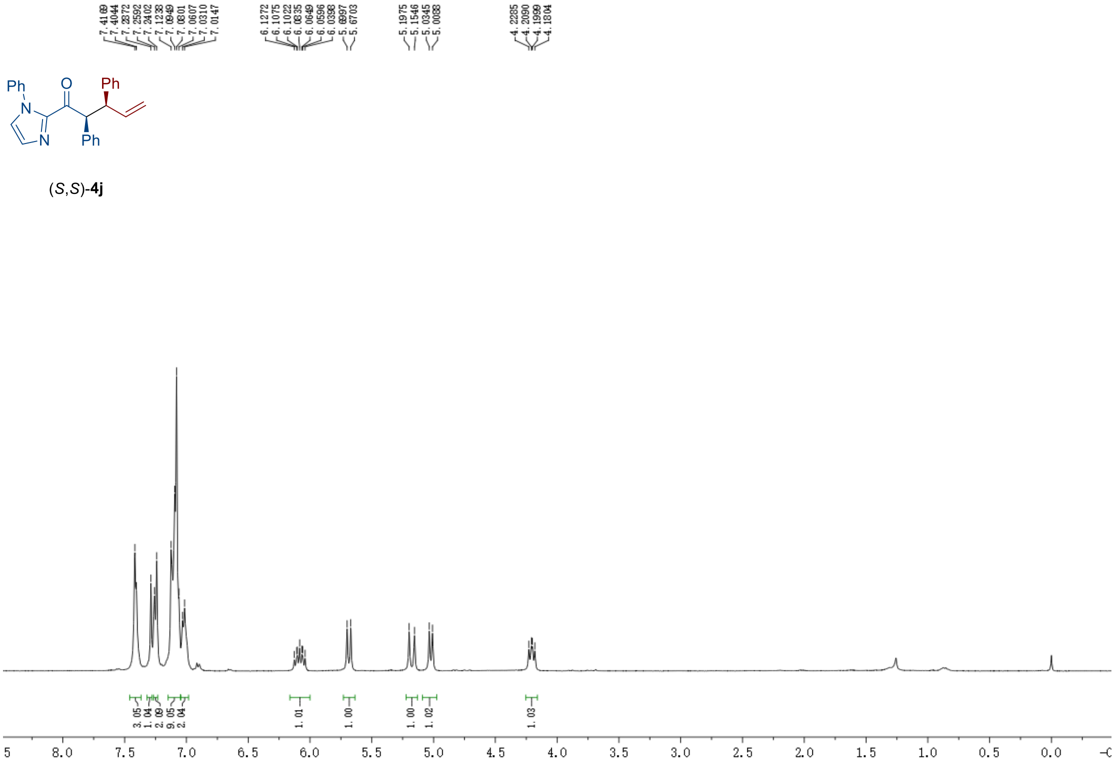

^{13}C NMR spectrum of **4j**

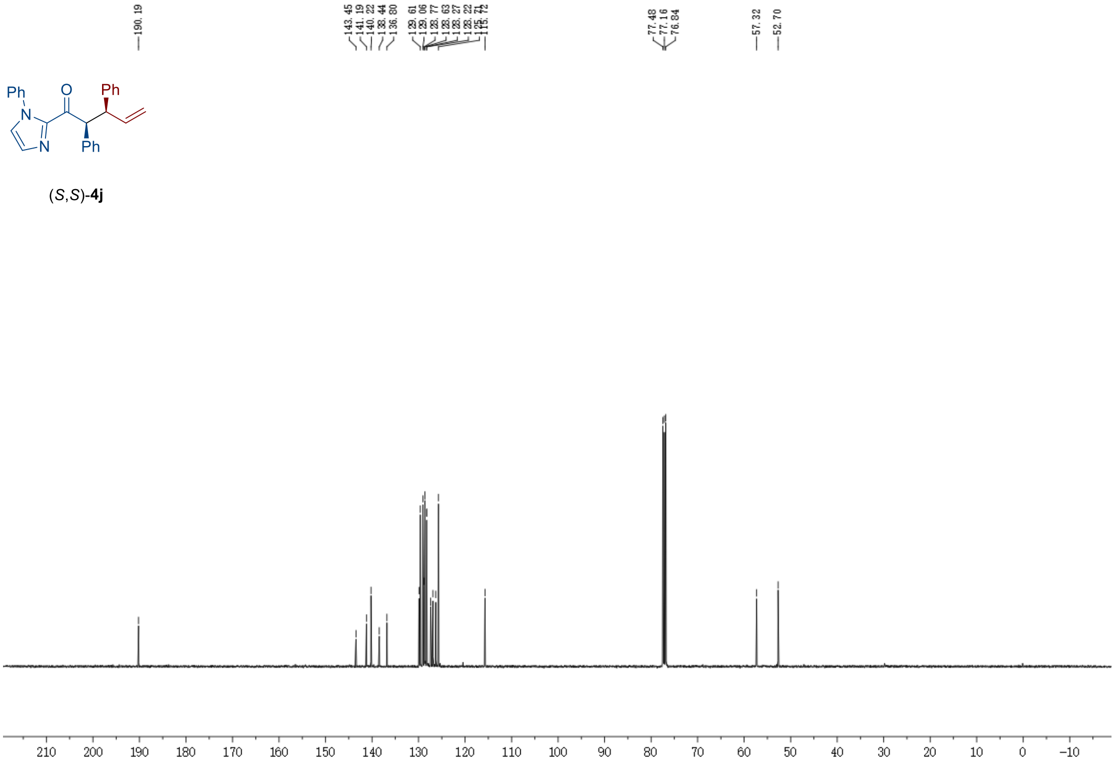

Supplementary Figure 36. NMR spectra of compound **4j**

^{1}H NMR spectrum of **4k**

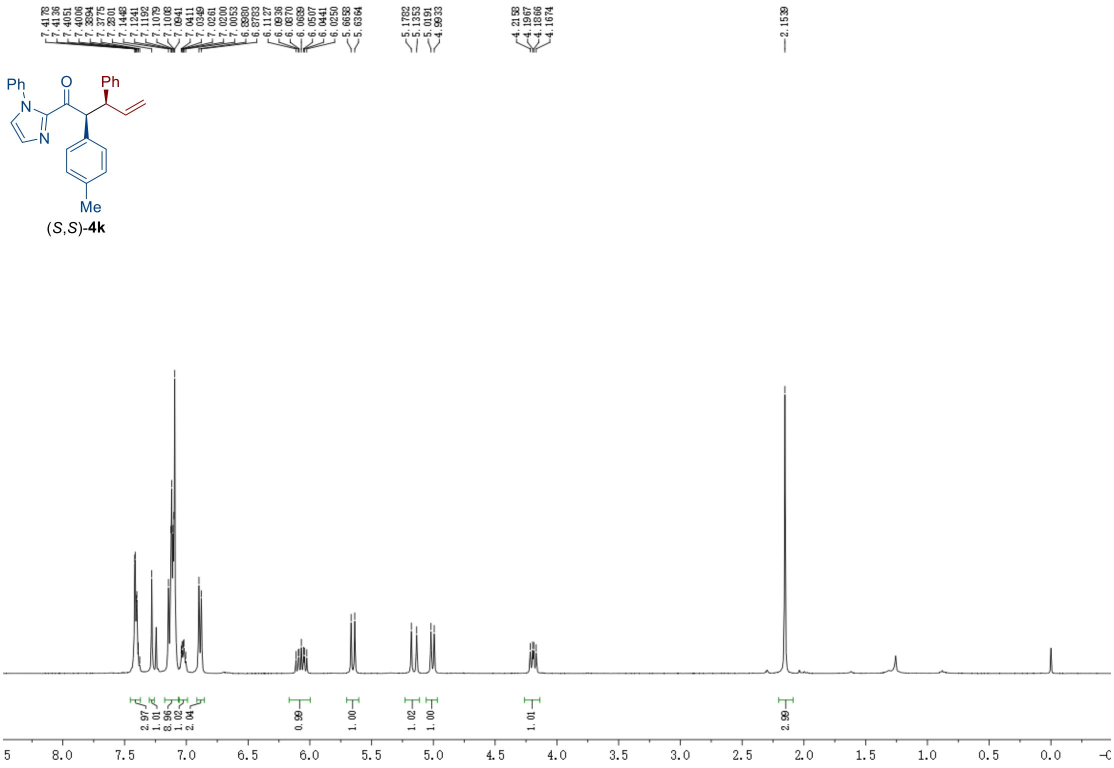

^{13}C NMR spectrum of **4k**

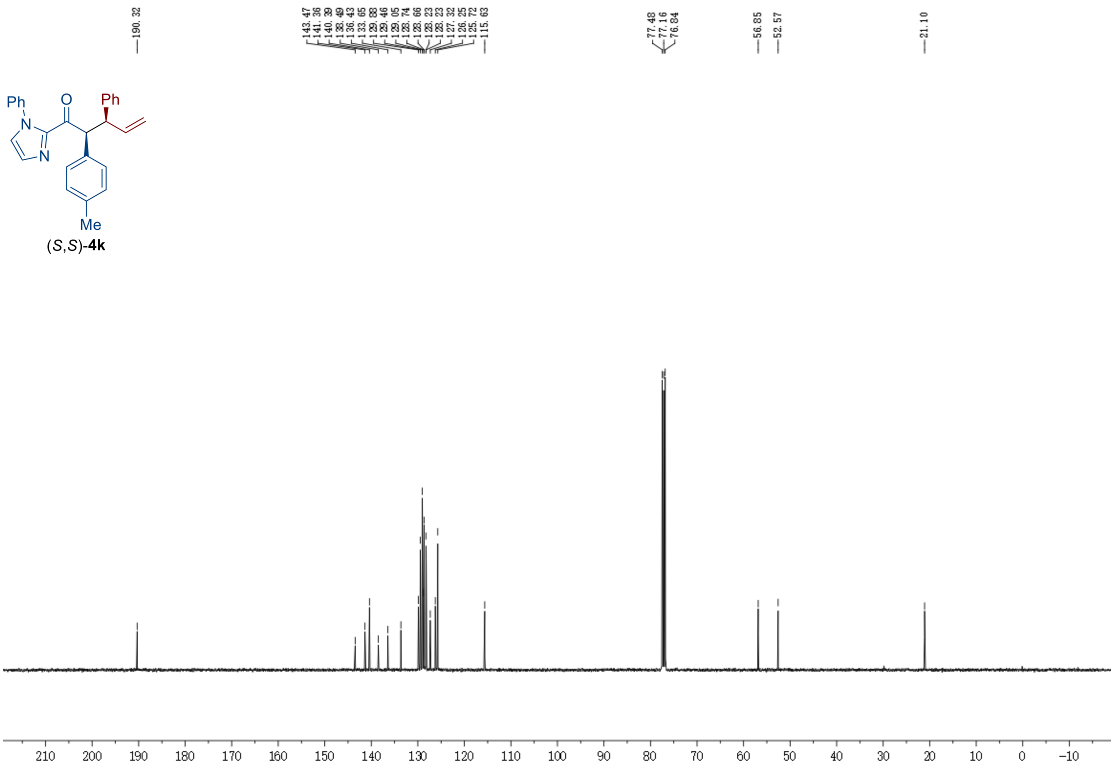

Supplementary Figure 37. NMR spectra of compound **4k**

^{1}H NMR spectrum of **4l**

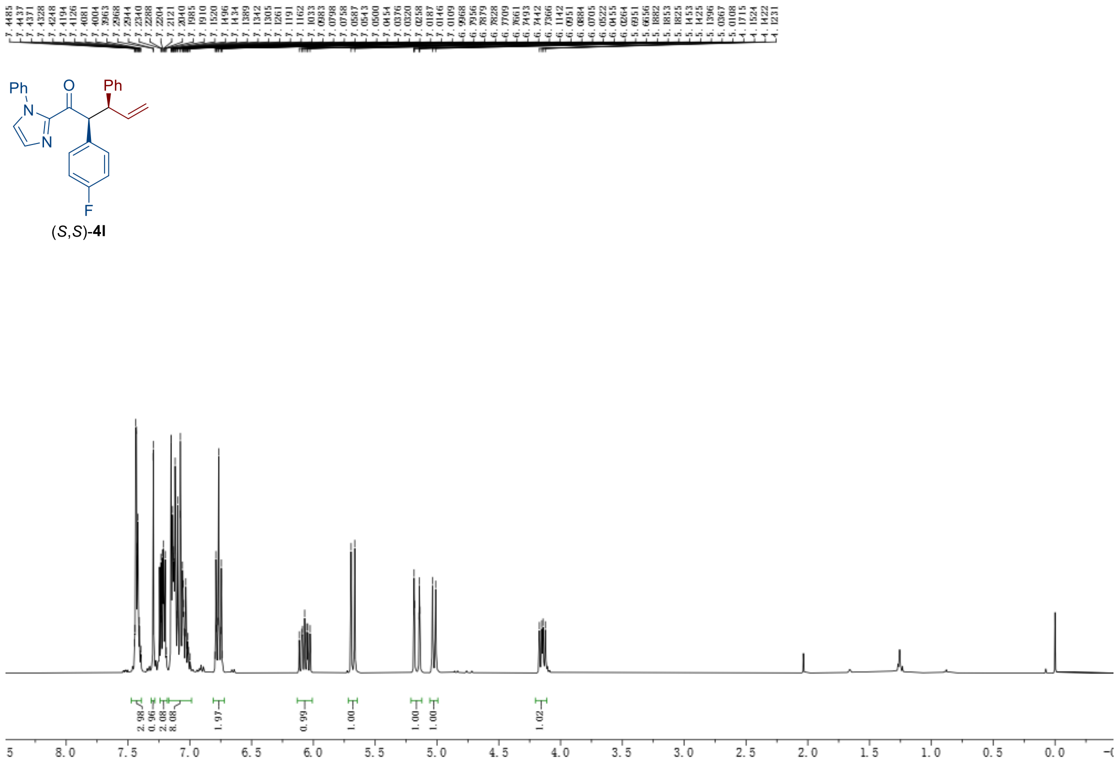

^{13}C NMR spectrum of **4l**

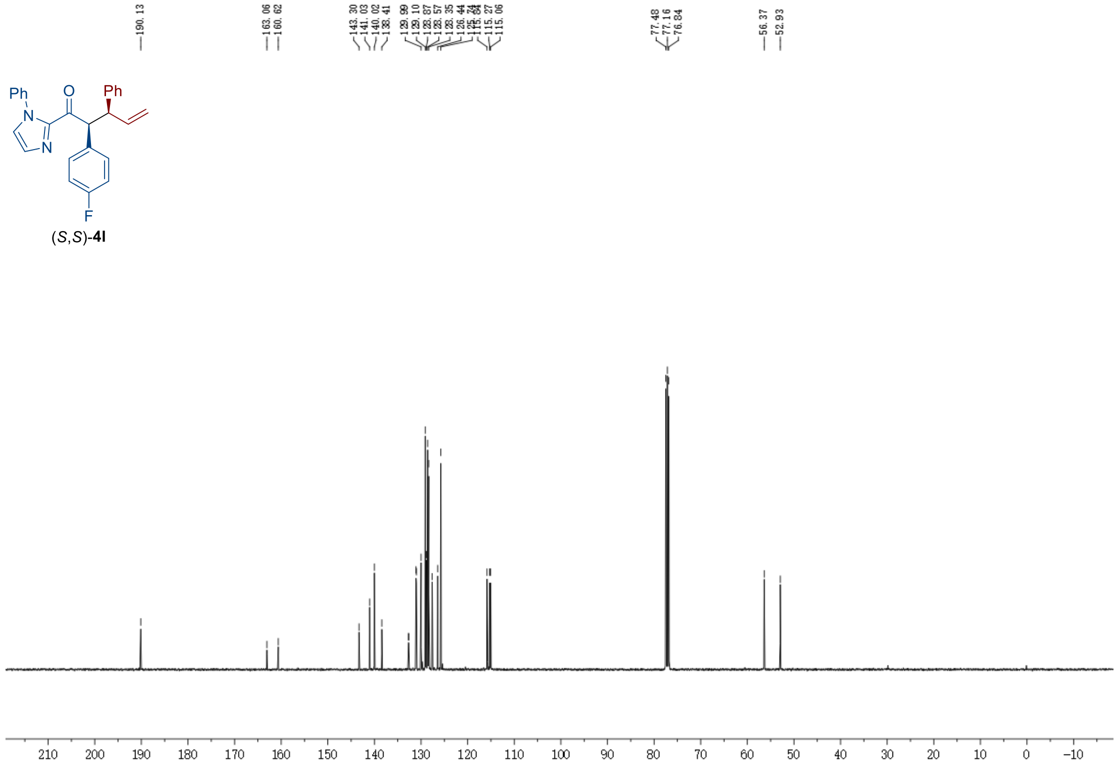

^{19}F NMR spectrum of **4l**

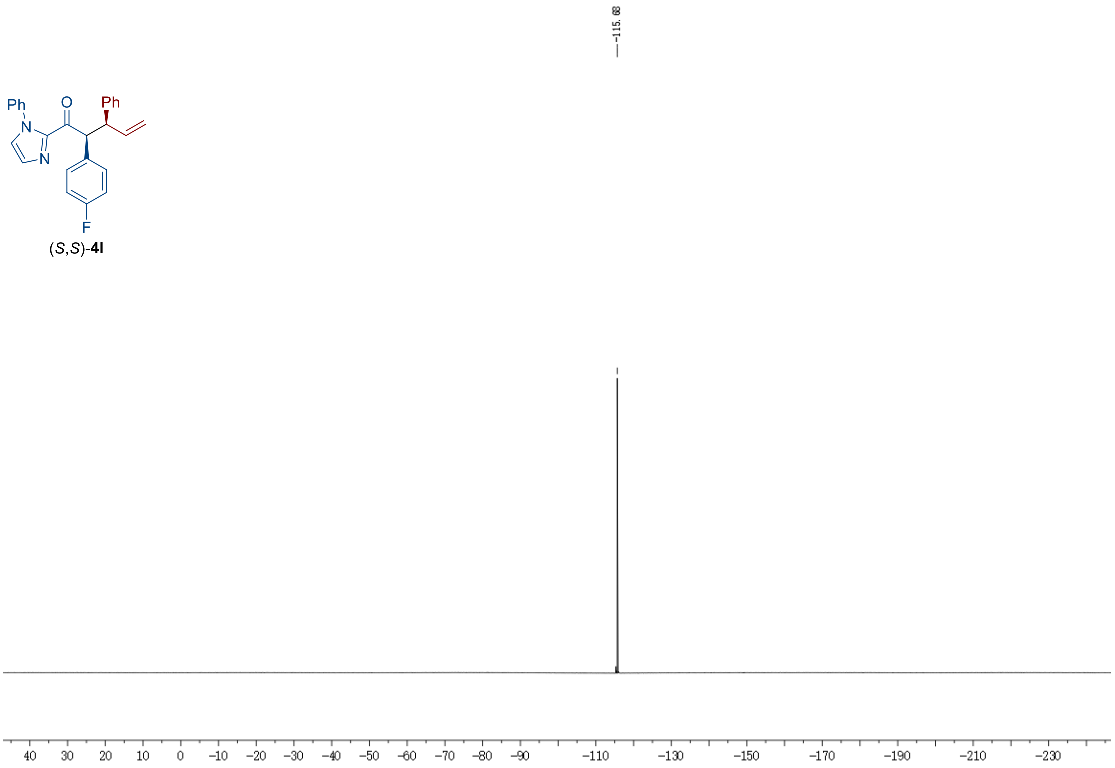

Supplementary Figure 38. NMR spectra of compound **4l**

^{1}H NMR spectrum of **4m**

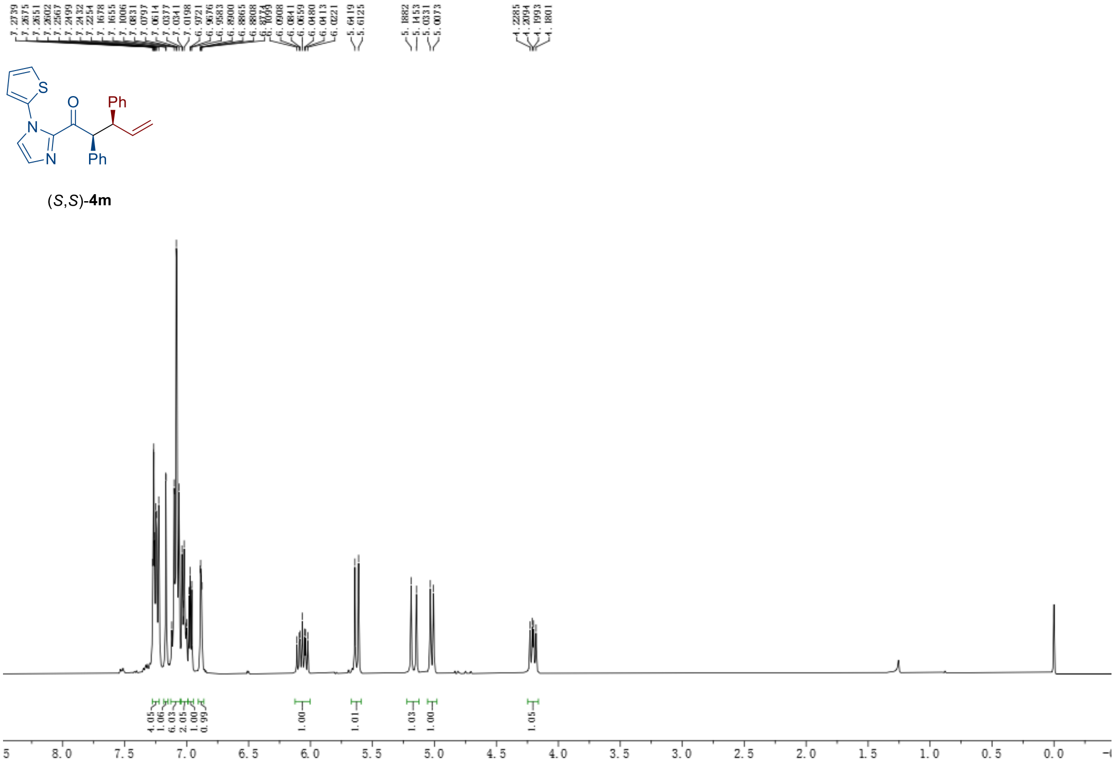

^{13}C NMR spectrum of **4m**

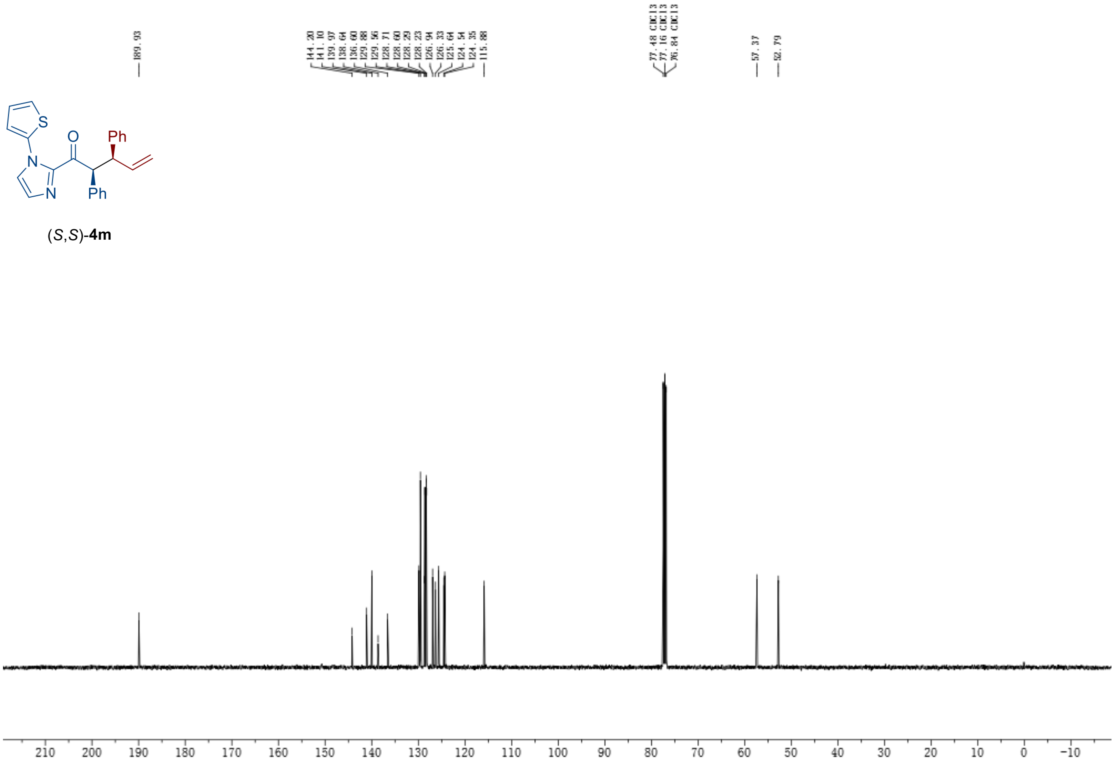

Supplementary Figure 39. NMR spectra of compound **4m**

^{1}H NMR spectrum of **4n**

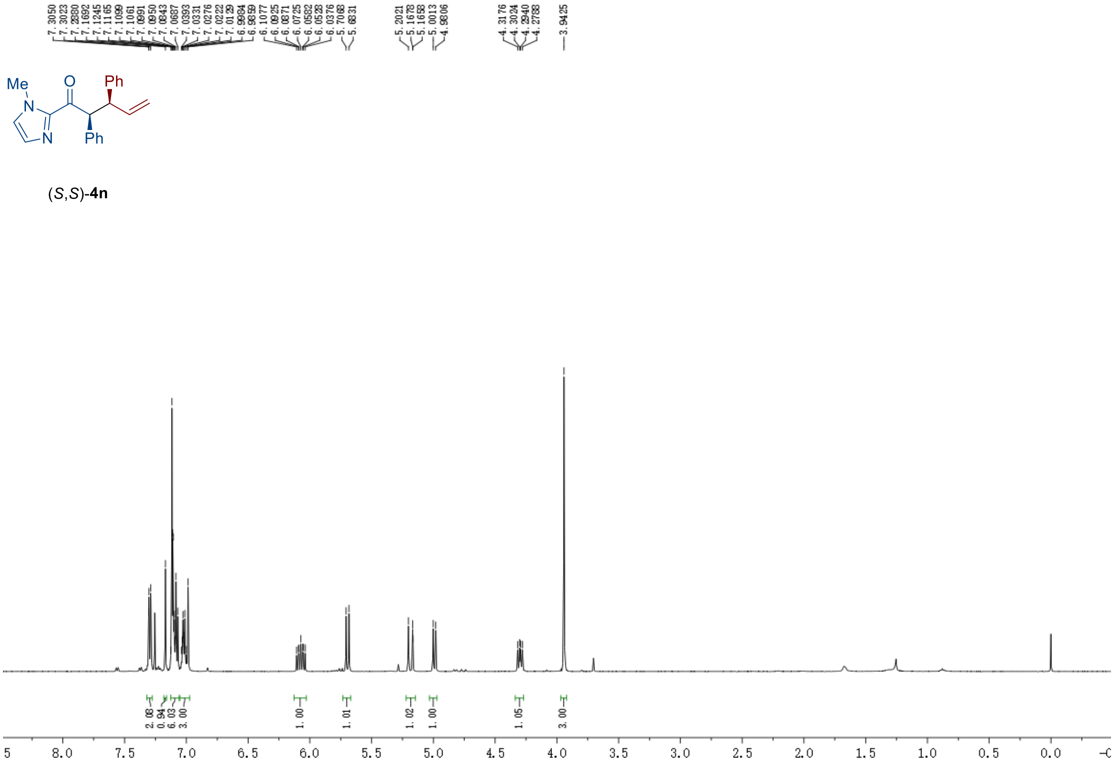

^{13}C NMR spectrum of **4n**

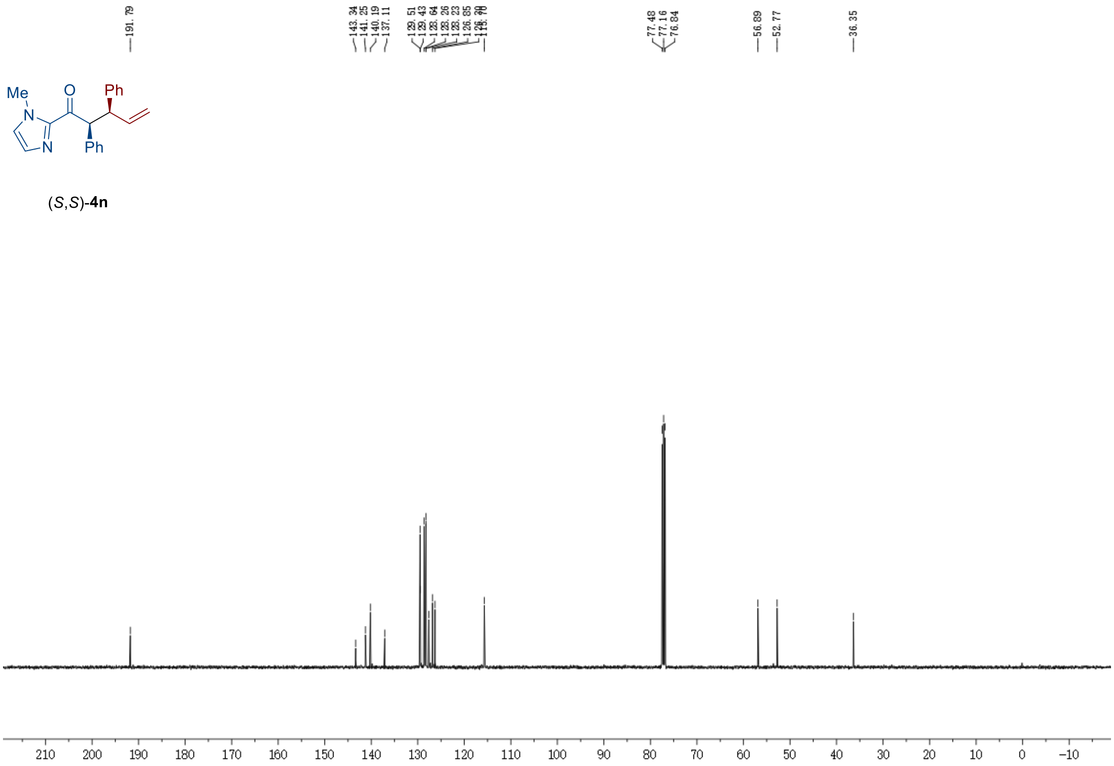

Supplementary Figure 40. NMR spectra of compound **4n**

^{1}H NMR spectrum of **4o**

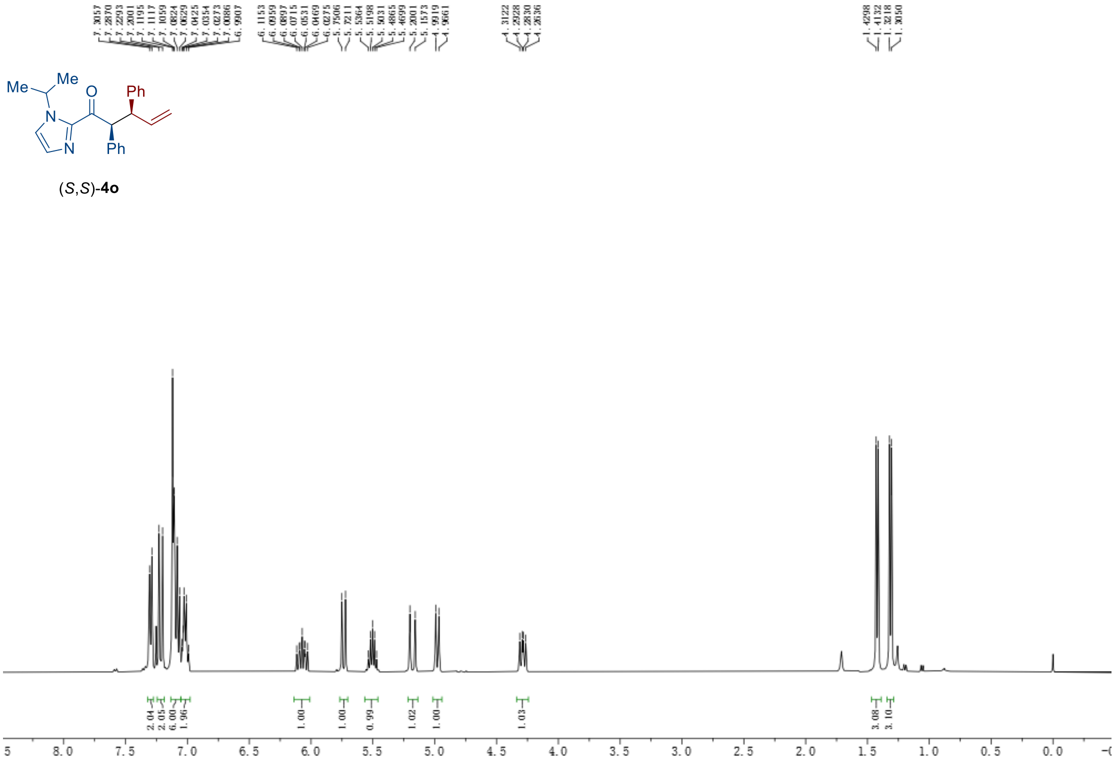

^{13}C NMR spectrum of **4o**

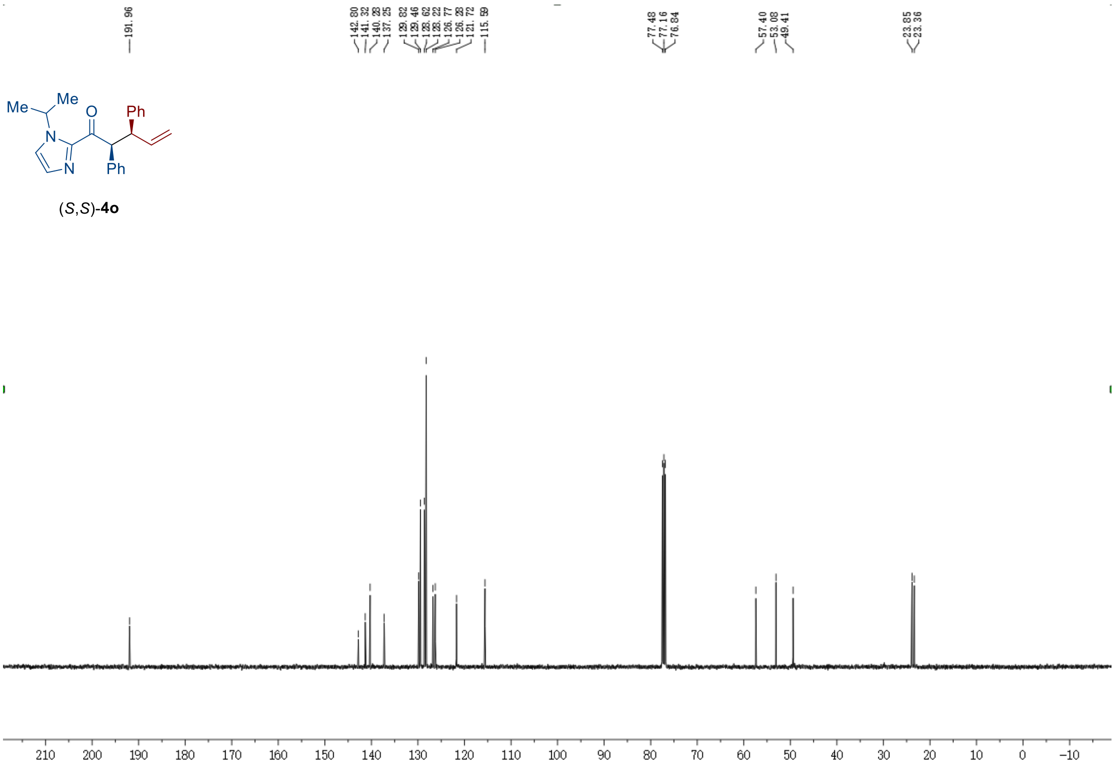

Supplementary Figure 41. NMR spectra of compound **4o**

^{1}H NMR spectrum of **4p**

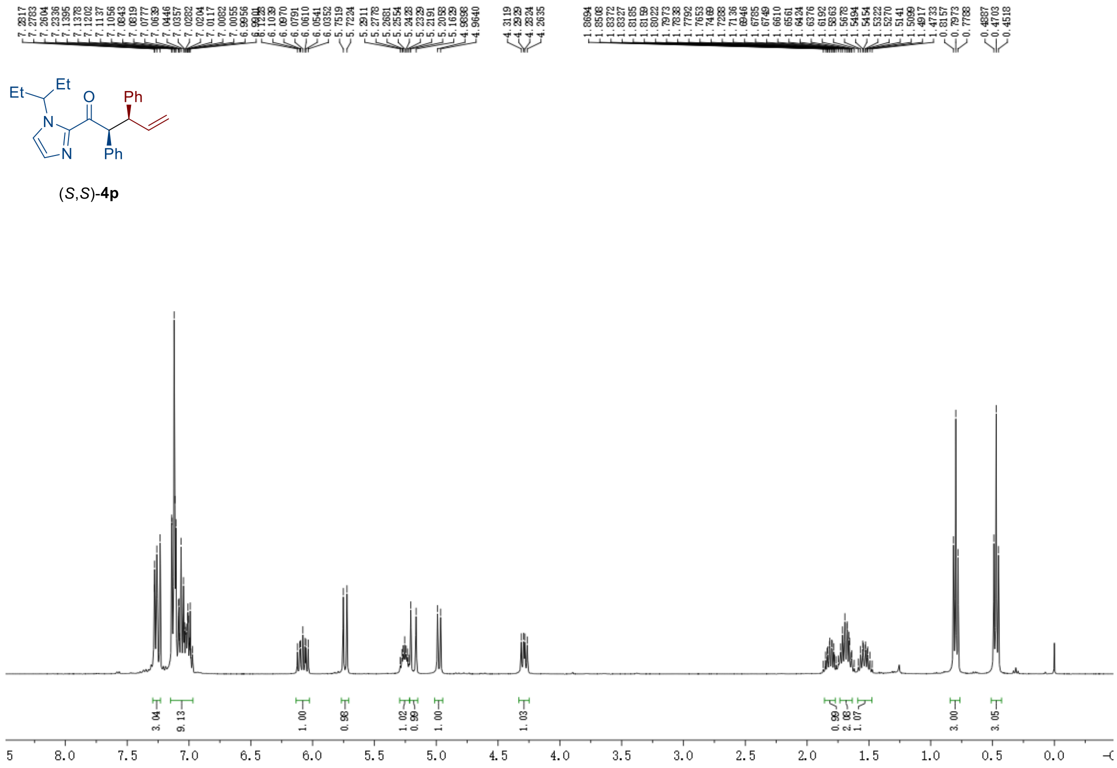

^{13}C NMR spectrum of **4p**

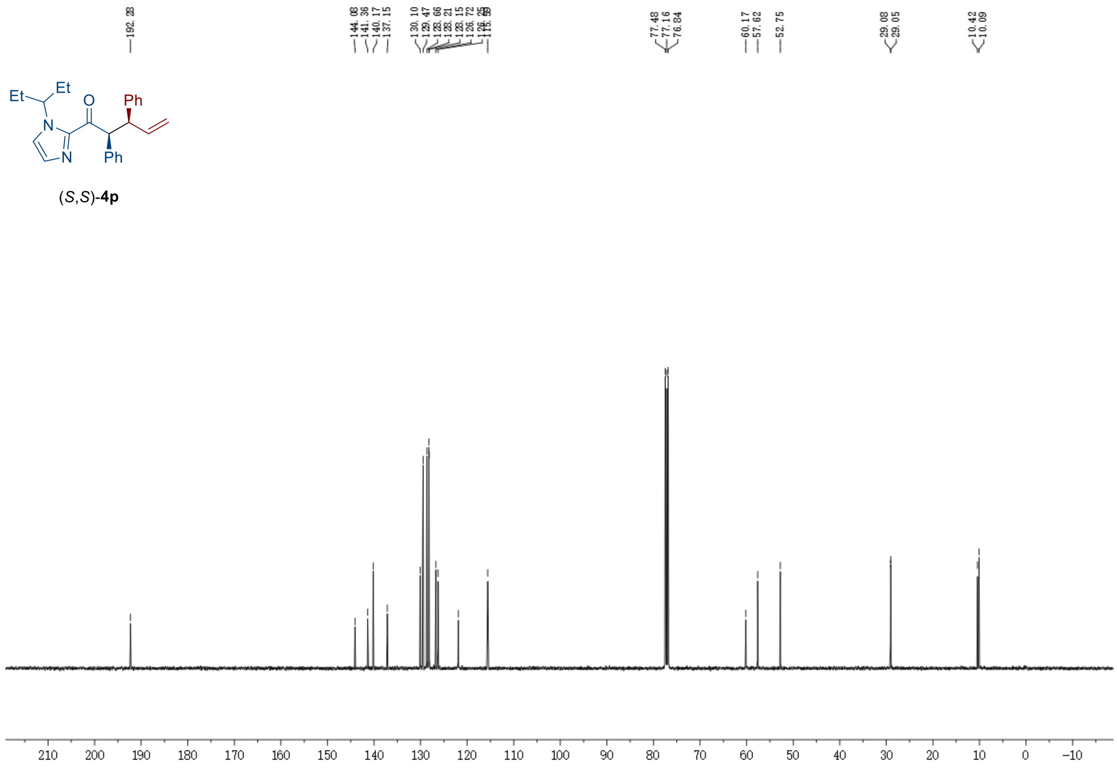

Supplementary Figure 42. NMR spectra of compound **4p**

^{1}H NMR spectrum of **4q**

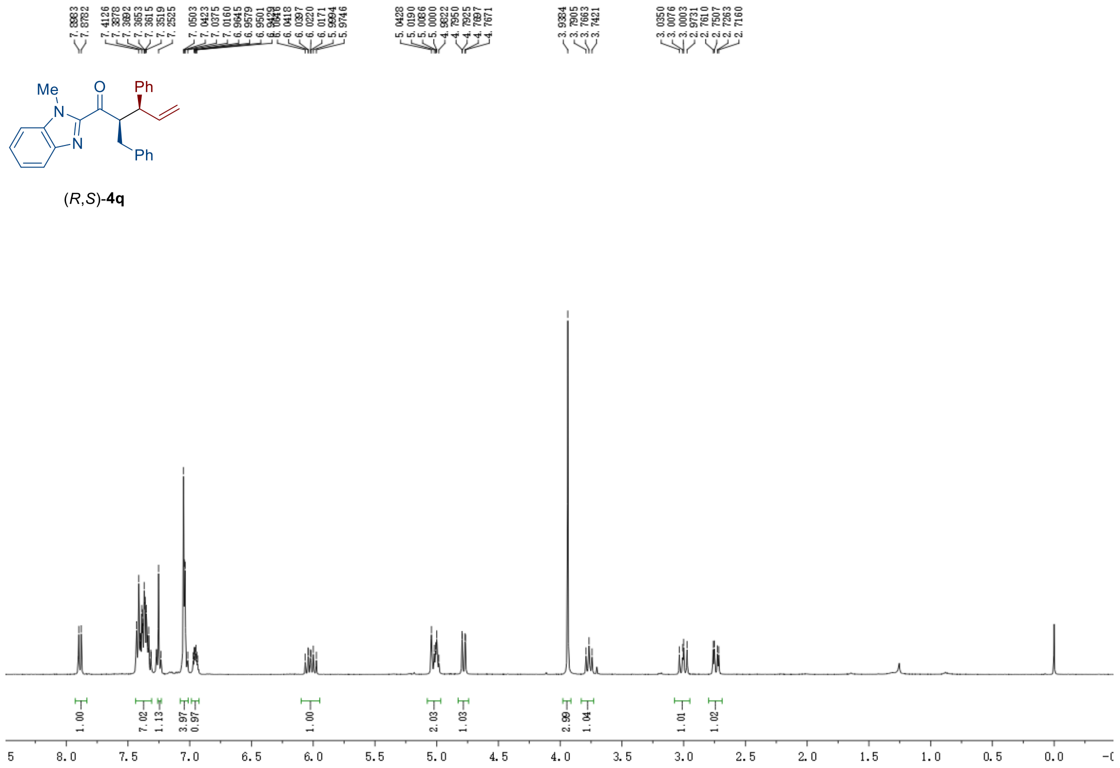

^{13}C NMR spectrum of **4q**

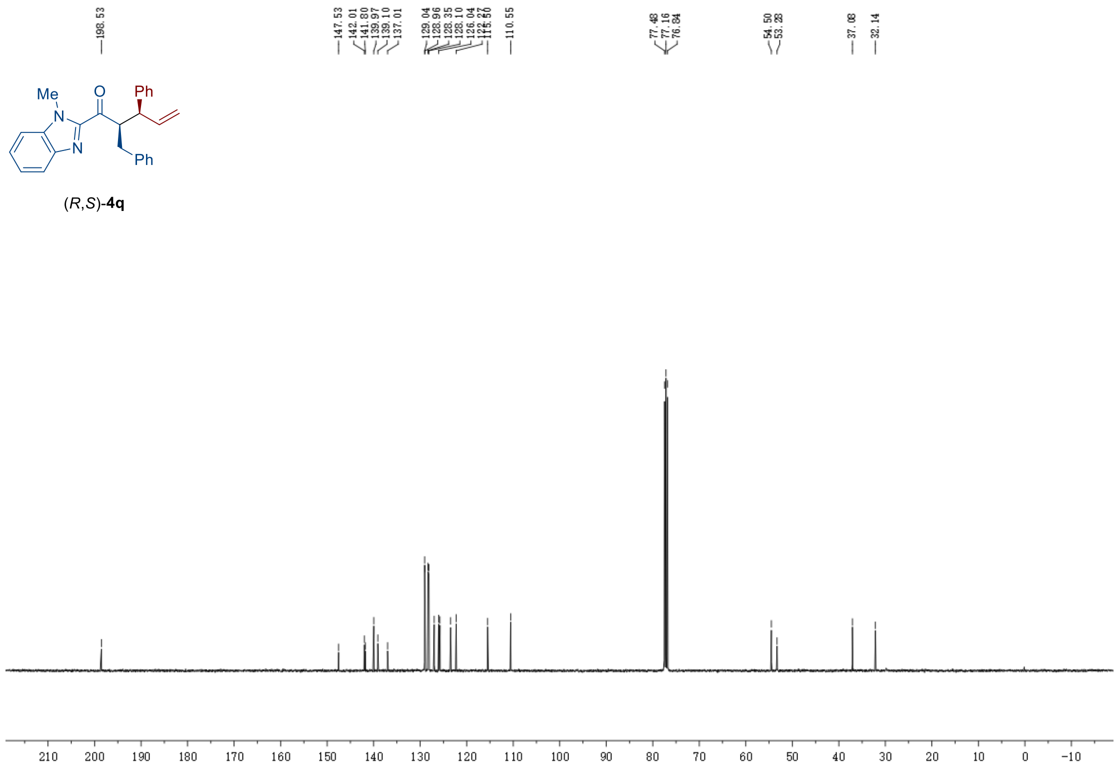

Supplementary Figure 43. NMR spectra of compound **4q**

^{1}H NMR spectrum of **4r**

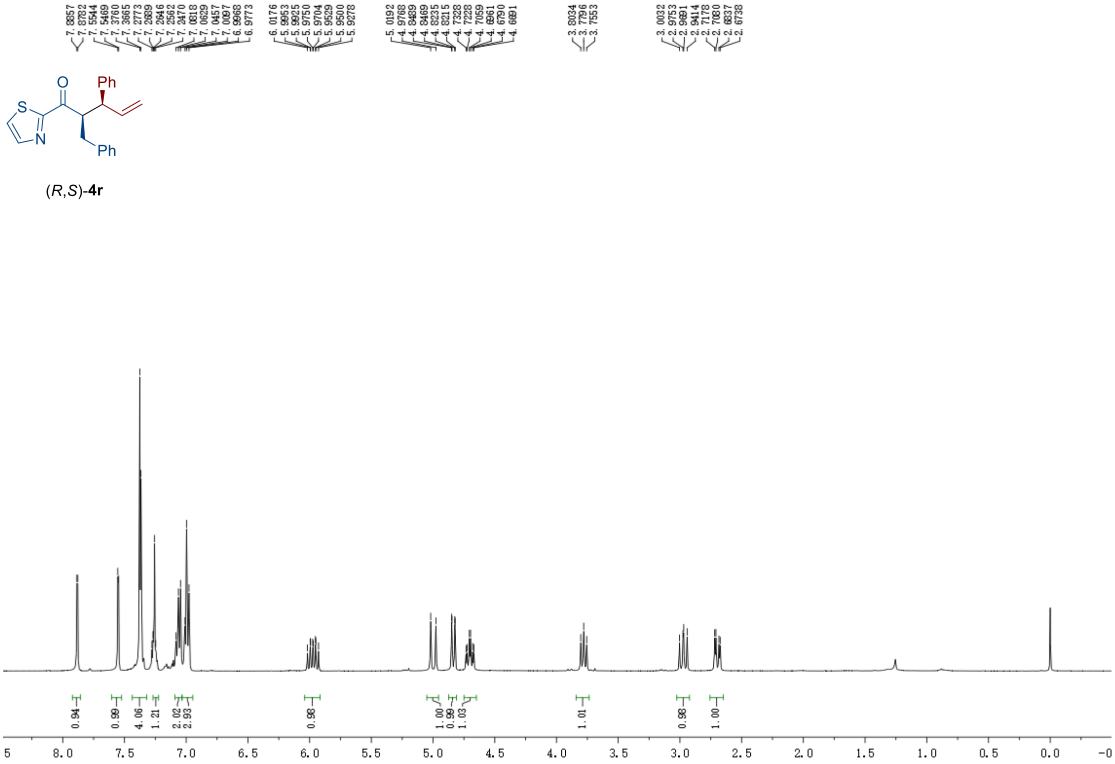

^{13}C NMR spectrum of **4r**

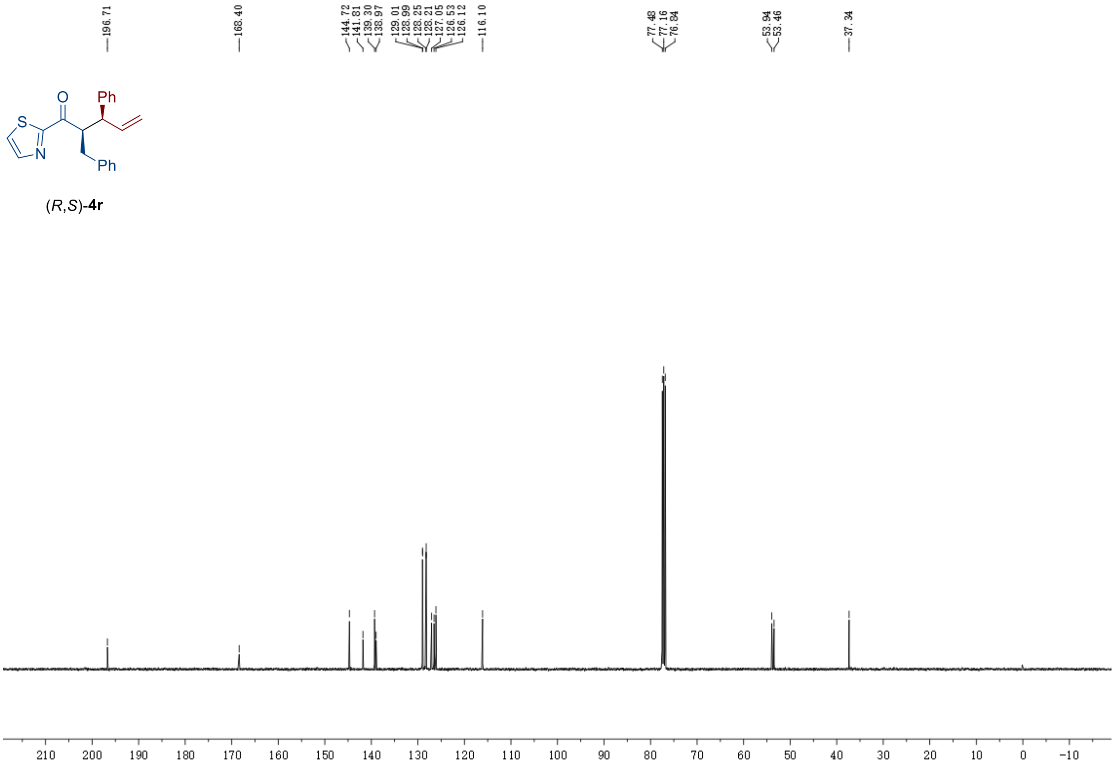

Supplementary Figure 44. NMR spectra of compound **4r**

^{1}H NMR spectrum of **4s**

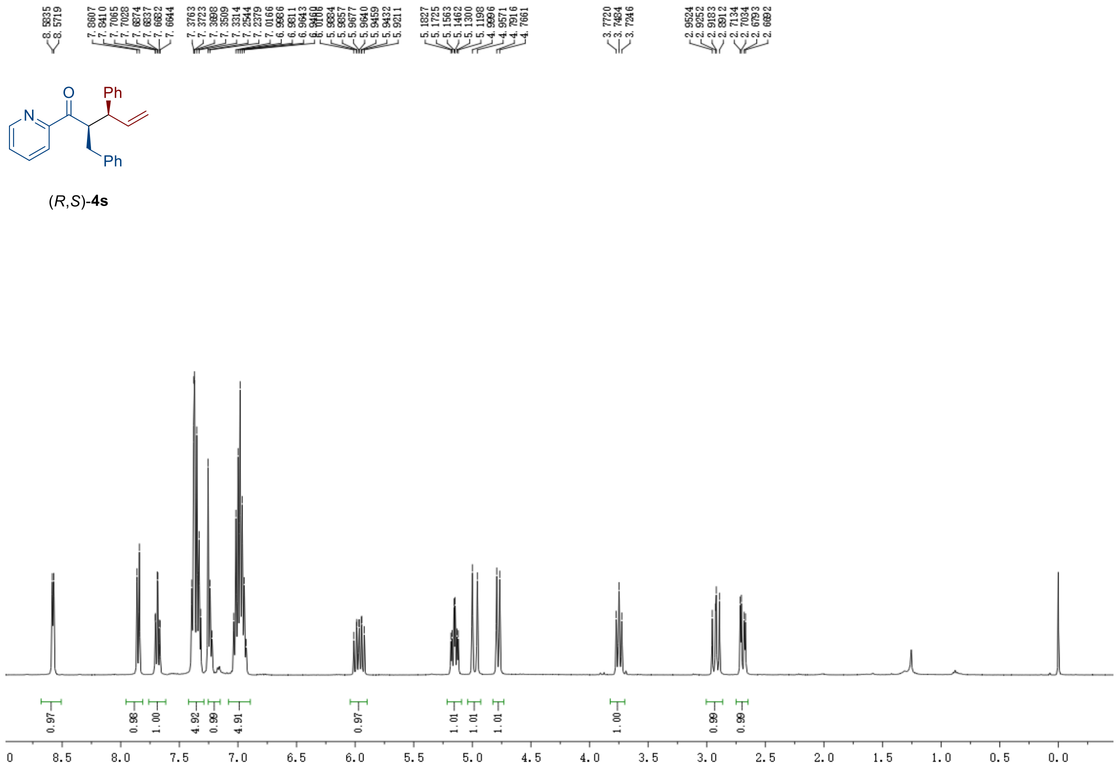

^{13}C NMR spectrum of **4s**

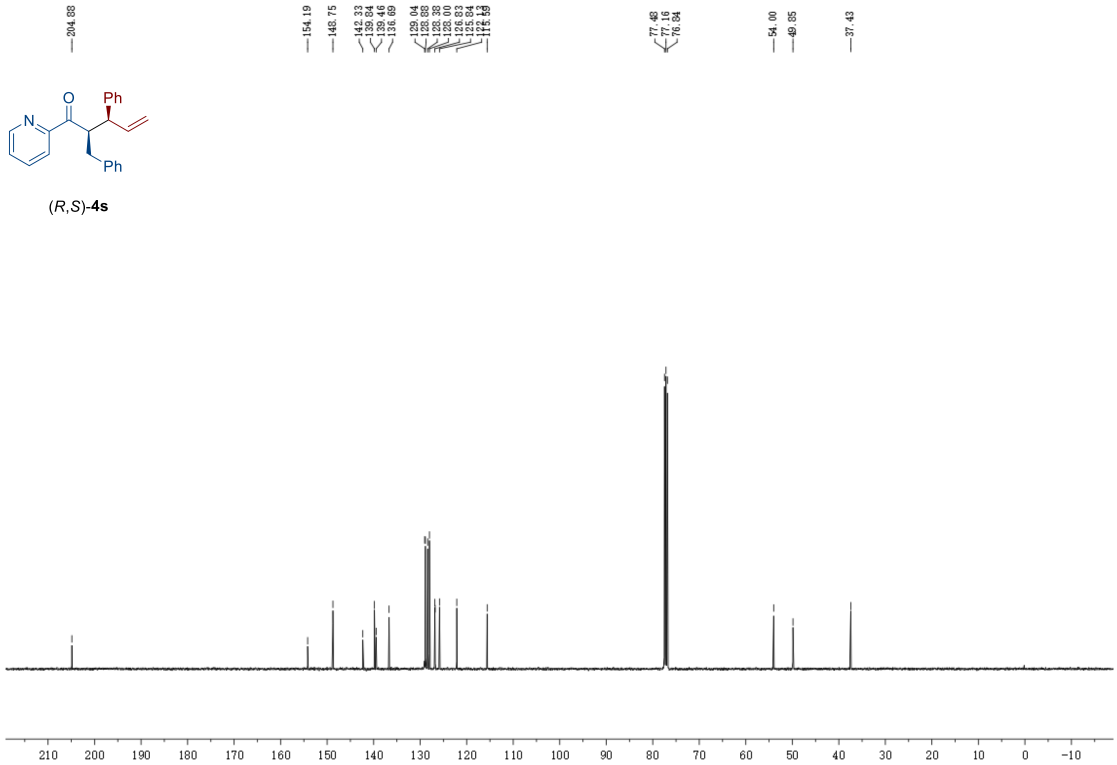

Supplementary Figure 45. NMR spectra of compound **4s**

^{1}H NMR spectrum of **4t**

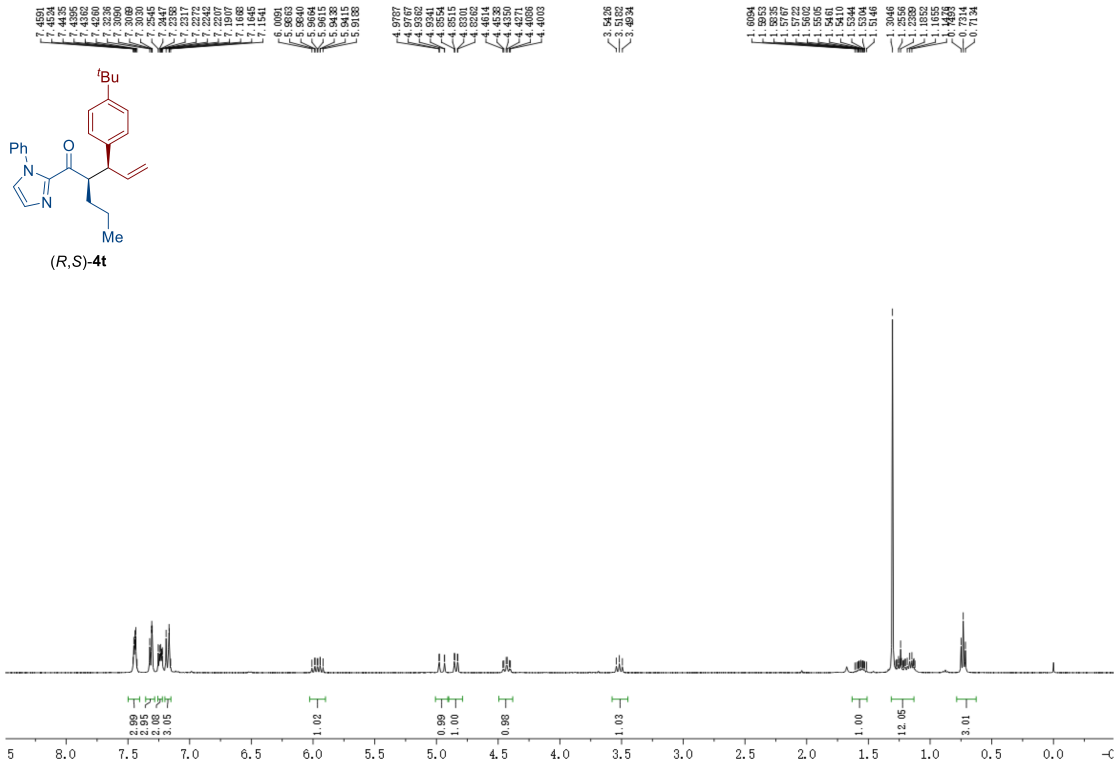

^{13}C NMR spectrum of **4t**

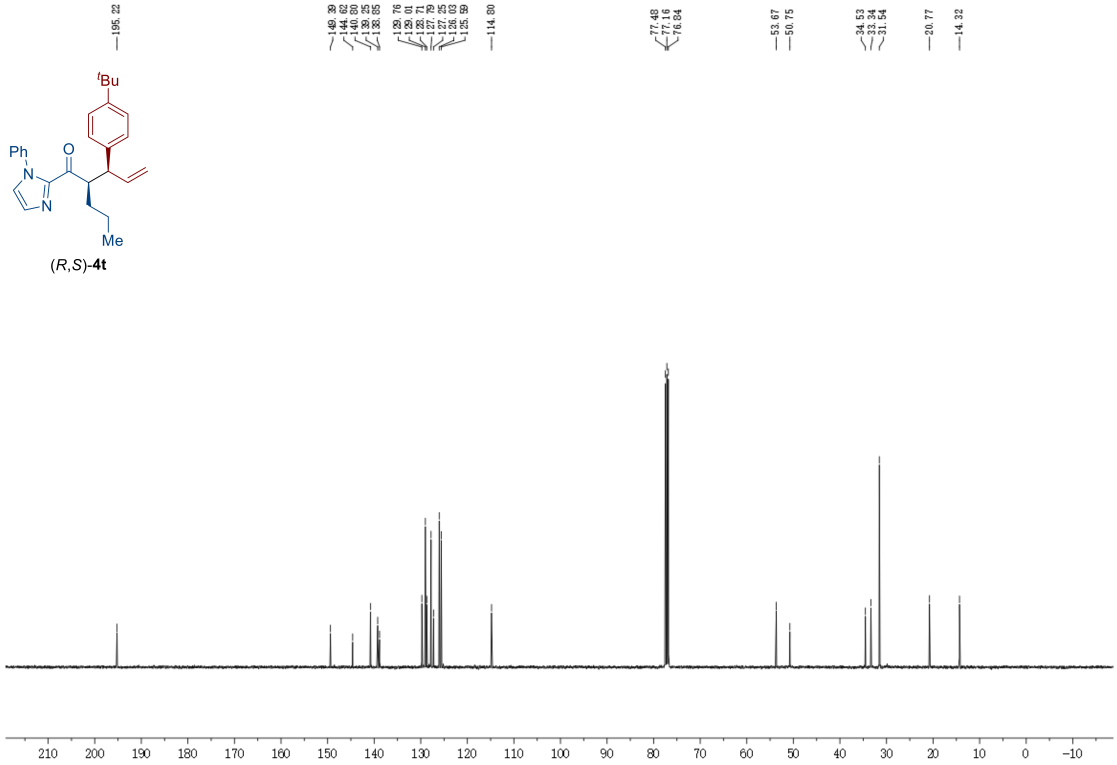

Supplementary Figure 46. NMR spectra of compound **4t**

^{1}H NMR spectrum of **4u**

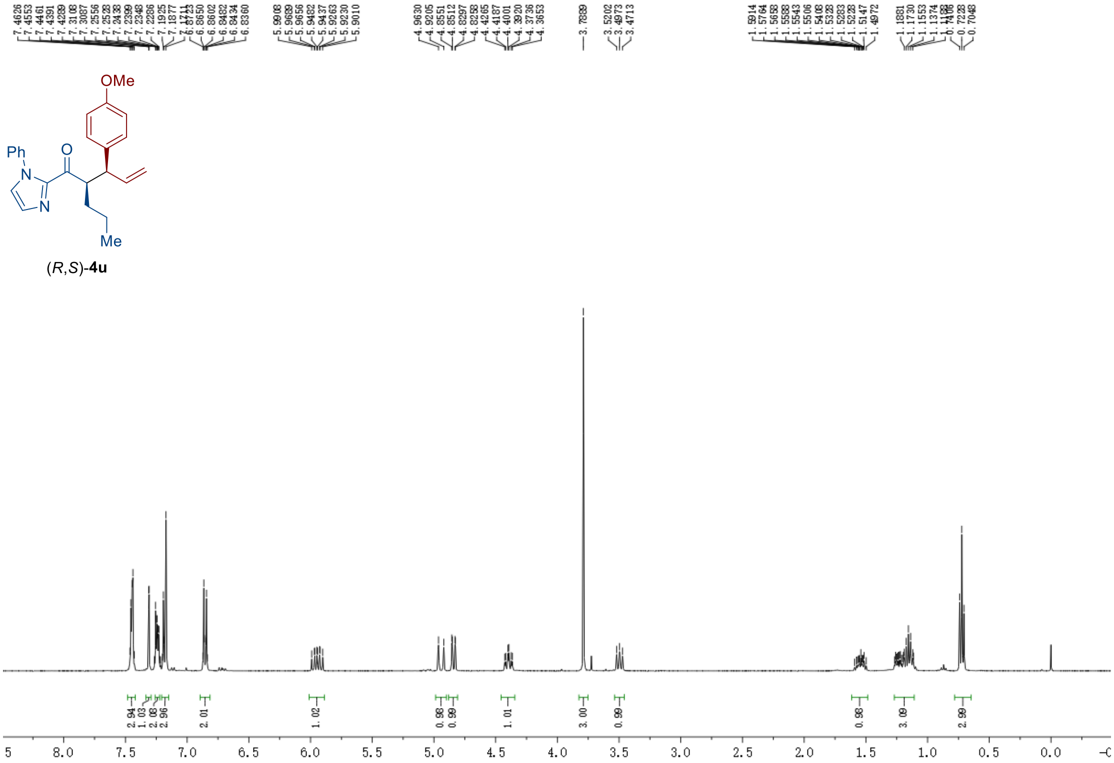

^{13}C NMR spectrum of **4u**

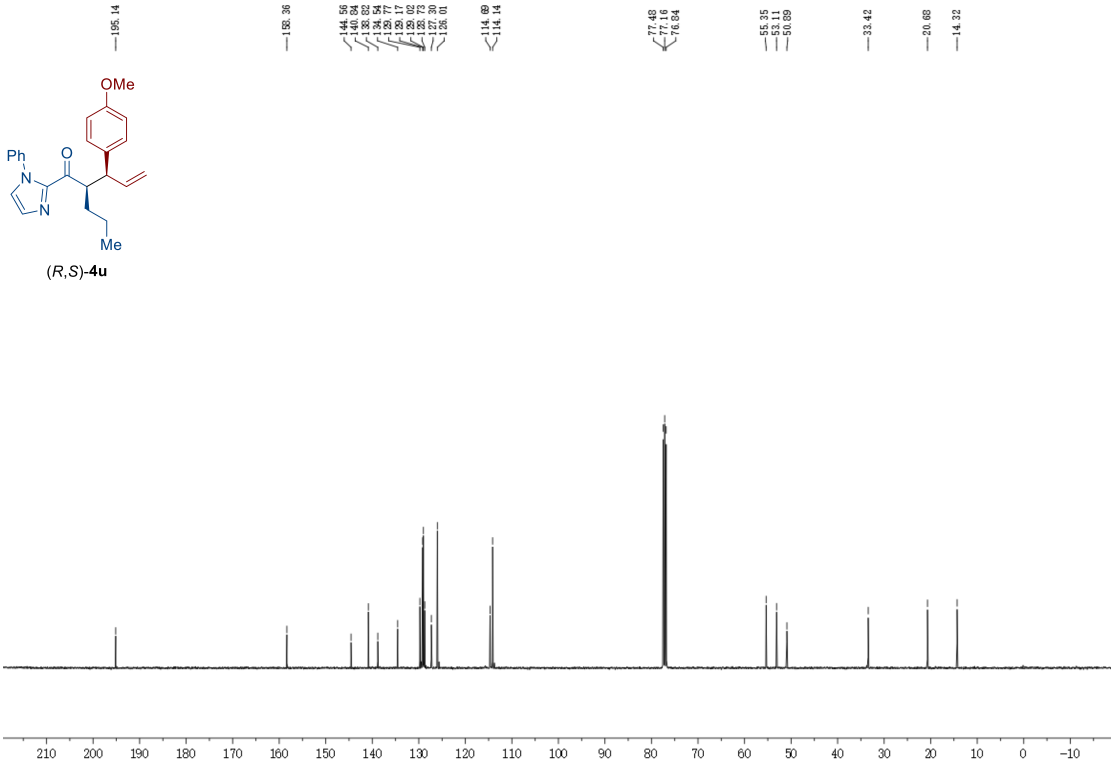

Supplementary Figure 47. NMR spectra of compound **4u**

^{1}H NMR spectrum of **4v**

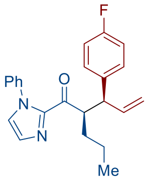

(*R*,*S*)-**4v**

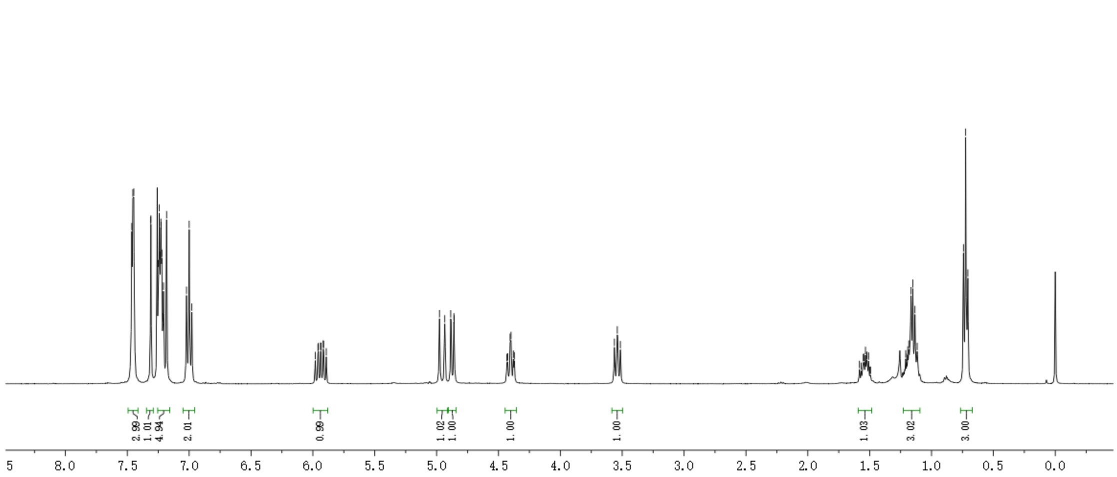

^{13}C NMR spectrum of **4v**

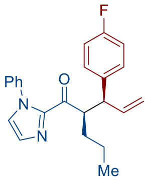

(*R*,*S*)-**4v**

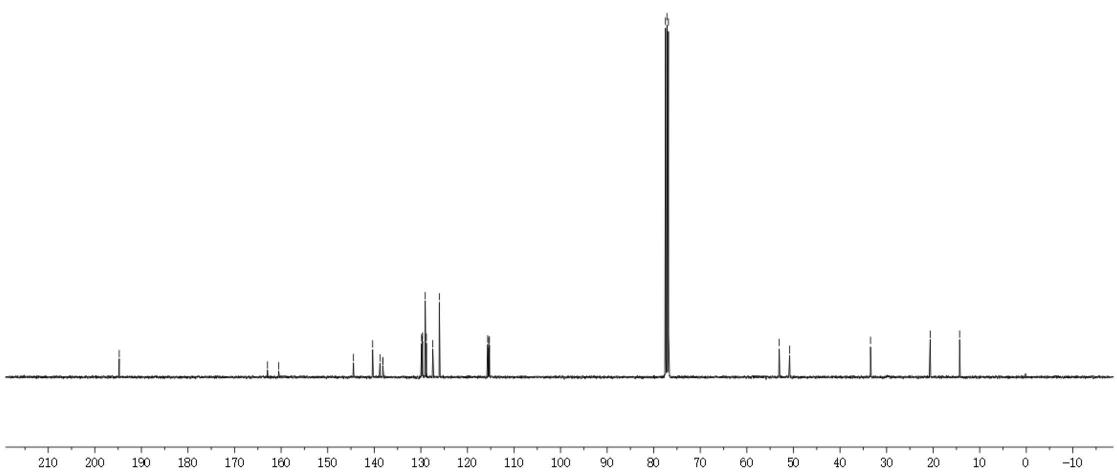

^{19}F NMR spectrum of **4v**

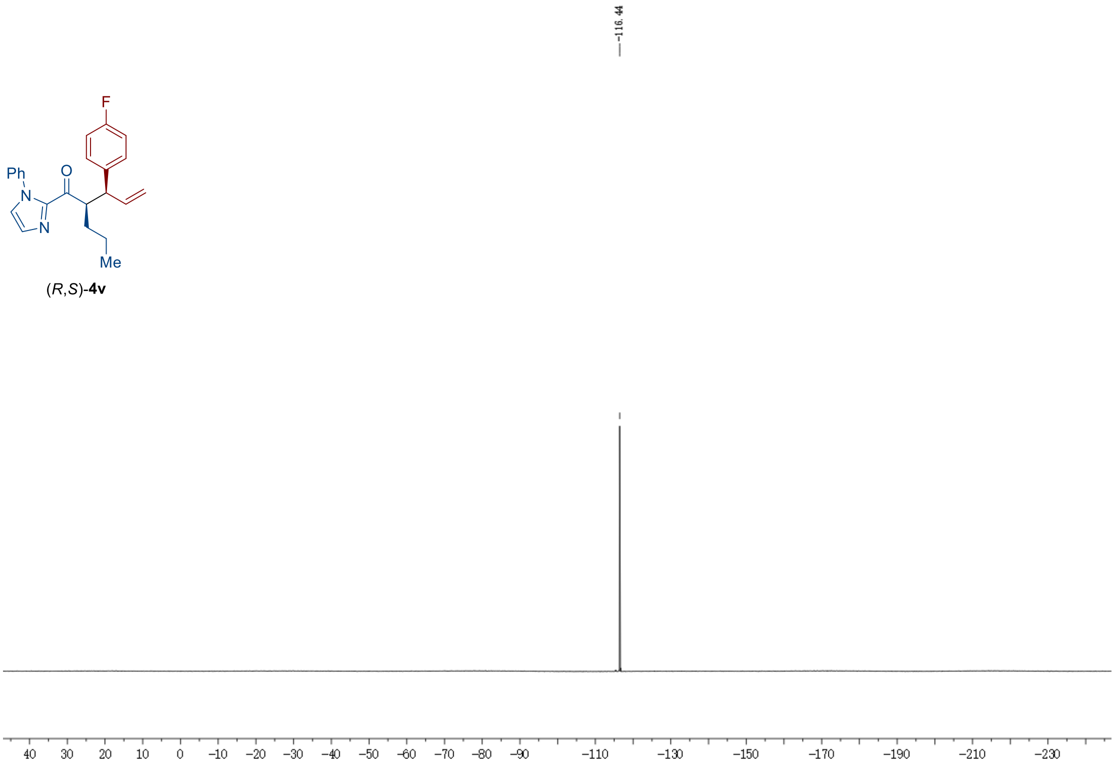

Supplementary Figure 48. NMR spectra of compound **4v**

^{1}H NMR spectrum of **4w**

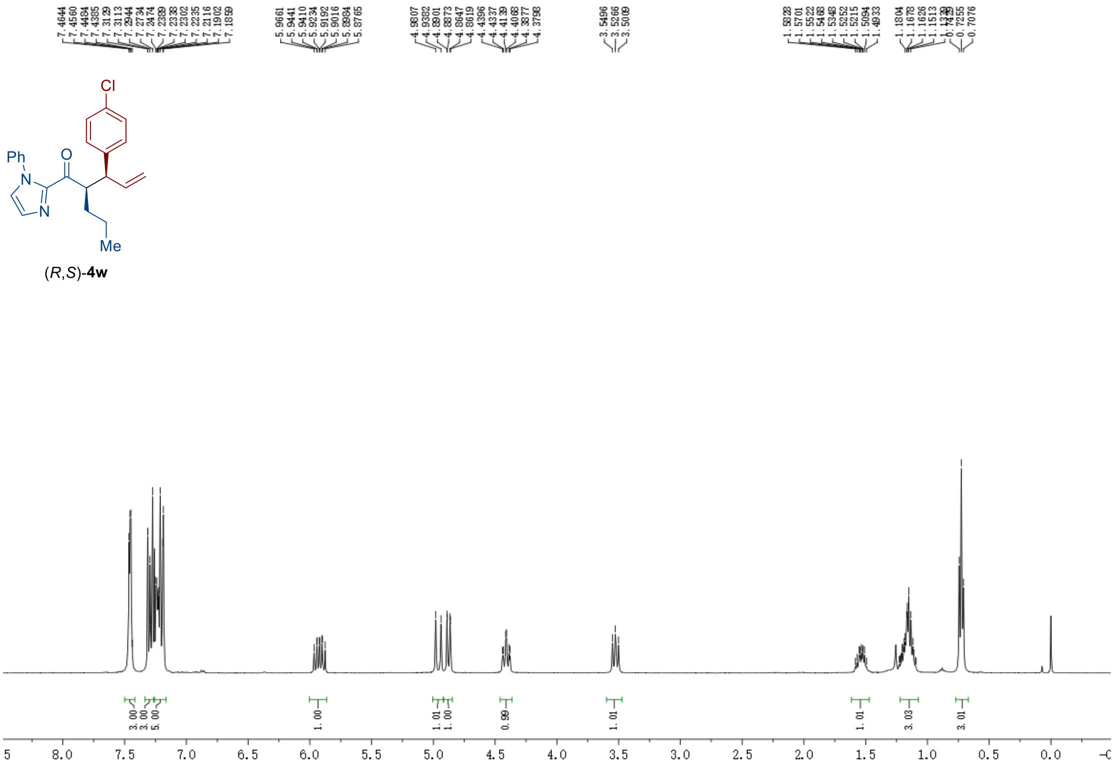

^{13}C NMR spectrum of **4w**

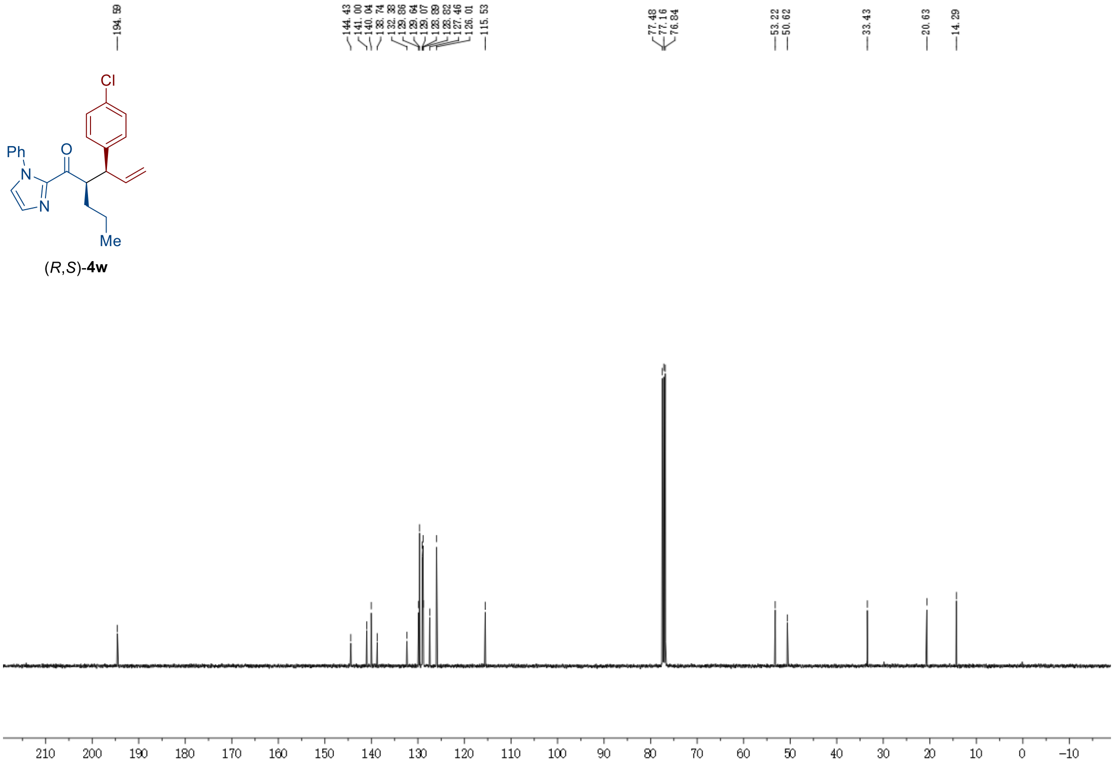

Supplementary Figure 49. NMR spectra of compound **4w**

^{1}H NMR spectrum of **4x**

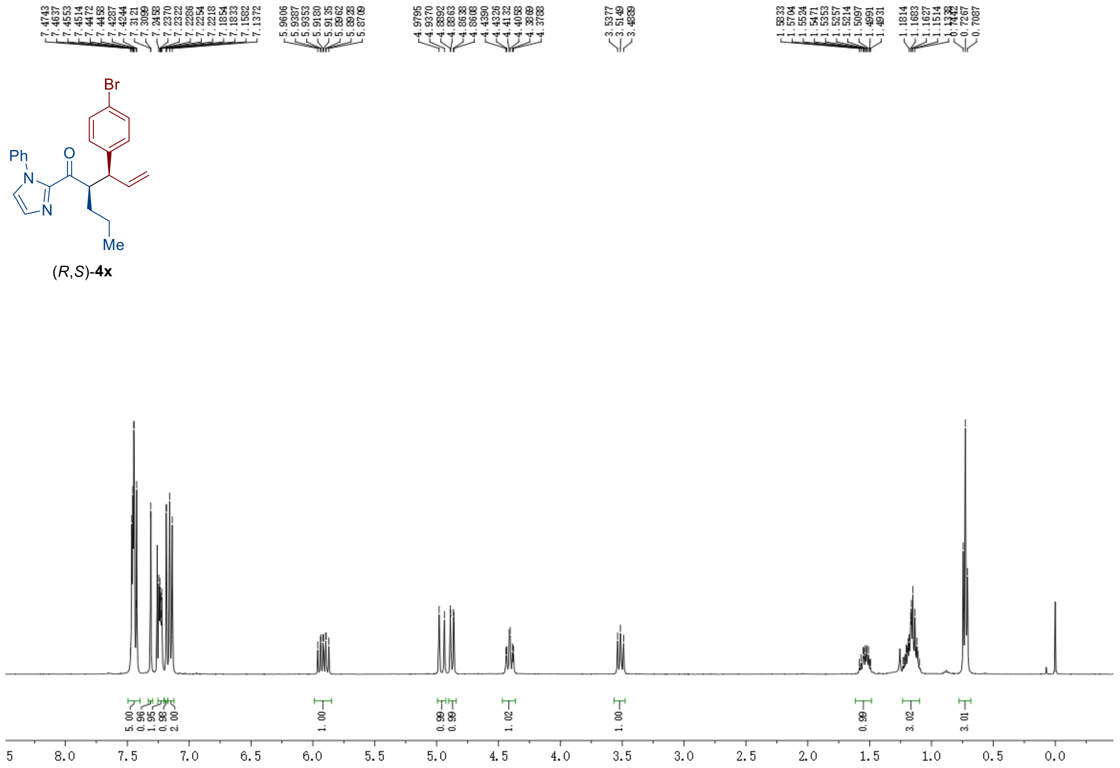

^{13}C NMR spectrum of **4x**

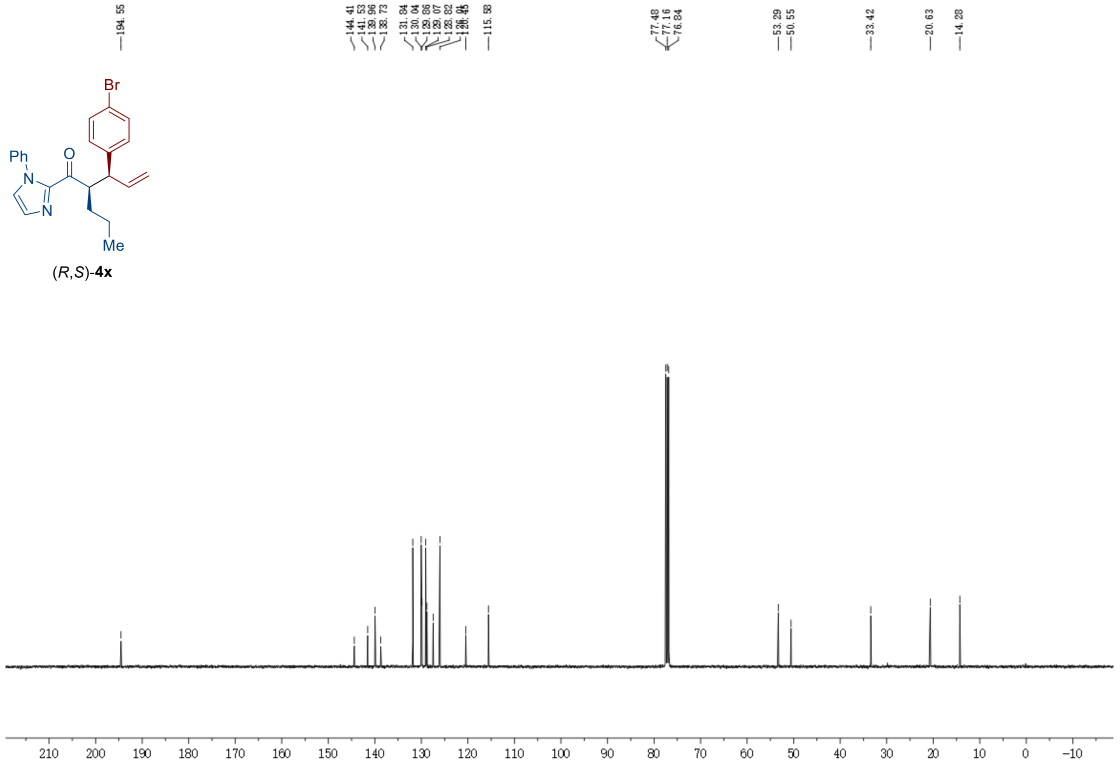

Supplementary Figure 50. NMR spectra of compound **4x**

^{1}H NMR spectrum of **4y**

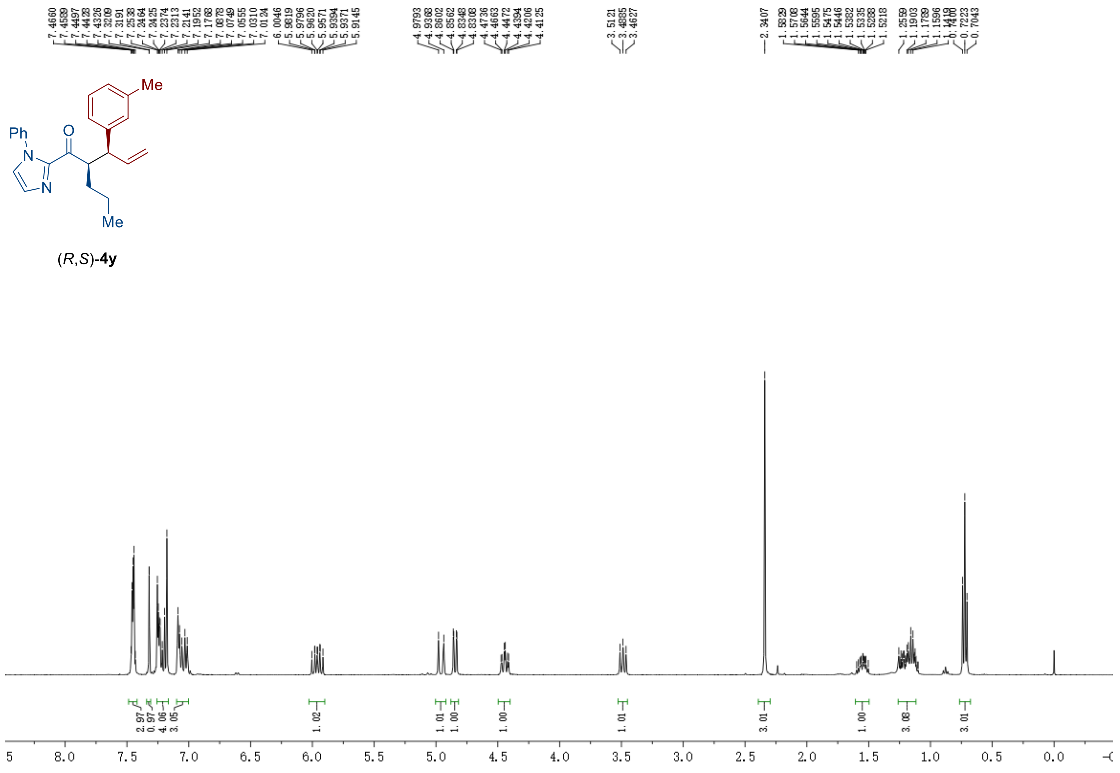

^{13}C NMR spectrum of **4y**

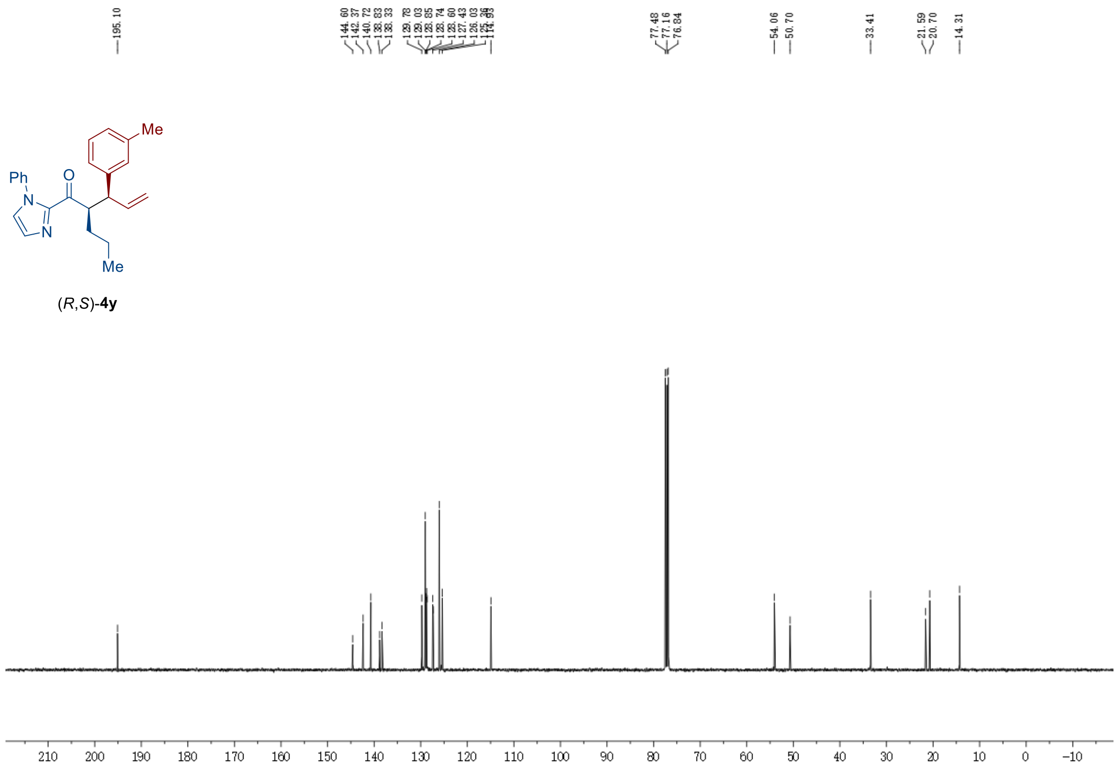

Supplementary Figure 51. NMR spectra of compound **4y**

^{1}H NMR spectrum of **4z**

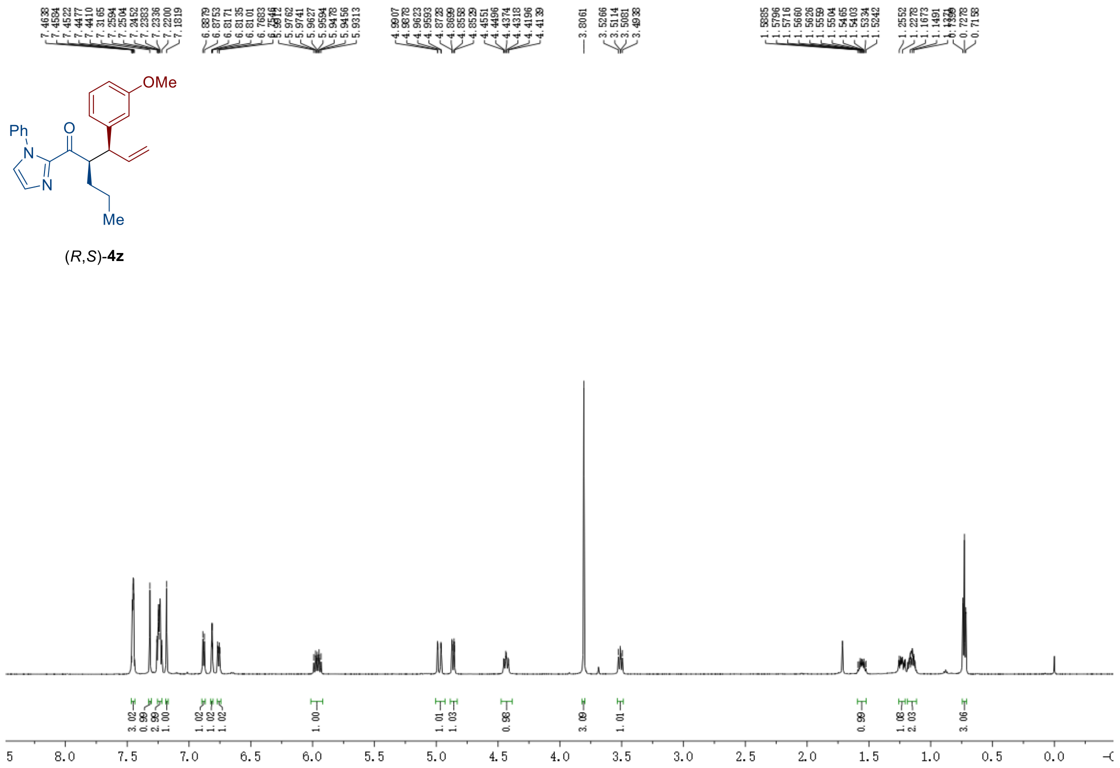

^{13}C NMR spectrum of **4z**

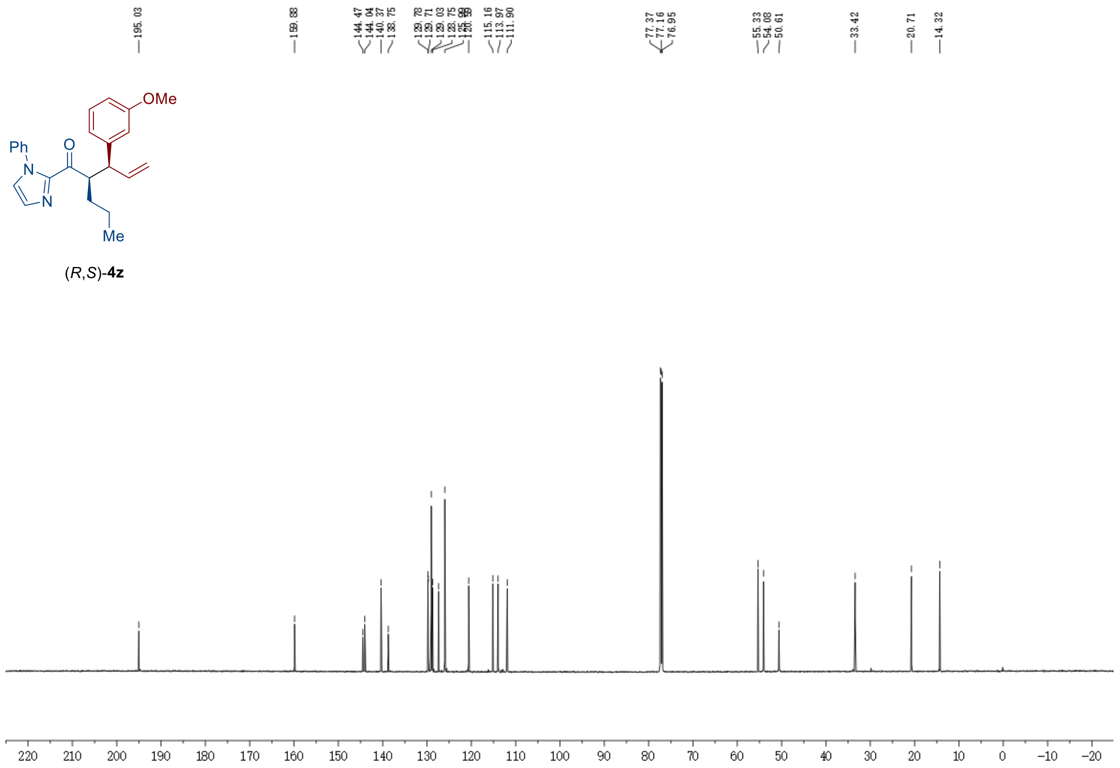

Supplementary Figure 52. NMR spectra of compound **4z**

^{1}H NMR spectrum of **4aa**

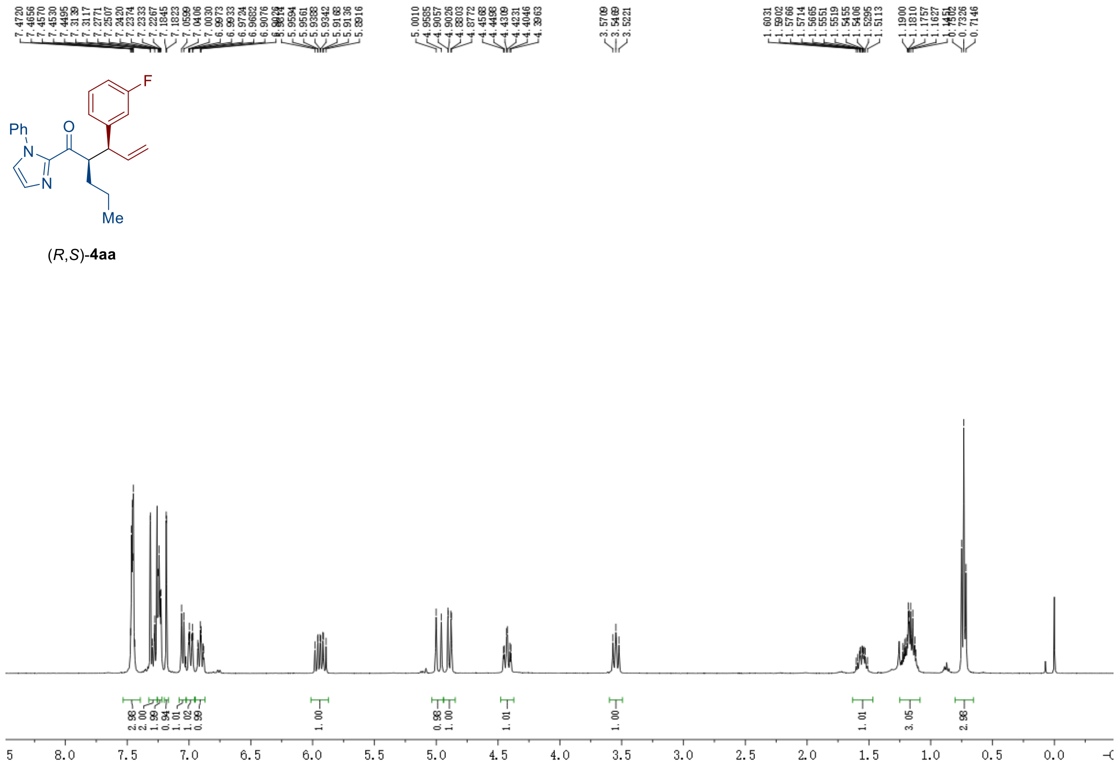

^{13}C NMR spectrum of **4aa**

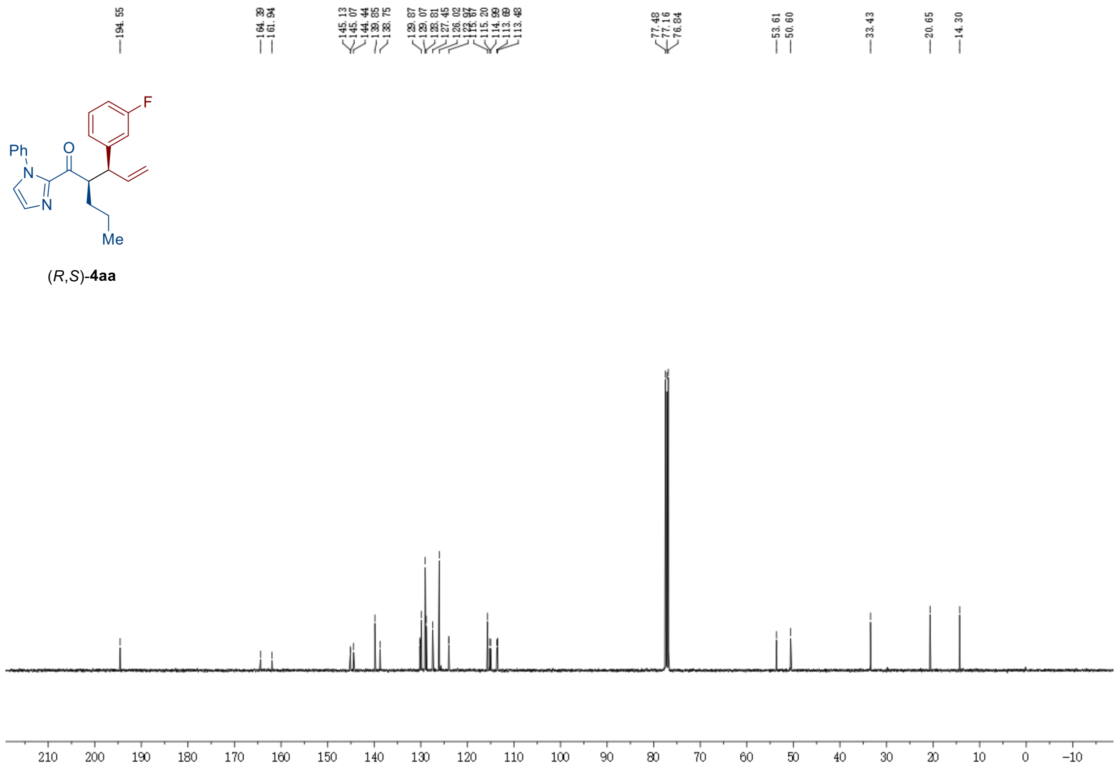

^{19}F NMR spectrum of **4aa**

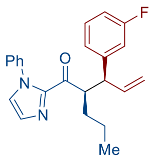
(*R*,*S*)-**4aa**

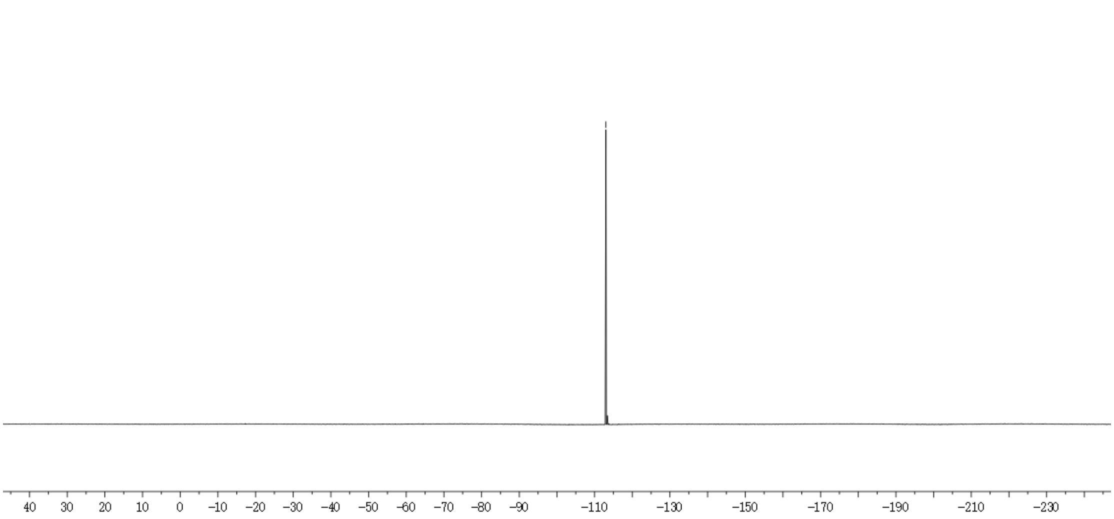

Supplementary Figure 53. NMR spectra of compound **4aa**

^{1}H NMR spectrum of **4ab**

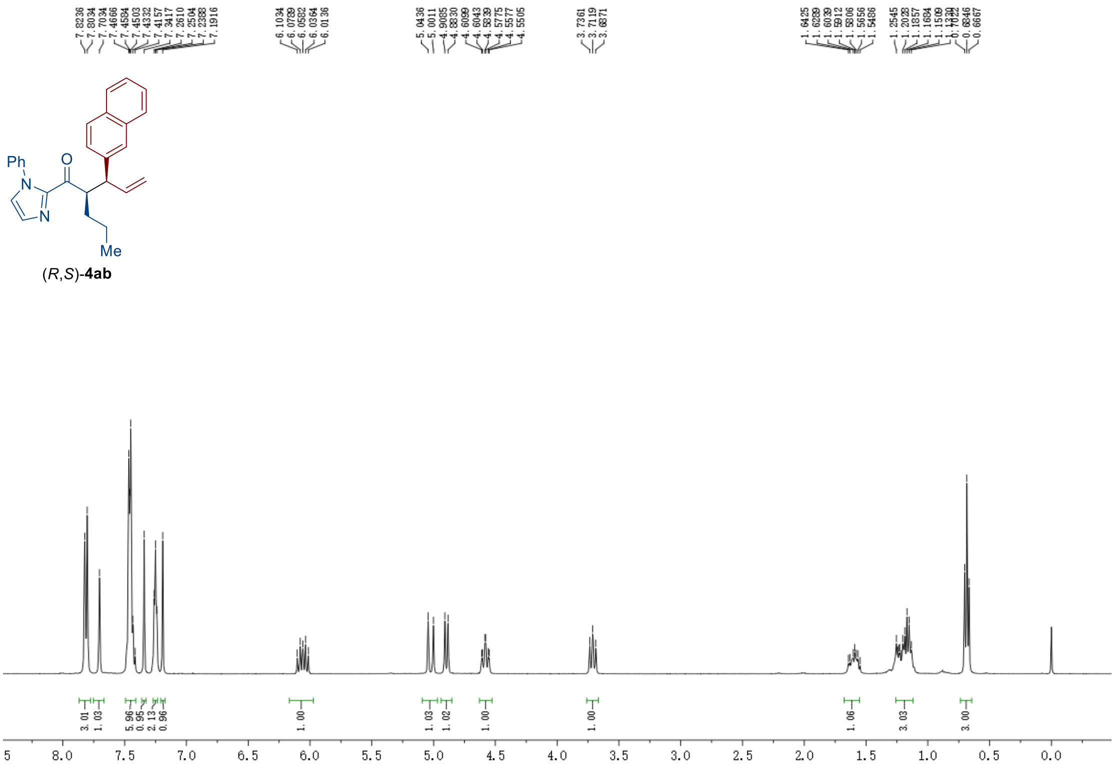

^{13}C NMR spectrum of **4ab**

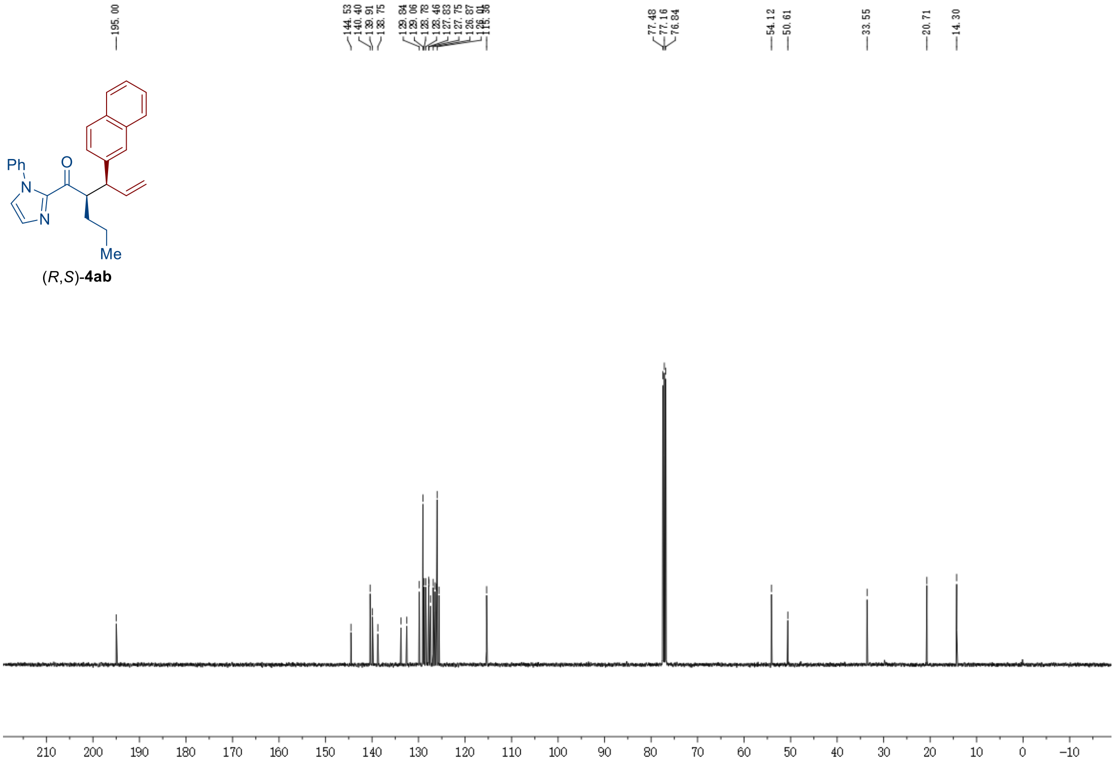

Supplementary Figure 54. NMR spectra of compound **4ab**

^{1}H NMR spectrum of **4ac**

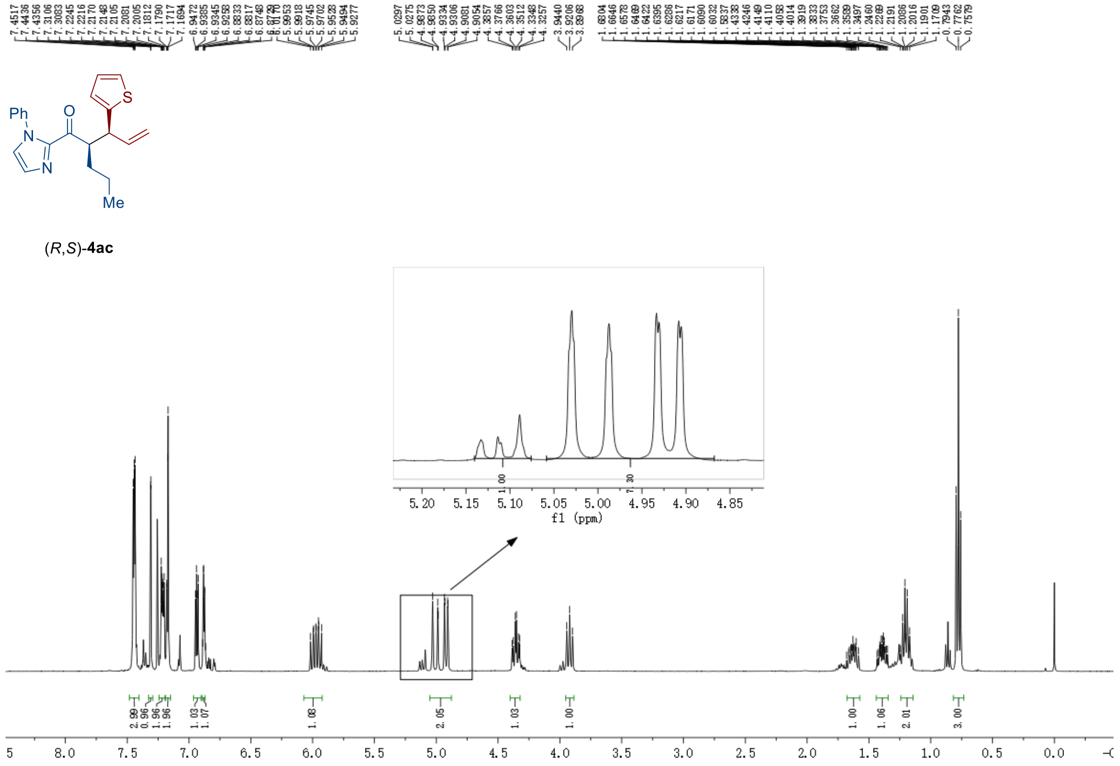

^{13}C NMR spectrum of **4ac**

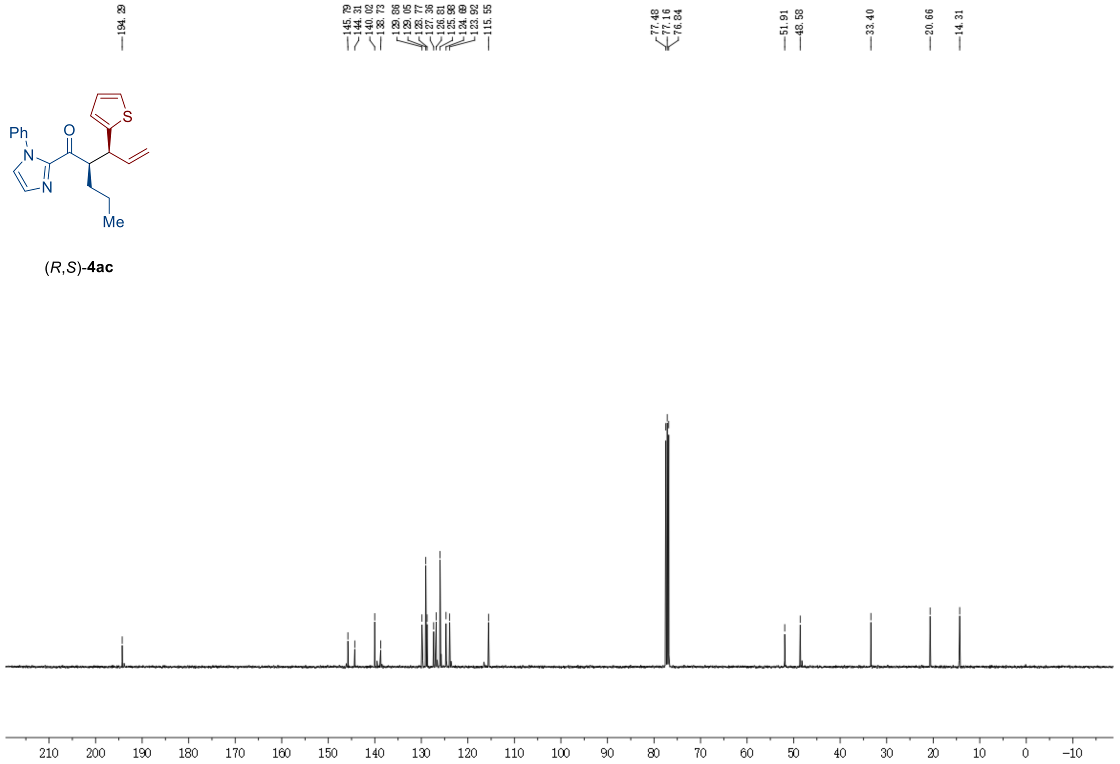

Supplementary Figure 55. NMR spectra of compound **4ac**

^{1}H NMR spectrum of **4ad**

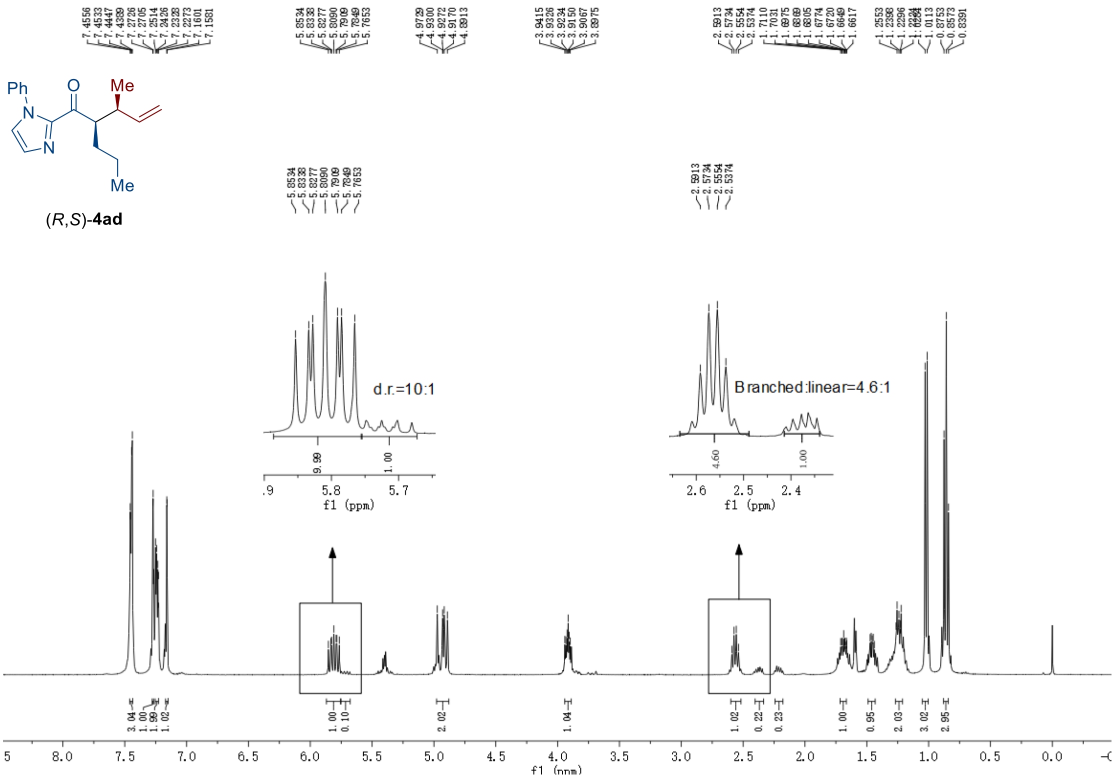

^{13}C NMR spectrum of **4ad**

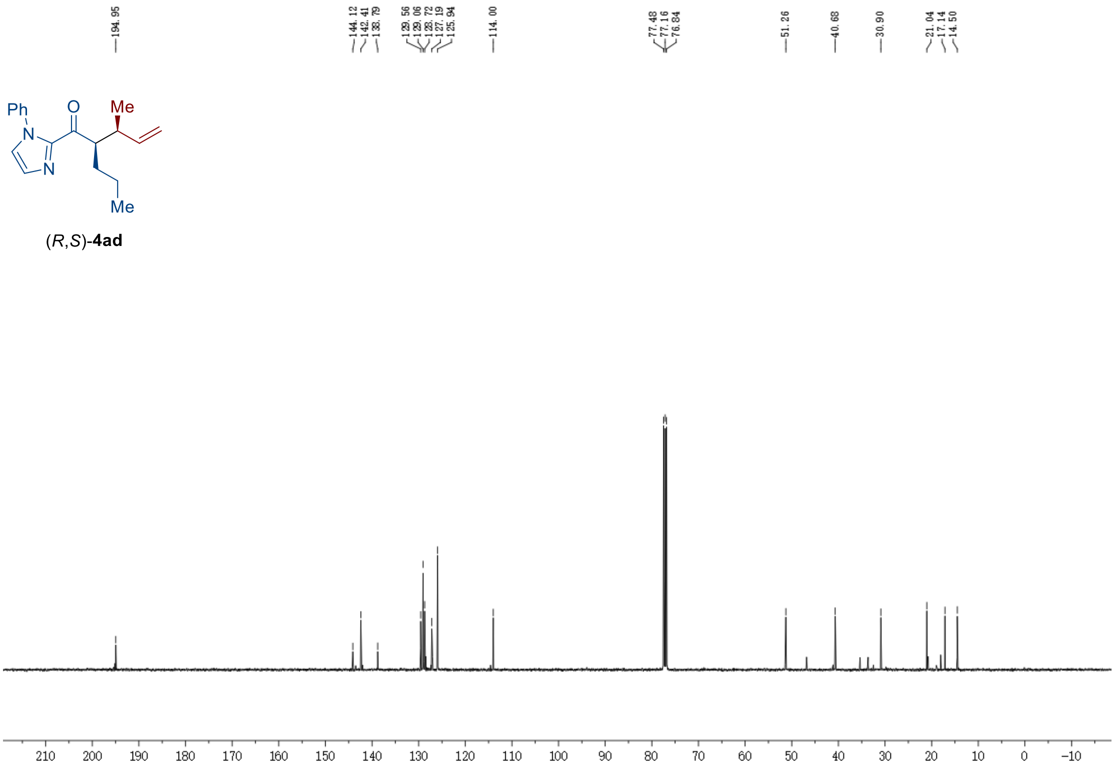

Supplementary Figure 56. NMR spectra of compound **4ad**

^{1}H NMR spectrum of **4ae**

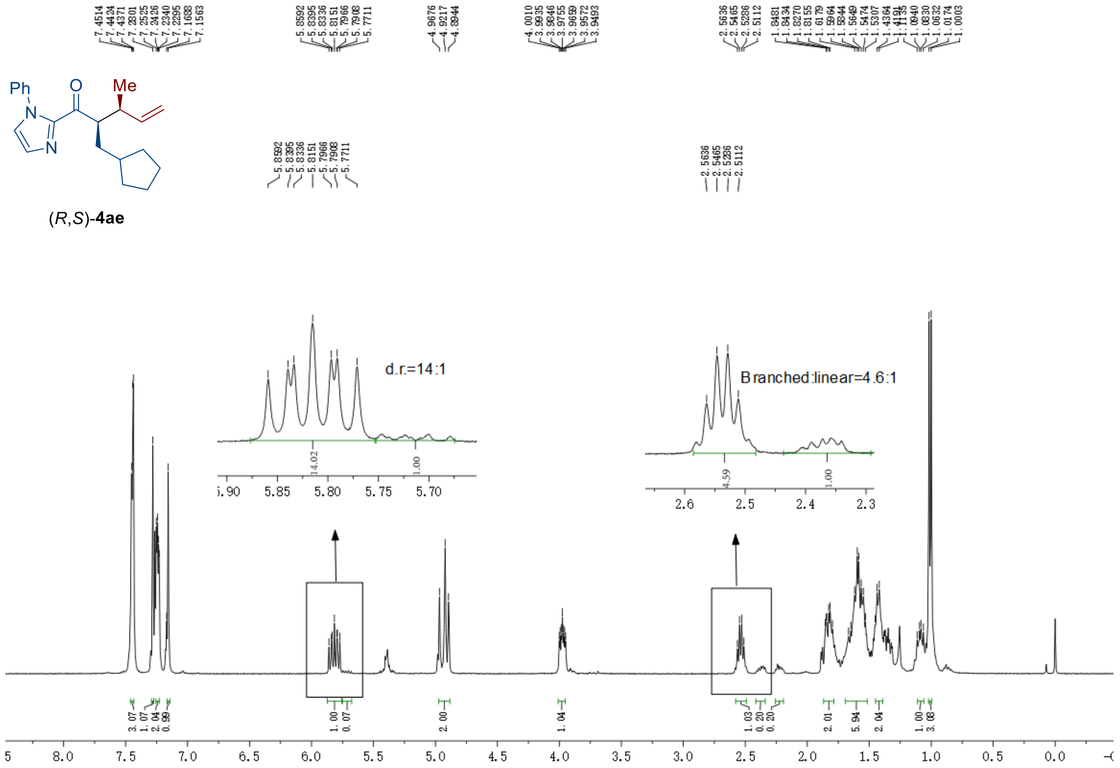

^{13}C NMR spectrum of **4ae**

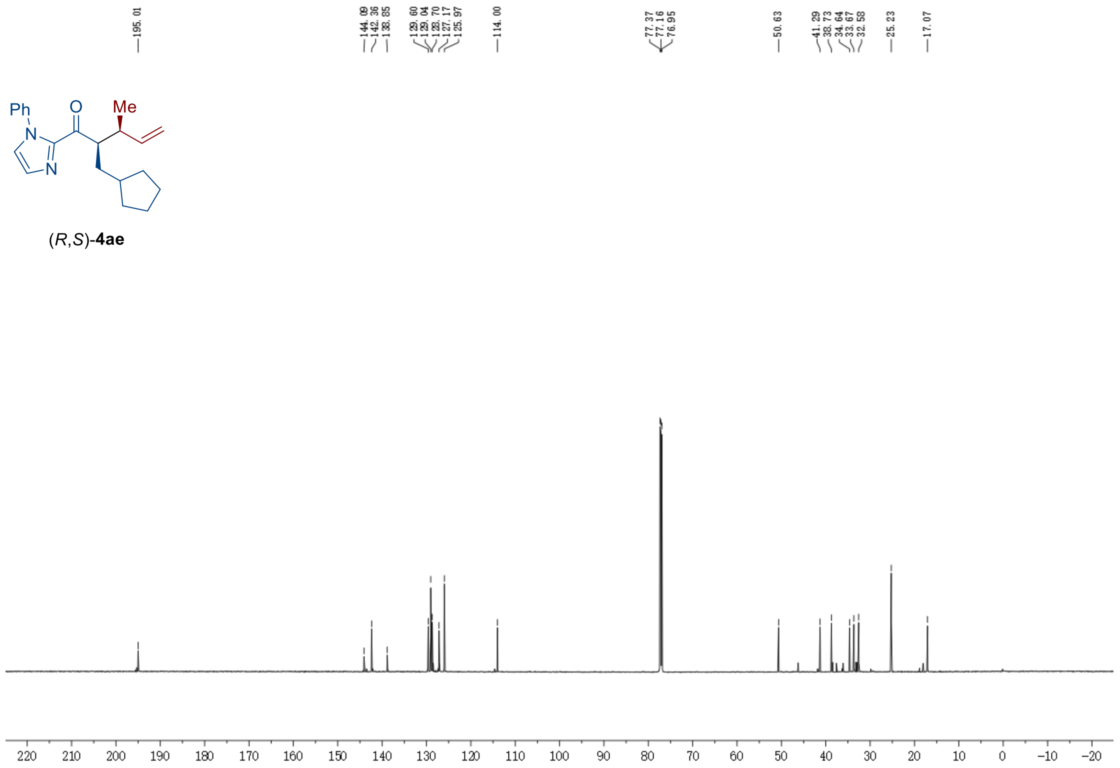

Supplementary Figure 57. NMR spectra of compound **4ae**

^{1}H NMR spectrum of (*R*,*S*)-**4af**

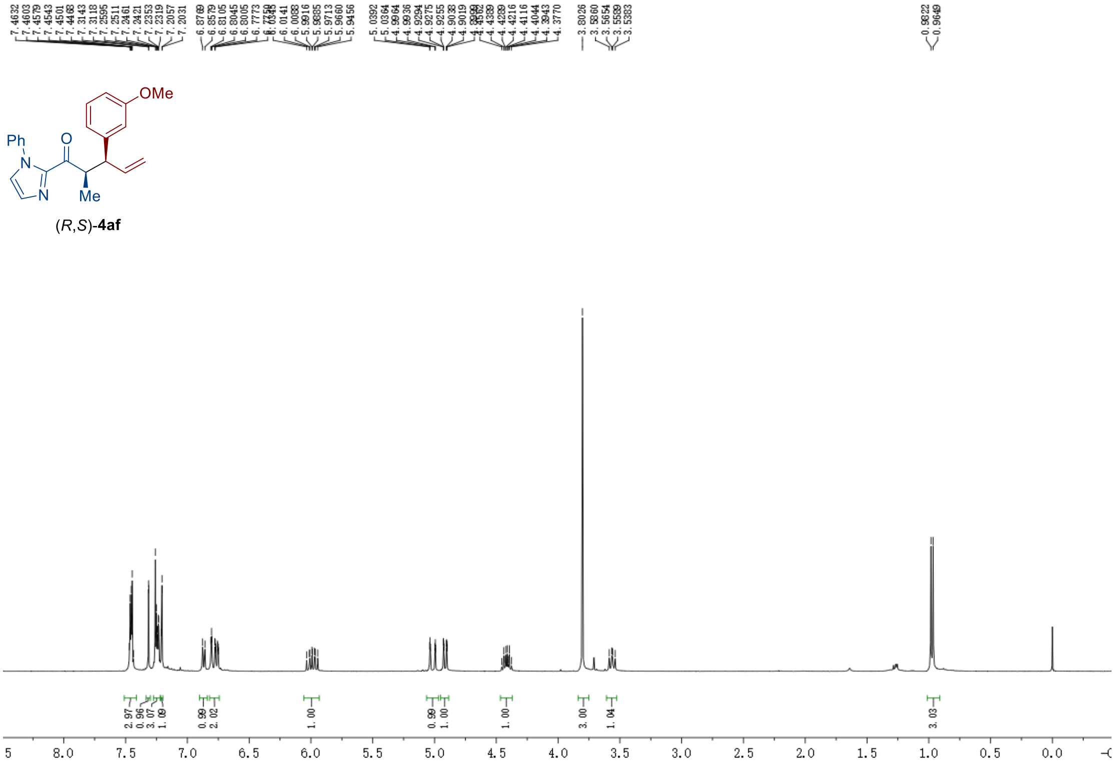

^{13}C NMR spectrum of (*R*,*S*)-**4af**

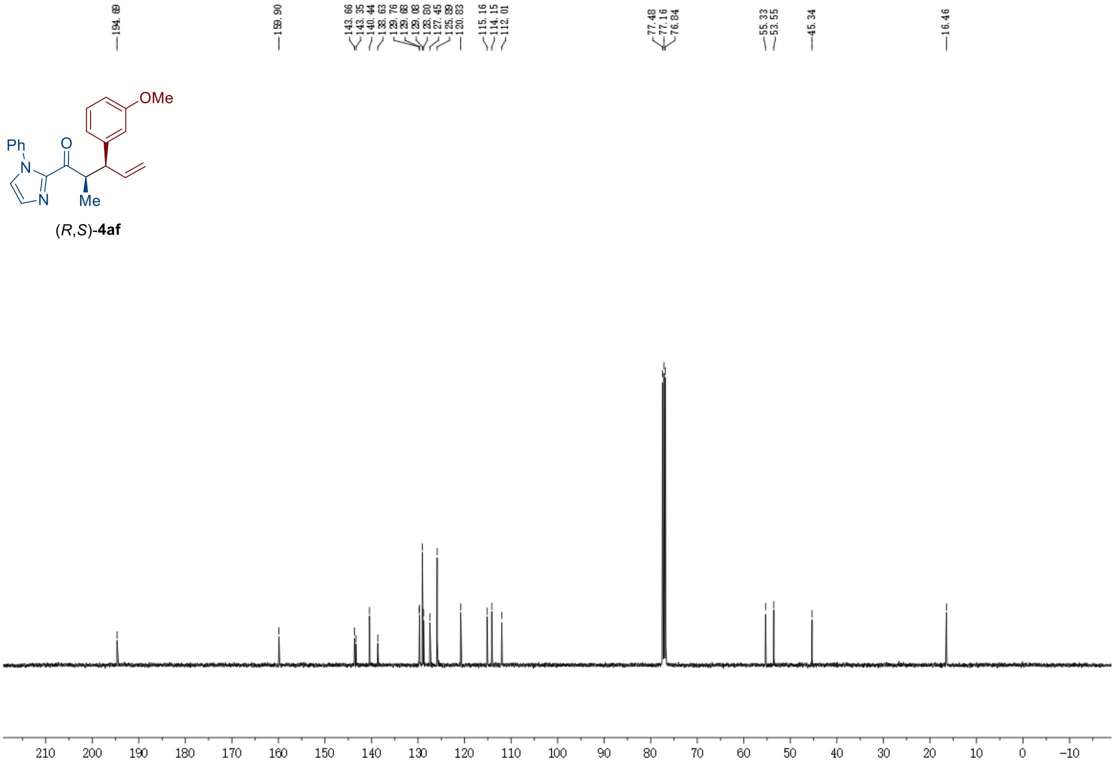

Supplementary Figure 58. NMR spectra of compound (*R*,*S*)-**4af**

^{1}H NMR spectrum of (*S*,*R*)-**4af**

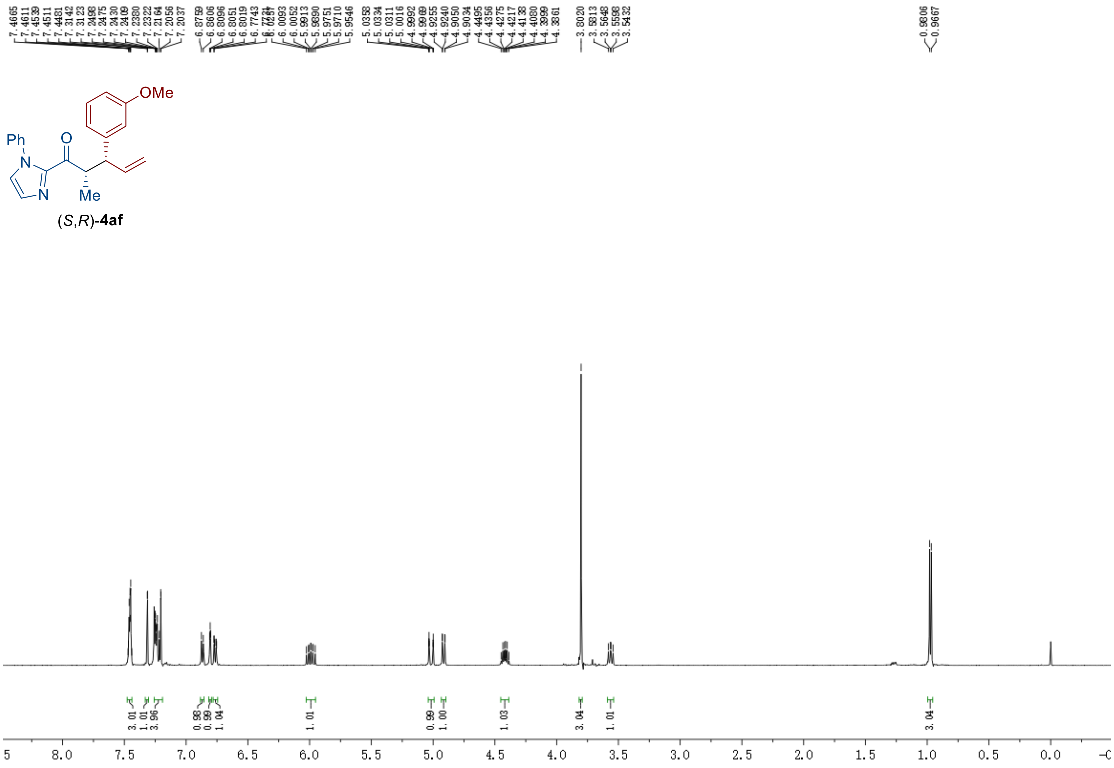

^{13}C NMR spectrum of (*S*,*R*)-**4af**

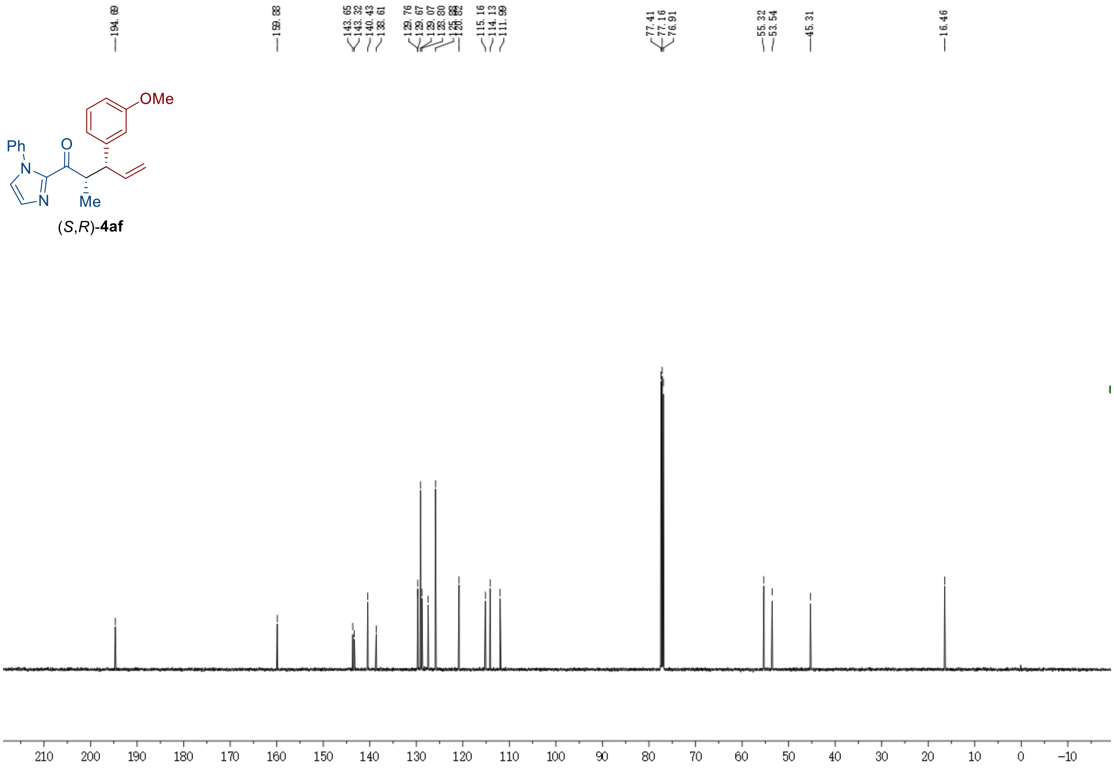

Supplementary Figure 59. NMR spectra of compound (*S*,*R*)-**4af**

^{1}H NMR spectrum of (*S*,*S*)-**4af**

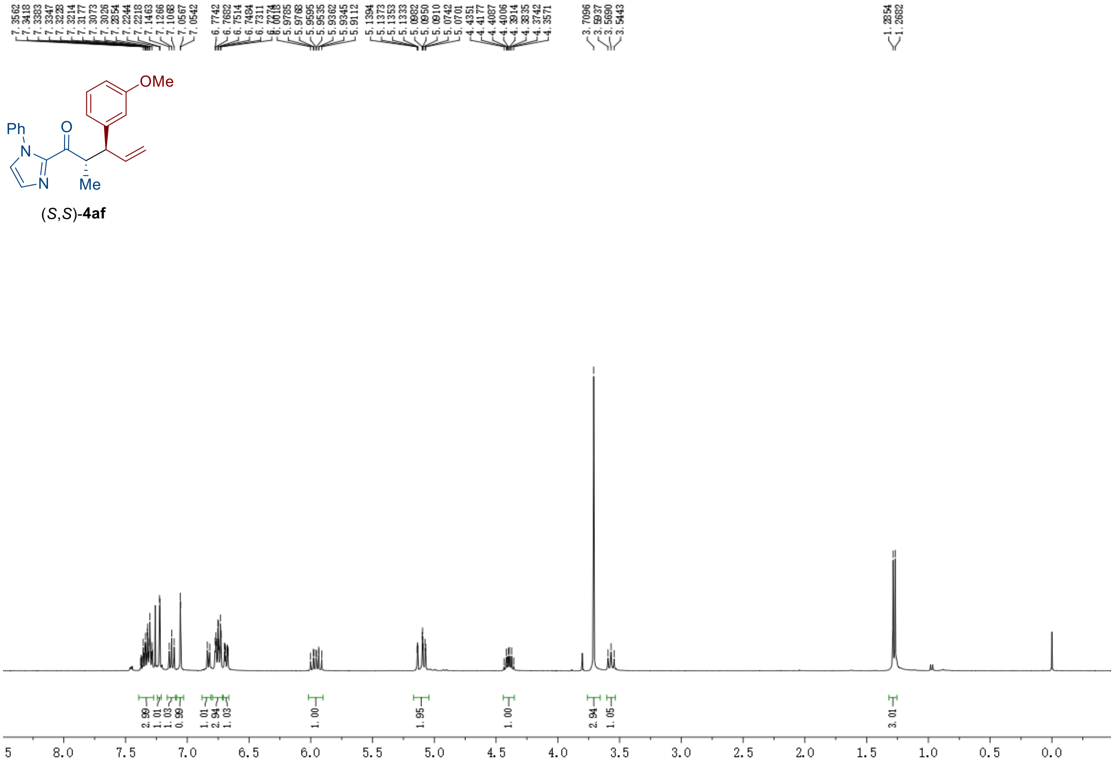

^{13}C NMR spectrum of (*S*,*S*)-**4af**

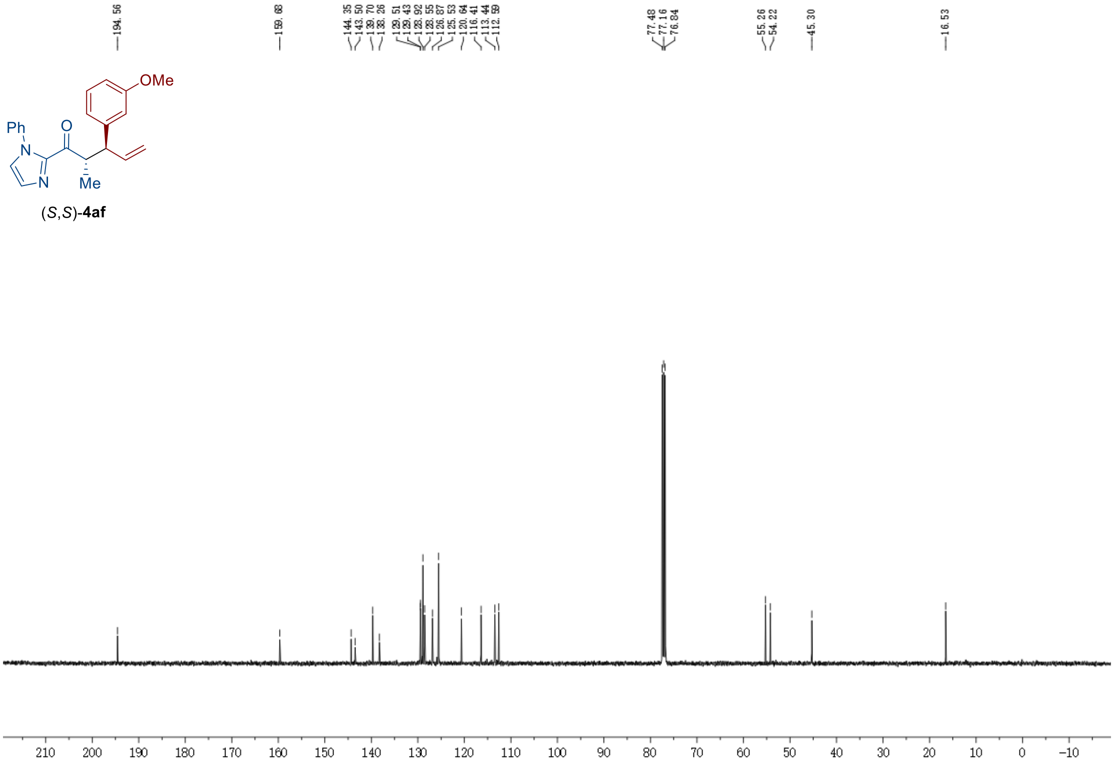

Supplementary Figure 60. NMR spectra of compound (*S*,*S*)-**4af**

^{1}H NMR spectrum of (*R*,*R*)-**4af**

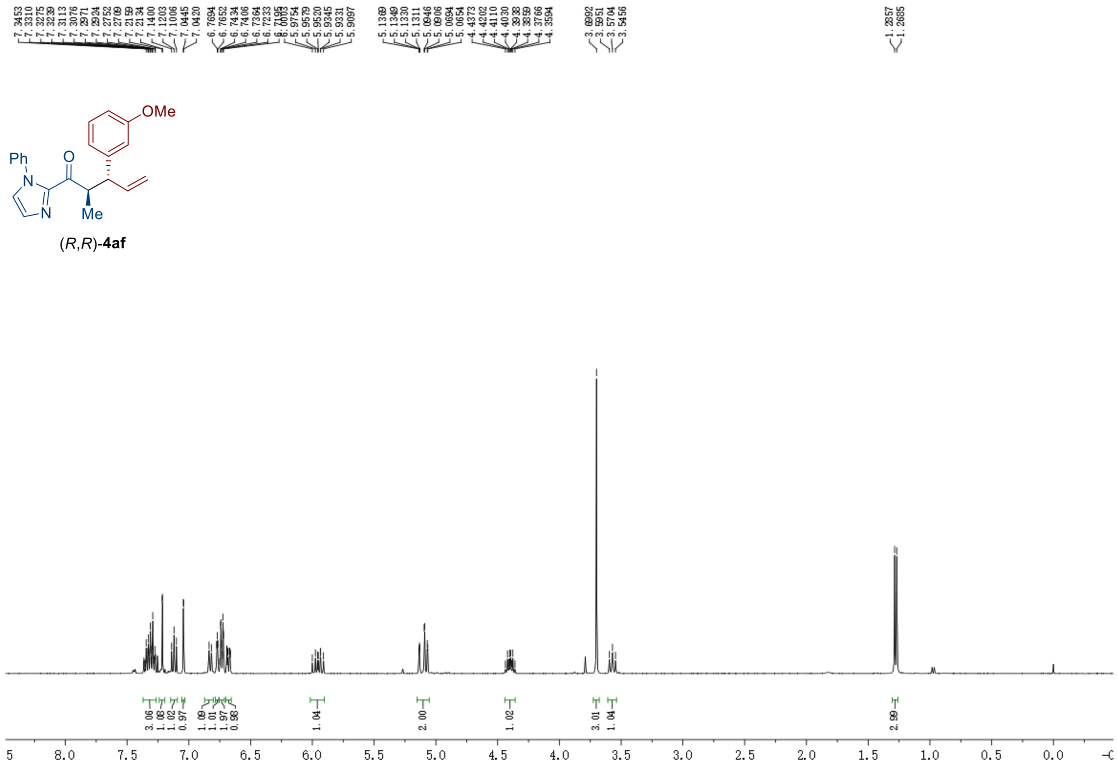

^{13}C NMR spectrum of (*R*,*R*)-**4af**

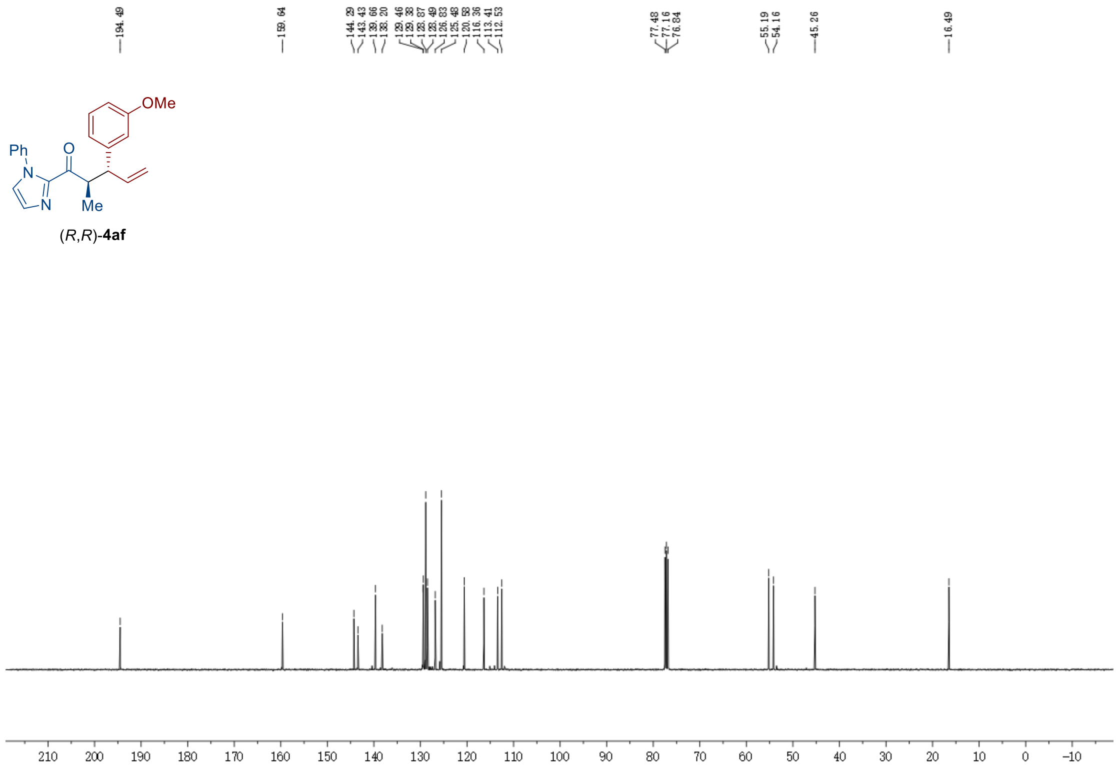

Supplementary Figure 61. NMR spectra of compound (*R*,*R*)-**4af**

^{1}H NMR spectrum of **7**

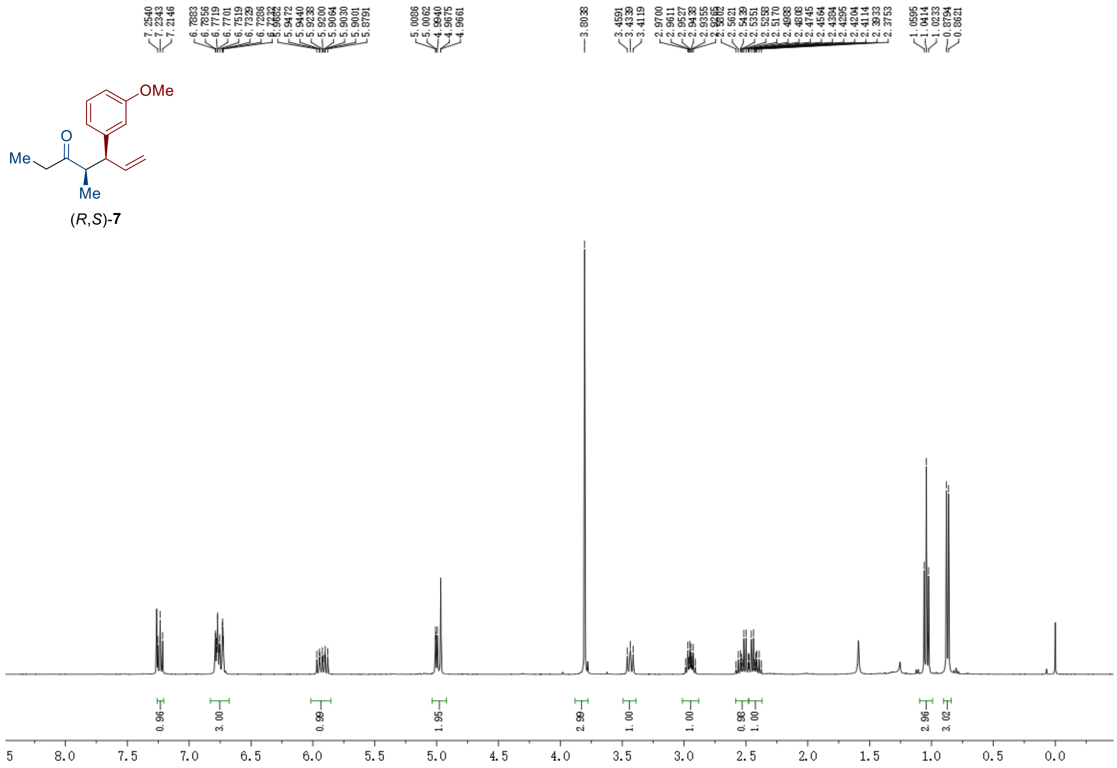

^{13}C NMR spectrum of **7**

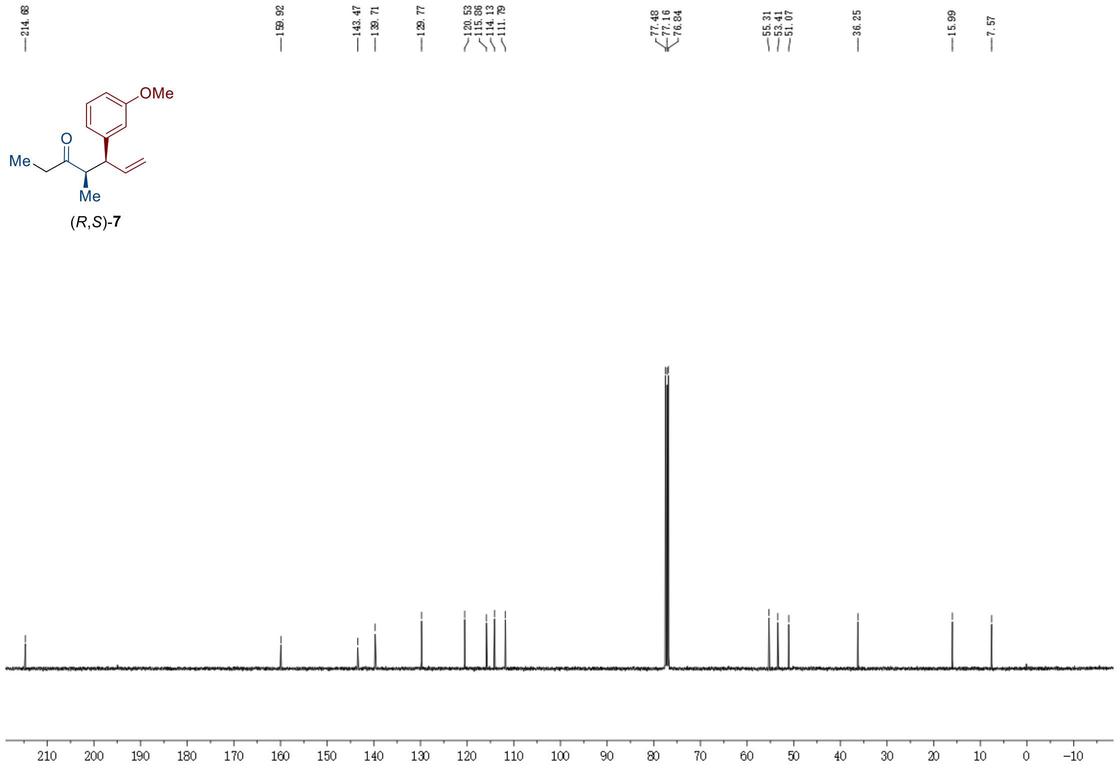

Supplementary Figure 62. NMR spectra of compound **7**

^{1}H NMR spectrum of **8**

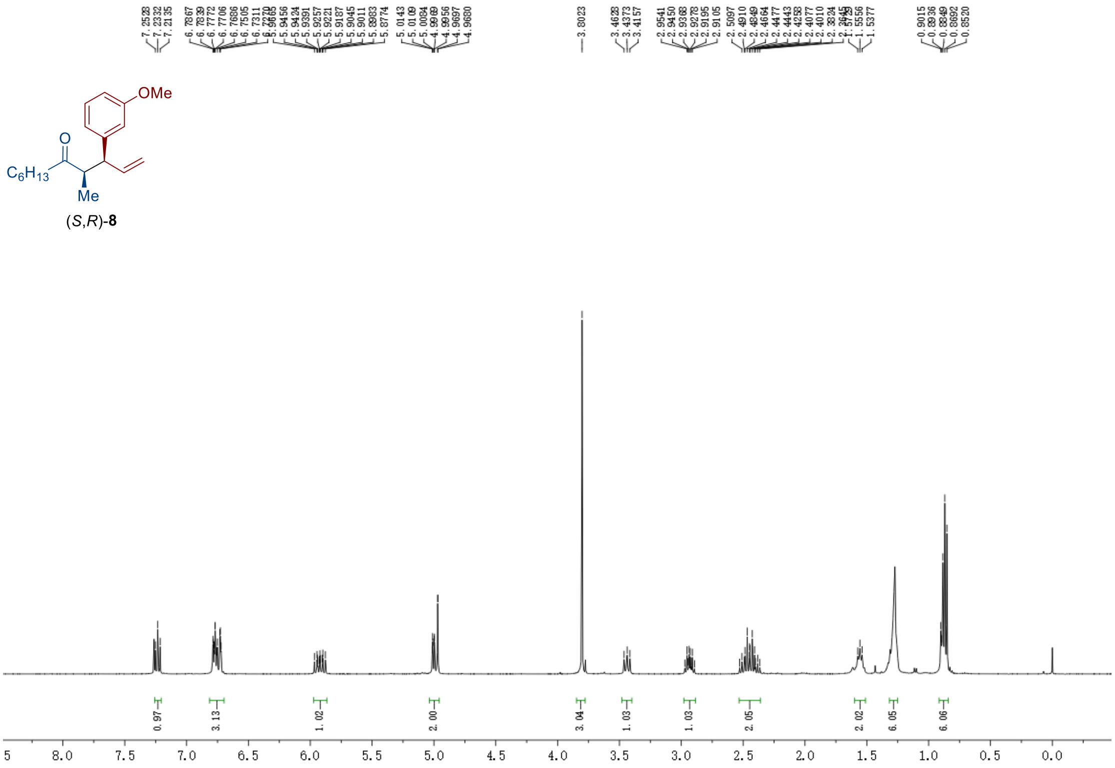

^{13}C NMR spectrum of **8**

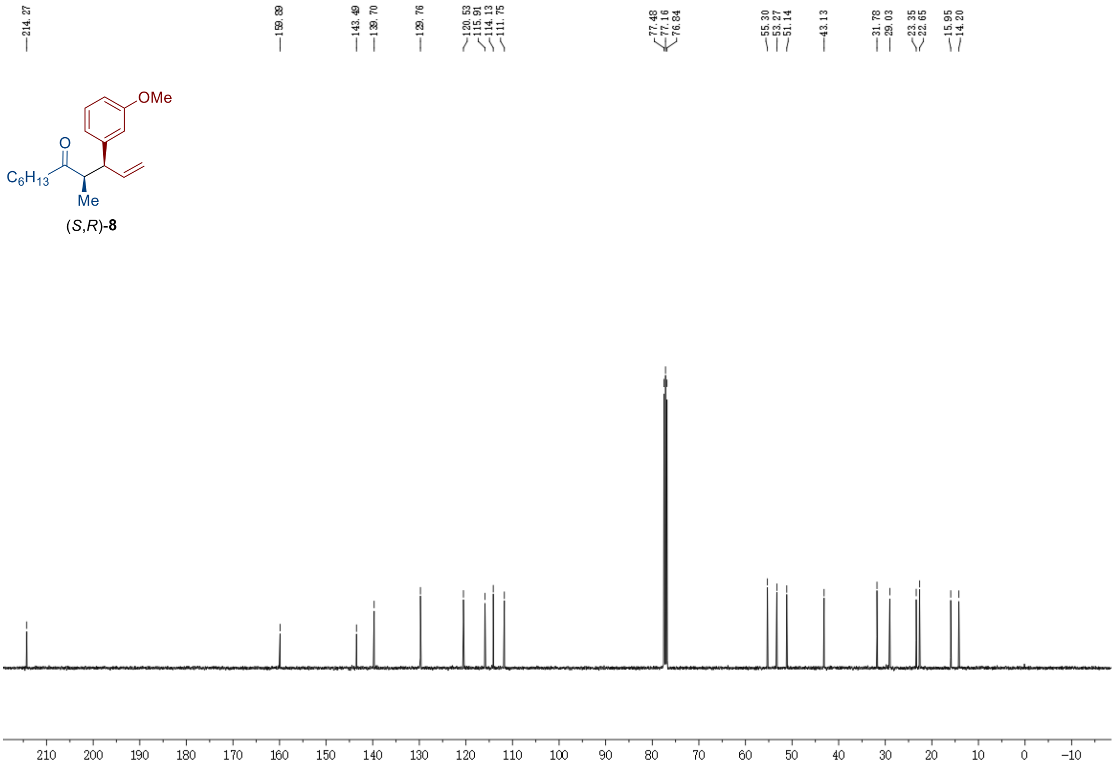

Supplementary Figure 63. NMR spectra of compound **8**

^{1}H NMR spectrum of **9**

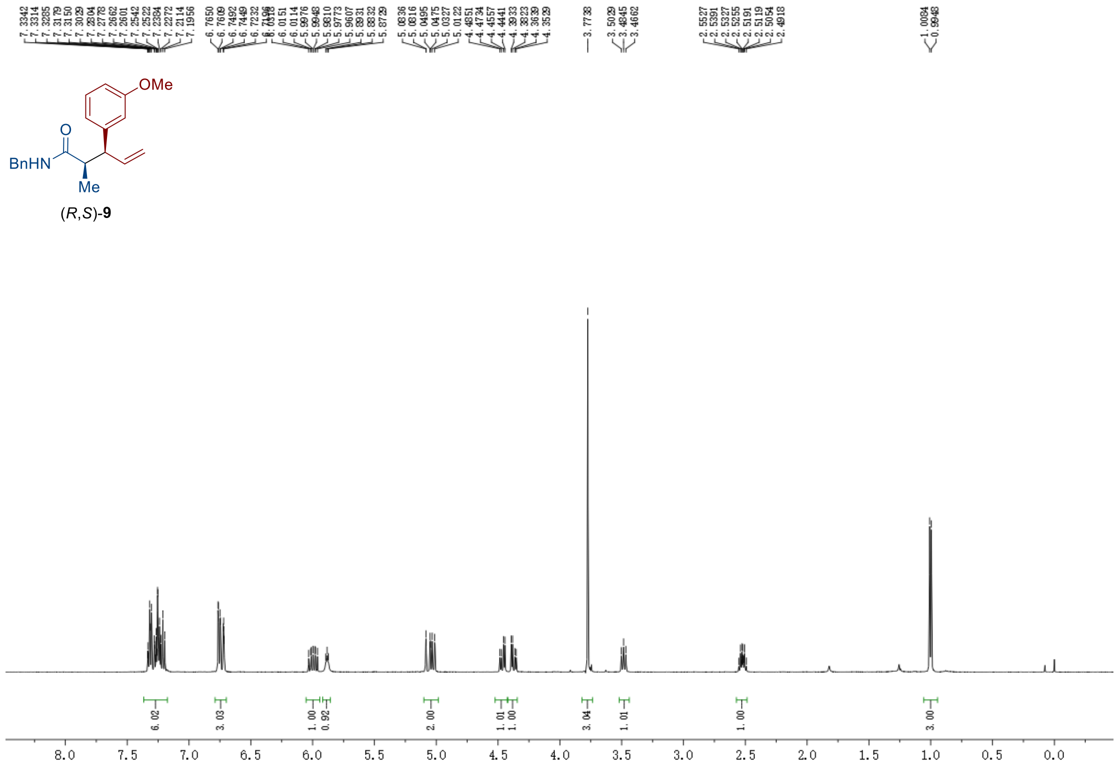

^{13}C NMR spectrum of **9**

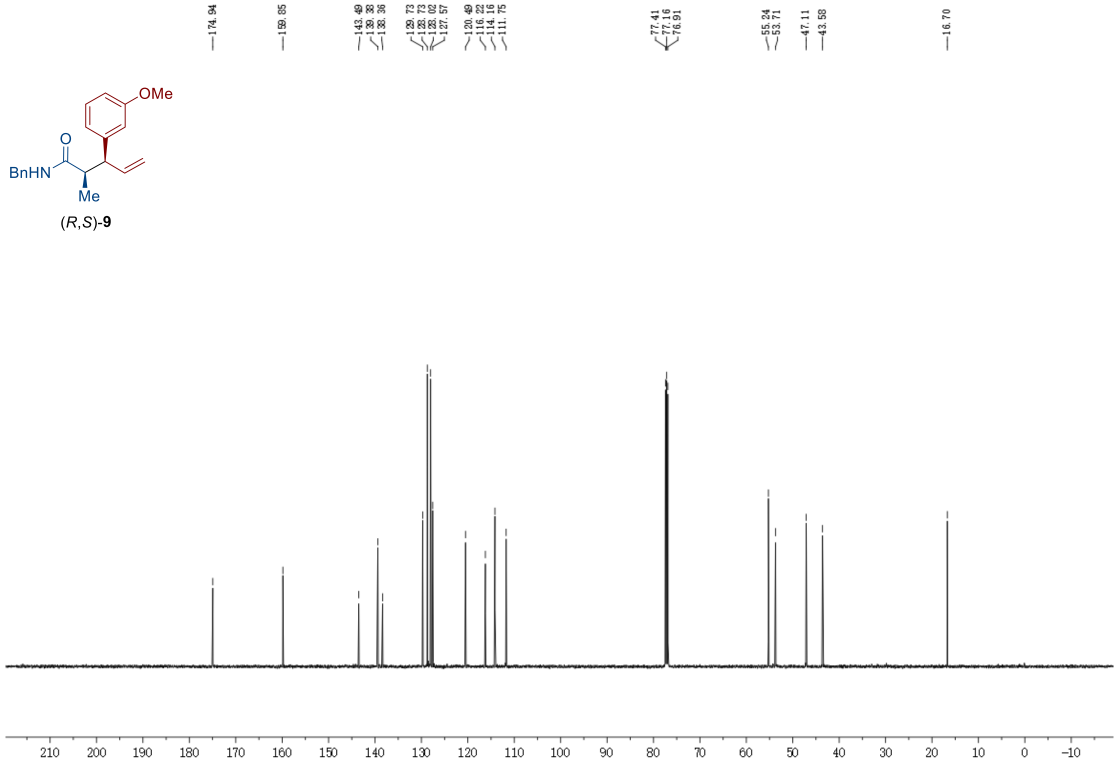

Supplementary Figure 64. NMR spectra of compound **9**

^{1}H NMR spectrum of **10**

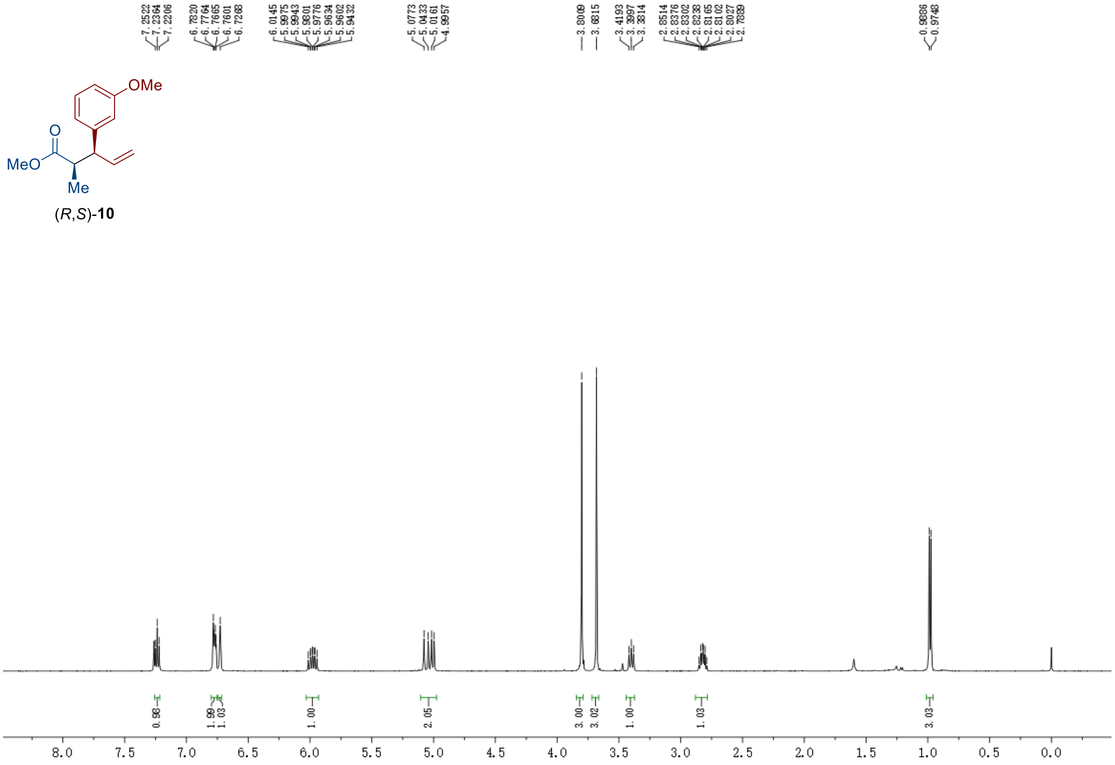

^{13}C NMR spectrum of **10**

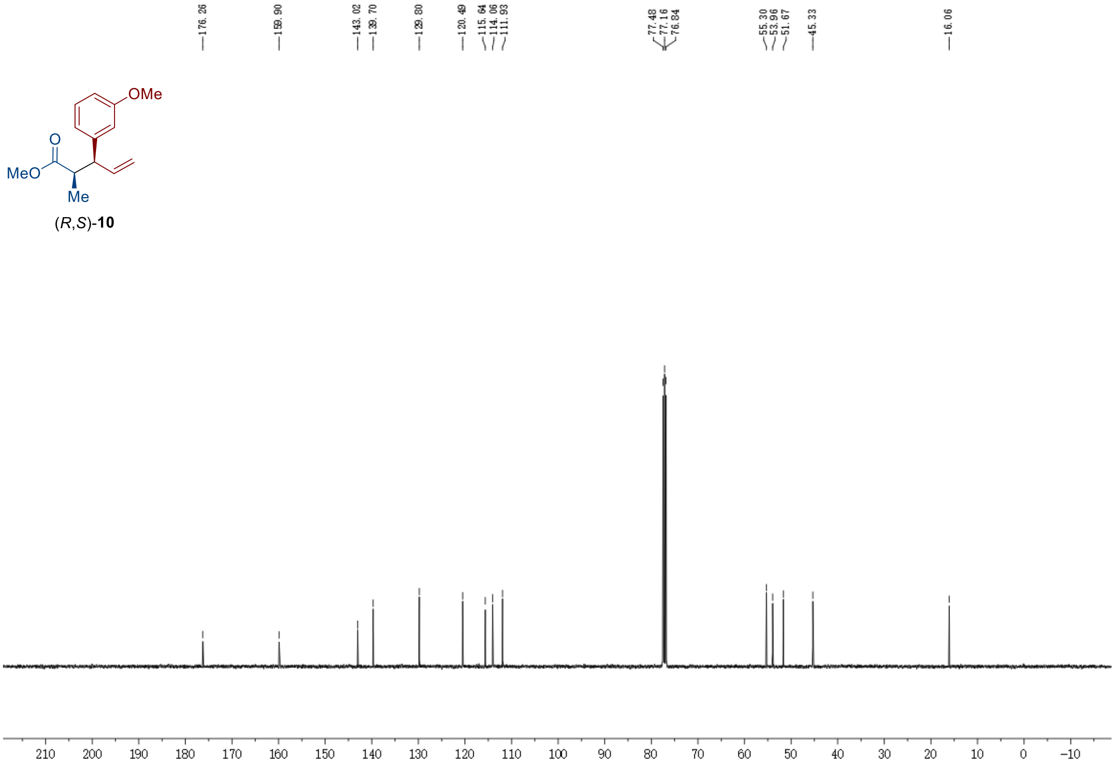

Supplementary Figure 65. NMR spectra of compound **10**

^{1}H NMR spectrum of **11**

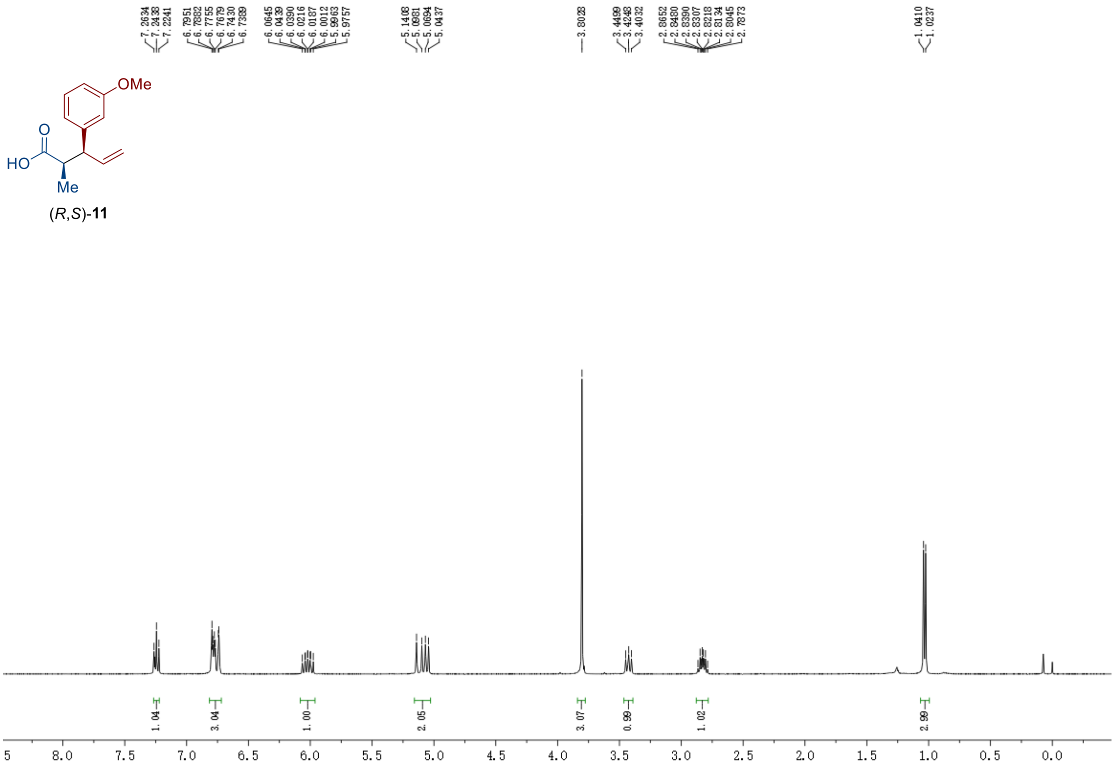

^{13}C NMR spectrum of **11**

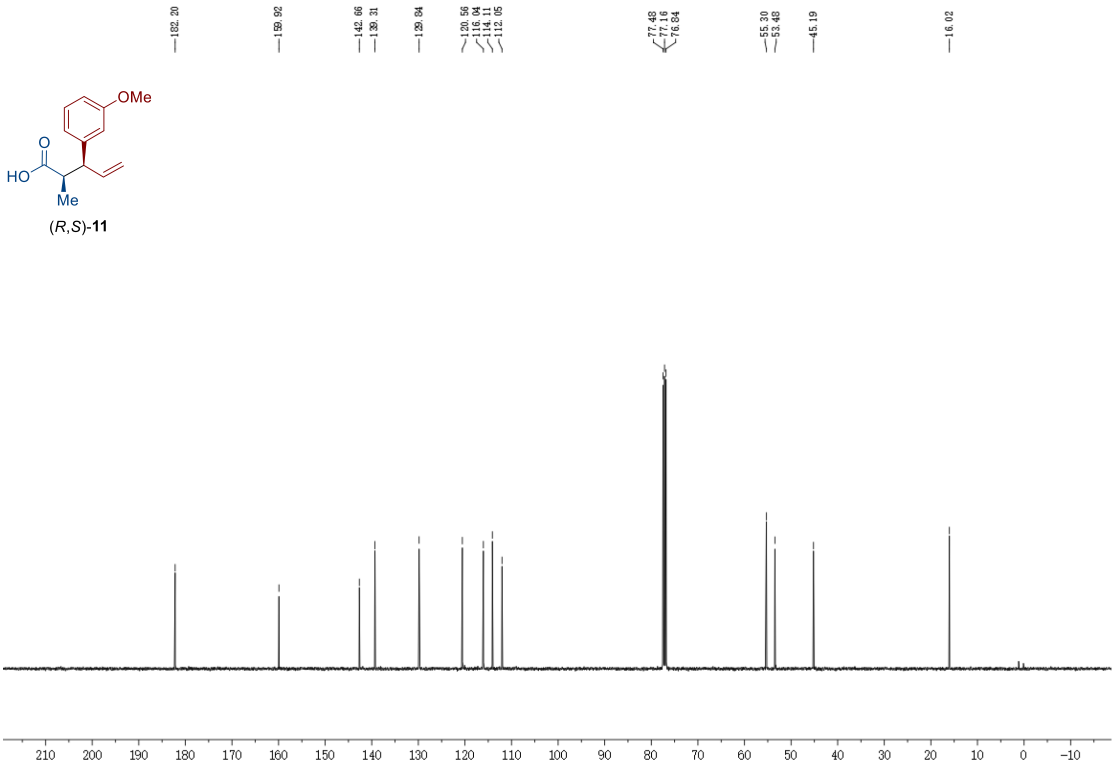

Supplementary Figure 66. NMR spectra of compound **11**

^{1}H NMR spectrum of **12**

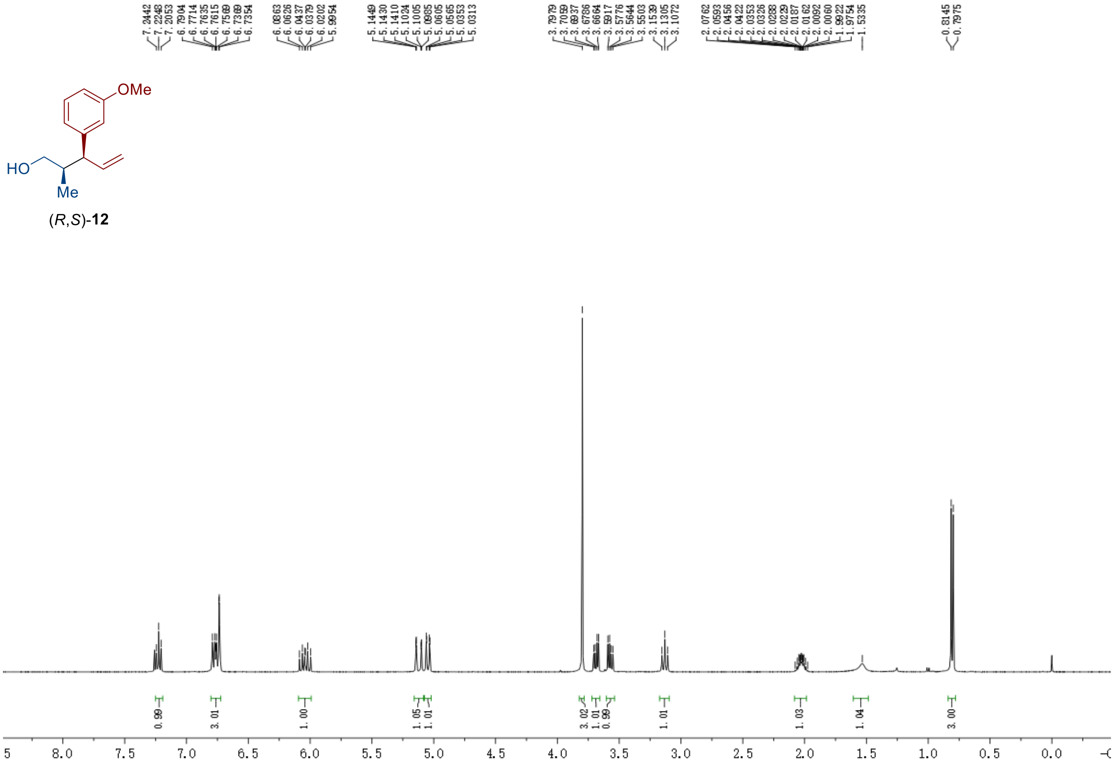

^{13}C NMR spectrum of **12**

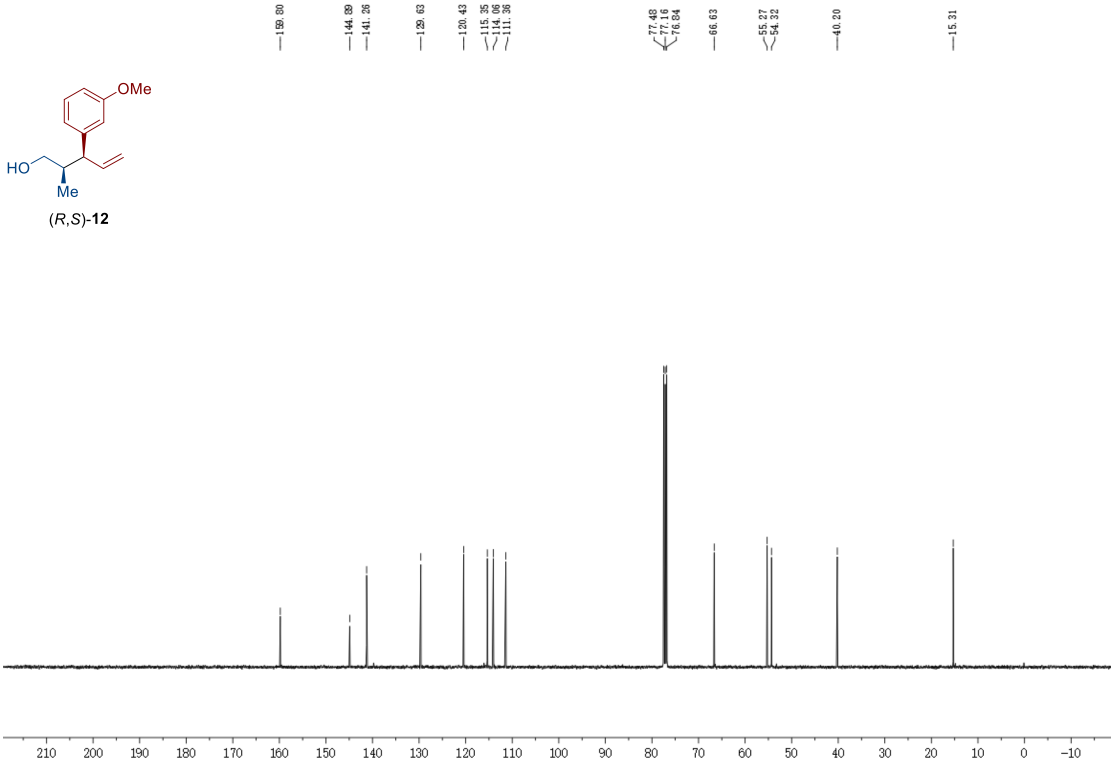

Supplementary Figure 67. NMR spectra of compound **12**

^{1}H NMR spectrum of **13**

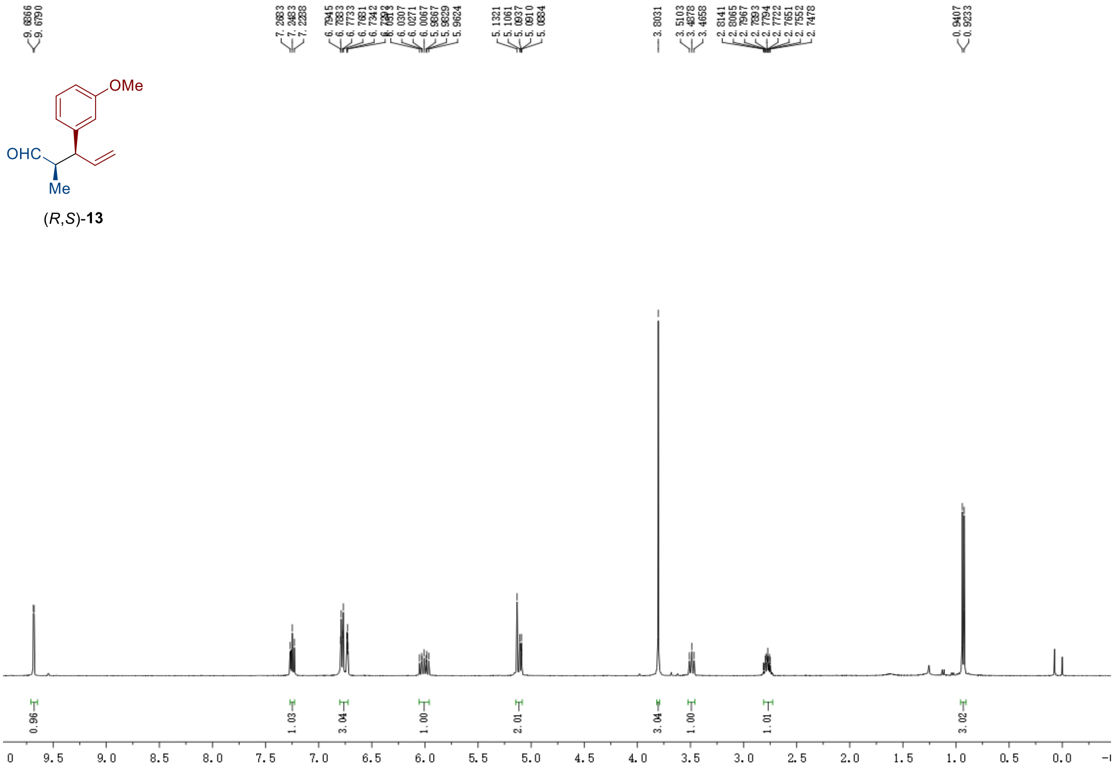

^{13}C NMR spectrum of **13**

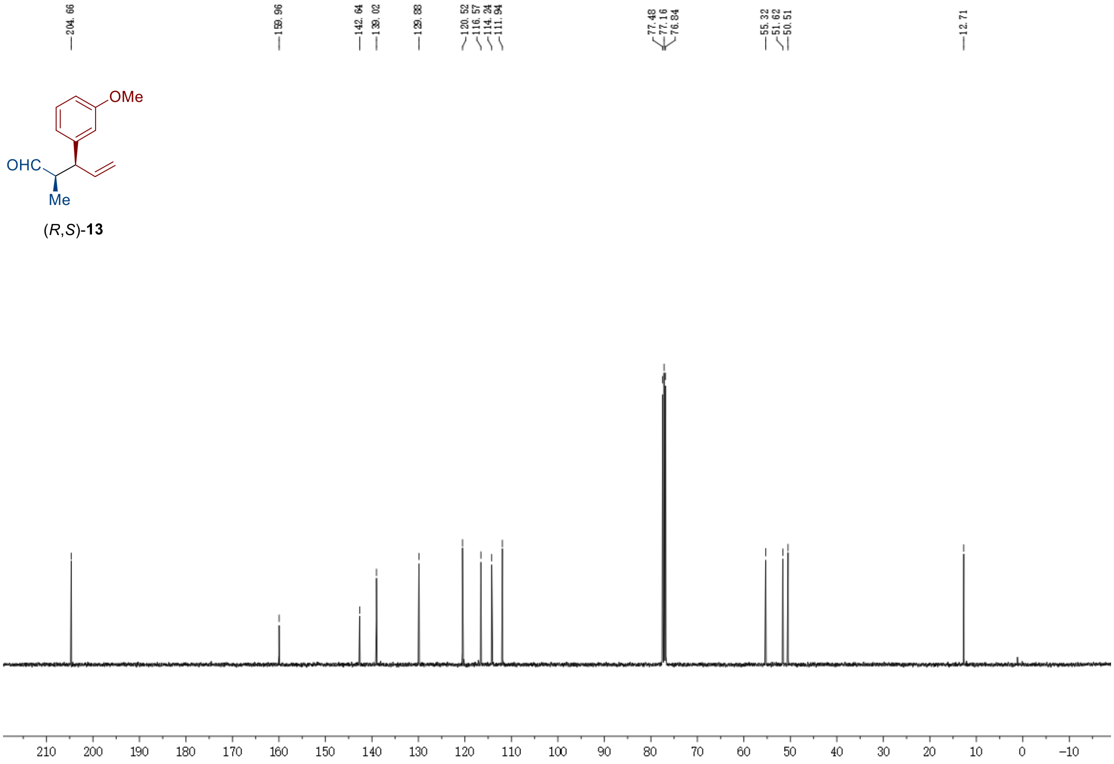

Supplementary Figure 68. NMR spectra of compound **13**

^{1}H NMR spectrum of **14**

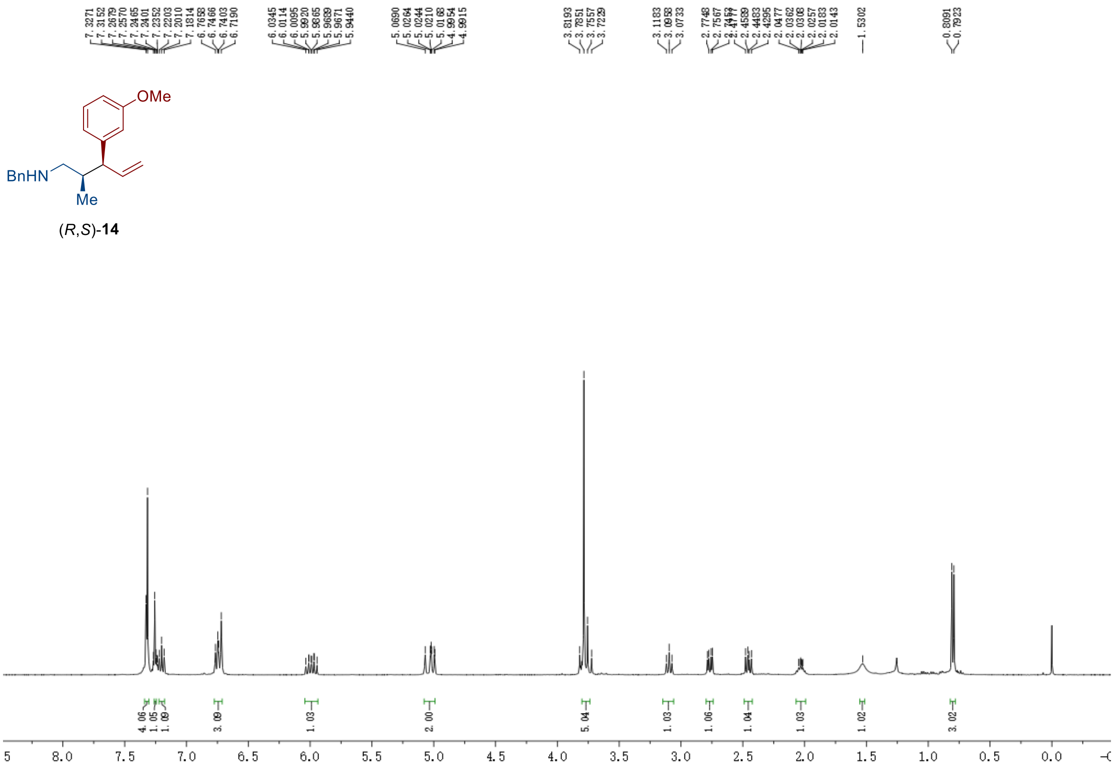

^{13}C NMR spectrum of **14**

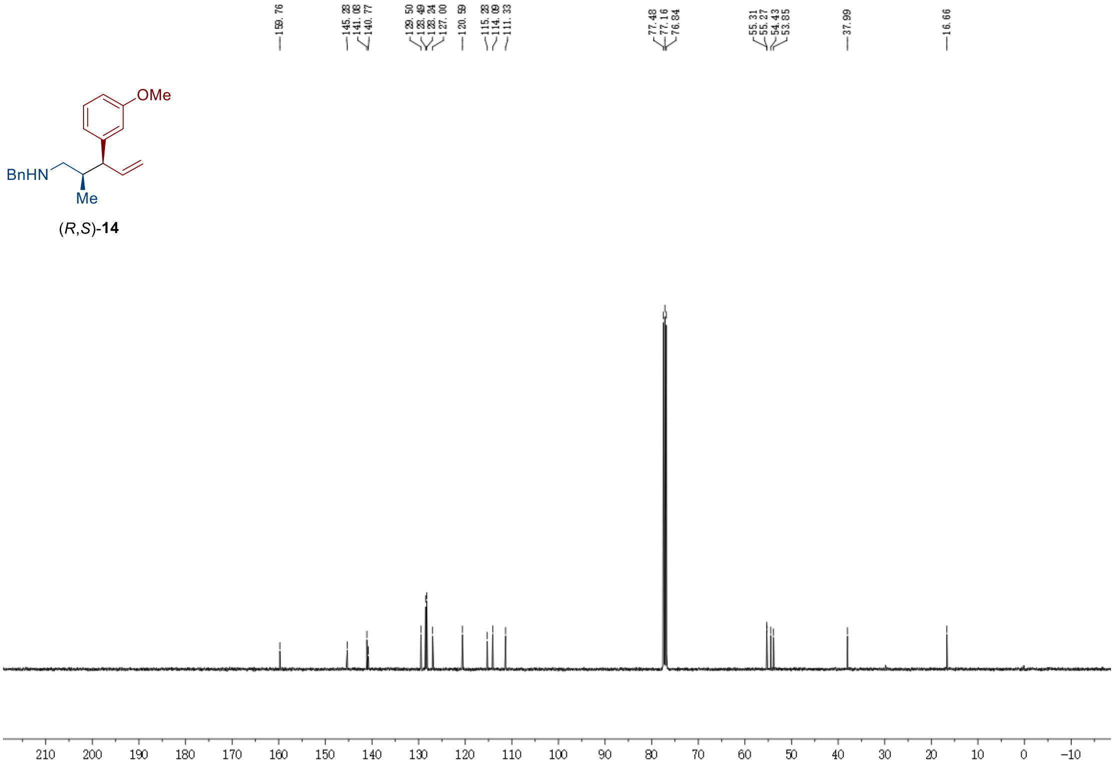

Supplementary Figure 69. NMR spectra of compound **14**

^{1}H NMR spectrum of **15**

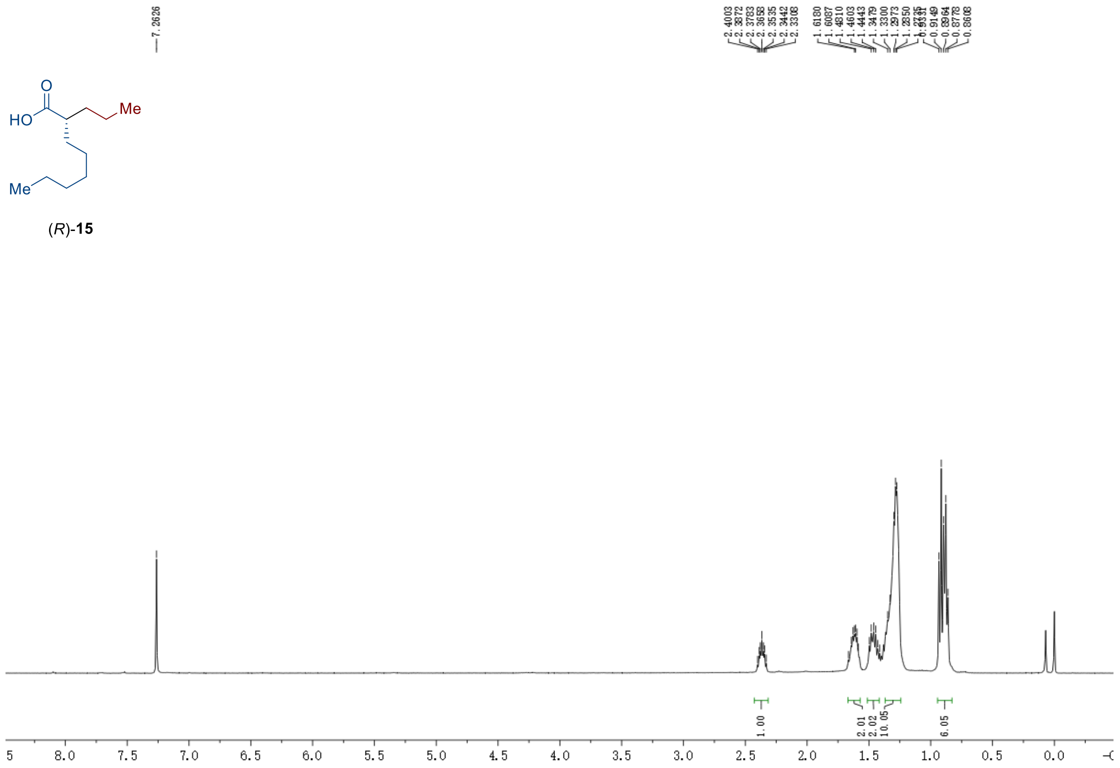

^{13}C NMR spectrum of **15**

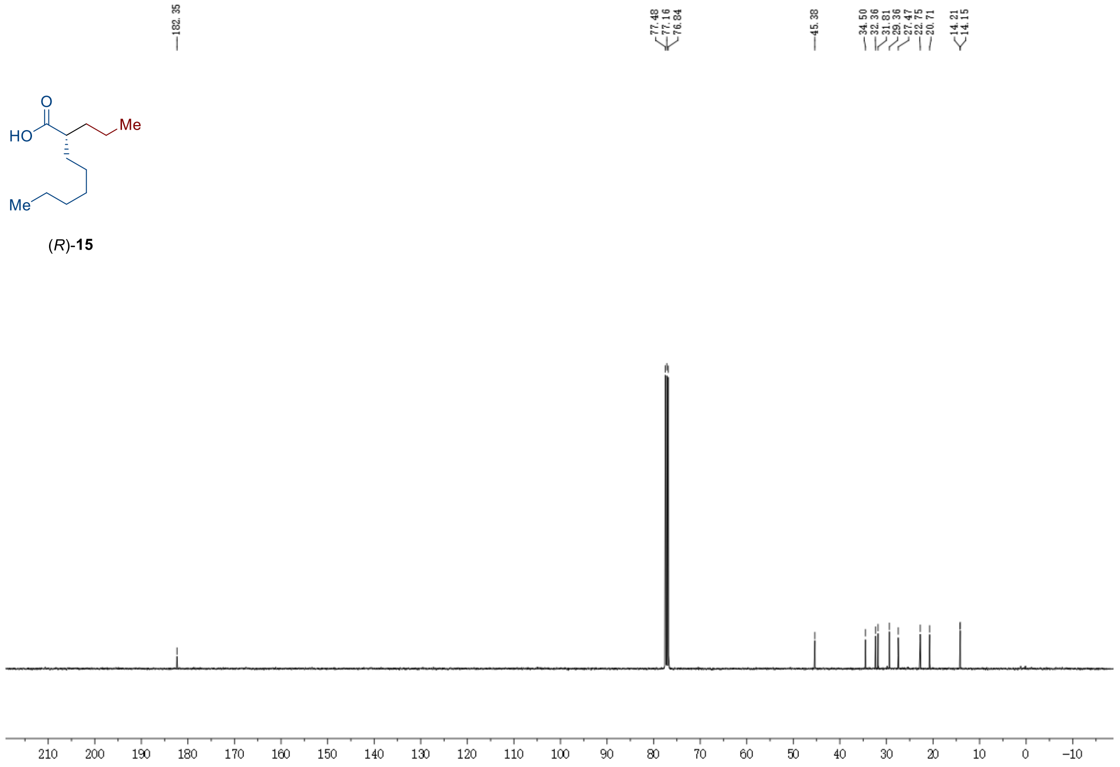

Supplementary Figure 70. NMR spectra of compound **15**

^{1}H NMR spectrum of **16**

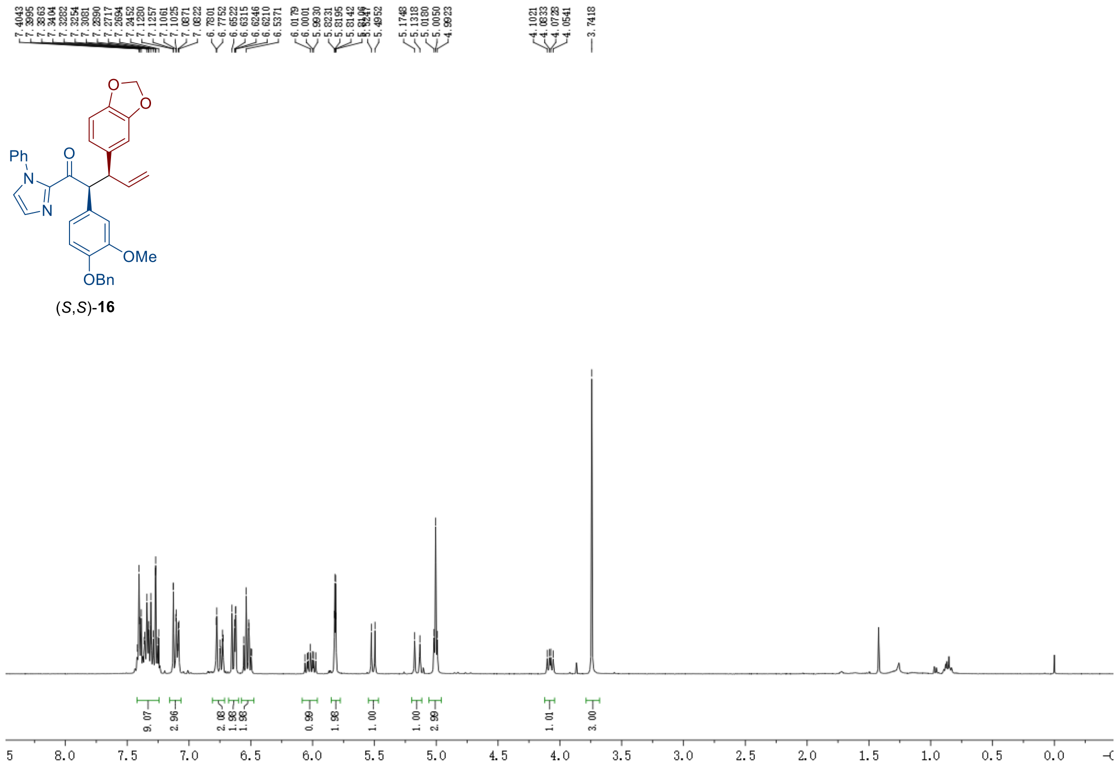

^{13}C NMR spectrum of **16**

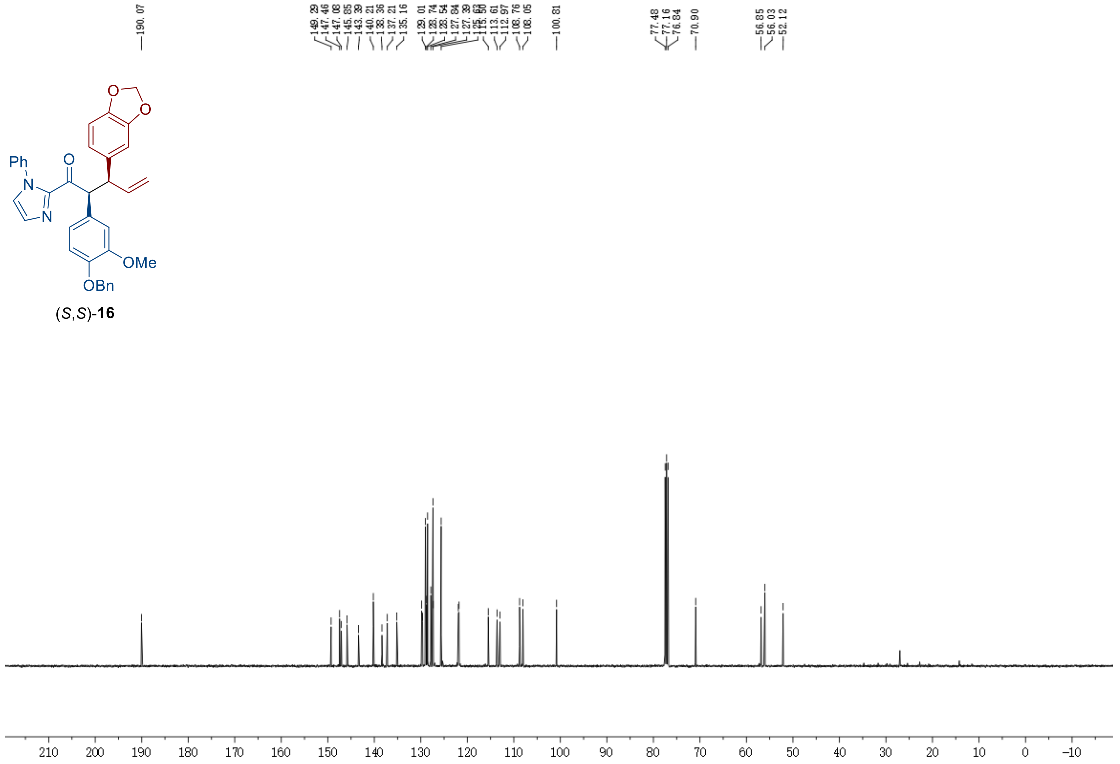

Supplementary Figure 71. NMR spectra of compound **16**

^{1}H NMR spectrum of **17**

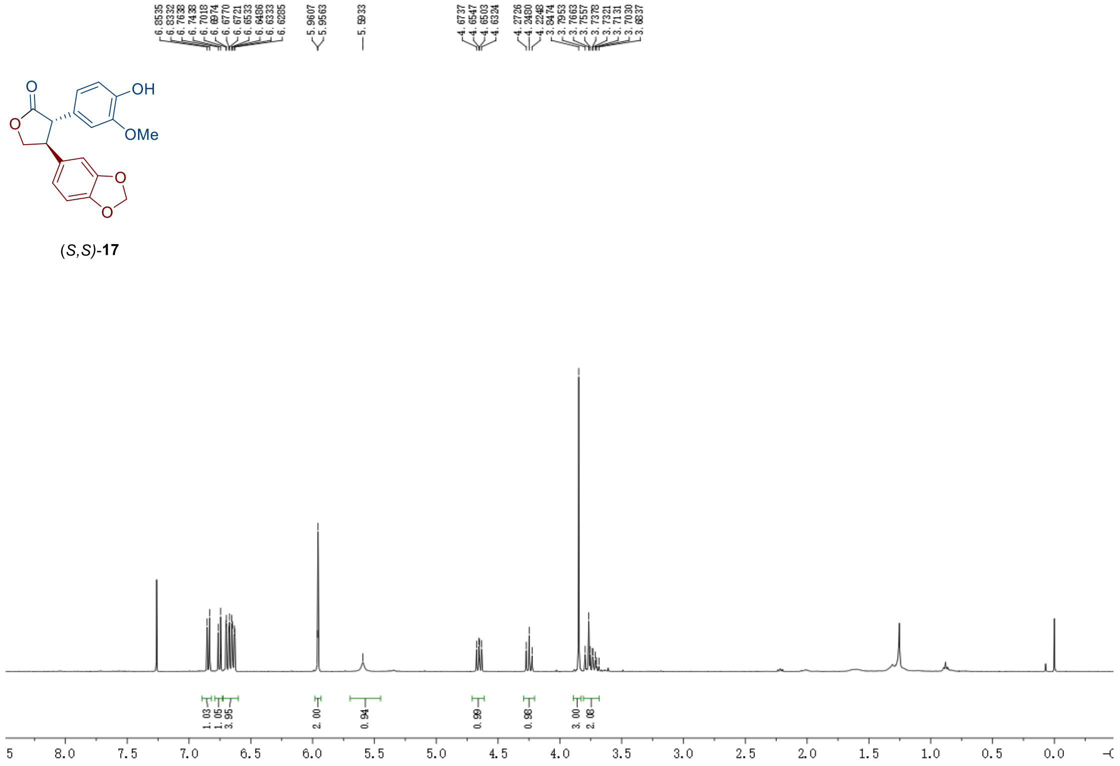

^{13}C NMR spectrum of **17**

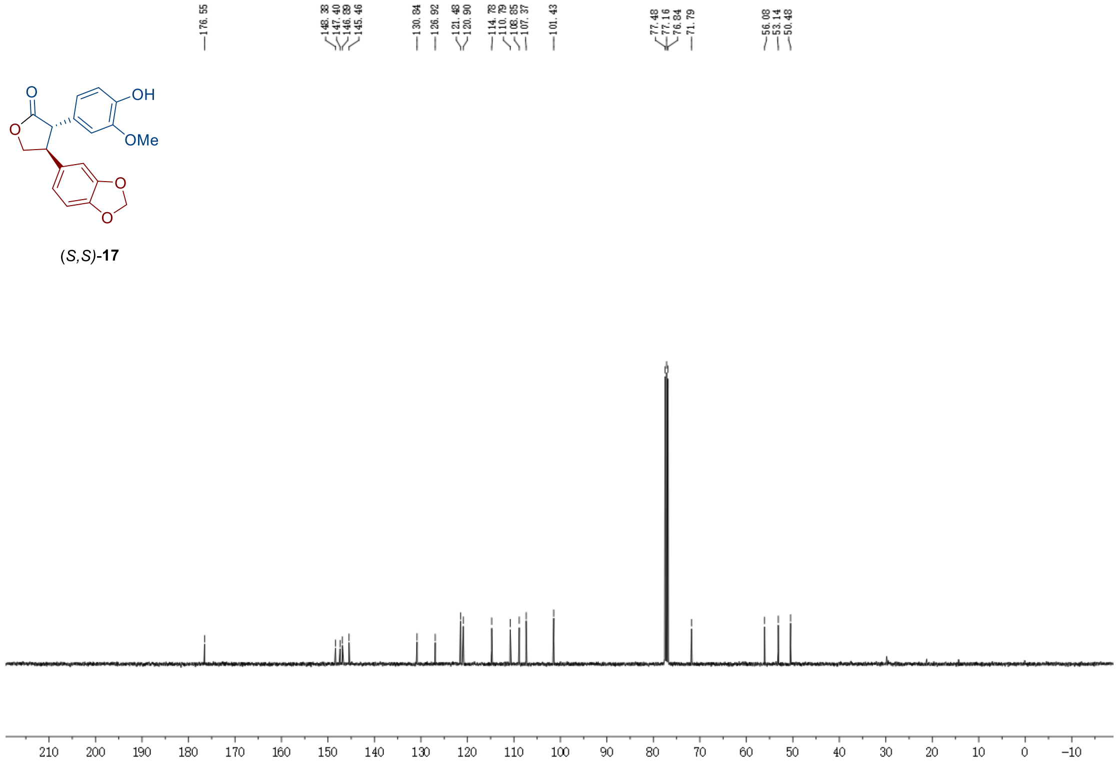

Supplementary Figure 72. NMR spectra of compound **17**

^{1}H NMR spectrum of (*R*,*S*)-**18**

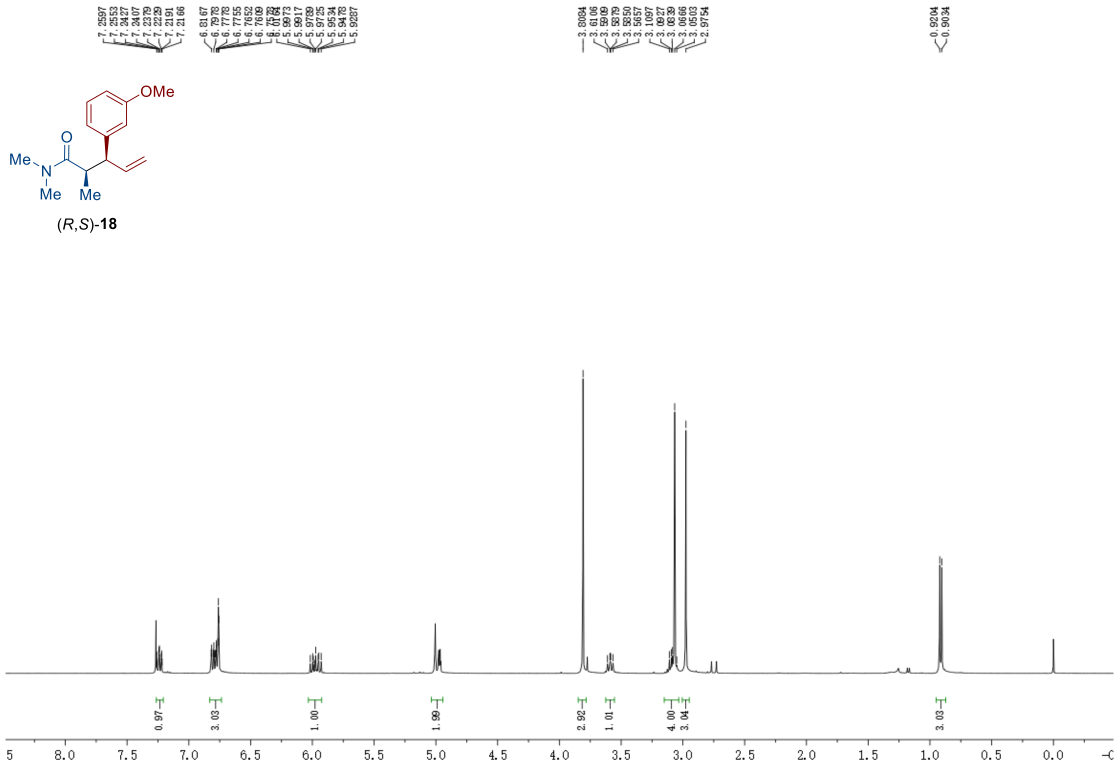

^{13}C NMR spectrum of (*R*,*S*)-**18**

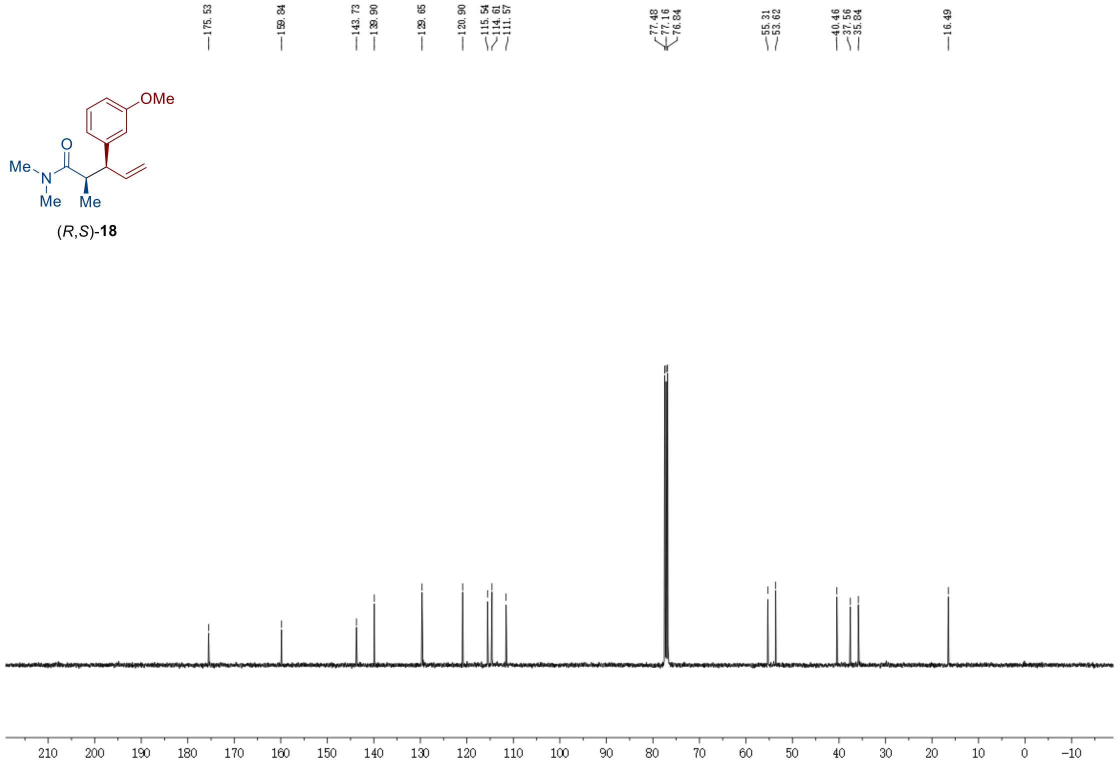

Supplementary Figure 73. NMR spectra of compound (*R*,*S*)-**18**

^{1}H NMR spectrum of (*R*,*R*)-**18**

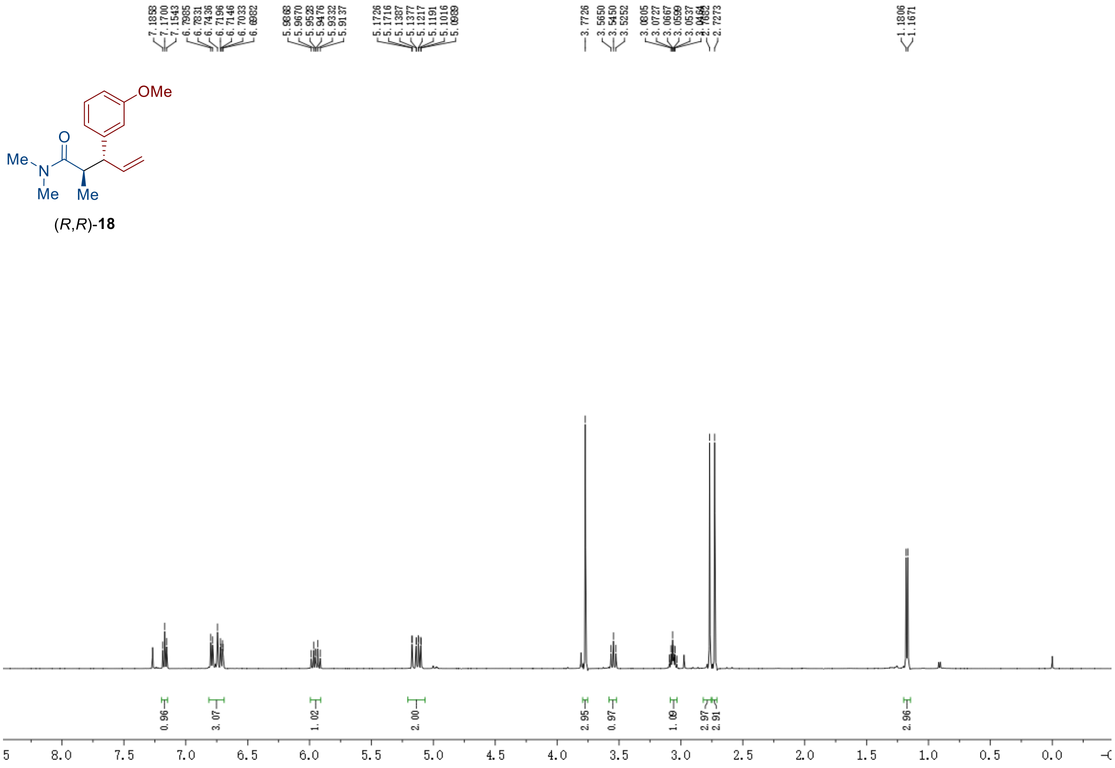

^{13}C NMR spectrum of (*R*,*R*)-**18**

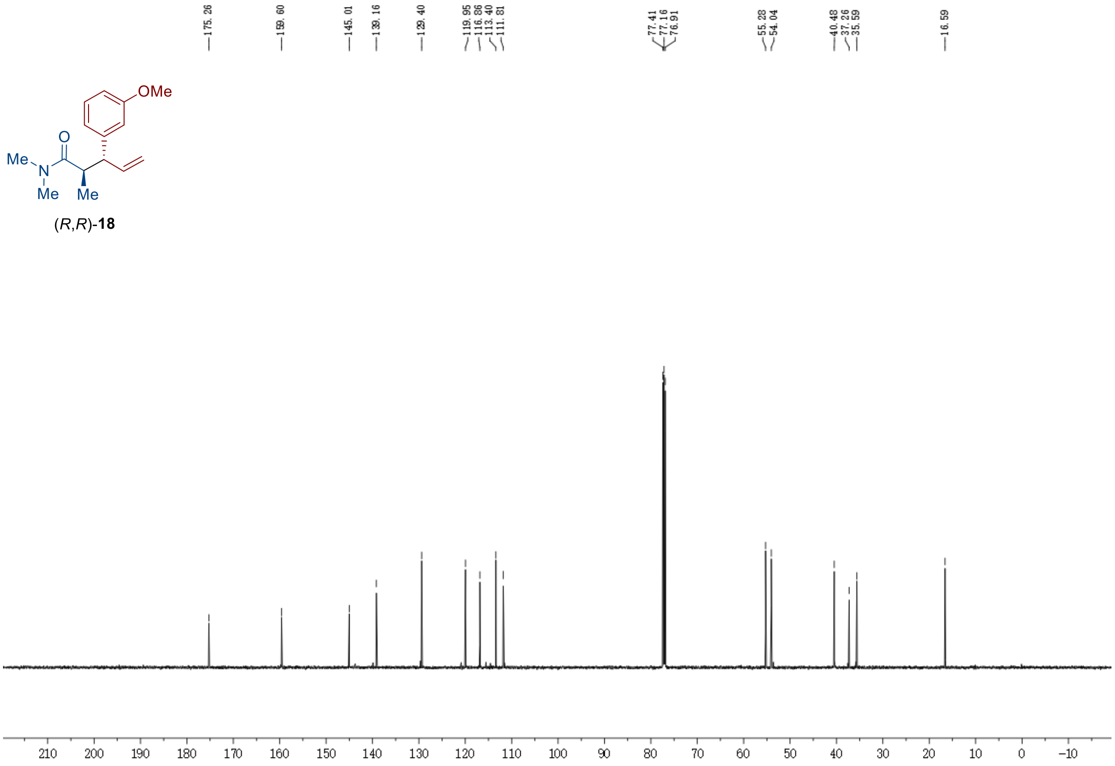

Supplementary Figure 74. NMR spectra of compound (*R*,*R*)-**18**

^{1}H NMR spectrum of (*S*,*R*)-**18**

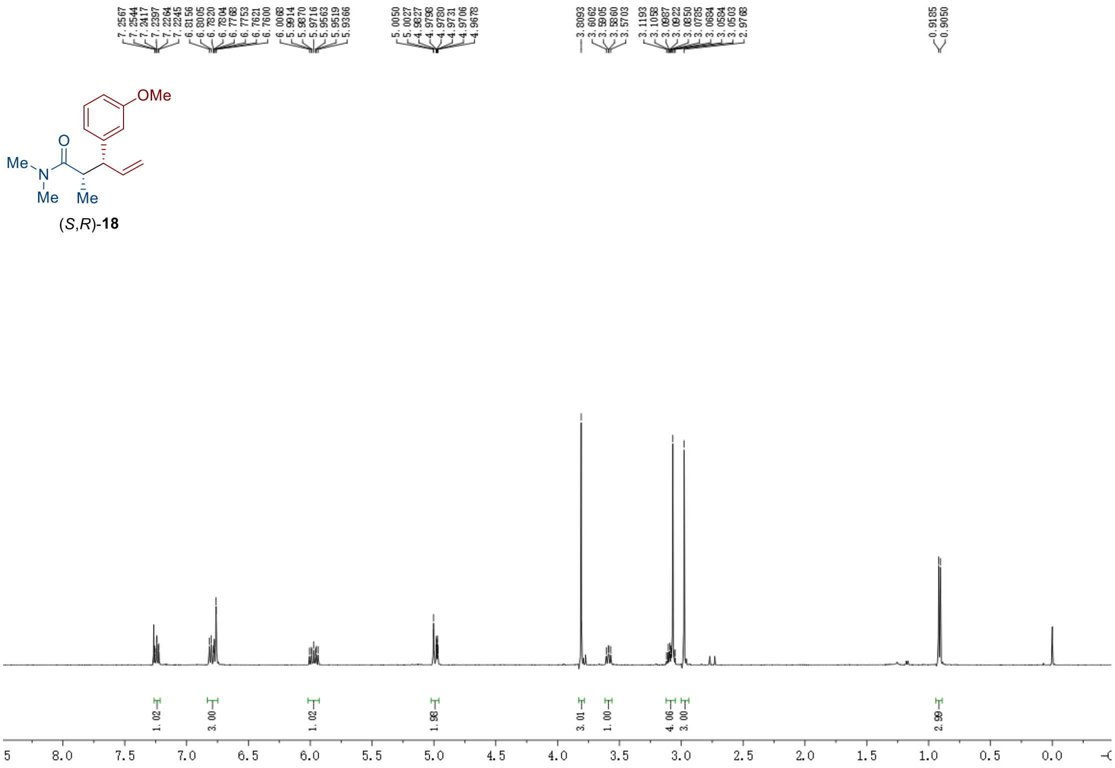

^{13}C NMR spectrum of (*S*,*R*)-**18**

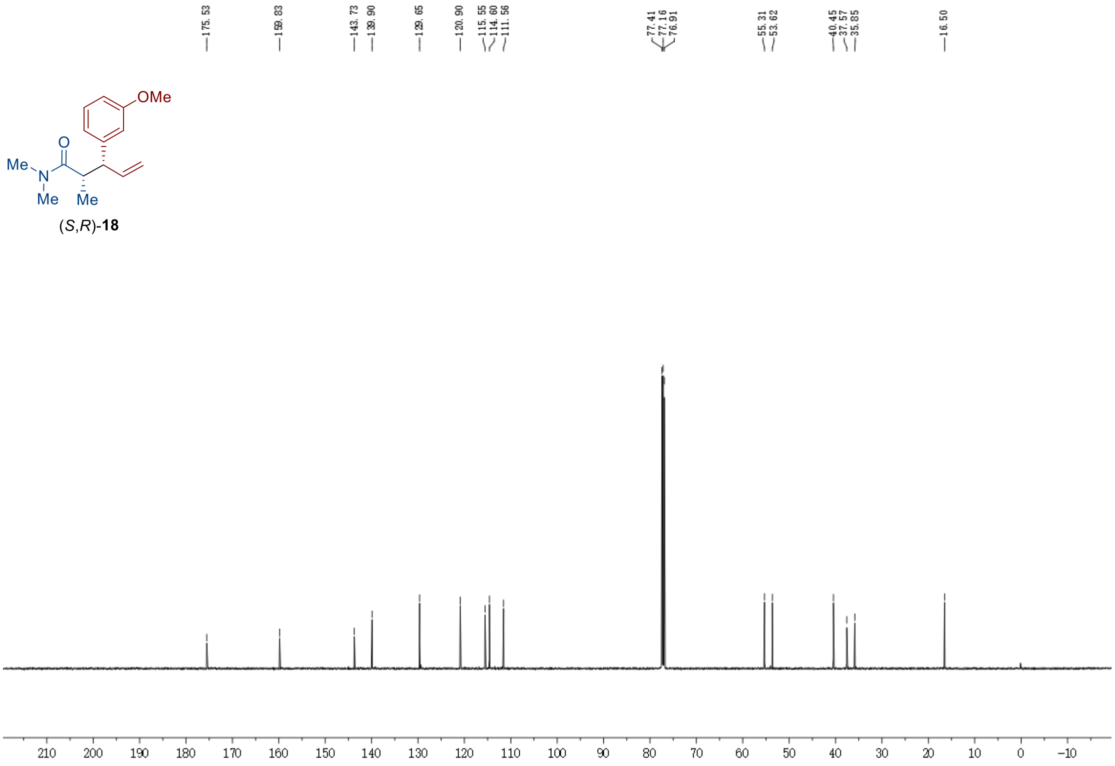

Supplementary Figure 75. NMR spectra of compound (*S*,*R*)-**18**

^{1}H NMR spectrum of (*S*,*S*)-**18**

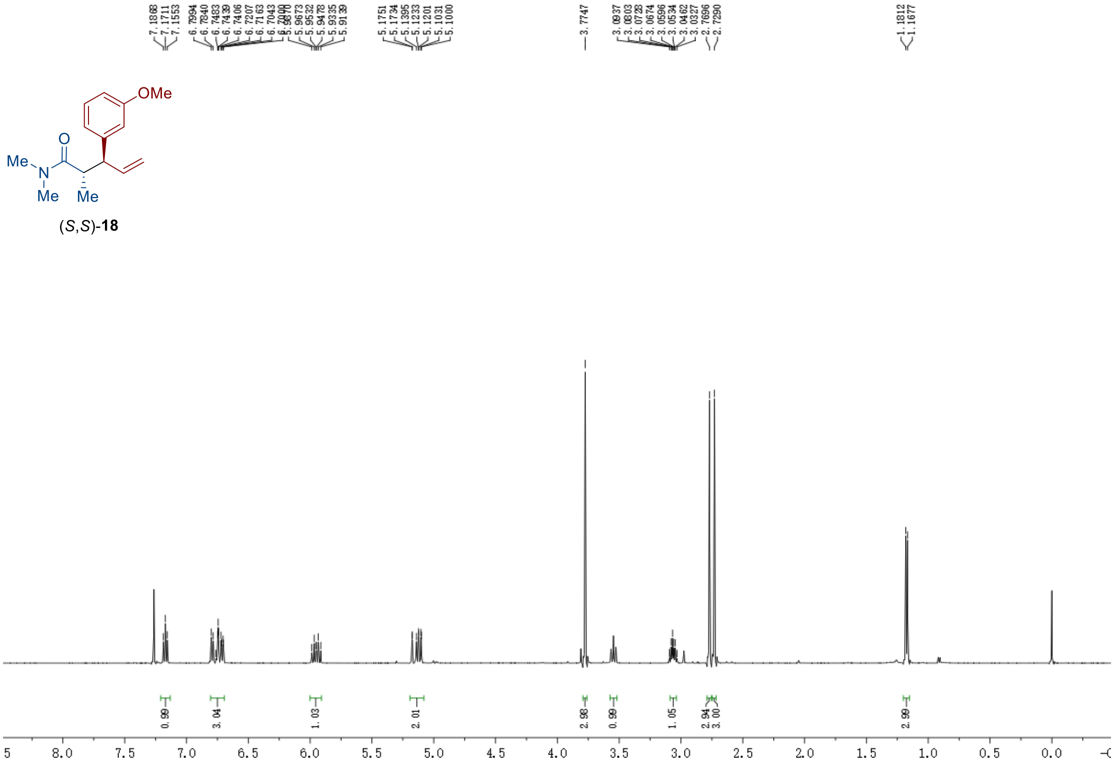

^{13}C NMR spectrum of (*S*,*S*)-**18**

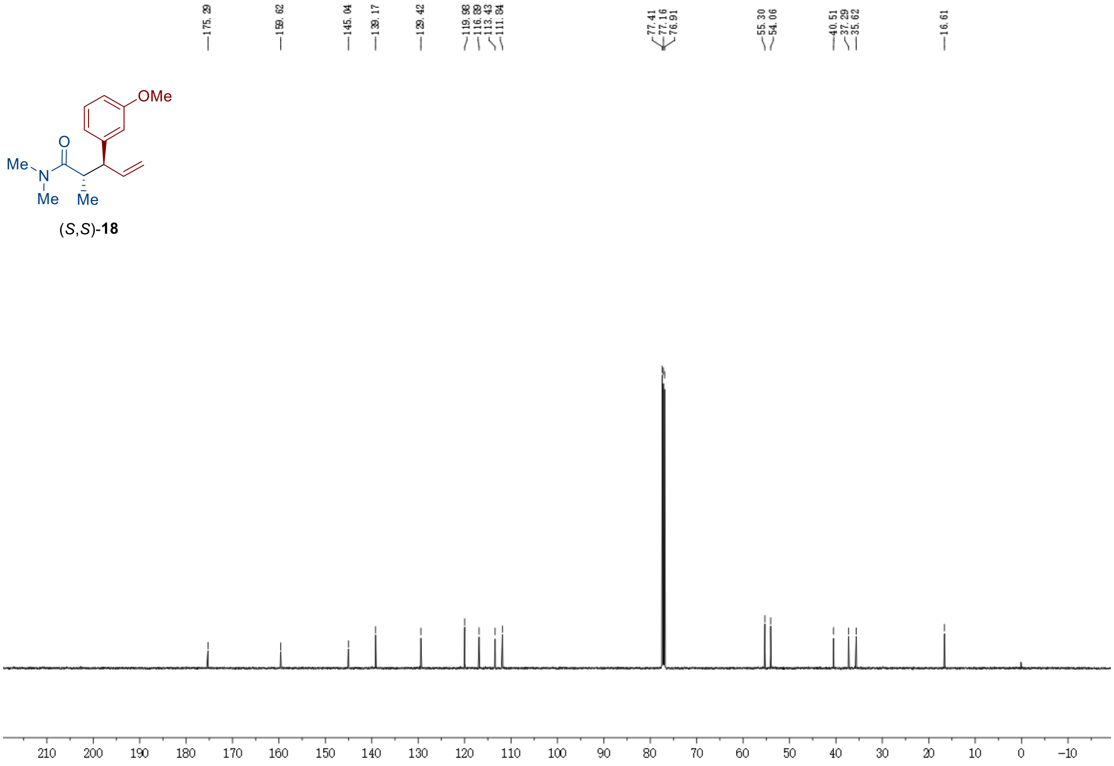

Supplementary Figure 76. NMR spectra of compound (*S*,*S*)-**18**

^{1}H NMR spectrum of (*R*,*S*)-**19**

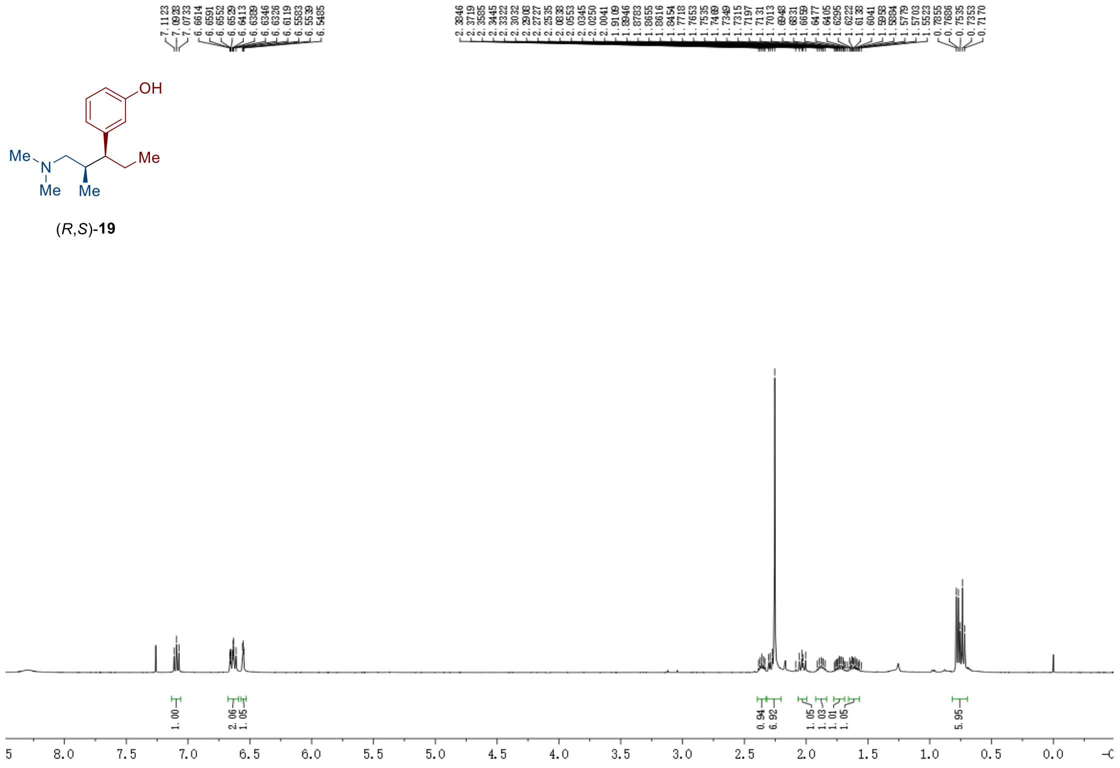

^{13}C NMR spectrum of (*R*,*S*)-**19**

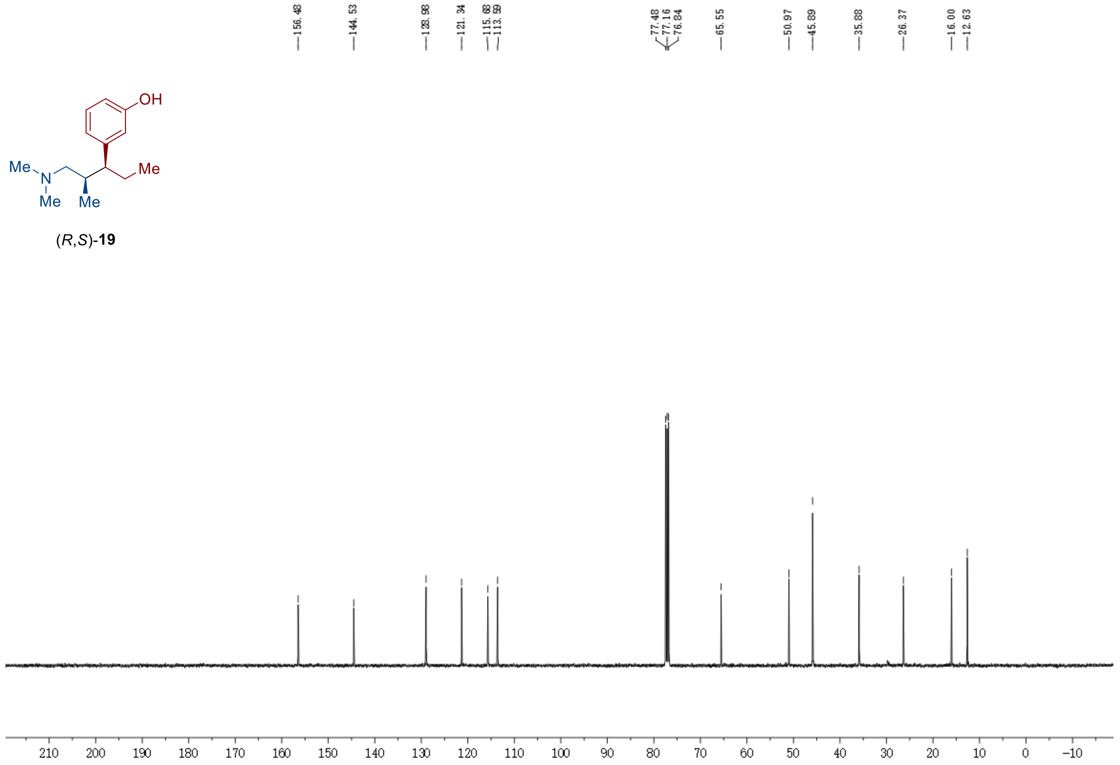

Supplementary Figure 77. NMR spectra of compound (*R*,*S*)-**19**

^{1}H NMR spectrum of (*R*,*R*)-**19**

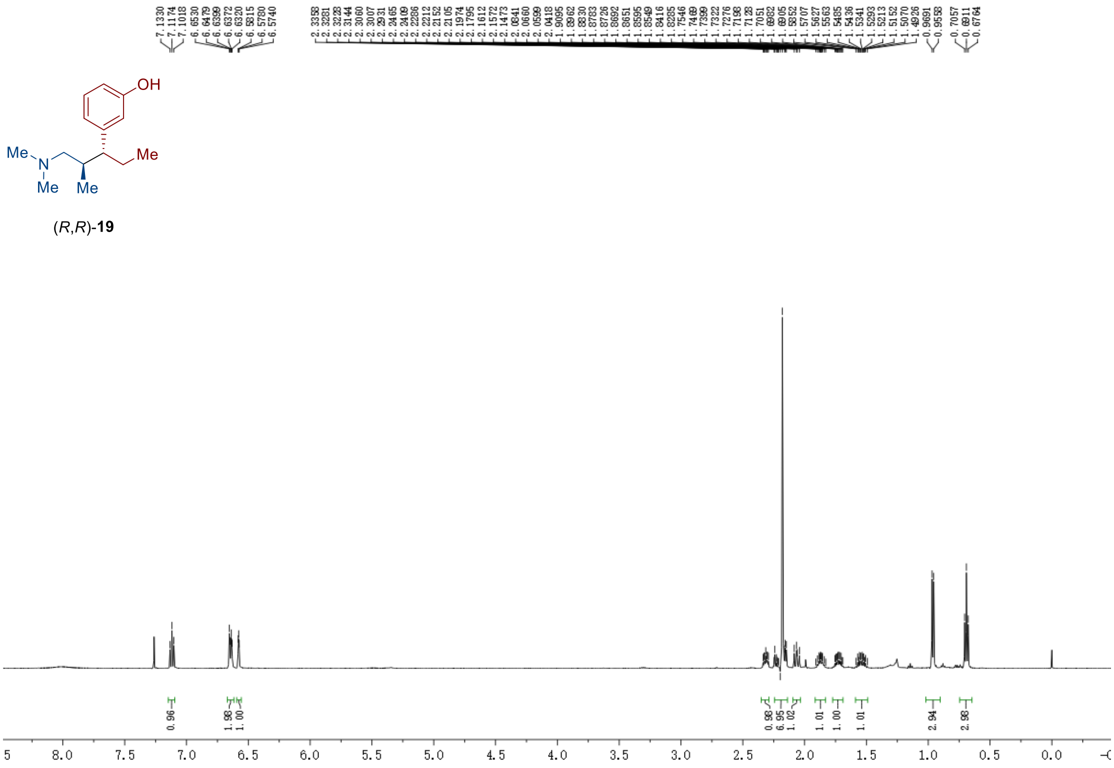

^{13}C NMR spectrum of (*R*,*R*)-**19**

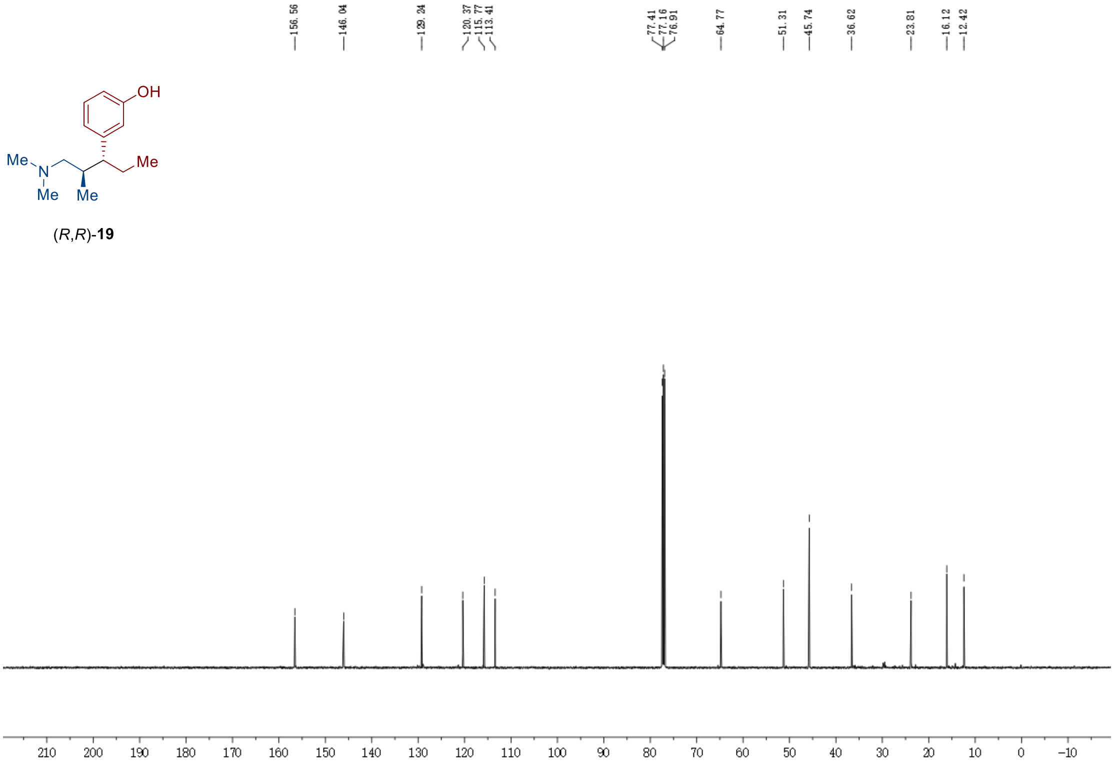

Supplementary Figure 78. NMR spectra of compound (*R*,*R*)-**19**

^{1}H NMR spectrum of (*S*,*R*)-**19**

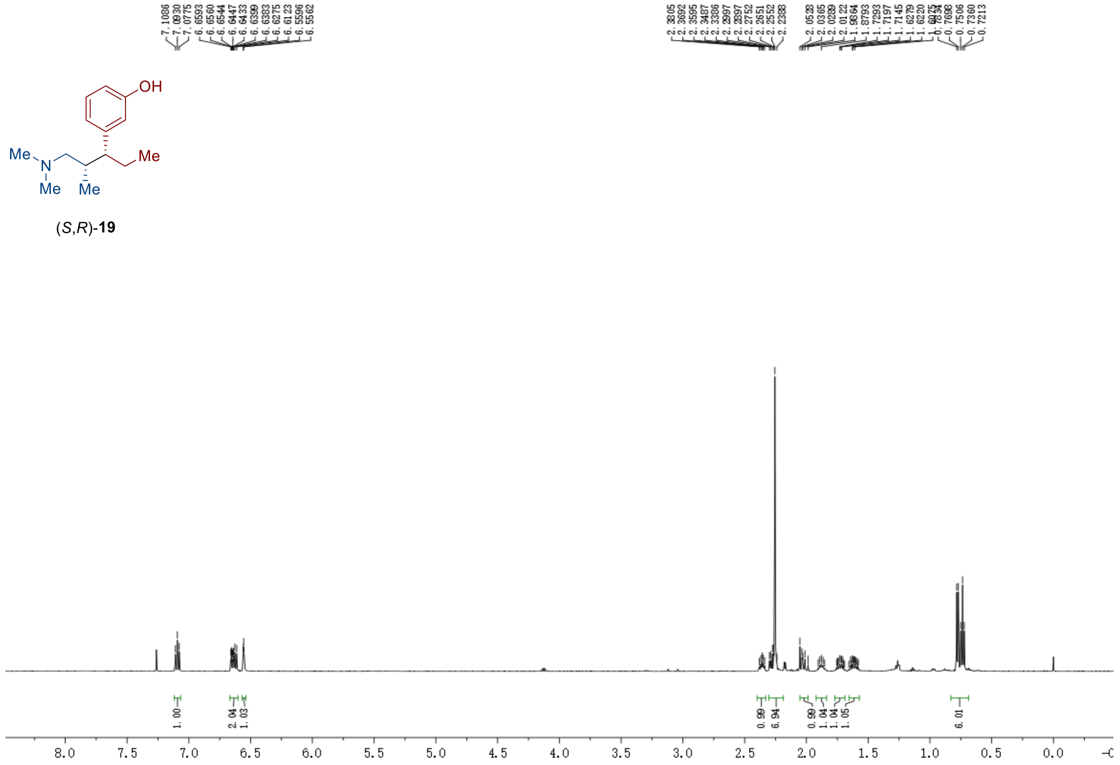

^{13}C NMR spectrum of (*S*,*R*)-**19**

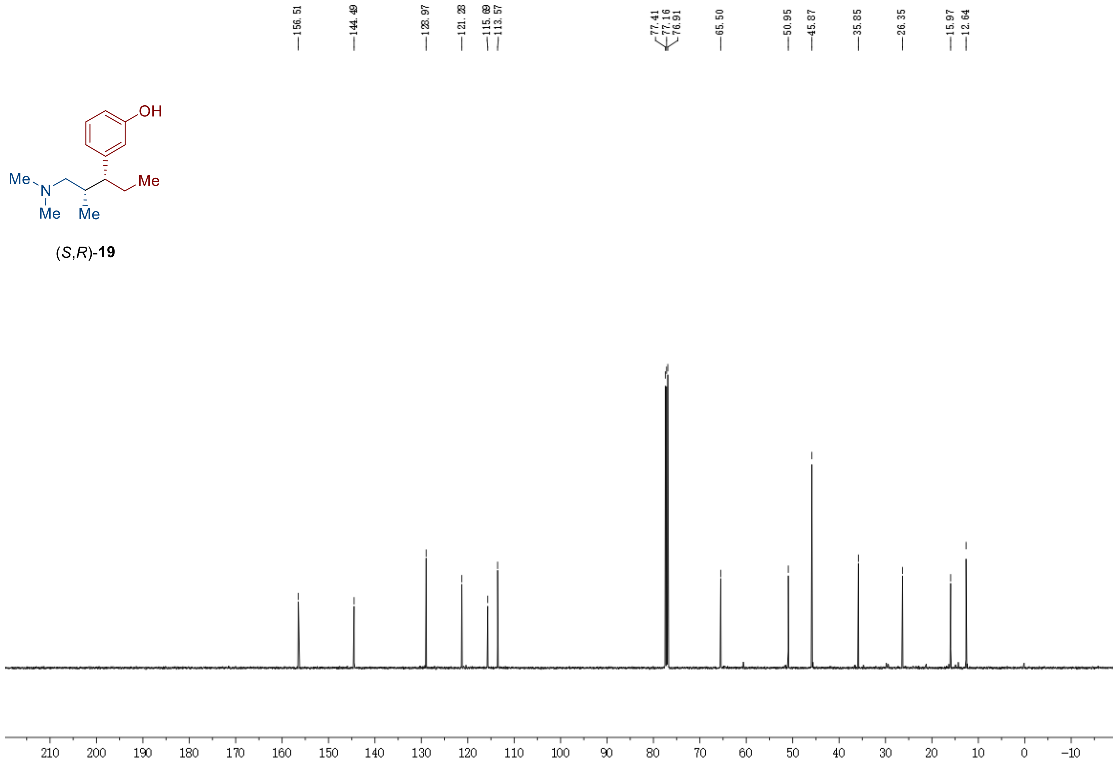

Supplementary Figure 79. NMR spectra of compound (*S*,*R*)-**19**

^{1}H NMR spectrum of (*S*,*S*)-**19**

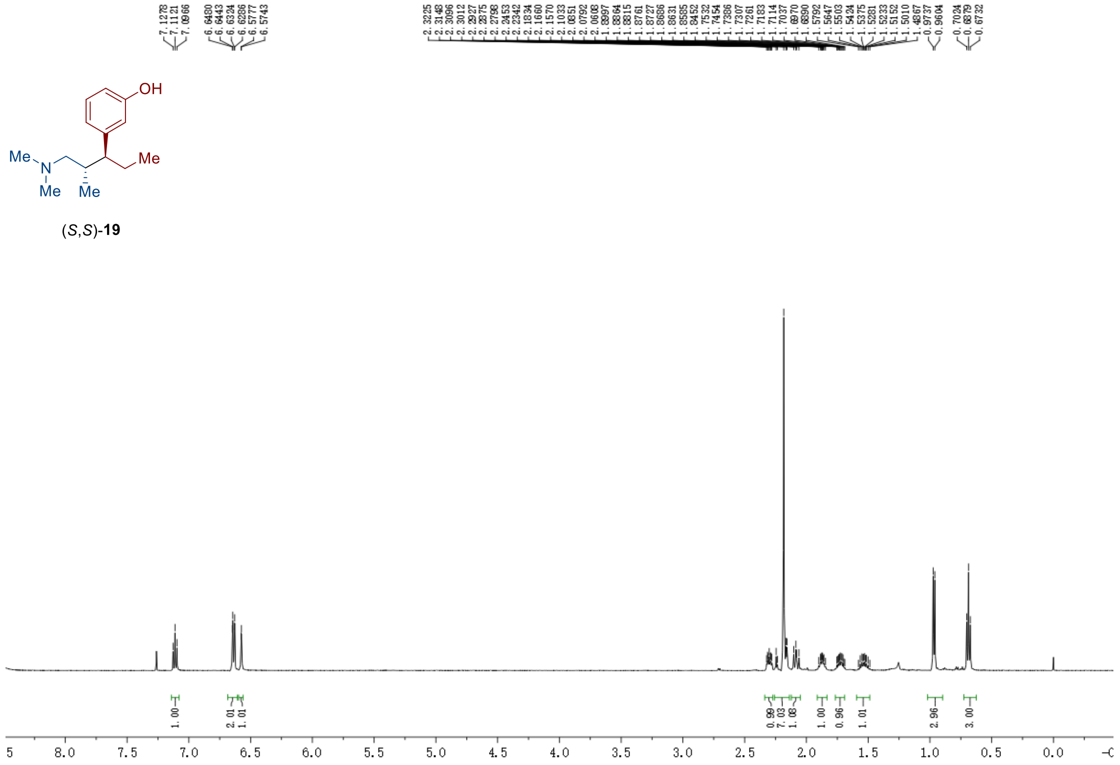

^{13}C NMR spectrum of (*S*,*S*)-**19**

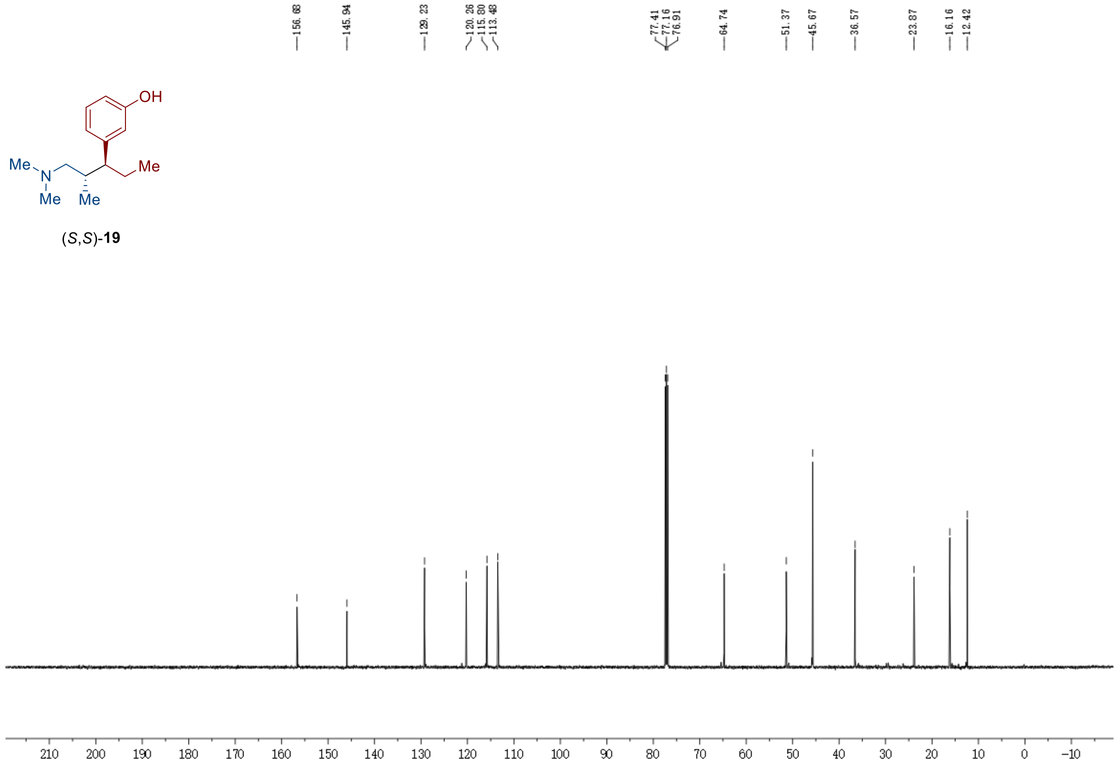

Supplementary Figure 80. NMR spectra of compound (*S*,*S*)-**19**

7. HPLC traces

rac-**3a**

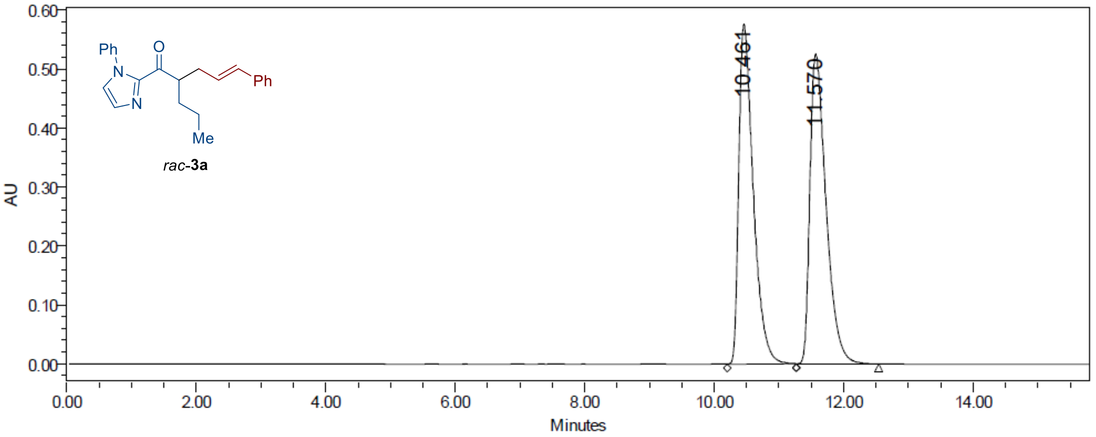

	RT	Area	% Area	Height
1	10.461	8907639	49.67	575861
2	11.570	9025751	50.33	525784

(*R*)-**3a**

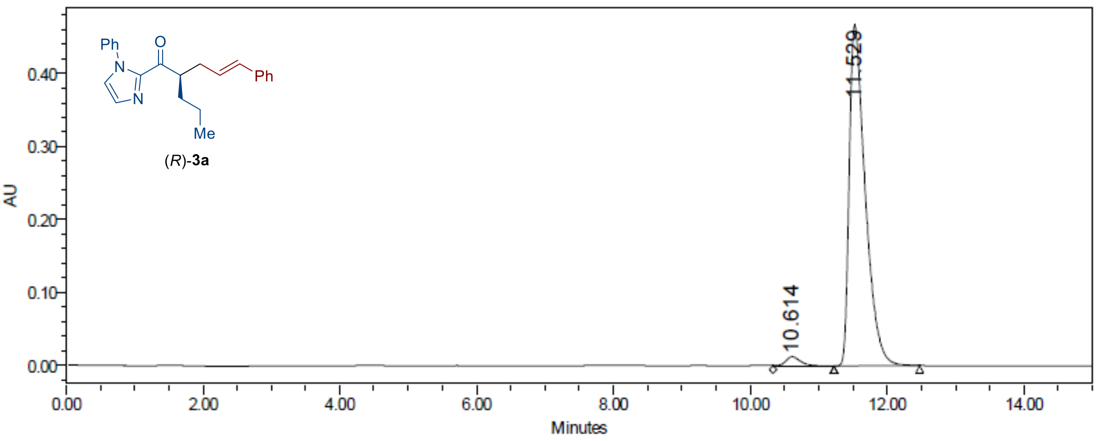

	RT	Area	% Area	Height
1	10.614	189391	2.42	12625
2	11.529	7636007	97.58	467340

Supplementary Figure 81. HPLC spectra of compound **3a**

(*S*)-**3a**

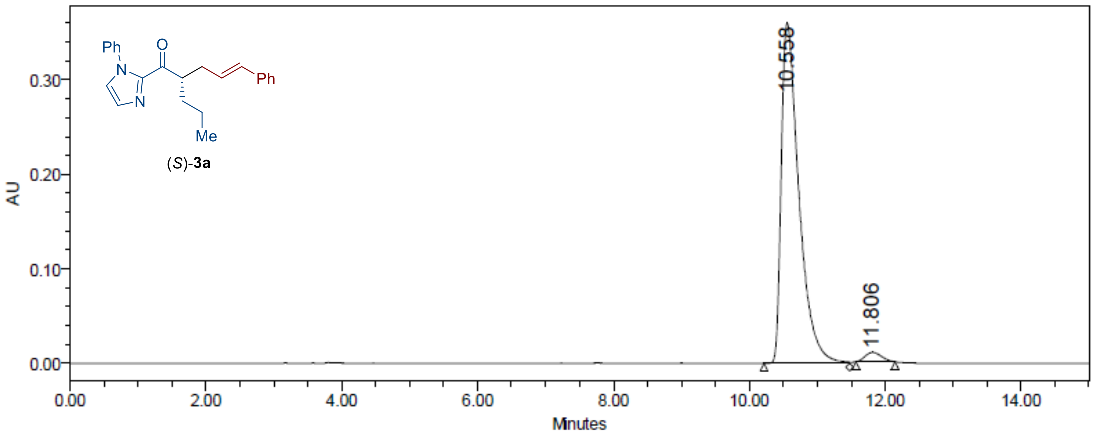

	RT	Area	% Area	Height
1	10.558	6493084	97.48	359694
2	11.806	167690	2.52	9968

rac-**3b**

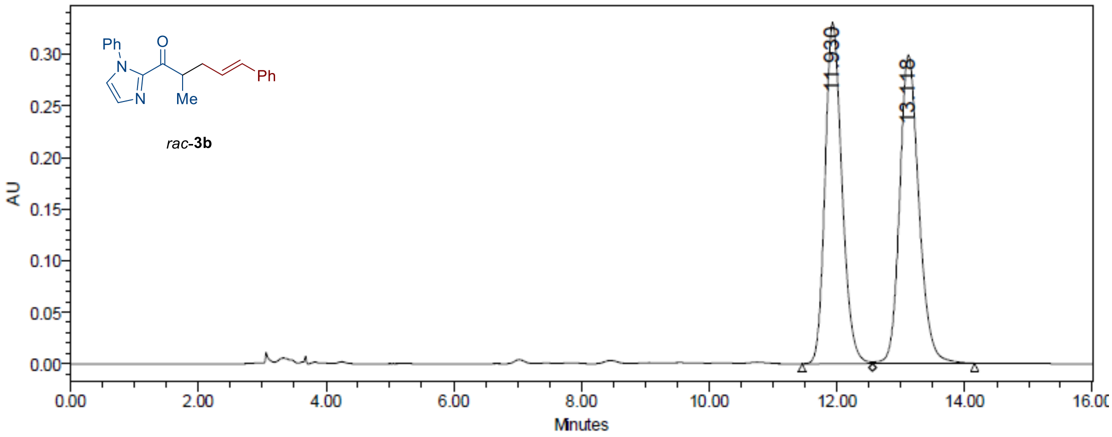

	RT	Area	% Area	Height
1	11.930	6087301	49.38	330245
2	13.118	6239772	50.62	298909

(*R*)-**3b**

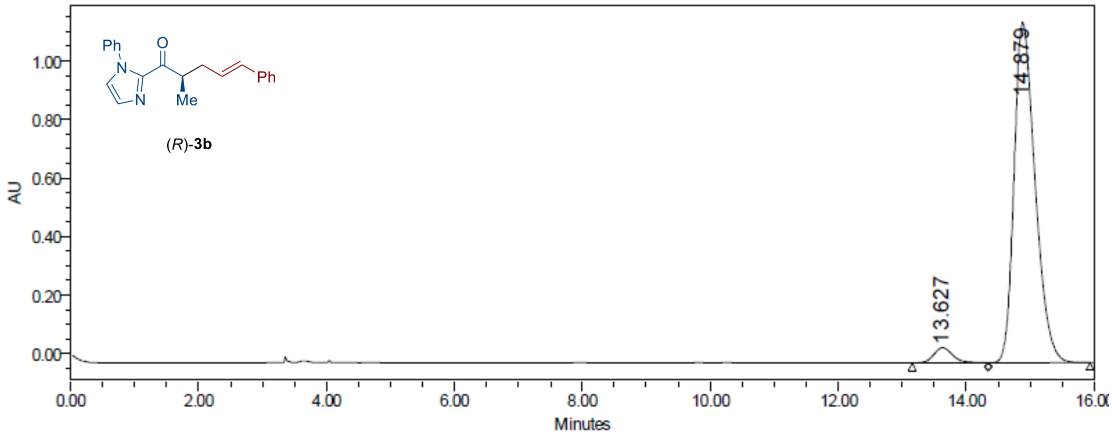

	RT	Area	% Area	Height
1	13.627	1081886	3.91	51986
2	14.879	26601912	96.09	1160802

Supplementary Figure 82. HPLC spectra of compound **3b**

rac-**3c**

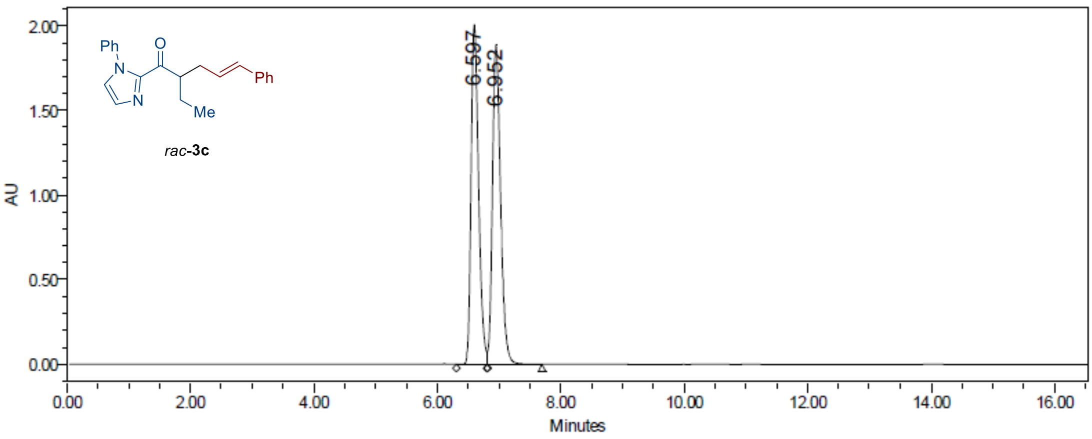

	RT	Area	% Area	Height
1	6.597	17124640	49.75	2006317
2	6.952	17296775	50.25	1888729

(*R*)-**3c**

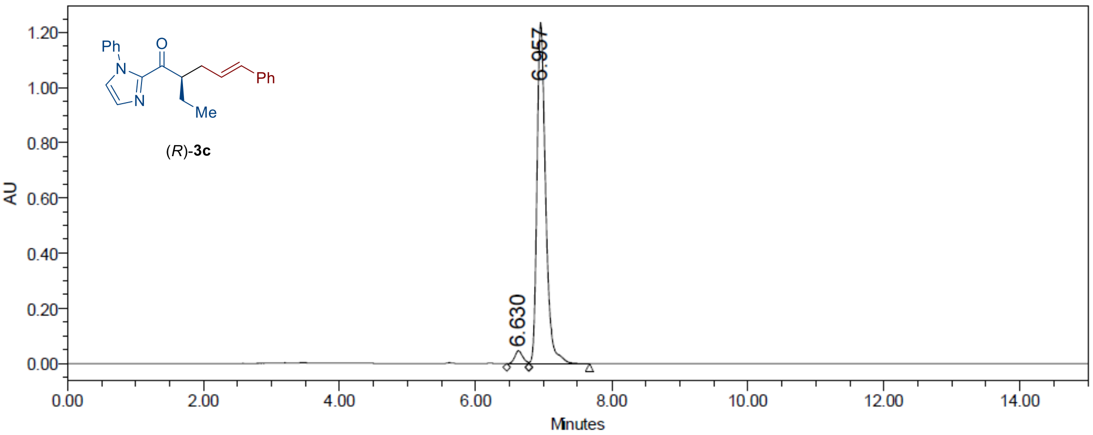

	RT	Area	% Area	Height
1	6.630	374849	3.32	46312
2	6.957	10923328	96.68	1234864

Supplementary Figure 83. HPLC spectra of compound **3c**

rac-**3d**

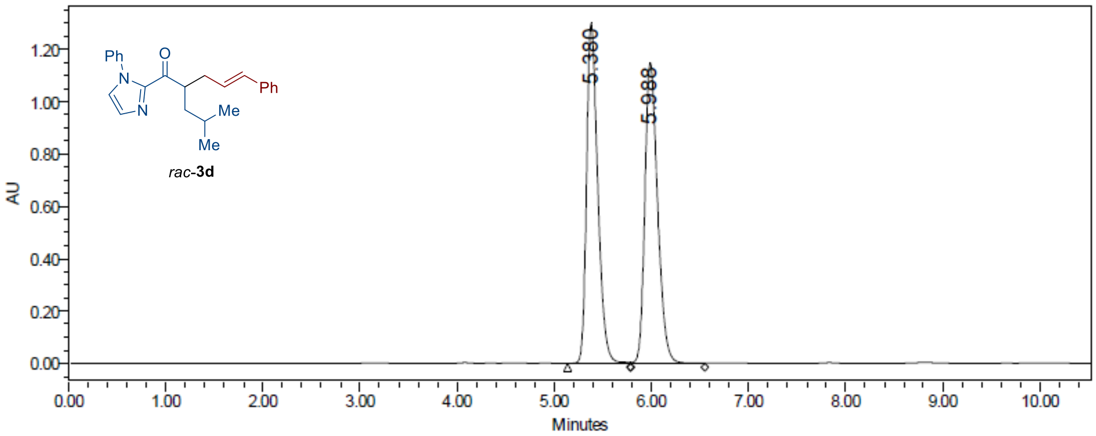

	RT	Area	% Area	Height
1	5.380	10420372	49.77	1299656
2	5.988	10515835	50.23	1150281

(*R*)-**3d**

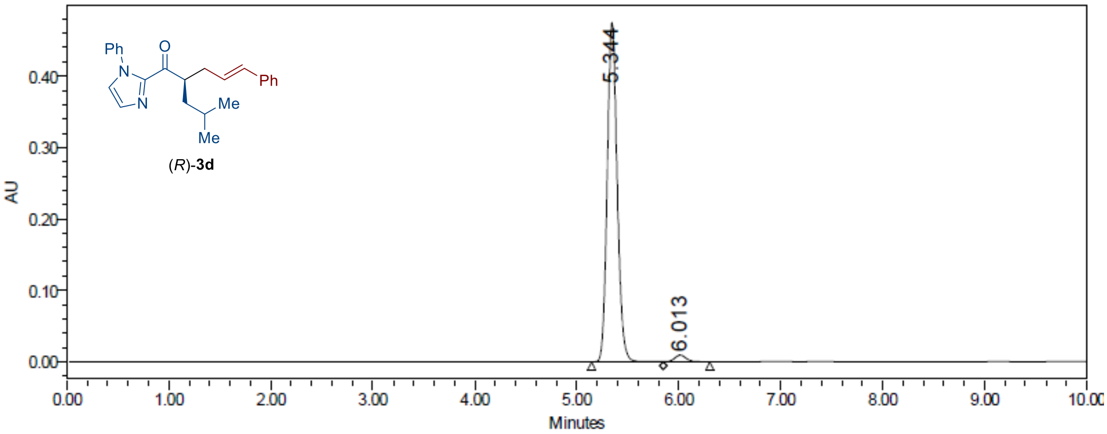

	RT	Area	% Area	Height
1	5.344	3201264	97.76	474832
2	6.013	73366	2.24	9222

Supplementary Figure 84. HPLC spectra of compound **3d**

rac-**3e**

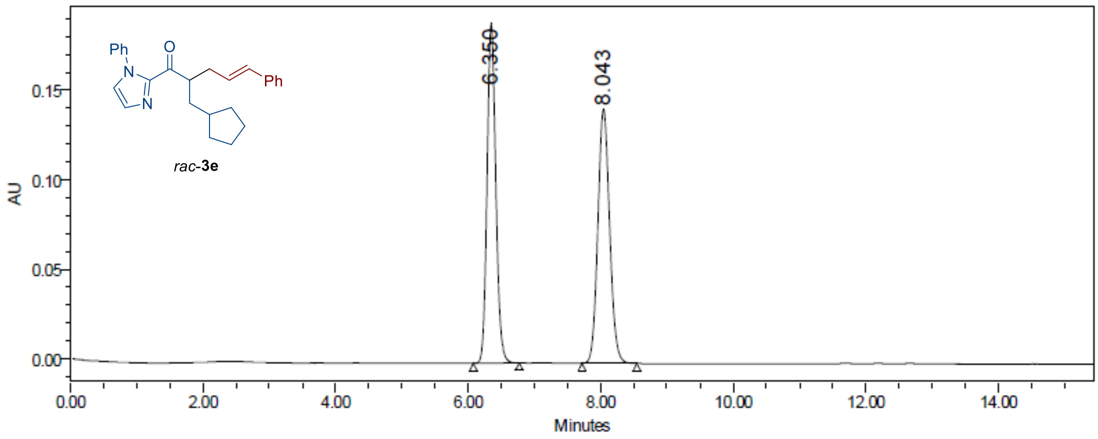

	RT	Area	% Area	Height
1	6.350	1748442	49.78	189674
2	8.043	1764115	50.22	141966

(*R*)-**3e**

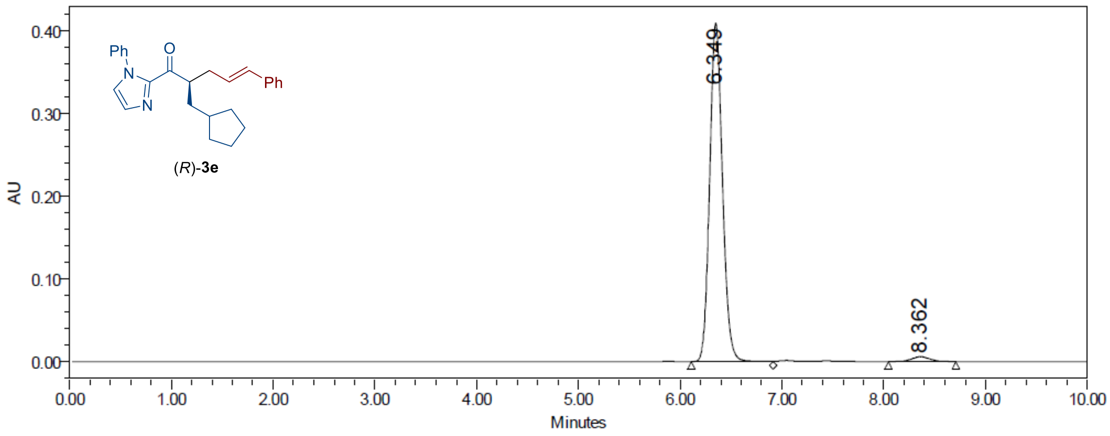

	RT	Area	% Area	Height
1	6.349	3584319	97.95	408913
2	8.362	75043	2.05	5860

Supplementary Figure 85. HPLC spectra of compound **3e**

rac-**3f**

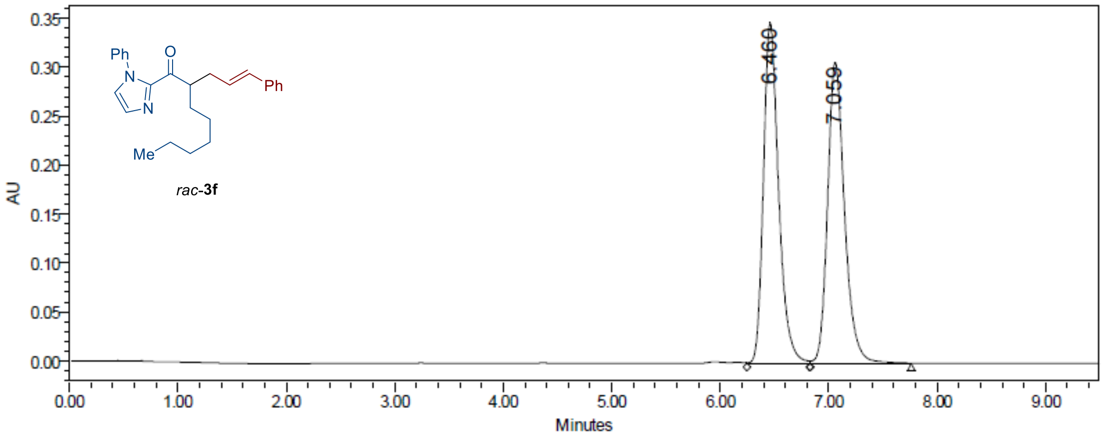

	RT	Area	% Area	Height
1	6.460	3367870	50.19	347493
2	7.059	3342962	49.81	307374

(R)-**3f**

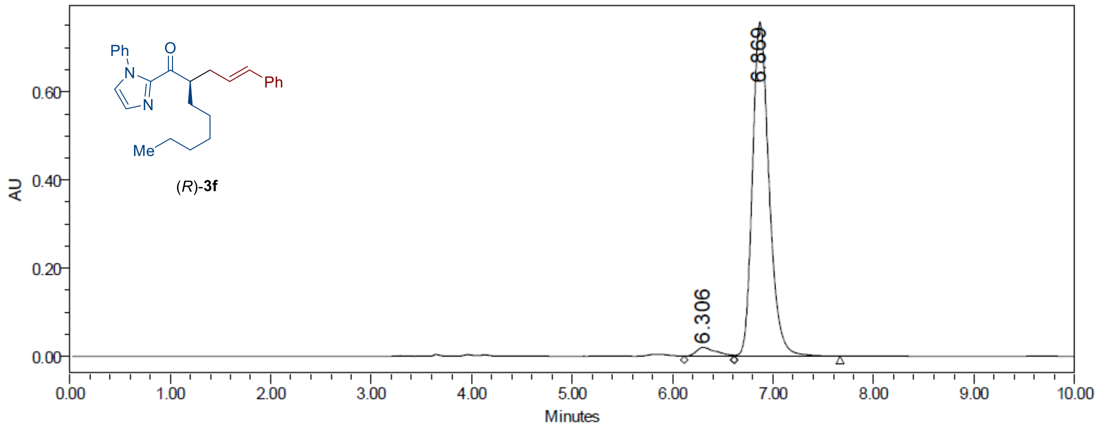

	RT	Area	% Area	Height
1	6.306	270383	3.02	20055
2	6.869	8680748	96.98	756651

(*S*)-**3f**

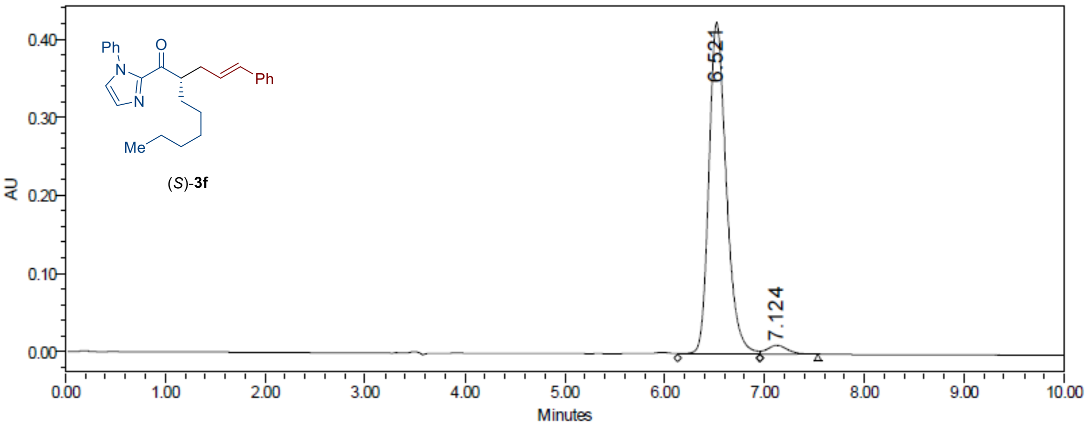

	RT	Area	% Area	Height
1	6.521	5178556	97.00	424140
2	7.124	160056	3.00	11365

Supplementary Figure 86. HPLC spectra of compound **3f**

rac-**3g**

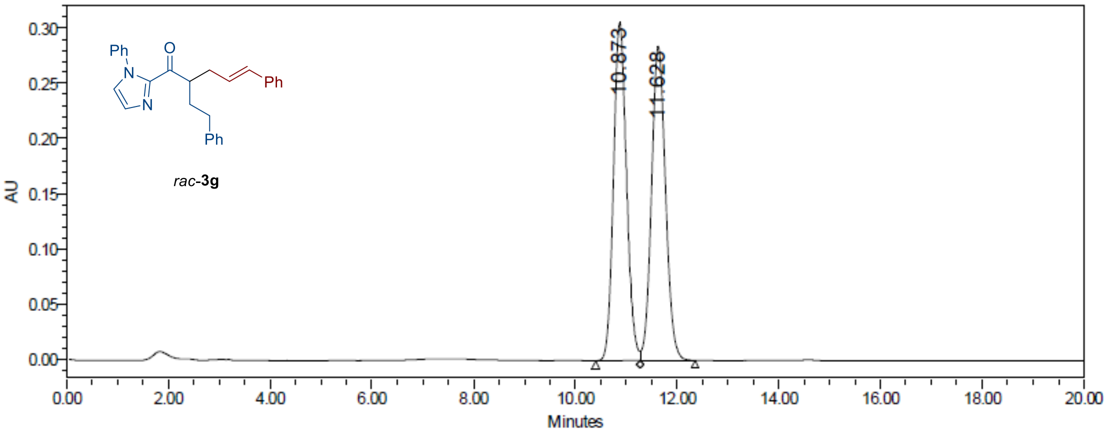

	RT	Area	% Area	Height
1	10.873	5407959	49.57	306144
2	11.628	5502806	50.43	284757

(*R*)-**3g**

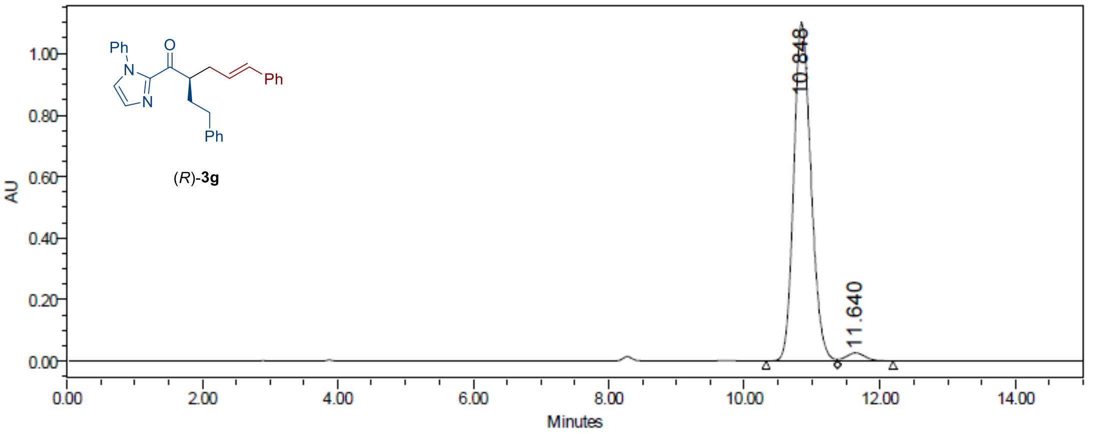

	RT	Area	% Area	Height
1	10.848	19386099	97.47	1098845
2	11.640	504110	2.53	25933

Supplementary Figure 87. HPLC spectra of compound **3g**

rac-**3h**

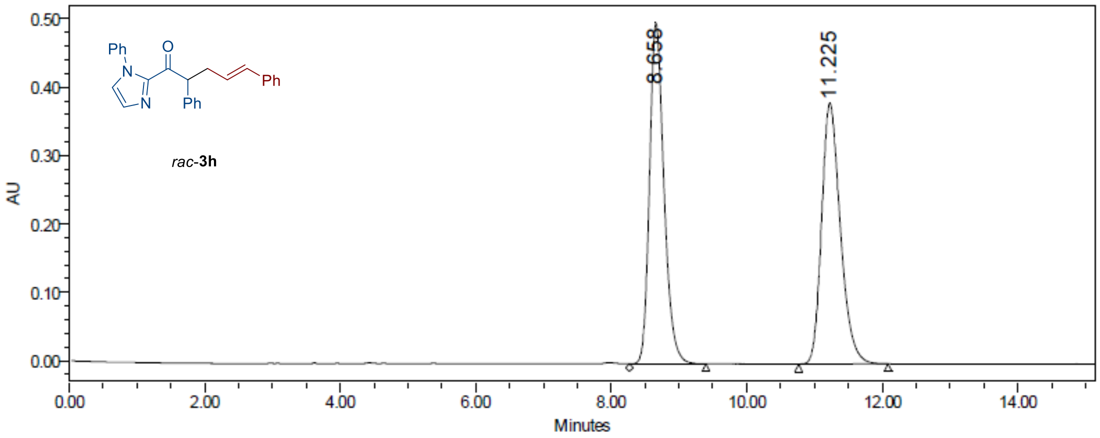

	RT	Area	% Area	Height
1	8.658	7430743	50.32	498234
2	11.225	7337006	49.68	381010

(*S*)-**3h**

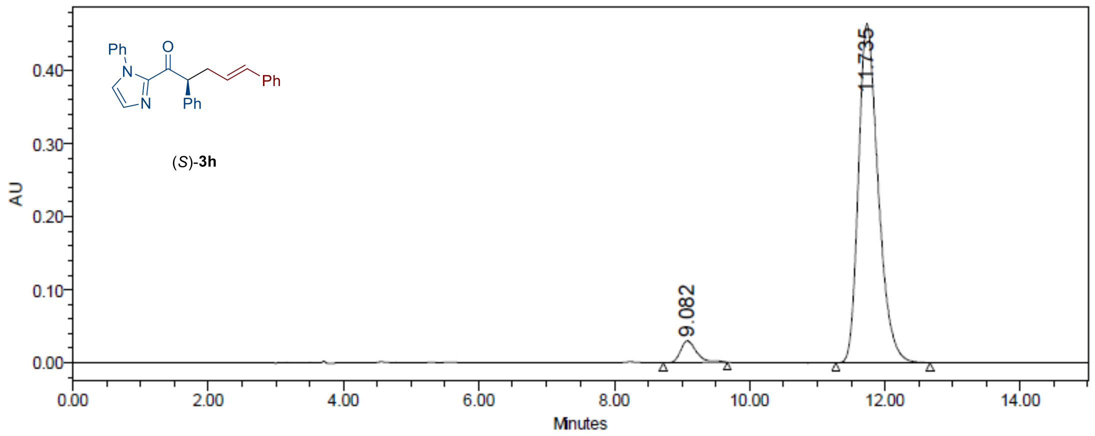

	RT	Area	% Area	Height
1	9.082	495762	4.99	29643
2	11.735	9446451	95.01	463600

Supplementary Figure 88. HPLC spectra of compound **3h**

rac-**3i**

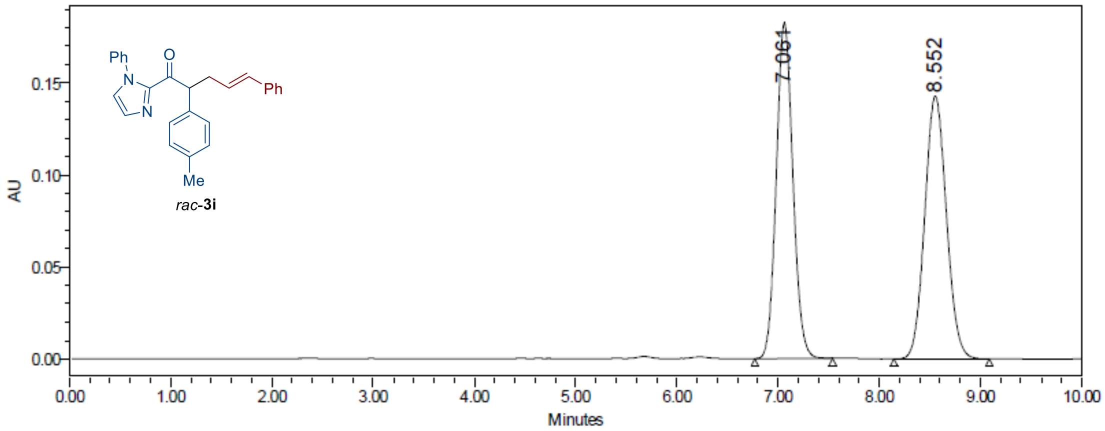

	RT	Area	% Area	Height
1	7.061	2090475	49.63	182290
2	8.552	2121751	50.37	142865

(*S*)-**3i**

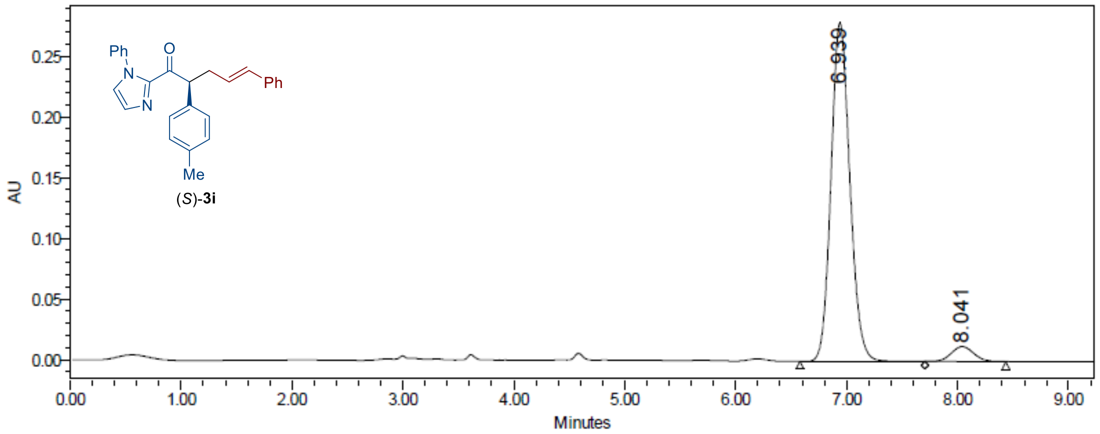

	RT	Area	% Area	Height
1	6.939	3303333	94.99	278989
2	8.041	174348	5.01	12366

Supplementary Figure 89. HPLC spectra of compound **3i**

rac-**3j**

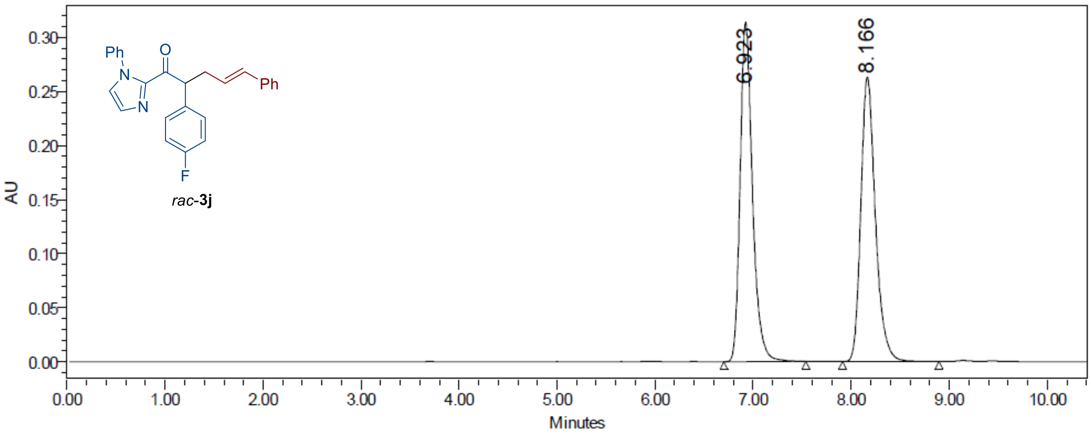

	RT	Area	% Area	Height
1	6.923	2737162	50.29	313489
2	8.166	2705166	49.71	262889

(*S*)-**3j**

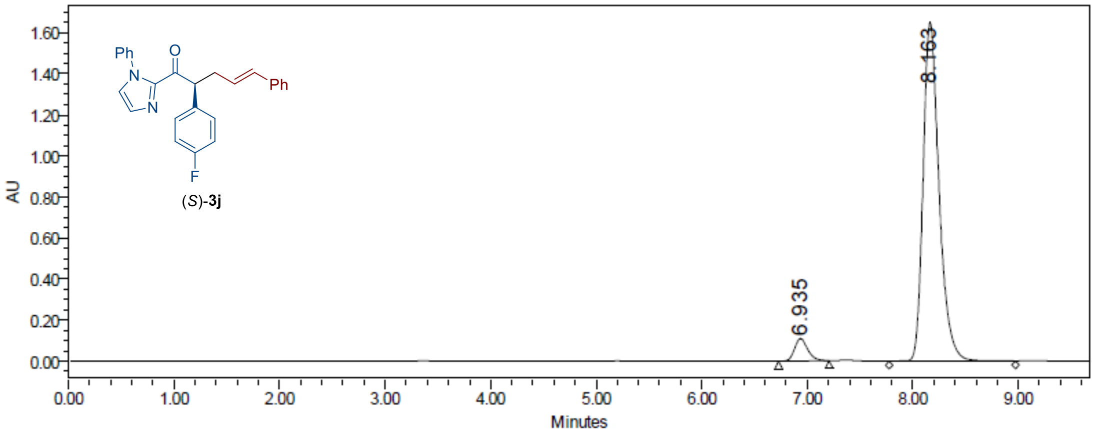

	RT	Area	% Area	Height
1	6.935	930033	5.12	109312
2	8.163	17236191	94.88	1648222

Supplementary Figure 90. HPLC spectra of compound **3j**

rac-**3k**

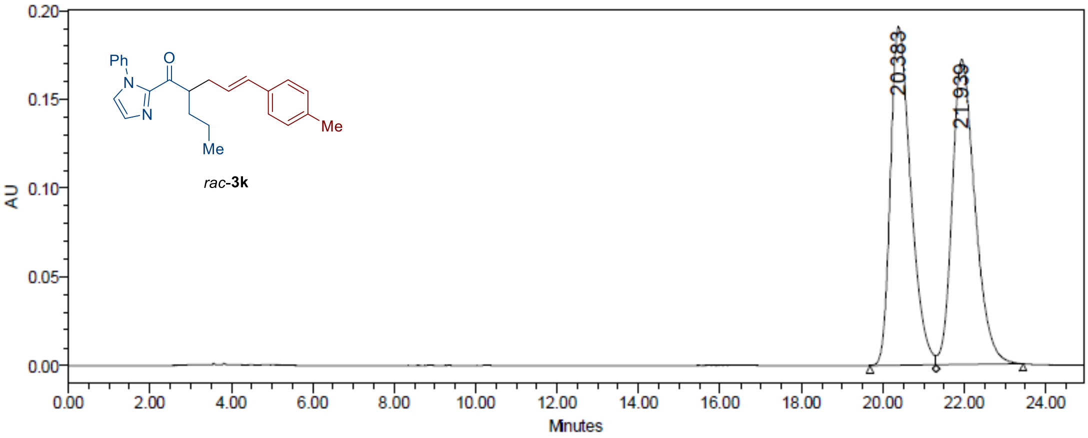

	RT	Area	% Area	Height
1	20.383	6540790	49.51	190513
2	21.939	6669031	50.49	171937

(*R*)-**3k**

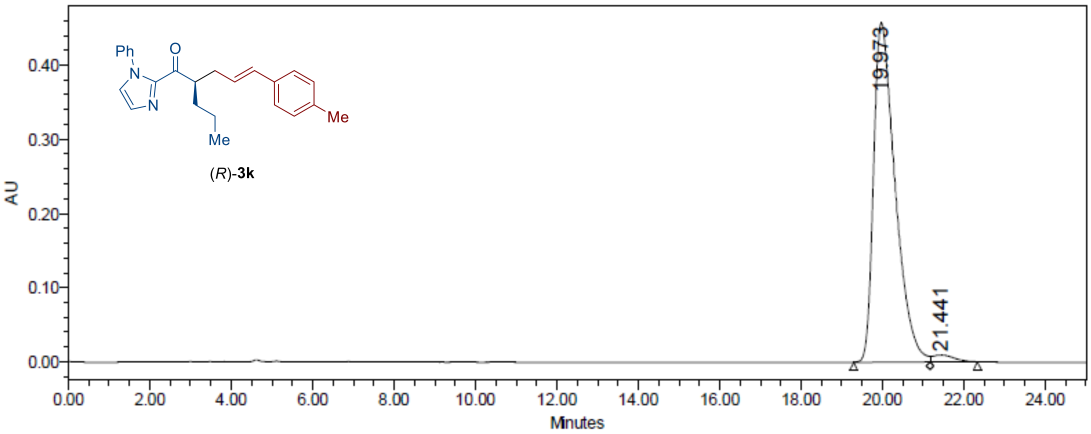

	RT	Area	% Area	Height
1	19.973	16811310	97.98	457450
2	21.441	346424	2.02	9234

Supplementary Figure 91. HPLC spectra of compound **3k**

rac-**3l**

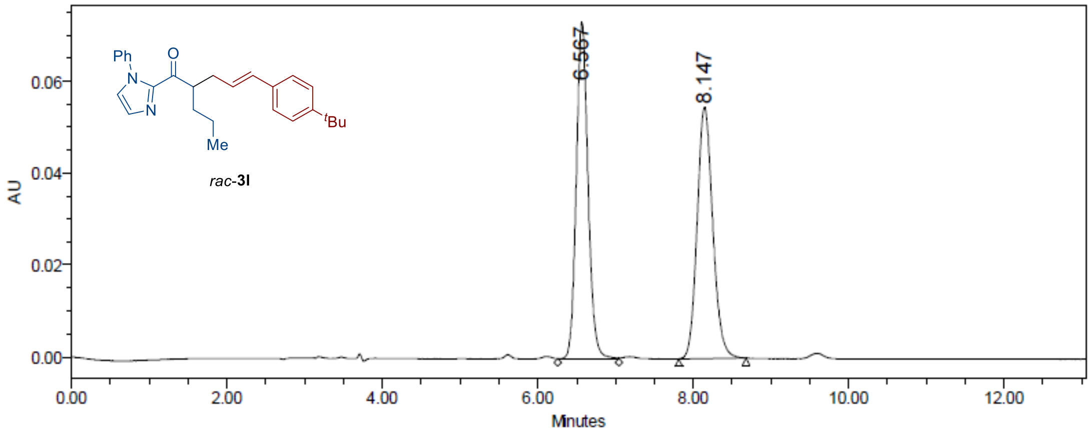

	RT	Area	% Area	Height
1	6.567	764422	49.92	73174
2	8.147	767002	50.08	54785

(*R*)-**3l**

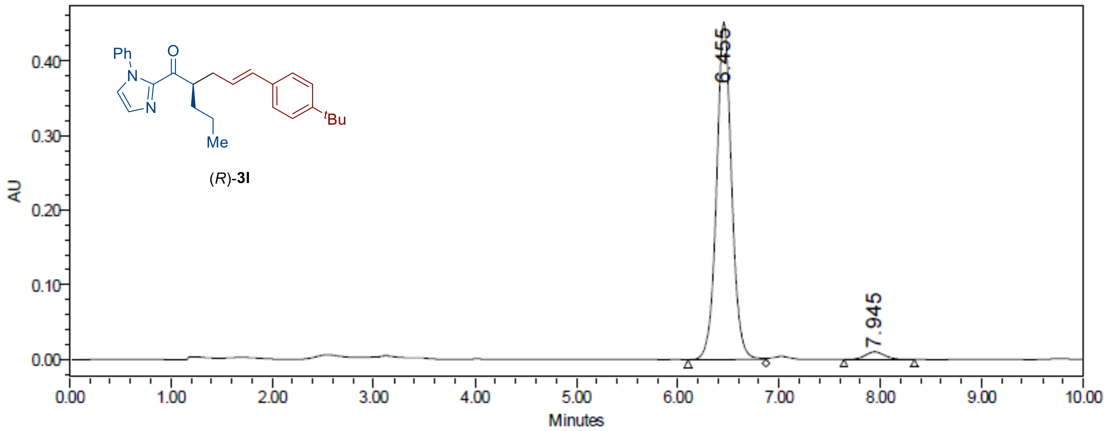

	RT	Area	% Area	Height
1	6.455	4684028	97.13	450122
2	7.945	138323	2.87	10189

Supplementary Figure 92. HPLC spectra of compound **3l**

rac-**3m**

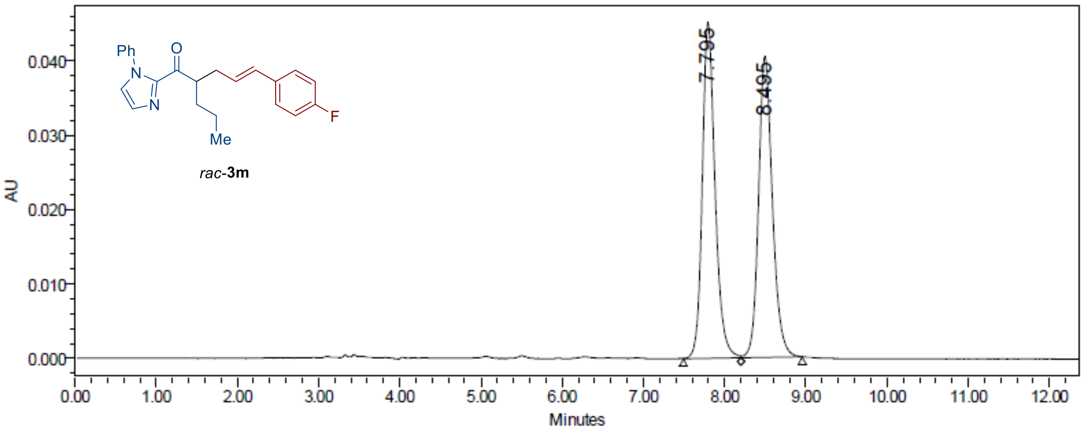

	RT	Area	% Area	Height
1	7.795	490464	50.59	45025
2	8.495	479114	49.41	40408

(*R*)-**3m**

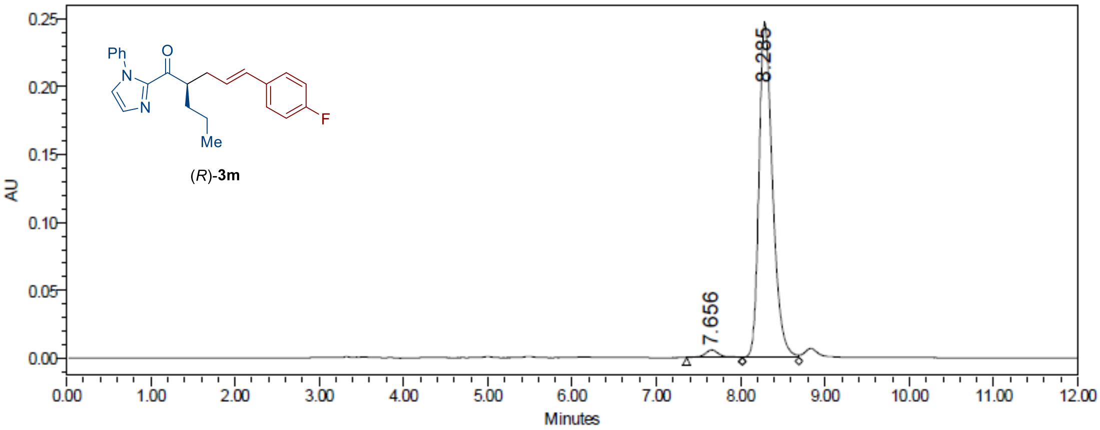

	RT	Area	% Area	Height
1	7.656	68528	2.42	5612
2	8.285	2765072	97.58	246749

Supplementary Figure 93. HPLC spectra of compound **3m**

rac-**3n**

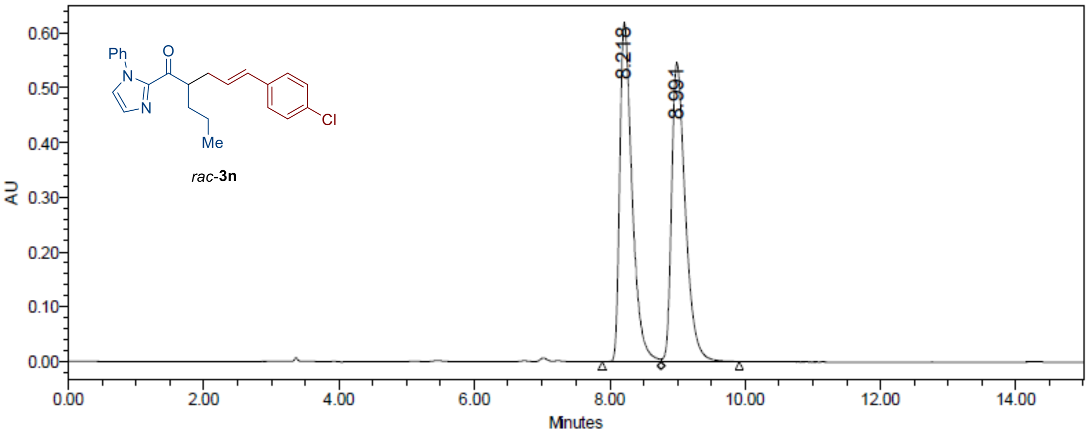

	RT	Area	% Area	Height
1	8.218	7876547	50.34	619083
2	8.991	7771575	49.66	546138

(*R*)-**3n**

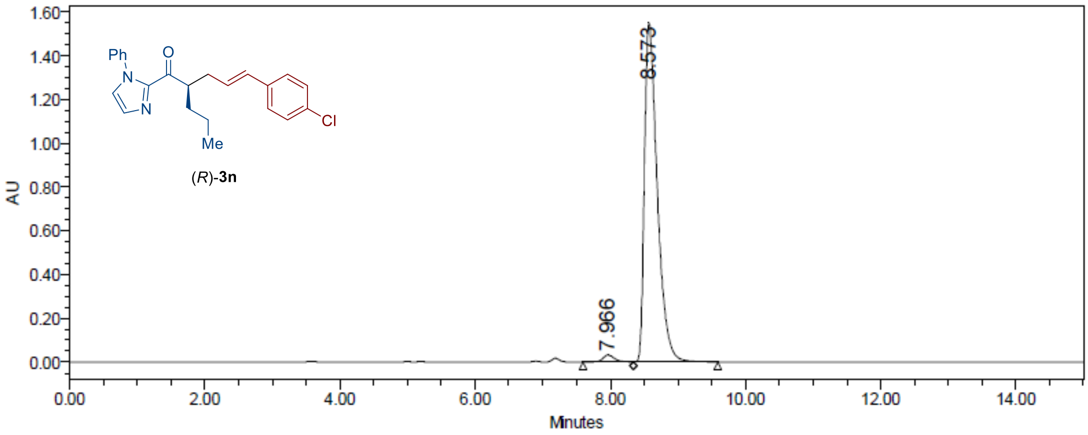

	RT	Area	% Area	Height
1	7.966	385186	1.87	32248
2	8.573	20176287	98.13	1548736

Supplementary Figure 94. HPLC spectra of compound **3n**

rac-**3o**

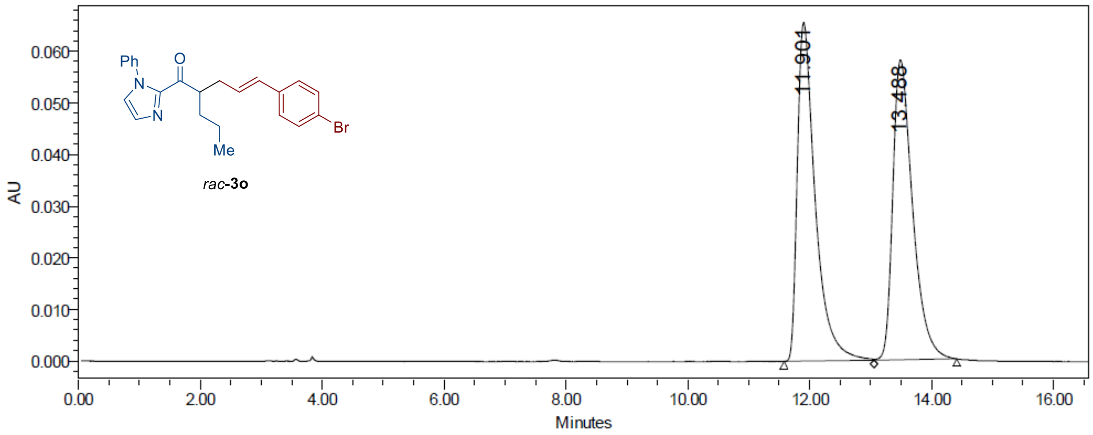

	RT	Area	% Area	Height
1	11.901	1338579	50.53	65555
2	13.488	1310330	49.47	58028

(*R*)-**3o**

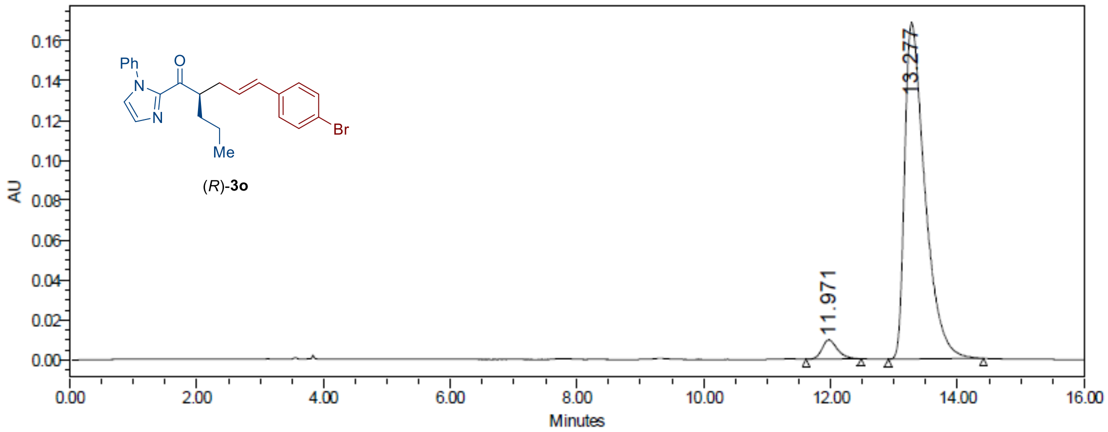

	RT	Area	% Area	Height
1	11.971	162933	4.16	9652
2	13.277	3752600	95.84	168154

Supplementary Figure 95. HPLC spectra of compound **3o**

rac-**3p**

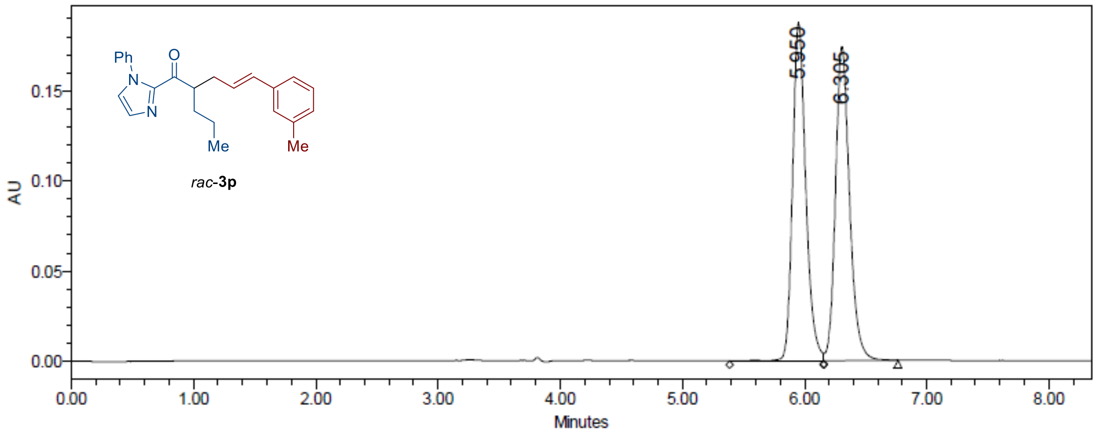

	RT	Area	% Area	Height
1	5.950	1409082	50.23	187433
2	6.305	1396173	49.77	174287

(*R*)-**3p**

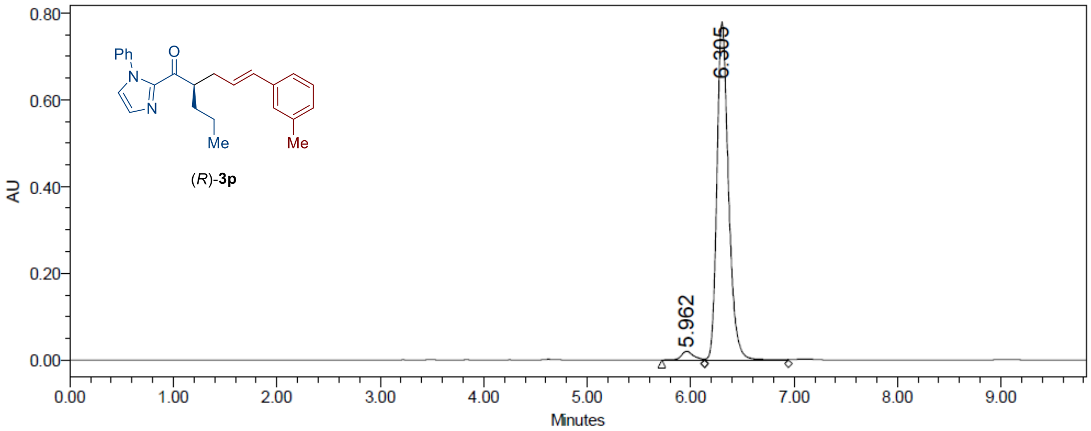

	RT	Area	% Area	Height
1	5.962	160685	2.59	19988
2	6.305	6050328	97.41	778797

Supplementary Figure 96. HPLC spectra of compound **3p**

rac-**3q**

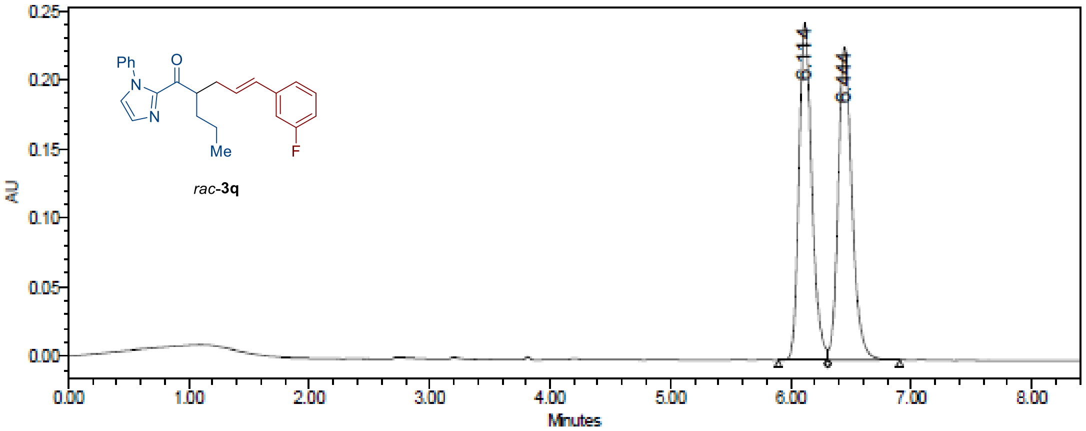

	RT	Area	% Area	Height
1	6.114	1807723	49.97	243061
2	6.444	1809613	50.03	225945

(*R*)-**3q**

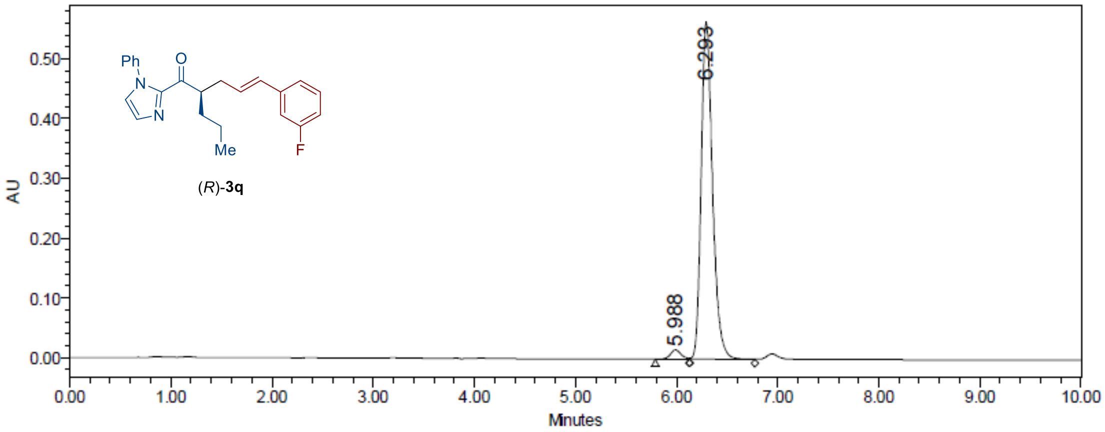

	RT	Area	% Area	Height
1	5.988	113879	2.52	15490
2	6.293	4402386	97.48	564037

Supplementary Figure 97. HPLC spectra of compound **3q**

rac-**3r**

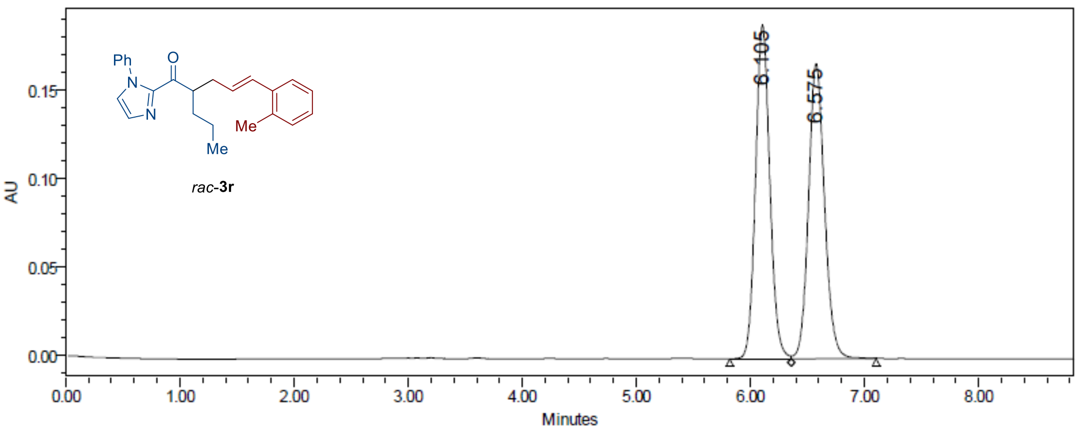

	RT	Area	% Area	Height
1	6.105	1634255	49.97	188472
2	6.575	1635944	50.03	166861

(*R*)-**3r**

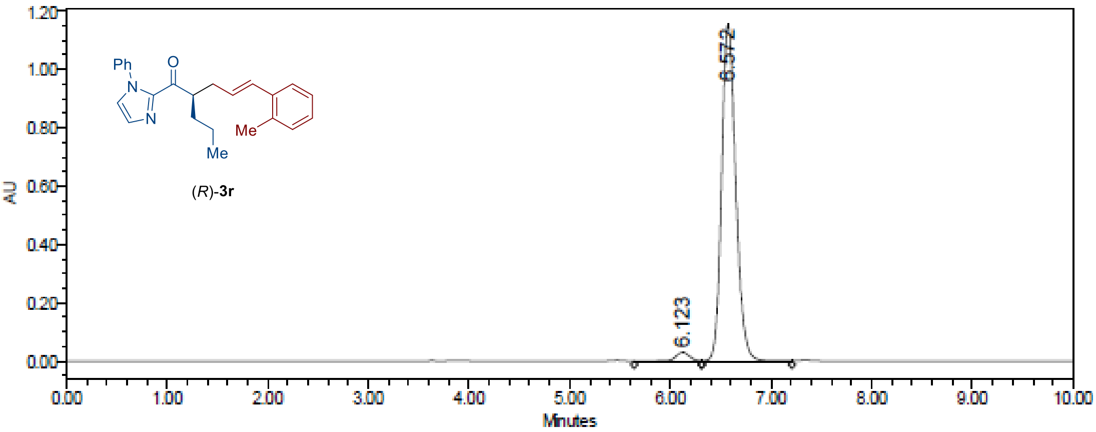

	RT	Area	% Area	Height
1	6.123	275862	2.41	29808
2	6.572	11155985	97.59	1153105

Supplementary Figure 98. HPLC spectra of compound **3r**

rac-**3s**

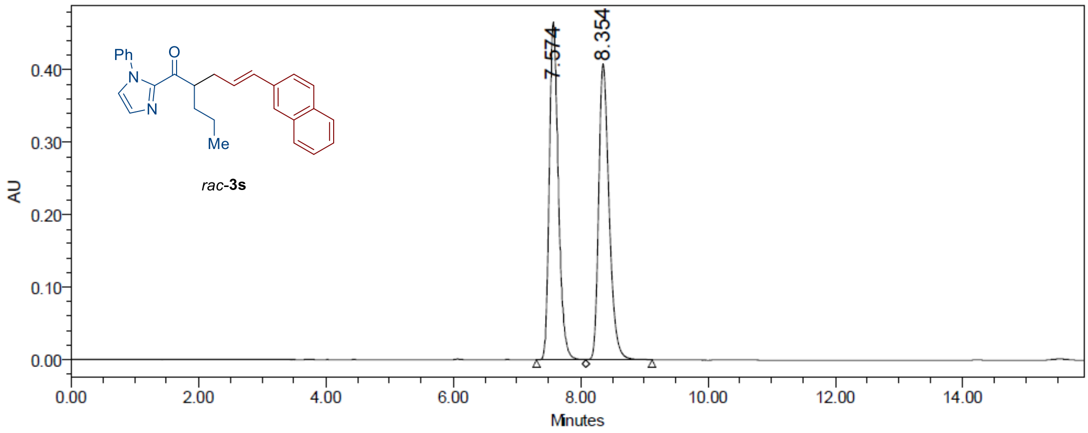

	RT	Area	% Area	Height
1	7.574	4601964	49.60	466188
2	8.354	4676288	50.40	408352

(*R*)-**3s**

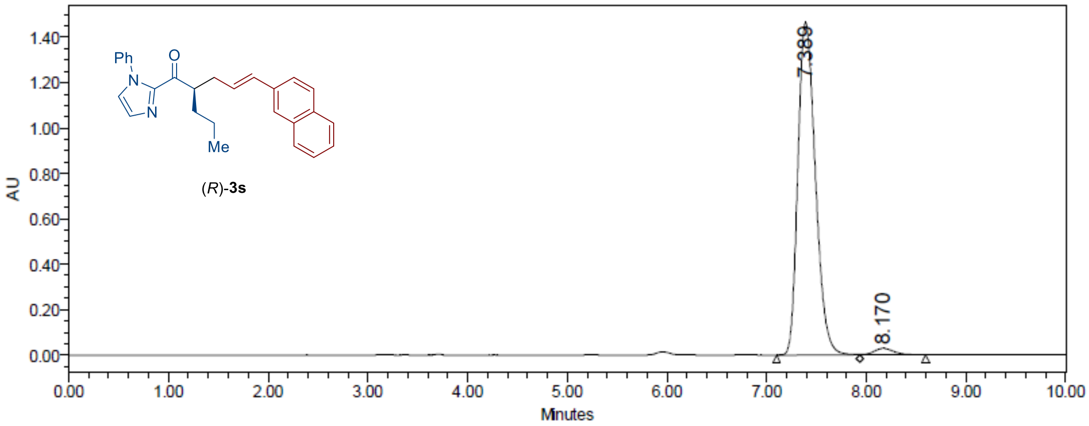

	RT	Area	% Area	Height
1	7.389	17851055	97.86	1466093
2	8.170	390989	2.14	28272

Supplementary Figure 99. HPLC spectra of compound **3s**

rac-**3t**

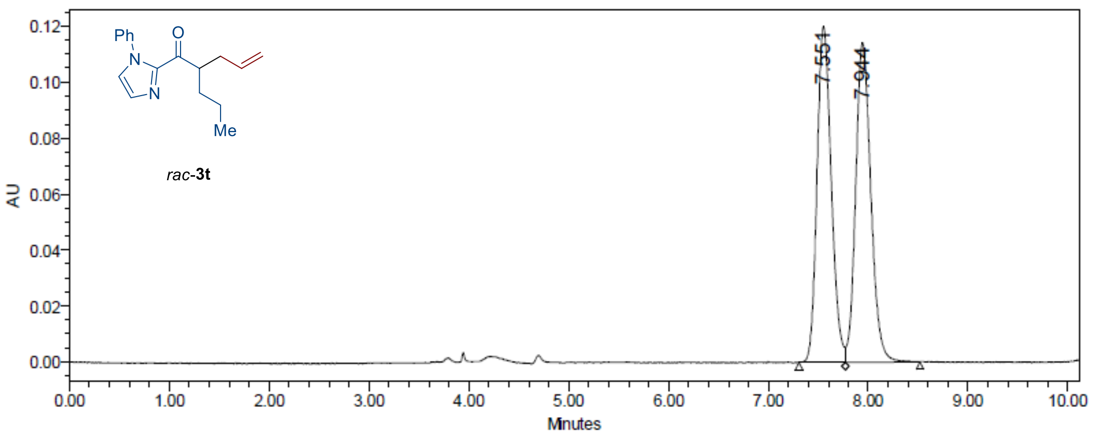

	RT	Area	% Area	Height
1	7.551	1184716	49.51	119914
2	7.944	1208255	50.49	114098

(*R*)-**3t**

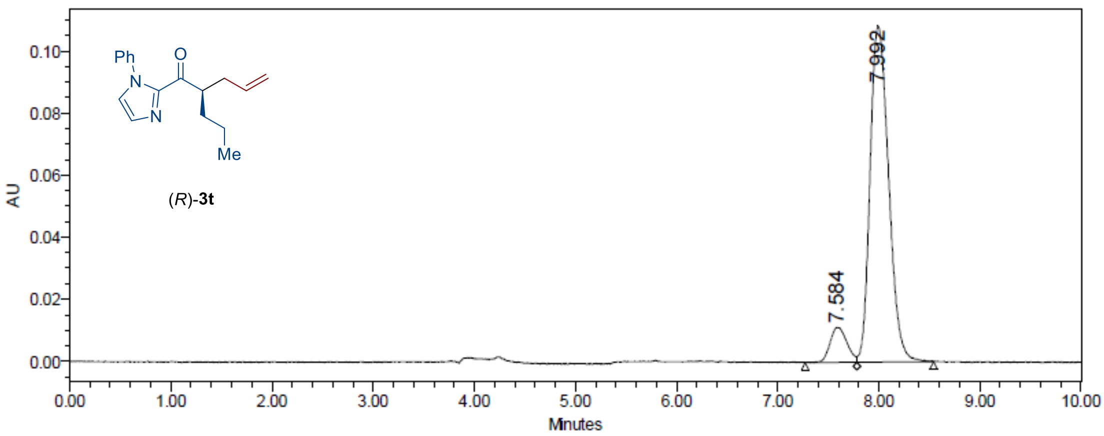

	RT	Area	% Area	Height
1	7.584	133283	8.78	11392
2	7.992	1384535	91.22	108422

Supplementary Figure 100. HPLC spectra of compound **3t**

rac-**3u**

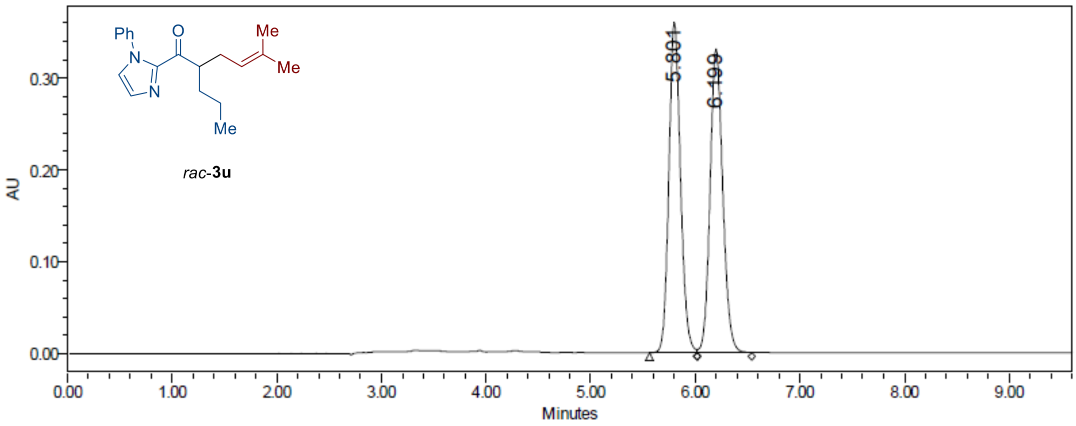

	RT	Area	% Area	Height
1	5.801	2757035	49.82	359046
2	6.199	2777182	50.18	330343

(*R*)-**3u**

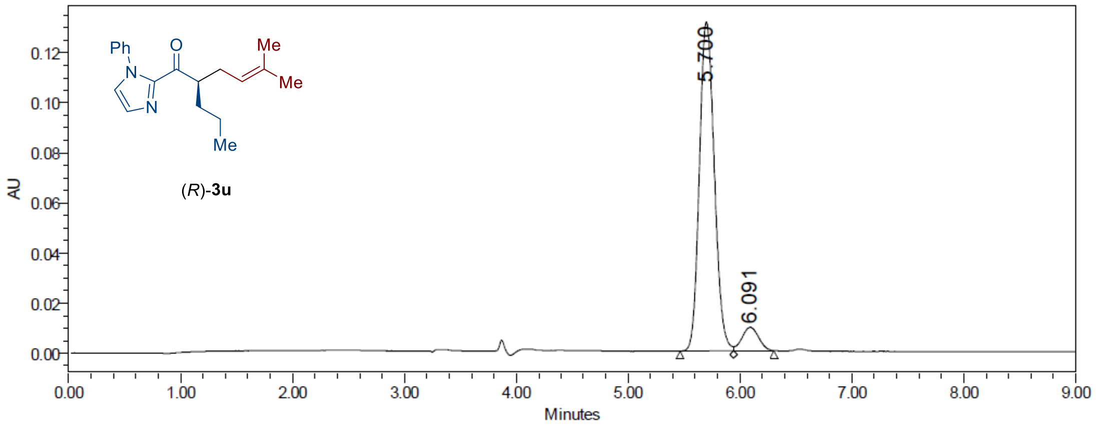

	RT	Area	% Area	Height
1	5.700	1220960	92.65	131433
2	6.091	96872	7.35	9342

Supplementary Figure 101. HPLC spectra of compound **3u**

rac-**4a**

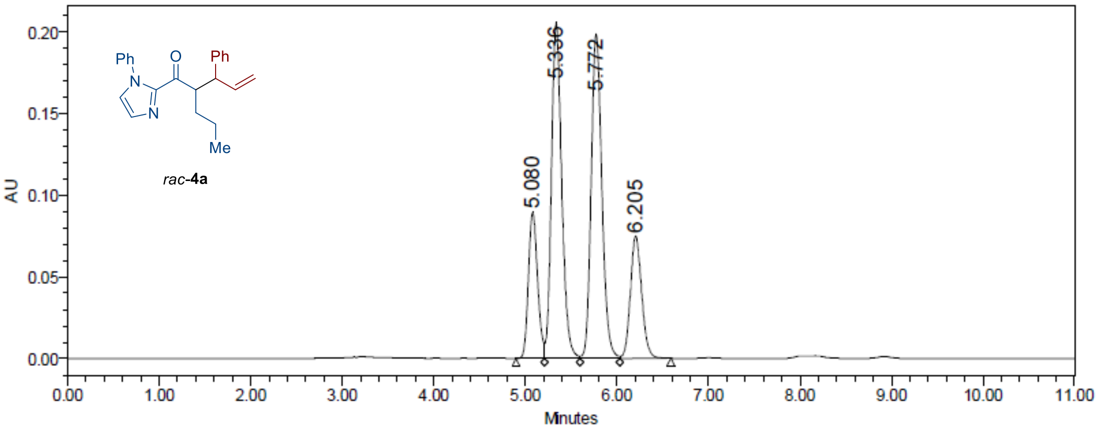

	RT	Area	% Area	Height
1	5.080	625474	13.98	89418
2	5.336	1591672	35.57	205465
3	5.772	1617565	36.15	198508
4	6.205	640305	14.31	74903

(*R*,*S*)-**4a**

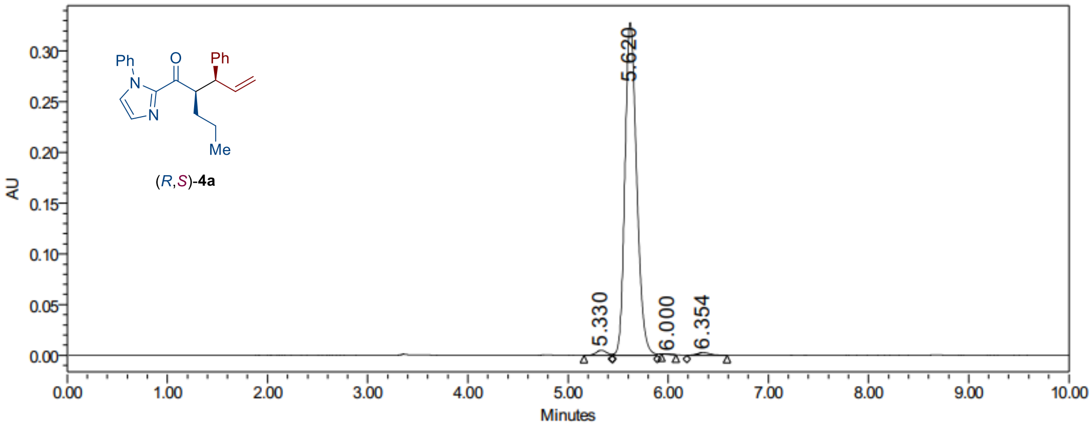

	RT	Area	% Area	Height
1	5.330	37145	1.36	5100
2	5.620	2662850	97.68	328374
3	6.000	2010	0.07	403
4	6.354	24190	0.89	2730

(*S*,*S*)-**4a**

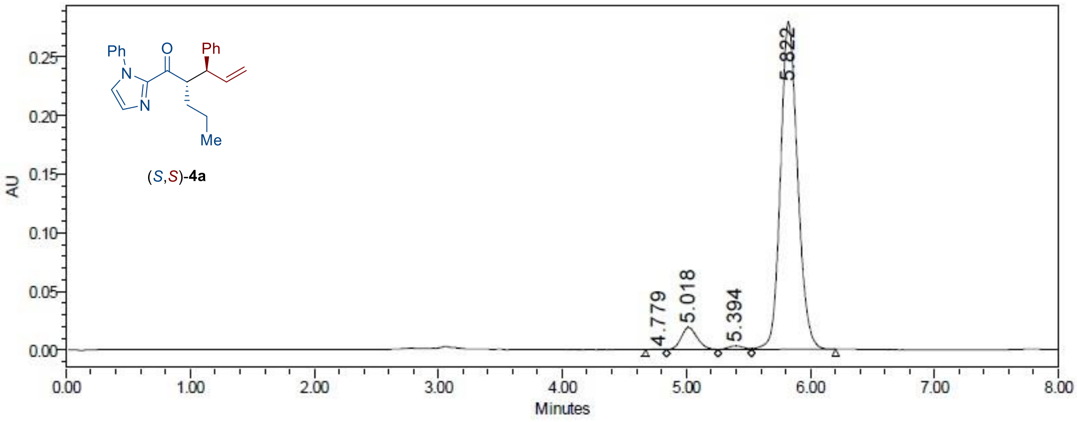

	RT	Area	% Area	Height
1	4.779	2796	0.10	415
2	5.018	168422	5.86	18783
3	5.394	25434	0.89	3018
4	5.822	2676925	93.16	279456

(*R*,*R*)-**4a**

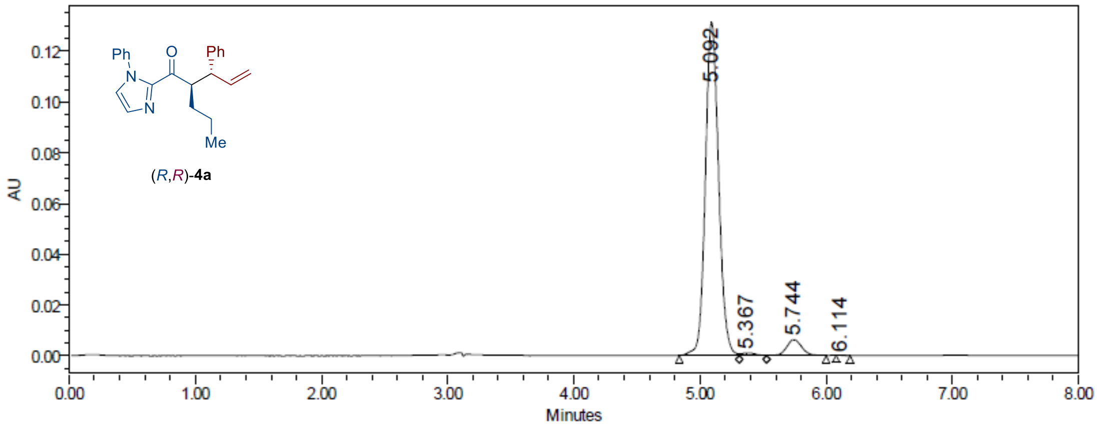

	RT	Area	% Area	Height
1	5.092	953671	94.16	131258
2	5.367	7468	0.74	1008
3	5.744	51571	5.09	6310
4	6.114	153	0.02	51

(*S*,*R*)-**4a**

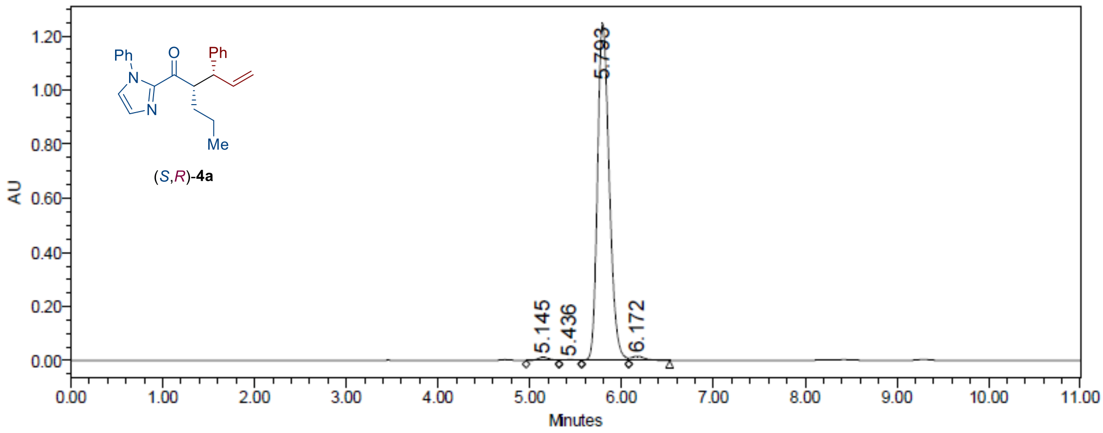

	RT	Area	% Area	Height
1	5.145	100124	0.88	12514
2	5.436	24098	0.21	2777
3	5.793	11138826	97.61	1245451
4	6.172	148976	1.31	15210

Supplementary Figure 102. HPLC spectra of compound **4a**

rac-**4b**

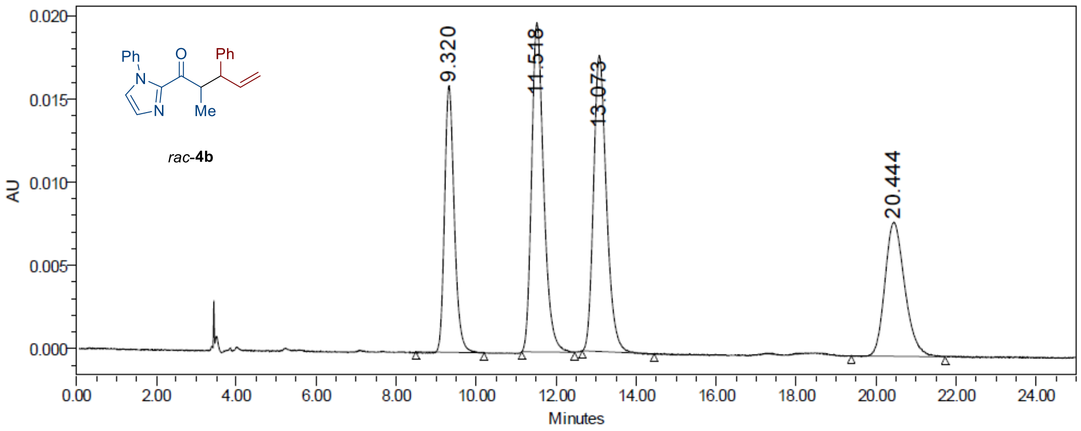

	RT	Area	% Area	Height
1	9.320	270451	19.90	16031
2	11.518	412588	30.36	19759
3	13.073	399032	29.36	17778
4	20.444	277019	20.38	8045

(*R*,*S*)-**4b**

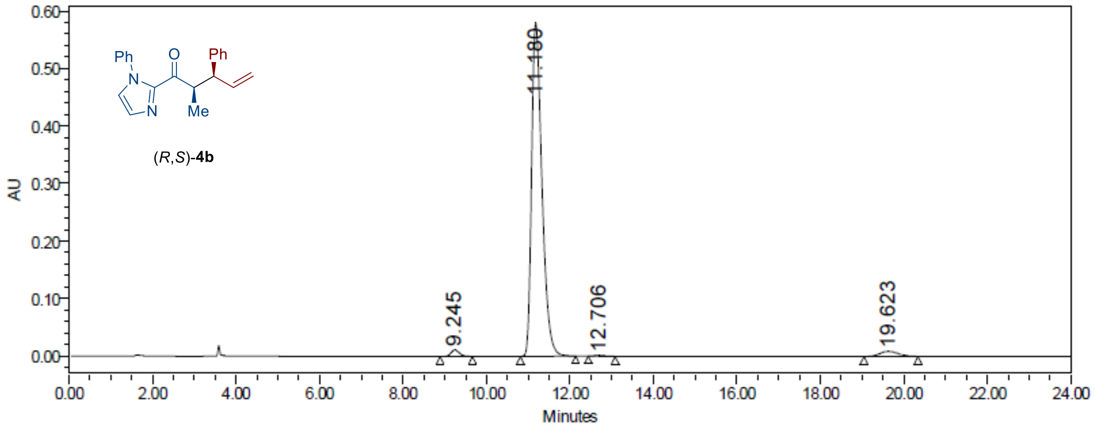

	RT	Area	% Area	Height
1	9.245	159877	1.52	11537
2	11.180	10045792	95.56	579016
3	12.706	30626	0.29	1720
4	19.623	275938	2.62	9074

Supplementary Figure 103. HPLC spectra of compound **4b**

rac-**4c**

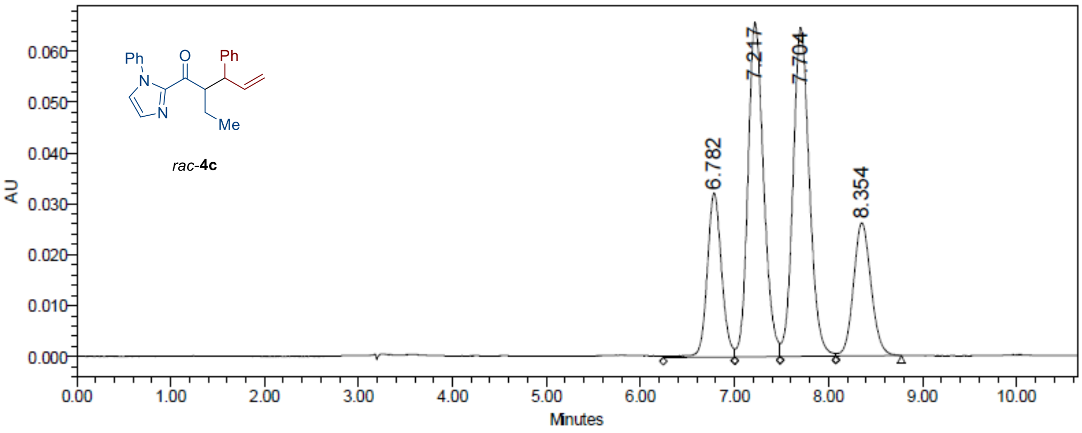

	RT	Area	% Area	Height
1	6.782	342521	15.40	32236
2	7.217	760007	34.17	65692
3	7.704	782792	35.20	64657
4	8.354	338722	15.23	26188

(*R*,*S*)-**4c**

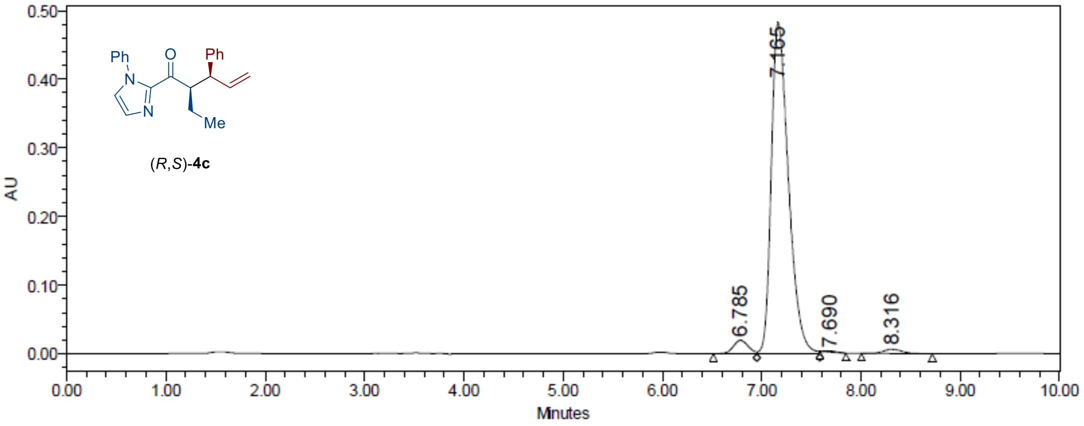

	RT	Area	% Area	Height
1	6.785	204987	3.37	19751
2	7.165	5775461	95.01	482207
3	7.690	13135	0.22	1649
4	8.316	85513	1.41	6571

Supplementary Figure 104. HPLC spectra of compound **4c**

rac-**4d**

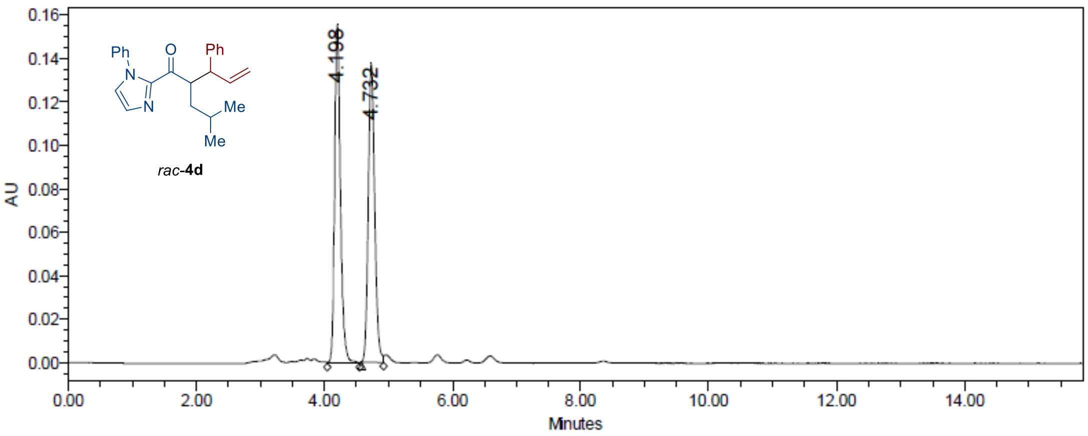

	RT	Area	% Area	Height
1	4.198	976724	50.43	155626
2	4.732	959929	49.57	137444

(*R*,*S*)-**4d**

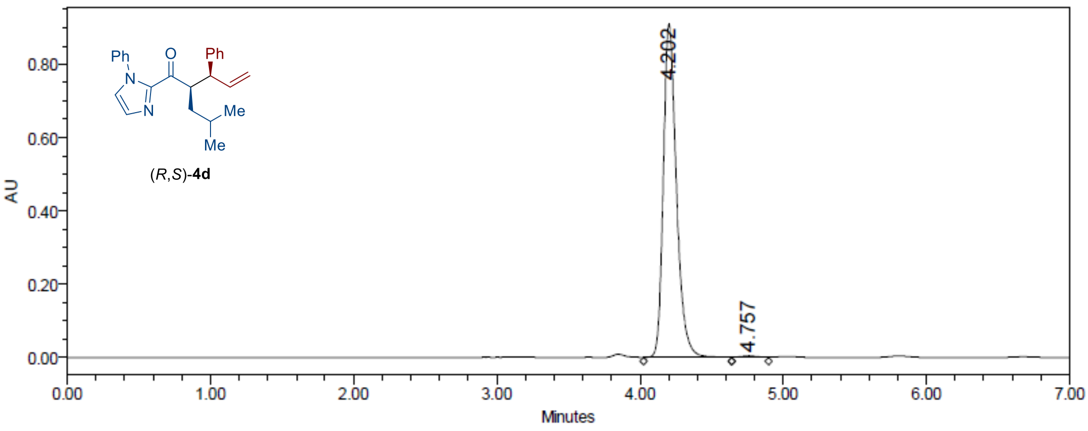

	RT	Area	% Area	Height
1	4.202	5789791	99.35	908887
2	4.757	37632	0.65	4642

Supplementary Figure 105. HPLC spectra of compound **4d**

rac-**4e**

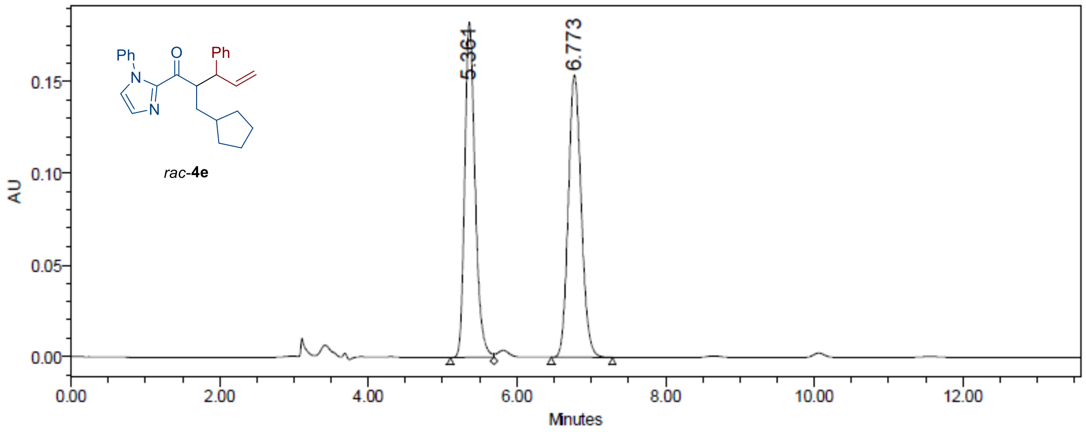

	RT	Area	% Area	Height
1	5.361	1766279	48.79	182426
2	6.773	1853785	51.21	153879

(*R*,*S*)-**4e**

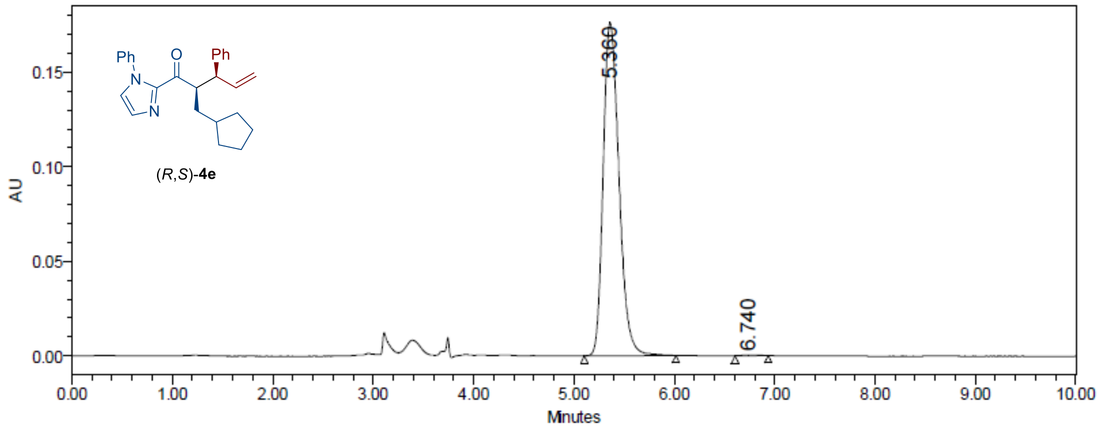

	RT	Area	% Area	Height
1	5.360	1925388	99.74	175812
2	6.740	5102	0.26	473

Supplementary Figure 106. HPLC spectra of compound **4e**

rac-**4f**

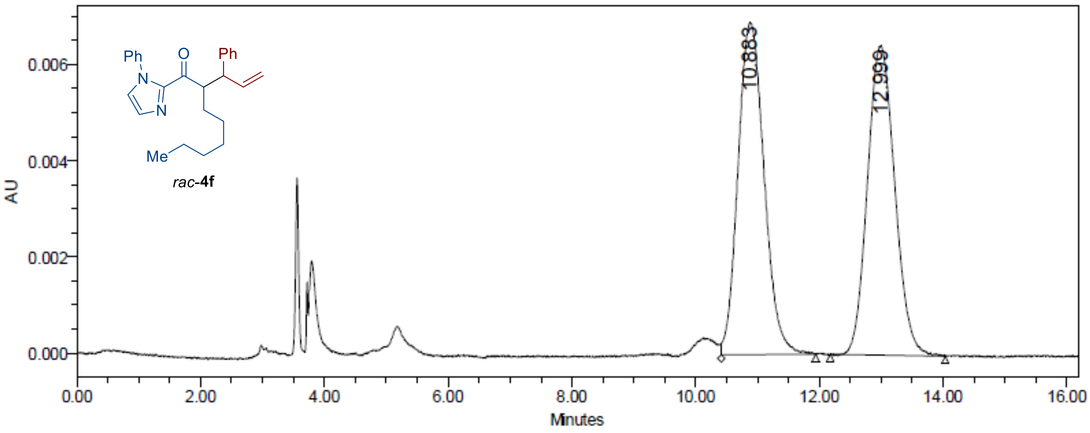

	RT	Area	% Area	Height
1	10.883	202350	50.63	6889
2	12.999	197319	49.37	6427

(*R*,*S*)-**4f**

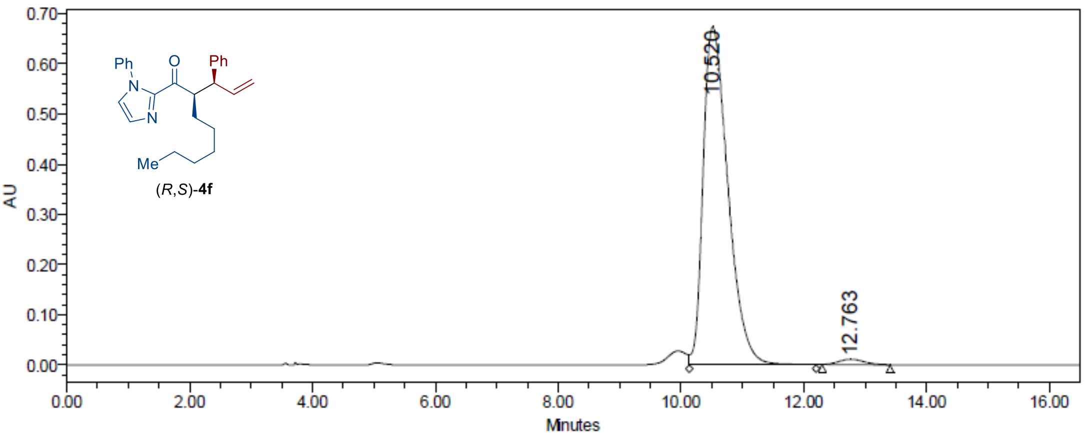

	RT	Area	% Area	Height
1	10.520	18795534	98.41	673046
2	12.763	304406	1.59	10692

Supplementary Figure 107. HPLC spectra of compound **4f**

rac-**4g**

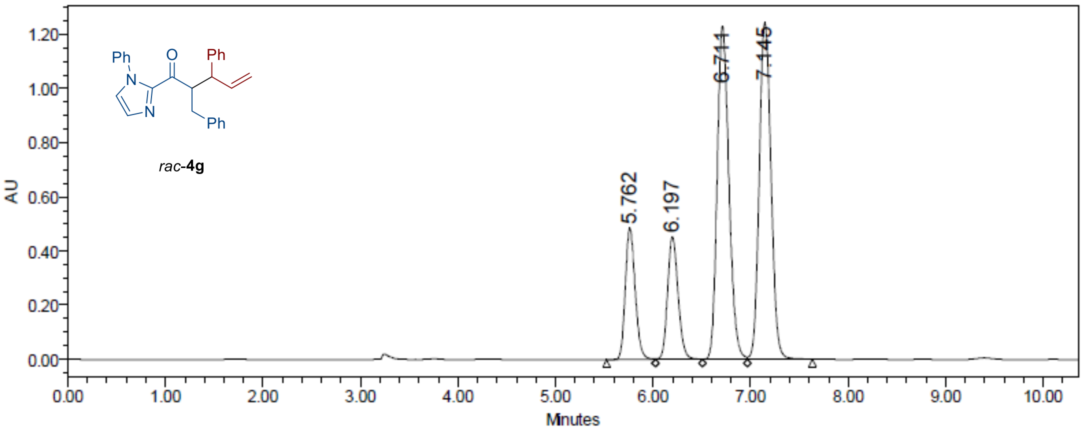

	RT	Area	% Area	Height
1	5.762	3387338	12.35	484618
2	6.197	3400644	12.40	453101
3	6.711	10221962	37.28	1228673
4	7.145	10410066	37.97	1241265

(*R*,*S*)-**4g**

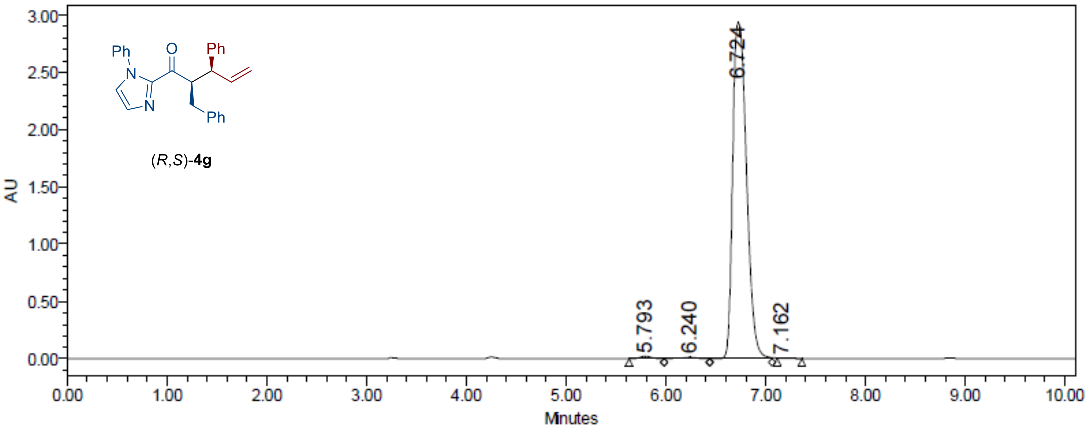

	RT	Area	% Area	Height
1	5.793	152531	0.54	21774
2	6.240	112850	0.40	11756
3	6.724	28073807	99.05	2934305
4	7.162	4079	0.01	-467

Supplementary Figure 108. HPLC spectra of compound **4g**

rac-**4h**

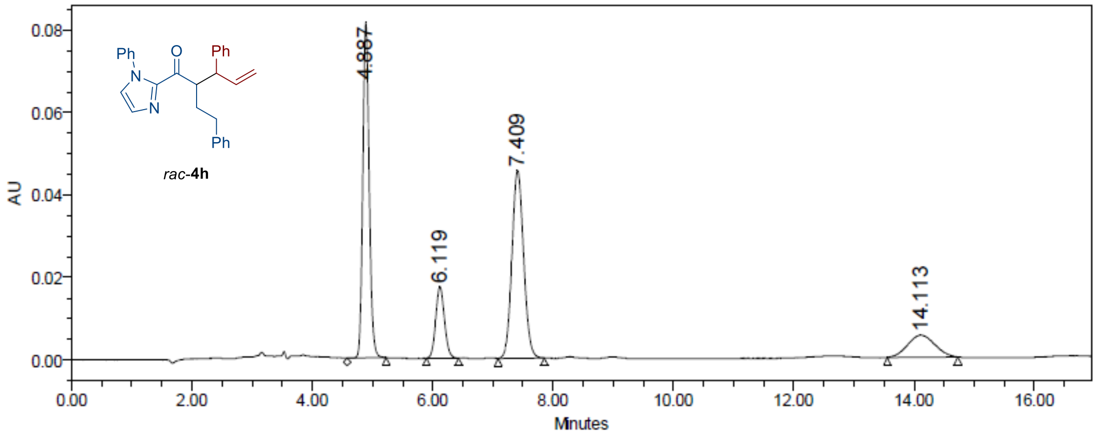

	RT	Area	% Area	Height
1	4.887	602875	38.59	81349
2	6.119	176113	11.27	17349
3	7.409	616831	39.48	45568
4	14.113	166534	10.66	5335

(*R*,*S*)-**4h**

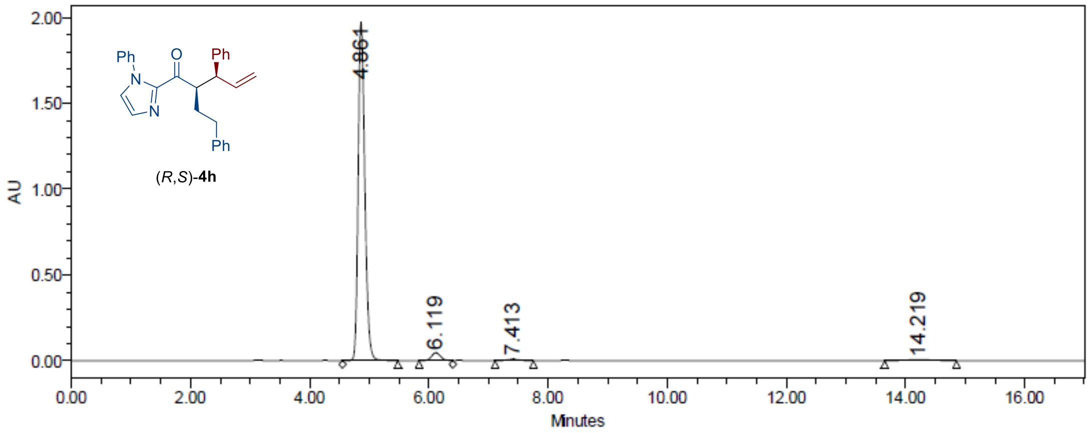

	RT	Area	% Area	Height
1	4.861	15361171	95.21	1969228
2	6.119	488034	3.02	46553
3	7.413	111660	0.69	8249
4	14.219	173584	1.08	5445

Supplementary Figure 109. HPLC spectra of compound **4h**

rac-**4i**

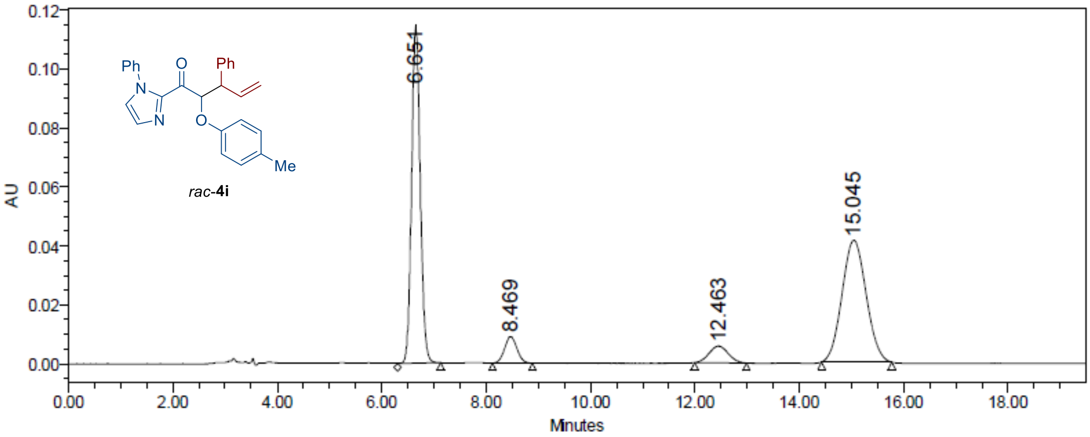

	RT	Area	% Area	Height
1	6.651	1313157	44.65	114476
2	8.469	148223	5.04	9035
3	12.463	144479	4.91	5655
4	15.045	1335382	45.40	41294

(*R*,*R*)-**4i**

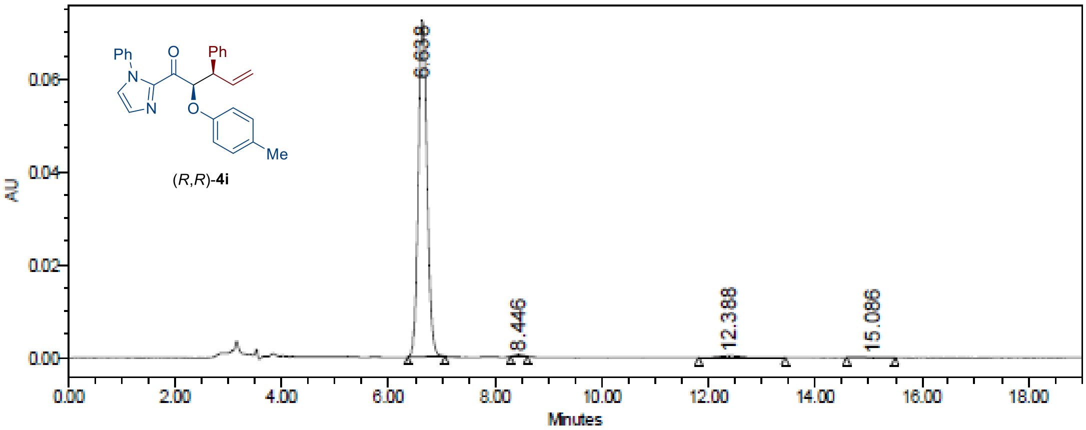

	RT	Area	% Area	Height
1	6.638	825359	98.36	72215
2	8.446	4285	0.51	372
3	12.388	7994	0.95	330
4	15.086	1504	0.18	72

Supplementary Figure 110. HPLC spectra of compound **4i**

rac-**4j**

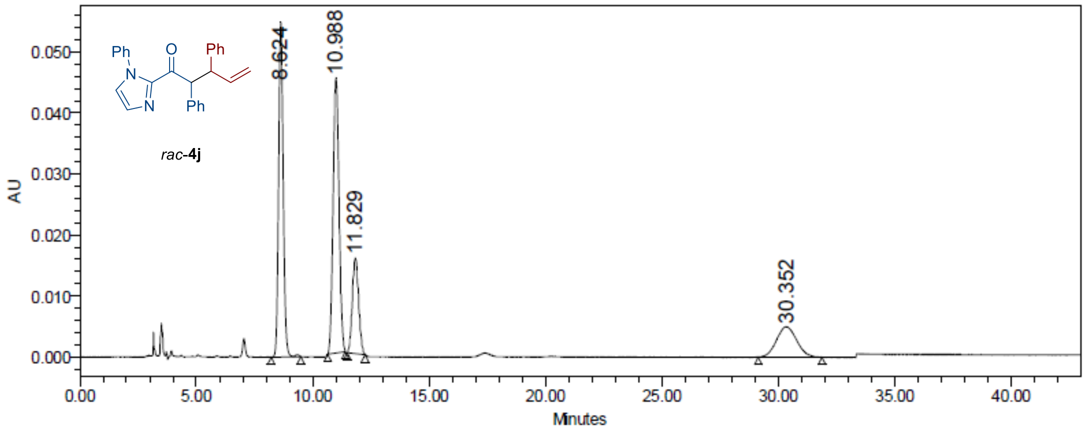

	RT	Area	% Area	Height
1	8.624	753529	35.06	54791
2	10.988	798109	37.13	45111
3	11.829	298585	13.89	15673
4	30.352	299109	13.92	5001

(*S*,*S*)-**4j**

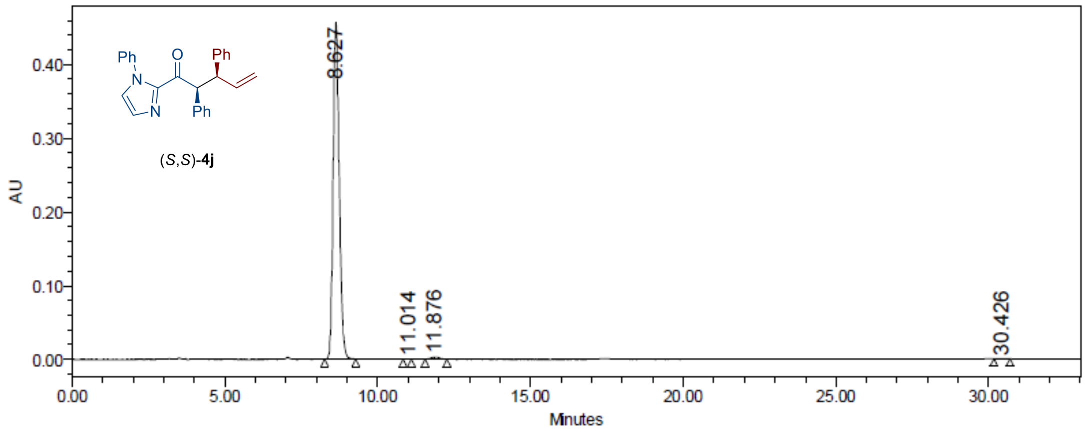

	RT	Area	% Area	Height
1	8.627	6248506	98.99	456134
2	11.014	826	0.01	116
3	11.876	59285	0.94	3129
4	30.426	3407	0.05	184

Supplementary Figure 111. HPLC spectra of compound **4j**

rac-**4k**

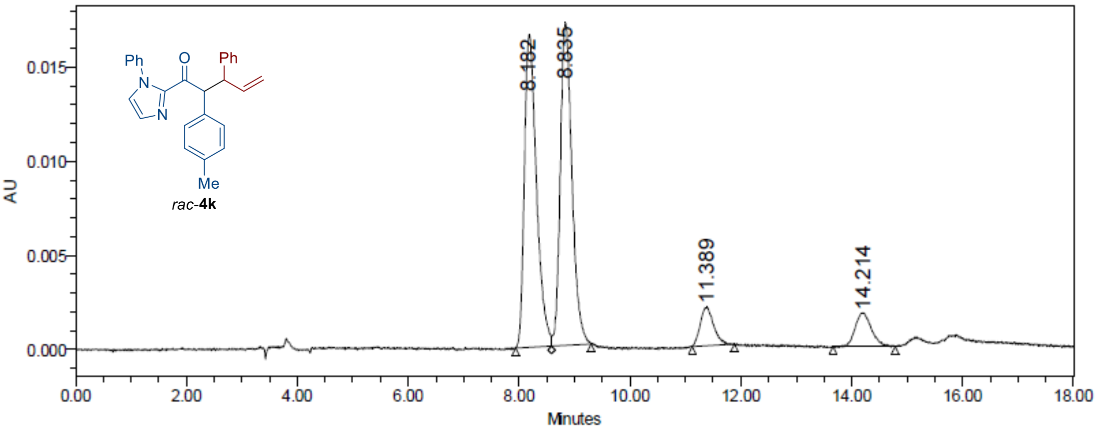

	RT	Area	% Area	Height
1	8.182	251250	43.77	16596
2	8.835	251740	43.85	17135
3	11.389	34472	6.01	2054
4	14.214	36577	6.37	1781

(*S*,*S*)-**4k**

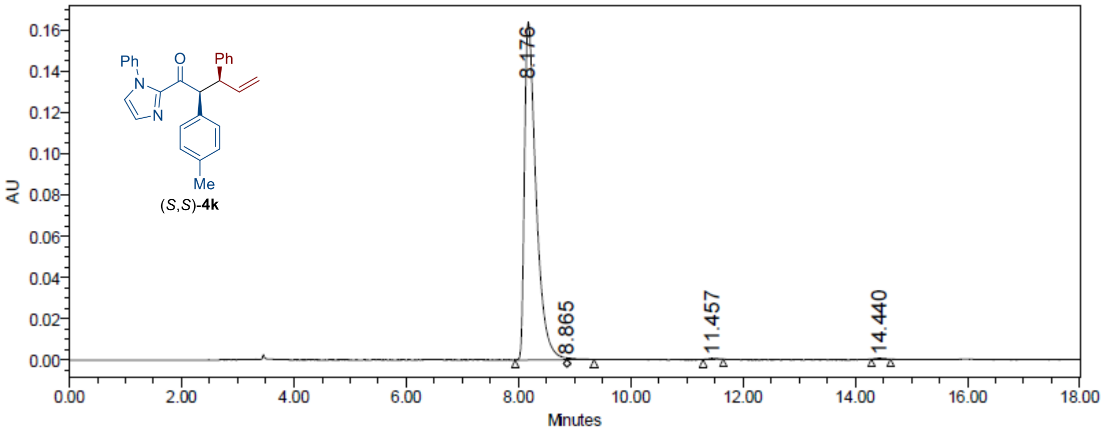

	RT	Area	% Area	Height
1	8.176	2355733	99.17	163452
2	8.865	8163	0.34	780
3	11.457	5727	0.24	482
4	14.440	5775	0.24	506

Supplementary Figure 112. HPLC spectra of compound **4k**

rac-**4l**

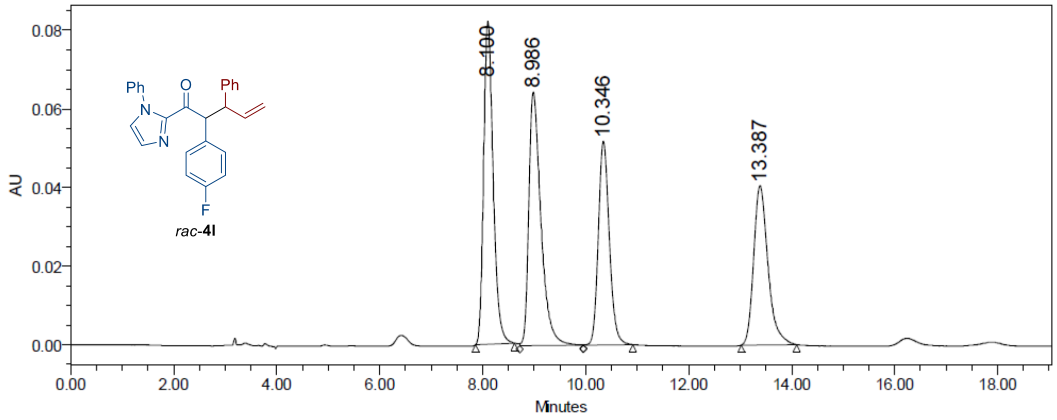

	RT	Area	% Area	Height
1	8.100	1048180	28.74	82075
2	8.986	1047301	28.72	64476
3	10.346	769169	21.09	51763
4	13.387	782259	21.45	40473

(*S*,*S*)-**4l**

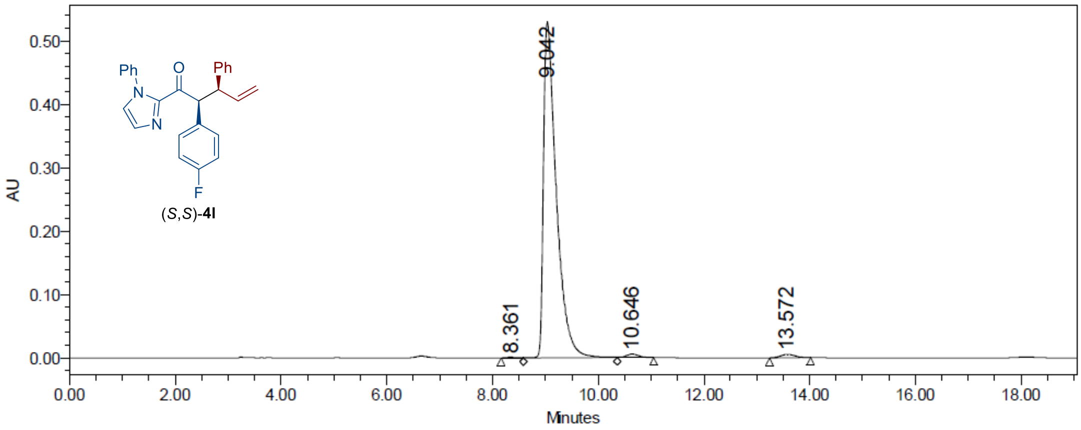

	RT	Area	% Area	Height
1	8.361	10821	0.12	868
2	9.042	8904956	97.80	529504
3	10.646	92839	1.02	5615
4	13.572	97105	1.07	5255

Supplementary Figure 113. HPLC spectra of compound **4l**

rac-**4m**

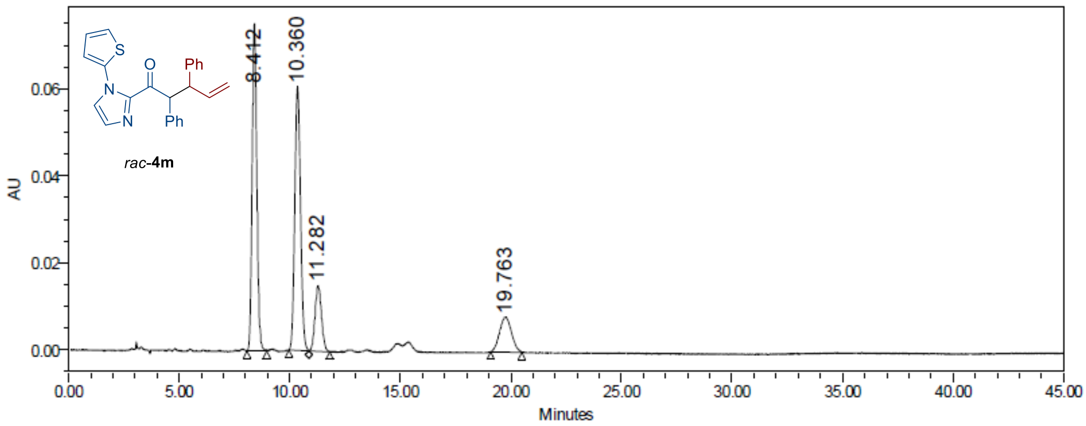

	RT	Area	% Area	Height
1	8.412	1100931	39.19	75250
2	10.360	1106604	39.39	60628
3	11.282	302327	10.76	15113
4	19.763	299618	10.66	8063

(*S*,*S*)-**4m**

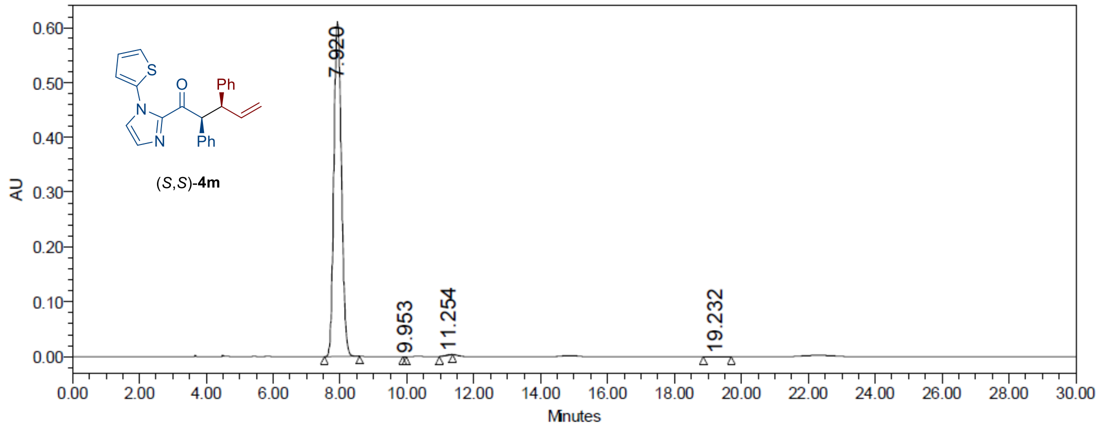

	RT	Area	% Area	Height
1	7.920	9493485	99.92	610033
2	9.953	140	0.00	64
3	11.254	6885	0.07	664
4	19.232	815	0.01	-54

Supplementary Figure 114. HPLC spectra of compound **4m**

rac-**4n**

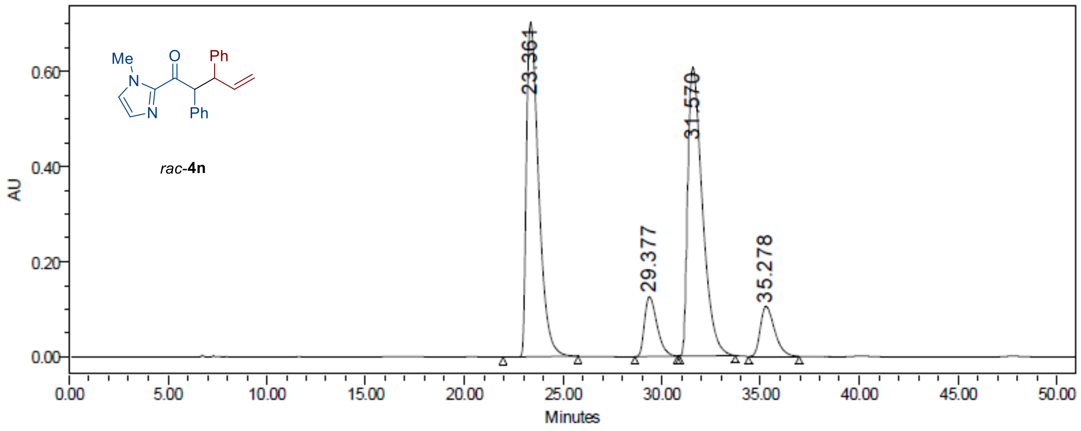

	RT	Area	% Area	Height
1	23.361	30409432	42.01	701493
2	29.377	5591079	7.72	125914
3	31.570	31076307	42.93	606424
4	35.278	5313233	7.34	105152

(*S*,*S*)-**4n**

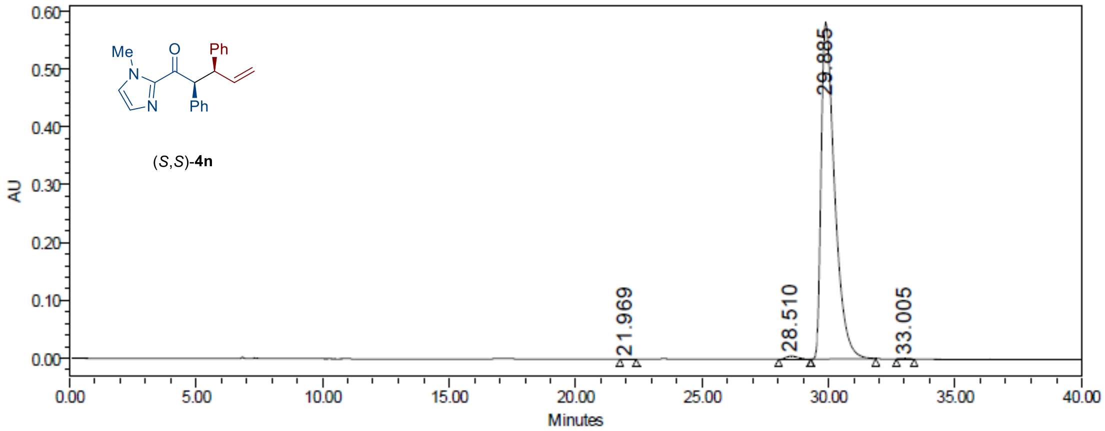

	RT	Area	% Area	Height
1	21.969	486	0.00	-50
2	28.510	179415	0.82	5532
3	29.885	21712512	99.08	581324
4	33.005	22050	0.10	890

Supplementary Figure 115. HPLC spectra of compound **4n**

rac-**4o**

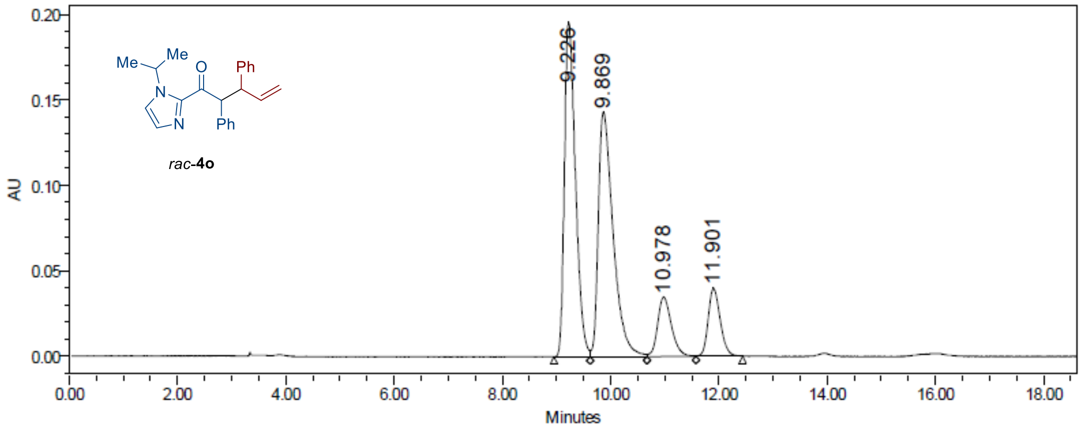

	RT	Area	% Area	Height
1	9.226	2744458	40.86	195596
2	9.869	2730504	40.65	143309
3	10.978	621271	9.25	34851
4	11.901	620249	9.23	39801

(*S*,*S*)-**4o**

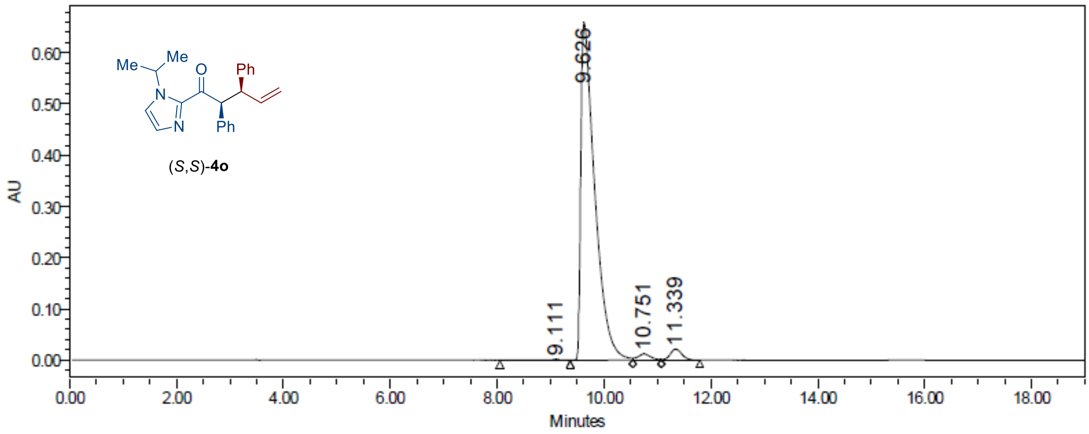

	RT	Area	% Area	Height
1	9.111	23951	0.19	1650
2	9.626	12006023	95.52	658505
3	10.751	207765	1.65	12002
4	11.339	331007	2.63	21668

Supplementary Figure 116. HPLC spectra of compound **4o**

rac-**4p**

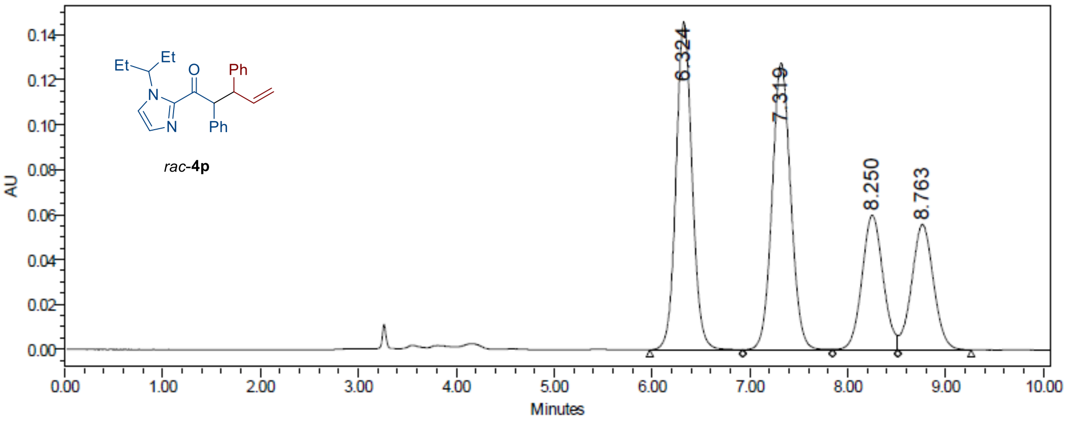

	RT	Area	% Area	Height
1	6.324	1663224	32.53	145577
2	7.319	1679950	32.86	127528
3	8.250	899915	17.60	59942
4	8.763	869474	17.01	55801

(*S*,*S*)-**4p**

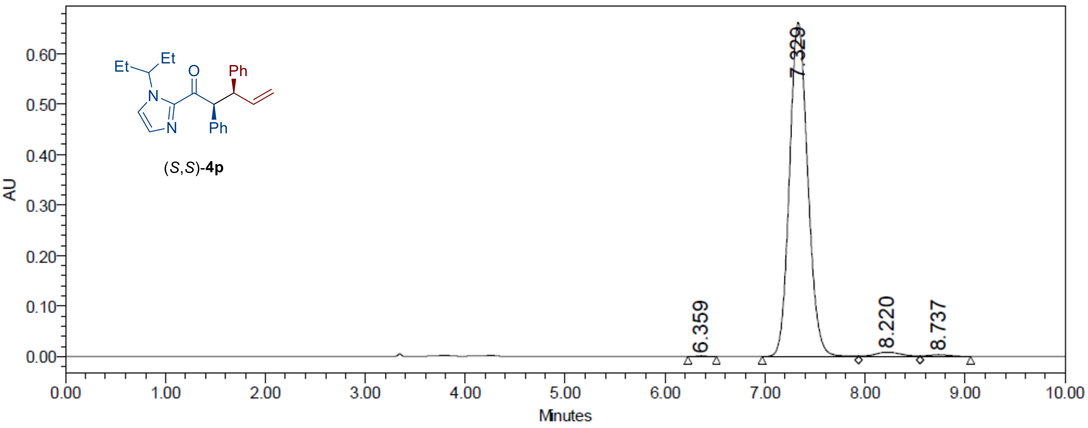

	RT	Area	% Area	Height
1	6.359	5889	0.07	643
2	7.329	8377784	97.71	661829
3	8.220	143116	1.67	8279
4	8.737	47048	0.55	3185

Supplementary Figure 117. HPLC spectra of compound **4p**

rac-**4q**

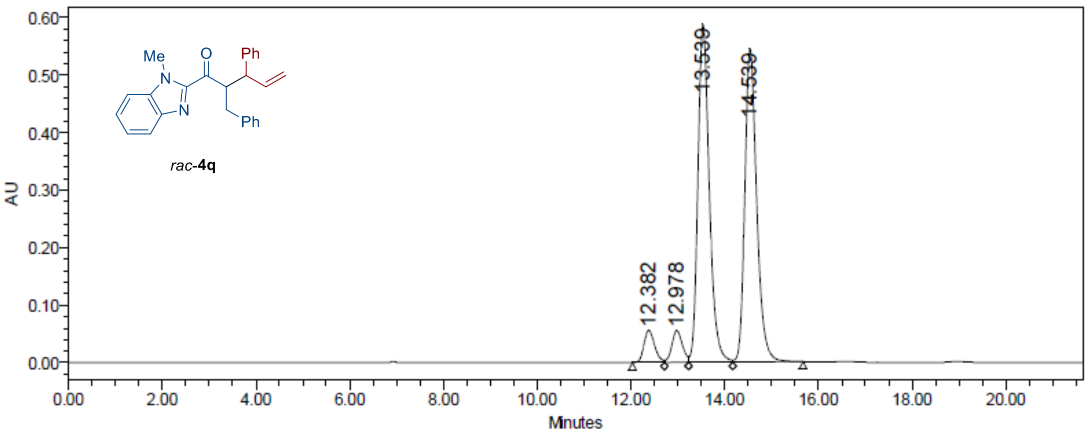

	RT	Area	% Area	Height
1	12.382	873181	4.10	56105
2	12.978	866229	4.07	55099
3	13.539	9922905	46.59	587282
4	14.539	9635253	45.24	544592

(*R*,*S*)-**4q**

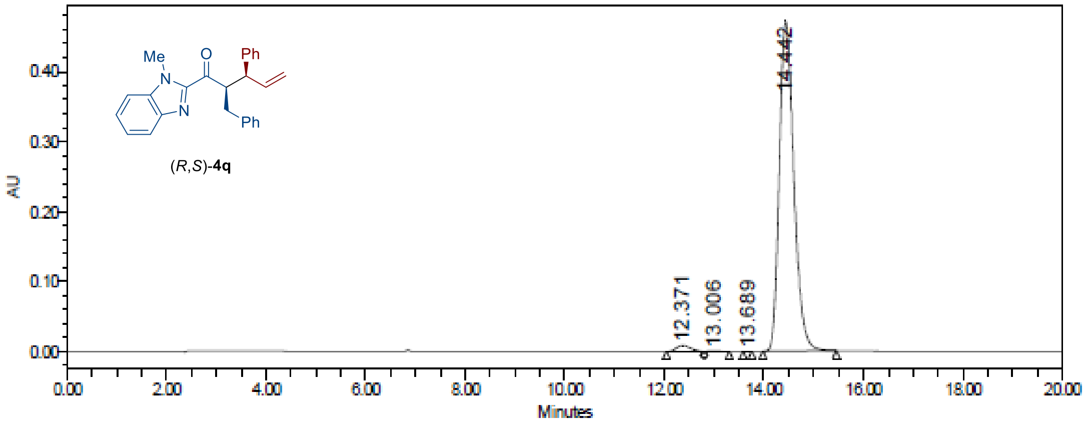

	RT	Area	% Area	Height
1	12.371	162355	1.67	8687
2	13.006	13079	0.13	737
3	13.689	90	0.00	31
4	14.442	9550040	98.20	471401

Supplementary Figure 118. HPLC spectra of compound **4q**

rac-**4r**

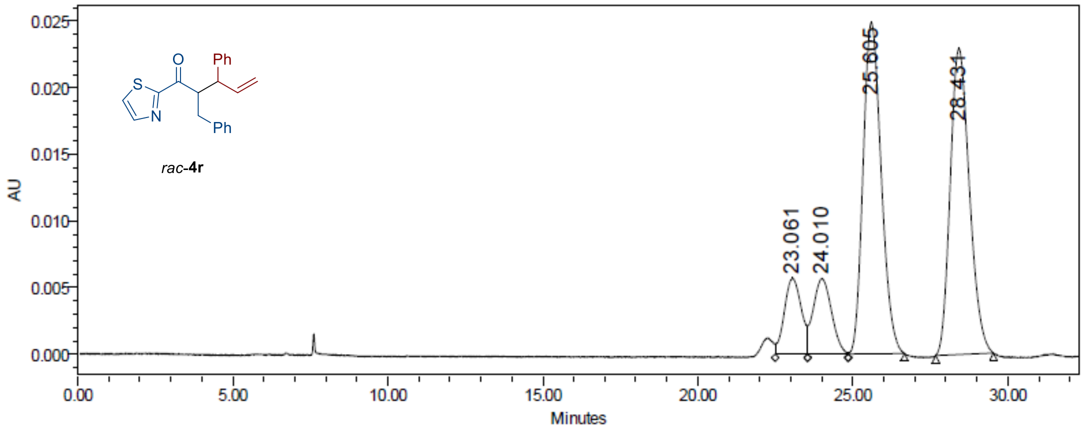

	RT	Area	% Area	Height
1	23.061	215449	8.82	5690
2	24.010	232380	9.52	5677
3	25.605	1008044	41.28	24852
4	28.431	986180	40.38	22988

(*R*,*S*)-**4r**

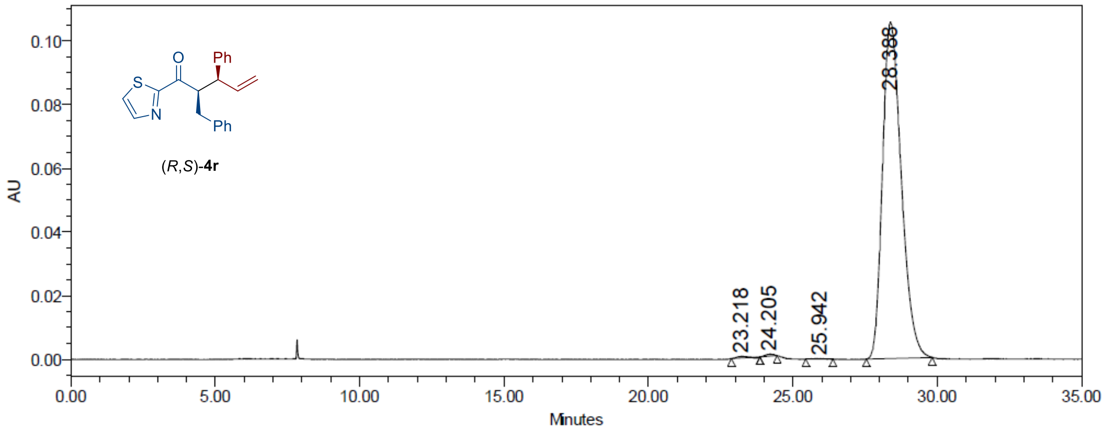

	RT	Area	% Area	Height
1	23.218	15998	0.34	588
2	24.205	14418	0.30	689
3	25.942	3895	0.08	158
4	28.388	4730311	99.28	105574

Supplementary Figure 119. HPLC spectra of compound **4r**

rac-**4s**

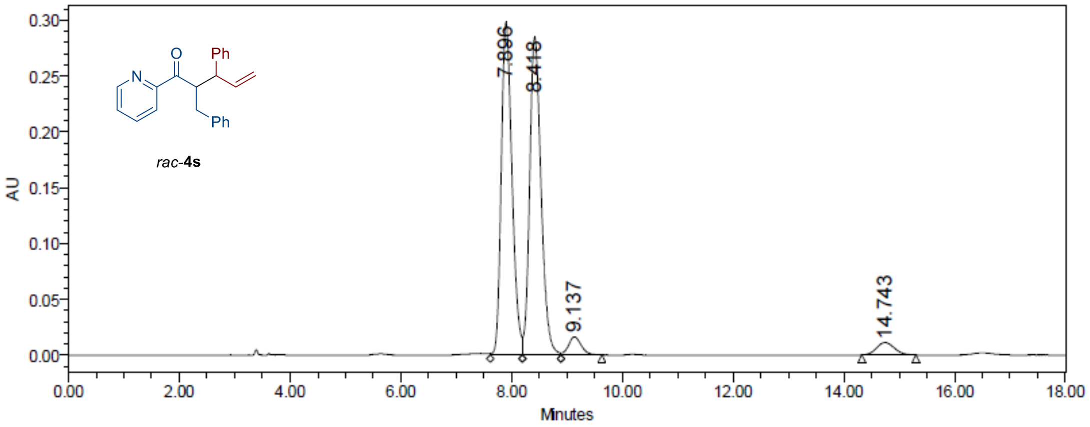

	RT	Area	% Area	Height
1	7.896	4029748	46.74	297552
2	8.418	4078703	47.31	284726
3	9.137	259500	3.01	16308
4	14.743	253433	2.94	11178

(*R*,*S*)-**4s**

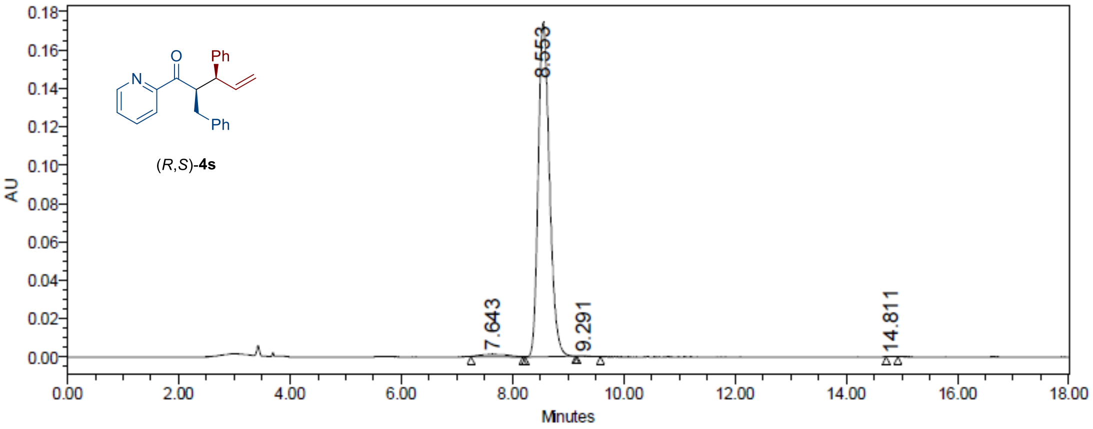

	RT	Area	% Area	Height
1	7.643	41077	1.65	1246
2	8.553	2442923	98.26	173970
3	9.291	1863	0.07	205
4	14.811	425	0.02	86

Supplementary Figure 120. HPLC spectra of compound **4s**

rac-**4t**

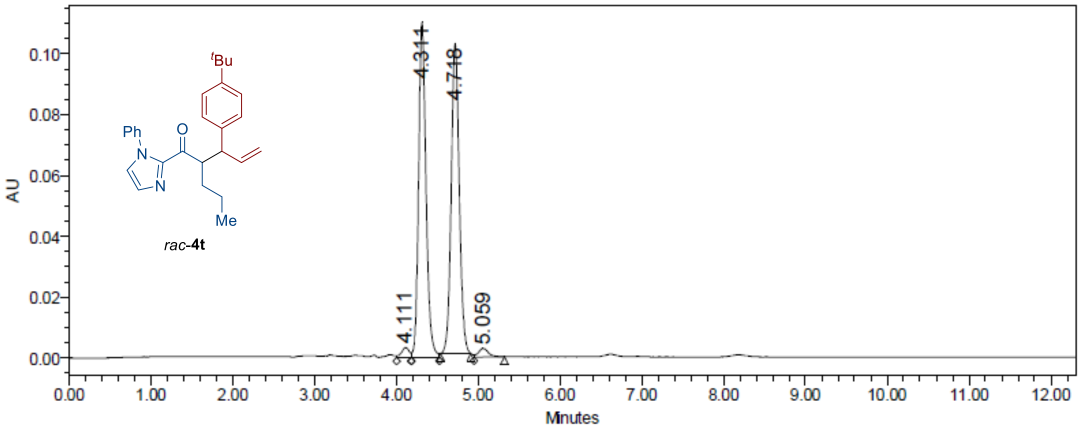

	RT	Area	% Area	Height
1	4.111	20697	1.45	3242
2	4.311	689137	48.18	109938
3	4.718	697531	48.77	101837
4	5.059	23012	1.61	2750

(*R*,*S*)-**4t**

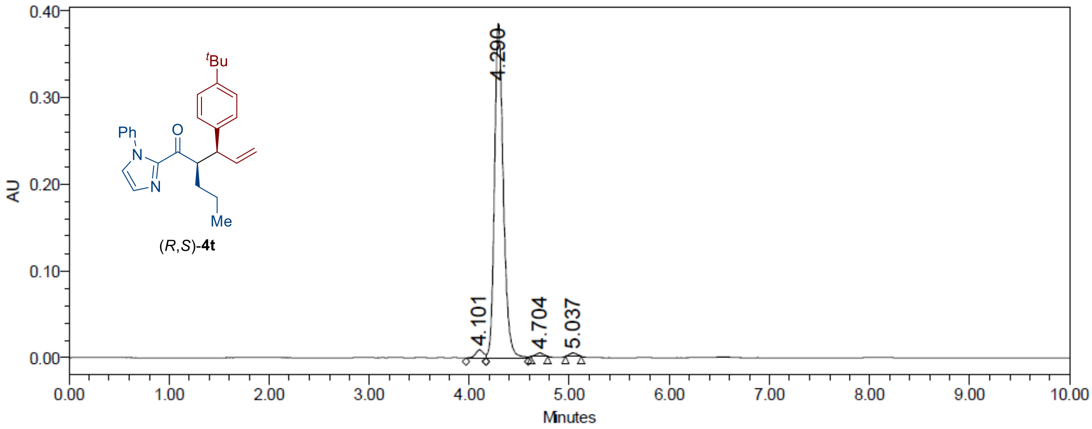

	RT	Area	% Area	Height
1	4.101	50593	2.07	9275
2	4.290	2350284	96.24	384923
3	4.704	20476	0.84	3878
4	5.037	20652	0.85	3823

Supplementary Figure 121. HPLC spectra of compound **4t**

rac-**4u**

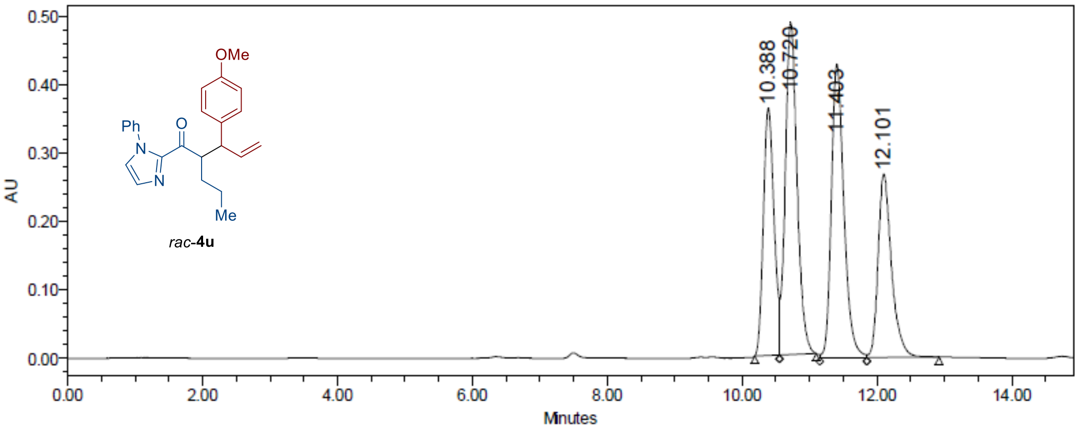

	RT	Area	% Area	Height
1	10.388	3965876	20.81	361464
2	10.720	5853125	30.72	484855
3	11.403	5553877	29.15	428455
4	12.101	3680302	19.32	267722

(*R*,*S*)-**4u**

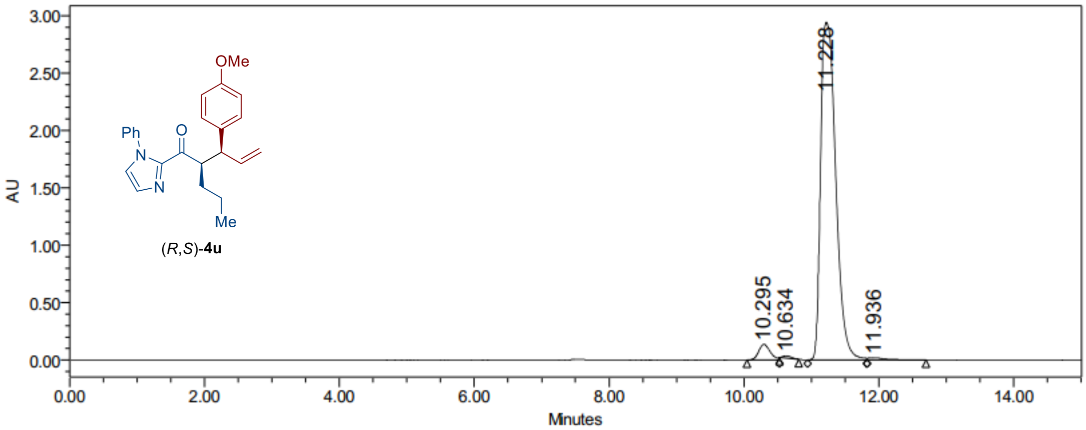

	RT	Area	% Area	Height
1	10.295	1593844	3.33	136859
2	10.634	192629	0.40	22142
3	11.228	45749664	95.62	2941385
4	11.936	309945	0.65	18518

Supplementary Figure 122. HPLC spectra of compound **4u**

rac-**4v**

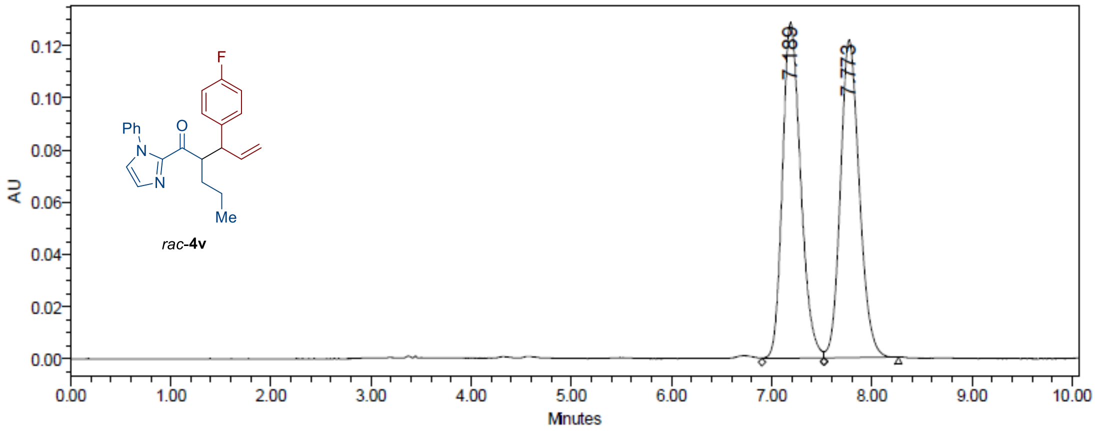

	RT	Area	% Area	Height
1	7.189	1617146	50.15	128530
2	7.773	1607717	49.85	121727

(*R*,*S*)-**4v**

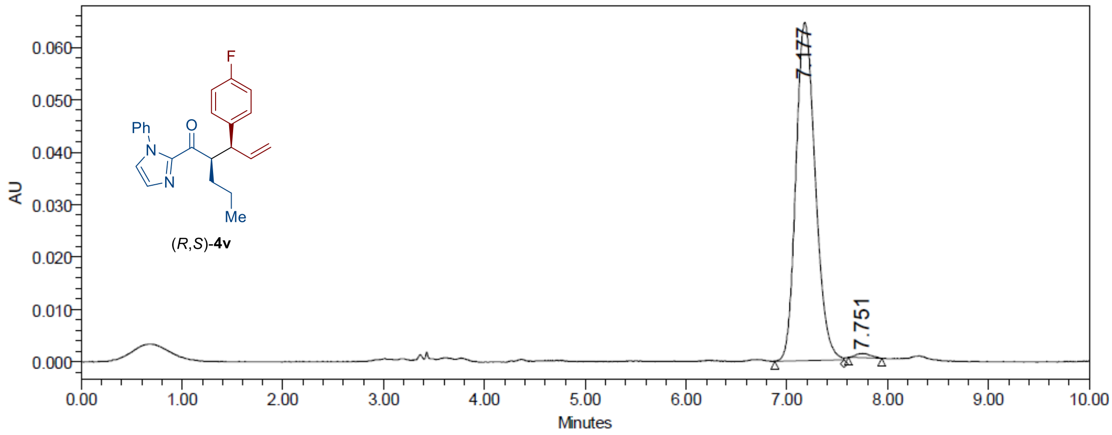

	RT	Area	% Area	Height
1	7.177	864171	99.04	64453
2	7.751	8406	0.96	827

Supplementary Figure 123. HPLC spectra of compound **4v**

rac-**4w**

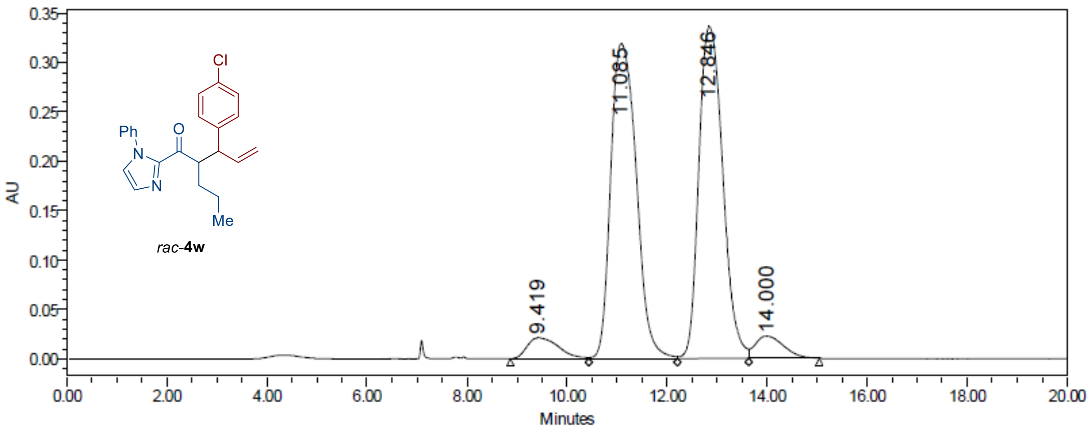

	RT	Area	% Area	Height
1	9.419	912068	3.76	20956
2	11.085	11235773	46.32	318650
3	12.846	11217430	46.25	335673
4	14.000	889396	3.67	22494

(*R*,*S*)-**4w**

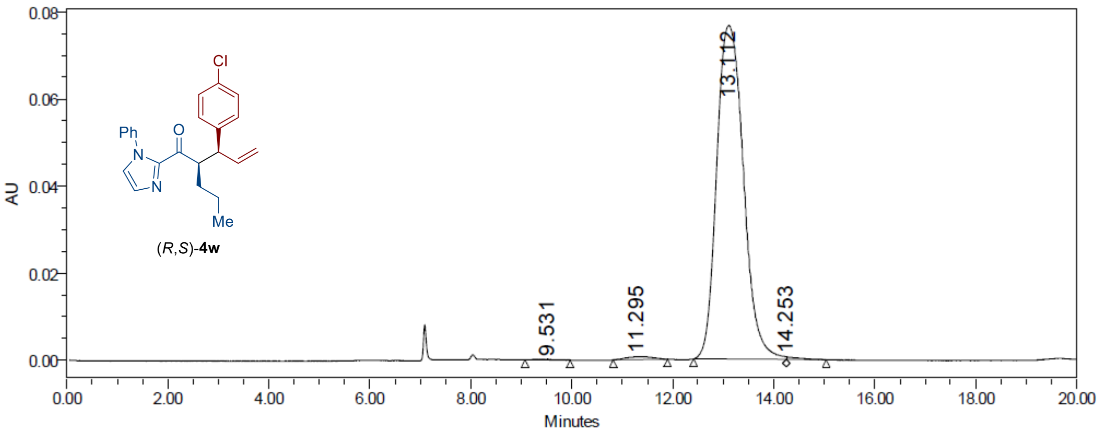

	RT	Area	% Area	Height
1	9.531	3755	0.13	143
2	11.295	24016	0.84	671
3	13.112	2821726	98.64	76595
4	14.253	11128	0.39	604

Supplementary Figure 124. HPLC spectra of compound **4w**

rac-**4x**

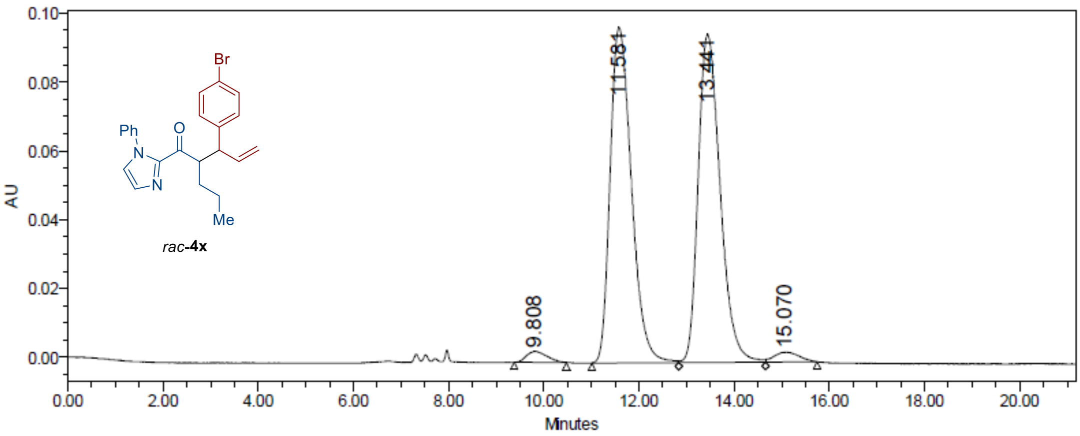

	RT	Area	% Area	Height
1	9.808	94405	1.50	3207
2	11.581	3031792	48.30	97374
3	13.441	3045672	48.52	95327
4	15.070	104719	1.67	2918

(*R*,*S*)-**4x**

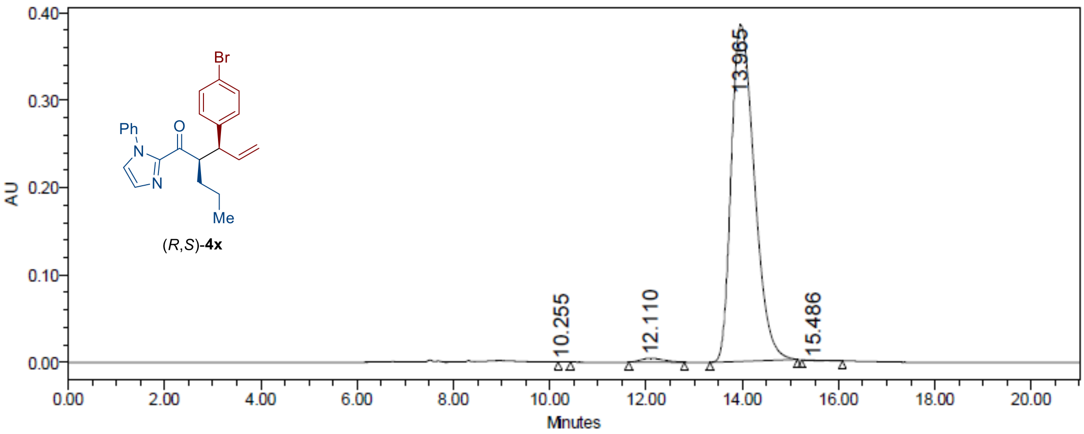

	RT	Area	% Area	Height
1	10.255	389	0.00	-71
2	12.110	147395	1.13	4483
3	13.965	12866307	98.77	384729
4	15.486	12214	0.09	-533

Supplementary Figure 125. HPLC spectra of compound **4x**

rac-**4y**

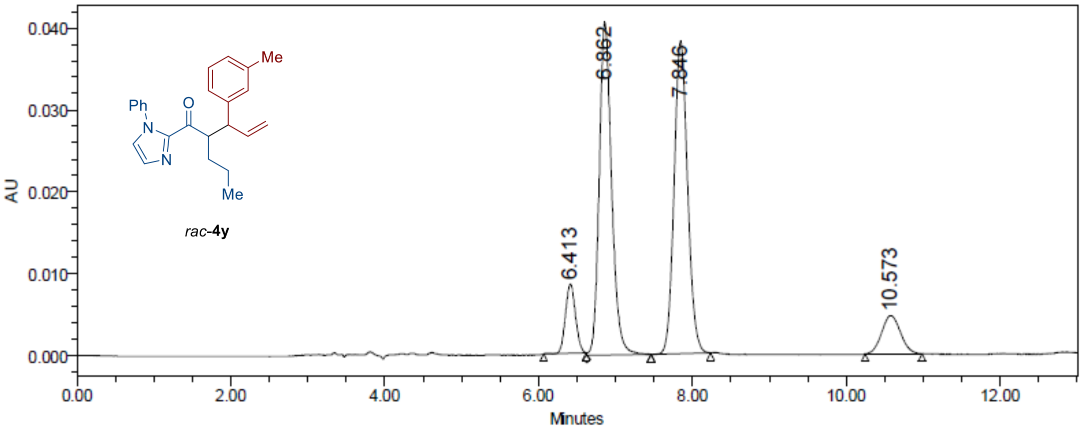

	RT	Area	% Area	Height
1	6.413	76712	7.12	8447
2	6.862	455631	42.30	40666
3	7.846	464532	43.12	38192
4	10.573	80328	7.46	4693

(*R*,*S*)-**4y**

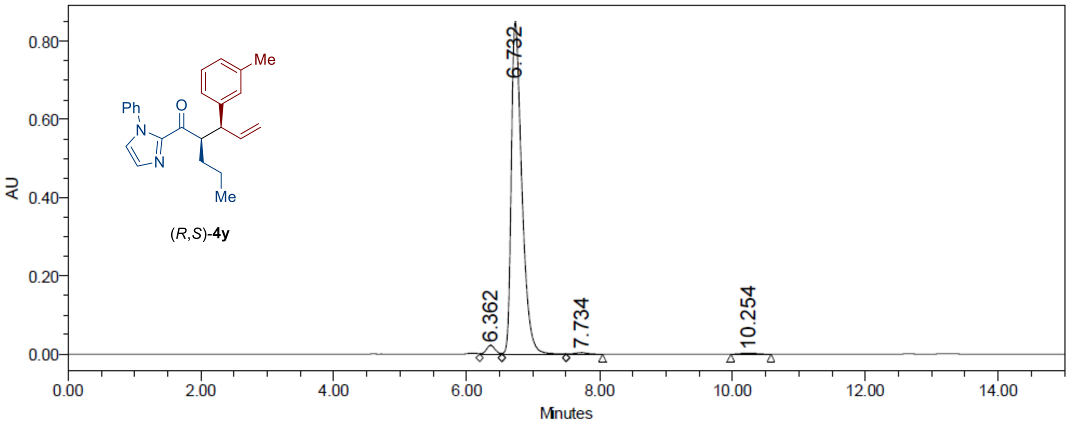

	RT	Area	% Area	Height
1	6.362	201084	2.07	22258
2	6.732	9399545	96.99	849302
3	7.734	47959	0.49	3679
4	10.254	42744	0.44	2707

Supplementary Figure 126. HPLC spectra of compound **4y**

rac-**4z**

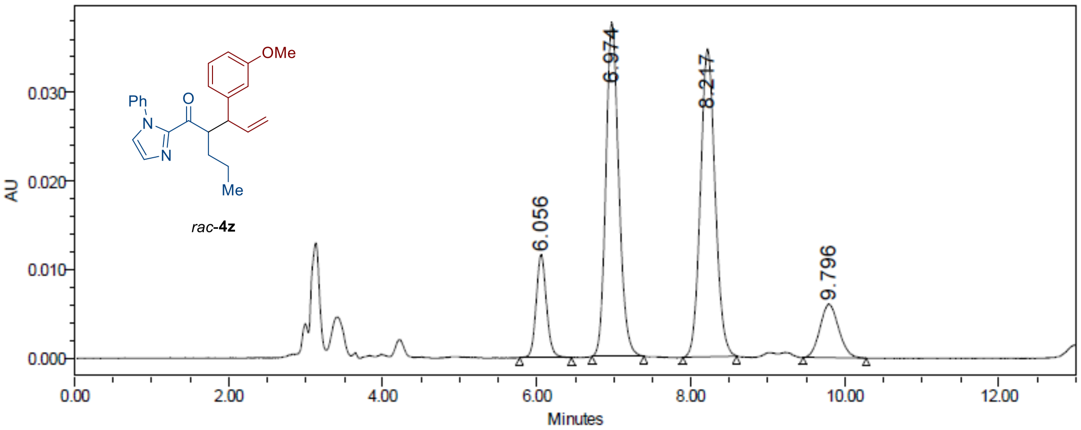

	RT	Area	% Area	Height
1	6.056	108597	9.78	11607
2	6.974	439204	39.55	37606
3	8.217	460967	41.51	34716
4	9.796	101842	9.17	6008

(*R*,*S*)-**4z**

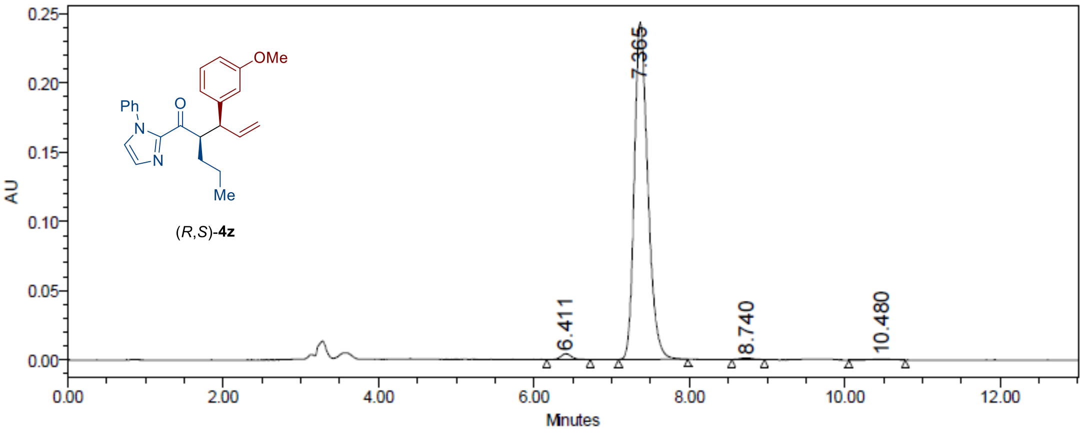

	RT	Area	% Area	Height
1	6.411	39516	1.32	4220
2	7.365	2924415	97.83	242961
3	8.740	14737	0.49	1184
4	10.480	10470	0.35	625

Supplementary Figure 127. HPLC spectra of compound **4z**

rac-**4aa**

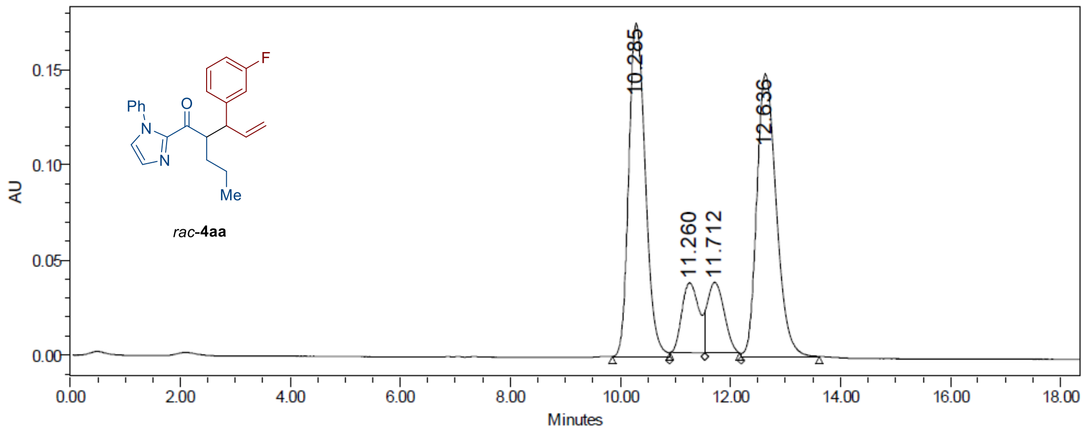

	RT	Area	% Area	Height
1	10.285	3646446	41.13	174971
2	11.260	797536	9.00	36636
3	11.712	780473	8.80	36933
4	12.636	3640556	41.07	148338

(*R*,*S*)-**4aa**

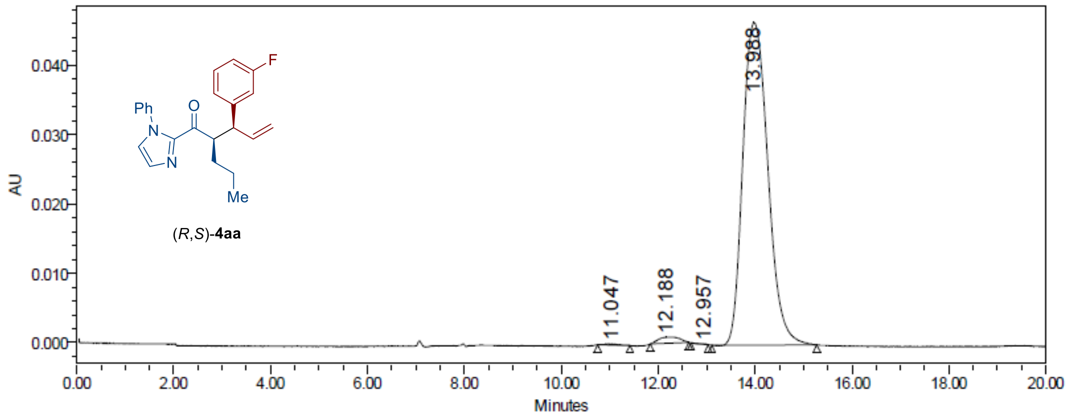

	RT	Area	% Area	Height
1	11.047	2902	0.17	151
2	12.188	27473	1.62	910
3	12.957	426	0.03	52
4	13.988	1669332	98.19	46474

Supplementary Figure 128. HPLC spectra of compound **4aa**

rac-**4ab**

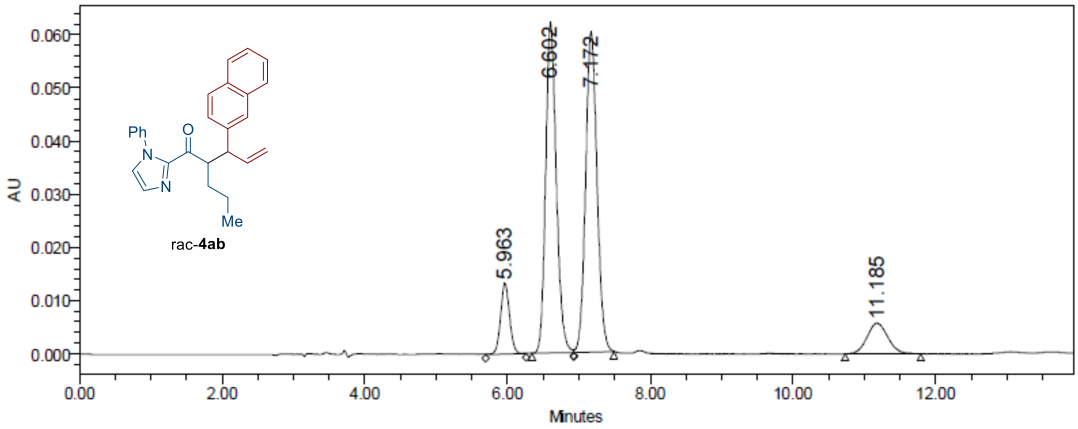

	RT	Area	% Area	Height
1	5.963	116803	7.54	13281
2	6.602	646340	41.73	62084
3	7.172	669039	43.20	60143
4	11.185	116525	7.52	5763

(*R*,*S*)-**4ab**

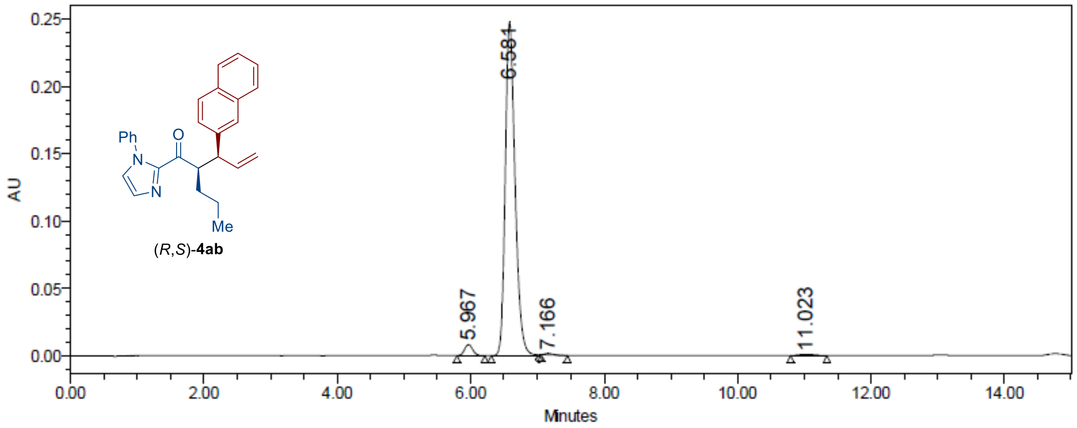

	RT	Area	% Area	Height
1	5.967	69542	2.65	8305
2	6.581	2534105	96.43	247241
3	7.166	9129	0.35	1049
4	11.023	15151	0.58	906

Supplementary Figure 129. HPLC spectra of compound **4ab**

rac-**4ac**

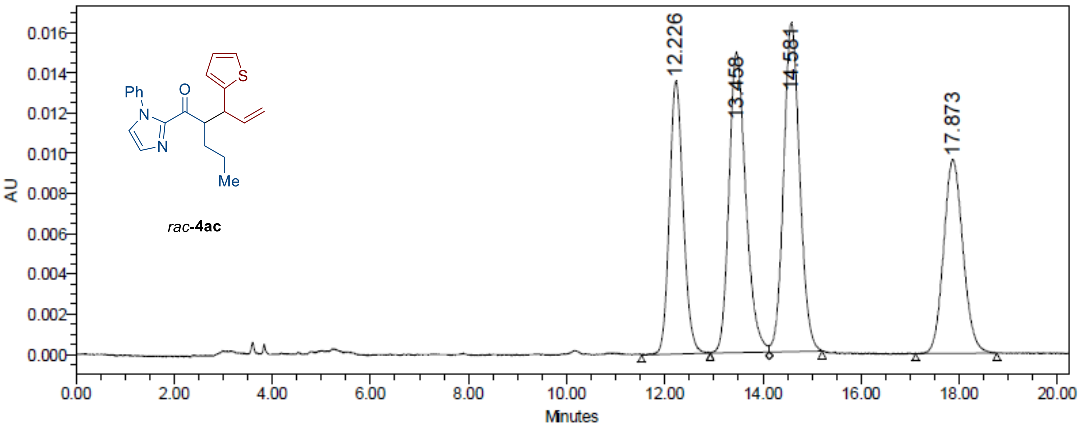

	RT	Area	% Area	Height
1	12.226	264985	21.06	13569
2	13.458	355991	28.29	14961
3	14.581	372243	29.58	16341
4	17.873	265296	21.08	9634

(*R*,*S*)-**4ac**

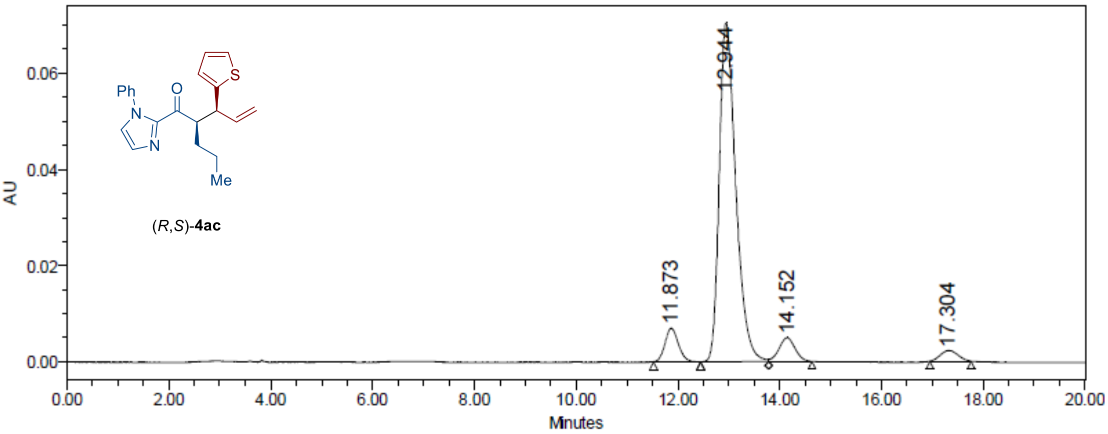

	RT	Area	% Area	Height
1	11.873	128807	6.88	6998
2	12.944	1579123	84.40	70452
3	14.152	109477	5.85	4955
4	17.304	53620	2.87	2251

Supplementary Figure 130. HPLC spectra of compound **4ac**

rac-**4ad**

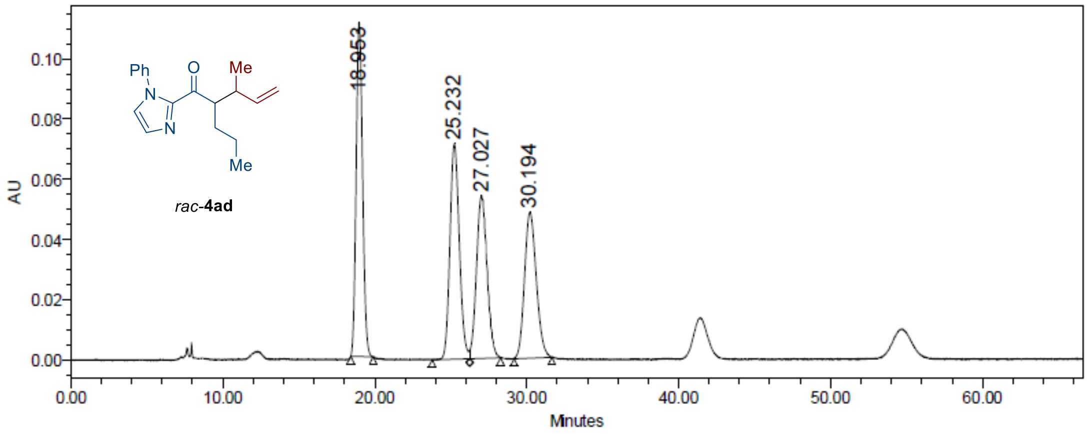

	RT	Area	% Area	Height
1	18.953	3282275	28.26	110867
2	25.232	3092370	26.62	71474
3	27.027	2601536	22.40	54157
4	30.194	2638481	22.72	48521

(*R*,*S*)-**4ad**

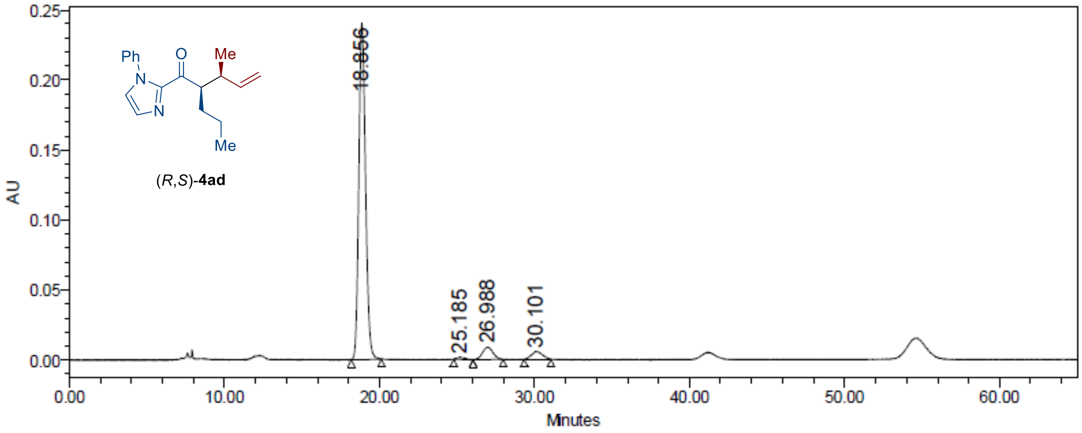

	RT	Area	% Area	Height
1	18.856	7245780	90.58	239765
2	25.185	49853	0.62	1422
3	26.988	431049	5.39	9064
4	30.101	272837	3.41	5573

Supplementary Figure 131. HPLC spectra of compound **4ad**

rac-**4ae**

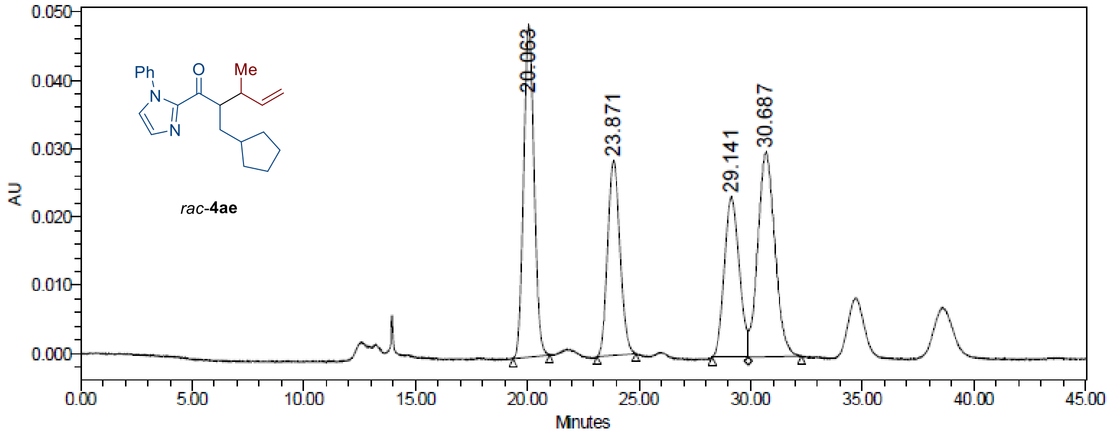

	RT	Area	% Area	Height
1	20.063	1586216	29.45	48737
2	23.871	1097684	20.38	28564
3	29.141	1092887	20.29	23459
4	30.687	1609522	29.88	29918

(*R*,*S*)-**4ae**

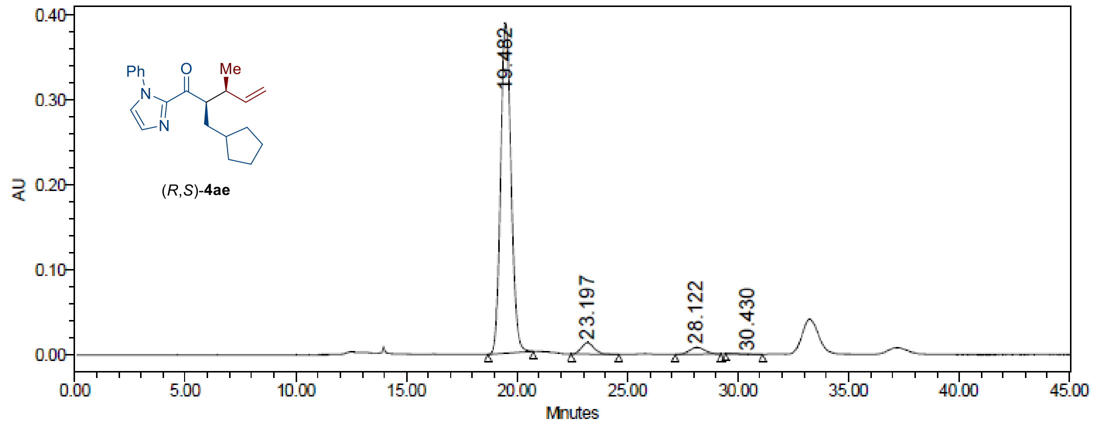

	RT	Area	% Area	Height
1	19.482	12808933	93.18	388280
2	23.197	536415	3.90	13523
3	28.122	373652	2.72	8164
4	30.430	27739	0.20	-611

Supplementary Figure 132. HPLC spectra of compound **4ae**

rac-**4af**

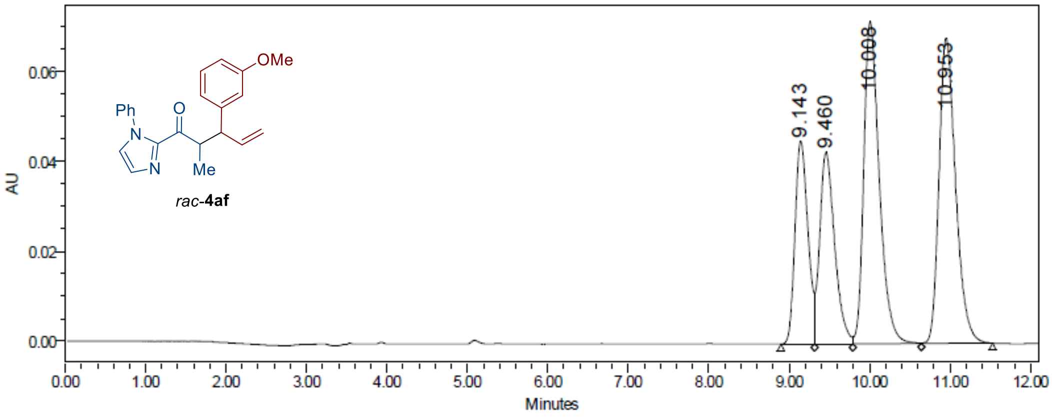

	RT	Area	% Area	Height
1	9.143	504378	16.88	45139
2	9.460	551255	18.45	42619
3	10.008	963223	32.24	71559
4	10.953	968509	32.42	67895

(*R*,*S*)-**4af**

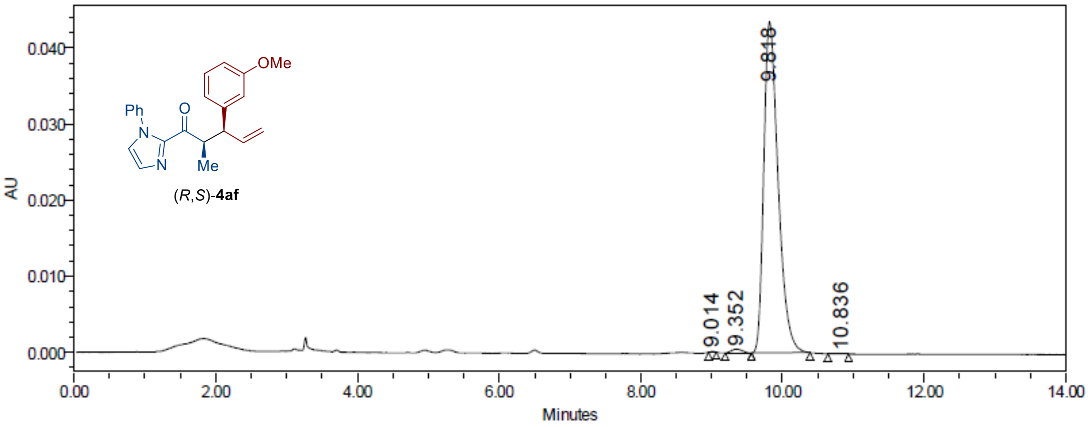

	RT	Area	% Area	Height
1	9.014	244	0.04	68
2	9.352	5593	0.90	521
3	9.818	612385	99.02	43480
4	10.836	232	0.04	37

(*R*,*R*)-**4af**

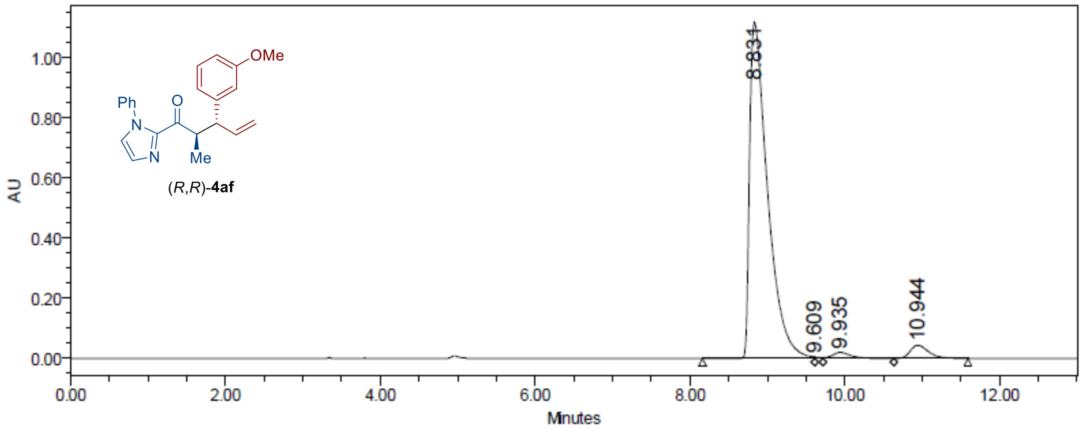

	RT	Area	% Area	Height
1	8.831	18089622	94.38	1117375
2	9.609	22989	0.12	4850
3	9.935	332901	1.74	20027
4	10.944	722279	3.77	44048

(*S*,*R*)-**4af**

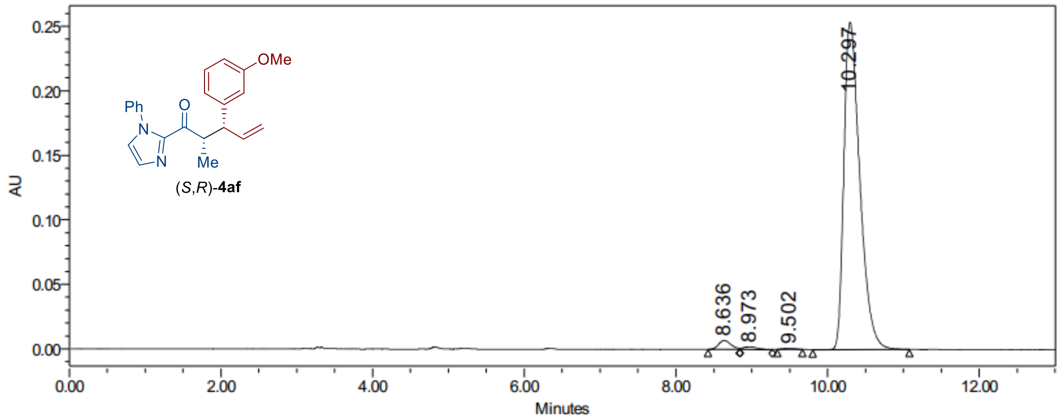

	RT	Area	% Area	Height
1	8.636	80900	2.14	6989
2	8.973	26801	0.71	2083
3	9.502	8910	0.24	854
4	10.297	3671696	96.92	253654

(*S*,*S*)-**4af**

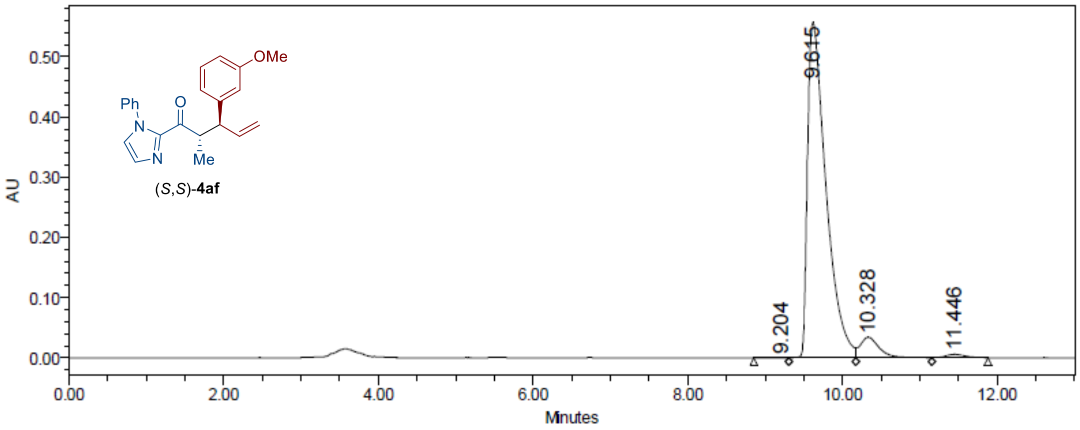

	RT	Area	% Area	Height
1	9.204	746	0.01	-92
2	9.615	9201831	93.08	556309
3	10.328	587022	5.94	34336
4	11.446	96816	0.98	5890

Supplementary Figure 133. HPLC spectra of compound **4af**

rac-**7**

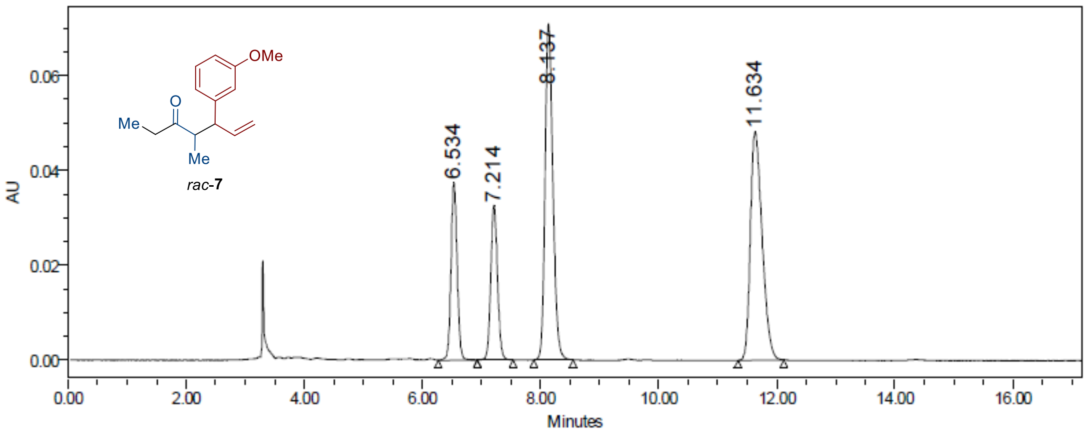

	RT	Area	% Area	Height
1	6.534	268183	14.52	37466
2	7.214	259173	14.03	32576
3	8.137	657545	35.60	70910
4	11.634	662259	35.85	48149

(*R*,*S*)-**7**

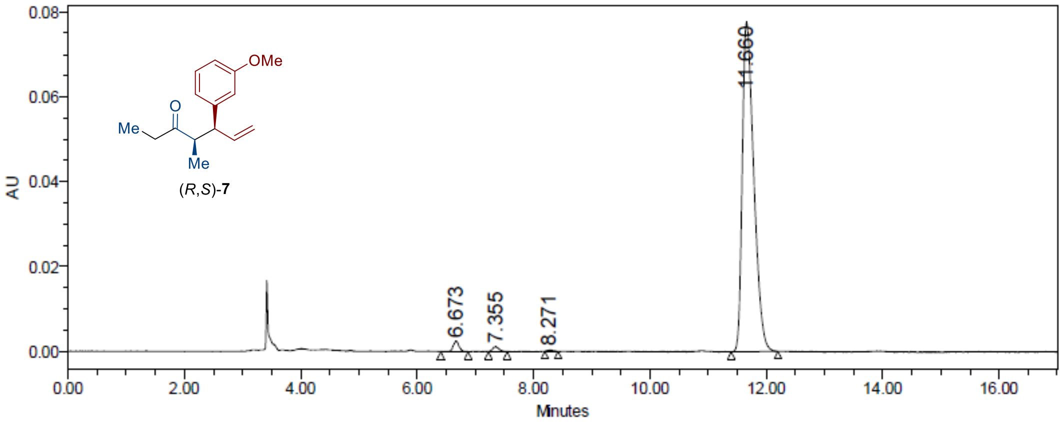

	RT	Area	% Area	Height
1	6.673	18344	1.66	2488
2	7.355	9630	0.87	1178
3	8.271	2444	0.22	359
4	11.660	1074686	97.25	77745

Supplementary Figure 134. HPLC spectra of compound **7**

rac-**8**

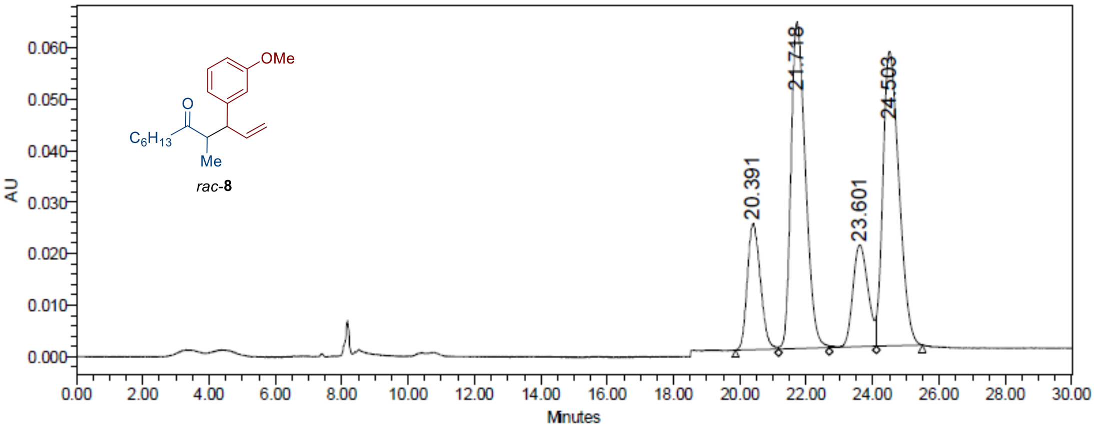

	RT	Area	% Area	Height
1	20.391	671682	13.04	24521
2	21.718	1921483	37.30	63358
3	23.601	635992	12.34	19856
4	24.503	1922697	37.32	57279

(*S*,*R*)-**8**

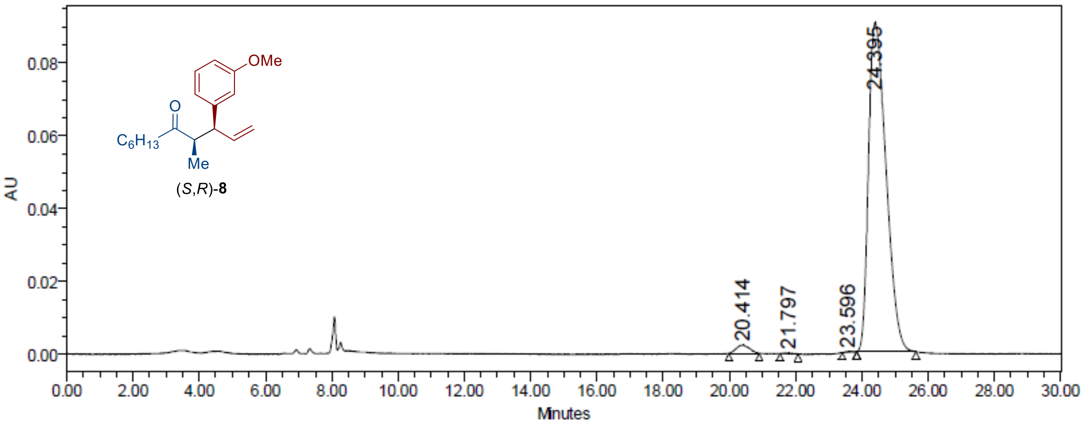

	RT	Area	% Area	Height
1	20.414	63808	1.90	2304
2	21.797	2755	0.08	221
3	23.596	2561	0.08	240
4	24.395	3282696	97.94	90497

Supplementary Figure 135. HPLC spectra of compound **8**

rac-**9**

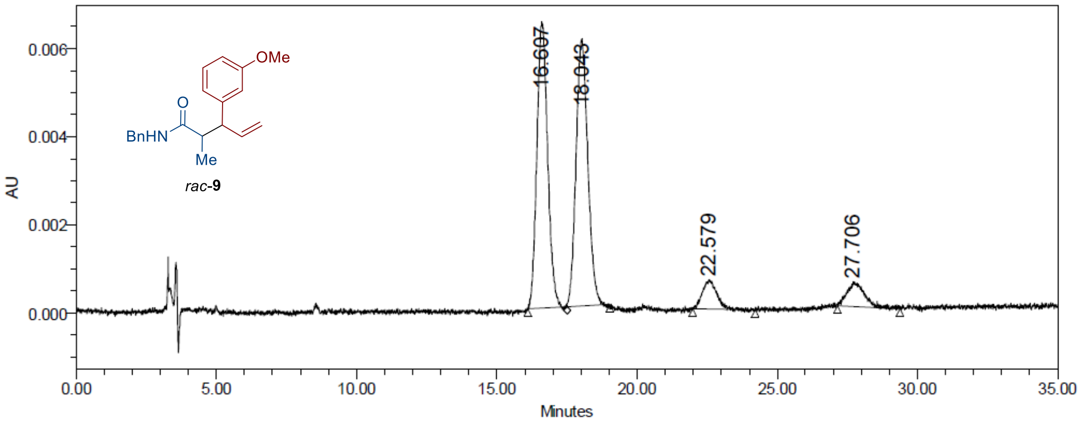

	RT	Area	% Area	Height
1	16.607	174299	43.60	6491
2	18.043	177174	44.32	6060
3	22.579	24142	6.04	663
4	27.706	24165	6.04	555

(*R*,*S*)-**9**

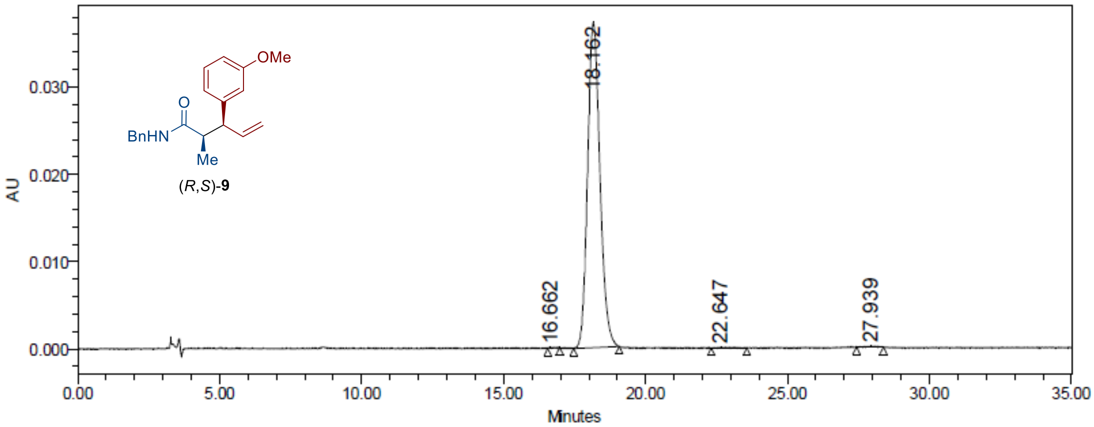

	RT	Area	% Area	Height
1	16.662	696	0.06	71
2	18.162	1124924	99.62	37232
3	22.647	2003	0.18	91
4	27.939	1581	0.14	107

Supplementary Figure 136. HPLC spectra of compound **9**

rac-**10**

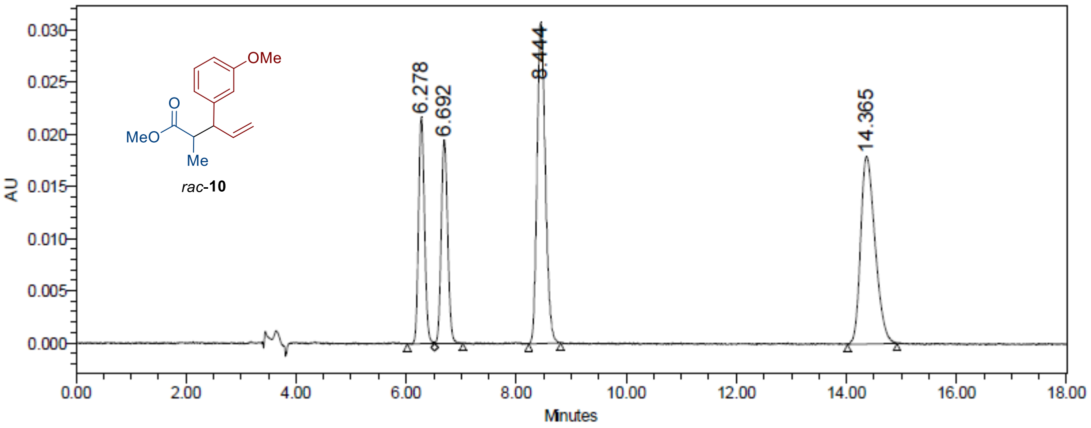

	RT	Area	% Area	Height
1	6.278	163141	16.79	21703
2	6.692	158270	16.29	19489
3	8.444	323134	33.26	30731
4	14.365	326958	33.65	17987

(*R*,*S*)-**10**

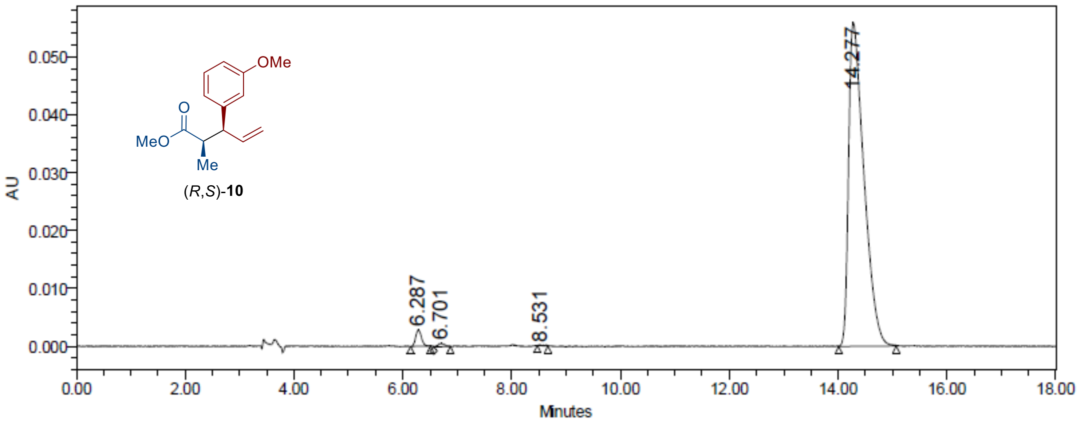

	RT	Area	% Area	Height
1	6.287	20751	1.86	2905
2	6.701	4953	0.44	581
3	8.531	596	0.05	112
4	14.277	1090413	97.64	55822

Supplementary Figure 137. HPLC spectra of compound **10**

rac-**11**

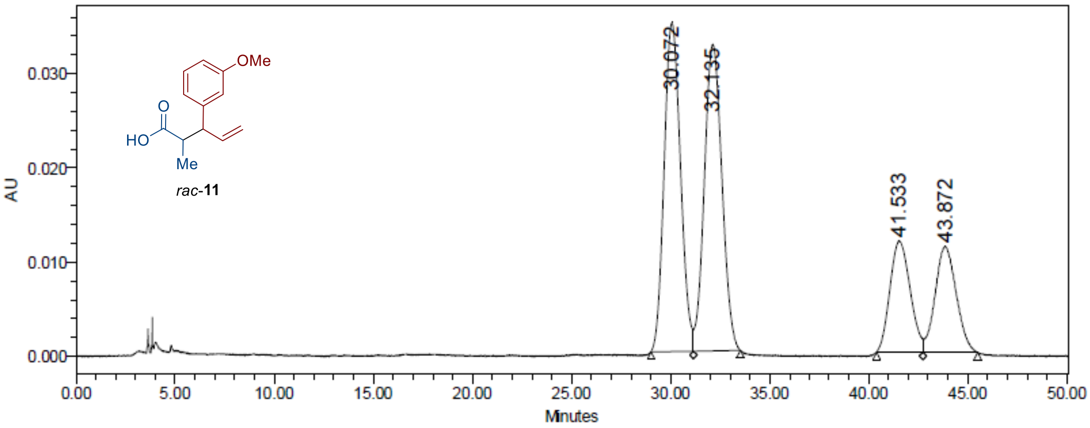

	RT	Area	% Area	Height
1	30.072	1996077	35.20	34922
2	32.135	2001150	35.29	32518
3	41.533	834007	14.71	11821
4	43.872	838807	14.79	11265

(*R*,*S*)-**11**

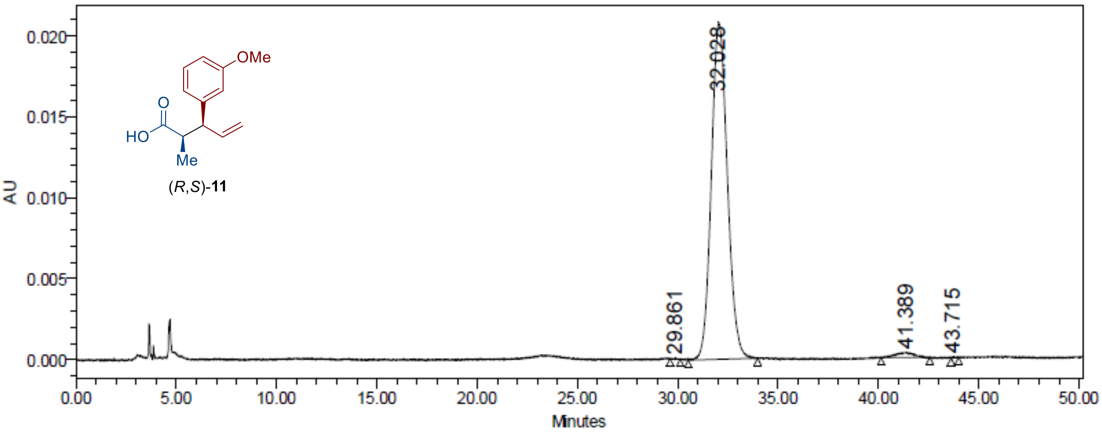

	RT	Area	% Area	Height
1	29.861	459	0.04	63
2	32.028	1151803	98.30	20785
3	41.389	18961	1.62	317
4	43.715	510	0.04	85

Supplementary Figure 138. HPLC spectra of compound **11**

rac-**12**

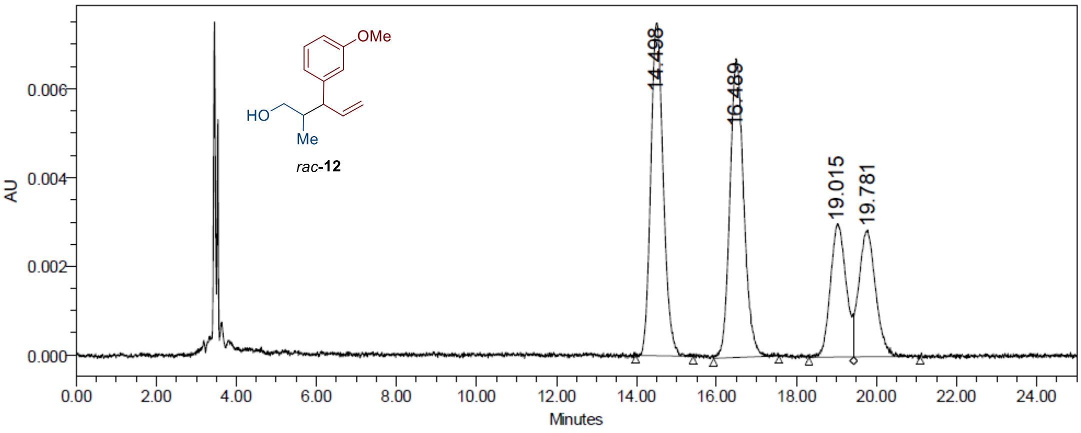

	RT	Area	% Area	Height
1	14.498	161684	33.09	7484
2	16.489	161704	33.09	6721
3	19.015	82097	16.80	2995
4	19.781	83128	17.01	2853

(*R*,*S*)-**12**

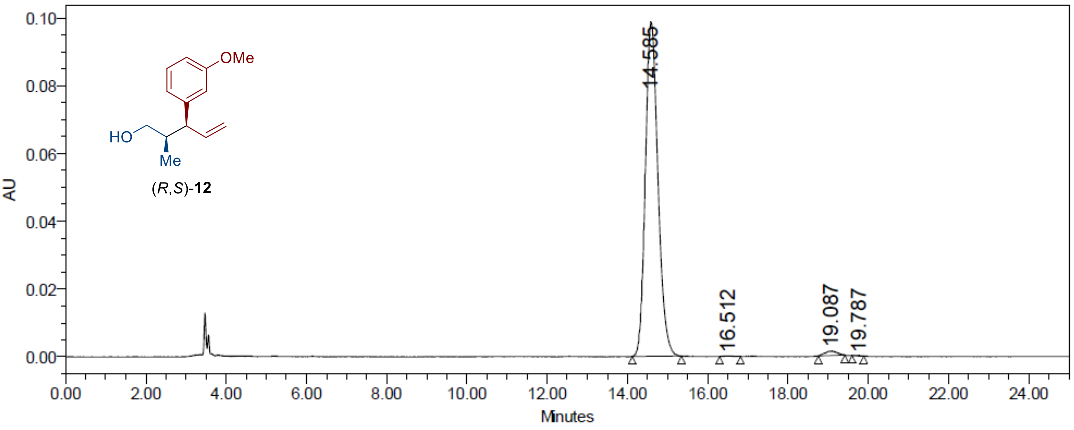

	RT	Area	% Area	Height
1	14.585	2154475	98.60	98694
2	16.512	2046	0.09	161
3	19.087	28206	1.29	1325
4	19.787	288	0.01	54

Supplementary Figure 139. HPLC spectra of compound **12**

rac-**13**

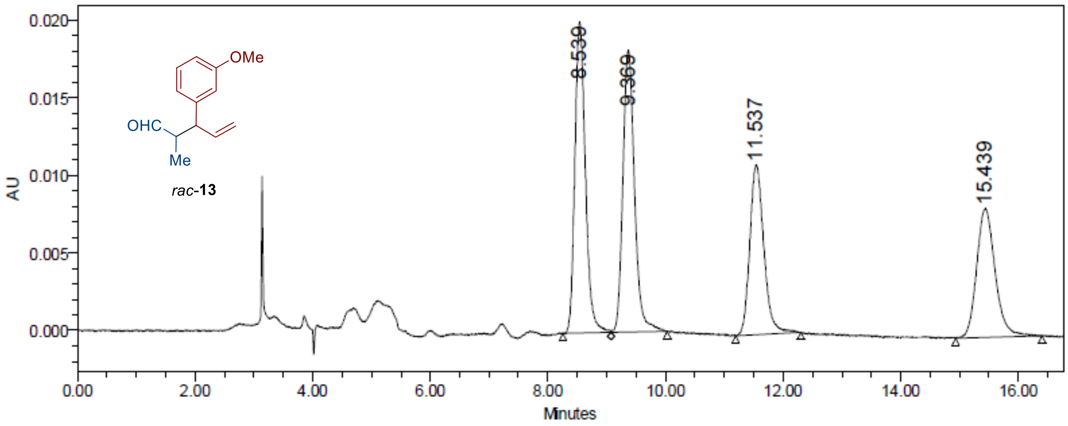

	RT	Area	% Area	Height
1	8.539	249675	28.96	20011
2	9.369	248647	28.85	18161
3	11.537	182412	21.16	10944
4	15.439	181272	21.03	8295

(*R*,*S*)-**13**

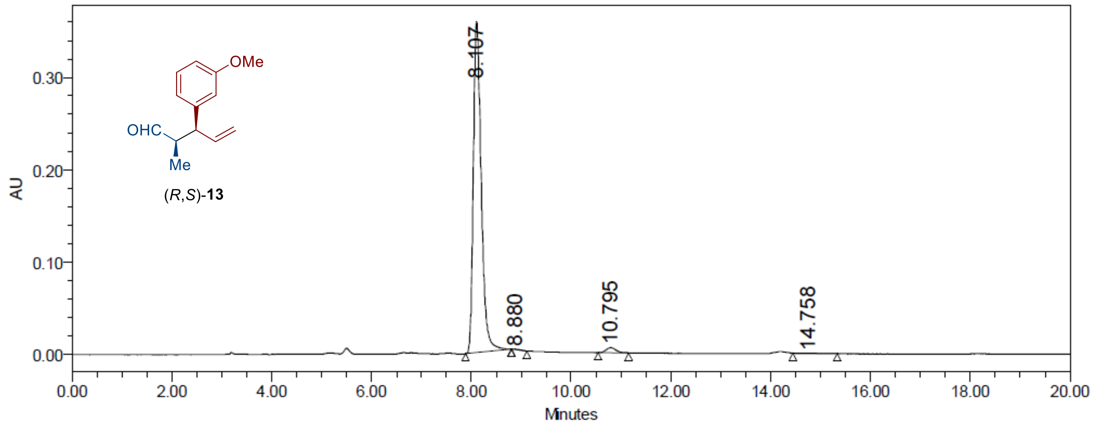

	RT	Area	% Area	Height
1	8.107	3884327	97.78	357961
2	8.880	2284	0.06	328
3	10.795	78234	1.97	5687
4	14.758	7706	0.19	-276

Supplementary Figure 140. HPLC spectra of compound **13**

rac-**14**

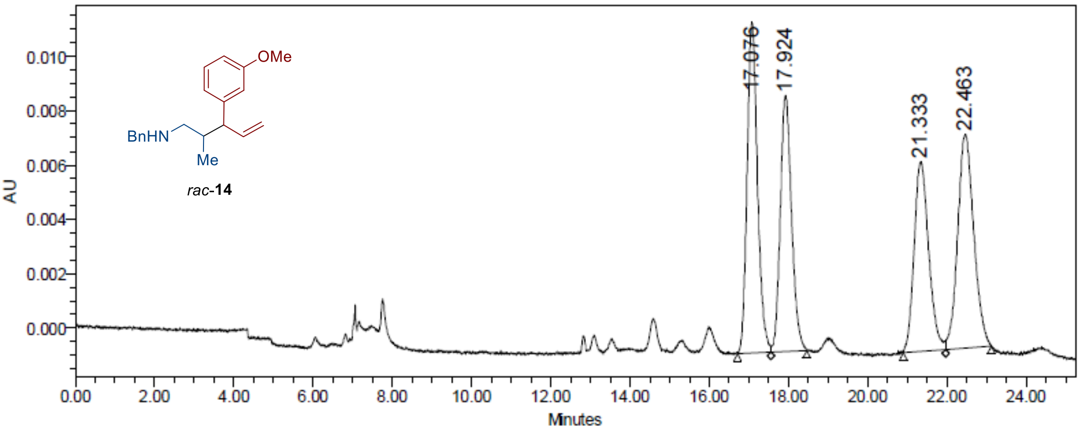

	RT	Area	% Area	Height
1	17.076	222241	27.39	12166
2	17.924	187283	23.09	9431
3	21.333	178706	22.03	6985
4	22.463	223041	27.49	7872

(*R*,*S*)-**14**

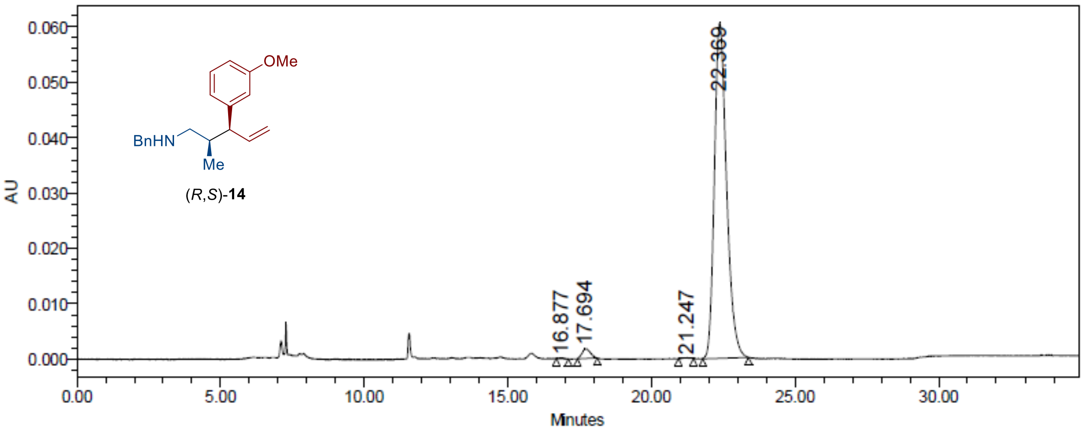

	RT	Area	% Area	Height
1	16.877	1129	0.06	124
2	17.694	36056	1.99	1788
3	21.247	2038	0.11	153
4	22.369	1769227	97.83	60514

Supplementary Figure 141. HPLC spectra of compound **14**

rac-**15**

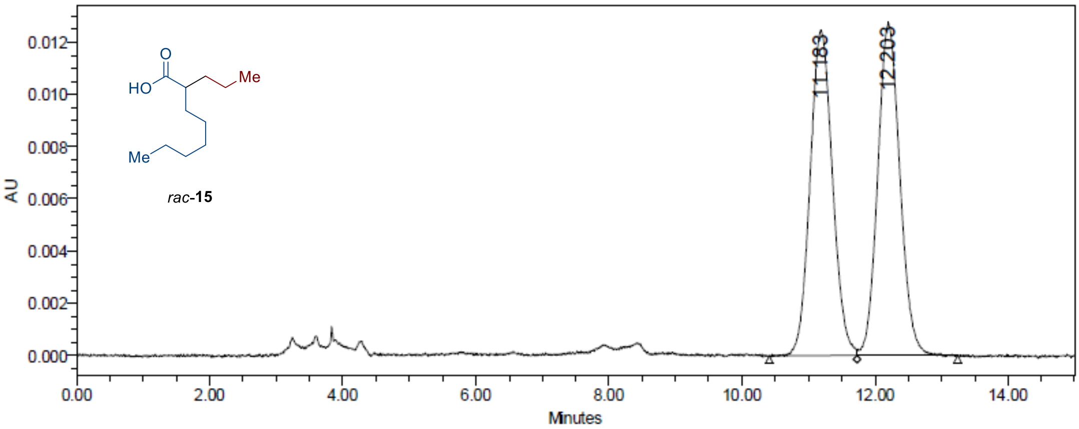

	RT	Area	% Area	Height
1	11.183	291445	49.76	12450
2	12.203	294228	50.24	12754

(*R*)-**15**

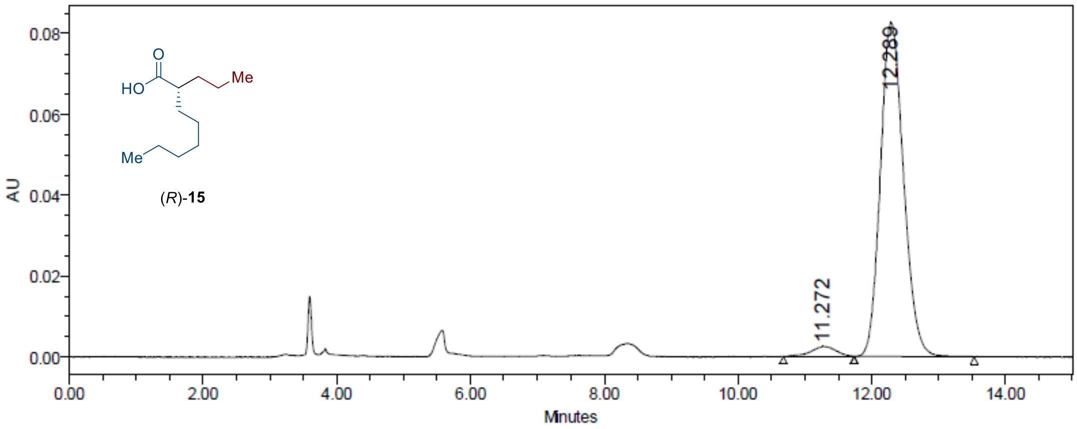

	RT	Area	% Area	Height
1	11.272	63127	3.09	2443
2	12.289	1980977	96.91	82615

Supplementary Figure 142. HPLC spectra of compound **15**

rac-**16**

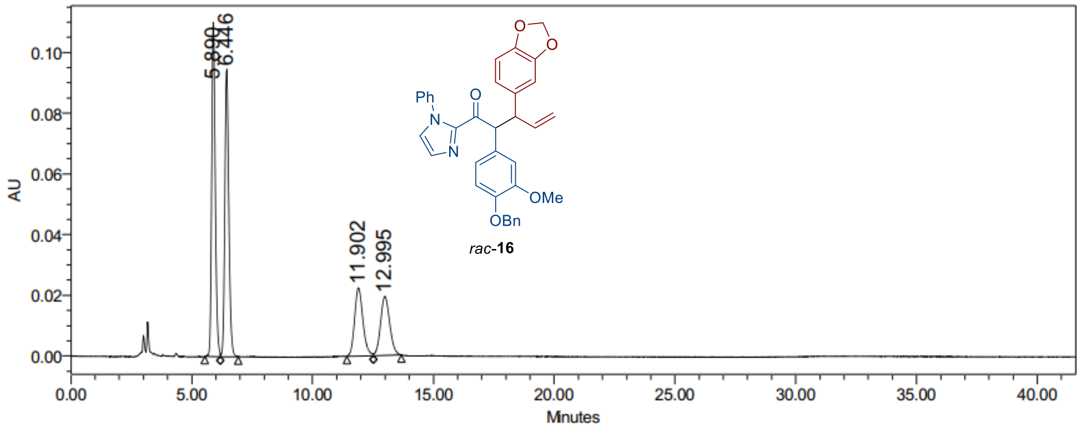

	RT	Area	% Area	Height
1	5.890	1130709	34.63	110150
2	6.446	1080710	33.10	94661
3	11.902	537076	16.45	22467
4	12.995	516358	15.82	19454

(*S*,*S*)-**16**

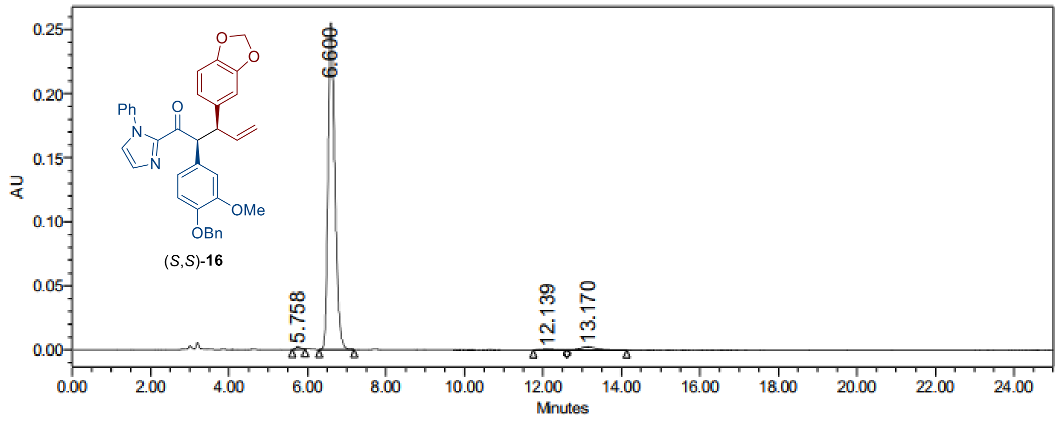

	RT	Area	% Area	Height
1	5.758	16032	0.52	2115
2	6.600	3011550	96.96	255238
3	12.139	13362	0.43	562
4	13.170	65120	2.10	2397

Supplementary Figure 143. HPLC spectra of compound **16**

rac-**17**

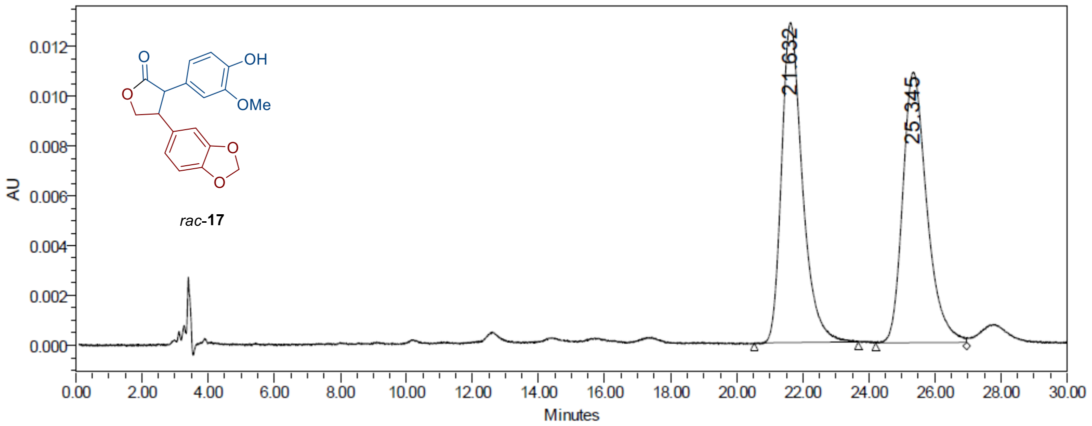

	RT	Area	% Area	Height
1	21.632	564123	50.57	12855
2	25.345	551437	49.43	10872

(*S*,*S*)-**17**

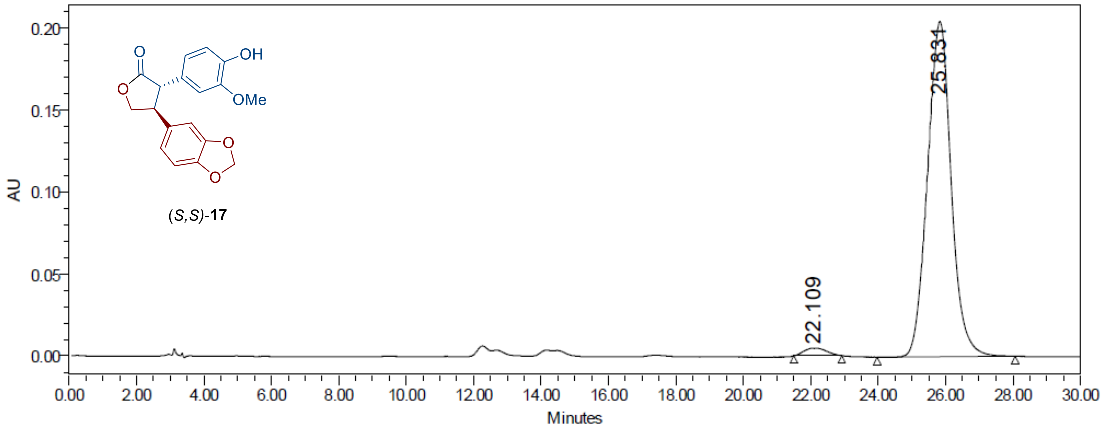

	RT	Area	% Area	Height
1	22.109	217707	2.14	4787
2	25.831	9937450	97.86	204187

Supplementary Figure 144. HPLC spectra of compound **17**

rac-**18**

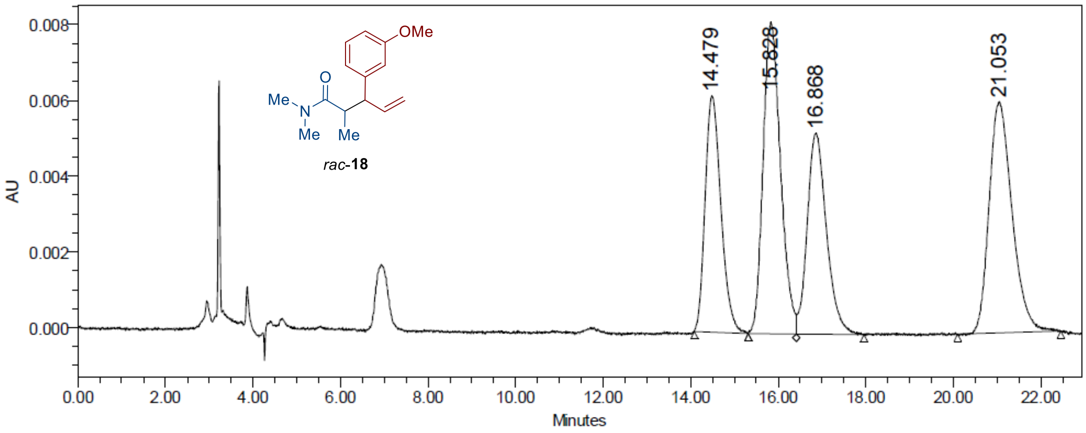

	RT	Area	% Area	Height
1	14.479	156499	20.31	6241
2	15.828	228876	29.70	8227
3	16.868	162439	21.08	5313
4	21.053	222785	28.91	6109

(*R*,*S*)-**18**

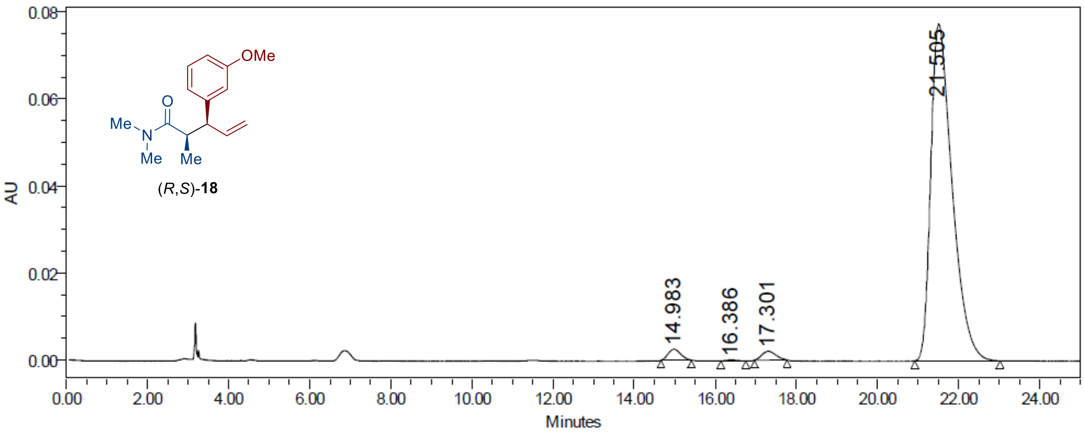

	RT	Area	% Area	Height
1	14.983	55444	1.89	2562
2	16.386	4644	0.16	252
3	17.301	49558	1.69	2065
4	21.505	2818584	96.26	77379

(*R*,*R*)-**18**

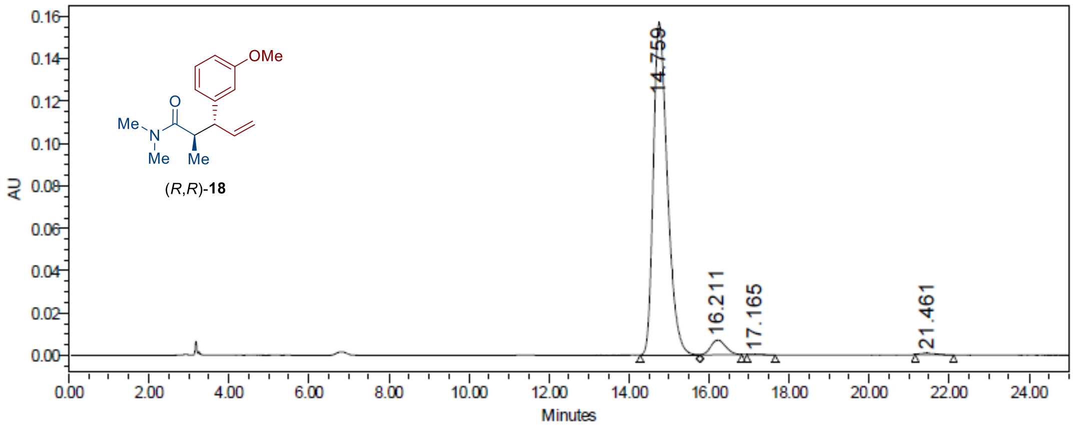

	RT	Area	% Area	Height
1	14.759	3855302	95.04	156777
2	16.211	177965	4.39	7018
3	17.165	3881	0.10	227
4	21.461	19436	0.48	749

(*S*,*R*)-**18**

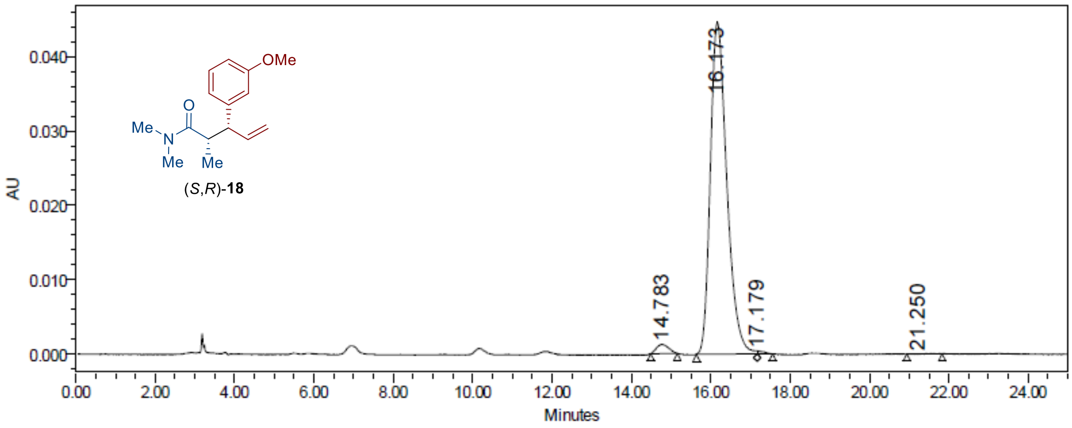

	RT	Area	% Area	Height
1	14.783	26366	2.00	1267
2	16.173	1286486	97.55	44572
3	17.179	4917	0.37	410
4	21.250	1044	0.08	-60

(*S*,*S*)-**18**

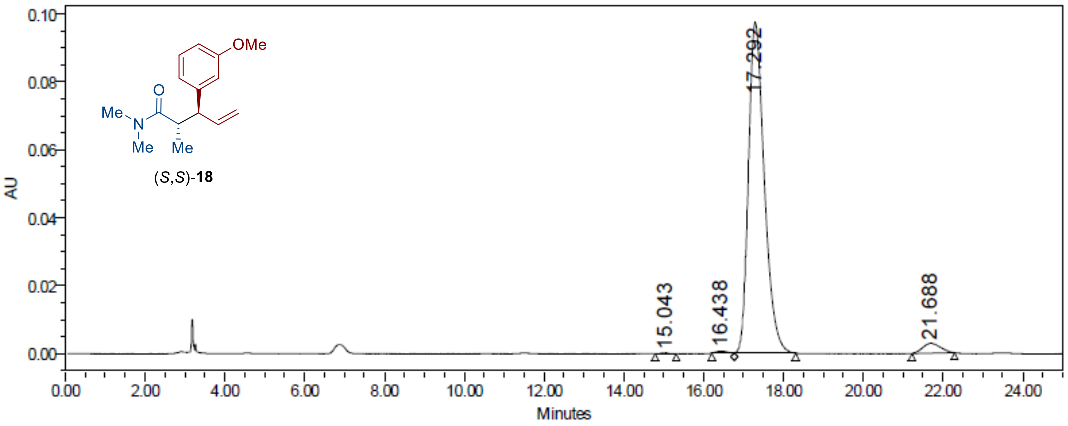

	RT	Area	% Area	Height
1	15.043	2809	0.10	175
2	16.438	10162	0.36	574
3	17.292	2692293	96.34	97257
4	21.688	89295	3.20	2862

Supplementary Figure 145. HPLC spectra of compound **18**

rac-**19**

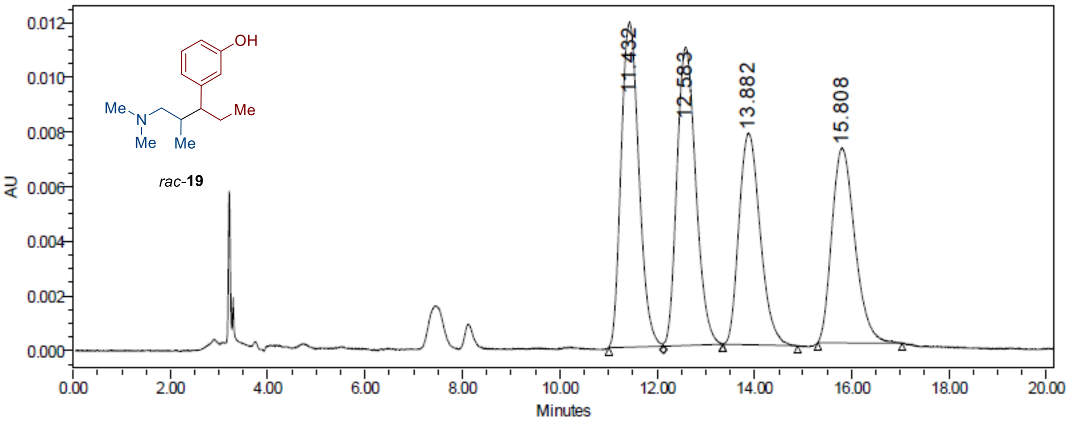

	RT	Area	% Area	Height
1	11.432	301770	28.26	11886
2	12.583	295545	27.68	10923
3	13.882	235062	22.01	7765
4	15.808	235386	22.04	7149

(*R*,*S*)-**19**

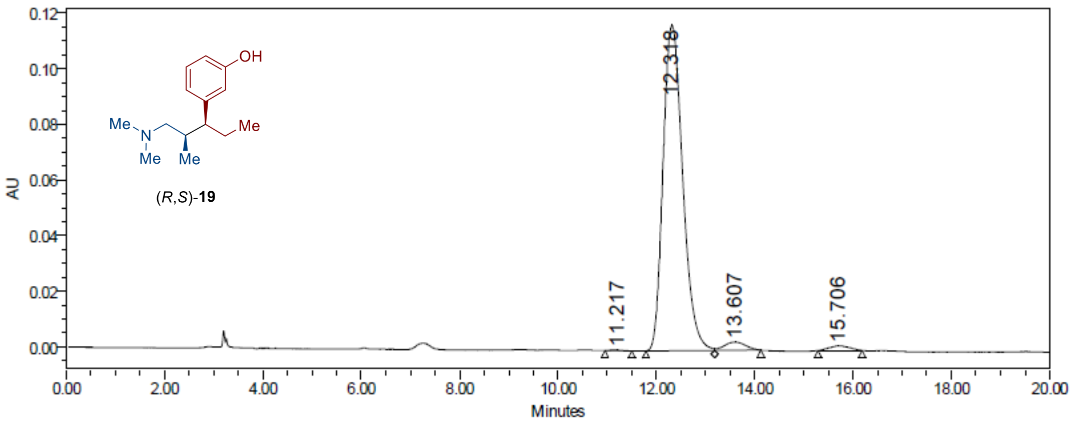

	RT	Area	% Area	Height
1	11.217	3145	0.09	207
2	12.318	3218096	95.84	116909
3	13.607	89544	2.67	2997
4	15.706	47111	1.40	1595

(*R*,*R*)-**19**

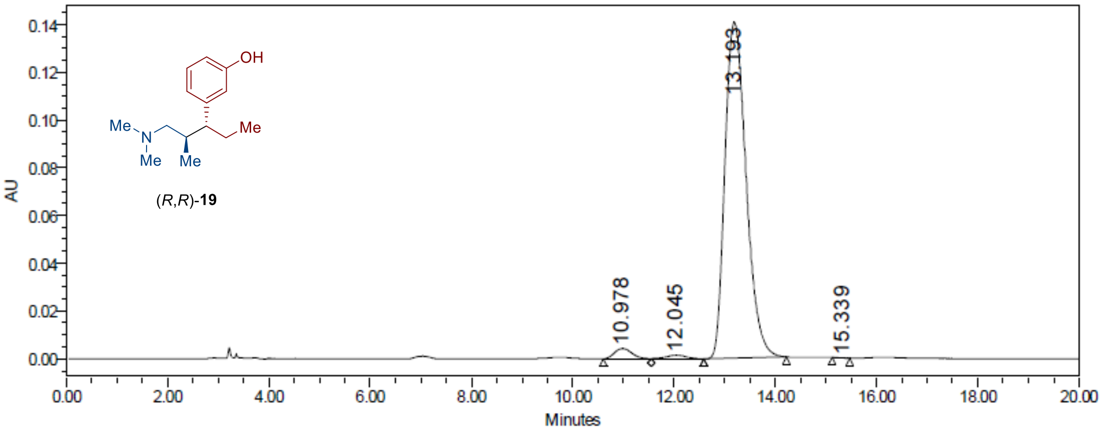

	RT	Area	% Area	Height
1	10.978	103807	2.46	4291
2	12.045	42956	1.02	1557
3	13.193	4080294	96.51	140483
4	15.339	591	0.01	-63

(*S*,*R*)-**19**

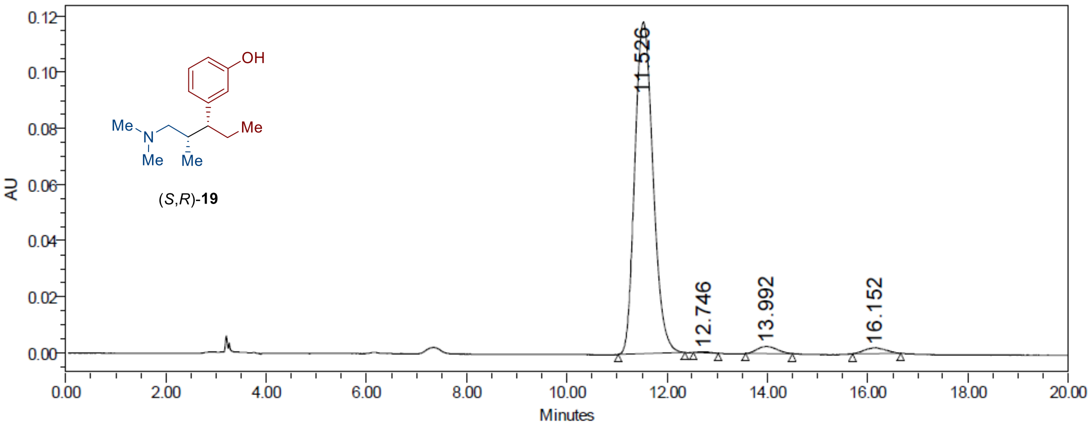

	RT	Area	% Area	Height
1	11.526	3011751	95.63	118158
2	12.746	5895	0.19	392
3	13.992	71332	2.26	2562
4	16.152	60555	1.92	1999

(*S*,*S*)-**19**

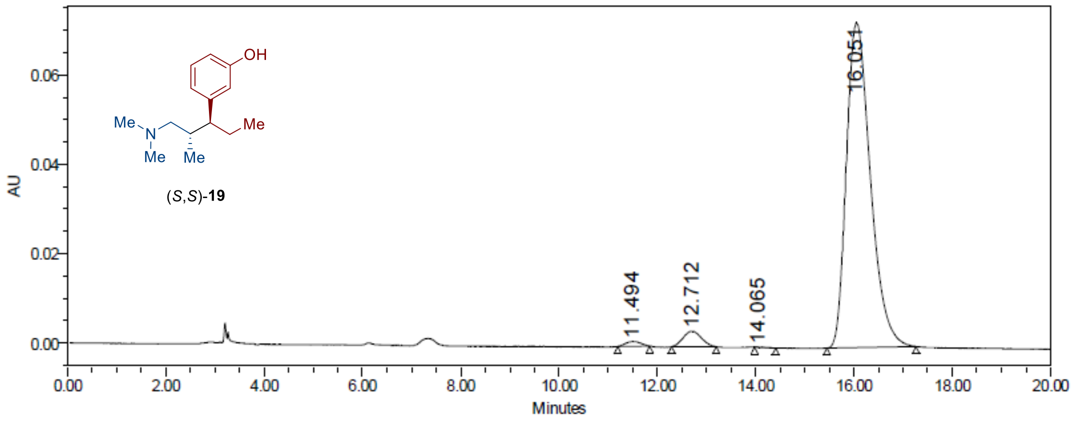

	RT	Area	% Area	Height
1	11.494	23058	0.90	1099
2	12.712	87324	3.40	3439
3	14.065	398	0.02	-52
4	16.051	2456555	95.69	72599

Supplementary Figure 146. HPLC spectra of compound **19**

4. Supplementary References

1. Wang, C., Zheng, Y., Huo H. & Meggers, E. Merger of visible light induced oxidation and enantioselective alkylation with a chiral iridium catalyst. *Chem. Eur. J.* **21**, 7355–7359 (2015**)**.
2. Gong, J., Li, S.-W., Qurban, S. & Kang, Q. Enantioselective Mannich reaction employing 1,3,5-triaryl-1,3,5-triazinanes catalyzed by chiral-at-metal rhodium complexes. *Eur. J. Org. Chem*. 3584–3593 (2017).
3. Dong, Y.-X., Zhang, C.-L., Gao, Z.-H. & Ye, S. Iminoacylation of alkenes via photoredox N-heterocyclic carbene catalysis. *Org. Lett.* **25**, 855–860 (2023).
4. Xiang, J.-C., Wang, Q. & Zhu, J. Radical-cation cascade to cryltetralin cyclic ether lignans under visible-light photoredox catalysis. *Angew. Chem. Int. Ed.* **59**, 21195–21202 (2020).
5. Spoehrle, S. S. M., West, T. H., Taylor, J. E., Slawin, A. M. Z. & Smith, A. D. Tandem palladium and isothiourea relay catalysis: Enantioselective synthesis of α-amino acid derivatives via allylic amination and [2,3]-sigmatropic rearrangement. *J. Am. Chem. Soc*. **139**, 11895–11902 (2017).
6. Zhang, Q., Chang, X. & Guo, C. Asymmetric Lewis acid catalyzed electrochemical alkylation. *Angew. Chem. Int. Ed*. **58**, 6999–7003 (2019).
